# Global, regional, and national burden of exposure to chewing tobacco, 1990–2023: a systematic analysis of the Global Burden of Disease Study 2023

**DOI:** 10.1016/j.langlo.2026.104043

**Published:** 2026-09-30

**Authors:** GBD 2023 Chewing Tobacco Collaborators, Gabriela Fernanda Gil, Jason A Anderson, Xiaochen Dai, Luisa S Flor, Oliver J McElvaney, Brooks W Morgan, Erin C Mullany, Erin M O'Connell, Bhoomadevi A, Hasan Aalruz, Rashad Abdul-Ghani, Rizwan Suliankatchi Abdulkader, Parisa Abedi, Olugbenga Olusola Abiodun, Olumide Abiodun, Nagah M Abourashed, Dariush Abtahi, Fuad Hamdi A Abuadas, Mohammad H Abuadas, Rana Kamal Abu-Farha, Ahmed Abu-Zaid, Anirudh Balakrishna Acharya, Apurba Acharya, Swetha Acharya, Babatope Oluwadamilare Adebiyi, Miracle Ayomikun Adesina, Juliana Bunmi Adetunji, Usha Adiga, Aanuoluwapo Adeyimika Afolabi, Muhammad Sohail Afzal, Feleke Doyore Agide, Aqeel Ahmad, Khabir Ahmad, Khurshid Ahmad, Sajjad Ahmad, Ayman Ahmed, Md Faisal Ahmed, Naveed Ahmed, Shahzaib Ahmed, Sara Akeel, Akil Akhtar, Tomi Akinyemiju, Omar Al Omari, Mohammad Al Qadire, Yazan Al Thaher, Mohammad Ahmmad Mahmoud Al Zoubi, Timothy Olukunle Aladelusi, Mohammed Sultan Al-Ak'hali, Mohammad Khursheed Alam, Rasmieh Mustafa Al-Amer, Turki M Alanzi, Shams Moh'd Fares Khalaf Al-Anzi, Ahmad M Al-Bashaireh, Mohammed S Aldossary, Raouf Alebshehy, Aqeel M Alenazi, Fahad D Algahtani, Abdelazeem M Algammal, Khalid F Alhabib, Fadwa Naji Alhalaiqa, Amidu Alhassan, Ashraf Alhumaidi, Inayat Ali, Irfan Ali, Kamran Ali, Mohammad Daud Ali, Rafat Ali, Syed Shujait Ali, Syed Mohamed Aljunid, Sameer Abdulmalik Alkubati, Wesam Taher Almagharbeh, Mohammed J Almalki, Sabah Al-Marwani, Margret Beaula Alocious Sukumar, Mahmoud A Alomari, Jaber S Alqahtani, Mohammad R Alqudimat, Ahmad Rajeh Al-Qudimat, Ahmed Yaseen Alqutaibi, Sahel Majed Alrousan, Mohammed Amjed Alsaegh, Najim Z Alshahrani, Ali Salman Al-Shami, Zaid Altaany, Awais Altaf, Hassan Alwafi, Yaser Mohammed Al-Worafi, Hany Aly, Mohammad Sharif Ibrahim Alyahya, Abdulmohsen Hamdan Al-Zalabani, Karem H Alzoubi, Adel S Al-Zubairi, Revati Amin, Tarek Tawfik Amin, Etsay Woldu Anbesu, Syam S Andra, Abhishek Anil, Ah Sruthi Anil Kumar, Prapti Anjana, Kabilan Annadurai, Saeid Anvari, Razique Anwer, Gururaj Arakeri, Aleksandr Y Aravkin, Harshit Arora, Monika Arora, Umesh Raj Aryal, Mohammad Ashfaque, Tahira Ashraf, Muhammad Shahzad Aslam, Yuni Asri, Anil Raj Assariparambil, Omer Atac, Seyyed Shamsadin Athari, Samuel Babajide Atoyebi, Alok Atreya, Avinash Aujayeb, Manal Awad, Kemal Lemnuro Awol, Ahmad Ayed, Syed Babar Jamal Bacha, Ashish D Badiye, Riadh Badraoui, Youngoh Bae, Nasser Bagheri, Khlood K Baghlaf, Abdul Rasheed Bahar, Yasemin Bahar, Razieh Bahreini, Parvathy Balachandran, Raju Balaji, Yatan Pal Singh Balhara, Biswajit Banik, Hiba Jawdat Barqawi, Amadou Barrow, Fadi Basha, Md Abu Bashar, Almaz Nibret Belay, Uzma Iqbal Belgaumi, Abdulrahman Babatunde Bello, Ibrahim Olajide Bello, Kelemu Zelalem Berhanu, Pankaj Bhardwaj, Sonu Bhaskar, Ashok Kumar Bhati, Sudip Bhattacharya, Bijit Biswas, Eyob Ketema Bogale, Archith Boloor, Dejana Braithwaite, Zahid A Butt, Senthilkumar C, Sanjay C J, Zhong Cao, Felix Carvalho, Márcia Carvalho, Sandra Carvalho, Joao Mauricio Castaldelli-Maia, Maria Sofia Cattaruzza, Sandip Chakraborty, Rama Mohan Chandika, Vijay Kumar Chattu, Sirshendu Chaudhuri, Akhilanand Chaurasia, Snehal Prakash Chavhan, Meng Xuan Chen, Saravanan Chinnaiyan, Bryan Chong, Sonali Gajanan Choudhari, Dinh-Toi Chu, Isaac Sunday Chukwu, Sunghyun Chung, Alexandru Corlateanu, Claudia Cosma, Natalia Cruz-Martins, Omid Dadras, Namrata Dagli, Gloria Dalla Costa, Mouni Damineni, Lalit Dandona, Rakhi Dandona, Samuel Demissie Darcho, Yagnik Dave, Marco Del Riccio, Ismail Dergaa, Vinoth Gnana Chellaiyan Devanbu, Syed Masudur Rahman Dewan, Arkadeep Dhali, Amol S Dhane, Bibha Dhungel, Kimia Didehvar, Hoa Thi Do, Pranavi Dohare, Sushil Dohare, Cátia Domingues, Jasmin Dönicke, Ojas Prakashbhai Doshi, Jiang Du, Emeka W Dumbili, Senbagam Duraisamy, Siddhartha Dutta, Kristina Edvardsson, Michael Ekholuenetale, Suhaib El Khatib, Iman El Sayed, Mohamed Ahmed Eladl, Omar Abdelsadek Abdou Elmeligy, Ashraf A El-Metwally, Abdelgawad Salah Eltahawy, Collins Nduka Esomchi, Elochukwu Ezenwankwo, Devaraj Ezhilarasan, Adeniyi Francis Fagbamigbe, Ali Faramarzi, Mohammad Fareed, Carla Sofia E Sá Farinha, Munazza Fatima, Alireza Feizkhah, Feten Fekih-Romdhane, Nuno Ferreira, Anteneh Fikrie, Filippos T Filippidis, Florian Fischer, James L Fisher, Muktar A Gadanya, Márió Gajdács, Balasankar Ganesan, Balasubramanian Ganesh, Shivaprakash Gangachannaiah, Fernando Jr Barroga Garcia, Rupesh K Gautam, Haftay Gebremedhin, Mizan Habtemichael Gebresilassie, Khalid Yaser Ghailan, Lobna Gharaibeh, Sailaja Ghimire, Sama Ghoba, Ehsan Gholami, Zainab Gholami, Thinh Gia Gia Nguyen, Artyom Urievich Gil, Gamze Gökulu, Pouya Goleij, Divya Gopinath, Michal Grivna, Bin Guan, Zenawi Hagos Gufue, Damitha Asanga Gunawardane, Sasidhar Gunturu, Anish Kumar Gupta, Bhawna Gupta, Himanshu Gupta, Prakash C Gupta, Rajeev Gupta, Sunanda Gupta, Yonas Deressa Guracho, Parham Habibzadeh, Yohannes Gebreegziabher Haile, Ferdous Hakim, Esam Halboub, Sajid Hameed, Nasrin Hanifi, Arief Hargono, Ahmed I Hasaballah, Faizul Hasan, Ikramul Hasan, Shamimul Hasan, Towhid Hasan, Nada Tawfig Hashim, Ahmad Shoeb Hashmi, Md Saquib Hasnain, Nageeb Hassan, Simon I Hay, Behzad Heibati, Mohammad Heidari, Zaw Myo Hein, Mohamed Fawzi Hemida, Brenda Yuliana Herrera-Serna, Yuta Hiraike, Ashley Mark Hopkins, Lubna Hossain, Sahadat Hossain, Mehdi Hosseinzade, Weijun Huang, Javid Hussain, Bing-Fang Hwang, Luigi Francesco Iannone, Segun Emmanuel Ibitoye, Olayinka Stephen Ilesanmi, Irena M Ilic, Milena D Ilic, Mohammad Tarique Imam, Mustapha Immurana, Lalu Muhammad Irham, CV Irshad, M Mofizul Islam, Farhad Islami, Juan S Izquierdo-Condoy, Jaison Jacob, Abhishek Jaiswal, Mihajlo Jakovljevic, Qazi Mohammad Sajid Jamal, Rajiv Janardhanan, Ruwan Duminda Jayasinghe, Achala Upendra Jayatilleke, Gwang Hun Jeong, Bijay Mukesh Jeswani, Zixiang Ji, Shuai Jin, Jobin Jose, Alex Joseph, Nitin Joseph, Charity Ehimwenma Joshua, Hema Shree K, Vaishali K, Billingsley Kaambwa, Zubair Kabir, Vidya Kadashetti, Dler Hussein Kadir, Pradnya Vishal Kakodkar, Ebbie Kalan, Mehnaz Kamal, Mona Kanaan, Jiseung Kang, Kehinde Kazeem Kanmodi, Surya Kant, Neeti Kapoor, Shalini Kapoor, Debasish Kar, Sujita Kumar Kar, Neila Karagezova, Mohmed Isaqali Karobari, Manoj Kumar Kashyap, Chukwudi Keke, Asaad Khalid, Maseer Khan, Moien Ab Khan, Muhammad Arslan Khan, Muhammad Umer Khan, Serab Khan, Sumaiya Khan, Zahid Khan, Zelal Kharaba, Sameer Uttamaro Khasbage, Samira Khoshvaght, Mahmood Khosrowjerdi, P Ratan Khuman, Min Seo Kim, Marta A Kisiel, Shivakumar KM, Prakash Babu Kodali, Oleksii Korzh, Archana Koul, Sindhura Lakshmi Koulmane Laxminarayana, Kewal Krishan, Abhishek Krishna, Deepak Krishnamurthy, Nitya Krishnasamy, Pranav S Kshtriya, Chong-Han Kua, Garry Kuan, Claudia E Kuehni, Omar Kujan, Mukhtar Kulimbet, Ashok Kumar, Dhasarathi Kumar, Diwakar Kumar, G Anil Kumar, Laksh Kumar, Mohan Kumar, Nithin Kumar, Pawan Kumar, Pradeep Kumar, Rakesh Kumar, Tushar Kumar, V Udaya Kumar, Om P Kurmi, Maria Dyah Kurniasari, Krishna Prasad Kurpad, Taeho Kwak, Chandrakant Lahariya, Hanpeng Lai, Timo Lajunen, Agung Dwi Laksono, Yilma Markos Larebo, Savita Lasrado, Munjae Lee, Seung Won Lee, Hui Li, Jiaying Li, Qiang Li, Si Li, Xingang Li, Zhengrui Li, Yanxue Lian, Daniel Lindholm, Bingsheng Liu, Gang Liu, Jue Liu, José Francisco López-Gil, Giancarlo Lucchetti, Abhilash Ludhiadch, Pincheng Luo, Hung N Luu, Miltiadis D Lytras, Monika Machoy-Rakoczy, Ahmed A Madfa, Samatar Abshir Mahamed, Mansour Adam Mahmoud, Kashish Malhotra, Lokesh Manjani, Farheen Mansoor, Miquel Martorell, Yasith Mathangasinghe, Medha Mathur, Neeta Mathur, Khurshid A Mattoo, Enkeleint A Mechili, John Robert Carabeo Medina, Jitendra Kumar Meena, Ravi Mehrotra, Vini Mehta, Satish Melwani, Aamir Raoof Memon, Godfred Antony Menezes, Ritesh G Menezes, Tarig H Merghani, Tomislav Mestrovic, Chamila Dinushi Kukulege Mettananda, Mohamed M M Metwally, Muhammad Agus Mikrajab, Giuseppe Minervini, Erkin M Mirrakhimov, Vinaytosh Mishra, Sanjeev Misra, Tridip Mitra, Farduus Ibraahim Mohamed, Nouh Saad Mohamed, Ibrahim Mohammadzadeh, Zaidan Mohammed, Syam Mohan, Nagaishwarya Moka, Ali H Mokdad, Hirbaye Mokona, Himel Mondal, Rafael Silveira Moreira, Rohith Motappa, Sumaira Mubarik, Amartya Mukhopadhyay, Malaisamy Muniyandi, Christopher J L Murray, Krishna Murti, Vignesh Murugan, David Musoke, Sathish Muthu, Karikalan Nagarajan, Kiran P P Nagdeo, Ganesh R Naik, Gopal Nambi, Vinay Nangia, Shumaila Nargus, Mohammad Zakaria Nassani, Zuhair S Natto, Biswa Prakash Nayak, Kinjal Nayak, Andrew Kamsoko Ndakotsu, Sabina Onyinye Nduaguba, Samata Nepal, Marie Ng, Cuong Tat Nguyen, Duy Cao Nguyen, Huong Lan Thi Nguyen, Trang Nguyen, Ali Nikoobar, Vikram Niranjan, Fred Nugen, Wahyu Pudji Nugraheni, Nazik Nurelhuda, Bogdan Oancea, Ramez M Odat, Fabio Massimo Oddi, Ismail A Odetokun, Olusegun Olatunji Ojedoyin, Osaretin Christabel Okonji, Andrew T Olagunju, Jeby Jose Olickal, Ahmed Omar Bali, Hope Onohuean, Miracle Chinonso Oparah, Michal Ordak, Augustus Osborne, Tope Oyelade, Mahesh P A, Alicia Padron-Monedero, Raul Felipe Palma-Alvarez, Alok Kumar Panda, Deepshikha Pande Katare, Ashok Pandey, Sanjib Raj Pandey, Ioannis Pantazopoulos, Shahina Pardhan, Tarang Parekh, Romil R Parikh, Arpit Parmar, Pragnesh Parmar, Sameena Parveen, Roberto Passera, Bhargav Umeshchandra Patel, Satyananda Patel, Shivani Patel, Vipin Patidar, Ashlesh Patil, Dimitrios Patoulias, Uttam Paudel, Shrikant Pawar, Saikiran Pendem, Prince Peprah, Gavin Pereira, Olumuyiwa James Peter, Tung Thanh Pham, Edoardo Pirera, Roman V Polibin, Ramesh Poluru, Thantrira Porntaveetus, Amit Porwal, Priyanka Porwal, Pranil Man Singh Pradhan, Elton Junio Sady Prates, Harsh Priya, Bharathi M Purohit, Jagadeesh Puvvula, Ibrahim Qattea, Raghu Anekal Radhakrishnan, Venkatraman Radhakrishnan, Yashpal Singh Raghav, Pracheth Raghuveer, Fakher Rahim, Sajjad Rahimi, Firman Suryadi Rahman, Md Ferdous Rahman, Mohammad Hifz Ur Rahman, Mosiur Rahman, Amita Rai, Sunil Kumar Raina, Jeffrey Pradeep Raj, Mahmoud Mohammed Ramadan, Majed Ramadan, Kadar Ramadhan, Shakthi Kumaran Ramasamy, Pramod W Ramteke, Lokesh Rana, Shailendra Singh Rana, Chhabi Lal Ranabhat, Kumuda Rao, Sowmya J Rao, Devarajan Rathish, Tapash Ranjan Rautray, Salman Rawaf, Abdul Rehman, Mina Rezaei, Nazila Rezaei, Ravi Rohilla, Susanne Röhr, Chanapong Rojanaworarit, Himanshu Sekhar Rout, Shubhanjali Roy, Rohini Ruhil, Chandan S N, Cameron John Sabet, Mamta Sachdeva Dhingra, Basema Ahmad Saddik, Mohammad Reza Saeb, Umar Saeed, Dominic Sagoe, Indranil Saha, Amirhossein Sahebkar, Vaibhav Sahni, Ambika Sahoo, Mirza Rizwan Sajid, Joseph W Sakshaug, Afeez Abolarinwa Salami, Mohamed A Saleh, Janmejaya Samal, Abdallah M Samy, Siddaram Shivaji Sarate, Aswini Saravanan, Yaser Sarikhani, Mohammad Sarmadi, Gargi Sachin Sarode, Sachin C Sarode, Mukesh Kumar Sathya Narayanan, Aanchal Satija, Shakta Mani Satyam, Ganesh Kumar Saya, Christophe Schinckus, Art Schuermans, Siddharthan Selvaraj, Mohammad H Semreen, Subramanian Senthilkumaran, Yashendra Sethi, Seyed Mohammad Seyed Alshohadaei, Ahmed Nabil Shaaban, Arash Shaban-Nejad, Muhammed Shabil, Sweni Shah, Sunder Sham, Muhammad Aaqib Shamim, Alfiya Shamsutdinova, Abhishek Shankar, Mohammed Shannawaz, Bhoopesh Kumar Sharma, Manoj Sharma, Ravi Kumar Sharma, Shreya Sharma, Vishal Sharma, Somia Shehzadi, Mahdi Sheikh, Mahabalesh Shetty, Pavanchand Shetty, Premalatha K Shetty, Hui-Zhong Shi, Arun Shirali, Shirindokht Shirazi, Kawkab Shishani, Aminu Shittu, Ivy Shiue, Ambreen Shoaib, Aman Shrestha, Sunil Shrestha, Dharmsheel Shrivastav, Kerem Shuval, Negussie Boti Sidamo, Abhinav Singh, Amit Singh, Bhim Pratap Singh, Brij Pal Singh, Jasvinder A Singh, Jawahar Singh, Poornima Suryanath Singh, Prashant Kumar Singh, Ragini D Singh, Surjit Singh, Virendra Singh, Gisha Sivan, Gowri Sivaramakrishnan, Valentin Yurievich Skryabin, Farrukh Sobia, Md Salman Sohel, Lencho Kajela Solbana, Solikhah Solikhah, Mansi Soni, Joan B Soriano, Michele Sorrentino, Chandrashekhar T Sreeramareddy, Vipul Srivastava, Paschalis Steiropoulos, Marjia Sultana, Zhong Sun, Shanthosh Priyan Sundaram, Johan Sundström, Jithin Surendran, Chandan Kumar Swain, Celine Tabche, Mohammad Tabish, Iman M Talaat, Avni Tambi, Mircea Tampa, Benjamin Kye Jyn Tan, Ingan Ukur Tarigan, Aigul Yelgondiyevna Tazhiyeva, Gebremaryam Temesgen, Reem Temsah, Masayuki Teramoto, Kavumpurathu Raman Thankappan, Rekha Thapar, Madi Tleshev, Thang Huu Tran, Domenico Trico, Aristidis Tsatsakis, Evangelia Eirini Tsermpini, Abdul Rohim Tualeka, Himayat Ullah, Brigid Unim, Bhaskaran Unnikrishnan, Abdulkadir Usman Sambo, Berna Uzun, Hande Uzunçıbuk, Rakesh V R, Sai Mahesh Vajjala, Zahir Vally, Jef Van Den Eynde, Joe Varghese, Siddhartha Alluri Varma, Sampara Vasishta, Vishnu Priya Veeraraghavan, Balachandar Vellingiri, U Venkatesh, Narayanaswamy Venketasubramanian, Akshaya Kumar Verma, Vignesh Vikram, David Villarreal-Zegarra, Liang Wang, Shu Wang, Yaojie Wang, Fei-Long Wei, Dakshitha Praneeth Wickramasinghe, Nuwan Darshana Wickramasinghe, Faustine Williams, Shulan Wu, Hong Xiao, Wanqing Xie, Zemenu Shiferaw Yadita, Kaiqi Yang, Renjulal Yesodharan, Arzu Yiğit, Vahit Yiğit, Biksegn Asrat Yirdaw, S Yogeshkumar, Dong Keon Yon, Naohiro Yonemoto, Chuanhua Yu, Monal Yuwanati, Muhammad Sohail Zafar, Emilia Zainal Abidin, Fathiah Zakham, Hamed Zandian, Michael Zastrozhin, Saad Zbiri, Mohammed G M Zeariya, Alemu Birara Zemariam, Eyael M Zeru, Haijun Zhang, Zhiqiang Zhang, Zhi-Jiang Zhang, Lei Zheng, Ming-Hua Zheng, Carl Zhou, Mohamed Ali Zoromba, Ahed H Zyoud, Emmanuela Gakidou

## Abstract

**Background:**

Exposure to chewing tobacco is a known health risk factor but has received less attention than smoking in global tobacco control efforts. As the evidence base linking chewing tobacco use to poor health grows, it is essential to closely track the changing use of chewing tobacco distinct from other forms of smokeless tobacco, as well as the estimated burden associated with its use. These data are essential to inform policy makers who seek to identify and avert the health burden associated with these dangerous products.

**Methods:**

We estimated the prevalence of chewing tobacco use and attributable health burden for individuals aged 15 years and older across 204 countries and territories by age and sex to produce temporal estimates from 1990 to 2023 as part of the Global Burden of Diseases, Injuries, and Risk Factors Study 2023. We leveraged self-reported survey data on use of any smokeless tobacco products spanning 188 countries globally to inform estimates of chewing tobacco use. Further, we undertook a systematic review and meta-analysis using the burden of proof methodology to generate associated relative risks. Together, these estimates served as inputs to generate cause-specific, location-specific, age-specific, sex-specific, and year-specific population-attributable fractions and the resulting attributable measures of health burden.

**Findings:**

In 2023, we estimate 241 million (95% uncertainty interval [UI] 165–355) users of chewing tobacco globally, a prevalence of 3·9% (95% UI 2·7–5·8) among individuals aged 15 years and older. Consumption was concentrated in south Asia, where India had 64·3% (56·1–71·8) of all male chewing tobacco users and 54·8% (47·1–62·4) of all female users. Bangladesh followed with 7·6% (6·0–9·6) and 19·0% (14·5–24·6) of all male and female chewing tobacco users, respectively. Globally, use peaked in older ages, with a prevalence of 5·1% (3·2–8·0) among people aged 60–64 years. Chewing tobacco use was linked to 251 thousand deaths (149–389) in 2023, 152 thousand (96·2–235) of which occurred in males and 99·7 thousand (53·2–164) in females. When adding years of life lost to disability, the total burden attributed to chewing tobacco use was 6·48 million (3·96–9·91) disability-adjusted life-years (DALYs) in 2023. This burden corresponds to 133·5 (85·8–201·8) DALYs per 100 000 males and 66·9 (35·6–109·9) DALYs per 100 000 females. The burden attributable to chewing tobacco was linked to six outcomes, the largest of which was stroke (55·9 [29·0–92·9] DALYs per 100 000 individuals attributable to chewing tobacco) followed by lip and oral cavity cancer (17·4 [11·3–24·3] DALYs per 100 000). However, the underlying epidemiological evidence ranges from moderate to weak.

**Interpretation:**

We observed little change over time in the prevalence of chewing tobacco use, accompanied by a growing burden of associated death and disability. This burden of stroke and head and neck cancers is seen most heavily in south Asia. Chewing tobacco remains an understudied risk factor of public health concern and requires signatories of the WHO Framework Convention on Tobacco Control to increase efforts to reduce exposure alongside those made to mitigate the harms of other tobacco products.

**Funding:**

Bloomberg Philanthropies and Gates Foundation.

## Introduction

Tobacco use, including smoked, smokeless, and inhaled tobacco, remains a leading modifiable risk factor for premature morbidity and mortality worldwide.^1^^,^^2^ Chewing tobacco—a form of masticated tobacco encompassing a range of products—is specifically consumed without combustion yet delivers nicotine and carcinogenic nitrosamines through direct mucosal absorption.^3^, ^4^, ^5^, ^6^ The use of chewing tobacco is concentrated in south and southeast Asia but is not limited to this geographical region,^7^ and chewing tobacco has been causally linked to multiple disease outcomes, including head and neck cancers and adverse cardiovascular health, through toxicological and epidemiological analyses.^4^^,^^8^^,^^9^

**Research in context**

**Evidence before this study**

To identify studies relevant to assessing the impact of chewing tobacco on global disease burden, we conducted a systematic literature search of Ovid MEDLINE and PubMed for articles published from database inception to May 26, 2025, using the search strategy (“chewing tobacco” OR “smokeless tobacco”) AND (“prevalence” OR “epidemiology”) AND (“disease burden” OR “mortality” OR “DALYs” OR “YLL” OR “disability-adjusted life-years”) AND (“global” OR “national” OR “regional”), without language or year restrictions. Overwhelmingly, previous studies have assessed the health burden of smokeless tobacco as an aggregate category without isolating the distinct burden from chewing tobacco. As such, specific burdens attributable to chewing tobacco had not been fully quantified. A 2006 publication linked the aggregate of smokeless tobacco to 4·7 million disability-adjusted life-years (DALYs) lost and 204 309 ischaemic heart disease deaths using risk estimates from the INTERHEART study. A 2015 study of 113 countries estimated that, in 2010, smokeless tobacco was responsible for 1·7 million DALYs lost and 62 283 deaths from cancers of the mouth, pharynx, and oesophagus. An updated study of 127 countries in 2020 showed a worsening burden in 2017, with an estimated 2·5 million DALYs lost and 90 791 deaths from head and neck cancers due to all smokeless tobacco products. To date, the only global study that distinguishes the burden of chewing tobacco from non-chewed forms of smokeless tobacco is the Global Burden of Diseases, Injuries, and Risk Factors Study (GBD). Moreover, a 2024 meta-analysis re-evaluated the strength of existing evidence on the association between chewing tobacco and health outcomes, both affirming the role of chewing tobacco as a unique and substantial health risk and enabling more robust estimates of disease burden.

**Added value of this study**

Despite being linked to numerous serious health risks, chewing tobacco use has received less scrutiny than the use of other tobacco-related products. In the GBD comparative risk assessment framework, chewing tobacco is thereby contextualised as a global health risk factor with estimates of attributable burden of disease for 204 countries and territories by age and sex from 1990 to 2023. This study represents the largest and most comprehensive analysis of chewing tobacco burden by age, sex, and time conducted to date. GBD 2023 leverages updated relative risks for health outcomes associated with chewing tobacco in previous GBD rounds, incorporates new risk–outcome associations identified through rigorous systematic reviews, and utilises nationally representative survey data. By incorporating updated health risks and a considerably larger number of data sources, the present analysis offers a substantial improvement upon previous GBD rounds.

**Implications of all the available evidence**

This study highlights the limited effect of tobacco control measures on chewing tobacco use and justifies increased emphasis on chewing tobacco products in such initiatives. Our findings also characterise regional variation in chewing tobacco and the disproportionate effect of chewing tobacco on low-income and middle-income countries. Taken together, the updated population-level data and refined methodology described in this study can be used by researchers and policy makers to inform focused regulatory and prevention strategies that limit the uptake of chewing tobacco, address its health consequences, and promote cessation. The resulting estimates also have implications for the timing of population-based interventions, particularly among children and young people, and in areas of social and economic disadvantage.

In contrast to combustible tobacco use, which has been studied extensively for more than half a century,^10^^,^^11^ chewing tobacco remains comparatively neglected in both research and policy. Major tobacco control initiatives and legislative measures have emphasised the hazards of first-hand and second-hand smoke and have focused on public bans, taxation, and marketing restrictions for cigarettes without covering non-combustible tobacco products.^12^^,^^13^ Meanwhile, smokeless forms of tobacco remain socially visible and often culturally embedded,^14^^,^^15^ with aggressive marketing directed toward young people.^16^^,^^17^ The focus of the policy on smoking is also mirrored in the scientific literature. The absence of comprehensive regulation and robust chewing tobacco-specific data, combined with conceptions that chewing is less harmful than smoking,^18^^,^^19^ has facilitated sustained use across generations.

To design effective health policy, reliable estimates of prevalence, exposure patterns, and attributable disease burden specific to chewing tobacco are required. The efforts to date to quantify this burden reveal that more than 273 million adults use chewing tobacco^7^ and that millions of disability-adjusted life-years (DALYs) annually are linked to smokeless tobacco.^20^ The current evidence base is constrained by the low number of outcomes studied and the aggregation of disparate products under the umbrella of smokeless tobacco, despite differing chemical composition, nitrosamine content, and pH of these products.^3^^,^^21^^,^^22^ These gaps and ambiguity in the available data might obscure the magnitude of population-level risk where data are weakest, paradoxically under-representing the true burden in the very regions where chewing tobacco is most common.

In this context, an updated and methodologically rigorous reassessment of the global burden of disease attributable to chewing tobacco is warranted. By applying improved exposure data, updated and refined cause-specific risk estimates, and enhanced geographical resolution beyond the typical regions of interest, the Global Burden of Disease Study (GBD) 2023 can augment previous analyses and provide the empirical foundation needed for tobacco control policy worldwide. This manuscript was produced with contributions from the GBD Collaborator Network and in accordance with the GBD Protocol.^23^

## Methods

### Overview

We estimated the burden of disease attributable to use of chewing tobacco utilising the GBD comparative risk assessment framework. We defined current exposure to chewing tobacco as daily or occasional use of a masticated tobacco product within the past 30 days. Here, we summarise the key analytical steps: (1) estimating prevalence of exposure to chewing tobacco by age, sex, geography, and time, (2) estimating the relative risk of health outcomes causally linked with use of chewing tobacco, and (3) calculating population-attributable fractions (PAFs) for males and females, separately, in each 5-year age bin from age 15–19 years to ≥95 years. As part of GBD 2023, these PAFs and resulting attributable burden were produced for 204 countries and territories, 21 regions, seven super-regions, and globally, for every year between 1990 and 2023. The full GBD location hierarchy, populations, and age standards are in appendix 1 (pp 3–8, 11–18). This study adheres to the GATHER statement (appendix 1 pp 8–11).^24^

### Prevalence of chewing tobacco use

For GBD 2023, we maintained the same estimation process used for chewing tobacco exposure in previous GBD rounds, which have been described in detail elsewhere.^7^ Briefly, there are three primary steps to the estimation process: (1) data cleaning, (2) adjustment of smokeless tobacco data to reflect chewing tobacco, and (3) final spatiotemporal Gaussian process regression (ST-GPR). We identified geographically representative surveys with self-reported data on the use of any smokeless tobacco product among those catalogued in the Global Health Data Exchange. We aimed to capture current use as defined within the surveys. More details on our inclusion and exclusion criteria have been published elsewhere (appendix 1 pp 18–19).^1^^,^^7^ The present manuscript updates previous prevalence estimates with 799 total surveys (75% from low-income and middle-income countries), including 103 new sources spanning three more locations with at least one data input and 19 surveys from years more recent than 2019 (appendix 2 p 1). Included surveys can be found in the GBD 2023 Data Input Sources Tool.^25^

The initial step in cleaning extracted prevalence surveys was mapping data to any current smokeless tobacco product use (n=711) or, when specific to a product, to one of two mutually exclusive groups: chewed tobacco products (n=229) or non-chewed tobacco products (n=200). A single survey might include data on more than one type of tobacco; therefore, source counts are not exclusive. The non-chewing tobacco data informed an intermediate step of the estimation process—parsing out the distribution of chewing tobacco versus non-chewing tobacco products that contribute to all smokeless tobacco use (appendix 2 pp 2–6, 7)—but did not directly inform our final estimates. The products included in each of these categories can be found in appendix 1 (pp 19–24) alongside methods used to generate the adjustments. Using both the adjusted smokeless tobacco datapoints and the chewing tobacco-specific data, we ran a final ST-GPR model that produced estimates of chewing tobacco use by age, sex, location, and year. Details on ST-GPR are described in full elsewhere and provided in appendix 1 (p 25).^1^

### Health risks associated with chewing tobacco

The relative risks used in GBD 2023 to associate chewing tobacco use with health outcomes and the data and methods used to derive them have been published elsewhere and present a substantial improvement to previous GBD cycles.^1^^,^^9^^,^^26^ Briefly, these relative risks are the product of systematic reviews of peer-reviewed literature indexed in PubMed, Global Index Medicus, and Web of Science between Jan 1, 1970, and Jan 30, 2023 (appendix 1 pp 25–26). In our systematic reviews, we aimed to identify all cohort or case–control studies from generalisable populations that reported on the association between chewing tobacco (or a chewing tobacco product) and relevant health outcomes. PRISMA diagrams, detailed inclusion and exclusion criteria, search strings, and review protocol can be found in the previous publication of these analyses.^9^ Of 4591 records screened, we identified 111 publications reporting on 103 cohorts or case–control studies with all the necessary data. The full list of sources can be found online on the Burden of Proof visualisation tool.^27^

Leveraging this expanded dataset, we derived updated relative risk estimates for lip and oral cavity cancer and oesophageal cancer and new relative risk estimates for stroke, ischaemic heart disease, nasopharyngeal cancer, other pharyngeal cancer, and laryngeal cancer using the burden of proof meta-analytic methodology.^26^ Details on this methodology, including chewing tobacco-specific results, have been published elsewhere.^9^ Compared with traditional meta-analytic approaches, the burden of proof methodology more systematically addresses outlier detection, bias correction, and accounting for within-study and between-study heterogeneity (appendix 1 pp 26–27).^9^^,^^26^ Finally, the burden of proof methodology produces measures of the health risks associated with chewing tobacco and the strength of the available evidence (appendix 2 p 8).^9^^,^^27^

Risk–outcome pairs are eligible for inclusion in the GBD if there is sufficient evidence of a statistically significant association between the risk factor and the outcome. The association between chewing tobacco and ischaemic heart disease did not meet this threshold (appendix 2 p 8), but all the other risk–outcome pairs explored did.^9^ Consequently, GBD 2023 estimates of chewing tobacco-attributable disease burden are informed by relative risk estimates for both of the two health outcomes associated with chewing tobacco in GBD 2021 and four new health outcomes. These relative risks do not vary by sex, age, geography, or time. When sufficient data were available to evaluate potential differences in risk by sex, we derived sex-specific relative risks (appendix 1 pp 27–28) but found that they had overlapping uncertainties and, consequently, opted to use the more data-rich sex-agnostic relative risks.

### PAFs

To produce the burden attributable to the use of chewing tobacco products, PAFs were estimated using the prevalence and relative risk values and applied to GBD estimates of outcome-specific burden. PAFs are the difference between disease burden at current levels of chewing tobacco and the burden that would have occurred with no chewing tobacco (appendix 1 pp 28–29). Following standard GBD methodology that accounts for the simultaneous joint burden of health risk factors,^1^ the PAFs were then multiplied by the estimated DALYs and deaths associated with each outcome to produce the attributable chewing tobacco DALYs and deaths. These metrics are reported as the mean, and 2·5th and 97·5th percentiles of 250 draws of each posterior distribution to propagate statistical uncertainty. Differences between the years 1990 and 2023 were considered significant if the 95% uncertainty interval (UI) of the difference did not cross the null. Counts are presented to three significant figures, and percentages and rates are presented to one decimal place.

### Role of the funding source

The funders of the study had no role in study design, data collection, data analysis, data interpretation, or writing of the report.

## Results

### Prevalence

In 2023, there were 241 million (95% UI 165–355) people consuming chewing tobacco globally, translating to a prevalence of 3·9% (95% UI 2·7–5·8) among individuals aged 15 years and older (table 1). The use of chewing tobacco was more prevalent in males (5·4% [3·9–7·5]) than females (2·5% [1·4–4·2]; table 1). The largest number of chewing tobacco users was observed in south Asia, where 14·6% (10·2–20·9) of the population aged 15 years and older used chewing tobacco products (table 1; figure 1; appendix 2 pp 9–23). The country with the largest number of chewing tobacco users was India with 105 million (79·4–139) male and 42·9 million (23·4–72·6) female users, followed by Bangladesh (12·2 million [9·96–15·1] males and 14·4 million [9·53–20·6] females) and Pakistan (10·4 million [5·84–17·7] males and 2·77 million [1·17–5·55] females; appendix 2 pp 9–23). Notably, the highest prevalence of chewing tobacco use among males was observed in Nepal, with 30·6% (22·4–40·2) age-standardised prevalence, followed by Palau (23·6% [13·5–36·9]), Bhutan (20·4% [12·8–30·7]), and Bangladesh (20·9% [17·1–25·7]; figure 1; appendix 2 pp 24–51). Among females, the highest prevalence was observed in Palau (25·5% [13·8–40·8]) and Bangladesh (26·3% [17·7–36·9]; figure 1; appendix 2 pp 24–51).

**Table 1:** Prevalence of chewing tobacco use among individuals aged 15 years and older by super-region and sex, 1990 and 2023

	Overall chewing tobacco prevalence			Chewing tobacco prevalence among females			Chewing tobacco prevalence among males		
	1990	2023	Percentage change	1990	2023	Percentage change	1990	2023	Percentage change
Global	4·3% (2·4 to 7·0)	3·9% (2·7 to 5·8)	–7·7% (–49·8 to 88·4)	2·6% (1·3 to 4·6)	2·5% (1·4 to 4·2)	–5·7% (–58·1 to 136·5)	5·9% (3·4 to 9·3)	5·4% (3·9 to 7·5)	–8·9% (–46·8 to 71·7)
Central Europe, eastern Europe, and central Asia	0·4% (0·2 to 0·9)	0·6% (0·3 to 1·2)	43·2% (–50·2 to 372·4)	0·3% (0·1 to 0·6)	0·3% (0·1 to 0·7)	15·3% (–62·8 to 302·7)	0·6% (0·2 to 1·2)	0·9% (0·4 to 1·8)	54·6% (–45·2 to 383·6)
High income	0·9% (0·5 to 1·5)	1·1% (0·6 to 1·7)	15·6% (–44·5 to 165·8)	0·2% (0·1 to 0·5)	0·4% (0·1 to 0·8)	47·1% (–52·2 to 408·7)	1·6% (1·0 to 2·5)	1·8% (1·1 to 2·7)	8·0% (–44·3 to 127·1)
Latin America and Caribbean	0·5% (0·2 to 1·0)	0·5% (0·2 to 1·0)	0·1% (–66·3 to 242·3)	0·4% (0·1 to 0·9)	0·4% (0·1 to 0·8)	–3·4% (–68·6 to 239·8)	0·5% (0·2 to 1·2)	0·6% (0·2 to 1·2)	2·8% (–64·5 to 239·3)
North Africa and Middle East	1·1% (0·5 to 2·2)	1·0% (0·5 to 1·9)	–9·3% (–63·2 to 163·7)	0·6% (0·2 to 1·3)	0·5% (0·2 to 1·1)	–13·0% (–67·9 to 173·4)	1·6% (0·7 to 3·1)	1·4% (0·7 to 2·6)	–8·9% (–62·2 to 156·9)
South Asia	20·1% (10·9 to 32·2)	14·6% (10·2 to 20·9)	–27·3% (–60·6 to 43·6)	13·3% (6·7 to 22·8)	9·7% (5·6 to 15·7)	–27·4% (–67·1 to 68·2)	26·4% (14·9 to 40·8)	19·5% (14·6 to 26·2)	–26·3% (–57·2 to 39·4)
Southeast Asia, east Asia, and Oceania	1·2% (0·5 to 2·4)	1·4% (0·6 to 2·6)	14·4% (–56·7 to 247·9)	1·0% (0·4 to 2·1)	1·0% (0·5 to 2·1)	2·6% (–61·4 to 221·1)	1·3% (0·5 to 2·6)	1·7% (0·7 to 3·1)	23·2% (–54·1 to 273·0)
Sub-Saharan Africa	1·2% (0·5 to 2·5)	1·1% (0·5 to 2·2)	–8·0% (–65·8 to 186·2)	1·1% (0·4 to 2·2)	1·0% (0·4 to 1·9)	–8·5% (–67·6 to 206·1)	1·4% (0·6 to 2·8)	1·3% (0·6 to 2·4)	–7·0% (–64·1 to 177·8)

Data in parentheses are 95% uncertainty intervals. The age-standardised prevalence for more granular locations is in appendix 2.

**Figure 1:**
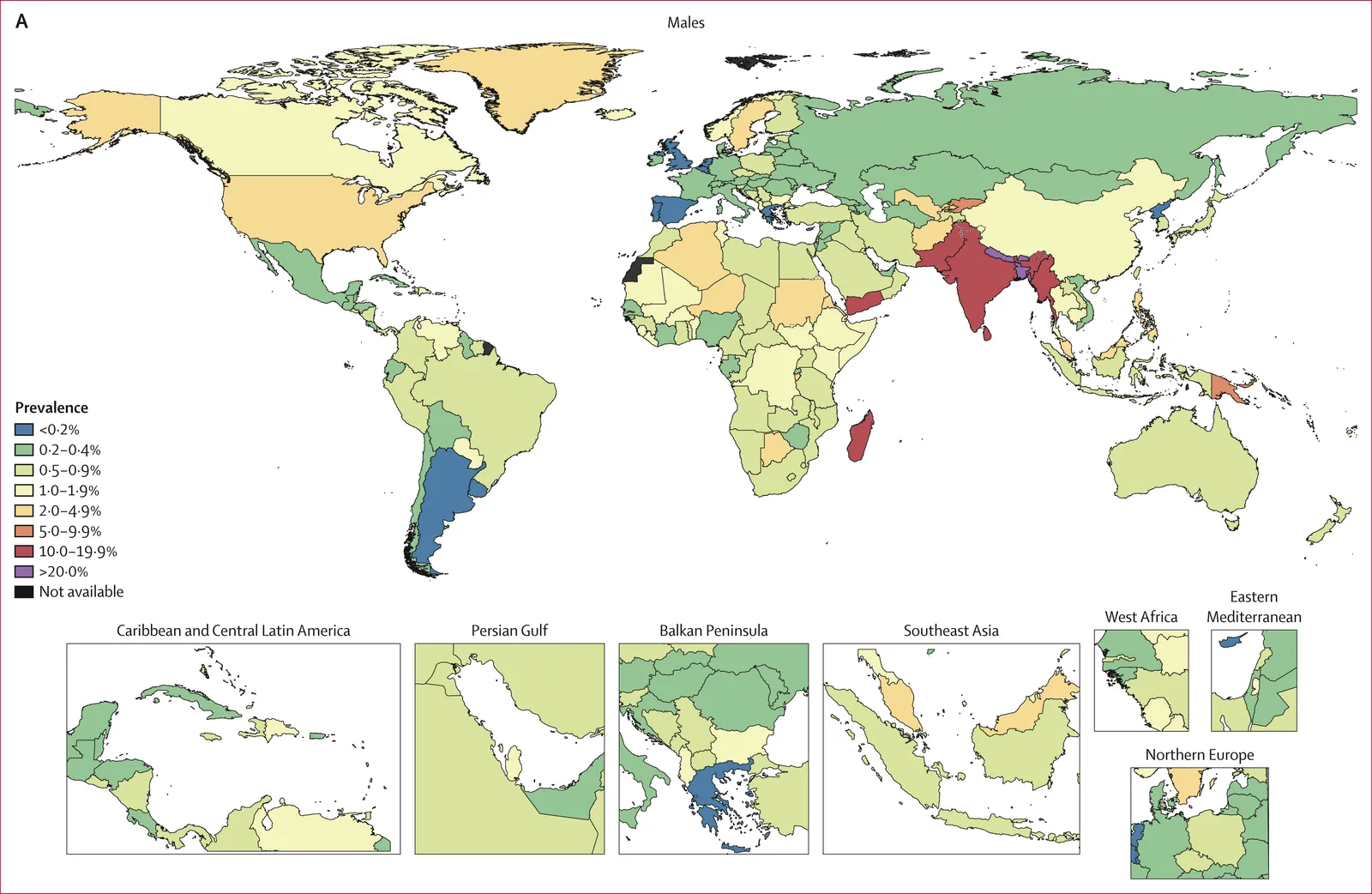

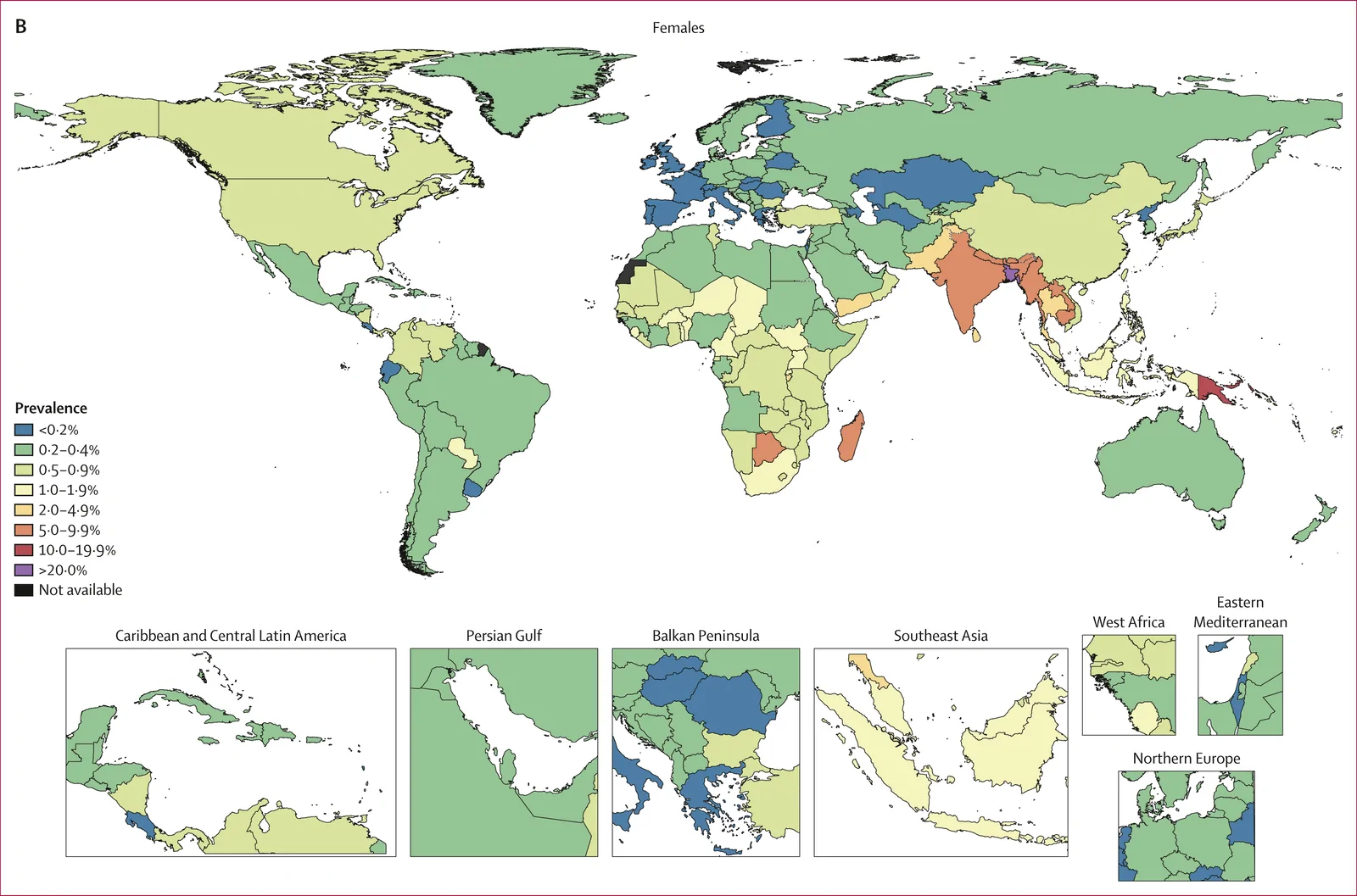
Age-standardised prevalence of chewing tobacco use in males (A) and females (B), among individuals aged 15 years and older, 2023 The countries in these maps are coloured in accordance with the age-standardised prevalence of chewing tobacco use by sex for people aged 15 years and older with red and purple indicating higher prevalence and blue indicating lower prevalence. Chewing tobacco use is specifically defined as current use of masticated tobacco products. The country-specific age-standardised prevalence estimates and prevalence estimates for people aged 15 years and older are in appendix 2 (pp 24–51).

Globally, the consumption of chewing tobacco begins to rise around the age of 20 years to 24 years with an estimated prevalence of 2·9% (95% UI 1·6–4·9) and peak around the age of 60 years to 64 years at a prevalence of 5·1% (3·2–8·0; appendix 2 p 52). However, in south Asia, males had the highest prevalence of chewing tobacco consumption of 27·3% (17·3–40·2) at the age of 60 years to 64 years, but the peak of chewing tobacco use for females was observed at ages older than 80 years with an estimated prevalence of 21·8% (12·9–33·7). Bangladesh stands out as a country where chewing tobacco prevalence among females is higher among all age groups older than 30 years relative to males (appendix 2 p 52). This difference is still most marked among Bangladeshi individuals older than 80 years where female prevalence (55·7% [40·1–71·4]) of chewing tobacco product use was over double that of males (26·8% [16·4–40·4]).

Between 1990 and 2023, the number of chewing tobacco users was estimated to have a non-significant increase of 90·7 million (95% UI –26·7 to 220) users (appendix 2 pp 9–23). No countries saw a significant decrease in chewing tobacco use, and 24 countries had significant percent increases in the number of users (appendix 2 pp 9–23). The largest absolute increase was observed in Bangladesh with 15·0 million (4·95 to 25·2) new chewing tobacco users between 1990 and 2023. Nepal, Madagascar, and Yemen each also had an increase of over 500 000 chewing tobacco users from 1990 to 2023 (appendix 2 p 53). Simultaneously, age-standardised prevalence remained constant with a change of –7·7% (–49·8 to 88·4) globally between 1990 and 2023 (table 1). All countries saw non-significant changes in age-standardised chewing tobacco prevalence from 1990 to 2023 except for males in Estonia, where prevalence consistently remained below 1% (appendix 2 pp 38–51).

### Attributable burden

Chewing tobacco use resulted in 251 thousand deaths (95% UI 149–389) in 2023, 152 thousand (96·2–235) of which occurred in males (table 2; appendix 2 pp 54–82). An estimated 210 thousand (128–318) of these chewing tobacco-attributable deaths occurred in south Asia (table 2), including 24·9 (15·8–37·6) deaths per 100 000 males and 16·5 (9·0–26·8) deaths per 100 000 females (appendix 2 pp 69–82). South Asia additionally drove the observed global increase in the number of attributable deaths by 134·2% (38·7–321·0) among males and 133·0% (19·8–317·1) among females (table 2). During the same period, age-standardised mortality rates have remained largely unchanged globally (appendix 2 pp 69–82). Importantly, some of the highest burden countries fell outside of south Asia, with over 90% of all male and female chewing tobacco-related deaths in India, Bangladesh, Pakistan, China, Thailand, USA, Myanmar, Nepal, Madagascar, Sri Lanka, Philippines, and Indonesia in 2023 (table 3; appendix 2 pp 54–68).

**Table 2:** Number of chewing tobacco-attributable deaths among individuals aged 15 years and older by super-region and sex, 1990 and 2023

	Overall deaths attributable to chewing tobacco (thousands)			Chewing tobacco deaths among females (thousands)			Chewing tobacco deaths among males (thousands)		
	1990	2023	Percentage change	1990	2023	Percentage change	1990	2023	Percentage change
Global	108 (55·4 to 177)	251 (149 to 389)	133·7% (31·9 to 311·6)	42·8 (21·8 to 78·6)	99·7 (53·2 to 164)	133·0% (19·8 to 317·1)	64·7 (33·5 to 108)	152 (96·2 to 235)	134·2% (38·7 to 321·0)
Central Europe, eastern Europe, and central Asia	1·32 (0·453 to 3·00)	1·32 (0·434 to 2·92)	0·0% (–64·2 to 210·4)	0·550 (0·174 to 1·30)	0·450 (0·140 to 1·07)	–18·1% (–71·2 to 168·1)	0·765 (0·290 to 1·68)	0·865 (0·296 to 1·86)	13·0% (–59·4 to 238·4)
High income	4·92 (2·80 to 8·16)	5·23 (3·34 to 8·77)	6·4% (–37·0 to 90·9)	0·755 (0·250 to 1·74)	0·862 (0·276 to 2·01)	14·2% (–59·2 to 262·8)	4·16 (2·55 to 6·45)	4·37 (2·84 to 6·98)	4·9% (–35·3 to 72·8)
Latin America and Caribbean	0·670 (0·236 to 1·53)	1·22 (0·406 to 2·73)	82·2% (–34·6 to 477·5)	0·264 (0·0856 to 0·625)	0·436 (0·140 to 1·01)	64·8% (–41·7 to 438·0)	0·405 (0·154 to 0·898)	0·784 (0·274 to 1·70)	93·5% (–29·7 to 495·7)
North Africa and Middle East	2·09 (0·876 to 4·16)	2·42 (1·27 to 4·44)	16·2% (–38·2 to 138·9)	0·629 (0·233 to 1·29)	0·687 (0·354 to 1·33)	9·2% (–48·2 to 134·7)	1·46 (0·632 to 3·00)	1·74 (0·882 to 3·20)	19·2% (–38·5 to 154·5)
South Asia	79·8 (43·3 to 127)	210 (128 to 318)	163·3% (51·8 to 357·0)	31·3 (16·6 to 55·1)	83·6 (45·6 to 137)	166·9% (37·4 to 376·1)	48·5 (26·0 to 76·2)	127 (82·0 to 188)	161·0% (51·1 to 358·0)
Southeast Asia, east Asia, and Oceania	16·3 (6·18 to 34·6)	25·9 (10·3 to 52·7)	58·6% (–34·0 to 331·6)	8·15 (3·04 to 16·5)	11·3 (4·8 to 22·4)	38·1% (–43·4 to 259·9)	8·20 (3·16 to 18·5)	14·7 (5·71 to 30·8)	79·0% (–31·8 to 386·9)
Sub-Saharan Africa	2·33 (0·898 to 4·93)	4·95 (1·94 to 10·4)	112·9% (–16·7 to 450·3)	1·12 (0·394 to 2·31)	2·43 (0·873 to 5·50)	117·3% (–19·0 to 512·9)	1·21 (0·510 to 2·54)	2·53 (1·08 to 5·45)	108·9% (–17·6 to 450·0)

Data in parentheses are 95% uncertainty intervals. Count data are given to three significant figures. The number of attributable deaths for more granular locations is in appendix 2.

**Table 3:** The ten countries with the highest numbers of chewing tobacco users and attributable DALYs and deaths among males and females globally, 2023

	Chewing tobacco users			Deaths attributable to chewing tobacco			DALYs attributable to chewing tobacco		
	Top 10 country rank	Number of users (millions)	Proportion of global users	Top 10 country rank	Number of deaths (thousands)	Proportion of global attributable deaths	Top 10 country rank	DALYs (thousands)	Proportion of global attributable DALYs
**Males**									
Bangladesh	2	12·2 (9·96–15·1)	7·6% (6·0–9·6)	2	14·4 (10·2–19·7)	9·7% (6·9–13·0)	2	405 (296–537)	10·0% (6·8–13·7)
China	4	6·58 (2·62–13·6)	3·9% (1·8–6·6)	3	6·82 (2·36–14·7)	4·4% (2·3–7·2)	4	175 (63·2–382)	4·1% (2·1–6·7)
India	1	105 (79·4–139)	64·3% (56·1–71·8)	1	104 (65·4–158)	68·6% (62·2–74·4)	1	2820 (1810–4210)	67·6% (61·5–73·1)
Madagascar	8	1·12 (0·618–1·81)	0·7% (0·5–0·8)	10	0·730 (0·388–1·34)	0·5% (0·3–0·6)	9	25·4 (14·4–45·1)	0·6% (0·4–0·8)
Myanmar	6	3·81 (1·94–6·25)	2·3% (1·4–3·1)	5	3·73 (1·65–7·20)	2·4% (1·4–3·6)	5	134 (61·8–256)	3·2% (1·8–4·6)
Nepal	7	3·19 (2·32–4·20)	1·9% (1·7–2·1)	7	2·04 (1·32–3·08)	1·4% (1·1–1·7)	7	61·0 (41·0–88·0)	1·5% (1·2–1·8)
Pakistan	3	10·4 (5·84–17·7)	6·3% (4·6–8·0)	4	6·37 (3·26–12·0)	4·2% (2·9–5·7)	3	203 (106–381)	4·8% (3·4–6·3)
Philippines	10	1·01 (0·425–2·00)	0·6% (0·3–1·0)	9	0·799 (0·316–1·71)	0·5% (0·3–0·8)	10	21·7 (8·44–47·4)	0·5% (0·3–0·8)
Sri Lanka	9	1·08 (0·508–1·90)	0·6% (0·4–0·9)	8	1·26 (0·564–2·29)	0·8% (0·5–1·1)	8	33·2 (15·5–59·3)	0·8% (0·5–1·1)
USA	5	5·90 (3·67–9·22)	3·6% (3·0–4·5)	6	3·05 (1·89–4·69)	2·1% (1·4–3·3)	6	73·2 (45·2–114)	1·8% (1·3–2·6)
**Females**									
Bangladesh	2	14·4 (9·53–20·6)	19·0% (14·5–24·6)	2	18·5 (10·8–27·6)	19·0% (13·7–25·3)	2	446 (270–643)	19·9% (14·4–25·9)
Cambodia	··	··	··	8	0·988 (0·482–1·80)	1·0% (0·5–2·0)	8	20·1 (10·0–36·4)	0·9% (0·4–1·8)
China	3	3·18 (1·19–6·80)	3·9% (2·2–6·0)	6	1·99 (0·624–4·54)	1·9% (1·0–3·2)	6	41·3 (12·6–96·2)	1·7% (0·9–2·9)
India	1	42·9 (23·4–72·6)	54·8% (47·1–62·4)	1	59·4 (30·1–101)	59·5% (51·0–67·5)	1	1360 (693–2310)	59·0% (51·0–67·0)
Indonesia	6	1·12 (0·43–2·33)	1·4% (0·8–2·1)	4	2·53 (0·844–5·60)	2·5% (1·2–3·9)	5	52·1 (18·0–121)	2·2% (1·1–3·4)
Madagascar	8	0·730 (0·397–1·22)	0·9% (0·9–1·0)	··	··	··	··	··	··
Myanmar	5	1·69 (0·722–3·21)	2·1% (1·4–2·9)	5	2·11 (0·729–4·35)	2·1% (1·2–3·2)	4	56·6 (20·0–115)	2·4% (1·4–3·6)
Nepal	10	0·582 (0·311–0·996)	0·7% (0·7–0·8)	10	0·766 (0·339–1·46)	0·8% (0·5–1·1)	10	15·4 (7·10–28·3)	0·7% (0·5–0·9)
Pakistan	4	2·77 (1·17–5·55)	3·5% (2·2–4·8)	3	4·89 (2·14–9·11)	4·9% (3·0–7·1)	3	123 (52·9–224)	5·3% (3·3–7·6)
Thailand	7	1·01 (0·412–2·02)	1·3% (0·8–1·8)	7	1·21 (0·459–2·41)	1·2% (0·6–1·9)	7	24·1 (8·95–49·7)	1·0% (0·6–1·6)
USA	9	0·696 (0·262–1·48)	0·9% (0·5–1·3)	··	··	··	··	··	··
Viet Nam	··	··	··	9	0·966 (0·329–2·15)	0·9% (0·5–1·6)	9	17·4 (5·85–38·2)	0·7% (0·4–1·3)

Data in parentheses are 95% uncertainty intervals. Count data are given to three significant figures. DALY=disability-adjusted life-year.

When accounting for premature mortality, an estimated 6·17 million (95% UI 3·76–9·37) years of life lost were attributed to chewing tobacco use globally in 2023, which was a 115·6% (95% UI 21·6–276·3) increase from 1990 (appendix 2 pp 83–97). Moreover, the rate of attributable years of life lost among males (127·3 [80·7–192·1] per 100 000) was double that among females (63·6 [33·8–105·1] per 100 000; appendix 2 pp 98–111). A further 307 thousand (164–568) years lived with disability were attributable to chewing tobacco in 2023, a 128·1% (29·3–305·9) increase from 1990 (appendix 2 pp 112–141). Years lived with disability were split more evenly between sexes with 194 thousand (108–346) years lived with disability among males and 113 thousand (56·1–223) among females (appendix 2 pp 112–126).

Combining years of life lost and years lived with disability, the total number of DALYs associated with chewing tobacco use was estimated at 6·48 million (3·96–9·91) in 2023 (table 4; appendix 2 pp 142–156). Globally, chewing tobacco contributed to 133·5 (85·8–201·8) DALYs per 100 000 males and 66·9 (35·6–109·9) DALYs per 100 000 females (figure 2; appendix 2 pp 157–172). The region with the highest age-standardised DALY rates was south Asia with 611·1 (402·3–903·0) per 100 000 males and 349·4 (188·7–564·8) per 100 000 females (appendix 2 pp 157–171). Unlike other tobacco risk factors, where attributable DALYs disproportionately affect males in most world regions,^28^ the rates of DALYs attributable to chewing tobacco were higher among females than among males in 32 countries (figure 3). The highest gaps in DALY rates between females and males were in Cambodia (377·0 [186·9–688·4] DALYs per 100 000 females compared with 53·4 [20·3–129·5] per 100 000 males) and Bangladesh (1082·2 [656·7–1579·9] DALYs per 100 000 females compared with 768·7 [555·5–1012·8] per 100 000 males; figure 3; appendix 2 pp 157–171). Moreover, Bangladesh had the highest rates of chewing tobacco DALYs in 2023 and made up 19·9% (14·4–25·9) and 10·0% (6·8–13·7) of the female and male global DALYs attributable to chewing tobacco, respectively (Figure 3, Figure 4; table 3). Akin to chewing tobacco-related deaths, China, India, Madagascar, Myanmar, Nepal, Pakistan, Philippines, Sri Lanka, and the USA joined Bangladesh to make up over 90% of the attributable DALYs globally for males (table 3). For females, Bangladesh was joined by Cambodia, China, India, Indonesia, Myanmar, Nepal, Pakistan, Thailand, and Viet Nam as the countries with the highest number of DALYs, totalling over 90% of the attributable DALYs for females (table 3). Although global age-standardised DALY rates have not changed notably since 1990, the number of attributable DALYs increased by 116·2% (22·1–277·7; appendix 2 pp 142–156).

**Table 4:** Number of DALYs attributable to chewing tobacco among individuals aged 15 years and older by super-region and sex, 1990 and 2023

	Overall DALYs attributable to chewing tobacco (thousands)			Chewing tobacco DALYs among females (thousands)			Chewing tobacco DALYs among males (thousands)		
	1990	2023	Percentage change	1990	2023	Percentage change	1990	2023	Percentage change
Global	3000 (1550 to 5000)	6480 (3960 to 9910)	116·2% (22·1 to 277·7)	1080 (550 to 1990)	2300 (1220 to 3790)	113·2% (10·6 to 281·9)	1920 (989 to 3180)	4180 (2670 to 6320)	117·9% (31·4 to 278·6)
Central Europe, eastern Europe, and central Asia	31·9 (11·7 to 72·3)	31·8 (10·5 to 70·8)	–0·4% (–64·4 to 201·4)	10·6 (3·36 to 24·9)	8·19 (2·54 to 19·4)	–22·5% (–73·1 to 151·5)	21·3 (8·33 to 46·2)	23·6 (8·26 to 51·2)	10·6% (–60·1 to 223·9)
High income	106 (62·3 to 176)	116 (72·4 to 199)	9·0% (–40·4 to 110·1)	13·5 (4·46 to 31·5)	15·2 (4·83 to 35·4)	13·1% (–60·1 to 263·8)	92·9 (55·6 to 148)	101 (62·6 to 171)	8·4% (–38·1 to 96·2)
Latin America and Caribbean	16·3 (5·93 to 37·3)	27·4 (8·99 to 60·7)	68·4% (–40·2 to 431·1)	5·96 (1·96 to 14·0)	8·81 (2·80 to 20·5)	47·9% (–48·2 to 383·2)	10·3 (3·91 to 22·9)	18·6 (6·57 to 40·0)	80·2% (–35·4 to 456·9)
North Africa and Middle East	51·8 (21·6 to 106)	59·8 (30·5 to 112)	15·5% (–39·3 to 146·8)	14·1 (5·21 to 28·8)	14·9 (7·57 to 30·1)	6·4% (–50·1 to 136·0)	37·7 (16·4 to 79·4)	44·9 (23·0 to 85·5)	19·0% (–39·2 to 161·3)
South Asia	2280 (1260 to 3610)	5430 (3420 to 8090)	138·0% (39·2 to 305·5)	819 (433 to 1460)	1940 (1060 to 3130)	137·1% (24·3 to 313·1)	1460 (787 to 2280)	3490 (2310 to 5090)	138·4% (42·5 to 309·4)
Southeast Asia, east Asia, and Oceania	438 (169 to 945)	667 (265 to 1360)	52·3% (–37·2 to 304·6)	185 (68·5 to 385)	246 (107 to 505)	32·5% (–46·1 to 244·0)	253 (101 to 573)	422 (168 to 866)	66·8% (–34·7 to 354·0)
Sub-Saharan Africa	68·3 (27·6 to 145)	143 (59·6 to 296)	108·8% (–18·3 to 416·8)	29·3 (10·8 to 61·0)	63·2 (23·8 to 138)	115·6% (–15·4 to 495·1)	39·0 (17·1 to 82·3)	79·5 (35·4 to 170)	103·7% (–19·6 to 415·0)

Data in parentheses are 95% uncertainty intervals. Count data are given to three significant figures. The number of attributable DALYs for more granular locations are in appendix 2. DALYs=disability-adjusted life-years.

**Figure 2:**
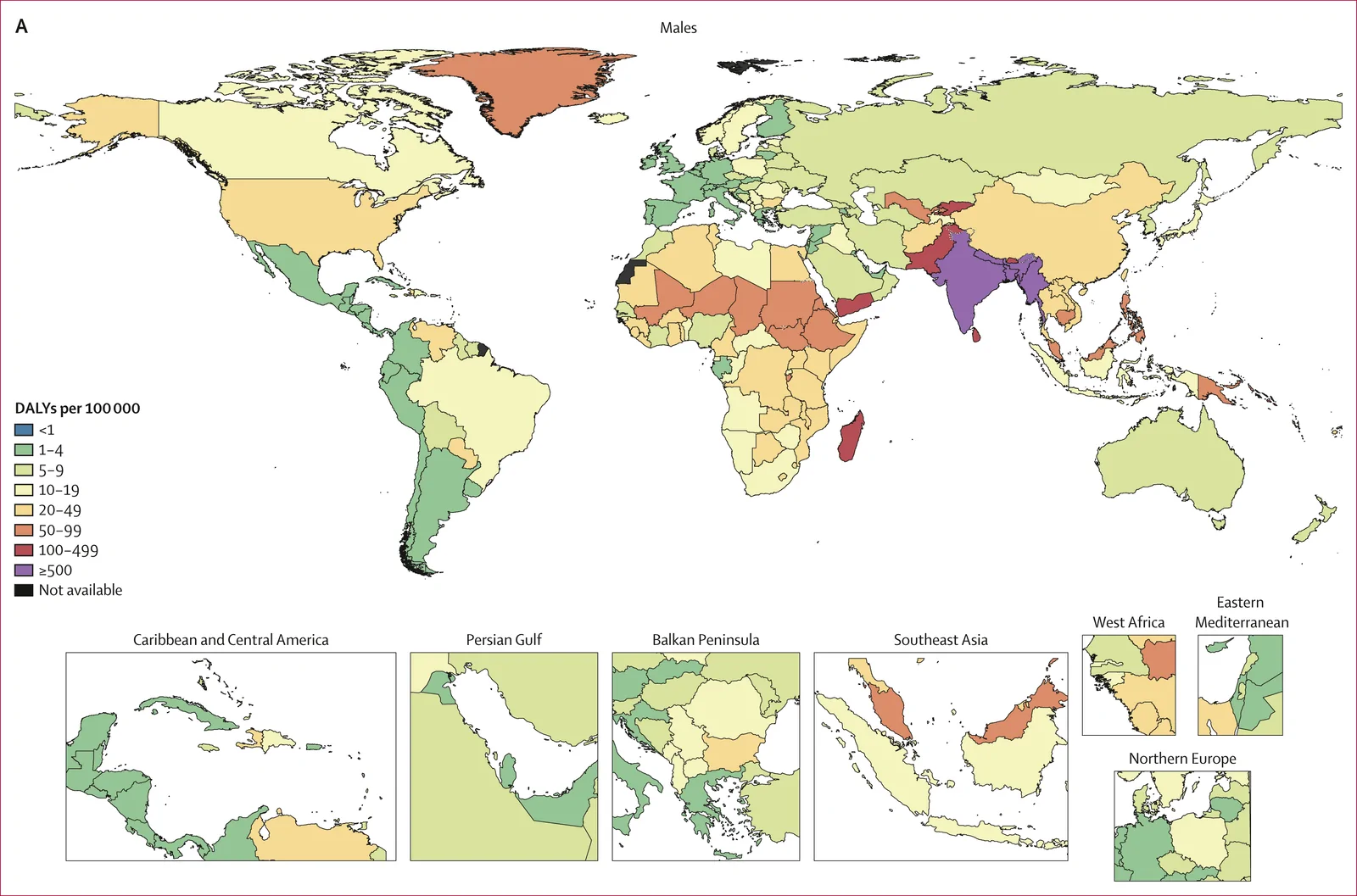

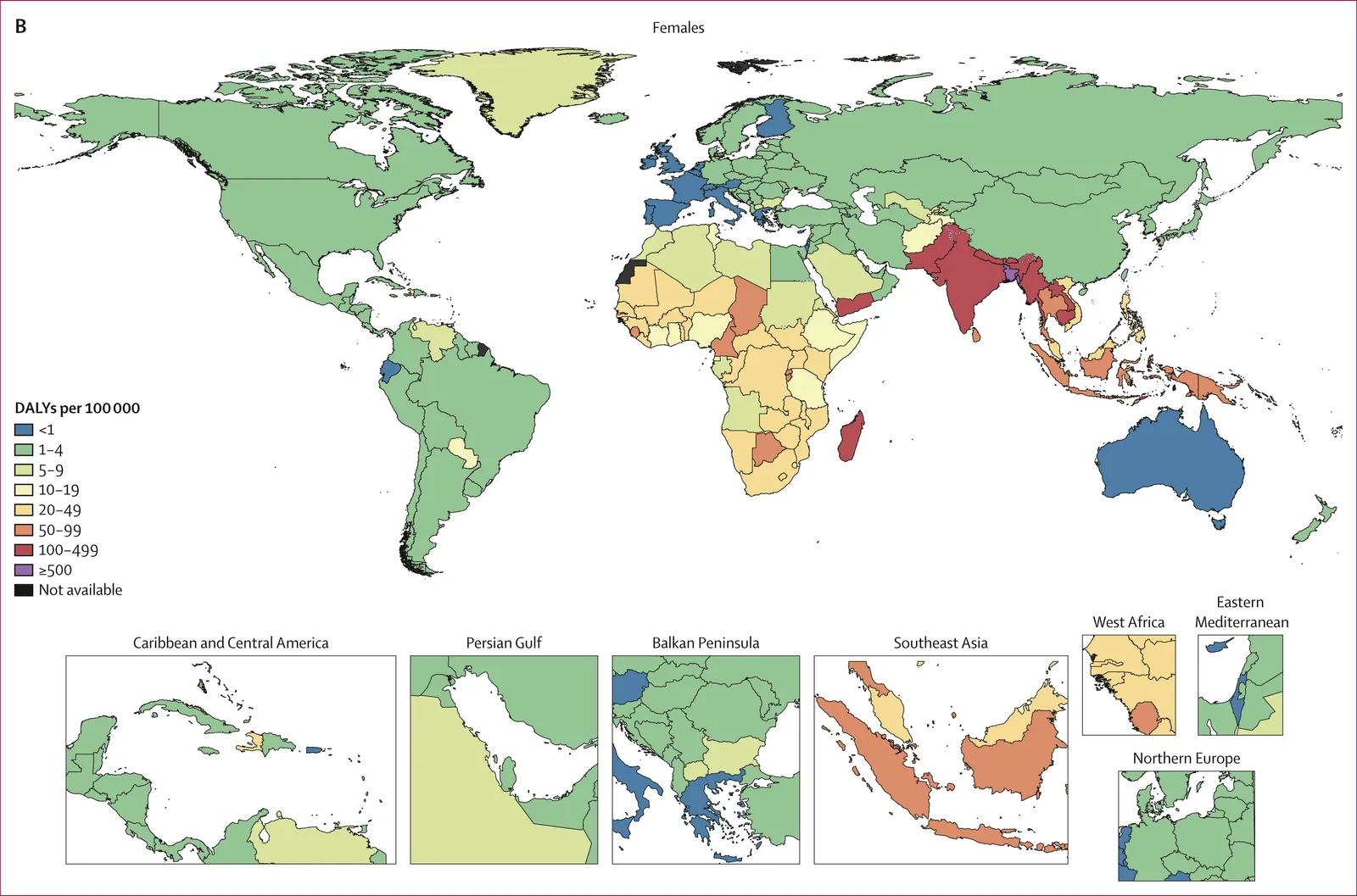
Age-standardised rates of DALYs attributable to chewing tobacco use in males (A) and females (B), among individuals aged 15 years and older, 2023 The countries on this map are coloured in accordance with the age-standardised rate of DALYs attributable to chewing tobacco use per 100 000 individuals aged 15 years and older by sex. Purple indicates high DALY rates and blue indicates lower rates. Chewing tobacco use is specifically defined as current use of masticated tobacco products, and the DALYs depicted here manifest through the combined burden of six health outcomes associated with this risk factor: stroke, lip and oral cavity cancer, oesophageal cancer, laryngeal cancer, nasopharyngeal cancer, and other pharyngeal cancer. The country-specific age-standardised DALY rates for people aged 15 years and older are in appendix 2 (pp 157–171). DALYs=disability-adjusted life-years.

**Figure 3:**
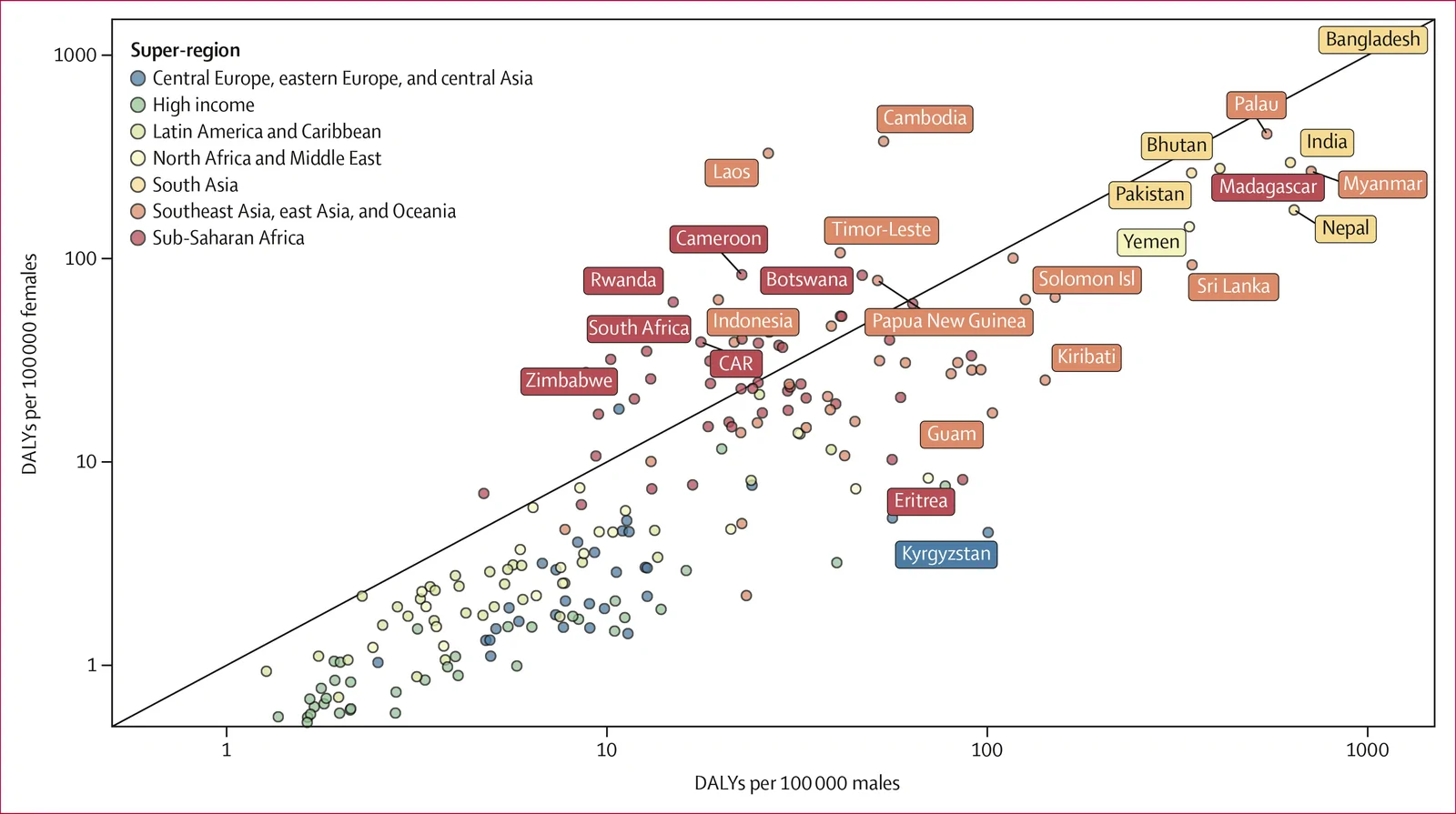
Comparison of age-standardised rates of DALYs attributable to chewing tobacco use between females and males by location, 2023 This figure compares the age-standardised rates for people aged 15 years and older of DALYs among males (x-axis) and females (y-axis) with each dot representing one of the 204 countries and territories estimated as part of The Global Burden of Diseases, Injuries, and Risk Factors Study 2023. The dots are filled to indicate their corresponding super-region. The diagonal line reflects the line of equivalence between female and male DALY rates, so the countries above the line have higher rates among females than among males, and below the line have higher rates among males than females. The axes are scaled to facilitate the visualisation of all countries despite heterogenous chewing tobacco burden, and country labels have been added for locations where the rate of DALYs among females is more than 20 per 100 000 greater than the rate among males or the rate among males is more than 75 per 100 000 greater than that among females to balance relevance and readability. The country-specific and sex-specific age-standardised DALY rates for people aged 15 years and older are in appendix 2 (pp 157–171). CAR=Central African Republic. DALYs=disability-adjusted life-years. Isl=islands.

**Figure 4:**
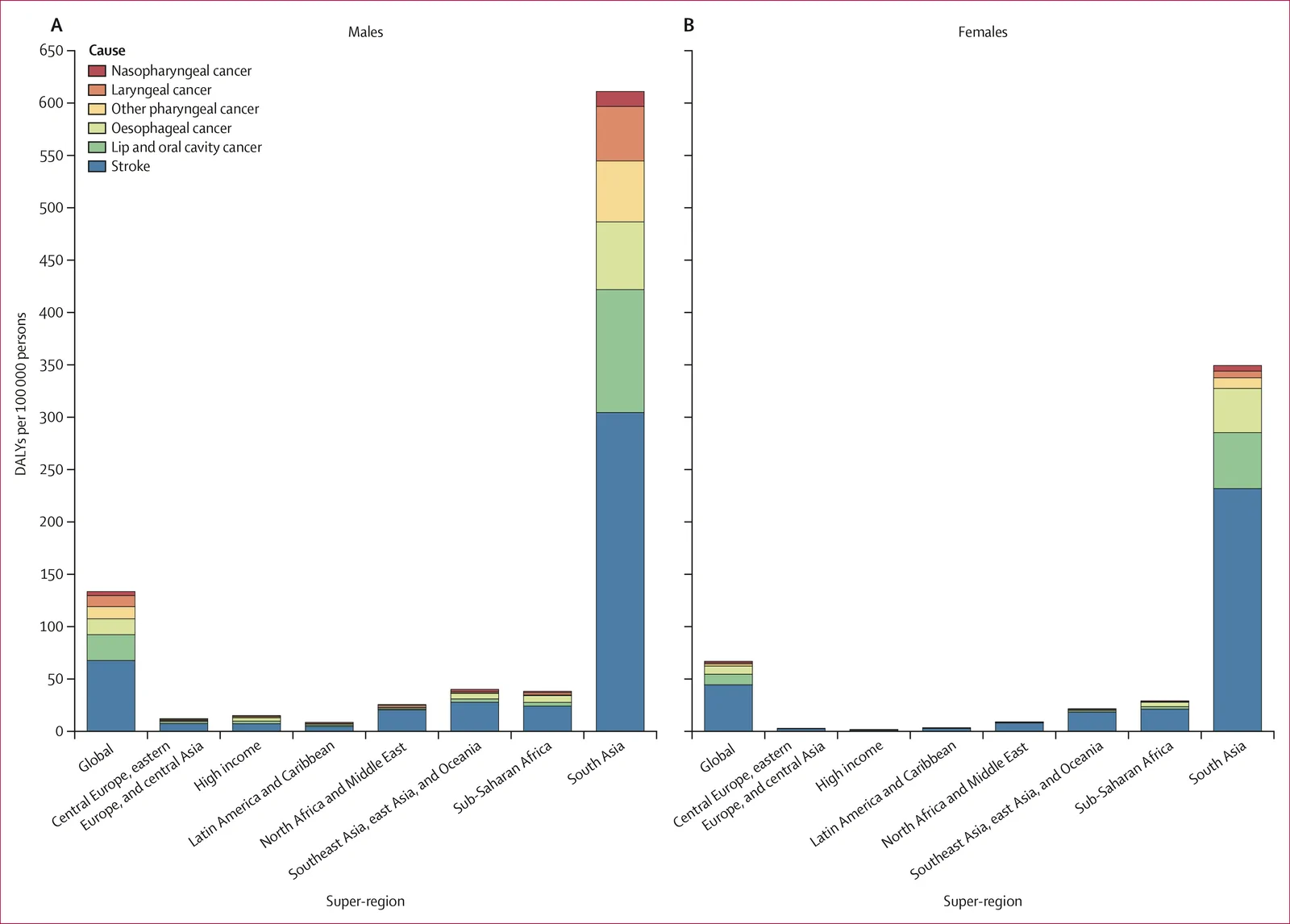
Cause-specific age-standardised rates of DALYs attributable to chewing tobacco use by super-region and globally for males (A) and females (B), 2023 Each bar presents the cause-specific age-standardised DALY rates attributable to chewing tobacco among individuals aged 15 years and older stacked within a specific geographic region. DALYs=disability-adjusted life-years.

The burden attributable to chewing tobacco manifested through six associated health outcomes: lip and oral cavity cancer, laryngeal cancer, nasopharyngeal cancer, other pharyngeal cancer, oesophageal cancer, and stroke (appendix 2 pp 173–174). As evidenced in the burden of proof analyses, these six associations are two-star and one-star risk–outcome pairs, reflecting moderate and weak underlying evidence, respectively.^9^ Globally, stroke contributed the largest number of chewing tobacco DALYs equivalent to 55·9 (95% UI 29·0–92·9) stroke-related DALYs per 100 000 individuals (figure 4; appendix 2 pp 173–174). Although chewing tobacco accounted for a relatively small fraction (2·4% [1·2–3·8]) of global stroke DALYs, its contribution to stroke DALYs in south Asia was nearly four times higher (9·2% [4·9–14·8]; appendix 2 pp 175–178). Lip and oral cavity cancer DALYs attributable to chewing tobacco were the second highest with an estimate of 17·4 (11·3–24·3) DALYs per 100 000. Chewing tobacco accounted for 17·3% (12·2–23·3) of all lip and oral cavity cancer-related DALYs (appendix 2 pp 173–178). The remaining four outcomes made up less than a quarter of all chewing tobacco burden, including 11·5 (7·8–20·2) for oesophageal cancer, 6·7 (3·8–11·6) for other pharyngeal cancer, 6·0 (3·4–10·5) for laryngeal cancer, and 2·6 (1·3–4·2) for nasopharyngeal cancer DALYs per 100 000 individuals (appendix 2 pp 173–174; figure 4). Conversely, these chewing tobacco-attributable DALYs accounted for 5% to 14% of the cause-specific DALYs, marking a substantial contribution to the global burden of each condition (appendix 2 pp 175–178). In south Asia, the share of chewing tobacco-attributable DALYs for head and neck cancers was substantially higher, ranging from 30·5% (21·9–41·5) of lip and oral cavity cancer DALYs to 18·4% (11·5–26·7) of oesophageal cancer DALYs (appendix 2 pp 175–179). The gap in chewing tobacco-attributable DALYs between females and males in south Asia is reflective of the markedly higher rate of lip and oral cavity cancer DALYs attributable to chewing tobacco among males (117·4 [73·8–168·0] per 100 000) than females (53·4 [31·8–82·3] per 100 000). The DALY rates of laryngeal cancer and other pharyngeal cancer in males were also nine and five times higher, respectively, than in females in south Asia (appendix 2 pp 175–178; figure 4).

## Discussion

This study provides an updated overview of trends in the global prevalence and novel findings on disease burden attributable to chewing tobacco use. In 2023, there were an estimated 241 million (95% UI 165–355) chewing tobacco users, with the majority residing in south Asia, particularly in India and Bangladesh. Considerable increases in the number of chewing tobacco users were found in several countries including Bangladesh, Madagascar, and Papua New Guinea. Furthermore, chewing tobacco continues to lead to substantial health loss that is highly geographically concentrated by sex with ten countries making up over 90% of chewing tobacco-related deaths and DALYs.

Unlike the progress achieved under the WHO Framework Convention on Tobacco Control (FCTC) in smoking tobacco control in the past few decades, the same progress is not evident in the context of chewing tobacco.^29^ In line with the FCTC, particularly Articles 9 and 10,^30^ a range of policies have been introduced over the years; however, considerable gaps remain in policy implementation and enforcement.^31^ For example, in India—the country with the largest number of chewing tobacco users—a ban on the popular product gutkha was introduced in 2012 as part of the Food Safety and Standards Act; yet, it continues to be available on the market in alternative forms.^32^ Labelling and packaging legislation introduced in south Asian countries, including Bangladesh, India, and Pakistan, is frequently violated.^33^, ^34^, ^35^ In contrast, taxation policies, one of the most effective measures for smoking tobacco control, have been implemented in several high-prevalence countries, including Bangladesh.^36^^,^^37^ In India, taxation on chewing tobacco is subject to multiple layers of taxes, including goods and services tax, compensation cess, excise duty, and National Calamity Contingent Duty.^38^^,^^39^ Despite these measures, industry interference on legislation, adapted sales strategies to circumvent regulations, and inconsistent enforcement have enabled chewing tobacco products to remain available and affordable.^40^, ^41^, ^42^, ^43^

Unlike previous studies that reported an optimistic trend in chewing tobacco control,^33^^,^^34^^,^^44^ the trends in prevalence and burden reported in this study underscore the urgency for continued and strengthened policy interventions. In south Asia, the epicentre of the chewing tobacco epidemic, cultural normalisation driven by deep-rooted tradition has contributed to its widespread acceptability and users might not be responsive to health warning displays or advertisement prohibitions.^15^^,^^45^^,^^46^ The higher prevalence of chewing tobacco use among rural and low-income populations,^47^^,^^48^ further emphasises the need for tailored tobacco control policies and research into the specific subgroups of populations with high smokeless tobacco use.^5^^,^^33^^,^^34^^,^^44^^,^^45^^,^^49^ For example, illegal sales of gutkha in rural India are still happening, and alternative packaging such as twin packets containing chewing tobacco and spice mixtures are also easily accessible and largely unregulated.^50^

The importance of contextualised policy is further emphasised by the difference in chewing tobacco patterns by sex and age. The 32 countries with higher rates of chewing tobacco DALY rates among females compared with males point to different gender-related factors coming into play with chewing tobacco. For example, in many south Asian contexts, chewing tobacco has been reported to be more socially acceptable for women than smoking, which might contribute to the different use patterns.^51^ Our results indicate a need to target initiation prevention at an early age, and at the same time, with increasing prevalence into late adulthood, cessation strategies must be crafted to reach individuals across a broad age spectrum. Considerations of these age patterns and future research into the drivers of use at older ages are essential when tailoring chewing tobacco control programmes. Creative and context-specific solutions are needed to alter the course of chewing tobacco burden.

In GBD 2023, we incorporated for the first time additional health outcomes associated with chewing tobacco use (appendix 2 p 179). The results show that stroke accounted for the largest number of DALYs attributable to chewing tobacco, followed by lip and oral cavity cancer, and oesophageal cancer. Health loss is particularly pronounced in south Asia, with over 8% of stroke-related DALYs in the region attributable to chewing tobacco use. Importantly, although there were only three studies reporting on this association, they displayed resounding consistency on the association between chewing tobacco and stroke, attributed in part to chewing tobacco’s role increasing blood pressure,^52^ impairing inner linings of blood vessels,^53^ and exacerbating metabolic dysfunction.^9^^,^^54^ Similarly, as of 2023, India has the world’s largest population affected by lip and oral cavity cancer, with Pakistan and Bangladesh ranking fourth and fifth, respectively, globally.^55^ Approximately 32% of the lip and oral cancer DALYs in these countries are attributable to chewing tobacco use. Prolonged exposure to carcinogens contained in chewing tobacco causes fibroblast injury and DNA and RNA damage, ultimately leading to lip and oral cavity cancer.^56^ For countries in south Asia, mitigating the burden of cardiovascular diseases and cancers and achieving Sustainable Development Goal 3.4 will require deliberate integrations of chewing tobacco control into national non-communicable disease prevention strategies.^57^^,^^58^

This study has several limitations. First, data on chewing tobacco use were self-reported and might be subject to reporting bias that could vary across countries, cultural norms, and over time. Second, survey questions on chewing tobacco use differ across instruments, none of which capture all the available chewing tobacco product types. Products have divergent chemical compositions and additives, including non-tobacco ingredients such as areca nut or khat, which might affect health loss associated with their use in a way that this study is not able to capture. Survey questions also can differ in terms of the period they define as current use. Although most of our included studies use our preferred 30-day period, we also included alternative survey-defined current use, including those without explicit recall time constraints.

Third, in estimating health loss associated with chewing tobacco, we considered six outcomes with sufficient scientific evidence, and which meet the GBD study’s inclusion criteria. The six associations with chewing tobacco are two-star and one-star risk–outcome pairs, reflecting moderate and weak underlying evidence, respectively, and would benefit from future research into these and other health consequences.^9^ Furthermore, the evidence base on the health effects associated with chewing tobacco is evolving, with much of the existing literature not disentangling the health consequences of chewing tobacco from those of other forms of smokeless tobacco. At present, the association between chewing tobacco and ischaemic heart disease received a zero-star rating in the burden of proof analysis, indicating insufficient evidence of a statistically significant relationship, and cannot be included in GBD estimates. We will continue to update the systematic review in future GBD rounds and reassess the association as new evidence becomes available. Our approach also captures geographical variation in health loss through location-specific prevalence but is unable to capture differences in the chewing intensity and risks associated with different types of chewing tobacco products. As more research accumulates, this analysis will be updated to incorporate the latest evidence.

Fourth, this study did not examine the dual use of chewing tobacco and other tobacco products, which has direct implications for health loss. Although we primarily used data on health risks for chewing tobacco that controlled for the use of smoked tobacco products and the GBD framework accounts for joint risk factor estimation,^1^ this study does not otherwise account for or capture the potential compounding effects of dual use on health. Dual use of tobacco products might have a large impact in high-burden regions such as south Asia where smoking prevalence is also high. Furthermore, the present analysis captures the burden of current chewing tobacco use, but due to evidence sparsity, we are not able to capture the health risks that might persist among former users.

Chewing tobacco use contributes to considerable disease burden especially in south Asia, affecting millions in the region. Prevalence trends have remained largely stagnant globally in the past three decades with increases observed in several nations. Furthermore, chewing tobacco persistently makes up over 3% of the total global tobacco disease burden (appendix 2 p 181). Although embedded within the scope of the WHO FCTC, chewing tobacco has yet to be approached with the same degree of policy attention dedicated to smoking tobacco. Given that health loss associated with chewing tobacco is concentrated in ten countries, there is an urgent need for context-specific, gender-specific, and age-specific evidence-based strategies to address this public health issue, especially in these countries.

## Data sharing

This study follows the GATHER statement. To download the data used in these analyses, please visit the GBD 2023 Sources Tool at https://ghdx.healthdata.org/gbd-2023/sources.

## Declaration of interests

M S Aslam reports grants or contracts from Xiamen University Malaysia Research Fund (XMUMRF/2025-C15/ITCM/0006, XMUMRF/2023-C11/ISEM/0041, and XMUMRF/2026-C17/ITCM/0007) outside the submitted work. S Bhaskar reports grants or contracts from Japan Society for the Promotion of Science (JSPS), Japanese Ministry of Education, Culture, Sports, Science and Technology Grant-in-Aid for Scientific Research (grant 23KF0126) and from JSPS and the Australian Academy of Science JSPS International Fellowship (grant P23712); payment or honoraria for lectures, presentations, speakers bureaus, manuscript writing or educational events from Japan Stroke Society, Japan (Honoraria for Invited Keynote Presentation at the 50th Annual Meeting of the Japan Stroke Society, Osaka) and from Semey Medical University, Kazakhstan (Honorarium for Invited Professorship and Teaching Course in Neurocardiovascular Therapy); support for attending meetings or travel from Japan Stroke Society, Japan (Travel Award for Invited Keynote Presentation at the 50th Annual Meeting of the Japan Stroke Society, Osaka); leadership or fiduciary role in other board, society, committee or advocacy group, paid or unpaid as District Chair, Diversity, Equity, Inclusion & Belonging, Rotary District 9675 (Sydney, NSW, Australia); chair, Founding Member, and Manager of Global Health and Migration Hub Community, Global Health Hub Germany (Berlin, Germany); Editorial Board Member for *PLOS One*, *BMC Neurology*, *Frontiers in Neurology*, *Frontiers in Stroke*, *Frontiers in Public Health*, *Journal of Aging Research*, *Neurology International*, Diagnostics, and *BMC Medical Research Methodology*; member of the College of Reviewers, Canadian Institutes of Health Research, Government of Canada; director of research for the World Headache Society (Bengaluru, India); Expert adviser or reviewer, Cariplo Foundation (Milan, Italy); visiting director for the National Cerebral and Cardiovascular Center, Department of Neurology, Division of Cerebrovascular Medicine and Neurology, Suita (Osaka, Japan); member of the Scientific Review Committee, Cardiff University Biobank (Cardiff, UK); Rotary Reconciliation Action Plan Chair; and Healthcare and Medical Adviser, Japan Connect (Osaka, Japan), outside the submitted work. M Carvalho reports other financial or non-financial interests from Laboratório Associado para a Química Verde (Associated Laboratory for Green Chemistry) Rede de Química e Tecnologia (Network of Chemistry and Technology), University of Porto, Porto, Portugal, and from Fundação para a Ciência e a Tecnologia/Ministério da Ciência, Tecnologia e Ensino Superior, project UID/50006/2025 (DOI 10.54499/UID/50006/2025) outside the submitted work. R Dandona reports grants or contracts from Mariwala Health Initiative, Mumbai, India, and from Indian Council of Medical Research, India; participation on a Data Safety Monitoring Board or Advisory Board with the Independent Programme Oversight Committee for Community-Pharmacies Managing Hypertension: Intervention Development and Evaluation in Bangladesh and Pakistan (NIHR 206976; chair), the Funding Committee, Global National Institute for Health and Care Research (NIHR) Global Health Research Groups Call 5, NIHR, UK (member), and the Health Metrics Sciences MS Admissions committee, University of Washington (WA, USA), outside the submitted work. A M Hopkins reports grants or contracts from National Health and Medical Research Council, Australia, Tour de Cure, Australia, Flinders Foundation, and The Hospital Research Foundation (all research grant funding), and from Boehringer Ingelheim (investigator-initiated funding, outside the scope of the current study); and payment or honoraria for lectures, presentations, speakers bureaus, manuscript writing, or educational events from AstraZeneca (speaker fees), outside the submitted work. I M Ilic reports support for the present manuscript from the Ministry of Science, Technological Development and Innovation of the Republic of Serbia (451-03-137/2025-03/200110). M D Ilic reports support for the present manuscript from the Ministry of Science, Technological Development and Innovation of the Republic of Serbia (451-03-47/2023-01/200111). M K Kashyap reports grants or contracts from Indian Council of Medical Research, New Delhi (5/13/55/2020/NCD-III); payment or honoraria for lectures, presentations, speakers bureaus, manuscript writing or educational events from Institute of Liver and Biliary Sciences, (New Delhi, India) and from Kuvempu University (Shimoga, Karnataka, India); patents planned, issued or pending: 2023-1100-3940 (Indian patent-pending) and 2023-1105-8515 (Indian patent-pending) outside the submitted work. K Krishan reports other financial or non-financial interests from the University Grants Commission Centre of Advanced Study, Centre of Advanced Study II (awarded to the Department of Anthropology, Panjab University, Chandigarh, India) and from Rashtriya Uchchatar Shiksha Abhiyan (RUSA) 2.0 grant, Ministry of Education, Panjab University, Chandigarh, India outside the submitted work. G Liu reports grants or contracts from the Al & Val Rosenstrauss Fellowship, Rebecca L Cooper Foundation (fellowship to support G Liu’s salary during the work) outside the submitted work. J Liu reports support for the present manuscript and grants or contracts from Beijing Natural Science Foundation (Z24004) and from the National Natural Science Foundation (72474005). R S Moreira reports grants or contracts from Conselho Nacional de Desenvolvimento Científico e Tecnológico (National Council for Scientific and Technological Development), National Council for Scientific and Technological Development (research productivity scholarship 308986/2025-3, which is related to research using the GBD) outside the submitted work. B Oancea reports support for the present manuscript from the Romanian National Research, Development, and Innovation Plan 2022–2027 (project PNRR/2022/C9/MCID/I8 number 1672173292, contract number 760231, and project PNRR/2022/C9/MCID/I8 number 842027778, contract number 760096). R F Palma-Alvarez reports payment or honoraria for lectures, presentations, speakers bureaus, manuscript writing or educational events from Angelini, Casen Recordati, Lundbeck, Rovi, Rubió, and Takeda; support for attending meetings or travel from Angelini, Italfarmaco, Advanz Pharma, and Rovi outside the submitted work. A K Panda reports support for the present manuscript from the Environmental Science Laboratory, School of Applied Sciences, Kalinga Institute of Industrial Technology (KIIT) Deemed to Be University, Bhubaneswar, Odisha, India and from the Centre for Water Research and Climate Change, KIIT Deemed to Be University, Bhubaneswar, Odisha, India and from Achyuta Samanta (founder of Kalinga Institute of Industrial Technology, Deemed to be University, Kalinga Institute of Social Sciences, Deemed to be University and Art of Giving). R Passera reports participation on a Data Safety Monitoring Board or Advisory Board as Member of the Data Safety Monitoring Board for the Consolidation with ADCT-402 (loncastuximab tesirine) after immunochemotherapy: a phase II study in BTKi-treated/ineligible Relapse/Refractory Mantle Cell Lymphoma patients clinical trial, Fondazione Italiana Linfomi (FIL), Alessandria, Italy; leadership or fiduciary role in other board, society, committee or advocacy group, paid or unpaid as Member of the European Society for Blood and Marrow Transplantation Statistical Committee, European Society for Blood and Marrow Transplantation, Paris, France; Member of the FIL Statistical Committee, FIL, Alessandria, Italy; and past member 2020–2023 (biostatistician) of the Institutional Review Board/Independent Ethics Committee Comitato Etico Azienda Ospedaliera. Antonio e Biagio Alessandria-Azienda Sanitaria Locale Alessandria-Vercelli, Italy (no payment received for all) outside the submitted work. A Shaban-Nejad reports grants or contracts from National Cancer Institute, Tennessee Department of Health (research grant unrelated to this manuscript), and National Institute on Minority Health and Health Disparities (research grant unrelated to this manuscript); consulting fees from Haleon (honoraria for panel participation; unrelated to this manuscript); support for attending meetings or travel from Haleon and from National Comprehensive Cancer Network; outside the submitted work. V Sharma reports other financial or non-financial interests from Directorate of Forensic Science Services (Ministry of Home Affairs [India]), research project DFSS28(1)2019/EMR/6, Department of Forensic Science (formerly Institute of Forensic Science and Criminology), Panjab University, Chandigarh, India; RUSA 2.0 grant to Panjab University; and Partnerships for Accelerated Innovation and Research grant (ANRF/PAIR/2025/000006/PAIR) to Panjab University from ANRF outside the submitted work. J A Singh reports consulting fees from ROMTech, Atheneum, Clearview Healthcare Partners, Yale, Hulio, Horizon Pharmaceuticals/DINORA, ANI/Exeltis USA, SOBI Pharmaceuticals, Frictionless Solutions, Schipher, Crealta/Horizon, Medisys, Fidia, PK Med, Two Labs, Adept Field Solutions, Clinical Care Options, Putnam Associates, Focus Forward, Navigant Consulting, Spherix, MedIQ, Jupiter Life Science, UBM, Trio Health, Medscape, WebMD, and Practice Point Communications; the National Institutes of Health (NIH); and the American College of Rheumatology (consultant fees paid to J A Singh for each entity); payment or honoraria for lectures, presentations, speakers bureaus, manuscript writing or educational events as J A Singh is on the speaker’s bureau of Simply Speaking (J A Singh previously served as a consultant and consultant fees were paid to J A Singh); support for attending meetings or travel as past steering committee member of Outcome Measures in Rheumatology (OMERACT; J A Singh previously received support from the organisation to attend their meeting every 2 years); participation on a Data Safety Monitoring Board or Advisory Board with the US Food and Drug Administration Arthritis Advisory Committee (J A Singh served previously as a member; no financial support); leadership or fiduciary role in other board, society, committee or advocacy group, paid or unpaid as past steering committee member of the OMERACT, an international organization that develops measures for clinical trials and receives arm’s length funding from 12 pharmaceutical companies (J A Singh previously received support from the organisation to attend their meeting every 2 years); stock or stock options in Atyr Pharmaceuticals, Atai Life Sciences, Kintara Therapeutics, Intelligent Biosolutions, Acumen Pharmaceutical, TPT Global Tech, Vaxart Pharmaceuticals, Atyu Biopharma, Adaptimmune Therapeutics, GeoVax Labs, Pieris Pharmaceuticals, Enzolytics, Seres Therapeutics, Tonix Pharmaceuticals Holding, Aebona Pharmaceuticals, and Charlotte’s Web Holdings (J A Singh owns stock options); and previously in Amarin, Viking, and Moderna Pharmaceuticals (J A Singh previously owned stock options) outside the submitted work. F Williams reports support for the present manuscript from the National Institute on Minority Health and Health Disparities, NIH (Division of Intramural Research); the findings and conclusions presented in this paper are those of the author(s) and do not necessarily reflect the views of the NIH or the US Department of Health and Human Services. E Zainal Abidin reports grants or contracts from the Medical Research Council Public Health Intervention Development Scheme, Research Grant (MR/W027801/1) — Communities Facilitating Increasing Smoke-Free Homes (research grant provided to Universiti Putra Malaysia for research on encouraging smoke-free homes in Malaysia focusing on tobacco control), outside the submitted work. All other collaborators declare no competing interests.
