# Supplementary material for: Global, regional, and national burden of exposure to chewing tobacco, 1990–2023: a systematic analysis of the Global Burden of Disease Study 2023
Source: Lancet Glob Health. 2026 Sep 30;14(11):None. doi: 10.1016/j.langlo.2026.104043 (PMC13755197; doi:10.1016/j.langlo.2026.104043)
Supplement: Supplementary file 1 [file mmc1.pdf]

THE LANCET
Global Health

## Supplementary appendix 1

This appendix formed part of the original submission and has been peer reviewed. We post it as supplied by the authors.

Supplement to: Global, regional, and national burden of exposure to chewing tobacco, 1990–2023: a systematic analysis of the Global Burden of Disease Study 2023. *Lancet Glob Health* 2026. https://doi.org/10.1016/j.langlo.2026.104043

# Supplementary Methods and Author Information

## Global, regional, and national burden of exposure to chewing tobacco 1990-2023: a systematic analysis of the Global Burden of Disease 2023

GBD Collaborators

## Contents

**Supplementary Methods and Author Information** ........ 1

Section 1.1: Relevant meta-data and GATHER compliance ........ 3

Table 1.1: GBD location hierarchy ........ 3

Table 1.2: GATHER checklist ........ 8

Table 1.3: GBD population estimates for ages 15+ ........ 11

Table 1.4: GBD standard population weight estimates ........ 18

Section 1.2: Inclusion and exclusion criteria for exposure data ........ 18

Section 1.3: Product categorization ........ 19

Section 1.4: ST-GPR Methods ........ 25

Section 1.5: Umbrella review for RRs ........ 25

Section 1.6: Burden of Proof Methodology ........ 26

Table 1.5: Bias covariates adjusted for in the final Burden of Proof relative risk model27

Section 1.7: Sex-specific exploration for relative risk estimates ........ 27

Table 1.6: Comparison of sex-specific sensitivity relative risks without between-study heterogeneity from Burden of Proof to the relative risks used in GBD 2021: ........ 28

Section 1.8: TMREL and PAFs ........ 28

## Section 1.1: Relevant meta-data and GATHER compliance

### Table 1.1: GBD location hierarchy

| Hierarchy level | GBD location |
|---|---|
| **Global** | |
| **Super-region** | Central Europe, Eastern Europe, and Central Asia |
| **Region** | Central Asia |
| **Country/Territory** | Armenia |
| **Country/Territory** | Azerbaijan |
| **Country/Territory** | Georgia |
| **Country/Territory** | Kazakhstan |
| **Country/Territory** | Kyrgyzstan |
| **Country/Territory** | Mongolia |
| **Country/Territory** | Tajikistan |
| **Country/Territory** | Turkmenistan |
| **Country/Territory** | Uzbekistan |
| **Region** | Central Europe |
| **Country/Territory** | Albania |
| **Country/Territory** | Bosnia and Herzegovina |
| **Country/Territory** | Bulgaria |
| **Country/Territory** | Croatia |
| **Country/Territory** | Czechia |
| **Country/Territory** | Hungary |
| **Country/Territory** | Montenegro |
| **Country/Territory** | North Macedonia |
| **Country/Territory** | Poland |
| **Country/Territory** | Romania |
| **Country/Territory** | Serbia |
| **Country/Territory** | Slovakia |
| **Country/Territory** | Slovenia |
| **Region** | Eastern Europe |
| **Country/Territory** | Belarus |
| **Country/Territory** | Estonia |
| **Country/Territory** | Latvia |
| **Country/Territory** | Lithuania |
| **Country/Territory** | Republic of Moldova |
| **Country/Territory** | Russian Federation |
| **Country/Territory** | Ukraine |
| **Super-region** | High-income |
| **Region** | Australasia |
| **Country/Territory** | Australia |
| **Country/Territory** | New Zealand |

| | |
|---|---|
| **Region** | High-income Asia Pacific |
| **Country/Territory** | Brunei Darussalam |
| **Country/Territory** | Japan |
| **Country/Territory** | Republic of Korea |
| **Country/Territory** | Singapore |
| **Region** | High-income North America |
| **Country/Territory** | Canada |
| **Country/Territory** | Greenland |
| **Country/Territory** | United States of America |
| **Region** | Southern Latin America |
| **Country/Territory** | Argentina |
| **Country/Territory** | Chile |
| **Country/Territory** | Uruguay |
| **Region** | Western Europe |
| **Country/Territory** | Andorra |
| **Country/Territory** | Austria |
| **Country/Territory** | Belgium |
| **Country/Territory** | Cyprus |
| **Country/Territory** | Denmark |
| **Country/Territory** | Finland |
| **Country/Territory** | France |
| **Country/Territory** | Germany |
| **Country/Territory** | Greece |
| **Country/Territory** | Iceland |
| **Country/Territory** | Ireland |
| **Country/Territory** | Israel |
| **Country/Territory** | Italy |
| **Country/Territory** | Luxembourg |
| **Country/Territory** | Malta |
| **Country/Territory** | Monaco |
| **Country/Territory** | Netherlands |
| **Country/Territory** | Norway |
| **Country/Territory** | Portugal |
| **Country/Territory** | San Marino |
| **Country/Territory** | Spain |
| **Country/Territory** | Sweden |
| **Country/Territory** | Switzerland |
| **Country/Territory** | United Kingdom |
| **Super-region** | Latin America and Caribbean |
| **Region** | Andean Latin America |
| **Country/Territory** | Bolivia (Plurinational State of) |
| **Country/Territory** | Ecuador |

| | |
|---|---|
| **Country/Territory** | Peru |
| **Region** | Caribbean |
| **Country/Territory** | Antigua and Barbuda |
| **Country/Territory** | Bahamas |
| **Country/Territory** | Barbados |
| **Country/Territory** | Belize |
| **Country/Territory** | Bermuda |
| **Country/Territory** | Cuba |
| **Country/Territory** | Dominica |
| **Country/Territory** | Dominican Republic |
| **Country/Territory** | Grenada |
| **Country/Territory** | Guyana |
| **Country/Territory** | Haiti |
| **Country/Territory** | Jamaica |
| **Country/Territory** | Puerto Rico |
| **Country/Territory** | Saint Kitts and Nevis |
| **Country/Territory** | Saint Lucia |
| **Country/Territory** | Saint Vincent and the Grenadines |
| **Country/Territory** | Suriname |
| **Country/Territory** | Trinidad and Tobago |
| **Country/Territory** | United States Virgin Islands |
| **Region** | Central Latin America |
| **Country/Territory** | Colombia |
| **Country/Territory** | Costa Rica |
| **Country/Territory** | El Salvador |
| **Country/Territory** | Guatemala |
| **Country/Territory** | Honduras |
| **Country/Territory** | Mexico |
| **Country/Territory** | Nicaragua |
| **Country/Territory** | Panama |
| **Country/Territory** | Venezuela (Bolivarian Republic of) |
| **Region** | Tropical Latin America |
| **Country/Territory** | Brazil |
| **Country/Territory** | Paraguay |
| **Super-region** | North Africa and Middle East |
| **Region** | North Africa and Middle East |
| **Country/Territory** | Afghanistan |
| **Country/Territory** | Algeria |
| **Country/Territory** | Bahrain |
| **Country/Territory** | Egypt |
| **Country/Territory** | Iran (Islamic Republic of) |
| **Country/Territory** | Iraq |

| | |
|---|---|
| **Country/Territory** | Jordan |
| **Country/Territory** | Kuwait |
| **Country/Territory** | Lebanon |
| **Country/Territory** | Libya |
| **Country/Territory** | Morocco |
| **Country/Territory** | Oman |
| **Country/Territory** | Palestine |
| **Country/Territory** | Qatar |
| **Country/Territory** | Saudi Arabia |
| **Country/Territory** | Sudan |
| **Country/Territory** | Syrian Arab Republic |
| **Country/Territory** | Tunisia |
| **Country/Territory** | Türkiye |
| **Country/Territory** | United Arab Emirates |
| **Country/Territory** | Yemen |
| **Super-region** | South Asia |
| **Region** | South Asia |
| **Country/Territory** | Bangladesh |
| **Country/Territory** | Bhutan |
| **Country/Territory** | India |
| **Country/Territory** | Nepal |
| **Country/Territory** | Pakistan |
| **Super-region** | Southeast Asia, East Asia, and Oceania |
| **Region** | East Asia |
| **Country/Territory** | China |
| **Country/Territory** | Democratic People's Republic of Korea |
| **Country/Territory** | Taiwan |
| **Region** | Oceania |
| **Country/Territory** | American Samoa |
| **Country/Territory** | Cook Islands |
| **Country/Territory** | Fiji |
| **Country/Territory** | Guam |
| **Country/Territory** | Kiribati |
| **Country/Territory** | Marshall Islands |
| **Country/Territory** | Micronesia (Federated States of) |
| **Country/Territory** | Nauru |
| **Country/Territory** | Niue |
| **Country/Territory** | Northern Mariana Islands |
| **Country/Territory** | Palau |
| **Country/Territory** | Papua New Guinea |
| **Country/Territory** | Samoa |
| **Country/Territory** | Solomon Islands |

| | |
|---|---|
| **Country/Territory** | Tokelau |
| **Country/Territory** | Tonga |
| **Country/Territory** | Tuvalu |
| **Country/Territory** | Vanuatu |
| **Region** | Southeast Asia |
| **Country/Territory** | Cambodia |
| **Country/Territory** | Indonesia |
| **Country/Territory** | Lao People's Democratic Republic |
| **Country/Territory** | Malaysia |
| **Country/Territory** | Maldives |
| **Country/Territory** | Mauritius |
| **Country/Territory** | Myanmar |
| **Country/Territory** | Philippines |
| **Country/Territory** | Seychelles |
| **Country/Territory** | Sri Lanka |
| **Country/Territory** | Thailand |
| **Country/Territory** | Timor-Leste |
| **Country/Territory** | Viet Nam |
| **Super-region** | Sub-Saharan Africa |
| **Region** | Central Sub-Saharan Africa |
| **Country/Territory** | Angola |
| **Country/Territory** | Central African Republic |
| **Country/Territory** | Congo |
| **Country/Territory** | Democratic Republic of the Congo |
| **Country/Territory** | Equatorial Guinea |
| **Country/Territory** | Gabon |
| **Region** | Eastern Sub-Saharan Africa |
| **Country/Territory** | Burundi |
| **Country/Territory** | Comoros |
| **Country/Territory** | Djibouti |
| **Country/Territory** | Eritrea |
| **Country/Territory** | Ethiopia |
| **Country/Territory** | Kenya |
| **Country/Territory** | Madagascar |
| **Country/Territory** | Malawi |
| **Country/Territory** | Mozambique |
| **Country/Territory** | Rwanda |
| **Country/Territory** | Somalia |
| **Country/Territory** | South Sudan |
| **Country/Territory** | Uganda |
| **Country/Territory** | United Republic of Tanzania |
| **Country/Territory** | Zambia |

| | |
|---|---|
| **Region** | Southern Sub-Saharan Africa |
| **Country/Territory** | Botswana |
| **Country/Territory** | Eswatini |
| **Country/Territory** | Lesotho |
| **Country/Territory** | Namibia |
| **Country/Territory** | South Africa |
| **Country/Territory** | Zimbabwe |
| **Region** | Western Sub-Saharan Africa |
| **Country/Territory** | Benin |
| **Country/Territory** | Burkina Faso |
| **Country/Territory** | Cabo Verde |
| **Country/Territory** | Cameroon |
| **Country/Territory** | Chad |
| **Country/Territory** | Côte d'Ivoire |
| **Country/Territory** | Gambia |
| **Country/Territory** | Ghana |
| **Country/Territory** | Guinea |
| **Country/Territory** | Guinea-Bissau |
| **Country/Territory** | Liberia |
| **Country/Territory** | Mali |
| **Country/Territory** | Mauritania |
| **Country/Territory** | Niger |
| **Country/Territory** | Nigeria |
| **Country/Territory** | Sao Tome and Principe |
| **Country/Territory** | Senegal |
| **Country/Territory** | Sierra Leone |
| **Country/Territory** | Togo |

## Table 1.2: GATHER checklist

| **Item #** | **Checklist item** | **Reported on page #** |
|---|---|---|
| **Objectives and funding** | | |
| **1** | Define the indicator(s), populations (including age, sex, and geographic entities), and time period(s) for which estimates were made. | Methods (p. 7 and p. 11) |
| **2** | List the funding sources for the work. | "Acknowledgements" |
| **Data Inputs** | | |

| | | |
|---|---|---|
| *For all data inputs from multiple sources that are synthesized as part of the study:* | | |
| **3** | Describe how the data were identified and how the data were accessed. | Methods (p. 8 and p. 10); Appendix Section 1.2 |
| **4** | Specify the inclusion and exclusion criteria. Identify all ad-hoc exclusions. | Methods (p. 8 and p. 10); Appendix Section 1.2; Gil et al., 2023 (ref. 9) |
| **5** | Provide information on all included data sources and their main characteristics. For each data source used, report reference information or contact name/institution, population represented, data collection method, year(s) of data collection, sex and age range, diagnostic criteria or measurement method, and sample size, as relevant. | Publicly available in the GBD 2023 Sources tool (ref. 25) and the Burden of Proof visualizations tool (ref. 27) |
| **6** | Identify and describe any categories of input data that have potentially important biases (e.g., based on characteristics listed in item 5). | Methods (p. 8-11); Appendix Section 1.5; Gil et al., 2023 (ref. 9) |
| *For data inputs that contribute to the analysis but were not synthesized as part of the study:* | | |
| **7** | Describe and give sources for any other data inputs. | N/A |
| *For all data inputs:* | | |
| **8** | Provide all data inputs in a file format from which data can be efficiently extracted (e.g., a spreadsheet rather than a PDF), including all relevant meta-data listed in item 5. For any data inputs that cannot be shared because of ethical or legal reasons, such as third-party ownership, provide a contact name or the name of the institution that retains the right to the data. | Publicly available in the GBD 2023 Sources tool (ref. 25) and the Burden of Proof visualizations tool (ref. 27); Data availability statement |
| **Data analysis** | | |

| | | |
|---|---|---|
| **9** | Provide a conceptual overview of the data analysis method. A diagram may be helpful. | Methods (p. 7) |
| **10** | Provide a detailed description of all steps of the analysis, including mathematical formulae. This description should cover, as relevant, data cleaning, data pre-processing, data adjustments and weighting of data sources, and mathematical or statistical model(s). | Methods (p. 8-9, 11-12); GBD 2023 capstone (ref. 1) |
| **11** | Describe how candidate models were evaluated and how the final model(s) were selected. | Methods (p. 8-9, 11); Gil et al., 2023 (ref. 9) |
| **12** | Provide the results of an evaluation of model performance, if done, as well as the results of any relevant sensitivity analysis. | Gil et al., 2023 (ref. 9); GBD 2023 capstone (ref. 1); Appendix Section 1.6 |
| **13** | Describe methods for calculating uncertainty of the estimates. State which sources of uncertainty were, and were not, accounted for in the uncertainty analysis. | Methods (p. 9, p. 11-12) |
| **14** | State how analytic or statistical source code used to generate estimates can be accessed. | Code availability statement |
| **Results and Discussion** | | |
| **15** | Provide published estimates in a file format from which data can be efficiently extracted. | Appendix 2; GBD Results tool |
| **16** | Report a quantitative measure of the uncertainty of the estimates (e.g. uncertainty intervals). | Appendix 2; Table 1; Table 2; Results (p. 12-16) |
| **17** | Interpret results in light of existing evidence. If updating a previous set of estimates, describe the reasons for changes in estimates. | Discussion (p. 16-18); Research in Context |
| **18** | Discuss limitations of the estimates. Include a discussion of any modelling assumptions or data limitations that affect interpretation of the estimates. | Discussion (p. 18-19) |

Table 1.3: GBD population estimates for ages 15+

| GBD location | 1990 (in thousands) | | | 2023 (in thousands) | | |
|---|---|---|---|---|---|---|
| | Overall | Females | Males | Overall | Females | Males |
| **Global** | 3600000 | 1810000 | 1800000 | 6040000 | 3040000 | 3.00E+06 |
| **Central Europe, Eastern Europe, and Central Asia** | 317000 | 169000 | 148000 | 344000 | 182000 | 162000 |
| **Central Asia** | 44600 | 23200 | 21400 | 70300 | 36000 | 34300 |
| **Armenia** | 2380 | 1240 | 1140 | 2460 | 1330 | 1130 |
| **Azerbaijan** | 4920 | 2580 | 2340 | 8180 | 4180 | 3990 |
| **Georgia** | 4160 | 2240 | 1930 | 2860 | 1520 | 1340 |
| **Kazakhstan** | 11500 | 6060 | 5490 | 14300 | 7490 | 6860 |
| **Kyrgyzstan** | 2790 | 1450 | 1340 | 4880 | 2510 | 2370 |
| **Mongolia** | 1260 | 636 | 625 | 2330 | 1200 | 1120 |
| **Tajikistan** | 3050 | 1550 | 1500 | 6770 | 3360 | 3410 |
| **Turkmenistan** | 2200 | 1130 | 1070 | 3890 | 1910 | 1990 |
| **Uzbekistan** | 12300 | 6270 | 5990 | 24600 | 12500 | 12100 |
| **Central Europe** | 96500 | 49900 | 46700 | 98300 | 50900 | 47500 |
| **Albania** | 2230 | 1090 | 1140 | 2030 | 1040 | 991 |
| **Bosnia and Herzegovina** | 3410 | 1720 | 1690 | 2730 | 1410 | 1320 |
| **Bulgaria** | 6970 | 3570 | 3400 | 5940 | 3080 | 2860 |
| **Croatia** | 3920 | 2050 | 1880 | 3330 | 1740 | 1590 |
| **Czechia** | 8080 | 4220 | 3860 | 9150 | 4680 | 4470 |
| **Hungary** | 8260 | 4360 | 3900 | 8240 | 4330 | 3900 |
| **Montenegro** | 461 | 232 | 228 | 538 | 273 | 265 |
| **North Macedonia** | 1460 | 735 | 729 | 1540 | 778 | 759 |
| **Poland** | 29400 | 15300 | 14100 | 33000 | 17200 | 15800 |
| **Romania** | 17900 | 9210 | 8730 | 16200 | 8440 | 7810 |
| **Serbia** | 7300 | 3700 | 3600 | 7740 | 3880 | 3860 |
| **Slovakia** | 3950 | 2050 | 1900 | 4560 | 2350 | 2210 |
| **Slovenia** | 1560 | 812 | 744 | 1800 | 899 | 901 |
| **Eastern Europe** | 176000 | 95800 | 80200 | 176000 | 95500 | 80200 |
| **Belarus** | 8070 | 4380 | 3690 | 7910 | 4330 | 3580 |
| **Estonia** | 1220 | 664 | 556 | 1130 | 603 | 530 |
| **Latvia** | 2150 | 1170 | 975 | 1600 | 871 | 725 |
| **Lithuania** | 2850 | 1530 | 1320 | 2420 | 1300 | 1110 |

| | | | | | | |
|---|---|---|---|---|---|---|
| **Republic of Moldova** | 3230 | 1730 | 1500 | 3080 | 1630 | 1450 |
| **Russian Federation** | 117000 | 63500 | 53400 | 126000 | 68100 | 57500 |
| **Ukraine** | 41600 | 22900 | 18700 | 33900 | 18600 | 15300 |
| **High-income** | 721000 | 373000 | 349000 | 928000 | 475000 | 453000 |
| **Australasia** | 15700 | 7980 | 7730 | 26300 | 13400 | 12900 |
| **Australia** | 13100 | 6630 | 6440 | 22100 | 11300 | 10800 |
| **New Zealand** | 2640 | 1350 | 1290 | 4150 | 2090 | 2060 |
| **High-income Asia Pacific** | 137000 | 69900 | 66900 | 161000 | 82600 | 78800 |
| **Brunei Darussalam** | 170 | 78.6 | 91.2 | 368 | 173 | 195 |
| **Japan** | 101000 | 52000 | 49200 | 110000 | 57100 | 53100 |
| **Republic of Korea** | 33100 | 16700 | 16400 | 46000 | 23000 | 23000 |
| **Singapore** | 2400 | 1200 | 1200 | 4890 | 2390 | 2500 |
| **High-income North America** | 220000 | 114000 | 106000 | 308000 | 156000 | 151000 |
| **Canada** | 21700 | 11100 | 10600 | 32200 | 16400 | 15800 |
| **Greenland** | 41.3 | 18.5 | 22.8 | 44.9 | 21.1 | 23.9 |
| **United States of America** | 198000 | 103000 | 95500 | 276000 | 140000 | 136000 |
| **Southern Latin America** | 34800 | 18000 | 16800 | 55800 | 29000 | 26800 |
| **Argentina** | 23100 | 11900 | 11100 | 37400 | 19500 | 17900 |
| **Chile** | 9370 | 4850 | 4520 | 15600 | 7980 | 7620 |
| **Uruguay** | 2330 | 1220 | 1120 | 2760 | 1460 | 1300 |
| **Western Europe** | 314000 | 163000 | 151000 | 377000 | 193000 | 183000 |
| **Andorra** | 45 | 21 | 24 | 80.2 | 38.4 | 41.8 |
| **Austria** | 6410 | 3380 | 3040 | 7760 | 3960 | 3790 |
| **Belgium** | 8170 | 4210 | 3960 | 9700 | 4950 | 4750 |
| **Cyprus** | 577 | 289 | 288 | 1140 | 584 | 558 |
| **Denmark** | 4260 | 2170 | 2080 | 4970 | 2510 | 2460 |
| **Finland** | 4040 | 2100 | 1940 | 4730 | 2400 | 2330 |
| **France** | 46500 | 24100 | 22400 | 57700 | 30100 | 27700 |
| **Germany** | 67100 | 35100 | 32000 | 72500 | 37000 | 35500 |
| **Greece** | 8450 | 4320 | 4130 | 9290 | 4770 | 4520 |
| **Iceland** | 190 | 95.3 | 95 | 316 | 153 | 163 |
| **Ireland** | 2640 | 1340 | 1300 | 4300 | 2190 | 2110 |
| **Israel** | 3440 | 1770 | 1670 | 7240 | 3670 | 3580 |

| | | | | | | |
|---|---|---|---|---|---|---|
| **Italy** | 47600 | 24800 | 22800 | 51500 | 26600 | 25000 |
| **Luxembourg** | 315 | 162 | 153 | 554 | 276 | 278 |
| **Malta** | 284 | 146 | 139 | 485 | 229 | 255 |
| **Monaco** | 27.2 | 14.4 | 12.8 | 33.8 | 17.3 | 16.5 |
| **Netherlands** | 12200 | 6210 | 5980 | 15100 | 7620 | 7440 |
| **Norway** | 3440 | 1750 | 1690 | 4560 | 2270 | 2290 |
| **Portugal** | 8070 | 4230 | 3840 | 9210 | 4870 | 4340 |
| **San Marino** | 17.4 | 8.9 | 8.49 | 30.9 | 15.8 | 15.1 |
| **Spain** | 31000 | 16000 | 15000 | 41200 | 21200 | 20100 |
| **Sweden** | 7040 | 3590 | 3440 | 8780 | 4370 | 4400 |
| **Switzerland** | 5710 | 2920 | 2790 | 7650 | 3870 | 3780 |
| **United Kingdom** | 46400 | 24100 | 22300 | 57400 | 29600 | 27800 |
| **Latin America and Caribbean** | 246000 | 126000 | 120000 | 456000 | 236000 | 220000 |
| **Andean Latin America** | 23200 | 11800 | 11400 | 48800 | 24800 | 24000 |
| **Bolivia (Plurinational State of)** | 3710 | 1910 | 1800 | 8660 | 4410 | 4260 |
| **Ecuador** | 6140 | 3120 | 3010 | 13200 | 6870 | 6310 |
| **Peru** | 13400 | 6790 | 6570 | 27000 | 13500 | 13400 |
| **Caribbean** | 24000 | 12300 | 11700 | 36700 | 18800 | 17900 |
| **Antigua and Barbuda** | 42 | 22 | 20 | 74.4 | 38.2 | 36.1 |
| **Bahamas** | 177 | 91.7 | 85.5 | 333 | 174 | 159 |
| **Barbados** | 192 | 102 | 90.7 | 256 | 135 | 121 |
| **Belize** | 105 | 51.9 | 53.2 | 294 | 151 | 143 |
| **Bermuda** | 47.8 | 24.8 | 23 | 55.1 | 28.6 | 26.5 |
| **Cuba** | 8370 | 4180 | 4190 | 9510 | 4820 | 4700 |
| **Dominica** | 49.7 | 25.5 | 24.2 | 56.1 | 27.5 | 28.6 |
| **Dominican Republic** | 4480 | 2320 | 2160 | 8220 | 4180 | 4040 |
| **Grenada** | 63.6 | 32.8 | 30.9 | 90.7 | 44.2 | 46.5 |
| **Guyana** | 487 | 248 | 240 | 597 | 304 | 293 |
| **Haiti** | 3680 | 1910 | 1760 | 8890 | 4620 | 4280 |
| **Jamaica** | 1540 | 794 | 743 | 2250 | 1150 | 1100 |
| **Puerto Rico** | 2640 | 1390 | 1260 | 2920 | 1550 | 1380 |
| **Saint Kitts and Nevis** | 27.5 | 14.2 | 13.3 | 38.8 | 20.3 | 18.5 |
| **Saint Lucia** | 85.8 | 44.7 | 41.1 | 148 | 74.6 | 73.5 |

| | | | | | | |
|---|---|---|---|---|---|---|
| **Saint Vincent and the Grenadines** | 68.9 | 34.9 | 34 | 91.8 | 44.9 | 46.9 |
| **Suriname** | 260 | 129 | 131 | 447 | 230 | 217 |
| **Trinidad and Tobago** | 800 | 401 | 399 | 1130 | 567 | 561 |
| **United States Virgin Islands** | 74.2 | 38.9 | 35.3 | 71.2 | 37 | 34.2 |
| **Central Latin America** | 1.00E+05 | 51500 | 48600 | 197000 | 102000 | 94700 |
| **Colombia** | 20900 | 10700 | 10200 | 40900 | 21000 | 19800 |
| **Costa Rica** | 1920 | 971 | 950 | 4160 | 2080 | 2070 |
| **El Salvador** | 3150 | 1660 | 1490 | 4670 | 2570 | 2100 |
| **Guatemala** | 4340 | 2240 | 2100 | 11300 | 5930 | 5340 |
| **Honduras** | 2500 | 1280 | 1220 | 7210 | 3780 | 3430 |
| **Mexico** | 51800 | 26800 | 25100 | 101000 | 52400 | 48600 |
| **Nicaragua** | 2070 | 1080 | 989 | 4840 | 2490 | 2350 |
| **Panama** | 1560 | 774 | 787 | 3120 | 1580 | 1530 |
| **Venezuela (Bolivarian Republic of)** | 11700 | 5950 | 5770 | 19900 | 10500 | 9430 |
| **Tropical Latin America** | 99200 | 50800 | 48400 | 173000 | 90200 | 83200 |
| **Brazil** | 96900 | 49600 | 47300 | 169000 | 87800 | 80900 |
| **Paraguay** | 2380 | 1190 | 1190 | 4640 | 2340 | 2290 |
| **North Africa and Middle East** | 199000 | 97200 | 102000 | 454000 | 217000 | 237000 |
| **North Africa and Middle East** | 199000 | 97200 | 102000 | 454000 | 217000 | 237000 |
| **Afghanistan** | 5800 | 3070 | 2730 | 19900 | 9910 | 9990 |
| **Algeria** | 14600 | 7280 | 7330 | 31800 | 15800 | 16000 |
| **Bahrain** | 345 | 134 | 211 | 1270 | 441 | 834 |
| **Egypt** | 33200 | 16300 | 17000 | 72400 | 34900 | 37500 |
| **Iran (Islamic Republic of)** | 31700 | 15500 | 16200 | 67800 | 33400 | 34400 |
| **Iraq** | 10300 | 4970 | 5310 | 29500 | 14300 | 15300 |
| **Jordan** | 2120 | 995 | 1120 | 9500 | 4320 | 5190 |
| **Kuwait** | 1150 | 460 | 686 | 3850 | 1370 | 2480 |
| **Lebanon** | 2070 | 1060 | 1010 | 4310 | 2180 | 2130 |
| **Libya** | 2430 | 1120 | 1300 | 5600 | 2710 | 2890 |
| **Morocco** | 15600 | 7890 | 7720 | 28300 | 14000 | 14200 |

| | | | | | | |
|---|---|---|---|---|---|---|
| **Oman** | 1150 | 404 | 743 | 3810 | 1310 | 2500 |
| **Palestine** | 1080 | 550 | 535 | 3460 | 1700 | 1760 |
| **Qatar** | 322 | 87.2 | 234 | 2610 | 715 | 1900 |
| **Saudi Arabia** | 9310 | 3760 | 5560 | 24900 | 8740 | 16200 |
| **Sudan** | 11100 | 5640 | 5450 | 24800 | 12300 | 12600 |
| **Syrian Arab Republic** | 6800 | 3340 | 3460 | 13000 | 6770 | 6280 |
| **Tunisia** | 5240 | 2610 | 2640 | 9270 | 4710 | 4550 |
| **Türkiye** | 36900 | 18400 | 18600 | 67400 | 33900 | 33500 |
| **United Arab Emirates** | 1290 | 376 | 915 | 8720 | 2130 | 6590 |
| **Yemen** | 6540 | 3270 | 3270 | 21300 | 10700 | 10600 |
| **South Asia** | 660000 | 317000 | 343000 | 1380000 | 680000 | 697000 |
| **South Asia** | 660000 | 317000 | 343000 | 1380000 | 680000 | 697000 |
| **Bangladesh** | 60500 | 29300 | 31200 | 124000 | 63300 | 60600 |
| **Bhutan** | 360 | 166 | 194 | 576 | 276 | 300 |
| **India** | 526000 | 253000 | 274000 | 1080000 | 532000 | 549000 |
| **Nepal** | 11000 | 5590 | 5440 | 22400 | 11700 | 10700 |
| **Pakistan** | 62000 | 29200 | 32700 | 150000 | 72600 | 77000 |
| **Southeast Asia, East Asia, and Oceania** | 1190000 | 587000 | 603000 | 1770000 | 880000 | 893000 |
| **East Asia** | 891000 | 434000 | 457000 | 1220000 | 602000 | 619000 |
| **China** | 861000 | 419000 | 442000 | 1180000 | 580000 | 598000 |
| **Democratic People's Republic of Korea** | 14900 | 7740 | 7210 | 21700 | 11200 | 10500 |
| **Taiwan** | 14900 | 7190 | 7690 | 20500 | 10400 | 10000 |
| **Oceania** | 3860 | 1870 | 1990 | 9330 | 4540 | 4790 |
| **American Samoa** | 29.7 | 14.6 | 15.1 | 35.4 | 17.4 | 18 |
| **Cook Islands** | 12.3 | 5.92 | 6.41 | 11 | 5.69 | 5.34 |
| **Fiji** | 479 | 238 | 241 | 665 | 330 | 335 |
| **Guam** | 95.8 | 43.8 | 52 | 123 | 60.9 | 61.9 |
| **Kiribati** | 45 | 23.2 | 21.8 | 84.9 | 43.9 | 41 |
| **Marshall Islands** | 23.5 | 11.5 | 12 | 28.1 | 13.8 | 14.3 |
| **Micronesia (Federated States of)** | 57.3 | 28.3 | 29.1 | 73.2 | 36.1 | 37.1 |
| **Nauru** | 5.78 | 2.82 | 2.97 | 7.88 | 3.92 | 3.96 |

| | | | | | | |
|---|---|---|---|---|---|---|
| **Niue** | 1.52 | 0.771 | 0.744 | 1.29 | 0.679 | 0.615 |
| **Northern Mariana Islands** | 33.2 | 15.4 | 17.8 | 36.8 | 17.6 | 19.3 |
| **Palau** | 10.7 | 5.16 | 5.54 | 14.7 | 6.68 | 8.03 |
| **Papua New Guinea** | 2400 | 1160 | 1240 | 6890 | 3330 | 3560 |
| **Samoa** | 98.9 | 47.1 | 51.8 | 135 | 67.6 | 67.8 |
| **Solomon Islands** | 186 | 89.5 | 96.3 | 548 | 268 | 279 |
| **Tokelau** | 1.02 | 0.521 | 0.498 | 1.21 | 0.599 | 0.608 |
| **Tonga** | 57.3 | 29 | 28.2 | 68.7 | 36.8 | 31.9 |
| **Tuvalu** | 6.05 | 3.29 | 2.75 | 7.56 | 3.7 | 3.86 |
| **Vanuatu** | 85 | 41.6 | 43.3 | 210 | 106 | 104 |
| **Southeast Asia** | 296000 | 151000 | 144000 | 543000 | 274000 | 269000 |
| **Cambodia** | 5620 | 3080 | 2550 | 12200 | 6370 | 5840 |
| **Indonesia** | 118000 | 59900 | 57800 | 220000 | 109000 | 111000 |
| **Lao People's Democratic Republic** | 2330 | 1220 | 1120 | 5240 | 2610 | 2630 |
| **Malaysia** | 11100 | 5550 | 5550 | 26500 | 12600 | 13900 |
| **Maldives** | 117 | 57 | 60.5 | 440 | 163 | 277 |
| **Mauritius** | 770 | 387 | 383 | 1070 | 543 | 529 |
| **Myanmar** | 25800 | 13200 | 12500 | 41600 | 21900 | 19600 |
| **Philippines** | 37900 | 19000 | 18900 | 81400 | 40500 | 40900 |
| **Seychelles** | 49.4 | 24.9 | 24.5 | 83.5 | 37.1 | 46.5 |
| **Sri Lanka** | 11600 | 5800 | 5800 | 17800 | 9330 | 8460 |
| **Thailand** | 40100 | 20400 | 19600 | 56400 | 29700 | 26700 |
| **Timor-Leste** | 448 | 218 | 230 | 920 | 453 | 467 |
| **Viet Nam** | 41800 | 22200 | 19600 | 78100 | 39800 | 38400 |
| **Sub-Saharan Africa** | 267000 | 136000 | 131000 | 708000 | 365000 | 342000 |
| **Central Sub-Saharan Africa** | 29700 | 15200 | 14500 | 83400 | 42100 | 41200 |
| **Angola** | 5590 | 2760 | 2830 | 19800 | 10500 | 9370 |
| **Central African Republic** | 1510 | 787 | 728 | 3520 | 1810 | 1710 |
| **Congo** | 1350 | 699 | 652 | 3740 | 1890 | 1850 |

| | | | | | | |
|---|---|---|---|---|---|---|
| **Democratic Republic of the Congo** | 20400 | 10500 | 9910 | 54000 | 26900 | 27200 |
| **Equatorial Guinea** | 226 | 123 | 103 | 998 | 457 | 540 |
| **Gabon** | 573 | 290 | 283 | 1240 | 646 | 590 |
| **Eastern Sub-Saharan Africa** | 1.00E+05 | 51500 | 48900 | 265000 | 136000 | 129000 |
| **Burundi** | 2940 | 1540 | 1400 | 7980 | 3960 | 4020 |
| **Comoros** | 254 | 130 | 124 | 567 | 283 | 284 |
| **Djibouti** | 226 | 108 | 119 | 911 | 421 | 490 |
| **Eritrea** | 1820 | 947 | 875 | 4320 | 2100 | 2220 |
| **Ethiopia** | 26100 | 13200 | 12900 | 67900 | 34200 | 33800 |
| **Kenya** | 12000 | 6130 | 5870 | 33500 | 17100 | 16400 |
| **Madagascar** | 6450 | 3260 | 3180 | 18400 | 9460 | 8940 |
| **Malawi** | 5240 | 2710 | 2530 | 12200 | 6380 | 5850 |
| **Mozambique** | 7260 | 3930 | 3330 | 18100 | 9690 | 8460 |
| **Rwanda** | 3830 | 2000 | 1830 | 8700 | 4550 | 4150 |
| **Somalia** | 4140 | 2010 | 2130 | 11600 | 5730 | 5870 |
| **South Sudan** | 3200 | 1490 | 1700 | 5830 | 2980 | 2850 |
| **Uganda** | 8890 | 4590 | 4300 | 25200 | 13100 | 12100 |
| **United Republic of Tanzania** | 13900 | 7290 | 6560 | 37900 | 19700 | 18200 |
| **Zambia** | 4180 | 2160 | 2030 | 11800 | 6170 | 5590 |
| **Southern Sub-Saharan Africa** | 31900 | 16800 | 15200 | 63300 | 33000 | 30300 |
| **Botswana** | 738 | 396 | 342 | 1690 | 876 | 816 |
| **Eswatini** | 424 | 232 | 192 | 790 | 404 | 386 |
| **Lesotho** | 848 | 496 | 352 | 1300 | 676 | 622 |
| **Namibia** | 810 | 422 | 389 | 1950 | 1010 | 940 |
| **South Africa** | 23600 | 12300 | 11200 | 48000 | 25000 | 23000 |
| **Zimbabwe** | 5570 | 2900 | 2680 | 9610 | 5120 | 4490 |
| **Western Sub-Saharan Africa** | 105000 | 53000 | 52100 | 296000 | 154000 | 141000 |
| **Benin** | 2430 | 1320 | 1110 | 7990 | 4140 | 3850 |
| **Burkina Faso** | 4800 | 2590 | 2200 | 13300 | 7040 | 6210 |
| **Cabo Verde** | 197 | 108 | 88.8 | 365 | 182 | 183 |
| **Cameroon** | 5560 | 2900 | 2650 | 19700 | 9940 | 9710 |
| **Chad** | 3100 | 1640 | 1460 | 9430 | 4820 | 4610 |

| | | | | | | |
|---|---|---|---|---|---|---|
| **Côte d'Ivoire** | 6500 | 3170 | 3340 | 20700 | 9780 | 10900 |
| **Gambia** | 522 | 262 | 260 | 1500 | 775 | 730 |
| **Ghana** | 8260 | 4260 | 4000 | 21400 | 10900 | 10400 |
| **Guinea** | 3250 | 1730 | 1520 | 7870 | 4180 | 3690 |
| **Guinea-Bissau** | 530 | 280 | 249 | 1250 | 653 | 600 |
| **Liberia** | 1460 | 729 | 735 | 3710 | 1830 | 1880 |
| **Mali** | 4530 | 2370 | 2160 | 12800 | 6490 | 6280 |
| **Mauritania** | 1130 | 583 | 548 | 2700 | 1400 | 1300 |
| **Niger** | 3960 | 2030 | 1930 | 13200 | 6740 | 6410 |
| **Nigeria** | 50600 | 24700 | 25900 | 138000 | 74500 | 64000 |
| **Sao Tome and Principe** | 64.9 | 33.4 | 31.4 | 148 | 73.7 | 73.9 |
| **Senegal** | 3980 | 2070 | 1910 | 11100 | 5620 | 5450 |
| **Sierra Leone** | 2310 | 1200 | 1120 | 5070 | 2570 | 2510 |
| **Togo** | 1880 | 999 | 883 | 5280 | 2770 | 2510 |

### Table 1.4: GBD standard population weight estimates

| Age group name | Age group weight |
|---|---|
| **15 to 19** | 0.082891 |
| **20 to 24** | 0.078012 |
| **25 to 29** | 0.075914 |
| **30 to 34** | 0.073217 |
| **35 to 39** | 0.068281 |
| **40 to 44** | 0.061474 |
| **45 to 49** | 0.055113 |
| **50 to 54** | 0.049131 |
| **55 to 59** | 0.043459 |
| **60 to 64** | 0.036822 |
| **65 to 69** | 0.029851 |
| **70 to 74** | 0.022653 |
| **75 to 79** | 0.015976 |
| **80 plus** | 0.02027 |

## Section 1.2: Inclusion and exclusion criteria for exposure data

Surveys were eligible for inclusion if they contained data on individuals' current use of any smokeless tobacco product, were representative of a GBD location, and included individuals aged 10 years and older surveyed after 1980. All data sources were evaluated for acceptability before inclusion in models. We included sources that included self-

reported data on the use of chewing tobacco, non-chew smokeless tobacco, or all smokeless tobacco products among those aged 10 and older. Sources were required to be representative of the relevant population of a GBD national or sub-national location. Data on the use of chewed products that did not include tobacco, such as local products like betel quid without tobacco, were excluded. Data for 10–14-year-olds and from 1980-1990 are included to inform the trend of chewing tobacco prevalence over time and ages, although they are not reported directly.

## Section 1.3: Product categorization

We ran two distinct ST-GPR models using chewing tobacco-specific data and non-chewed tobacco data, respectively. These estimates were then used to produce a corresponding age-, sex-, location-, and year-specific ratio of chewing tobacco use to combined chewing and non-chewing tobacco use. We applied the corresponding ratio to each data point reporting smokeless tobacco use and added the variance of the ratio to ensure the data reflect only chewing tobacco use and account for the additional uncertainty introduced through this adjustment. This intermediate step adjusts data from 65.0% of the surveys included that reported only on broad smokeless tobacco use to ensure that the resulting prevalence estimates reflect only chewing tobacco use.

Data cleaning also included splitting data available only in aggregated age or sex groups to the ages and sexes relevant to GBD estimation. We leveraged sources with data in the standard 5-year age bins separated for females and males to derive product category-specific age and sex patterns of use by location and year. These observed patterns were then applied to the aggregated data points to transform these data into relevant demographic groups that can be used in the consecutive modelling steps.

| Product | Location specific | Overarching type | What is it? | Mode of use | Notes |
|---|---|---|---|---|---|
| **Paan, pan, betel quid, pan masala or betel nut** | Southeast Asia | SLT; chew | Generally contains betel leaf, slaked lime and areca nut, sometimes spices such as clove, saffron, aniseed, turmeric, | Placed in the mouth and chewed. | We are only interested in paan WITH tobacco. |

| | | | | | |
|---|---|---|---|---|---|
| | | | mustard, or other sweeteners are added. Tobacco is optional. | | |
| **Khaini, khainee, or surti** | India, Nepal and Bangladesh primarily | SLT | Sun-dried or fermented tobacco leaves with slaked lime. | Placed in-between gum and cheek and sucked. | |
| **Guthka or gutka** | Primarily India, and Indians living in Canada, America or the UK | SLT; chew | Areca nut, slaked lime, catechu and sun-dried, roasted, finely chopped tobacco with flavorings and sweeteners. | Held in mouth and chewed or sucked. | |
| **Zarda** | India, Bangladesh, Pakistan, Nepal and Myanmar | SLT; chew | Flaked chewing tobacco flavored with spices, herbs, etc. Often used in betel quid or mixed with with or areca nut. | Chewed | |
| **Sada Pata or chadha** | India, Bangladesh, Bhutan and Myanmar | SLT; chew | Air cured tobacco. Sometimes used in betel quid. | Chewed or held in mouth. | |

| | | | | | |
|---|---|---|---|---|---|
| **Gul or qul** | India and Bangladesh | SLT | Powdered tobacco product containing ash of tendu leaves sold in cans. | Applied to teeth and gums. | |
| **Mishri** | India, primarily Gujarat and Maharashtra. | SLT | Roasted, powdered tobacco. | Applied to gums. | |
| **Mawa** | India, primarily Gujarat and Maharashtra. | SLT; chew | Thin areca nut shavings with slaked lime and tobacco flakes. | Chewed | |
| **Khat, chat, qaad, qat** | Saudi Arabia, Yemen, and Somalia primarily | SLT; chew | Stimulant drug made from the leaves and twigs, evergreen shrub. Active ingredients are cathine and cathinone. | Typically chewed like tobacco — retained in the cheek and chewed — and can also be smoked | Only interested in Khat WITH tobacco |
| **Nasvai, nasvay, nas, naas, or naswar** | Afghanistan, Pakistan, Iran, UAE, South Africa, Turkmenistan, Kyrgyzstan, Uzbekistan | SLT | A mixture of powdered tobacco, ash, cotton or sesame oil, water, and sometimes lime or gum. Similar to dip or snus. | Rolled into a ball and placed under the tongue or in the cheek. Held in the mouth and sucked on. | |

| | | | | | |
|---|---|---|---|---|---|
| **Dry snuff, nossi, or suret** | USA, Europe, India | SLT | Dry, powdered tobacco. | Oral or nasal use. | |
| **Wet snuff or dip** | USA, Canada, Mexico, South Africa | SLT | Moist tobacco that's often flavored. | Held in the mouth, normally users spit. | |
| **Snus** | Nordic countries (Sweden, Norway, Iceland, Finland, Denmark), USA, Canada, Brazil, South Africa | SLT | Tobacco, moisturizers, salt, sodium carbonate, and sweeteners or flavorings. | Placed between lip and gum for long periods of time (no spitting). | Similar but not the same as dip in America. |
| **Man pori, mainpuri, kapoori** | India/Pakistan, primarily in the Mainpuri district of Uttar Pradesh | SLT; chew | Mixture of finely cut betel nut and small pieces of tobacco leaves treated in slaked lime and flavoring agents such as powdered cloves, cardamom, Kewara and sandalwood powder. Catechu is sometimes used. | Chewed and smoked | |

| | | | | | |
|---|---|---|---|---|---|
| **Rapé** | Brazil; Has cultural significance among aboriginals. | SLT | Dried tobacco leaf typically flavored using tonka bean, clove, cinnamon, camphor or ashes from selected trees. | Inhaled through the nose | |
| **Kouben** | Palau | Unknown | This source, which may not be reliable, is the only indication that kouben should be considered a form of chewing tobacco because it refers to setting up "a plan for one of (their) investigators to stop chewing kouben." All other sources seem to refer to kouben only in a list with other forms of tobacco or as a drug. | | |
| **Maras powder** | Turkey | SLT | The powder is typically mixed with wood ash, | Applied between the gums | |

| | | | | | |
|---|---|---|---|---|---|
| | | | such as that of oak, walnut or grapevine. | and lips or cheek and kept in the mouth for 5 minutes-hours. | |
| **Toombak** | Sudan | SLT | A coarse powder of dried toombak leaves is mixed with a concentrated solution of natron (sodium bicarbonate) in water until the product is moist and hardened. This product is kept in firmly sealed tin containers for about 2 hr, then it is ready for sale or use and is also called toombak. | Placed between gum and lip or cheeks (dipping) for a few minutes to several hours | |
| **Shamma(h)** | Arabic Penninsula countries | SLT | | Placed between gum and lip or cheeks (dipping) for a few minutes to | |

| | | | | | |
|---|---|---|---|---|---|
| | | | | several hours | |

## Section 1.4: ST-GPR Methods

We used a two-phase approach using ST-GPR models at each stage to derive prevalence of chewing tobacco product use. This modelling approach used a linear model with a global intercept and fixed effects by age. Then, it used the residuals of this first-stage model weighed by distance in time, age, and location to account for data-driven spatiotemporal variation and borrow strength from data in similar geographic regions or age groups. In the final step, the model quantified the uncertainty of the estimates as a product of the uncertainty in the inputs. Through the posterior distribution of the Gaussian process that characterizes this final step of ST-GPR, we produced 1 000 draws for each estimation unit, which were propagated forward to estimate attributable burden and used to calculate the reported mean, 2.5$^{th}$, and 97.5$^{th}$ percentiles of prevalence.

## Section 1.5: Umbrella review for RRs

Systematic reviews were conducted separately for ischemic heart disease, stroke and head and neck cancers. These outcomes were selected through an umbrella review of existing meta-analyses that identified them as relevant outcomes related to chewing tobacco beyond the two that were associated with chewing tobacco in prior GBD cycles. We conducted an internal umbrella review in March 2021 to identify health outcomes potentially related to chewing tobacco with sufficient published data to merit the investment of extensive resources in a full systematic review. We searched PubMed using the following search string:

("smokeless tobacco"[tiab] OR "Tobacco, Smokeless"[Mesh] OR "Dipping Tobacco"[tiab] OR "Oral Tobacco"[tiab] OR bajjar[tiab] OR ("betel quid"[tiab] AND tobacco[tiab]) OR "chewing tobacco"[tiab] OR chimó[tiab] ORsnuff[tiab] OR snuif[tiab] OR dip[tiab] OR dohra[tiab] OR gudakhu[tiab] OR gul[tiab] OR gutka[tiab] OR gutkha[tiab] OR "hnat hsey"[tiab] OR iq'mik[tiab] OR khaini[tiab] OR kharra[tiab] OR khiwam[tiab] OR khimam[tiab] OR kiwam[tiab] ORkimam[tiab] OR "lal dant manjan"[tiab] OR ("loose leaf"[tiab] AND (chew[tiab] OR tobacco[tiab])) OR mainpuri[tiab] OR maras[tiab] OR mawa[tiab] OR mshri[tiab] OR naffa[tiab] OR nas[Supplementary Concept] OR ((nas[tiab] OR nass[tiab]) AND tobacco[tiab]) OR naswar[tiab] OR nasway[tiab] OR nasvay[tiab] OR neffa[tiab] OR((pan[tiab] OR paan[tiab]) AND tobacco[tiab]) OR

(plug[tiab] AND tobacco[tiab]) OR (rapé[tiab] AND tobacco[tiab]) OR ((red[tiab] OR tobacco[tiab]) AND (toothpowder[tiab] OR toothpaste[tiab])) OR shammah[tiab] OR snus[tiab] OR taaba[tiab] OR tapkeer[tiab] OR tawa[tiab] OR tombol[tiab] OR toombak[tiab] OR tuibur[tiab] OR "tobacco water"[tiab] OR (twist[tiab] AND tobacco[tiab]) OR zarda[tiab]) AND ("Risk"[Mesh] OR "relative risk"[tiab] OR "hazard ratio"[tiab] OR"odds ratio"[tiab] OR "rate"[tiab] OR "risk"[tiab]) AND ("Cohort Studies"[Mesh] OR "Prospective Studies"[Mesh] OR "Cohort"[tiab] OR "prospective"[tiab] OR "longitudinal"[tiab] OR "Case-Control Studies"[Mesh] OR "case-control"[tiab] OR "case control"[tiab] OR "case-crossover"[tiab]) AND ("1970/01/01"[PDat] : "2021/12/31"[PDat]) NOT (animals[MeSH] NOT humans[MeSH])

It yielded 1,102 hits of which 45 were identified as potentially relevant meta-analyses/systematic reviews. These 45 studies were categorized based on the outcomes of interest described in the publication. For the outcomes with more than one systematic review/meta-analysis, we conducted an in-depth evaluation of the available evidence described and the overall findings. These outcomes included pancreatic cancer, diabetes, and dental caries, but these were determined to have insufficient data at the time of the review to be merit the use of the Burden of Proof approach. However, head and neck cancers, including oral cancer and esophageal cancer (existing GBD risk-outcome pairs with chewing tobacco), and cardiovascular diseases were found to have a broad swath of potentially useful data. Furthermore, the findings of existing meta-analyses were mixed, indicating both that a full systematic review was feasible and that the evidence warranted further investigation with a more comprehensive meta-analytic framework to parse through diverse publications.

As a result of this effort, we opted to conduct systematic reviews for the grouping of head and neck cancers, expanding to three more than previously investigated in relation to chewing tobacco in the GBD, and for stroke and ischemic heart disease as leading cardiovascular diseases.

## Section 1.6: Burden of Proof Methodology

The Burden of Proof methodology applies a likelihood-based approach to trimming 10% of outliers for models with more than 10 data points and tests potential biases for inclusion in the final model to account for significant and detectable sources of variation.

The following potential sources of bias were evaluated in our Burden of Proof meta-analysis:

- Geographical representativeness
- Representativeness of the sample relative to the target population

- Degree of adjustment of the effect size
- The temporal element of the exposed group's use of chewing tobacco (e.g. current chewers or ever chewers)
- Chewing tobacco product sub-types
- Methodology used to ascertain the outcome of interest
- Whether the effect size was derived from the entire sample or from a specific sub-group after sampling.
- The use of aggregate outcome definitions for head and neck cancers

Only the following were selected for use in the models based on algorithmic bias detection:

### Table 1.5: Bias covariates adjusted for in the final Burden of Proof relative risk model

| Health outcome | Bias covariates selected from the Lasso regression |
|---|---|
| **Stroke** | None were selected |
| **Esophageal cancer** | Level of adjustment |
| **Lip and oral cavity cancer** | Chewing tobacco product sub-type; Sub-population analysis |
| **Laryngeal cancer** | Aggregate outcome definition; Level of adjustment |
| **Nasopharyngeal cancer** | Level of adjustment |
| **Other pharynx cancer** | Aggregate outcome definition; Level of adjustment |

The Burden of Proof methodology quantifies the remaining unexplained between-study heterogeneity and produces two estimates of uncertainty: Confidence intervals without between-study heterogeneity reflecting conventional uncertainty measures and confidence intervals with between-study heterogeneity, which fully accounts for the consistency (or lack thereof) observed across available data. Supplementary Burden of Proof metrics are also generated that reflect conservative measures of the health risks associated with chewing tobacco and the strength of the available evidence.

## Section 1.7: Sex-specific exploration for relative risk estimates

In GBD 2021 and prior, we used sex-specific relative risks for the association between chewing tobacco and lip and oral cavity cancer and esophageal cancer. As such, we explored the possibility of using sex-specific relative risks produced by the updated Burden

of Proof methodology. We require a minimum of 3 studies to inform relative risk estimates for GBD and the Burden of Proof methodology, so we were only able to run sex-specific relative risks when there were 3 or more studies reporting sex-specific effect sizes to use in the stratified models.

### Table 1.6: Comparison of sex-specific sensitivity relative risks without between-study heterogeneity from Burden of Proof to the relative risks used in GBD 2021:

| Health outcome | Burden of Proof results for GBD 2023 | | GBD 2021 and 2019 estimates | |
|---|---|---|---|---|
| | Male | Female | Male | Female |
| **Stroke** | 1.70 (1.10–2.63) | N/A | N/A | N/A |
| **Esophageal cancer** | 1.64 (1.21–2.23) | 2.14 (1.35–3.40) | 2.57 (1.85–3.42) | 2.58 (1.88–3.48) |
| **Lip and oral cavity cancer** | 3.42 (3.53–4.62) | 5.45 (3.21–9.27) | 3.02 (2.02–4.47) | 8.09 (5.46–11.77) |
| **Laryngeal cancer** | 1.75 (1.30–2.35) | N/A | N/A | N/A |
| **Nasopharyngeal cancer** | 2.51 (1.51–4.18) | 1.96 (1.00–3.85) | N/A | N/A |
| **Other pharynx cancer** | 1.66 (1.27–2.19) | 1.96 (1.00–3.85) | N/A | N/A |

## Section 1.8: TMREL and PAFs

For chewing tobacco, specifically, PAFs are generated based on the assumption that the lowest feasible exposure level that results in the least disease risk (in other words, the theoretical minimum risk exposure level) is no chewing tobacco or a prevalence of 0. As a result, the PAF formula simplifies as follows:

$$PAF = \frac{P(RR - 1)}{[P(RR - 1) + 1]}$$

Using draw-level calculations allowed for the propagation of statistical uncertainty throughout the estimation process, with the 1 000 draws of exposure estimates being down sampled to match the 250 relative risk and resulting 250 PAF draws. This process has been shown to minimally affect the propagated uncertainty when tested for other risk factors. These simulations demonstrated that using 250 draws for the final modeling stages consistently captures the "true" distribution and does not lead to inappropriately narrow uncertainty estimates, even for risk factors with wide exposure UIs.

To help assess the impact of reduced draws, our mathematical team conducted the following experiment. They first generated 1000 samples from a random variable $X \sim N(30,10^2)$ to be used as the "true" distribution. For each n depicted in the plot below, they subset n samples with which they calculated the sample standard deviation. They then estimated a 95% uncertainty interval $30 \pm 1.96s$ and computed the percent of the "true" distribution covered by that 95% uncertainty interval. For each n, these steps were conducted 1000 times. The distribution of coverages from those 1000 simulations for each number of draws is shown below in green:

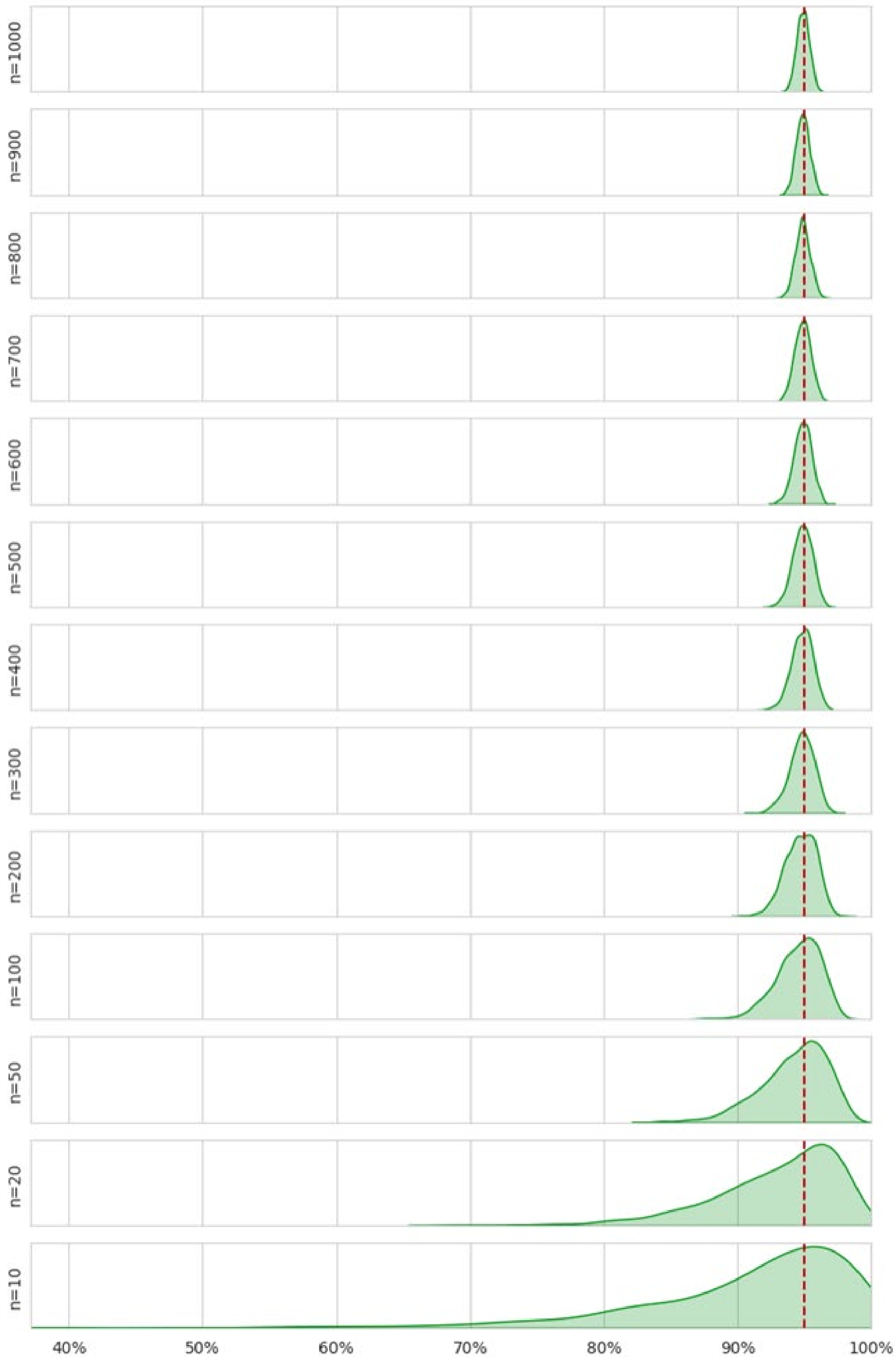

n=1000
n=900
n=800
n=700
n=600
n=500
n=400
n=300
n=200
n=100
n=50
n=20
n=10
40%
50%
60%
70%
80%
90%
100%
