# Supplementary material for: Global, regional, and national burden of exposure to chewing tobacco, 1990–2023: a systematic analysis of the Global Burden of Disease Study 2023
Source: Lancet Glob Health. 2026 Sep 30;14(11):None. doi: 10.1016/j.langlo.2026.104043 (PMC13755197; doi:10.1016/j.langlo.2026.104043)

THE LANCET
Global Health

Supplementary appendix 2

This appendix formed part of the original submission and has been peer reviewed. We post it as supplied by the authors.

Supplement to: Global, regional, and national burden of exposure to chewing tobacco, 1990–2023: a systematic analysis of the Global Burden of Disease Study 2023. *Lancet Glob Health* 2026. https://doi.org/10.1016/j.langlo.2026.104043

Supplementary Results

Global, regional, and national burden of exposure to chewing tobacco, 1990-2023: a systematic analysis of the Global Burden of Disease Study 2023

GBD Collaborators

Table of Contents

Appendix Figure 2·1: Year of most recent survey of chewing tobacco use, by country 1

Appendix Table 2·1: Ratio of chewing tobacco use to smokeless tobacco use, by age, sex, and region, 2023 2

Appendix Figure 2·2: Age-standardized ratio of chewing tobacco use to smokeless tobacco use, age 15+, by sex, 2023 7

Appendix Table 2·2: Burden of Proof results for health outcomes attributable to chewing tobacco use 8

Appendix Table 2·3: Number of users 1990 and 2023, ages 15+ 9

Appendix Table 2·4: All-age prevalence of chewing tobacco use in 1990 and 2023, among ages 15+ 24

Appendix Table 2·5: Age-standardized prevalence of chewing tobacco use in 1990 and 2023, among ages 15+ 38

Appendix Figure 2·3: Prevalence of chewing tobacco use by age and sex, among ages 15+, global and South Asia, 2023 52

Appendix Figure 2·4: Number of new users of chewing tobacco by region, among ages 15+, comparing 1990 and 2023 53

Appendix Table 2·6: Number of deaths attributable to chewing tobacco use in 1990 and 2023, among ages 15+ 54

Appendix Table 2·7: Age-standardized death rate attributable to chewing tobacco use in 1990 and 2023, among ages 15+ 69

Appendix Table 2·8: Number of YLL attributable to chewing tobacco use in 1990 and 2023, among ages 15+ 83

Appendix Table 2·9: Age-standardized YLL rate attributable to chewing tobacco use in 1990 and 2023, among ages 15+ 98

Appendix Table 2·10: Number of YLD attributable to chewing tobacco use in 1990 and 2023, among ages 15+ 112

Appendix Table 2·11: Age-standardized YLD rate attributable to chewing tobacco use in 1990 and 2023, among ages 15+ 127

Appendix Table 2·12: Number of DALYs attributable to chewing tobacco use in 1990 and 2023, among ages 15+ 142

Appendix Table 2·13: Age-standardized DALY rate attributable to chewing tobacco use in 1990 and 2023, among ages 15+ 157

Appendix Figure 2·5: DALY rates attributable to chewing tobacco use (per 100,000), by measure and region, among ages 15+, comparing 1990 and 2023 172

Appendix Table 2·14: Cause-specific DALY rates attributable to chewing tobacco use in 2023, among ages 15+, by super-region 173

Appendix Table 2·15: Number and proportion of total DALYs for each cause attributable to chewing tobacco, among ages 15+, in 2023 175

Appendix Figure 2·6: Cause-specific DALY rates attributable to chewing tobacco use in 2023, among ages 15+, by sex and South Asian nation 179

Appendix Figure 2·7: Age-standardized rate of DALYs attributable to chewing tobacco use, age 15+, by sex, 2023 180

Appendix Figure 2·8: Ratio of DALYs attributable to chewing tobacco use to DALYs attributable to smoking, age 30+, by sex, 2023 181

Appendix Figure 2·1: Year of most recent survey of chewing tobacco use, by country

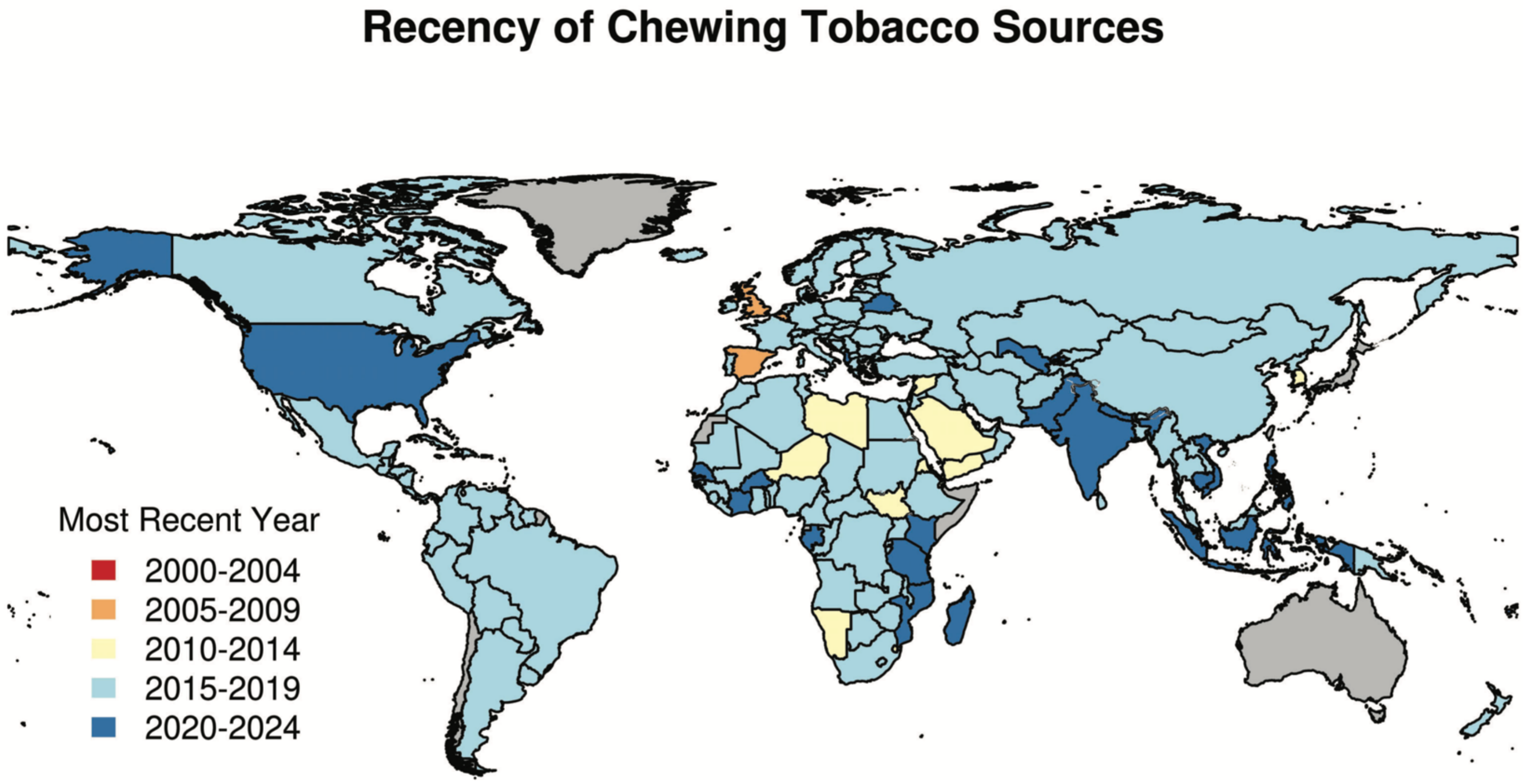

Appendix Table 2·1: Ratio of chewing tobacco use to smokeless tobacco use, by age, sex, and region, 2023

	Age Group	Female	Male
**Global**			
	10 to 14	0·573	0·719
	15 to 19	0·587	0·664
	20 to 24	0·620	0·677
	25 to 29	0·637	0·679
	30 to 34	0·657	0·675
	35 to 39	0·657	0·686
	40 to 44	0·647	0·687
	45 to 49	0·662	0·687
	50 to 54	0·659	0·698
	55 to 59	0·609	0·686
	60 to 64	0·547	0·713
	65 to 69	0·528	0·658
	70 to 74	0·457	0·663
	75 to 79	0·421	0·637
	80+	0·409	0·582
**Southeast Asia, East Asia, and Oceania**			
	10 to 14	0·638	0·725
	15 to 19	0·623	0·644
	20 to 24	0·644	0·662
	25 to 29	0·681	0·661
	30 to 34	0·739	0·669
	35 to 39	0·764	0·701
	40 to 44	0·782	0·716
	45 to 49	0·801	0·715
	50 to 54	0·769	0·744
	55 to 59	0·670	0·741
	60 to 64	0·572	0·773
	65 to 69	0·515	0·658
	70 to 74	0·413	0·631
	75 to 79	0·382	0·601
	80+	0·373	0·561

	Age Group	Female	Male
**Central Europe, Eastern Europe, and Central Asia**			
	10 to 14	0·418	0·520
	15 to 19	0·441	0·426
	20 to 24	0·464	0·428
	25 to 29	0·461	0·441
	30 to 34	0·466	0·447
	35 to 39	0·459	0·506
	40 to 44	0·453	0·554
	45 to 49	0·464	0·573
	50 to 54	0·472	0·555
	55 to 59	0·466	0·519
	60 to 64	0·462	0·578
	65 to 69	0·511	0·582
	70 to 74	0·470	0·705
	75 to 79	0·478	0·632
	80+	0·505	0·544
**High-income**			
	10 to 14	0·264	0·497
	15 to 19	0·228	0·356
	20 to 24	0·272	0·350
	25 to 29	0·298	0·378
	30 to 34	0·346	0·403
	35 to 39	0·323	0·387
	40 to 44	0·308	0·411
	45 to 49	0·322	0·417
	50 to 54	0·309	0·442
	55 to 59	0·289	0·435
	60 to 64	0·263	0·501
	65 to 69	0·277	0·505
	70 to 74	0·224	0·555
	75 to 79	0·210	0·540
	80+	0·226	0·492
**Latin America and Caribbean**			

	Age Group	Female	Male
	10 to 14	0·412	0·527
	15 to 19	0·456	0·486
	20 to 24	0·478	0·477
	25 to 29	0·473	0·462
	30 to 34	0·469	0·447
	35 to 39	0·463	0·450
	40 to 44	0·457	0·446
	45 to 49	0·470	0·434
	50 to 54	0·484	0·420
	55 to 59	0·495	0·368
	60 to 64	0·511	0·445
	65 to 69	0·585	0·459
	70 to 74	0·552	0·559
	75 to 79	0·581	0·586
	80+	0·593	0·579
**North Africa and Middle East**			
	10 to 14	0·425	0·484
	15 to 19	0·447	0·345
	20 to 24	0·455	0·321
	25 to 29	0·437	0·307
	30 to 34	0·429	0·286
	35 to 39	0·414	0·287
	40 to 44	0·395	0·275
	45 to 49	0·410	0·289
	50 to 54	0·441	0·261
	55 to 59	0·429	0·243
	60 to 64	0·374	0·293
	65 to 69	0·352	0·241
	70 to 74	0·341	0·263
	75 to 79	0·359	0·254
	80+	0·399	0·218
**South Asia**			
	10 to 14	0·749	0·955

	Age Group	Female	Male
	15 to 19	0·766	0·940
	20 to 24	0·815	0·961
	25 to 29	0·835	0·967
	30 to 34	0·832	0·968
	35 to 39	0·820	0·973
	40 to 44	0·815	0·964
	45 to 49	0·810	0·967
	50 to 54	0·826	0·960
	55 to 59	0·830	0·947
	60 to 64	0·813	0·953
	65 to 69	0·822	0·943
	70 to 74	0·813	0·941
	75 to 79	0·738	0·926
	80+	0·780	0·903
**Sub-Saharan Africa**			
	10 to 14	0·348	0·442
	15 to 19	0·396	0·351
	20 to 24	0·408	0·347
	25 to 29	0·390	0·344
	30 to 34	0·377	0·317
	35 to 39	0·347	0·327
	40 to 44	0·317	0·316
	45 to 49	0·311	0·300
	50 to 54	0·302	0·305
	55 to 59	0·263	0·305
	60 to 64	0·257	0·330
	65 to 69	0·251	0·302
	70 to 74	0·191	0·332
	75 to 79	0·209	0·281
	80+	0·221	0·202
	10 to 14	0·655	0·752
	15 to 19	0·637	0·680

Age Group	Female	Male
20 to 24	0·659	0·667
25 to 29	0·697	0·672
30 to 34	0·747	0·686
35 to 39	0·774	0·720
40 to 44	0·793	0·743
45 to 49	0·810	0·747
50 to 54	0·771	0·771
55 to 59	0·665	0·768
60 to 64	0·552	0·796
65 to 69	0·495	0·668
70 to 74	0·399	0·633
75 to 79	0·372	0·600
80+	0·370	0·557

Appendix Figure 2·2: Age-standardized ratio of chewing tobacco use to smokeless tobacco use, age 15+, by sex, 2023

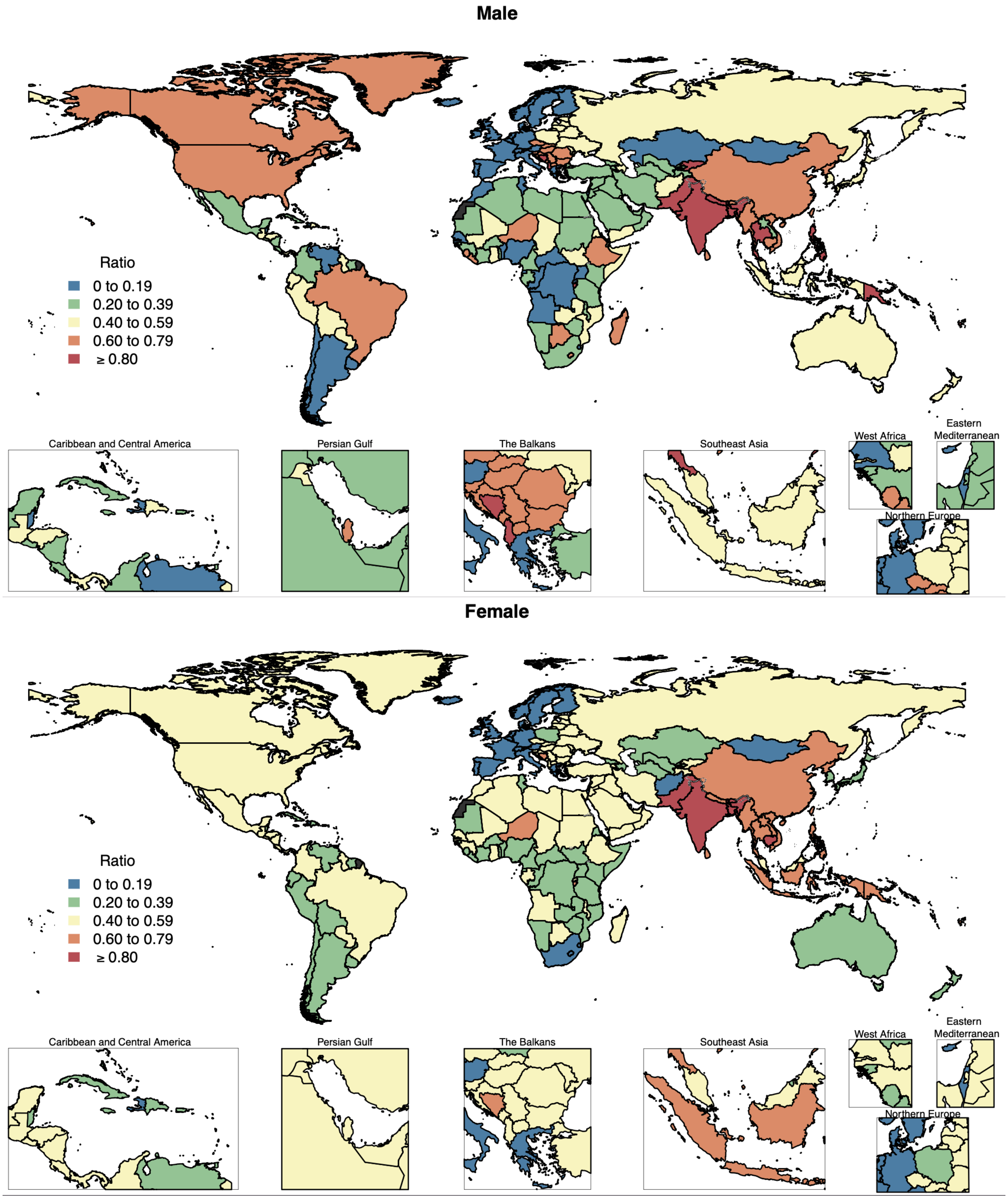

Appendix Table 2·2: Burden of Proof results for health outcomes attributable to chewing tobacco use

Health Outcome	RR (95% UI without γ)	RR (95% UI with γ)	BPRF	Star rating	No. of studies	Data points
Stroke	1·46 (1·28, 1·68)	1·46 (1·11, 1·93)	1·16	☆☆	3	4
Esophageal Cancer	2·14 (1·77, 2·57)	2·14 (0·89, 5·15)	1·02	☆☆	22	27
Lip and oral cavity cancer	3·64 (3·00, 4·41)	3·64 (0·66, 19·95)	0·87	☆	70	95
Larynx cancer	2·66 (1·98, 3·57)	2·66 (0·52, 13·63)	0·68	☆	24	27
Nasopharynx cancer	2·50 (1·79, 3·49)	2·50 (0·49, 12·66)	0·64	☆	17	21
Other pharynx cancer	2·33 (1·80, 3·01)	2·33 (0·45, 12·04)	0·59	☆	31	38
Ischemic heart disease	1·30 (0·88, 1·92)	1·30 (0·29, 5·83)	N/A		8	9

Appendix Table 2·3: Number of users 1990 and 2023, ages 15+

	Female			Male			Both		
	Number of users (thousands)		Change (%)	Number of users (thousands)		Change (%)	Number of users (thousands)		Change (%)
	1990	2023		1990	2023		1990	2023	
Global	44800 (21700–81200)	77800 (43400–130000)	73·6 (-23·9–338)	106000 (60800–167000)	164000 (119000–227000)	54·5 (-9·9–194)	151000 (83400–246000)	241000 (165000–355000)	60·2 (-13·4–228)
**Central Europe, Eastern Europe, and Central Asia**	435 (153–958)	515 (193–1110)	18·3 (-61·8–314)	883 (347–1820)	1360 (580–2670)	54 (-45·2–382)	1320 (499–2780)	1870 (777–3790)	42·2 (-50·4–369)
Central Asia	71·7 (25·1–157)	117 (44–252)	63·3 (-47·1–474)	426 (171–861)	843 (372–1610)	98·1 (-25·6–507)	497 (199–1010)	960 (414–1850)	93·1 (-29·3–514)
Armenia	2·71 (0·945–5·94)	2·51 (0·939–5·41)	-7·1 (-70–225)	3·08 (1·14–6·54)	3·03 (1·19–6·29)	-1·9 (-66·5–223)	5·79 (2·09–12·5)	5·54 (2·13–11·7)	-4·3 (-68·1–224)
Azerbaijan	4·79 (1·67–10·5)	7·65 (2·86–16·5)	59·7 (-48·5–459)	8·82 (3·27–18·7)	14·2 (5·6–29·4)	60·9 (-45–429)	13·6 (4·94–29·2)	21·8 (8·46–45·9)	60·5 (-46·2–439)
Georgia	5·82 (2·04–12·8)	4·38 (1·64–9·41)	-24·8 (-75·7–163)	5·9 (2·19–12·5)	3·94 (1·56–8·19)	-33·2 (-77·2–120)	11·7 (4·22–25·3)	8·32 (3·19–17·6)	-29 (-76·4–140)
Kazakhstan	12·1 (4·23–26·6)	13·2 (4·92–28·3)	8·8 (-64·9–281)	24·9 (9·24–52·8)	30·9 (12·2–64)	23·8 (-57·6–307)	37 (13·5–79·4)	44 (17·1–92·4)	18·9 (-60–299)
Kyrgyzstan	6·62 (2·32–14·5)	9·26 (3·46–19·9)	40 (-54·8–389)	84·7 (37·7–160)	169 (88·4–296)	100 (-21·9–483)	91·3 (40·4–174)	179 (92·4–320)	95·8 (-24·3–479)
Mongolia	1·73 (0·606–3·8)	2·79 (1·04–5·99)	60·9 (-48–464)	2·25 (0·835–4·77)	3·49 (1·38–7·25)	55·1 (-46·9–413)	3·98 (1·44–8·56)	6·28 (2·42–13·2)	57·6 (-47·4–434)
Tajikistan	8·29 (2·92–18·1)	21 (7·95–44·8)	153 (-17·7–795)	9·69 (3·6–20·5)	20·8 (8·26–43·1)	115 (-26·2–608)	18 (6·52–38·6)	41·8 (16·2–87·9)	133 (-22·2–694)
Turkmenistan	2·02 (0·707–4·45)	3·23 (1·2–6·94)	59·4 (-48·6–458)	3·26 (1·21–6·91)	5·61 (2·21–11·7)	72 (-41·2–465)	5·29 (1·91–11·4)	8·84 (3·42–18·6)	67·2 (-44·1–462)
Uzbekistan	27·6 (9·65–60·4)	53 (19·8–114)	92·4 (-37·8–573)	283 (110–573)	591 (248–1170)	109 (-24·1–561)	310 (119–633)	644 (268–1280)	108 (-25·4–562)
Central Europe	109 (38·8–244)	154 (58·5–336)	41·3 (-54·5–393)	170 (64·1–364)	246 (99·3–518)	45·1 (-50·1–379)	279 (103–607)	400 (158–854)	43·6 (-51·8–384)
Albania	4·26 (1·49–9·33)	3·51 (1·31–7·52)	-17·8 (-73·4–187)	14·4 (5·48–30)	11·3 (4·57–23·3)	-21·2 (-72·7–157)	18·7 (6·97–39·3)	14·9 (5·88–30·8)	-20·4 (-72·9–165)
Bosnia and Herzegovina	6·4 (2·24–14)	4·76 (1·78–10·2)	-25·6 (-75·9–159)	12·3 (4·59–25·8)	8·97 (3·56–18·5)	-27 (-74·8–138)	18·7 (6·83–39·8)	13·7 (5·34–28·7)	-26·5 (-75·2–145)

Return to Table of Contents

	Female			Male			Both		
	Number of users (thousands)		Change (%)	Number of users (thousands)		Change (%)	Number of users (thousands)		Change (%)
	1990	2023		1990	2023		1990	2023	
Bulgaria	12·5 (4·37–27·4)	16·4 (6·15–35·1)	31·2 (-57·5–361)	21·6 (8·05–45·5)	29·5 (11·8–60·7)	36·8 (-52·9–354)	34·1 (12·4–72·9)	45·9 (18–95·8)	34·8 (-54·5–358)
Croatia	4·09 (1·43–8·98)	3·85 (1·44–8·27)	-5·9 (-69·6–229)	5 (1·85–10·6)	5·09 (2·01–10·6)	1·9 (-65·2–235)	9·09 (3·28–19·6)	8·94 (3·45–18·8)	-1·6 (-67·2–233)
Czechia	9·17 (3·2–20·1)	16·4 (6·15–35·3)	79·3 (-42–529)	11·9 (4·4–25·2)	26 (10·3–53·7)	119 (-24·8–627)	21 (7·61–45·3)	42·4 (16·5–89)	102 (-32·4–586)
Hungary	7·42 (2·59–16·3)	7·84 (2·93–16·9)	5·7 (-65·9–270)	8·58 (3·18–18·2)	9·27 (3·66–19·3)	8 (-63·1–255)	16 (5·76–34·5)	17·1 (6·58–36·1)	6·9 (-64·4–262)
Montenegro	0·747 (0·261–1·64)	0·992 (0·371–2·13)	32·8 (-57·1–364)	1·34 (0·499–2·83)	1·82 (0·722–3·76)	35·7 (-53·3–345)	2·09 (0·76–4·47)	2·81 (1·09–5·89)	34·7 (-54·7–351)
North Macedonia	2·08 (0·728–4·57)	2·54 (0·949–5·45)	21·8 (-60·6–326)	3·27 (1·21–6·92)	4·14 (1·64–8·59)	26·6 (-56·6–316)	5·36 (1·94–11·5)	6·68 (2·59–14)	24·7 (-58·2–319)
Poland	32·3 (11·3–70·9)	62·9 (23·5–135)	94·6 (-37·1–579)	41·5 (15·4–87·7)	82·8 (32·7–172)	99·5 (-31·8–553)	73·8 (26·7–159)	146 (56·3–307)	97·3 (-34·1–564)
Romania	15·5 (5·43–34·1)	15·6 (5·83–33·6)	0·5 (-67·6–252)	27·1 (10·1–57·5)	34·2 (13·5–70·8)	25·9 (-56·9–314)	42·7 (15·5–91·7)	49·8 (19·3–104)	16·6 (-60·8–293)
Serbia	10·2 (3·55–22·3)	12·7 (4·75–27·3)	25 (-59·6–337)	16·9 (6·27–35·8)	22·8 (9·01–47·2)	34·7 (-53·8–342)	27·1 (9·83–58)	35·5 (13·8–74·4)	31·1 (-56–340)
Slovakia	3·04 (1·06–6·69)	3·84 (1·43–8·25)	26 (-59·3–341)	3·71 (1·37–7·88)	5·04 (1·99–10·5)	35·7 (-53·7–346)	6·76 (2·44–14·6)	8·88 (3·42–18·7)	31·3 (-56·2–344)
Slovenia	1·52 (0·529–3·33)	2·82 (1·06–6·06)	86·2 (-39·8–552)	1·95 (0·722–4·14)	5·02 (1·98–10·4)	157 (-12–747)	3·47 (1·25–7·47)	7·84 (3·04–16·5)	126 (-24·2–666)
Eastern Europe	254 (88·9–557)	243 (90·5–520)	-4·3 (-69·2–233)	288 (107–609)	271 (107–560)	-6 (-67·9–209)	542 (196–1170)	514 (197–1080)	-5·2 (-68·5–220)
Belarus	9·01 (3·15–19·8)	7·96 (2·97–17·1)	-11·7 (-71·5–209)	13 (4·84–27·6)	13 (5·14–27)	-0·2 (-65·8–228)	22 (7·98–47·4)	21 (8·11–44·1)	-4·9 (-68·2–221)
Estonia	1·15 (0·401–2·52)	2·11 (0·788–4·52)	83·3 (-40·7–543)	1·13 (0·417–2·39)	3·92 (1·56–8·08)	248 (19·7–1060)	2·27 (0·818–4·91)	6·03 (2·35–12·6)	165 (-11·3–808)
Latvia	1·9 (0·664–4·18)	1·62 (0·605–3·48)	-14·9 (-72·5–198)	1·75 (0·649–3·72)	1·67 (0·659–3·47)	-4·8 (-67·5–214)	3·66 (1·31–7·9)	3·29 (1·26–6·95)	-10·1 (-70·1–206)
Lithuania	2·66 (0·93–5·85)	2·55 (0·953–5·48)	-4·2 (-69·1–235)	2·77 (1·02–5·87)	2·88 (1·14–5·98)	4·2 (-64·4–243)	5·43 (1·95–11·7)	5·43 (2·09–11·5)	0·1 (-66·7–239)

Return to Table of Contents

	Female			Male			Both		
	Number of users (thousands)		Change (%)	Number of users (thousands)		Change (%)	Number of users (thousands)		Change (%)
	1990	2023		1990	2023		1990	2023	
Republic of Moldova	4·26 (1·49–9·33)	3·58 (1·34–7·7)	-15·8 (-72·8–195)	5·15 (1·91–10·9)	5·1 (2·02–10·6)	-0·9 (-66·1–226)	9·41 (3·4–20·2)	8·69 (3·36–18·3)	-7·6 (-69·1–212)
Russian Federation	190 (66·3–415)	189 (70·7–406)	-0·3 (-67·8–249)	199 (74·1–421)	193 (76·3–400)	-3·3 (-66·9–218)	389 (140–837)	382 (147–806)	-1·8 (-67·3–233)
Ukraine	45·5 (15·9–100)	36·4 (13·6–78·3)	-20 (-74·2–180)	64·4 (23·9–136)	51·1 (20·2–106)	-20·7 (-72·9–161)	110 (39·8–236)	87·5 (33·8–184)	-20·4 (-73·4–168)
**High-income**	896 (316–1950)	1630 (612–3490)	82 (-40·8–527)	5640 (3480–8570)	7540 (4710–11800)	33·8 (-31·6–188)	6530 (3830–10500)	9170 (5530–15100)	40·4 (-32–217)
Australasia	19·9 (6·96–43·7)	39·3 (14·7–84·6)	97·1 (-36·2–591)	53·7 (19·9–113)	89·8 (35·5–186)	67·4 (-42·6–448)	73·6 (26·9–157)	129 (50·2–270)	75·5 (-40·9–484)
Australia	15·1 (5·28–33·2)	31 (11·6–66·7)	105 (-33·8–618)	46·9 (17·4–99)	78·3 (31–162)	67 (-42·7–447)	62 (22·7–132)	109 (42·6–229)	76·3 (-40·5–486)
New Zealand	4·81 (1·68–10·5)	8·26 (3·09–17·7)	71·9 (-44·4–501)	6·79 (2·52–14·4)	11·6 (4·58–24)	70·5 (-41·6–459)	11·6 (4·2–24·9)	19·8 (7·67–41·7)	71·1 (-42·8–476)
High-income Asia Pacific	218 (76·2–478)	396 (148–849)	81·6 (-41·2–536)	499 (185–1050)	680 (270–1410)	36·3 (-53·2–347)	717 (262–1530)	1080 (418–2260)	50·1 (-49·5–401)
Brunei Darussalam	0·66 (0·232–1·44)	1·68 (0·633–3·59)	155 (-16·9–789)	0·843 (0·314–1·78)	2·03 (0·808–4·2)	141 (-16·8–690)	1·5 (0·546–3·21)	3·72 (1·44–7·78)	147 (-16·9–732)
Japan	165 (57·6–362)	278 (104–595)	68·3 (-45·5–489)	389 (145–822)	477 (189–986)	22·5 (-57·9–301)	554 (202–1180)	755 (293–1580)	36·1 (-54·1–354)
Republic of Korea	48·5 (16·9–106)	104 (38·8–222)	114 (-30·8–649)	99·2 (36·8–210)	178 (70·5–368)	79·6 (-38·4–489)	148 (53·8–316)	282 (109–591)	90·8 (-35·9–539)
Singapore	3·81 (1·33–8·36)	12·7 (4·75–27·2)	232 (7·6–1060)	9·5 (3·53–20·1)	23·1 (9·14–47·7)	143 (-16·5–696)	13·3 (4·86–28·4)	35·7 (13·9–74·8)	168 (-9·5–795)
High-income North America	404 (144–869)	803 (303–1710)	98·9 (-34·5–575)	4730 (3000–6870)	6170 (3840–9540)	30·4 (-30·5–153)	5130 (3230–7690)	6970 (4360–10800)	35·8 (-29·8–180)
Canada	51·9 (18·3–113)	108 (40·3–230)	107 (-32·6–617)	278 (108–560)	265 (105–548)	-4·6 (-65·6–189)	330 (126–673)	373 (146–779)	13 (-60·3–250)
Greenland	0·0608 (0·0213–0·133)	0·086 (0·0322–0·185)	41·4 (-54·3–395)	0·876 (0·334–1·8)	0·865 (0·351–1·75)	-1·2 (-64·5–218)	0·937 (0·356–1·93)	0·951 (0·383–1·93)	1·5 (-63·8–228)
United States of America	352 (126–756)	696 (262–1480)	97·7 (-34·8–569)	4450 (2870–6440)	5900 (3670–9220)	32·6 (-28·4–152)	4800 (3040–7000)	6590 (4090–10200)	37·3 (-28·3–173)

	Female			Male			Both		
	Number of users (thousands)		Change (%)	Number of users (thousands)		Change (%)	Number of users (thousands)		Change (%)
	1990	2023		1990	2023		1990	2023	
Southern Latin America	38·9 (13·6–85·5)	57·8 (21·6–124)	48·5 (-52·1–419)	34·8 (12·9–73·7)	48·1 (18·9–99·9)	38·2 (-52·8–354)	73·7 (26·5–159)	106 (40·5–224)	43·6 (-52·4–387)
Argentina	26·2 (9·14–57·4)	39 (14·6–83·8)	48·9 (-51·9–421)	18·4 (6·81–39·1)	28·1 (11·1–58·4)	52·5 (-48–401)	44·6 (16–96·5)	67 (25·6–142)	50·4 (-50·3–412)
Chile	10·4 (3·62–22·8)	16·1 (6·02–34·7)	55·6 (-49·8–444)	14·3 (5·29–30·3)	17·7 (6·99–36·9)	24·1 (-57·6–307)	24·6 (8·91–53)	33·9 (13–71·5)	37·3 (-54·3–362)
Uruguay	2·4 (0·839–5·27)	2·71 (1·01–5·84)	13 (-63·6–296)	2·1 (0·775–4·45)	2·27 (0·896–4·73)	8·5 (-63–257)	4·5 (1·61–9·72)	4·99 (1·91–10·6)	10·9 (-63·3–277)
Western Europe	214 (74·9–471)	333 (124–716)	55·2 (-49·9–444)	319 (119–674)	559 (223–1160)	75·3 (-39·6–478)	533 (194–1140)	892 (347–1870)	67·2 (-43·8–465)
Andorra	0·0297 (0·0104–0·0654)	0·0654 (0·0244–0·141)	120 (-29·1–670)	0·0516 (0·0191–0·11)	0·118 (0·0466–0·246)	129 (-21·7–653)	0·0814 (0·0295–0·175)	0·184 (0·0711–0·387)	126 (-24·4–659)
Austria	4·69 (1·64–10·3)	8·13 (3·04–17·5)	73·4 (-44–507)	5·93 (2·19–12·6)	13·4 (5·27–27·7)	125 (-23–641)	10·6 (3·83–22·9)	21·5 (8·31–45·2)	102 (-32·3–585)
Belgium	5·29 (1·85–11·6)	6·49 (2·42–14)	22·6 (-60·5–329)	5·83 (2·15–12·4)	7·49 (2·95–15·6)	28·5 (-56·2–323)	11·1 (4–24)	14 (5·37–29·5)	25·7 (-58·2–326)
Cyprus	0·381 (0·133–0·838)	0·833 (0·311–1·79)	119 (-29·5–665)	0·471 (0·174–0·999)	1·02 (0·402–2·12)	117 (-26–613)	0·852 (0·307–1·84)	1·85 (0·713–3·92)	118 (-27·6–635)
Denmark	3·57 (1·25–7·84)	6·7 (2·5–14·4)	87·5 (-39·4–557)	5·37 (1·99–11·4)	11·5 (4·55–23·9)	114 (-26·7–604)	8·95 (3·24–19·2)	18·2 (7·05–38·3)	104 (-31·8–586)
Finland	2·71 (0·946–5·95)	4·31 (1·61–9·26)	58·9 (-48·7–457)	4·9 (1·82–10·4)	13·3 (5·28–27·5)	172 (-6·8–797)	7·61 (2·76–16·3)	17·6 (6·89–36·8)	131 (-21·9–682)
France	30·5 (10·6–67)	44·6 (16·7–96)	46·4 (-52·8–413)	36·6 (13·5–77·7)	56·5 (22·3–117)	54·2 (-47·3–407)	67·1 (24·2–145)	101 (38·9–213)	50·7 (-49·8–410)
Germany	45·9 (16–101)	80·8 (30·2–174)	76·1 (-43·2–517)	50·9 (18·8–108)	113 (44·6–235)	122 (-24·2–631)	96·8 (34·8–209)	194 (74·8–409)	100 (-33·2–579)
Greece	5·59 (1·95–12·3)	6·93 (2·59–14·9)	24·1 (-60–334)	6·28 (2·32–13·3)	8·14 (3·21–16·9)	29·6 (-55·8–326)	11·9 (4·27–25·6)	15·1 (5·79–31·8)	27 (-57·8–330)
Iceland	0·201 (0·0702–0·441)	0·459 (0·171–0·985)	128 (-26·4–699)	0·592 (0·22–1·25)	2·3 (0·92–4·73)	289 (34·5–1190)	0·793 (0·291–1·69)	2·76 (1·09–5·71)	248 (18·7–1070)
Ireland	1·68 (0·586–3·69)	3·14 (1·17–6·75)	86·6 (-39·8–553)	2·09 (0·771–4·43)	4·3 (1·7–8·94)	106 (-29·6–578)	3·77 (1·36–8·12)	7·44 (2·87–15·7)	97·4 (-34·2–567)

	Female			Male			Both		
	Number of users (thousands)		Change (%)	Number of users (thousands)		Change (%)	Number of users (thousands)		Change (%)
	1990	2023		1990	2023		1990	2023	
Israel	2·53 (0·882–5·56)	6·34 (2·37–13·6)	151 (-19·2–777)	3·62 (1·34–7·67)	10·3 (4·06–21·4)	185 (-2·8–836)	6·15 (2·22–13·2)	16·6 (6·43–35)	171 (-9·5–813)
Italy	31·4 (10·9–68·9)	40·7 (15·2–87·5)	29·8 (-58·1–355)	37·6 (13·9–79·8)	59·7 (23·6–124)	58·8 (-45·7–423)	69 (24·8–149)	100 (38·7–212)	45·6 (-51·4–393)
Luxembourg	0·203 (0·0709–0·447)	0·362 (0·135–0·779)	78 (-42·6–523)	0·221 (0·0817–0·469)	0·433 (0·171–0·902)	96·1 (-33·1–545)	0·424 (0·153–0·916)	0·795 (0·306–1·68)	87·4 (-37·7–535)
Malta	0·187 (0·0653–0·411)	0·307 (0·115–0·661)	64 (-47·1–474)	0·228 (0·0842–0·483)	0·466 (0·184–0·969)	105 (-30·2–573)	0·415 (0·15–0·895)	0·773 (0·298–1·63)	86·3 (-37·9–530)
Monaco	0·0201 (0·00703–0·0442)	0·0293 (0·0109–0·0629)	45·4 (-53·1–409)	0·0271 (0·01–0·0575)	0·0459 (0·0181–0·0954)	69·5 (-42·1–457)	0·0472 (0·017–0·102)	0·0752 (0·029–0·158)	59·2 (-46·8–438)
Netherlands	7·84 (2·74–17·2)	11·2 (4·18–24·1)	42·9 (-53·9–401)	9·23 (3·41–19·6)	13·6 (5·35–28·2)	47·1 (-49·8–384)	17·1 (6·15–36·8)	24·8 (9·53–52·3)	45·2 (-51·7–391)
Norway	4·49 (1·57–9·85)	10·3 (3·86–22·1)	130 (-25·7–706)	9·52 (3·55–20·1)	32·9 (13·3–67·4)	246 (19·5–1040)	14 (5·12–30)	43·2 (17·1–89·5)	209 (4·6–942)
Portugal	5·4 (1·88–11·9)	7·11 (2·65–15·3)	31·9 (-57·5–362)	5·58 (2·06–11·8)	7·87 (3·1–16·4)	41·1 (-51·8–364)	11 (3·95–23·7)	15 (5·76–31·7)	36·5 (-54·6–363)
San Marino	0·0127 (0·00443–0·0279)	0·0267 (0·00997–0·0575)	110 (-32·2–636)	0·0189 (0·007–0·0402)	0·043 (0·017–0·0894)	127 (-22·4–647)	0·0316 (0·0114–0·0681)	0·0697 (0·0269–0·147)	120 (-26·4–643)
Spain	20·2 (7·03–44·3)	27·8 (10·4–59·7)	37·7 (-55·6–382)	21·6 (7·97–45·8)	30·9 (12·2–64·4)	43·4 (-51·1–372)	41·7 (15–90·1)	58·7 (22·5–124)	40·7 (-53·3–376)
Sweden	7·11 (2·48–15·6)	20·5 (7·67–43·8)	188 (-6·8–911)	67·6 (25·8–139)	111 (45·5–223)	64 (-42·1–427)	74·7 (28·3–155)	131 (53·2–267)	75·8 (-38·6–471)
Switzerland	4·22 (1·47–9·26)	6·8 (2·54–14·6)	61·2 (-48–465)	6·49 (2·4–13·8)	12·4 (4·91–25·8)	91·8 (-34·4–531)	10·7 (3·87–23)	19·2 (7·45–40·5)	79·8 (-39·8–506)
United Kingdom	30·3 (10·6–66·6)	38·8 (14·5–83·5)	27·9 (-58·8–348)	38·2 (14·1–81)	48·5 (19·1–101)	27·1 (-56·6–318)	68·5 (24·7–148)	87·3 (33·6–184)	27·4 (-57·6–330)
**Latin America and Caribbean**	515 (181–1130)	907 (342–1950)	76·1 (-42·8–519)	670 (253–1410)	1220 (495–2540)	82·7 (-36·9–501)	1190 (434–2540)	2130 (837–4490)	79·8 (-39·4–514)
Andean Latin America	57 (20–125)	81·3 (30·4–175)	42·7 (-53·8–398)	66·3 (24·7–140)	108 (42·7–224)	62·7 (-44·1–433)	123 (44·7–265)	189 (73·2–398)	53·4 (-48·7–418)
Bolivia (Plurinational State of)	6·08 (2·13–13·3)	11·2 (4·18–24)	83·9 (-40·6–543)	8·57 (3·18–18·1)	16 (6·33–33·2)	86·9 (-36–514)	14·7 (5·31–31·4)	27·2 (10·5–57·2)	85·7 (-37·9–525)

	Female			Male			Both		
	Number of users (thousands)		Change (%)	Number of users (thousands)		Change (%)	Number of users (thousands)		Change (%)
	1990	2023		1990	2023		1990	2023	
Ecuador	6·62 (2·31–14·5)	12·9 (4·82–27·8)	95 (-37·1–582)	7·51 (2·78–15·9)	13·4 (5·3–27·9)	78·9 (-38·9–488)	14·1 (5·09–30·5)	26·4 (10·1–55·7)	86·5 (-38–530)
Peru	44·3 (15·5–96·7)	57·2 (21·4–123)	29·1 (-58·1–350)	50·3 (18·7–106)	78·4 (31–162)	56 (-46·3–411)	94·5 (34·2–203)	136 (52·5–285)	43·4 (-52–384)
Caribbean	39 (14·1–88·4)	60·6 (23·5–134)	55·3 (-49·8–446)	69 (27·6–149)	116 (49·7–240)	67·6 (-42·3–445)	108 (41·3–237)	176 (73·6–372)	63·1 (-44·9–450)
Antigua and Barbuda	0·101 (0·0353–0·22)	0·141 (0·0528–0·302)	40·1 (-54·6–389)	0·175 (0·0654–0·367)	0·237 (0·0941–0·49)	35·6 (-53·1–343)	0·275 (0·101–0·587)	0·378 (0·147–0·792)	37·3 (-53·7–359)
Bahamas	0·235 (0·082–0·515)	0·464 (0·173–0·998)	97·9 (-36·1–592)	0·647 (0·241–1·36)	1·05 (0·415–2·16)	61·6 (-44·4–430)	0·882 (0·323–1·88)	1·51 (0·588–3·16)	71·3 (-42·1–470)
Barbados	0·271 (0·0947–0·594)	0·377 (0·141–0·81)	39·3 (-55–387)	0·319 (0·118–0·673)	0·402 (0·159–0·832)	26·1 (-56·8–314)	0·589 (0·213–1·27)	0·779 (0·3–1·64)	32·1 (-56–346)
Belize	0·195 (0·0682–0·427)	0·537 (0·201–1·15)	176 (-10·9–864)	0·202 (0·0749–0·428)	0·494 (0·195–1·02)	144 (-16·4–703)	0·397 (0·143–0·855)	1·03 (0·396–2·18)	160 (-13·7–779)
Bermuda	0·065 (0·0227–0·143)	0·0727 (0·0272–0·156)	11·9 (-63·9–291)	0·102 (0·0377–0·215)	0·101 (0·0398–0·209)	-1 (-66·1–225)	0·167 (0·0604–0·358)	0·173 (0·0669–0·365)	4 (-65·3–249)
Cuba	12·6 (4·39–27·5)	13·1 (4·89–28·1)	4·2 (-66·3–264)	13·7 (5·08–29)	12 (4·74–25)	-12·2 (-70–188)	26·3 (9·47–56·5)	25·1 (9·64–53·1)	-4·4 (-68·2–223)
Dominica	0·0426 (0·0149–0·0937)	0·0495 (0·0185–0·106)	16·1 (-62·6–306)	0·0997 (0·037–0·211)	0·106 (0·042–0·221)	6·7 (-63·5–250)	0·142 (0·0519–0·305)	0·156 (0·0605–0·327)	9·5 (-63·2–266)
Dominican Republic	7·4 (2·59–16·2)	13·3 (5–28·6)	80·4 (-41·7–537)	29·2 (11·3–61·9)	60·5 (25·8–118)	107 (-27·7–590)	36·6 (13·7–77)	73·8 (30·7–146)	102 (-31–579)
Grenada	0·135 (0·0474–0·297)	0·176 (0·0657–0·377)	29·7 (-58·1–353)	0·21 (0·0781–0·444)	0·284 (0·112–0·589)	35·3 (-53·6–343)	0·346 (0·125–0·74)	0·46 (0·178–0·966)	33·1 (-55·4–347)
Guyana	0·62 (0·217–1·36)	0·719 (0·269–1·54)	15·9 (-62·5–307)	0·874 (0·324–1·85)	0·962 (0·381–2)	10·1 (-62·3–264)	1·49 (0·541–3·21)	1·68 (0·649–3·54)	12·5 (-62·4–281)
Haiti	7·73 (2·71–16·9)	20·2 (7·61–43·2)	161 (-15·2–826)	9 (3·35–19)	22·7 (8·99–47)	152 (-13·6–731)	16·7 (6·06–35·9)	42·9 (16·6–90·2)	156 (-14·3–773)
Jamaica	2·6 (0·909–5·7)	3·65 (1·36–7·83)	40·2 (-54·7–390)	4·49 (1·67–9·49)	6·33 (2·5–13·1)	40·7 (-51·7–361)	7·09 (2·58–15·2)	9·97 (3·87–20·9)	40·5 (-52·8–371)
Puerto Rico	3·84 (1·34–8·42)	3·76 (1·41–8·08)	-2·1 (-68·4–243)	4·58 (1·7–9·71)	4·36 (1·72–9·05)	-4·9 (-67·4–214)	8·42 (3·04–18·1)	8·12 (3·13–17·1)	-3·6 (-67·9–226)

	Female			Male			Both		
	Number of users (thousands)		Change (%)	Number of users (thousands)		Change (%)	Number of users (thousands)		Change (%)
	1990	2023		1990	2023		1990	2023	
Saint Kitts and Nevis	0·0275 (0·00961–0·0604)	0·0408 (0·0152–0·0876)	48·1 (-52·2–418)	0·0364 (0·0135–0·0771)	0·0502 (0·0198–0·104)	38 (-52·9–353)	0·0639 (0·0231–0·138)	0·091 (0·035–0·192)	42·3 (-52·6–380)
Saint Lucia	0·157 (0·055–0·345)	0·203 (0·0761–0·437)	29·4 (-58·2–352)	0·199 (0·0738–0·421)	0·271 (0·107–0·562)	36·1 (-53·4–347)	0·356 (0·129–0·765)	0·474 (0·183–0·999)	33·1 (-55·5–349)
Saint Vincent and the Grenadines	0·0924 (0·0323–0·203)	0·101 (0·0378–0·217)	9·4 (-64·7–283)	0·26 (0·097–0·548)	0·277 (0·11–0·573)	6·6 (-63·3–250)	0·353 (0·129–0·75)	0·378 (0·148–0·791)	7·3 (-63·7–258)
Suriname	0·51 (0·178–1·12)	0·755 (0·282–1·62)	48·2 (-52·1–418)	1·21 (0·451–2·53)	1·54 (0·611–3·17)	27·6 (-55·8–317)	1·71 (0·629–3·64)	2·29 (0·893–4·79)	33·7 (-54·7–345)
Trinidad and Tobago	2·35 (0·823–5·13)	2·77 (1·04–5·94)	18·2 (-61·7–313)	3·63 (1·36–7·61)	3·95 (1·57–8·14)	8·8 (-62·4–257)	5·97 (2·18–12·7)	6·72 (2·61–14·1)	12·5 (-62·1–278)
United States Virgin Islands	0·075 (0·0262–0·165)	0·0779 (0·0291–0·167)	3·9 (-66·5–264)	0·101 (0·0377–0·215)	0·119 (0·0472–0·247)	17·5 (-59·7–289)	0·176 (0·0638–0·379)	0·197 (0·0763–0·414)	11·7 (-62·6–279)
Central Latin America	219 (76·7–478)	397 (149–851)	81·6 (-41·1–534)	279 (104–588)	513 (203–1060)	83·8 (-36·7–502)	498 (181–1070)	911 (352–1910)	82·9 (-38·7–515)
Colombia	82·8 (29·2–180)	134 (50·6–287)	62·4 (-47–467)	77·5 (28·8–163)	136 (54·1–282)	76·2 (-39·4–478)	160 (58–344)	271 (105–569)	69·1 (-43·4–472)
Costa Rica	1·96 (0·685–4·3)	3·99 (1·49–8·57)	103 (-34·4–611)	2·38 (0·879–5·04)	4·88 (1·92–10·1)	105 (-29·9–575)	4·34 (1·56–9·34)	8·87 (3·41–18·7)	104 (-31·9–591)
El Salvador	9·6 (3·37–21)	11·9 (4·47–25·6)	24·5 (-59·6–334)	9·81 (3·64–20·7)	12 (4·76–24·9)	22·8 (-57·9–302)	19·4 (7·01–41·7)	24 (9·24–50·6)	23·6 (-58·8–318)
Guatemala	6·43 (2·25–14·1)	16·7 (6·22–35·8)	159 (-16·4–806)	6·85 (2·54–14·5)	17·2 (6·77–35·6)	151 (-14·3–724)	13·3 (4·78–28·6)	33·8 (13–71·4)	155 (-15·3–762)
Honduras	3·55 (1·24–7·79)	10·3 (3·83–22)	189 (-6·7–911)	4·83 (1·79–10·2)	14 (5·53–29)	190 (-0·8–855)	8·38 (3·03–18)	24·2 (9·36–51)	189 (-3·4–878)
Mexico	64·5 (22·5–142)	125 (46·7–269)	93·7 (-37·5–578)	74 (27·4–157)	152 (59·9–316)	105 (-29·8–575)	139 (49·9–298)	277 (107–584)	100 (-33·4–576)
Nicaragua	6·39 (2·24–14)	12·9 (4·84–27·7)	102 (-34·4–606)	7·1 (2·64–15)	15·5 (6·15–32·2)	119 (-24·8–617)	13·5 (4·88–29)	28·5 (11–59·8)	111 (-29·5–612)
Panama	4·11 (1·44–8·99)	8 (3–17·1)	94·6 (-37–581)	4·14 (1·54–8·76)	7·71 (3·05–16)	86·1 (-36·3–511)	8·25 (2·98–17·8)	15·7 (6·05–33·1)	90·3 (-36·6–545)
Venezuela (Bolivarian Republic of)	39·5 (13·9–86·3)	74·3 (27·9–159)	88·2 (-38·9–557)	92·6 (34·8–193)	153 (61·3–314)	65·7 (-42·2–441)	132 (48·6–280)	228 (89·2–473)	72·4 (-41·2–474)

	Female			Male			Both		
	Number of users (thousands)		Change (%)	Number of users (thousands)		Change (%)	Number of users (thousands)		Change (%)
	1990	2023		1990	2023		1990	2023	
Tropical Latin America	200 (70·2–438)	368 (139–793)	83·8 (-40–554)	256 (95·8–537)	488 (199–1010)	90·8 (-33·8–533)	456 (167–976)	855 (338–1810)	87·8 (-36·6–541)
Brazil	186 (65·4–408)	340 (128–730)	82·8 (-40·8–547)	234 (87·5–492)	446 (181–917)	90·7 (-34·6–528)	420 (154–901)	787 (308–1650)	87·2 (-37·4–533)
Paraguay	13·7 (4·81–29·7)	27 (10·2–57·6)	98 (-35·3–590)	21·7 (8·14–45·3)	41·6 (16·6–85·4)	92 (-33–527)	35·3 (12·9–75)	68·7 (26·8–143)	94·4 (-33·9–550)
North Africa and Middle East	575 (222–1210)	1170 (506–2360)	104 (-25·9–570)	1520 (639–3050)	3380 (1700–6240)	122 (-10·4–548)	2100 (858–4240)	4550 (2200–8640)	117 (-14·7–553)
Afghanistan	10·1 (3·53–22·1)	28·9 (10·8–62)	186 (-7·5–903)	60·4 (23·1–126)	297 (134–565)	393 (74·3–1520)	70·5 (26·5–146)	326 (144–627)	363 (61·8–1450)
Algeria	34·4 (12·1–75·3)	78·6 (29·5–168)	128 (-26–701)	199 (77·3–411)	424 (180–841)	113 (-24·6–589)	234 (89–486)	503 (211–1010)	115 (-25·2–598)
Bahrain	0·711 (0·249–1·55)	2·04 (0·763–4·37)	187 (-7·1–901)	2·32 (0·866–4·89)	8·04 (3·19–16·6)	246 (19·5–1030)	3·03 (1·11–6·44)	10·1 (3·95–21)	232 (13–1000)
Egypt	38·4 (13·4–84·4)	79·4 (29·7–171)	106 (-33·4–623)	103 (38·3–218)	214 (84·6–443)	107 (-28·9–581)	142 (51·8–302)	293 (114–614)	107 (-30·1–592)
Iran (Islamic Republic of)	75·2 (26·3–165)	134 (50–287)	77·6 (-42·6–520)	166 (61·8–350)	302 (120–625)	82·1 (-37·2–495)	241 (88·2–514)	436 (170–912)	80·7 (-38·9–503)
Iraq	17·1 (5·98–37·5)	44·7 (16·7–95·9)	161 (-15·5–815)	33·4 (12·4–70·6)	84·6 (33·5–175)	153 (-13·2–731)	50·5 (18·4–108)	129 (50·2–271)	156 (-14–758)
Jordan	3·28 (1·15–7·18)	10·2 (3·81–21·9)	211 (0·4–986)	6·95 (2·59–14·7)	20·2 (7·98–41·8)	190 (-0·5–852)	10·2 (3·73–21·9)	30·4 (11·8–63·7)	197 (-0·2–893)
Kuwait	1·42 (0·495–3·1)	3·98 (1·49–8·54)	181 (-9·3–883)	3·6 (1·34–7·62)	13·1 (5·22–27·2)	265 (25·2–1110)	5·02 (1·83–10·7)	17·1 (6·71–35·8)	241 (15·4–1050)
Lebanon	6·33 (2·22–13·8)	12 (4·5–25·7)	89·5 (-38·6–562)	8·39 (3·13–17·7)	16·8 (6·66–34·7)	100 (-31·2–558)	14·7 (5·35–31·5)	28·8 (11·2–60·4)	95·5 (-34·4–560)
Libya	4·41 (1·54–9·64)	8·68 (3·24–18·6)	96·9 (-36·3–588)	11·1 (4·13–23·4)	21·8 (8·62–45)	95·9 (-32·6–541)	15·5 (5·68–33·1)	30·4 (11·9–63·6)	96·2 (-33·7–554)
Morocco	25·2 (8·82–55·3)	39·4 (14·7–84·6)	56·3 (-49·5–447)	66·6 (25·4–139)	111 (47·1–222)	67·4 (-40·6–448)	91·8 (33·9–194)	151 (62–306)	64·3 (-43·4–443)
Oman	2·38 (0·835–5·21)	7·59 (2·84–16·3)	219 (3·2–1010)	6·8 (2·53–14·3)	19·6 (7·76–40·5)	188 (-0·8–843)	9·18 (3·37–19·5)	27·2 (10·6–56·8)	196 (0·3–884)

	Female			Male			Both		
	Number of users (thousands)		Change (%)	Number of users (thousands)		Change (%)	Number of users (thousands)		Change (%)
	1990	2023		1990	2023		1990	2023	
Palestine	2·28 (0·799–5)	5·89 (2·21–12·6)	158 (-16·5–803)	6·81 (2·54–14·3)	20·3 (8·09–41·9)	199 (3·6–877)	9·09 (3·34–19·3)	26·2 (10·3–54·6)	189 (-1·7–859)
Qatar	0·28 (0·0982–0·614)	1·92 (0·719–4·13)	586 (122–2300)	2·5 (0·946–5·23)	16·1 (6·43–33)	542 (123–2000)	2·78 (1·04–5·84)	18 (7·15–37·2)	547 (123–2030)
Saudi Arabia	16·7 (5·85–36·6)	43·5 (16·3–93·3)	160 (-15·8–810)	41·3 (15·4–87·1)	112 (44·3–232)	171 (-6·9–787)	58 (21·2–124)	155 (60·6–325)	168 (-9·6–793)
Sudan	15·7 (5·5–34·5)	31·5 (11·8–67·8)	100 (-35·3–601)	151 (57·4–310)	409 (183–778)	171 (-2·9–785)	167 (63·2–343)	441 (194–847)	165 (-6·5–770)
Syrian Arab Republic	10·1 (3·53–22·1)	25·8 (9·66–55·2)	155 (-17·3–795)	10·4 (3·84–22)	28·4 (11·2–58·9)	174 (-6·1–802)	20·5 (7·38–44·1)	54·2 (20·9–114)	165 (-11·7–799)
Tunisia	22·2 (7·84–48)	30·6 (11·5–65·3)	38·1 (-54·6–377)	47 (17·8–97·5)	63·5 (25·4–130)	35 (-52·4–335)	69·2 (25·6–146)	94·1 (37–195)	36 (-53·2–348)
Türkiye	108 (38–237)	204 (76·3–436)	88 (-39·1–557)	139 (51·7–294)	227 (89·6–469)	62·7 (-44·1–433)	248 (89·7–531)	430 (166–905)	73·8 (-41·9–485)
United Arab Emirates	1·04 (0·362–2·27)	4·87 (1·82–10·5)	369 (51·5–1540)	3·84 (1·42–8·14)	21 (8·3–43·7)	447 (87–1700)	4·88 (1·79–10·4)	25·9 (10·1–54·2)	431 (79·4–1660)
Yemen	178 (77·3–344)	377 (202–681)	111 (-10–502)	454 (231–781)	947 (615–1480)	109 (8·1–381)	632 (313–1120)	1320 (828–2160)	109 (2·9–421)
South Asia	35900 (17800–62300)	60800 (35000–98600)	69·2 (-25–304)	87200 (48700–135000)	131000 (99300–175000)	50·7 (-12·8–184)	123000 (66500–198000)	192000 (135000–273000)	56·1 (-16·4–209)
Bangladesh	5960 (3130–9720)	14400 (9530–20600)	143 (27·1–393)	5760 (2970–9540)	12200 (9960–15100)	113 (22·5–315)	11700 (6000–19300)	26700 (20800–34600)	128 (29·1–347)
Bhutan	13·4 (5·48–26·4)	24·2 (13·4–39·7)	80·8 (-23·4–414)	39·1 (18·9–67·6)	61·7 (38·7–91·9)	57·8 (-22·9–252)	52·5 (24·6–94·2)	85·9 (53·5–132)	63·7 (-22·6–280)
India	28500 (14000–49800)	42900 (23400–72600)	50·4 (-36·3–283)	75100 (42600–115000)	105000 (79400–139000)	40·5 (-18·3–159)	104000 (56700–165000)	148000 (104000–211000)	43·2 (-22·8–183)
Nepal	304 (131–596)	582 (311–996)	91·2 (-17·9–423)	1830 (1050–2730)	3190 (2320–4200)	73·8 (5·4–211)	2140 (1190–3300)	3770 (2690–5150)	76·2 (3·2–227)
Pakistan	1120 (417–2300)	2770 (1170–5550)	146 (-13–734)	4490 (2080–8190)	10400 (5840–17700)	131 (-0·4–532)	5610 (2530–10600)	13100 (7090–22600)	134 (-4·2–560)
**Southeast Asia, East Asia, and Oceania**	5250 (2040–10900)	9710 (4400–19200)	84·9 (-29·9–477)	8210 (3370–16200)	14500 (6390–27700)	76·3 (-34·4–429)	13500 (5460–27000)	24200 (10900–46900)	79·6 (-31·8–442)

	Female			Male			Both		
	Number of users (thousands)		Change (%)	Number of users (thousands)		Change (%)	Number of users (thousands)		Change (%)
	1990	2023		1990	2023		1990	2023	
East Asia	1790 (626–3920)	3250 (1220–6960)	81·5 (-41·2–536)	4010 (1500–8460)	6670 (2650–13700)	66·3 (-42·8–446)	5800 (2120–12400)	9920 (3870–20700)	71 (-42·3–472)
China	1740 (609–3810)	3180 (1190–6800)	82·5 (-40·9–539)	3940 (1470–8310)	6580 (2620–13600)	66·9 (-42·5–449)	5680 (2080–12100)	9760 (3810–20400)	71·7 (-42–475)
Democratic People's Republic of Korea	15·4 (5·37–33·8)	22 (8·22–47·3)	43·2 (-53·8–401)	14·4 (5·33–30·6)	20·6 (8·13–42·9)	43 (-51·2–370)	29·8 (10·7–64·3)	42·6 (16·3–90·2)	43·1 (-52·5–386)
Taiwan	33·4 (11·7–73·2)	49·5 (18·5–106)	47·9 (-52·1–417)	57·3 (21·3–121)	73·4 (29·1–152)	28·1 (-56–320)	90·7 (33–194)	123 (47·6–258)	35·4 (-54·5–354)
Oceania	147 (64·2–309)	504 (280–881)	242 (33–872)	130 (57·6–265)	301 (143–585)	132 (-11·2–553)	277 (123–580)	805 (434–1470)	190 (14·8–711)
American Samoa	0·371 (0·133–0·794)	0·391 (0·149–0·823)	5·5 (-64·6–263)	0·723 (0·279–1·46)	0·738 (0·302–1·47)	2·1 (-62·6–224)	1·09 (0·412–2·26)	1·13 (0·451–2·3)	3·2 (-63·2–237)
Cook Islands	0·152 (0·0552–0·325)	0·117 (0·0448–0·245)	-23·3 (-74·3–162)	0·247 (0·095–0·505)	0·18 (0·0734–0·361)	-27·3 (-73·7–132)	0·4 (0·15–0·83)	0·297 (0·118–0·607)	-25·8 (-73·9–143)
Fiji	4·65 (1·65–10)	6·16 (2·34–13)	32·5 (-56·1–359)	5·8 (2·19–12)	7·78 (3·15–15·8)	34·2 (-52·6–336)	10·4 (3·84–22·1)	13·9 (5·48–28·8)	33·4 (-54·2–347)
Guam	1·27 (0·497–2·7)	1·97 (0·983–3·49)	54·5 (-42·8–389)	4·49 (1·99–8·47)	5·27 (3·17–8·39)	17·4 (-46·8–194)	5·76 (2·52–11·2)	7·24 (4·26–11·8)	25·6 (-45·4–232)
Kiribati	0·952 (0·368–1·98)	2·97 (1·51–5·19)	212 (17·5–875)	2·5 (1·1–4·66)	6·13 (3·35–9·95)	145 (7–555)	3·45 (1·47–6·63)	9·1 (4·87–15·2)	164 (9·7–635)
Marshall Islands	0·606 (0·248–1·23)	1·13 (0·621–1·9)	86·5 (-24·4–438)	1·58 (0·767–2·77)	2·05 (1·18–3·21)	29·5 (-41·4–219)	2·19 (1·02–4)	3·18 (1·8–5·1)	45·3 (-35·9–272)
Micronesia (Federated States of)	1·53 (0·598–3·08)	1·67 (0·71–3·23)	8·8 (-60·5–243)	2·6 (1·09–4·87)	2·89 (1·33–5·32)	11·4 (-55·5–213)	4·13 (1·69–7·94)	4·56 (2·04–8·55)	10·4 (-57·6–224)
Nauru	0·0192 (0·00676–0·042)	0·0263 (0·00988–0·0561)	36·6 (-55·6–381)	0·0238 (0·00889–0·0501)	0·0319 (0·0128–0·0658)	34·2 (-53·7–344)	0·043 (0·0156–0·0921)	0·0582 (0·0226–0·122)	35·3 (-54·5–362)
Niue	0·0101 (0·00358–0·0219)	0·00806 (0·00305–0·0171)	-20·2 (-73·8–179)	0·012 (0·00452–0·0248)	0·00979 (0·00411–0·0195)	-18·1 (-70·8–161)	0·0221 (0·00815–0·0466)	0·0178 (0·00717–0·0366)	-19·1 (-72·2–169)
Northern Mariana Islands	0·753 (0·289–1·53)	0·673 (0·287–1·32)	-10·6 (-68·1–186)	1·36 (0·566–2·61)	1·23 (0·561–2·29)	-9·5 (-64·9–167)	2·11 (0·863–4·14)	1·9 (0·849–3·61)	-9·9 (-66–174)
Palau	0·98 (0·431–1·79)	1·68 (0·912–2·71)	71·7 (-23·8–358)	1·09 (0·498–1·94)	1·91 (1·08–3·02)	75·7 (-20·3–344)	2·07 (0·929–3·72)	3·6 (1·99–5·73)	73·8 (-22–353)

	Female			Male			Both		
	Number of users (thousands)		Change (%)	Number of users (thousands)		Change (%)	Number of users (thousands)		Change (%)
	1990	2023		1990	2023		1990	2023	
Papua New Guinea	132 (54·2–256)	476 (256–792)	261 (45·2–941)	103 (42·7–194)	255 (115–477)	149 (-3·5–612)	234 (97·6–456)	731 (381–1270)	212 (27–792)
Samoa	0·443 (0·156–0·965)	0·659 (0·249–1·4)	48·7 (-51·5–425)	0·753 (0·284–1·58)	0·995 (0·4–2·04)	32·1 (-54·1–332)	1·2 (0·44–2·54)	1·65 (0·65–3·44)	38·2 (-53·1–366)
Solomon Islands	2·72 (0·987–5·8)	8·06 (3·14–16·8)	196 (-0·5–912)	3·89 (1·5–7·94)	11·3 (4·7–22·6)	191 (4·7–827)	6·62 (2·49–13·7)	19·4 (7·85–39·4)	193 (2·5–861)
Tokelau	0·00875 (0·0031–0·0189)	0·0105 (0·00398–0·0222)	20 (-60·3–317)	0·0103 (0·0039–0·0214)	0·0142 (0·00574–0·0289)	37·9 (-51·1–347)	0·0191 (0·00701–0·0403)	0·0247 (0·00973–0·0511)	29·7 (-55·4–334)
Tonga	0·155 (0·0544–0·34)	0·166 (0·0623–0·357)	7·1 (-65·3–274)	0·227 (0·0843–0·478)	0·21 (0·0833–0·436)	-7·1 (-68·1–204)	0·382 (0·139–0·818)	0·377 (0·146–0·793)	-1·3 (-66·9–231)
Tuvalu	0·0208 (0·0073–0·0455)	0·0213 (0·008–0·0456)	2·4 (-66·8–258)	0·0248 (0·00925–0·0523)	0·0302 (0·012–0·0624)	21·5 (-58·2–298)	0·0457 (0·0166–0·0978)	0·0515 (0·02–0·108)	12·8 (-62·2–280)
Vanuatu	1·12 (0·402–2·39)	2·75 (1·06–5·75)	145 (-17·4–743)	1·99 (0·771–4·04)	4·69 (1·93–9·34)	136 (-13·6–640)	3·11 (1·17–6·43)	7·44 (2·99–15·1)	139 (-15–676)
Southeast Asia	3330 (1350–6690)	5980 (2910–11400)	79·7 (-27·5–441)	4070 (1800–7660)	7500 (3590–13200)	84·3 (-25·3–399)	7400 (3150–14200)	13500 (6570–24600)	82·2 (-27·5–428)
Cambodia	289 (149–485)	519 (307–830)	79·6 (-8·7–308)	47·3 (18·2–97·1)	104 (43·9–200)	119 (-24·4–617)	336 (171–573)	622 (433–881)	85·2 (3·4–280)
Indonesia	889 (332–1810)	1120 (430–2330)	25·6 (-56·8–304)	182 (67·6–386)	539 (213–1120)	195 (1·2–872)	1070 (400–2200)	1650 (643–3440)	54·5 (-46·6–399)
Lao People's Democratic Republic	59·8 (23·9–122)	146 (71·4–266)	145 (-12·4–659)	6·09 (2·26–12·9)	13·8 (5·45–28·6)	126 (-22·5–644)	65·9 (26·2–135)	160 (77·9–290)	143 (-12·6–663)
Malaysia	69·3 (24·7–150)	204 (78·5–431)	195 (-2·4–921)	107 (40·8–222)	405 (172–805)	280 (32·9–1120)	176 (64·8–371)	610 (251–1230)	246 (19·3–1040)
Maldives	1·3 (0·499–2·74)	4·67 (1·94–9·09)	259 (22·8–1100)	2·61 (1·15–5·07)	13 (6·47–23·3)	400 (88·3–1360)	3·91 (1·64–7·81)	17·7 (8·41–32·2)	353 (65–1270)
Mauritius	9·46 (3·42–20)	15 (5·79–31·2)	58·8 (-45·6–442)	5·58 (2·09–11·7)	8·38 (3·34–17·2)	50 (-47·9–390)	15 (5·51–31·7)	23·4 (9·12–48·4)	55·5 (-46·4–422)
Myanmar	924 (357–1830)	1690 (722–3210)	83·3 (-33·5–472)	2340 (1120–4130)	3810 (1940–6250)	62·7 (-30·3–313)	3270 (1480–5980)	5510 (2690–9440)	68·5 (-30·3–343)
Philippines	202 (72–435)	383 (148–804)	89·4 (-38·3–563)	449 (175–924)	1010 (425–2000)	124 (-21·1–620)	651 (248–1360)	1390 (575–2800)	113 (-27·3–594)

	Female			Male			Both		
	Number of users (thousands)		Change (%)	Number of users (thousands)		Change (%)	Number of users (thousands)		Change (%)
	1990	2023		1990	2023		1990	2023	
Seychelles	0·456 (0·164–0·972)	0·695 (0·266–1·46)	52·6 (-48·6–425)	0·363 (0·136–0·761)	0·668 (0·266–1·37)	83·9 (-36·1–501)	0·819 (0·3–1·73)	1·36 (0·532–2·83)	66·5 (-43·1–460)
Sri Lanka	241 (90·7–495)	440 (179–874)	82·2 (-37·2–499)	664 (289–1260)	1080 (508–1900)	63 (-36·6–363)	905 (375–1730)	1520 (689–2760)	68·1 (-36·3–392)
Thailand	419 (157–872)	1010 (412–2020)	142 (-18·5–714)	180 (67·2–378)	353 (141–724)	95·9 (-32·3–546)	599 (222–1250)	1370 (554–2750)	128 (-22·6–663)
Timor-Leste	3·63 (1·32–7·71)	7·53 (2·93–15·7)	107 (-30·6–608)	2·6 (0·976–5·45)	5·95 (2·38–12·2)	129 (-20·7–657)	6·23 (2·29–13·1)	13·5 (5·31–28)	116 (-26·5–625)
Viet Nam	223 (80·6–475)	437 (169–916)	96·3 (-34·6–574)	77·9 (28·9–165)	152 (60·1–316)	95·3 (-33·1–542)	301 (109–640)	589 (229–1230)	96·1 (-34·2–567)
**Sub-Saharan Africa**	1220 (458–2600)	3010 (1330–5950)	146 (-12·3–724)	1710 (698–3440)	4070 (1920–7590)	138 (-9–614)	2930 (1150–6040)	7070 (3270–13500)	141 (-10·7–653)
Central Sub-Saharan Africa	124 (45·9–265)	234 (90·5–493)	88 (-37·6–559)	132 (49·3–277)	441 (185–893)	234 (17–1000)	256 (95·1–542)	675 (275–1380)	163 (-9·7–783)
Angola	8·47 (2·96–18·6)	32·2 (12–69·1)	280 (22·7–1230)	16·8 (6·26–35·5)	56·4 (22·4–117)	235 (15·4–1010)	25·3 (9·22–54·1)	88·6 (34·5–186)	250 (17·8–1080)
Central African Republic	6·22 (2·2–13·5)	14·4 (5·46–30·5)	131 (-24·4–713)	4·3 (1·6–9·08)	10·5 (4·16–21·6)	144 (-16·2–706)	10·5 (3·79–22·6)	24·8 (9·62–52·1)	136 (-21·1–712)
Congo	5·07 (1·79–11·1)	14·2 (5·36–30·4)	181 (-8·7–886)	2·84 (1·06–6)	9·28 (3·7–19·2)	227 (11·6–991)	7·91 (2·85–17·1)	23·5 (9·06–49·6)	197 (-1·3–928)
Democratic Republic of the Congo	103 (38·2–219)	169 (65·5–350)	64·1 (-46·7–466)	106 (39·9–223)	359 (151–719)	237 (16·9–1000)	209 (78·8–442)	528 (217–1070)	152 (-14·6–733)
Equatorial Guinea	0·733 (0·257–1·6)	2·22 (0·831–4·76)	203 (-1·9–955)	0·612 (0·227–1·29)	3·44 (1·36–7·12)	462 (92·5–1740)	1·35 (0·484–2·9)	5·66 (2·19–11·9)	321 (40·8–1330)
Gabon	1·02 (0·358–2·24)	2·11 (0·789–4·52)	106 (-33·4–623)	1·05 (0·388–2·22)	2·48 (0·979–5·14)	136 (-19·1–678)	2·07 (0·746–4·46)	4·58 (1·77–9·66)	121 (-26·2–652)
Eastern Sub-Saharan Africa	611 (230–1310)	1620 (771–3060)	165 (-0·8–763)	980 (418–1920)	2430 (1210–4370)	148 (-0·3–594)	1590 (645–3230)	4050 (2020–7410)	155 (-0·4–658)
Burundi	31·8 (11·5–67·9)	71·8 (28·9–143)	126 (-26–686)	39·2 (15·2–78·9)	85·2 (36–165)	117 (-23–604)	70·9 (26·6–146)	157 (64·6–308)	121 (-24·6–635)
Comoros	3·77 (1·42–7·82)	6·54 (2·64–13·3)	73·6 (-40·8–474)	4·98 (2·05–9·97)	10 (4·49–18·6)	101 (-26·4–517)	8·75 (3·4–17·6)	16·5 (7·12–31·7)	89·1 (-33–503)

	Female			Male			Both		
	Number of users (thousands)		Change (%)	Number of users (thousands)		Change (%)	Number of users (thousands)		Change (%)
	1990	2023		1990	2023		1990	2023	
Djibouti	1·19 (0·42–2·59)	4·47 (1·68–9·54)	276 (22·9–1210)	1·89 (0·708–3·95)	7·02 (2·8–14·4)	272 (29·6–1120)	3·08 (1·13–6·54)	11·5 (4·49–24)	274 (26·9–1150)
Eritrea	2·74 (0·958–6·01)	6·38 (2·38–13·7)	133 (-24·8–714)	25·4 (9·73–52·1)	55·4 (22·5–112)	118 (-23–605)	28·1 (10·7–58·1)	61·7 (24·9–126)	120 (-23·1–617)
Ethiopia	58·8 (20·6–129)	148 (55·9–320)	152 (-18–789)	232 (89·6–469)	512 (218–1010)	121 (-21·3–617)	291 (111–594)	660 (276–1330)	127 (-20·1–640)
Kenya	58·6 (20·8–127)	136 (51·6–291)	133 (-24·3–721)	64·4 (24·3–135)	182 (73·1–374)	183 (-2·7–827)	123 (45·1–262)	318 (125–664)	159 (-13·2–778)
Madagascar	211 (86·9–425)	730 (397–1220)	245 (42·3–902)	424 (202–750)	1120 (618–1810)	164 (15·9–583)	635 (289–1170)	1850 (1040–3050)	191 (23·8–681)
Malawi	26·9 (9·64–58)	47·8 (18·2–102)	77·8 (-41·8–519)	12·7 (4·73–26·9)	29·7 (11·8–61·6)	133 (-20–670)	39·6 (14·4–84·9)	77·5 (30–163)	95·7 (-34·8–573)
Mozambique	50·5 (17·9–109)	67·5 (25·4–144)	33·6 (-55·9–362)	31·6 (11·8–66·4)	64·9 (25·7–134)	105 (-29–569)	82·2 (29·8–175)	132 (51·2–278)	61·1 (-45·8–445)
Rwanda	37·5 (14–79·1)	73·1 (29·4–150)	95·1 (-35·1–561)	6·48 (2·4–13·7)	11·2 (4·44–23·4)	73·4 (-40·7–470)	43·9 (16·3–92·9)	84·3 (34·1–172)	91·9 (-35·9–542)
Somalia	18·1 (6·38–39·5)	48·9 (18·4–104)	169 (-12·1–838)	23·4 (8·73–49·2)	58·5 (23·2–121)	150 (-13·7–716)	41·6 (15·1–88·8)	107 (41·6–225)	158 (-13–767)
South Sudan	14·8 (5·21–32·3)	29·9 (11·2–63·7)	101 (-34·2–602)	24 (8·96–50·3)	38·8 (15·5–79·8)	61·9 (-43·8–429)	38·8 (14·2–82·6)	68·7 (26·7–144)	77 (-40·1–492)
Uganda	40·2 (14·3–86·9)	104 (40–221)	159 (-15·8–805)	28·3 (10·6–59·6)	89 (35·6–184)	215 (7·5–939)	68·5 (25–146)	193 (75·6–404)	182 (-6·4–860)
United Republic of Tanzania	40·5 (14·2–88·5)	104 (39·1–223)	157 (-16·6–800)	50·9 (19·1–107)	136 (54·3–281)	167 (-8·5–787)	91·5 (33·3–196)	240 (93·4–504)	163 (-12–797)
Zambia	13·5 (4·73–29·4)	40·2 (15·1–85·8)	199 (-2·9–951)	10·3 (3·84–21·9)	31·9 (12·6–66·1)	209 (5·9–918)	23·8 (8·57–51·2)	72·1 (27·8–152)	203 (1–936)
Southern Sub-Saharan Africa	192 (69·9–413)	358 (142–745)	85·8 (-37·2–536)	228 (85·9–472)	275 (110–567)	21 (-57·6–290)	420 (156–885)	633 (252–1320)	50·7 (-48·6–404)
Botswana	17·3 (6·81–35·4)	43·8 (21·2–80)	154 (-6–684)	8·01 (3·03–16·6)	17·5 (7·08–35·7)	119 (-23·2–613)	25·3 (9·88–52·6)	61·3 (28·7–115)	143 (-9·8–660)
Eswatini	0·857 (0·301–1·87)	1·93 (0·729–4·12)	125 (-27–701)	1·21 (0·451–2·54)	2·38 (0·949–4·92)	97·3 (-32·4–557)	2·06 (0·752–4·42)	4·31 (1·68–9·03)	109 (-30·1–615)

	Female			Male			Both		
	Number of users (thousands)		Change (%)	Number of users (thousands)		Change (%)	Number of users (thousands)		Change (%)
	1990	2023		1990	2023		1990	2023	
Lesotho	2·37 (0·835–5·17)	3·29 (1·24–7·01)	38·6 (-54·8–385)	3·72 (1·39–7·78)	5·23 (2·13–10·7)	40·6 (-51·7–366)	6·09 (2·25–13)	8·52 (3·37–17·7)	39·9 (-53·2–371)
Namibia	3·07 (1·09–6·67)	7·87 (2·99–16·7)	156 (-15·9–796)	3·08 (1·15–6·47)	6·91 (2·75–14·3)	125 (-23–648)	6·15 (2·24–13·1)	14·8 (5·74–31)	140 (-19·4–719)
South Africa	151 (53·3–325)	256 (97·2–541)	69·9 (-43·8–490)	198 (74·6–409)	222 (88·1–456)	12·1 (-60·6–260)	348 (128–734)	478 (185–997)	37·1 (-53·1–354)
Zimbabwe	17·8 (6·28–38·9)	44·1 (16·7–93·6)	147 (-19·2–766)	14 (5·21–29·6)	21·9 (8·65–45·3)	55·9 (-46·6–414)	31·9 (11·5–68·5)	66 (25·3–139)	107 (-31·1–604)
Western Sub-Saharan Africa	295 (105–635)	795 (308–1680)	169 (-12·3–843)	367 (140–763)	921 (380–1860)	151 (-12·7–720)	663 (245–1400)	1720 (687–3540)	159 (-12–774)
Benin	11·3 (4·03–24·6)	35·6 (14–74)	213 (2·7–994)	14·7 (5·51–30·7)	58·3 (24·6–115)	296 (35·9–1230)	26·1 (9·75–55)	93·8 (38·9–190)	260 (21·6–1130)
Burkina Faso	48 (17·9–102)	104 (42·4–207)	117 (-27·4–642)	29·3 (11·3–60·1)	62·7 (26–125)	114 (-25·7–587)	77·3 (28·8–162)	167 (68·4–332)	116 (-27·1–617)
Cabo Verde	0·56 (0·196–1·22)	0·739 (0·277–1·58)	32 (-57·3–362)	0·312 (0·116–0·661)	0·584 (0·231–1·21)	87·3 (-36–515)	0·871 (0·312–1·88)	1·32 (0·507–2·8)	51·8 (-49·7–419)
Cameroon	24·3 (8·91–51·9)	78·7 (31·3–163)	224 (6·5–1000)	14·6 (5·45–30·7)	51·3 (20·5–106)	252 (20·8–1060)	38·8 (14·2–82·6)	130 (51·8–268)	235 (13–1030)
Chad	13·8 (4·88–29·7)	38 (14·7–80·3)	177 (-9·4–864)	11·5 (4·31–24)	33·2 (13·5–68)	190 (0·4–846)	25·2 (9·19–53·6)	71·3 (28·2–148)	183 (-5–856)
Côte d'Ivoire	11·9 (4·17–26·1)	32 (12–68·6)	169 (-13·1–839)	12·9 (4·79–27·4)	41·4 (16·4–86)	221 (9·7–955)	24·8 (8·96–53·4)	73·4 (28·3–155)	196 (-1·3–902)
Gambia	1·15 (0·404–2·51)	3·24 (1·22–6·93)	182 (-8·4–886)	1·53 (0·571–3·21)	3·85 (1·55–7·9)	152 (-14·3–735)	2·68 (0·98–5·73)	7·09 (2·77–14·9)	165 (-11·4–799)
Ghana	17·8 (6·24–38·9)	52·2 (19·6–112)	193 (-4·9–931)	24·4 (9·17–51)	63·8 (25·7–132)	162 (-10·4–762)	42·1 (15·5–90)	116 (45·2–243)	175 (-8·1–836)
Guinea	7·3 (2·56–16)	14·6 (5·48–31·4)	101 (-35–600)	11·9 (4·41–25)	27·5 (10·9–56·8)	131 (-20·4–658)	19·2 (6·97–41)	42·1 (16·4–88·2)	120 (-26·1–637)
Guinea-Bissau	1·17 (0·409–2·55)	2·4 (0·897–5·14)	105 (-33·6–617)	1·08 (0·402–2·29)	2·23 (0·879–4·62)	105 (-29·8–574)	2·25 (0·811–4·85)	4·62 (1·78–9·76)	105 (-31·8–595)
Liberia	3·07 (1·08–6·72)	7·08 (2·65–15·2)	130 (-25·4–705)	7·18 (2·72–15)	19·7 (7·93–40·3)	174 (-6·2–801)	10·2 (3·81–21·7)	26·7 (10·6–55·6)	161 (-12·2–778)

	Female			Male			Both		
	Number of users (thousands)		Change (%)	Number of users (thousands)		Change (%)	Number of users (thousands)		Change (%)
	1990	2023		1990	2023		1990	2023	
Mali	10·6 (3·71–23·1)	25·2 (9·44–54)	138 (-22·9–731)	36·5 (14–75)	96·7 (40·6–190)	165 (-7–760)	47·1 (17·9–97·2)	122 (50·8–244)	159 (-8·7–748)
Mauritania	4·84 (1·7–10·5)	12·3 (4·62–26·1)	153 (-17·4–785)	5·78 (2·15–12·2)	14·6 (5·81–30·1)	153 (-12·8–728)	10·6 (3·86–22·7)	26·9 (10·4–56·2)	153 (-15–753)
Niger	17·9 (6·5–38·7)	65·7 (25·8–137)	266 (19–1160)	38·7 (15·3–80)	120 (51·8–231)	210 (11·6–896)	56·7 (21·5–118)	186 (76·7–369)	228 (13·8–976)
Nigeria	85·2 (29·9–186)	238 (89·3–510)	179 (-9·5–891)	131 (48·8–276)	267 (106–553)	104 (-30·1–579)	216 (78·6–462)	504 (195–1060)	134 (-21·8–696)
Sao Tome and Principe	0·14 (0·049–0·306)	0·309 (0·116–0·662)	121 (-28·4–676)	0·171 (0·0637–0·361)	0·491 (0·196–1·01)	187 (-1·4–855)	0·311 (0·113–0·668)	0·8 (0·312–1·68)	157 (-13·7–778)
Senegal	13·9 (4·88–30·2)	37·9 (14·2–80·8)	173 (-11·2–855)	6·75 (2·51–14·3)	16·2 (6·4–33·6)	140 (-17·8–693)	20·6 (7·38–44·5)	54 (20·6–114)	162 (-13·4–799)
Sierra Leone	14·2 (5·11–30·6)	23·6 (9·25–49·3)	66·2 (-45·3–480)	11·7 (4·42–24·4)	21·5 (8·64–44·1)	84 (-36·7–503)	25·9 (9·53–54·9)	45·1 (17·9–93·5)	74·2 (-41·5–490)
Togo	8·11 (2·87–17·6)	23 (8·72–48·8)	183 (-7·1–896)	7·75 (2·9–16·3)	20·3 (8·14–41·7)	162 (-9·7–764)	15·9 (5·77–33·9)	43·3 (16·9–90·5)	173 (-8·3–831)

Return to Table of Contents

Appendix Table 2·4: All-age prevalence of chewing tobacco use in 1990 and 2023, among ages 15+

	Female			Male			Both		
	All-age prevalence (%)		Change (%)	All-age prevalence (%)		Change (%)	All-age prevalence (%)		Change (%)
	1990	2023		1990	2023		1990	2023	
Global	2·5 (1·2–4·5)	2·6 (1·4–4·3)	3·3 (-54·7–160·8)	5·9 (3·4–9·3)	5·4 (3·9–7·6)	-7·6 (-46·2–75·4)	4·2 (2·3–6·8)	4 (2·7–5·9)	-4·5 (-48·4–95·6)
**Central Europe, Eastern Europe, and Central Asia**	0·3 (0·1–0·6)	0·3 (0·1–0·6)	9·6 (-64·7–283)	0·6 (0·2–1·2)	0·8 (0·4–1·6)	40·9 (-49·8–341·6)	0·4 (0·2–0·9)	0·5 (0·2–1·1)	31 (-54·3–331·6)
Central Asia	0·3 (0·1–0·7)	0·3 (0·1–0·7)	5·1 (-65·9–269·7)	2 (0·8–4)	2·5 (1·1–4·7)	23·6 (-53·6–278·7)	1·1 (0·4–2·3)	1·4 (0·6–2·6)	22·5 (-55·2–289·3)
Armenia	0·2 (0·1–0·5)	0·2 (0·1–0·4)	-13·3 (-72–203·4)	0·3 (0·1–0·6)	0·3 (0·1–0·6)	-0·9 (-66·1–226)	0·2 (0·1–0·5)	0·2 (0·1–0·5)	-7·4 (-69·1–213·6)
Azerbaijan	0·2 (0·1–0·4)	0·2 (0·1–0·4)	-1·4 (-68·2–245·1)	0·4 (0·1–0·8)	0·4 (0·1–0·7)	-5·8 (-67·8–209·6)	0·3 (0·1–0·6)	0·3 (0·1–0·6)	-3·4 (-67·6–224·3)
Georgia	0·3 (0·1–0·6)	0·3 (0·1–0·6)	10·7 (-64·2–287·5)	0·3 (0·1–0·6)	0·3 (0·1–0·6)	-3·9 (-67·2–215·8)	0·3 (0·1–0·6)	0·3 (0·1–0·6)	3·3 (-65·7–250·1)
Kazakhstan	0·2 (0·1–0·4)	0·2 (0·1–0·4)	-12 (-71·6–208·1)	0·5 (0·2–1)	0·4 (0·2–0·9)	-1 (-66·1–225·1)	0·3 (0·1–0·7)	0·3 (0·1–0·6)	-4·4 (-67·8–220·8)
Kyrgyzstan	0·5 (0·2–1)	0·4 (0·1–0·8)	-19·3 (-73·9–182·4)	6·3 (2·8–11·9)	7·2 (3·7–12·5)	13 (-55·9–229·3)	3·3 (1·5–6·2)	3·7 (1·9–6·6)	11·8 (-56·8–230·3)
Mongolia	0·3 (0·1–0·6)	0·2 (0·1–0·5)	-14·8 (-72·4–198·6)	0·4 (0·1–0·8)	0·3 (0·1–0·6)	-13·8 (-70·5–184·7)	0·3 (0·1–0·7)	0·3 (0·1–0·6)	-14·5 (-71·5–189·4)
Tajikistan	0·5 (0·2–1·2)	0·6 (0·2–1·3)	16·7 (-62·1–312·9)	0·6 (0·2–1·4)	0·6 (0·2–1·3)	-5·5 (-67·6–211·3)	0·6 (0·2–1·3)	0·6 (0·2–1·3)	4·7 (-65–257·6)
Turkmenistan	0·2 (0·1–0·4)	0·2 (0·1–0·4)	-5·1 (-69·4–232·2)	0·3 (0·1–0·6)	0·3 (0·1–0·6)	-7·5 (-68·4–203·9)	0·2 (0·1–0·5)	0·2 (0·1–0·5)	-5·4 (-68·3–218·4)
Uzbekistan	0·4 (0·2–1)	0·4 (0·2–0·9)	-3·2 (-68·7–238·3)	4·7 (1·8–9·6)	4·9 (2–9·7)	3·5 (-62·4–227·3)	2·5 (1–5·2)	2·6 (1·1–5·2)	3·6 (-62·8–230·1)
Central Europe	0·2 (0·1–0·5)	0·3 (0·1–0·7)	38·5 (-55·4–383·1)	0·4 (0·1–0·8)	0·5 (0·2–1·1)	42·7 (-50·9–371·1)	0·3 (0·1–0·6)	0·4 (0·2–0·9)	41 (-52·7–375·3)
Albania	0·4 (0·1–0·9)	0·3 (0·1–0·7)	-14·2 (-72·3–199·5)	1·3 (0·5–2·6)	1·1 (0·5–2·3)	-9·4 (-68·7–195·3)	0·8 (0·3–1·8)	0·7 (0·3–1·5)	-12·9 (-70·3–190·5)
Bosnia and Herzegovina	0·4 (0·1–0·8)	0·3 (0·1–0·7)	-9·4 (-70·7–215·5)	0·7 (0·3–1·5)	0·7 (0·3–1·4)	-6·5 (-67·8–205·1)	0·5 (0·2–1·2)	0·5 (0·2–1·1)	-8·3 (-69·1–205·8)
Bulgaria	0·3 (0·1–0·8)	0·5 (0·2–1·1)	52 (-50·7–433·7)	0·6 (0·2–1·3)	1 (0·4–2·1)	62·6 (-44–438·9)	0·5 (0·2–1)	0·8 (0·3–1·6)	58 (-46·7–437·5)
Croatia	0·2 (0·1–0·4)	0·2 (0·1–0·5)	10·4 (-64·4–286·4)	0·3 (0·1–0·6)	0·3 (0·1–0·7)	20·3 (-58·8–295·5)	0·2 (0·1–0·5)	0·3 (0·1–0·6)	15·8 (-61·4–291·3)

	Female			Male			Both		
	All-age prevalence (%)		Change (%)	All-age prevalence (%)		Change (%)	All-age prevalence (%)		Change (%)
	1990	2023		1990	2023		1990	2023	
Czechia	0·2 (0·1–0·5)	0·4 (0·1–0·8)	61·6 (-47·8–466·3)	0·3 (0·1–0·7)	0·6 (0·2–1·2)	89·2 (-35–528·2)	0·3 (0·1–0·6)	0·5 (0·2–1)	78·1 (-40·3–505·7)
Hungary	0·2 (0·1–0·4)	0·2 (0·1–0·4)	6·3 (-65·7–272·1)	0·2 (0·1–0·5)	0·2 (0·1–0·5)	8·1 (-63·1–255·2)	0·2 (0·1–0·4)	0·2 (0·1–0·4)	7·3 (-64·3–262·8)
Montenegro	0·3 (0·1–0·7)	0·4 (0·1–0·8)	13 (-63·4–295·1)	0·6 (0·2–1·2)	0·7 (0·3–1·4)	16·7 (-59·9–282·5)	0·5 (0·2–1)	0·5 (0·2–1·1)	15·2 (-61·3–286·2)
North Macedonia	0·3 (0·1–0·6)	0·3 (0·1–0·7)	15·1 (-62·8–302·6)	0·4 (0·2–1)	0·5 (0·2–1·1)	21·6 (-58·4–299·1)	0·4 (0·1–0·8)	0·4 (0·2–0·9)	18·8 (-60·2–299·5)
Poland	0·2 (0·1–0·5)	0·4 (0·1–0·8)	73·3 (-44–504·7)	0·3 (0·1–0·6)	0·5 (0·2–1·1)	78·2 (-39–483·1)	0·3 (0·1–0·5)	0·4 (0·2–0·9)	76 (-41·3–491·9)
Romania	0·2 (0·1–0·4)	0·2 (0·1–0·4)	9·6 (-64·6–284)	0·3 (0·1–0·7)	0·4 (0·2–0·9)	40·7 (-51·8–363·3)	0·2 (0·1–0·5)	0·3 (0·1–0·6)	28·8 (-56·7–333·7)
Serbia	0·3 (0·1–0·6)	0·3 (0·1–0·7)	19·1 (-61·5–316·8)	0·5 (0·2–1)	0·6 (0·2–1·2)	25·6 (-56·9–312·4)	0·4 (0·1–0·8)	0·5 (0·2–1)	23·6 (-58·5–315·3)
Slovakia	0·1 (0·1–0·3)	0·2 (0·1–0·4)	10 (-64·5–285·2)	0·2 (0·1–0·4)	0·2 (0·1–0·5)	16·8 (-60·1–284·4)	0·2 (0·1–0·4)	0·2 (0·1–0·4)	13·9 (-62–285·2)
Slovenia	0·2 (0·1–0·4)	0·3 (0·1–0·7)	68·1 (-45·7–489·1)	0·3 (0·1–0·6)	0·6 (0·2–1·2)	112·3 (-27·3–599·3)	0·2 (0·1–0·5)	0·4 (0·2–0·9)	95·4 (-34·5–561·9)
Eastern Europe	0·3 (0·1–0·6)	0·3 (0·1–0·5)	-3·9 (-69·1–234·6)	0·4 (0·1–0·8)	0·3 (0·1–0·7)	-6 (-67·9–208·8)	0·3 (0·1–0·7)	0·3 (0·1–0·6)	-5 (-68·5–220·5)
Belarus	0·2 (0·1–0·5)	0·2 (0·1–0·4)	-10·6 (-71·2–212·7)	0·4 (0·1–0·7)	0·4 (0·1–0·8)	2·8 (-64·8–238·4)	0·3 (0·1–0·6)	0·3 (0·1–0·6)	-3 (-67·5–227·4)
Estonia	0·2 (0·1–0·4)	0·3 (0·1–0·7)	101·8 (-34·7–607·8)	0·2 (0·1–0·4)	0·7 (0·3–1·5)	265·1 (25·4–1113·8)	0·2 (0·1–0·4)	0·5 (0·2–1·1)	185·2 (-4·5–877·4)
Latvia	0·2 (0·1–0·4)	0·2 (0·1–0·4)	14·7 (-63–301·6)	0·2 (0·1–0·4)	0·2 (0·1–0·5)	28 (-56·3–321·9)	0·2 (0·1–0·4)	0·2 (0·1–0·4)	21·1 (-59·8–311·8)
Lithuania	0·2 (0·1–0·4)	0·2 (0·1–0·4)	12·4 (-63·7–293·6)	0·2 (0·1–0·4)	0·3 (0·1–0·5)	23 (-57·9–305)	0·2 (0·1–0·4)	0·2 (0·1–0·5)	17·8 (-60·8–299·4)
Republic of Moldova	0·2 (0·1–0·5)	0·2 (0·1–0·5)	-10·9 (-71·2–211·9)	0·3 (0·1–0·7)	0·4 (0·1–0·7)	3 (-64·8–238·7)	0·3 (0·1–0·6)	0·3 (0·1–0·6)	-3·1 (-67·6–227·8)
Russian Federation	0·3 (0·1–0·7)	0·3 (0·1–0·6)	-7·1 (-70–225·1)	0·4 (0·1–0·8)	0·3 (0·1–0·7)	-10·1 (-69·2–195·8)	0·3 (0·1–0·7)	0·3 (0·1–0·6)	-8·6 (-69·6–209·5)
Ukraine	0·2 (0·1–0·4)	0·2 (0·1–0·4)	-1·6 (-68·2–244·7)	0·3 (0·1–0·7)	0·3 (0·1–0·7)	-3·1 (-66·8–218·8)	0·3 (0·1–0·6)	0·3 (0·1–0·5)	-2·4 (-67·4–229·2)

	Female			Male			Both		
	All-age prevalence (%)		Change (%)	All-age prevalence (%)		Change (%)	All-age prevalence (%)		Change (%)
	1990	2023		1990	2023		1990	2023	
**High-income**	0·2 (0·1–0·5)	0·3 (0·1–0·7)	42·9 (-53·5–392·1)	1·6 (1–2·5)	1·7 (1–2·6)	2·9 (-47·4–121·1)	0·9 (0·5–1·5)	1 (0·6–1·6)	9·1 (-47·2–146)
Australasia	0·2 (0·1–0·5)	0·3 (0·1–0·6)	17·6 (-61·9–312·5)	0·7 (0·3–1·5)	0·7 (0·3–1·4)	0·5 (-65·6–228·9)	0·5 (0·2–1)	0·5 (0·2–1)	5 (-64·6–249·7)
Australia	0·2 (0·1–0·5)	0·3 (0·1–0·6)	20·6 (-61·1–321·9)	0·7 (0·3–1·5)	0·7 (0·3–1·5)	-0·6 (-65·9–225·7)	0·5 (0·2–1)	0·5 (0·2–1)	4·3 (-64·8–246·7)
New Zealand	0·4 (0·1–0·8)	0·4 (0·1–0·8)	10·7 (-64·2–287·1)	0·5 (0·2–1·1)	0·6 (0·2–1·2)	6·8 (-63·4–250·5)	0·4 (0·2–0·9)	0·5 (0·2–1)	8·7 (-63·6–265·9)
High-income Asia Pacific	0·3 (0·1–0·7)	0·5 (0·2–1)	53·7 (-50·3–438·2)	0·7 (0·3–1·6)	0·9 (0·3–1·8)	15·7 (-60·2–279·2)	0·5 (0·2–1·1)	0·7 (0·3–1·4)	27·2 (-57·2–324·6)
Brunei Darussalam	0·8 (0·3–1·8)	1 (0·4–2·1)	15·9 (-62·2–304·5)	0·9 (0·3–1·9)	1 (0·4–2·2)	12·8 (-61·1–269·6)	0·9 (0·3–1·9)	1 (0·4–2·1)	14·1 (-61·6–284)
Japan	0·3 (0·1–0·7)	0·5 (0·2–1)	53·3 (-50·4–436·7)	0·8 (0·3–1·7)	0·9 (0·4–1·9)	13·4 (-61–271·5)	0·5 (0·2–1·2)	0·7 (0·3–1·4)	25 (-57·9–316·8)
Republic of Korea	0·3 (0·1–0·6)	0·5 (0·2–1)	55·1 (-49·8–443)	0·6 (0·2–1·3)	0·8 (0·3–1·6)	28·2 (-56–320·6)	0·4 (0·2–1)	0·6 (0·2–1·3)	37·3 (-53·9–359·6)
Singapore	0·3 (0·1–0·7)	0·5 (0·2–1·1)	67·1 (-45·9–485·1)	0·8 (0·3–1·7)	0·9 (0·4–1·9)	16·2 (-60–280·8)	0·6 (0·2–1·2)	0·7 (0·3–1·5)	31·7 (-55·6–338·8)
High-income North America	0·4 (0·1–0·8)	0·5 (0·2–1·1)	44·7 (-52·3–391·2)	4·5 (2·8–6·5)	4·1 (2·5–6·3)	-8·7 (-51·3–76·8)	2·3 (1·5–3·5)	2·3 (1·4–3·5)	-3·1 (-49·8–99·9)
Canada	0·5 (0·2–1)	0·7 (0·2–1·4)	39·9 (-54·5–383·6)	2·6 (1–5·3)	1·7 (0·7–3·5)	-36 (-76·9–93·5)	1·5 (0·6–3·1)	1·2 (0·5–2·4)	-24 (-73·3–135·5)
Greenland	0·3 (0·1–0·7)	0·4 (0·2–0·9)	24·3 (-59·8–334·7)	3·8 (1·5–7·9)	3·6 (1·5–7·3)	-5·6 (-66–203·5)	2·3 (0·9–4·7)	2·1 (0·9–4·3)	-6·6 (-66·7–201·5)
United States of America	0·3 (0·1–0·7)	0·5 (0·2–1·1)	45 (-52·1–390·8)	4·7 (3–6·7)	4·3 (2·7–6·8)	-6·7 (-49·6–77)	2·4 (1·5–3·5)	2·4 (1·5–3·7)	-1·3 (-48·5–96·4)
Southern Latin America	0·2 (0·1–0·5)	0·2 (0·1–0·4)	-7·8 (-70·3–222·4)	0·2 (0·1–0·4)	0·2 (0·1–0·4)	-13·5 (-70·5–184·3)	0·2 (0·1–0·5)	0·2 (0·1–0·4)	-10·5 (-70·4–203·7)
Argentina	0·2 (0·1–0·5)	0·2 (0·1–0·4)	-9·1 (-70·7–218)	0·2 (0·1–0·4)	0·2 (0·1–0·3)	-4·9 (-67·6–212·6)	0·2 (0·1–0·4)	0·2 (0·1–0·4)	-7·3 (-69·4–216)
Chile	0·2 (0·1–0·5)	0·2 (0·1–0·4)	-5·5 (-69·5–230·5)	0·3 (0·1–0·7)	0·2 (0·1–0·5)	-26·4 (-74·9–141·7)	0·3 (0·1–0·6)	0·2 (0·1–0·5)	-17·6 (-72·6–177·5)

	Female			Male			Both		
	All-age prevalence (%)		Change (%)	All-age prevalence (%)		Change (%)	All-age prevalence (%)		Change (%)
	1990	2023		1990	2023		1990	2023	
Uruguay	0·2 (0·1–0·4)	0·2 (0·1–0·4)	-6·1 (-69·7–228·7)	0·2 (0·1–0·4)	0·2 (0·1–0·4)	-6·9 (-68·2–206·3)	0·2 (0·1–0·4)	0·2 (0·1–0·4)	-6·4 (-69–217·9)
Western Europe	0·1 (0–0·3)	0·2 (0·1–0·4)	30·9 (-57·8–358·4)	0·2 (0·1–0·4)	0·3 (0·1–0·6)	44·6 (-50·2–377)	0·2 (0·1–0·4)	0·2 (0·1–0·5)	39·5 (-53·1–371·5)
Andorra	0·1 (0–0·3)	0·2 (0·1–0·4)	20 (-61·3–320·1)	0·2 (0·1–0·5)	0·3 (0·1–0·6)	31·7 (-55–332·9)	0·2 (0·1–0·4)	0·2 (0·1–0·5)	26·6 (-57·6–325·7)
Austria	0·1 (0–0·3)	0·2 (0·1–0·4)	47·8 (-52·3–417·6)	0·2 (0·1–0·4)	0·4 (0·1–0·7)	80·3 (-38·4–493·2)	0·2 (0·1–0·4)	0·3 (0·1–0·6)	67·3 (-44·1–466·2)
Belgium	0·1 (0–0·3)	0·1 (0–0·3)	4·3 (-66·4–265·1)	0·1 (0·1–0·3)	0·2 (0·1–0·3)	7·2 (-63·4–252·5)	0·1 (0–0·3)	0·1 (0·1–0·3)	5·9 (-64·8–258·6)
Cyprus	0·1 (0–0·3)	0·1 (0·1–0·3)	8·1 (-65·1–278·4)	0·2 (0·1–0·3)	0·2 (0·1–0·4)	12 (-61·8–268·3)	0·1 (0·1–0·3)	0·2 (0·1–0·3)	9·9 (-63·4–271·5)
Denmark	0·2 (0·1–0·4)	0·3 (0·1–0·6)	62·2 (-47·6–468·2)	0·3 (0·1–0·5)	0·5 (0·2–1)	81·5 (-37·9–496·9)	0·2 (0·1–0·5)	0·4 (0·1–0·8)	74·3 (-41·6–487·7)
Finland	0·1 (0–0·3)	0·2 (0·1–0·4)	39·2 (-55·1–387·4)	0·3 (0·1–0·5)	0·6 (0·2–1·2)	126 (-22·4–646·6)	0·2 (0·1–0·4)	0·4 (0·1–0·8)	97·7 (-33·3–568·3)
France	0·1 (0–0·3)	0·1 (0·1–0·3)	17·5 (-62·1–311·5)	0·2 (0·1–0·3)	0·2 (0·1–0·4)	24·6 (-57·5–309·8)	0·1 (0·1–0·3)	0·2 (0·1–0·4)	21·3 (-59·6–310·3)
Germany	0·1 (0–0·3)	0·2 (0·1–0·5)	67·1 (-46·1–485·6)	0·2 (0·1–0·3)	0·3 (0·1–0·7)	100·2 (-31·6–558·8)	0·1 (0·1–0·3)	0·3 (0·1–0·6)	85·4 (-38·1–528·7)
Greece	0·1 (0–0·3)	0·1 (0·1–0·3)	12·3 (-63·8–293·4)	0·2 (0·1–0·3)	0·2 (0·1–0·4)	18·4 (-59·6–289·2)	0·1 (0·1–0·3)	0·2 (0·1–0·3)	15·5 (-61·6–290·9)
Iceland	0·2 (0·1–0·5)	0·3 (0·1–0·6)	41·8 (-54·2–396·6)	0·6 (0·2–1·3)	1·4 (0·6–2·9)	126·7 (-21·6–651·1)	0·4 (0·2–0·9)	0·9 (0·3–1·8)	109·5 (-28·6–605·2)
Ireland	0·1 (0–0·3)	0·1 (0·1–0·3)	14·1 (-63·2–299·4)	0·2 (0·1–0·3)	0·2 (0·1–0·4)	27·2 (-56·6–318·2)	0·1 (0·1–0·3)	0·2 (0·1–0·4)	21·2 (-59·6–309·9)
Israel	0·1 (0–0·3)	0·2 (0·1–0·4)	21 (-61–323·7)	0·2 (0·1–0·5)	0·3 (0·1–0·6)	33·3 (-54·5–338·2)	0·2 (0·1–0·4)	0·2 (0·1–0·5)	28·7 (-57–334·3)
Italy	0·1 (0–0·3)	0·2 (0·1–0·3)	21 (-61–323·8)	0·2 (0·1–0·3)	0·2 (0·1–0·5)	45 (-50·5–377·2)	0·1 (0·1–0·3)	0·2 (0·1–0·4)	34·4 (-55·1–355)
Luxembourg	0·1 (0–0·3)	0·1 (0–0·3)	4·6 (-66·3–266·3)	0·1 (0·1–0·3)	0·2 (0·1–0·3)	7·8 (-63·2–254·3)	0·1 (0–0·3)	0·1 (0·1–0·3)	6·6 (-64·6–261)
Malta	0·1 (0–0·3)	0·1 (0–0·3)	4·2 (-66·4–264·9)	0·2 (0·1–0·3)	0·2 (0·1–0·4)	11·2 (-62·1–265·4)	0·1 (0·1–0·3)	0·2 (0·1–0·3)	9·3 (-63·5–269·9)
Monaco	0·1 (0–0·3)	0·2 (0·1–0·4)	20·8 (-61–322·8)	0·2 (0·1–0·5)	0·3 (0·1–0·6)	31·4 (-55·1–332)	0·2 (0·1–0·4)	0·2 (0·1–0·5)	28 (-57·3–332)

	Female			Male			Both		
	All-age prevalence (%)		Change (%)	All-age prevalence (%)		Change (%)	All-age prevalence (%)		Change (%)
	1990	2023		1990	2023		1990	2023	
Netherlands	0·1 (0–0·3)	0·1 (0·1–0·3)	16·5 (-62·4–307·8)	0·2 (0·1–0·3)	0·2 (0·1–0·4)	18·1 (-59·7–288·3)	0·1 (0·1–0·3)	0·2 (0·1–0·3)	17·4 (-60·9–297·2)
Norway	0·3 (0·1–0·6)	0·5 (0·2–1)	77·3 (-42·6–522·4)	0·6 (0·2–1·2)	1·4 (0·6–2·9)	155 (-12–740)	0·4 (0·1–0·9)	0·9 (0·4–2)	133 (-21–686·4)
Portugal	0·1 (0–0·3)	0·1 (0·1–0·3)	14·6 (-63–301·3)	0·1 (0·1–0·3)	0·2 (0·1–0·4)	24·8 (-57·4–310·3)	0·1 (0–0·3)	0·2 (0·1–0·3)	19·7 (-60·2–305·7)
San Marino	0·1 (0–0·3)	0·2 (0·1–0·4)	18·5 (-61·8–314·8)	0·2 (0·1–0·5)	0·3 (0·1–0·6)	27·7 (-56·4–319·8)	0·2 (0·1–0·4)	0·2 (0·1–0·5)	24 (-58·5–318)
Spain	0·1 (0–0·3)	0·1 (0–0·3)	4 (-66·5–264)	0·1 (0·1–0·3)	0·2 (0·1–0·3)	7·1 (-63·5–252·1)	0·1 (0–0·3)	0·1 (0·1–0·3)	5·6 (-64·9–257·8)
Sweden	0·2 (0·1–0·4)	0·5 (0·2–1)	136·5 (-23·5–730·3)	2 (0·8–4)	2·5 (1–5·1)	28·2 (-54·7–312·1)	1·1 (0·4–2·2)	1·5 (0·6–3)	40·9 (-50·8–357·5)
Switzerland	0·1 (0·1–0·3)	0·2 (0·1–0·4)	21·7 (-60·8–325·9)	0·2 (0·1–0·5)	0·3 (0·1–0·7)	41·5 (-51·6–365·5)	0·2 (0·1–0·4)	0·3 (0·1–0·5)	34·2 (-55·1–352·2)
United Kingdom	0·1 (0–0·3)	0·1 (0–0·3)	4·4 (-66·3–265·5)	0·2 (0·1–0·4)	0·2 (0·1–0·4)	1·8 (-65·3–234·5)	0·1 (0·1–0·3)	0·2 (0·1–0·3)	3·1 (-65·7–248·1)
**Latin America and Caribbean**	0·4 (0·1–0·9)	0·4 (0·1–0·8)	-5·8 (-69·4–231·5)	0·6 (0·2–1·2)	0·6 (0·2–1·2)	-0·2 (-65·5–228·2)	0·5 (0·2–1)	0·5 (0·2–1)	-2·8 (-67·2–231·8)
Andean Latin America	0·5 (0·2–1·1)	0·3 (0·1–0·7)	-32 (-77·9–137·4)	0·6 (0·2–1·2)	0·4 (0·2–0·9)	-22·9 (-73·5–152·8)	0·5 (0·2–1·1)	0·4 (0·1–0·8)	-27 (-75·6–146·1)
Bolivia (Plurinational State of)	0·3 (0·1–0·7)	0·3 (0·1–0·5)	-20·3 (-74·3–178·4)	0·5 (0·2–1)	0·4 (0·1–0·8)	-21 (-72·9–159·5)	0·4 (0·1–0·8)	0·3 (0·1–0·7)	-20·5 (-73·4–167·6)
Ecuador	0·2 (0·1–0·5)	0·2 (0·1–0·4)	-11·3 (-71·4–210·4)	0·2 (0·1–0·5)	0·2 (0·1–0·4)	-14·5 (-70·8–180·9)	0·2 (0·1–0·5)	0·2 (0·1–0·4)	-13·1 (-71·1–193·6)
Peru	0·7 (0·2–1·4)	0·4 (0·2–0·9)	-35·1 (-78·9–126·1)	0·8 (0·3–1·6)	0·6 (0·2–1·2)	-23·8 (-73·8–149·5)	0·7 (0·3–1·5)	0·5 (0·2–1·1)	-28·9 (-76·2–139·6)
Caribbean	0·3 (0·1–0·7)	0·3 (0·1–0·7)	1·3 (-67·2–256·4)	0·6 (0·2–1·3)	0·6 (0·3–1·3)	9·9 (-62·2–257·7)	0·5 (0·2–1)	0·5 (0·2–1)	6·7 (-64–259·5)
Antigua and Barbuda	0·5 (0·2–1)	0·4 (0·1–0·8)	-19·3 (-73·8–181·8)	0·9 (0·3–1·8)	0·7 (0·3–1·4)	-25 (-74·1–145)	0·7 (0·2–1·4)	0·5 (0·2–1·1)	-22·5 (-73·8–159·2)
Bahamas	0·3 (0·1–0·6)	0·3 (0·1–0·6)	4·4 (-66·3–265·2)	0·8 (0·3–1·6)	0·7 (0·3–1·4)	-12·9 (-70–185·4)	0·5 (0·2–1·1)	0·5 (0·2–1)	-8·7 (-69·2–203·9)
Barbados	0·3 (0·1–0·6)	0·3 (0·1–0·6)	5·1 (-66–268)	0·4 (0·1–0·7)	0·3 (0·1–0·7)	-5·8 (-67·7–209·6)	0·3 (0·1–0·7)	0·3 (0·1–0·6)	-0·7 (-66·9–235·4)
Belize	0·4 (0·1–0·8)	0·4 (0·1–0·8)	-5·5 (-69·4–230·2)	0·4 (0·1–0·8)	0·3 (0·1–0·7)	-9 (-68·9–199·2)	0·4 (0·1–0·8)	0·4 (0·1–0·7)	-7·2 (-69·2–213·9)

	Female			Male			Both		
	All-age prevalence (%)		Change (%)	All-age prevalence (%)		Change (%)	All-age prevalence (%)		Change (%)
	1990	2023		1990	2023		1990	2023	
Bermuda	0·3 (0·1–0·6)	0·3 (0·1–0·5)	-3 (-68·7–239·3)	0·4 (0·2–0·9)	0·4 (0·2–0·8)	-13·8 (-70·5–182·8)	0·3 (0·1–0·7)	0·3 (0·1–0·7)	-9·6 (-69·8–203·6)
Cuba	0·3 (0·1–0·7)	0·3 (0·1–0·6)	-9·7 (-70·8–216·1)	0·3 (0·1–0·7)	0·3 (0·1–0·5)	-21·6 (-73·2–157·4)	0·3 (0·1–0·7)	0·3 (0·1–0·6)	-15·9 (-72·1–184·4)
Dominica	0·2 (0·1–0·4)	0·2 (0·1–0·4)	7·6 (-65·3–276·7)	0·4 (0·2–0·9)	0·4 (0·1–0·8)	-9·8 (-69·1–196·1)	0·3 (0·1–0·6)	0·3 (0·1–0·6)	-3 (-67·4–224·1)
Dominican Republic	0·3 (0·1–0·7)	0·3 (0·1–0·7)	0 (-67·7–253·1)	1·3 (0·5–2·9)	1·5 (0·6–2·9)	10·8 (-61·3–269·1)	0·8 (0·3–1·7)	0·9 (0·4–1·8)	9·9 (-62·4–270)
Grenada	0·4 (0·1–0·9)	0·4 (0·1–0·9)	-3·9 (-68·9–236)	0·7 (0·3–1·4)	0·6 (0·2–1·3)	-10·2 (-69·2–194·3)	0·5 (0·2–1·2)	0·5 (0·2–1·1)	-6·7 (-68·7–213·6)
Guyana	0·3 (0·1–0·5)	0·2 (0·1–0·5)	-5·6 (-69·5–231·1)	0·4 (0·1–0·8)	0·3 (0·1–0·7)	-10 (-69·2–197·9)	0·3 (0·1–0·7)	0·3 (0·1–0·6)	-8·2 (-69·3–210·9)
Haiti	0·4 (0·1–0·9)	0·4 (0·2–0·9)	8·4 (-64·8–284)	0·5 (0·2–1·1)	0·5 (0·2–1·1)	3·9 (-64·4–242·6)	0·5 (0·2–1)	0·5 (0·2–1)	6 (-64·6–261)
Jamaica	0·3 (0·1–0·7)	0·3 (0·1–0·7)	-2·8 (-68·6–239·7)	0·6 (0·2–1·3)	0·6 (0·2–1·2)	-5·2 (-67·4–210·6)	0·5 (0·2–1)	0·4 (0·2–0·9)	-3·9 (-67·8–222·1)
Puerto Rico	0·3 (0·1–0·6)	0·2 (0·1–0·5)	-12·1 (-71·6–207·5)	0·4 (0·1–0·8)	0·3 (0·1–0·7)	-13·3 (-70·3–186)	0·3 (0·1–0·7)	0·3 (0·1–0·6)	-12·8 (-70·9–195)
Saint Kitts and Nevis	0·2 (0·1–0·4)	0·2 (0·1–0·4)	4 (-66·5–263·8)	0·3 (0·1–0·6)	0·3 (0·1–0·6)	-1 (-66·2–225·2)	0·2 (0·1–0·5)	0·2 (0·1–0·5)	1 (-66·4–240·5)
Saint Lucia	0·4 (0·1–0·8)	0·3 (0·1–0·6)	-22·5 (-75–170·8)	0·5 (0·2–1)	0·4 (0·1–0·8)	-24 (-74–149·8)	0·4 (0·2–0·9)	0·3 (0·1–0·7)	-22·9 (-74·3–160)
Saint Vincent and the Grenadines	0·3 (0·1–0·6)	0·2 (0·1–0·5)	-14·9 (-72·5–197·7)	0·8 (0·3–1·6)	0·6 (0·2–1·2)	-22·8 (-73·4–153·3)	0·5 (0·2–1·1)	0·4 (0·2–0·9)	-19·4 (-72·7–168·5)
Suriname	0·4 (0·1–0·9)	0·3 (0·1–0·7)	-17 (-73·2–190)	0·9 (0·3–1·9)	0·7 (0·3–1·5)	-23·2 (-73·4–151·3)	0·7 (0·2–1·4)	0·5 (0·2–1·1)	-22·4 (-73·7–158·6)
Trinidad and Tobago	0·6 (0·2–1·3)	0·5 (0·2–1)	-16·3 (-72·9–192·2)	0·9 (0·3–1·9)	0·7 (0·3–1·4)	-22·7 (-73·3–153·4)	0·7 (0·3–1·6)	0·6 (0·2–1·2)	-20·2 (-73·1–167·8)
United States Virgin Islands	0·2 (0·1–0·4)	0·2 (0·1–0·5)	9·1 (-64·8–282·4)	0·3 (0·1–0·6)	0·3 (0·1–0·7)	21·2 (-58·4–301·1)	0·2 (0·1–0·5)	0·3 (0·1–0·6)	16·3 (-61–294·5)
Central Latin America	0·4 (0·1–0·9)	0·4 (0·1–0·8)	-8·7 (-70·4–218·8)	0·6 (0·2–1·2)	0·5 (0·2–1·1)	-5·8 (-67·5–208·5)	0·5 (0·2–1·1)	0·5 (0·2–1)	-7·2 (-68·9–212·3)
Colombia	0·8 (0·3–1·7)	0·6 (0·2–1·4)	-17·2 (-73–189)	0·8 (0·3–1·6)	0·7 (0·3–1·4)	-9·6 (-68·9–196·2)	0·8 (0·3–1·6)	0·7 (0·3–1·4)	-13·5 (-71–192·6)
Costa Rica	0·2 (0·1–0·4)	0·2 (0·1–0·4)	-5·3 (-69·4–231·4)	0·3 (0·1–0·5)	0·2 (0·1–0·5)	-5·9 (-67·9–209·2)	0·2 (0·1–0·5)	0·2 (0·1–0·5)	-5·5 (-68·5–219·1)
El Salvador	0·6 (0·2–1·3)	0·5 (0·2–1)	-19·7 (-74–180·2)	0·7 (0·2–1·4)	0·6 (0·2–1·2)	-12·6 (-70–186·3)	0·6 (0·2–1·3)	0·5 (0·2–1·1)	-16·6 (-72·2–181·8)

	Female			Male			Both		
	All-age prevalence (%)		Change (%)	All-age prevalence (%)		Change (%)	All-age prevalence (%)		Change (%)
	1990	2023		1990	2023		1990	2023	
Guatemala	0·3 (0·1–0·6)	0·3 (0·1–0·6)	-2·3 (-68·4–241·9)	0·3 (0·1–0·7)	0·3 (0·1–0·7)	-1·7 (-66·4–223·3)	0·3 (0·1–0·7)	0·3 (0·1–0·6)	-2·1 (-67·4–231·4)
Honduras	0·3 (0·1–0·6)	0·3 (0·1–0·6)	-2 (-68·3–243·3)	0·4 (0·1–0·8)	0·4 (0·2–0·8)	2·6 (-64·9–238·4)	0·3 (0·1–0·7)	0·3 (0·1–0·7)	0·2 (-66·5–238·9)
Mexico	0·2 (0·1–0·5)	0·2 (0·1–0·5)	-1 (-68·1–246·3)	0·3 (0·1–0·6)	0·3 (0·1–0·6)	6 (-63·8–248·1)	0·3 (0·1–0·6)	0·3 (0·1–0·6)	2·7 (-65·8–247·1)
Nicaragua	0·6 (0·2–1·3)	0·5 (0·2–1·1)	-12·3 (-71·6–206)	0·7 (0·3–1·5)	0·7 (0·3–1·4)	-7·9 (-68·3–202)	0·7 (0·2–1·4)	0·6 (0·2–1·2)	-9·8 (-69·8–204·4)
Panama	0·5 (0·2–1·2)	0·5 (0·2–1·1)	-4·9 (-69·2–232·8)	0·5 (0·2–1·1)	0·5 (0·2–1)	-4·4 (-67·3–214)	0·5 (0·2–1·1)	0·5 (0·2–1·1)	-4·7 (-68·3–222·9)
Venezuela (Bolivarian Republic of)	0·7 (0·2–1·4)	0·7 (0·3–1·5)	6·6 (-65·4–272·1)	1·6 (0·6–3·3)	1·6 (0·6–3·3)	1·3 (-64·6–231)	1·1 (0·4–2·4)	1·1 (0·4–2·4)	1·3 (-65·4–237·1)
Tropical Latin America	0·4 (0·1–0·9)	0·4 (0·2–0·9)	3·6 (-66·2–268·4)	0·5 (0·2–1·1)	0·6 (0·2–1·2)	11·1 (-61·4–268·8)	0·5 (0·2–1)	0·5 (0·2–1)	7·5 (-63·7–266·7)
Brazil	0·4 (0·1–0·8)	0·4 (0·1–0·8)	3·3 (-66·5–265·2)	0·5 (0·2–1)	0·6 (0·2–1·1)	11·4 (-61·8–266·7)	0·4 (0·2–0·9)	0·5 (0·2–1)	7·5 (-64·1–263·4)
Paraguay	1·1 (0·4–2·5)	1·2 (0·4–2·5)	0·8 (-67–251·1)	1·8 (0·7–3·8)	1·8 (0·7–3·7)	-0·5 (-65·3–224·9)	1·5 (0·5–3·2)	1·5 (0·6–3·1)	-0·2 (-66·1–233·8)
North Africa and Middle East	0·6 (0·2–1·2)	0·5 (0·2–1·1)	-8·3 (-66·7–200·6)	1·5 (0·6–3)	1·4 (0·7–2·6)	-4·8 (-61·5–178·5)	1·1 (0·4–2·1)	1 (0·5–1·9)	-4·8 (-62·6–186·3)
Afghanistan	0·3 (0·1–0·7)	0·3 (0·1–0·6)	-11·3 (-71·3–211·1)	2·2 (0·8–4·6)	3 (1·3–5·7)	34·4 (-52·4–342·3)	1·2 (0·5–2·5)	1·6 (0·7–3·2)	34·9 (-52·9–350·5)
Algeria	0·5 (0·2–1)	0·5 (0·2–1·1)	5·2 (-65·9–268·7)	2·7 (1·1–5·6)	2·7 (1·1–5·3)	-2·5 (-65·4–216·1)	1·6 (0·6–3·3)	1·6 (0·7–3·2)	-1·2 (-65·6–220·6)
Bahrain	0·5 (0·2–1·2)	0·5 (0·2–1)	-12·6 (-71·7–205·3)	1·1 (0·4–2·3)	1 (0·4–2)	-12·5 (-69·8–186)	0·9 (0·3–1·9)	0·8 (0·3–1·6)	-10 (-69·4–198·7)
Egypt	0·2 (0·1–0·5)	0·2 (0·1–0·5)	-3·9 (-69–236·5)	0·6 (0·2–1·3)	0·6 (0·2–1·2)	-6 (-67·8–208·6)	0·4 (0·2–0·9)	0·4 (0·2–0·8)	-4·9 (-67·9–217·6)
Iran (Islamic Republic of)	0·5 (0·2–1·1)	0·4 (0·1–0·9)	-17·6 (-73·3–187·8)	1 (0·4–2·2)	0·9 (0·3–1·8)	-14·3 (-70·5–180·3)	0·8 (0·3–1·6)	0·6 (0·3–1·3)	-15·5 (-71·5–181·7)
Iraq	0·3 (0·1–0·8)	0·3 (0·1–0·7)	-9 (-70·6–218·3)	0·6 (0·2–1·3)	0·6 (0·2–1·1)	-12 (-69·8–188·8)	0·5 (0·2–1·1)	0·4 (0·2–0·9)	-11 (-70·1–198·3)
Jordan	0·3 (0·1–0·7)	0·2 (0·1–0·5)	-28·4 (-76·9–150·4)	0·6 (0·2–1·3)	0·4 (0·2–0·8)	-37·1 (-78·4–106·3)	0·5 (0·2–1)	0·3 (0·1–0·7)	-33·8 (-77·8–121·3)
Kuwait	0·3 (0·1–0·7)	0·3 (0·1–0·6)	-5·8 (-69·6–229·6)	0·5 (0·2–1·1)	0·5 (0·2–1·1)	0·9 (-65·4–234·8)	0·4 (0·2–0·9)	0·4 (0·2–0·9)	1·5 (-65·7–242·2)
Lebanon	0·6 (0·2–1·3)	0·5 (0·2–1·2)	-8 (-70·2–221·5)	0·8 (0·3–1·7)	0·8 (0·3–1·6)	-4·7 (-67·2–213·3)	0·7 (0·3–1·5)	0·7 (0·3–1·4)	-6 (-68·5–217·3)

	Female			Male			Both		
	All-age prevalence (%)		Change (%)	All-age prevalence (%)		Change (%)	All-age prevalence (%)		Change (%)
	1990	2023		1990	2023		1990	2023	
Libya	0·4 (0·1–0·9)	0·3 (0·1–0·7)	-18·3 (-73·6–185·2)	0·9 (0·3–1·8)	0·8 (0·3–1·6)	-11·5 (-69·5–189·7)	0·6 (0·2–1·4)	0·5 (0·2–1·1)	-14·9 (-71·2–183·6)
Morocco	0·3 (0·1–0·7)	0·3 (0·1–0·6)	-12·1 (-71·6–207·5)	0·9 (0·3–1·8)	0·8 (0·3–1·6)	-9·2 (-67·8–197·3)	0·6 (0·2–1·2)	0·5 (0·2–1·1)	-9·3 (-68·7–200·1)
Oman	0·6 (0·2–1·3)	0·6 (0·2–1·2)	-1·9 (-68·2–242·6)	0·9 (0·3–1·9)	0·8 (0·3–1·6)	-14·4 (-70·5–180·2)	0·8 (0·3–1·7)	0·7 (0·3–1·5)	-11 (-69·8–196·2)
Palestine	0·4 (0·1–0·9)	0·3 (0·1–0·7)	-16·7 (-73·1–191·4)	1·3 (0·5–2·7)	1·2 (0·5–2·4)	-9 (-68·5–197·4)	0·8 (0·3–1·8)	0·8 (0·3–1·6)	-9·5 (-69·2–200·7)
Qatar	0·3 (0·1–0·7)	0·3 (0·1–0·6)	-16·3 (-72·9–193)	1·1 (0·4–2·2)	0·8 (0·3–1·7)	-20·6 (-72·4–159·2)	0·9 (0·3–1·8)	0·7 (0·3–1·4)	-20·4 (-72·5–162·5)
Saudi Arabia	0·4 (0·2–1)	0·5 (0·2–1·1)	11·9 (-63·8–291·3)	0·7 (0·3–1·6)	0·7 (0·3–1·4)	-7·1 (-68·1–204·3)	0·6 (0·2–1·3)	0·6 (0·2–1·3)	0 (-66·2–233·7)
Sudan	0·3 (0·1–0·6)	0·3 (0·1–0·6)	-7·9 (-70·2–222·5)	2·8 (1·1–5·7)	3·3 (1·5–6·2)	17·8 (-57·8–284·5)	1·5 (0·6–3·1)	1·8 (0·8–3·4)	18·2 (-58·2–288·7)
Syrian Arab Republic	0·3 (0·1–0·7)	0·4 (0·1–0·8)	26 (-59·2–341·5)	0·3 (0·1–0·6)	0·5 (0·2–0·9)	51·1 (-48·2–397·4)	0·3 (0·1–0·6)	0·4 (0·2–0·9)	38 (-53·9–368·5)
Tunisia	0·9 (0·3–1·8)	0·6 (0·2–1·4)	-23·6 (-74·9–163·4)	1·8 (0·7–3·7)	1·4 (0·6–2·9)	-21·8 (-72·4–152·3)	1·3 (0·5–2·8)	1 (0·4–2·1)	-23 (-73·5–153·4)
Türkiye	0·6 (0·2–1·3)	0·6 (0·2–1·3)	1·8 (-67–255·7)	0·8 (0·3–1·6)	0·7 (0·3–1·4)	-9·8 (-69–195·5)	0·7 (0·2–1·4)	0·6 (0·2–1·3)	-4·8 (-68·1–220·4)
United Arab Emirates	0·3 (0·1–0·6)	0·2 (0·1–0·5)	-17·3 (-73·3–189·2)	0·4 (0·2–0·9)	0·3 (0·1–0·7)	-24 (-74–149·3)	0·4 (0·1–0·8)	0·3 (0·1–0·6)	-21·5 (-73·4–161·2)
Yemen	5·4 (2·4–10·5)	3·5 (1·9–6·4)	-35·2 (-72·4–84·6)	13·9 (7·1–23·9)	8·9 (5·8–13·9)	-35·9 (-66·8–48)	9·7 (4·8–17·1)	6·2 (3·9–10·1)	-35·7 (-68·4–59·9)
South Asia	11·3 (5·6–19·6)	8·9 (5·1–14·5)	-21·1 (-65–88·4)	25·4 (14·2–39·5)	18·8 (14·2–25·1)	-25·8 (-57·1–39·8)	18·7 (10·1–29·9)	14 (9·8–19·8)	-25·2 (-59·9–48)
Bangladesh	20·3 (10·7–33·1)	22·8 (15·1–32·6)	12·5 (-41·1–128·7)	18·5 (9·5–30·6)	20·2 (16·4–25)	9·4 (-37–113·6)	19·3 (9·9–31·8)	21·5 (16·8–27·9)	11·3 (-36·9–118·4)
Bhutan	8·1 (3·3–15·9)	8·8 (4·9–14·4)	8·6 (-53·9–208·6)	20·2 (9·8–34·9)	20·5 (12·9–30·6)	1·8 (-50·3–127·2)	14·6 (6·8–26·2)	14·9 (9·3–22·9)	2·1 (-51·7–137·1)
India	11·3 (5·5–19·7)	8·1 (4·4–13·7)	-28·6 (-69·8–81·8)	27·4 (15·6–42)	19·2 (14·5–25·4)	-29·9 (-59·2–29)	19·7 (10·8–31·3)	13·7 (9·6–19·5)	-30·2 (-62·4–38)

	Female			Male			Both		
	All-age prevalence (%)		Change (%)	All-age prevalence (%)		Change (%)	All-age prevalence (%)		Change (%)
	1990	2023		1990	2023		1990	2023	
Nepal	5·4 (2·3–10·7)	5 (2·7–8·5)	-8·7 (-60·8–149·7)	33·7 (19·3–50·2)	29·7 (21·6–39·1)	-12 (-46·6–57·6)	19·4 (10·8–29·9)	16·8 (12–22·9)	-13·4 (-49·3–60·8)
Pakistan	3·8 (1·4–7·9)	3·8 (1·6–7·6)	-0·8 (-64·9–236·3)	13·7 (6·3–25)	13·5 (7·6–23)	-1·8 (-57·7–168·8)	9·1 (4·1–17·1)	8·8 (4·7–15·1)	-3·1 (-60·3–173·5)
**Southeast Asia, East Asia, and Oceania**	0·9 (0·3–1·9)	1·1 (0·5–2·2)	23·5 (-53·2–285·2)	1·4 (0·6–2·7)	1·6 (0·7–3·1)	19·1 (-55·7–257·1)	1·1 (0·5–2·3)	1·4 (0·6–2·6)	20·6 (-54·2–264)
East Asia	0·4 (0·1–0·9)	0·5 (0·2–1·2)	30·9 (-57·6–358·6)	0·9 (0·3–1·9)	1·1 (0·4–2·2)	22·8 (-57·7–303·2)	0·7 (0·2–1·4)	0·8 (0·3–1·7)	24·8 (-57·9–317·6)
China	0·4 (0·1–0·9)	0·5 (0·2–1·2)	31·8 (-57·3–361·6)	0·9 (0·3–1·9)	1·1 (0·4–2·3)	23·3 (-57·5–305·5)	0·7 (0·2–1·4)	0·8 (0·3–1·7)	25·5 (-57·6–320·1)
Democratic People's Republic of Korea	0·2 (0·1–0·4)	0·2 (0·1–0·4)	-1 (-68·1–246·3)	0·2 (0·1–0·4)	0·2 (0·1–0·4)	-1·5 (-66·4–223·8)	0·2 (0·1–0·4)	0·2 (0·1–0·4)	-1·3 (-67·3–235)
Taiwan	0·5 (0·2–1)	0·5 (0·2–1)	2·1 (-67–256·6)	0·7 (0·3–1·6)	0·7 (0·3–1·5)	-1·8 (-66·3–221·7)	0·6 (0·2–1·3)	0·6 (0·2–1·3)	-1·5 (-66·9–230)
Oceania	7·9 (3·4–16·5)	11·1 (6·2–19·4)	41 (-45·2–300·7)	6·5 (2·9–13·3)	6·3 (3–12·2)	-3·7 (-63·1–171·6)	7·2 (3·2–15)	8·6 (4·7–15·8)	20·2 (-52·5–235·7)
American Samoa	2·5 (0·9–5·4)	2·2 (0·9–4·7)	-11·6 (-70·3–204·4)	4·8 (1·8–9·7)	4·1 (1·7–8·2)	-14·1 (-68·5–172·3)	3·7 (1·4–7·6)	3·2 (1·3–6·5)	-13·3 (-69·1–182·9)
Cook Islands	2·6 (0·9–5·5)	2·1 (0·8–4·3)	-20·2 (-73·2–172·9)	3·9 (1·5–7·9)	3·4 (1·4–6·8)	-12·6 (-68·4–178·5)	3·2 (1·2–6·7)	2·7 (1·1–5·5)	-17 (-70·8–172)
Fiji	2 (0·7–4·2)	1·9 (0·7–3·9)	-4·6 (-68·3–230·6)	2·4 (0·9–5)	2·3 (0·9–4·7)	-3·2 (-65·8–214·9)	2·2 (0·8–4·6)	2·1 (0·8–4·3)	-3·8 (-67–222·4)
Guam	2·9 (1·1–6·2)	3·2 (1·6–5·7)	11·2 (-58·8–251·9)	8·6 (3·8–16·3)	8·5 (5·1–13·6)	-1·4 (-55·4–146·9)	6 (2·6–11·7)	5·9 (3·5–9·6)	-2 (-57·4–159)
Kiribati	4·1 (1·6–8·5)	6·8 (3·4–11·8)	65·1 (-37·9–415·3)	11·5 (5–21·4)	14·9 (8·2–24·3)	30·3 (-43·2–247·8)	7·7 (3·3–14·7)	10·7 (5·7–17·9)	39·8 (-41·9–289·7)
Marshall Islands	5·3 (2·2–10·7)	8·2 (4·5–13·8)	55·3 (-37·1–347·4)	13·1 (6·4–23)	14·3 (8·2–22·4)	8·8 (-50·8–167·8)	9·3 (4·3–17)	11·3 (6·4–18·1)	21·5 (-46·4–211·3)
Micronesia (Federated States of)	5·4 (2·1–10·9)	4·6 (2–8·9)	-14·9 (-69·1–168·8)	8·9 (3·8–16·8)	7·8 (3·6–14·3)	-12·8 (-65·2–145·3)	7·2 (2·9–13·8)	6·2 (2·8–11·7)	-13·5 (-66·8–153·7)
Nauru	0·7 (0·2–1·5)	0·7 (0·3–1·4)	-1·8 (-68·1–245·8)	0·8 (0·3–1·7)	0·8 (0·3–1·7)	0·6 (-65·3–232·9)	0·7 (0·3–1·6)	0·7 (0·3–1·5)	-0·6 (-66·6–239·4)
Niue	1·3 (0·5–2·8)	1·2 (0·4–2·5)	-9·4 (-70·2–217)	1·6 (0·6–3·3)	1·6 (0·7–3·2)	-1 (-64·7–215·9)	1·5 (0·5–3·1)	1·4 (0·6–2·8)	-5·3 (-67·5–215·2)

	Female			Male			Both		
	All-age prevalence (%)		Change (%)	All-age prevalence (%)		Change (%)	All-age prevalence (%)		Change (%)
	1990	2023		1990	2023		1990	2023	
Northern Mariana Islands	4·9 (1·9–9·9)	3·8 (1·6–7·5)	-21·6 (-72–150·8)	7·6 (3·2–14·7)	6·4 (2·9–11·9)	-16·4 (-67·6–146·9)	6·4 (2·6–12·5)	5·2 (2·3–9·8)	-18·8 (-69·3–146·8)
Palau	19 (8·3–34·7)	25·2 (13·7–40·6)	32·7 (-41·1–254)	19·7 (9–34·9)	23·9 (13·5–37·6)	21·3 (-45–206·3)	19·3 (8·7–34·8)	24·5 (13·6–39)	26·5 (-43·2–229·8)
Papua New Guinea	11·4 (4·7–22·1)	14·3 (7·7–23·8)	25·3 (-49·6–261·1)	8·3 (3·4–15·7)	7·2 (3·2–13·4)	-13·2 (-66·4–148·2)	9·8 (4·1–19)	10·6 (5·5–18·4)	8·5 (-55·8–210·3)
Samoa	0·9 (0·3–2)	1 (0·4–2·1)	3·6 (-66·2–266·1)	1·5 (0·5–3)	1·5 (0·6–3)	1·1 (-64·9–230·4)	1·2 (0·4–2·6)	1·2 (0·5–2·5)	1·1 (-65·7–240·4)
Solomon Islands	3 (1·1–6·5)	3 (1·2–6·2)	-1·3 (-66·8–237·5)	4 (1·6–8·3)	4·1 (1·7–8·1)	0·3 (-63·9–219·4)	3·6 (1·3–7·4)	3·5 (1·4–7·2)	-0·6 (-65·2–225·8)
Tokelau	1·7 (0·6–3·6)	1·8 (0·7–3·7)	4·3 (-65·5–262·3)	2·1 (0·8–4·3)	2·3 (0·9–4·7)	12·9 (-59·9–265·8)	1·9 (0·7–4)	2 (0·8–4·2)	9·4 (-62·4–265·9)
Tonga	0·5 (0·2–1·2)	0·5 (0·2–1)	-15·4 (-72·6–195·4)	0·8 (0·3–1·7)	0·7 (0·3–1·4)	-17·8 (-71·7–169·2)	0·7 (0·2–1·4)	0·5 (0·2–1·2)	-17·7 (-72·4–176·3)
Tuvalu	0·6 (0·2–1·4)	0·6 (0·2–1·2)	-8·8 (-70·4–218·6)	0·9 (0·3–1·9)	0·8 (0·3–1·6)	-13·4 (-70·2–183·7)	0·8 (0·3–1·6)	0·7 (0·3–1·4)	-9·8 (-69·7–204·2)
Vanuatu	2·7 (1–5·7)	2·6 (1–5·4)	-3·4 (-67·5–232·3)	4·6 (1·8–9·3)	4·5 (1·9–9)	-2·1 (-64·1–207·7)	3·7 (1·4–7·6)	3·5 (1·4–7·2)	-3·3 (-65·6–214·2)
Southeast Asia	2·2 (0·9–4·4)	2·2 (1·1–4·2)	-0·5 (-59·9–199·6)	2·8 (1·2–5·3)	2·8 (1·3–4·9)	-1·3 (-60–167·3)	2·5 (1·1–4·8)	2·5 (1·2–4·5)	-0·7 (-60·5–187·4)
Cambodia	9·4 (4·9–15·8)	8·1 (4·8–13)	-13·2 (-55·9–97·1)	1·9 (0·7–3·8)	1·8 (0·8–3·4)	-4·6 (-67–212·4)	6 (3–10·2)	5·1 (3·5–7·2)	-14·7 (-52·4–75·1)
Indonesia	1·5 (0·6–3)	1 (0·4–2·1)	-31·2 (-76·3–121·4)	0·3 (0·1–0·7)	0·5 (0·2–1)	53·4 (-47·5–404·5)	0·9 (0·3–1·9)	0·8 (0·3–1·6)	-17·6 (-71·5–166·1)
Lao People's Democratic Republic	4·9 (2–10)	5·6 (2·7–10·2)	14·3 (-59·1–254·6)	0·5 (0·2–1·2)	0·5 (0·2–1·1)	-4 (-67·1–215·5)	2·8 (1·1–5·8)	3·1 (1·5–5·5)	8·3 (-61·1–239·8)
Malaysia	1·2 (0·4–2·7)	1·6 (0·6–3·4)	30·3 (-56·9–351·1)	1·9 (0·7–4)	2·9 (1·2–5·8)	51·1 (-47·1–386·9)	1·6 (0·6–3·3)	2·3 (0·9–4·6)	45 (-50·1–378·6)
Maldives	2·3 (0·9–4·8)	2·9 (1·2–5·6)	25·9 (-57–321·9)	4·3 (1·9–8·4)	4·7 (2·3–8·4)	9·2 (-58·9–219·9)	3·3 (1·4–6·6)	4 (1·9–7·3)	21·1 (-55·9–266·4)
Mauritius	2·4 (0·9–5·2)	2·8 (1·1–5·8)	13·4 (-61·2–287·1)	1·5 (0·5–3·1)	1·6 (0·6–3·3)	8·5 (-62·3–254·4)	2 (0·7–4·1)	2·2 (0·9–4·5)	11·8 (-61·5–275·2)
Myanmar	7 (2·7–13·9)	7·7 (3·3–14·7)	10·6 (-59·8–245·4)	18·7 (8·9–32·9)	19·4 (9·9–31·8)	3·8 (-55·5–163·4)	12·7 (5·7–23·2)	13·2 (6·5–22·7)	4·5 (-56·8–174·5)
Philippines	1·1 (0·4–2·3)	0·9 (0·4–2)	-10·9 (-71–211·9)	2·4 (0·9–4·9)	2·5 (1–4·9)	3·4 (-63·6–232·3)	1·7 (0·7–3·6)	1·7 (0·7–3·4)	-0·6 (-66·1–223·4)

	Female			Male			Both		
	All-age prevalence (%)		Change (%)	All-age prevalence (%)		Change (%)	All-age prevalence (%)		Change (%)
	1990	2023		1990	2023		1990	2023	
Seychelles	1·8 (0·7–3·9)	1·9 (0·7–3·9)	2·5 (-65·4–252·6)	1·5 (0·6–3·1)	1·4 (0·6–3)	-3·2 (-66·4–216·3)	1·7 (0·6–3·5)	1·6 (0·6–3·4)	-1·6 (-66·4–230·8)
Sri Lanka	4·2 (1·6–8·5)	4·7 (1·9–9·4)	13·2 (-61–272·1)	11·4 (5–21·6)	12·8 (6–22·4)	11·8 (-56·5–217·6)	7·8 (3·2–14·9)	8·6 (3·9–15·5)	9·6 (-58·5–220·9)
Thailand	2·1 (0·8–4·3)	3·4 (1·4–6·8)	66·6 (-43·9–460·7)	0·9 (0·3–1·9)	1·3 (0·5–2·7)	44 (-50·3–375·1)	1·5 (0·6–3·1)	2·4 (1–4·9)	62 (-45–442·2)
Timor-Leste	1·7 (0·6–3·5)	1·7 (0·6–3·5)	-0·3 (-66·6–240·4)	1·1 (0·4–2·4)	1·3 (0·5–2·6)	12·8 (-61–272·4)	1·4 (0·5–2·9)	1·5 (0·6–3)	5·3 (-64·3–252·6)
Viet Nam	1 (0·4–2·1)	1·1 (0·4–2·3)	9·8 (-63·4–276·9)	0·4 (0·1–0·8)	0·4 (0·2–0·8)	-0·3 (-65·9–227·7)	0·7 (0·3–1·5)	0·8 (0·3–1·6)	5 (-64·8–257)
**Sub-Saharan Africa**	0·9 (0·3–1·9)	0·8 (0·4–1·6)	-8·3 (-67·3–207·5)	1·3 (0·5–2·6)	1·2 (0·6–2·2)	-8·9 (-65·2–173)	1·1 (0·4–2·3)	1 (0·5–1·9)	-8·8 (-66·3–184·4)
Central Sub-Saharan Africa	0·8 (0·3–1·7)	0·6 (0·2–1·2)	-32·3 (-77·5–137·2)	0·9 (0·3–1·9)	1·1 (0·4–2·2)	17·6 (-58·8–288·2)	0·9 (0·3–1·8)	0·8 (0·3–1·7)	-6·3 (-67·8–214·4)
Angola	0·3 (0·1–0·7)	0·3 (0·1–0·7)	0·2 (-67·6–250·6)	0·6 (0·2–1·3)	0·6 (0·2–1·2)	1·4 (-65·1–236·5)	0·5 (0·2–1)	0·4 (0·2–0·9)	-1·2 (-66·8–233·2)
Central African Republic	0·8 (0·3–1·7)	0·8 (0·3–1·7)	0·4 (-67·1–253·1)	0·6 (0·2–1·2)	0·6 (0·2–1·3)	3·7 (-64·3–242·9)	0·7 (0·3–1·5)	0·7 (0·3–1·5)	1·6 (-66–249·3)
Congo	0·7 (0·3–1·6)	0·8 (0·3–1·6)	3·5 (-66·3–263·5)	0·4 (0·2–0·9)	0·5 (0·2–1)	15·4 (-60·6–285·1)	0·6 (0·2–1·3)	0·6 (0·2–1·3)	7·3 (-64·4–271)
Democratic Republic of the Congo	1 (0·4–2·1)	0·6 (0·2–1·3)	-35·8 (-79·2–121·3)	1·1 (0·4–2·3)	1·3 (0·6–2·6)	23·1 (-57·3–303)	1 (0·4–2·2)	1 (0·4–2)	-4·6 (-67·7–214·9)
Equatorial Guinea	0·6 (0·2–1·3)	0·5 (0·2–1)	-18·6 (-73·6–183·8)	0·6 (0·2–1·3)	0·6 (0·3–1·3)	6·9 (-63·4–250·6)	0·6 (0·2–1·3)	0·6 (0·2–1·2)	-4·8 (-68·1–223·4)
Gabon	0·4 (0·1–0·8)	0·3 (0·1–0·7)	-7·5 (-70·1–224·5)	0·4 (0·1–0·8)	0·4 (0·2–0·9)	13·4 (-61·2–273·1)	0·4 (0·1–0·8)	0·4 (0·1–0·8)	2·7 (-65·8–248·5)
Eastern Sub-Saharan Africa	1·2 (0·4–2·5)	1·2 (0·6–2·3)	0·6 (-62·4–227·3)	2 (0·9–3·9)	1·9 (0·9–3·4)	-6·1 (-62·3–162·7)	1·6 (0·6–3·2)	1·5 (0·8–2·8)	-3·5 (-62·3–187·1)
Burundi	2·1 (0·7–4·4)	1·8 (0·7–3·6)	-11·9 (-71·2–206·4)	2·8 (1·1–5·6)	2·1 (0·9–4·1)	-24·5 (-73·2–144·5)	2·4 (0·9–5)	2 (0·8–3·9)	-18·5 (-72·2–170·8)
Comoros	2·9 (1·1–6)	2·3 (0·9–4·7)	-20·1 (-72·7–164·4)	4 (1·7–8·1)	3·5 (1·6–6·6)	-12·4 (-67·9–169)	3·4 (1·3–6·9)	2·9 (1·3–5·6)	-15·2 (-70–170·3)
Djibouti	1·1 (0·4–2·4)	1·1 (0·4–2·3)	-4 (-68·6–234·2)	1·6 (0·6–3·3)	1·4 (0·6–2·9)	-9·9 (-68·6–194·6)	1·4 (0·5–2·9)	1·3 (0·5–2·6)	-7·2 (-68·5–210·4)
Eritrea	0·3 (0·1–0·6)	0·3 (0·1–0·7)	4·7 (-66·2–266·4)	2·9 (1·1–6)	2·5 (1–5·1)	-13·9 (-69·6–177·9)	1·5 (0·6–3·2)	1·4 (0·6–2·9)	-7·4 (-67·6–202·2)
Ethiopia	0·4 (0·2–1)	0·4 (0·2–0·9)	-2·5 (-68·3–243·9)	1·8 (0·7–3·6)	1·5 (0·6–3)	-15·5 (-69·8–174·7)	1·1 (0·4–2·3)	1 (0·4–2)	-12·6 (-69·3–184·6)

	Female			Male			Both		
	All-age prevalence (%)		Change (%)	All-age prevalence (%)		Change (%)	All-age prevalence (%)		Change (%)
	1990	2023		1990	2023		1990	2023	
Kenya	1 (0·3–2·1)	0·8 (0·3–1·7)	-16·4 (-72·8–195)	1·1 (0·4–2·3)	1·1 (0·4–2·3)	1·1 (-65·2–231·4)	1 (0·4–2·2)	1 (0·4–2)	-7·2 (-68·9–214·6)
Madagascar	6·5 (2·7–13)	7·7 (4·2–12·9)	19·2 (-50·9–245·7)	13·3 (6·4–23·6)	12·5 (6·9–20·3)	-6·1 (-58·8–142·8)	9·9 (4·5–18·1)	10 (5·6–16·6)	1·9 (-56·6–173·6)
Malawi	1 (0·4–2·1)	0·7 (0·3–1·6)	-24·6 (-75·3–162·7)	0·5 (0·2–1·1)	0·5 (0·2–1·1)	0·9 (-65·4–232·9)	0·8 (0·3–1·6)	0·6 (0·2–1·3)	-16·2 (-72·1–187·9)
Mozambique	1·3 (0·5–2·8)	0·7 (0·3–1·5)	-45·8 (-82·1–87·4)	0·9 (0·4–2)	0·8 (0·3–1·6)	-19·3 (-72·1–163·4)	1·1 (0·4–2·4)	0·7 (0·3–1·5)	-35·5 (-78·3–118·2)
Rwanda	1·9 (0·7–3·9)	1·6 (0·6–3·3)	-14·1 (-71·4–191)	0·4 (0·1–0·7)	0·3 (0·1–0·6)	-23·6 (-73·9–151·5)	1·1 (0·4–2·4)	1 (0·4–2)	-15·5 (-71·8–183)
Somalia	0·9 (0·3–2)	0·9 (0·3–1·8)	-5·5 (-69·2–229)	1·1 (0·4–2·3)	1 (0·4–2·1)	-9·3 (-68·7–196·1)	1 (0·4–2·1)	0·9 (0·4–1·9)	-7·8 (-69–209·4)
South Sudan	1 (0·3–2·2)	1 (0·4–2·1)	0·9 (-67·1–251·6)	1·4 (0·5–2·9)	1·4 (0·5–2·8)	-3·2 (-66·4–216·4)	1·2 (0·4–2·6)	1·2 (0·5–2·5)	-2·9 (-67·1–224·5)
Uganda	0·9 (0·3–1·9)	0·8 (0·3–1·7)	-9·2 (-70·5–217·1)	0·7 (0·2–1·4)	0·7 (0·3–1·5)	12 (-61·7–270)	0·8 (0·3–1·6)	0·8 (0·3–1·6)	-0·4 (-66·9–239·1)
United Republic of Tanzania	0·6 (0·2–1·2)	0·5 (0·2–1·1)	-4·8 (-69·1–233·4)	0·8 (0·3–1·6)	0·7 (0·3–1·5)	-3·7 (-67–219·7)	0·7 (0·2–1·4)	0·6 (0·2–1·3)	-4 (-67·8–228·1)
Zambia	0·6 (0·2–1·4)	0·7 (0·2–1·4)	4·4 (-66·1–267·4)	0·5 (0·2–1·1)	0·6 (0·2–1·2)	11·9 (-61·6–268·7)	0·6 (0·2–1·2)	0·6 (0·2–1·3)	7·8 (-64·1–268·4)
Southern Sub-Saharan Africa	1·1 (0·4–2·5)	1·1 (0·4–2·3)	-5·7 (-68·1–223)	1·5 (0·6–3·1)	0·9 (0·4–1·9)	-39·4 (-78·8–95·7)	1·3 (0·5–2·8)	1 (0·4–2·1)	-24 (-74·1–154·2)
Botswana	4·4 (1·7–9)	5 (2·4–9·1)	14·6 (-57·5–254·5)	2·3 (0·9–4·9)	2·1 (0·9–4·4)	-8·2 (-67·8–198·7)	3·4 (1·3–7·1)	3·6 (1·7–6·8)	5·8 (-60·6–231·3)
Eswatini	0·4 (0·1–0·8)	0·5 (0·2–1)	29·3 (-58·1–359·7)	0·6 (0·2–1·3)	0·6 (0·2–1·3)	-1·9 (-66·4–226·9)	0·5 (0·2–1)	0·5 (0·2–1·1)	12·1 (-62·5–283·4)
Lesotho	0·5 (0·2–1)	0·5 (0·2–1)	1·7 (-66·8–256·1)	1·1 (0·4–2·2)	0·8 (0·3–1·7)	-20·4 (-72·7–163·9)	0·7 (0·3–1·5)	0·7 (0·3–1·4)	-8·6 (-69·4–207·8)
Namibia	0·7 (0·3–1·6)	0·8 (0·3–1·7)	7·5 (-64·7–276·1)	0·8 (0·3–1·7)	0·7 (0·3–1·5)	-7·1 (-68·2–209·2)	0·8 (0·3–1·6)	0·8 (0·3–1·6)	0·1 (-66·4–241·3)
South Africa	1·2 (0·4–2·6)	1 (0·4–2·2)	-16 (-72·2–191·8)	1·8 (0·7–3·6)	1 (0·4–2)	-45·4 (-80·8–75·4)	1·5 (0·5–3·1)	1 (0·4–2·1)	-32·7 (-77–123·1)
Zimbabwe	0·6 (0·2–1·3)	0·9 (0·3–1·8)	39·8 (-54·3–390)	0·5 (0·2–1·1)	0·5 (0·2–1)	-7 (-68·2–206·3)	0·6 (0·2–1·2)	0·7 (0·3–1·4)	20·1 (-60–308·6)
Western Sub-Saharan Africa	0·6 (0·2–1·2)	0·5 (0·2–1·1)	-7·7 (-69·9–223·4)	0·7 (0·3–1·5)	0·7 (0·3–1·3)	-7·5 (-67·8–202·5)	0·6 (0·2–1·3)	0·6 (0·2–1·2)	-8 (-68·7–210·7)
Benin	0·9 (0·3–1·9)	0·9 (0·3–1·8)	-0·1 (-67·2–249)	1·3 (0·5–2·8)	1·5 (0·6–3)	14·3 (-60·7–283)	1·1 (0·4–2·3)	1·2 (0·5–2·4)	9·6 (-63–275·2)

Return to Table of Contents

	Female			Male			Both		
	All-age prevalence (%)		Change (%)	All-age prevalence (%)		Change (%)	All-age prevalence (%)		Change (%)
	1990	2023		1990	2023		1990	2023	
Burkina Faso	1·9 (0·7–3·9)	1·5 (0·6–2·9)	-19·9 (-73·2–173·3)	1·3 (0·5–2·7)	1 (0·4–2)	-24 (-73·6–143·7)	1·6 (0·6–3·4)	1·3 (0·5–2·5)	-21·8 (-73·6–159·7)
Cabo Verde	0·5 (0·2–1·1)	0·4 (0·2–0·9)	-22 (-74·7–172·9)	0·4 (0·1–0·7)	0·3 (0·1–0·7)	-9·2 (-69–198·4)	0·4 (0·2–1)	0·4 (0·1–0·8)	-18·3 (-72·9–179·4)
Cameroon	0·8 (0·3–1·8)	0·8 (0·3–1·6)	-5·3 (-68·9–222·4)	0·5 (0·2–1·2)	0·5 (0·2–1·1)	-3·8 (-67–218)	0·7 (0·3–1·5)	0·7 (0·3–1·4)	-5·3 (-68–220·8)
Chad	0·8 (0·3–1·8)	0·8 (0·3–1·7)	-5·7 (-69·1–228·8)	0·8 (0·3–1·6)	0·7 (0·3–1·5)	-8·5 (-68·3–198·9)	0·8 (0·3–1·7)	0·8 (0·3–1·6)	-7·1 (-68·8–214·2)
Côte d'Ivoire	0·4 (0·1–0·8)	0·3 (0·1–0·7)	-13 (-71·9–203·9)	0·4 (0·1–0·8)	0·4 (0·2–0·8)	-1·7 (-66·4–223·3)	0·4 (0·1–0·8)	0·4 (0·1–0·7)	-6·9 (-68·9–215·1)
Gambia	0·4 (0·2–1)	0·4 (0·2–0·9)	-4·5 (-69–233·7)	0·6 (0·2–1·2)	0·5 (0·2–1·1)	-10·4 (-69·5–196·7)	0·5 (0·2–1·1)	0·5 (0·2–1)	-8·1 (-69·3–211·6)
Ghana	0·4 (0·1–0·9)	0·5 (0·2–1)	14·3 (-62·9–301·9)	0·6 (0·2–1·3)	0·6 (0·2–1·3)	0·4 (-65·7–230·1)	0·5 (0·2–1·1)	0·5 (0·2–1·1)	6·4 (-64·5–261·6)
Guinea	0·4 (0·1–0·9)	0·4 (0·1–0·8)	-17·1 (-73·1–189·5)	0·8 (0·3–1·6)	0·7 (0·3–1·5)	-4·5 (-67·2–213)	0·6 (0·2–1·3)	0·5 (0·2–1·1)	-9·2 (-69·5–204·6)
Guinea-Bissau	0·4 (0·1–0·9)	0·4 (0·1–0·8)	-11·8 (-71·5–207·9)	0·4 (0·2–0·9)	0·4 (0·1–0·8)	-14·8 (-70·9–179·5)	0·4 (0·2–0·9)	0·4 (0·1–0·8)	-13·3 (-71·2–193·7)
Liberia	0·4 (0·1–0·9)	0·4 (0·1–0·8)	-8·3 (-70·3–220·7)	1 (0·4–2)	1 (0·4–2·1)	7·2 (-63·3–252·4)	0·7 (0·3–1·5)	0·7 (0·3–1·5)	3 (-65·4–246·5)
Mali	0·4 (0·2–1)	0·4 (0·1–0·8)	-13·3 (-71·9–202·8)	1·7 (0·6–3·5)	1·5 (0·6–3)	-8·8 (-68–196)	1 (0·4–2·1)	1 (0·4–1·9)	-8·2 (-67·6–200·4)
Mauritania	0·8 (0·3–1·8)	0·9 (0·3–1·9)	5·5 (-65·6–268·5)	1·1 (0·4–2·2)	1·1 (0·4–2·3)	6·3 (-63·3–248·4)	0·9 (0·3–2)	1 (0·4–2·1)	5·8 (-64·4–256·9)
Niger	0·9 (0·3–1·9)	1 (0·4–2)	10·1 (-64·2–279·9)	2 (0·8–4·1)	1·9 (0·8–3·6)	-6·5 (-66·4–200)	1·4 (0·5–3)	1·4 (0·6–2·8)	-1·2 (-65·8–223·7)
Nigeria	0·3 (0·1–0·8)	0·3 (0·1–0·7)	-7·6 (-70–228·2)	0·5 (0·2–1·1)	0·4 (0·2–0·9)	-17·3 (-71·7–175·4)	0·4 (0·2–0·9)	0·4 (0·1–0·8)	-14·6 (-71·4–191)
Sao Tome and Principe	0·4 (0·1–0·9)	0·4 (0·2–0·9)	0·2 (-67·5–252·2)	0·5 (0·2–1·1)	0·7 (0·3–1·4)	22·1 (-58·1–306·1)	0·5 (0·2–1)	0·5 (0·2–1·1)	13·1 (-62·1–285·9)
Senegal	0·7 (0·2–1·5)	0·7 (0·3–1·4)	0·6 (-67·3–252·2)	0·4 (0·1–0·7)	0·3 (0·1–0·6)	-16·1 (-71·3–177·2)	0·5 (0·2–1·1)	0·5 (0·2–1)	-5·8 (-68·9–223·1)
Sierra Leone	1·2 (0·4–2·6)	0·9 (0·4–1·9)	-22·5 (-74·5–170·7)	1 (0·4–2·2)	0·9 (0·3–1·8)	-18 (-71·8–168·7)	1·1 (0·4–2·4)	0·9 (0·4–1·8)	-20·5 (-73·3–169·1)
Togo	0·8 (0·3–1·8)	0·8 (0·3–1·8)	2·3 (-66·4–259·7)	0·9 (0·3–1·8)	0·8 (0·3–1·7)	-8 (-68·3–203·7)	0·8 (0·3–1·8)	0·8 (0·3–1·7)	-2·7 (-67·3–231·9)

Return to Table of Contents

Appendix Table 2·5: Age-standardized prevalence of chewing tobacco use in 1990 and 2023, among ages 15+

	Female			Male			Both		
	Age-standardized prevalence (%)		Change (%)	Age-standardized prevalence (%)		Change (%)	Age-standardized prevalence (%)		Change (%)
	1990	2023		1990	2023		1990	2023	
Global	2·6 (1·3–4·6)	2·5 (1·4–4·2)	-5·7 (-58·1–136·5)	5·9 (3·4–9·3)	5·4 (3·9–7·5)	-8·9 (-46·8–71·7)	4·3 (2·4–7)	3·9 (2·7–5·8)	-7·7 (-49·8–88·4)
**Central Europe, Eastern Europe, and Central Asia**	0·3 (0·1–0·6)	0·3 (0·1–0·7)	15·3 (-62·8–302·7)	0·6 (0·2–1·2)	0·9 (0·4–1·8)	54·6 (-45·2–383·6)	0·4 (0·2–0·9)	0·6 (0·3–1·2)	43·2 (-50·2–372·4)
Central Asia	0·3 (0·1–0·7)	0·3 (0·1–0·7)	6·3 (-65·5–273·9)	1·9 (0·8–3·8)	2·4 (1–4·6)	28·2 (-52–294·6)	1·1 (0·4–2·1)	1·3 (0·6–2·6)	27·5 (-53·5–305)
Armenia	0·2 (0·1–0·5)	0·2 (0·1–0·4)	-8·4 (-70·4–220·8)	0·3 (0·1–0·6)	0·3 (0·1–0·6)	7 (-63·4–252)	0·2 (0·1–0·5)	0·2 (0·1–0·5)	-0·1 (-66·7–238·5)
Azerbaijan	0·2 (0·1–0·4)	0·2 (0·1–0·4)	3·4 (-66·6–262)	0·4 (0·1–0·8)	0·4 (0·1–0·8)	-3·7 (-67·1–216·6)	0·3 (0·1–0·6)	0·3 (0·1–0·6)	0·7 (-66·2–238·3)
Georgia	0·3 (0·1–0·6)	0·3 (0·1–0·7)	18·9 (-61·6–316·5)	0·3 (0·1–0·6)	0·3 (0·1–0·7)	6·9 (-63·4–251·7)	0·3 (0·1–0·6)	0·3 (0·1–0·7)	13·1 (-62·4–283·4)
Kazakhstan	0·2 (0·1–0·4)	0·2 (0·1–0·4)	-10 (-71–215)	0·4 (0·2–0·9)	0·5 (0·2–0·9)	2·2 (-65–235·7)	0·3 (0·1–0·7)	0·3 (0·1–0·7)	-0·9 (-66·7–232·4)
Kyrgyzstan	0·5 (0·2–1)	0·4 (0·1–0·8)	-21 (-74·5–176·3)	6·4 (2·8–12·1)	6·9 (3·6–12·3)	8·3 (-57·9–219·4)	3·3 (1·5–6·2)	3·5 (1·8–6·4)	8·7 (-58·4–222·9)
Mongolia	0·2 (0·1–0·5)	0·2 (0·1–0·5)	-4·9 (-69·3–233·2)	0·4 (0·1–0·8)	0·3 (0·1–0·7)	-17·8 (-71·9–171·1)	0·3 (0·1–0·7)	0·3 (0·1–0·6)	-12·5 (-70·8–195·3)
Tajikistan	0·6 (0·2–1·2)	0·6 (0·2–1·4)	15·6 (-62·4–308·9)	0·6 (0·2–1·3)	0·6 (0·2–1·2)	-4·1 (-67·1–215·9)	0·6 (0·2–1·3)	0·6 (0·2–1·3)	4·9 (-64·9–258·8)
Turkmenistan	0·2 (0·1–0·4)	0·2 (0·1–0·4)	-5·4 (-69·5–231·1)	0·3 (0·1–0·6)	0·3 (0·1–0·6)	-8 (-68·6–202·2)	0·2 (0·1–0·5)	0·2 (0·1–0·5)	-5·2 (-68·3–219·1)
Uzbekistan	0·4 (0·2–0·9)	0·4 (0·2–0·9)	-2·9 (-68·6–239·6)	4·3 (1·7–8·8)	4·7 (2–9·2)	8 (-60·9–242·5)	2·3 (0·9–4·8)	2·5 (1–5)	8 (-61·3–244·7)
Central Europe	0·2 (0·1–0·5)	0·3 (0·1–0·7)	43·3 (-53·8–400·4)	0·4 (0·1–0·8)	0·5 (0·2–1·1)	48·1 (-48·9–390)	0·3 (0·1–0·6)	0·4 (0·2–0·9)	46·7 (-50·6–395·8)
Albania	0·4 (0·1–0·8)	0·4 (0·1–0·8)	8·3 (-65–278·6)	1·2 (0·4–2·4)	1·3 (0·5–2·7)	13·4 (-60·8–269·7)	0·8 (0·3–1·6)	0·9 (0·3–1·8)	9 (-62·9–262·5)
Bosnia and Herzegovina	0·4 (0·1–0·8)	0·4 (0·1–0·8)	5·9 (-65·8–269·1)	0·7 (0·3–1·5)	0·8 (0·3–1·7)	17·4 (-59·5–284)	0·5 (0·2–1·1)	0·6 (0·2–1·3)	13 (-61·9–277·6)
Bulgaria	0·4 (0·1–0·8)	0·6 (0·2–1·3)	64·8 (-46·5–480·6)	0·7 (0·2–1·4)	1·2 (0·5–2·4)	76 (-39·4–482·3)	0·5 (0·2–1·1)	0·9 (0·3–1·8)	73·7 (-41·4–490·2)
Croatia	0·2 (0·1–0·5)	0·3 (0·1–0·6)	21 (-60·9–323·7)	0·3 (0·1–0·6)	0·4 (0·1–0·8)	34 (-54·1–340·7)	0·2 (0·1–0·5)	0·3 (0·1–0·7)	28·5 (-57·1–334·4)

	Female			Male			Both		
	Age-standardized prevalence (%)		Change (%)	Age-standardized prevalence (%)		Change (%)	Age-standardized prevalence (%)		Change (%)
	1990	2023		1990	2023		1990	2023	
Czechia	0·2 (0·1–0·5)	0·4 (0·1–0·8)	67 (-46–485·7)	0·3 (0·1–0·7)	0·6 (0·2–1·2)	95·6 (-32·9–549·7)	0·3 (0·1–0·6)	0·5 (0·2–1)	85 (-38–529·7)
Hungary	0·2 (0·1–0·4)	0·2 (0·1–0·4)	11·9 (-63·9–291·9)	0·2 (0·1–0·5)	0·3 (0·1–0·5)	14·2 (-61–275·4)	0·2 (0·1–0·4)	0·2 (0·1–0·5)	13·5 (-62·2–283·9)
Montenegro	0·3 (0·1–0·7)	0·4 (0·2–0·9)	28·6 (-58·4–349·7)	0·6 (0·2–1·2)	0·8 (0·3–1·6)	36·1 (-53·2–346·5)	0·4 (0·2–1)	0·6 (0·2–1·3)	33·6 (-55–348·2)
North Macedonia	0·3 (0·1–0·6)	0·4 (0·1–0·8)	31 (-57·6–358·2)	0·4 (0·2–0·9)	0·6 (0·2–1·3)	37·7 (-52·8–352)	0·4 (0·1–0·8)	0·5 (0·2–1)	35·1 (-54·7–354·6)
Poland	0·2 (0·1–0·5)	0·4 (0·1–0·8)	71·5 (-44·6–498·6)	0·3 (0·1–0·6)	0·5 (0·2–1·1)	75·5 (-40–474·5)	0·3 (0·1–0·5)	0·4 (0·2–0·9)	74·1 (-41·9–485·8)
Romania	0·2 (0·1–0·4)	0·2 (0·1–0·4)	17·3 (-62·1–310·9)	0·3 (0·1–0·7)	0·4 (0·2–0·9)	41·8 (-51·5–366·8)	0·2 (0·1–0·5)	0·3 (0·1–0·7)	33·7 (-55·1–350·4)
Serbia	0·3 (0·1–0·6)	0·4 (0·1–0·8)	28·5 (-58·4–349·8)	0·5 (0·2–1)	0·7 (0·3–1·4)	36·1 (-53·3–346·8)	0·4 (0·1–0·8)	0·5 (0·2–1·1)	34·2 (-54·9–351·2)
Slovakia	0·1 (0·1–0·3)	0·2 (0·1–0·4)	20·9 (-61–323·5)	0·2 (0·1–0·4)	0·3 (0·1–0·5)	27·8 (-56·3–320·9)	0·2 (0·1–0·4)	0·2 (0·1–0·5)	25·4 (-58·2–324·6)
Slovenia	0·2 (0·1–0·4)	0·3 (0·1–0·7)	69·2 (-45·3–492·7)	0·3 (0·1–0·6)	0·6 (0·2–1·1)	108·2 (-28·7–585·7)	0·2 (0·1–0·5)	0·4 (0·2–0·9)	94·3 (-34·9–557·9)
Eastern Europe	0·3 (0·1–0·6)	0·3 (0·1–0·6)	5·9 (-66–268)	0·4 (0·1–0·8)	0·4 (0·2–0·8)	3·1 (-64·8–238·3)	0·3 (0·1–0·7)	0·3 (0·1–0·7)	4·5 (-65·3–251·6)
Belarus	0·2 (0·1–0·5)	0·2 (0·1–0·4)	-9·9 (-70·9–215·4)	0·4 (0·1–0·8)	0·4 (0·2–0·8)	8·9 (-62·7–258·6)	0·3 (0·1–0·6)	0·3 (0·1–0·6)	1·7 (-65·9–243·3)
Estonia	0·2 (0·1–0·4)	0·3 (0·1–0·7)	98·3 (-35·9–595·4)	0·2 (0·1–0·4)	0·7 (0·3–1·4)	236 (15·4–1016·1)	0·2 (0·1–0·4)	0·5 (0·2–1·1)	174·4 (-8·1–839·5)
Latvia	0·2 (0·1–0·4)	0·2 (0·1–0·4)	25·1 (-59·6–338·6)	0·2 (0·1–0·4)	0·3 (0·1–0·5)	44·9 (-50·5–378·1)	0·2 (0·1–0·4)	0·2 (0·1–0·5)	35·9 (-54·7–362·7)
Lithuania	0·2 (0·1–0·4)	0·2 (0·1–0·5)	27·5 (-58·8–346·6)	0·2 (0·1–0·4)	0·3 (0·1–0·7)	50·3 (-48·6–395·3)	0·2 (0·1–0·4)	0·3 (0·1–0·6)	40·3 (-53·2–376·2)
Republic of Moldova	0·3 (0·1–0·6)	0·3 (0·1–0·6)	5·5 (-65·9–269·6)	0·3 (0·1–0·7)	0·4 (0·1–0·8)	8·1 (-63–255·3)	0·3 (0·1–0·6)	0·3 (0·1–0·7)	7·6 (-64·1–263·8)
Russian Federation	0·3 (0·1–0·7)	0·3 (0·1–0·7)	1·9 (-67–256·9)	0·4 (0·1–0·8)	0·4 (0·2–0·8)	-1·7 (-66·3–223·6)	0·4 (0·1–0·8)	0·4 (0·1–0·8)	0 (-66·6–238·8)
Ukraine	0·2 (0·1–0·5)	0·2 (0·1–0·5)	8 (-65·1–278·8)	0·3 (0·1–0·7)	0·4 (0·1–0·8)	5·3 (-64–246·4)	0·3 (0·1–0·6)	0·3 (0·1–0·6)	6·9 (-64·2–260·7)

Return to Table of Contents

	Female			Male			Both		
	Age-standardized prevalence (%)		Change (%)	Age-standardized prevalence (%)		Change (%)	Age-standardized prevalence (%)		Change (%)
	1990	2023		1990	2023		1990	2023	
**High-income**	0·2 (0·1–0·5)	0·4 (0·1–0·8)	47·1 (-52·2–408·7)	1·6 (1–2·5)	1·8 (1·1–2·7)	8 (-44·3–127·1)	0·9 (0·5–1·5)	1·1 (0·6–1·7)	15·6 (-44·5–165·8)
Australasia	0·3 (0·1–0·6)	0·3 (0·1–0·7)	23·9 (-59·9–334·6)	0·7 (0·3–1·5)	0·7 (0·3–1·4)	-0·4 (-65·9–225·8)	0·5 (0·2–1)	0·5 (0·2–1)	6·3 (-64·2–254·1)
Australia	0·2 (0·1–0·5)	0·3 (0·1–0·6)	26·3 (-59·2–342·1)	0·7 (0·3–1·5)	0·7 (0·3–1·5)	-0·9 (-66–224·8)	0·5 (0·2–1)	0·5 (0·2–1·1)	5·7 (-64·3–251·5)
New Zealand	0·4 (0·1–0·8)	0·4 (0·2–0·9)	19·4 (-61·4–317·6)	0·5 (0·2–1·1)	0·5 (0·2–1·1)	1·4 (-65·3–232·6)	0·4 (0·2–0·9)	0·5 (0·2–1)	9·3 (-63·5–267·9)
High-income Asia Pacific	0·3 (0·1–0·7)	0·5 (0·2–1)	53·8 (-50·2–438·5)	0·7 (0·3–1·6)	0·8 (0·3–1·7)	9·5 (-62·4–259)	0·5 (0·2–1·1)	0·6 (0·3–1·4)	24 (-58·3–313·8)
Brunei Darussalam	0·9 (0·3–2)	1 (0·4–2)	3·9 (-66·1–262·3)	1 (0·4–2·1)	1 (0·4–2·1)	2·8 (-64·5–236·4)	1 (0·4–2·1)	1 (0·4–2·1)	3·1 (-65·3–246·7)
Japan	0·3 (0·1–0·7)	0·5 (0·2–1·1)	60·2 (-48·2–460·8)	0·8 (0·3–1·7)	0·9 (0·3–1·8)	10·3 (-62·1–261·6)	0·5 (0·2–1·2)	0·7 (0·3–1·4)	25·8 (-57·6–319·3)
Republic of Korea	0·3 (0·1–0·6)	0·4 (0·2–0·9)	38·5 (-55·2–384·7)	0·6 (0·2–1·4)	0·7 (0·3–1·4)	8·5 (-62·8–255·8)	0·5 (0·2–1)	0·6 (0·2–1·2)	20·5 (-59·5–303·2)
Singapore	0·3 (0·1–0·7)	0·5 (0·2–1·1)	60·2 (-48·2–460·8)	0·8 (0·3–1·7)	0·9 (0·3–1·8)	10·3 (-62·1–261·6)	0·6 (0·2–1·2)	0·7 (0·3–1·5)	25·8 (-57·6–319·4)
High-income North America	0·3 (0·1–0·7)	0·5 (0·2–1·1)	51·9 (-50·2–418·5)	4·5 (2·9–6·5)	4·3 (2·6–6·6)	-5·9 (-49·3–80·8)	2·4 (1·5–3·5)	2·4 (1·5–3·7)	1·1 (-47·4–106·8)
Canada	0·5 (0·2–1)	0·7 (0·2–1·4)	38·5 (-54·9–377·8)	2·6 (1–5·3)	1·7 (0·7–3·4)	-36·4 (-77·1–92·6)	1·5 (0·6–3·1)	1·2 (0·5–2·4)	-23·6 (-73·2–137·2)
Greenland	0·3 (0·1–0·7)	0·4 (0·2–0·9)	20·4 (-61·1–321·1)	3·7 (1·4–7·6)	3·7 (1·5–7·5)	-0·2 (-64·1–221·4)	2·2 (0·8–4·5)	2·1 (0·9–4·4)	-1·3 (-64·8–219·3)
United States of America	0·3 (0·1–0·7)	0·5 (0·2–1·1)	53·8 (-49·5–423·9)	4·7 (3·1–6·8)	4·6 (2·8–7·1)	-3·9 (-47·8–80·6)	2·5 (1·5–3·6)	2·5 (1·6–3·9)	3 (-46–103·5)
Southern Latin America	0·2 (0·1–0·5)	0·2 (0·1–0·4)	-3·8 (-69–236·6)	0·2 (0·1–0·4)	0·2 (0·1–0·4)	-11·3 (-69·7–191·6)	0·2 (0·1–0·5)	0·2 (0·1–0·4)	-7·4 (-69·3–214·4)
Argentina	0·2 (0·1–0·5)	0·2 (0·1–0·4)	-5·8 (-69·6–229·6)	0·2 (0·1–0·3)	0·2 (0·1–0·3)	-2·9 (-66·9–219·3)	0·2 (0·1–0·4)	0·2 (0·1–0·4)	-4·5 (-68·4–225·4)
Chile	0·2 (0·1–0·5)	0·2 (0·1–0·4)	-1·2 (-68·1–245·7)	0·3 (0·1–0·7)	0·2 (0·1–0·5)	-24·3 (-74·1–148·6)	0·3 (0·1–0·6)	0·2 (0·1–0·5)	-14·3 (-71·5–188·4)

	Female			Male			Both		
	Age-standardized prevalence (%)		Change (%)	Age-standardized prevalence (%)		Change (%)	Age-standardized prevalence (%)		Change (%)
	1990	2023		1990	2023		1990	2023	
Uruguay	0·2 (0·1–0·4)	0·2 (0·1–0·4)	-4·5 (-69·2–234·4)	0·2 (0·1–0·4)	0·2 (0·1–0·4)	-5·6 (-67·8–210·4)	0·2 (0·1–0·4)	0·2 (0·1–0·4)	-5 (-68·5–222·8)
Western Europe	0·1 (0–0·3)	0·2 (0·1–0·4)	35·1 (-56·4–373·3)	0·2 (0·1–0·4)	0·3 (0·1–0·6)	48·1 (-49–388·5)	0·2 (0·1–0·4)	0·2 (0·1–0·5)	43·3 (-51·8–384·2)
Andorra	0·1 (0–0·3)	0·2 (0·1–0·4)	22 (-60·6–327·1)	0·2 (0·1–0·5)	0·3 (0·1–0·6)	34·2 (-54·2–341)	0·2 (0·1–0·4)	0·2 (0·1–0·5)	29·2 (-56·7–334·6)
Austria	0·1 (0–0·3)	0·2 (0·1–0·5)	49·7 (-51·7–424·4)	0·2 (0·1–0·4)	0·3 (0·1–0·7)	75·2 (-40·1–476·6)	0·2 (0·1–0·4)	0·3 (0·1–0·6)	65·3 (-44·7–459)
Belgium	0·1 (0–0·3)	0·1 (0–0·3)	4·6 (-66·3–266·2)	0·1 (0·1–0·3)	0·2 (0·1–0·3)	7·2 (-63·4–252·4)	0·1 (0–0·3)	0·1 (0·1–0·3)	6·1 (-64·7–259·1)
Cyprus	0·1 (0–0·3)	0·2 (0·1–0·3)	13·2 (-63·5–296·5)	0·2 (0·1–0·3)	0·2 (0·1–0·4)	16 (-60·4–281·4)	0·1 (0·1–0·3)	0·2 (0·1–0·4)	14·7 (-61·8–287·7)
Denmark	0·2 (0·1–0·4)	0·3 (0·1–0·6)	61·2 (-47·9–464·9)	0·3 (0·1–0·5)	0·4 (0·2–0·9)	72·2 (-41·1–466·4)	0·2 (0·1–0·4)	0·3 (0·1–0·7)	70·3 (-43–474·3)
Finland	0·1 (0–0·3)	0·2 (0·1–0·4)	48·9 (-52–421·5)	0·3 (0·1–0·5)	0·7 (0·3–1·3)	151·8 (-13·5–732·4)	0·2 (0·1–0·4)	0·4 (0·2–0·9)	119·6 (-25·9–641·9)
France	0·1 (0–0·3)	0·2 (0·1–0·3)	24 (-60–334·1)	0·2 (0·1–0·4)	0·2 (0·1–0·5)	34 (-54·2–340·9)	0·1 (0·1–0·3)	0·2 (0·1–0·4)	29·9 (-56·7–339·2)
Germany	0·1 (0–0·3)	0·2 (0·1–0·5)	70·1 (-45·1–495·8)	0·2 (0·1–0·3)	0·3 (0·1–0·7)	98·8 (-32·1–554·3)	0·1 (0·1–0·3)	0·3 (0·1–0·6)	86·6 (-37·7–532·3)
Greece	0·1 (0–0·3)	0·2 (0·1–0·3)	17·6 (-62·1–311·9)	0·2 (0·1–0·3)	0·2 (0·1–0·4)	23·8 (-57·7–307·2)	0·1 (0·1–0·3)	0·2 (0·1–0·4)	21·1 (-59·7–310·1)
Iceland	0·2 (0·1–0·5)	0·3 (0·1–0·6)	42·3 (-54–398·3)	0·6 (0·2–1·3)	1·5 (0·6–3)	133·7 (-19·2–675·3)	0·4 (0·2–0·9)	0·9 (0·4–1·9)	115·3 (-26·6–625·6)
Ireland	0·1 (0–0·3)	0·1 (0·1–0·3)	17·6 (-62·1–311·7)	0·2 (0·1–0·3)	0·2 (0·1–0·5)	36·9 (-53·3–350·2)	0·1 (0·1–0·3)	0·2 (0·1–0·4)	28·4 (-57·2–334·4)
Israel	0·1 (0–0·3)	0·2 (0·1–0·4)	22 (-60·6–327·1)	0·2 (0·1–0·5)	0·3 (0·1–0·6)	34·2 (-54·2–341)	0·2 (0·1–0·4)	0·2 (0·1–0·5)	29·9 (-56·6–338·2)
Italy	0·1 (0–0·3)	0·2 (0·1–0·4)	37 (-55·8–380)	0·2 (0·1–0·3)	0·3 (0·1–0·5)	51·8 (-48·1–399·6)	0·1 (0·1–0·3)	0·2 (0·1–0·4)	45·8 (-51·3–393·7)
Luxembourg	0·1 (0–0·3)	0·1 (0–0·3)	4·7 (-66·2–266·5)	0·1 (0·1–0·3)	0·2 (0·1–0·3)	8·1 (-63·1–255·4)	0·1 (0–0·3)	0·1 (0·1–0·3)	6·7 (-64·5–261·4)

	Female			Male			Both		
	Age-standardized prevalence (%)		Change (%)	Age-standardized prevalence (%)		Change (%)	Age-standardized prevalence (%)		Change (%)
	1990	2023		1990	2023		1990	2023	
Malta	0·1 (0–0·3)	0·1 (0·1–0·3)	5 (-66·1–267·7)	0·2 (0·1–0·4)	0·2 (0·1–0·4)	16·1 (-60·4–281·7)	0·1 (0·1–0·3)	0·2 (0·1–0·3)	12·8 (-62·3–281·6)
Monaco	0·1 (0–0·3)	0·2 (0·1–0·4)	22 (-60·6–327·1)	0·2 (0·1–0·5)	0·3 (0·1–0·6)	34·2 (-54·2–341)	0·2 (0·1–0·4)	0·2 (0·1–0·5)	29·8 (-56·6–337·9)
Netherlands	0·1 (0–0·3)	0·2 (0·1–0·3)	23·1 (-60·3–330·9)	0·2 (0·1–0·3)	0·2 (0·1–0·4)	24·8 (-57·4–310·4)	0·1 (0·1–0·3)	0·2 (0·1–0·4)	24·1 (-58·7–319·7)
Norway	0·3 (0·1–0·6)	0·5 (0·2–1)	73·4 (-43·9–508·8)	0·6 (0·2–1·2)	1·4 (0·5–2·8)	140·9 (-16·9–694·3)	0·4 (0·2–0·9)	0·9 (0·4–1·9)	121·2 (-25·1–647·4)
Portugal	0·1 (0–0·3)	0·2 (0·1–0·3)	24·5 (-59·8–336·1)	0·1 (0·1–0·3)	0·2 (0·1–0·4)	34·7 (-54–343)	0·1 (0–0·3)	0·2 (0·1–0·4)	30·1 (-56·7–341)
San Marino	0·1 (0·1–0·3)	0·2 (0·1–0·4)	19·2 (-61·6–317·3)	0·2 (0·1–0·5)	0·3 (0·1–0·6)	30·8 (-55·3–330)	0·2 (0·1–0·4)	0·2 (0·1–0·5)	26·7 (-57·6–326·8)
Spain	0·1 (0–0·3)	0·1 (0–0·3)	4·6 (-66·3–266·2)	0·1 (0·1–0·3)	0·2 (0·1–0·3)	7·4 (-63·4–253)	0·1 (0–0·3)	0·1 (0·1–0·3)	6·2 (-64·7–259·6)
Sweden	0·2 (0·1–0·5)	0·5 (0·2–1)	125·1 (-27·1–690·3)	2 (0·8–4·2)	2·6 (1·1–5·3)	28·9 (-54·5–314·2)	1·1 (0·4–2·4)	1·6 (0·6–3·2)	38·6 (-51·5–350)
Switzerland	0·1 (0·1–0·3)	0·2 (0·1–0·4)	25 (-59·7–337·6)	0·2 (0·1–0·5)	0·4 (0·1–0·8)	54·1 (-47·3–407)	0·2 (0·1–0·4)	0·3 (0·1–0·6)	43·7 (-51·8–384·5)
United Kingdom	0·1 (0–0·3)	0·1 (0–0·3)	4·6 (-66·3–266·2)	0·2 (0·1–0·4)	0·2 (0·1–0·4)	2·3 (-65·1–236·1)	0·1 (0·1–0·3)	0·2 (0·1–0·3)	3·2 (-65·7–248·2)
**Latin America and Caribbean**	0·4 (0·1–0·9)	0·4 (0·1–0·8)	-3·4 (-68·6–239·8)	0·5 (0·2–1·2)	0·6 (0·2–1·2)	2·8 (-64·5–239·3)	0·5 (0·2–1)	0·5 (0·2–1)	0·1 (-66·3–242·3)
Andean Latin America	0·4 (0·1–0·9)	0·3 (0·1–0·7)	-21·7 (-74·6–173·3)	0·5 (0·2–1·1)	0·4 (0·2–0·9)	-12 (-69·8–188·8)	0·5 (0·2–1)	0·4 (0·1–0·8)	-16·3 (-72–182·6)
Bolivia (Plurinational State of)	0·3 (0·1–0·6)	0·3 (0·1–0·5)	-9·7 (-70·8–215·9)	0·4 (0·2–0·9)	0·4 (0·1–0·8)	-10·6 (-69·4–193·6)	0·3 (0·1–0·7)	0·3 (0·1–0·7)	-10 (-69·9–203·3)
Ecuador	0·2 (0·1–0·4)	0·2 (0·1–0·4)	-2·9 (-68·7–239·8)	0·2 (0·1–0·5)	0·2 (0·1–0·4)	-4·1 (-67·3–215·1)	0·2 (0·1–0·4)	0·2 (0·1–0·4)	-3·5 (-67·9–226·7)
Peru	0·6 (0·2–1·2)	0·4 (0·2–0·9)	-24 (-75·4–165·1)	0·7 (0·2–1·4)	0·6 (0·2–1·2)	-11·7 (-69·7–189·1)	0·6 (0·2–1·3)	0·5 (0·2–1·1)	-17·1 (-72·3–179·6)
Caribbean	0·3 (0·1–0·7)	0·3 (0·1–0·7)	4·4 (-66·2–267·6)	0·5 (0·2–1·2)	0·7 (0·3–1·4)	20·8 (-58·6–293·8)	0·4 (0·2–0·9)	0·5 (0·2–1)	15·1 (-61·3–290·2)
Antigua and Barbuda	0·4 (0·2–0·9)	0·4 (0·2–0·9)	-5·5 (-69·4–230·1)	0·8 (0·3–1·6)	0·7 (0·3–1·5)	-6·5 (-67·7–206·1)	0·6 (0·2–1·3)	0·6 (0·2–1·2)	-5·4 (-68·1–217·1)

	Female			Male			Both		
	Age-standardized prevalence (%)		Change (%)	Age-standardized prevalence (%)		Change (%)	Age-standardized prevalence (%)		Change (%)
	1990	2023		1990	2023		1990	2023	
Bahamas	0·3 (0·1–0·6)	0·3 (0·1–0·6)	3 (-66·7–260·5)	0·6 (0·2–1·4)	0·7 (0·3–1·4)	2·2 (-64·8–235·4)	0·4 (0·2–1)	0·5 (0·2–1)	2·2 (-65·5–241·5)
Barbados	0·3 (0·1–0·6)	0·3 (0·1–0·6)	1·4 (-67·2–254·9)	0·3 (0·1–0·7)	0·4 (0·2–0·8)	19·8 (-58·9–294·4)	0·3 (0·1–0·7)	0·3 (0·1–0·7)	11·1 (-62·8–276·4)
Belize	0·3 (0·1–0·7)	0·3 (0·1–0·7)	4·3 (-66·3–264·7)	0·3 (0·1–0·7)	0·3 (0·1–0·7)	-1·7 (-66·4–223·2)	0·3 (0·1–0·7)	0·3 (0·1–0·7)	1·2 (-66·4–242·4)
Bermuda	0·3 (0·1–0·6)	0·3 (0·1–0·6)	3·8 (-66·5–263·1)	0·4 (0·2–0·9)	0·5 (0·2–1)	4·1 (-64·4–241·4)	0·4 (0·1–0·8)	0·4 (0·1–0·8)	4·1 (-65·2–249·9)
Cuba	0·3 (0·1–0·6)	0·3 (0·1–0·6)	1·3 (-67·3–254·6)	0·3 (0·1–0·7)	0·3 (0·1–0·6)	-7·7 (-68·4–203·6)	0·3 (0·1–0·6)	0·3 (0·1–0·6)	-3·3 (-67·8–227·4)
Dominica	0·2 (0·1–0·4)	0·2 (0·1–0·4)	9·5 (-64·7–283·3)	0·4 (0·1–0·8)	0·4 (0·2–0·8)	2·7 (-64·9–237·1)	0·3 (0·1–0·6)	0·3 (0·1–0·6)	4·1 (-65–248)
Dominican Republic	0·3 (0·1–0·7)	0·3 (0·1–0·7)	-1·8 (-68·3–246·5)	1·2 (0·4–2·4)	1·4 (0·6–2·8)	23·1 (-57·1–312·2)	0·7 (0·3–1·5)	0·9 (0·4–1·7)	19·3 (-59·6–304·8)
Grenada	0·4 (0·1–0·9)	0·4 (0·2–0·9)	-0·2 (-67·7–248·9)	0·6 (0·2–1·4)	0·6 (0·3–1·3)	-0·2 (-65·8–227·2)	0·5 (0·2–1·1)	0·5 (0·2–1·1)	0·8 (-66·2–238·9)
Guyana	0·2 (0·1–0·5)	0·2 (0·1–0·5)	-1 (-68–247·2)	0·3 (0·1–0·7)	0·3 (0·1–0·7)	-4·9 (-67·4–214·7)	0·3 (0·1–0·6)	0·3 (0·1–0·6)	-3·2 (-67·7–227·9)
Haiti	0·4 (0·1–0·9)	0·4 (0·2–1)	5·8 (-65·7–275·3)	0·5 (0·2–1·2)	0·5 (0·2–1·1)	0·6 (-65·5–231·8)	0·5 (0·2–1)	0·5 (0·2–1)	2·5 (-65·7–248·8)
Jamaica	0·3 (0·1–0·7)	0·3 (0·1–0·7)	6·4 (-65·6–272·1)	0·5 (0·2–1·1)	0·6 (0·2–1·2)	8·6 (-62·7–256·2)	0·4 (0·2–0·9)	0·4 (0·2–0·9)	8·5 (-63·6–264·5)
Puerto Rico	0·3 (0·1–0·6)	0·3 (0·1–0·5)	-8·4 (-70·4–220·7)	0·4 (0·1–0·8)	0·4 (0·2–0·8)	6·7 (-63·5–252·1)	0·3 (0·1–0·7)	0·3 (0·1–0·7)	-0·3 (-66·7–238)
Saint Kitts and Nevis	0·2 (0·1–0·4)	0·2 (0·1–0·4)	9·8 (-64·6–284·4)	0·3 (0·1–0·5)	0·3 (0·1–0·6)	14·6 (-60·9–276·4)	0·2 (0·1–0·5)	0·2 (0·1–0·5)	11·8 (-62·8–277·3)
Saint Lucia	0·3 (0·1–0·7)	0·3 (0·1–0·6)	-9·9 (-70·9–215·2)	0·4 (0·2–0·9)	0·4 (0·2–0·8)	-13 (-70·2–186·2)	0·4 (0·1–0·8)	0·3 (0·1–0·7)	-11·2 (-70·3–199·9)
Saint Vincent and the Grenadines	0·2 (0·1–0·5)	0·2 (0·1–0·5)	1·8 (-67·1–256·4)	0·6 (0·2–1·3)	0·6 (0·3–1·3)	1·3 (-65·2–232·9)	0·4 (0·2–0·9)	0·4 (0·2–0·9)	2·3 (-65·4–241·8)
Suriname	0·4 (0·1–0·8)	0·3 (0·1–0·7)	-5·3 (-69·4–230·9)	0·8 (0·3–1·6)	0·7 (0·3–1·5)	-3·8 (-66·8–215)	0·6 (0·2–1·2)	0·5 (0·2–1·1)	-5·1 (-67·9–216·9)
Trinidad and Tobago	0·5 (0·2–1·2)	0·5 (0·2–1·2)	-1·8 (-68·2–243·2)	0·8 (0·3–1·7)	0·8 (0·3–1·7)	0 (-65·4–228·5)	0·7 (0·2–1·4)	0·7 (0·3–1·4)	-0·6 (-66·5–234·6)
United States Virgin Islands	0·2 (0·1–0·4)	0·2 (0·1–0·5)	23·9 (-60–334·4)	0·3 (0·1–0·6)	0·4 (0·2–0·9)	43·9 (-50·6–377·3)	0·2 (0·1–0·5)	0·3 (0·1–0·7)	37·1 (-54·1–365·8)
Central Latin America	0·4 (0·1–0·9)	0·4 (0·1–0·8)	-1·7 (-68·2–243·5)	0·6 (0·2–1·2)	0·5 (0·2–1·1)	-3·8 (-66·9–214·9)	0·5 (0·2–1)	0·5 (0·2–1)	-3·1 (-67·5–226)

	Female			Male			Both		
	Age-standardized prevalence (%)		Change (%)	Age-standardized prevalence (%)		Change (%)	Age-standardized prevalence (%)		Change (%)
	1990	2023		1990	2023		1990	2023	
Colombia	0·7 (0·2–1·4)	0·7 (0·3–1·4)	3·1 (-66·4–260·5)	0·7 (0·2–1·4)	0·7 (0·3–1·4)	2·3 (-64·8–235·7)	0·7 (0·2–1·4)	0·7 (0·3–1·4)	2·5 (-65·7–247·1)
Costa Rica	0·2 (0·1–0·4)	0·2 (0·1–0·4)	-2·4 (-68·5–241·6)	0·2 (0·1–0·5)	0·2 (0·1–0·5)	-1·5 (-66·4–223·6)	0·2 (0·1–0·5)	0·2 (0·1–0·5)	-1·7 (-67·3–232·2)
El Salvador	0·5 (0·2–1·1)	0·5 (0·2–1)	-7·4 (-70–223·3)	0·6 (0·2–1·2)	0·5 (0·2–1·1)	-6·5 (-68–206·4)	0·5 (0·2–1·2)	0·5 (0·2–1·1)	-7·4 (-69·1–212·9)
Guatemala	0·3 (0·1–0·6)	0·3 (0·1–0·6)	0·3 (-67·6–251·1)	0·3 (0·1–0·6)	0·3 (0·1–0·6)	1·8 (-65·2–234·6)	0·3 (0·1–0·6)	0·3 (0·1–0·6)	1·2 (-66·4–242·9)
Honduras	0·3 (0·1–0·6)	0·3 (0·1–0·6)	0·4 (-67·6–251·8)	0·4 (0·2–0·9)	0·4 (0·2–0·9)	1·1 (-65·4–233·4)	0·3 (0·1–0·7)	0·3 (0·1–0·7)	-0·1 (-66·6–237·1)
Mexico	0·2 (0·1–0·5)	0·2 (0·1–0·5)	-1·1 (-68·1–246)	0·3 (0·1–0·6)	0·3 (0·1–0·6)	5·5 (-64–246·5)	0·3 (0·1–0·6)	0·3 (0·1–0·6)	2·5 (-65·9–246·6)
Nicaragua	0·5 (0·2–1·1)	0·5 (0·2–1·1)	1·3 (-67·2–254·1)	0·6 (0·2–1·3)	0·6 (0·2–1·3)	0·8 (-65·4–230·5)	0·6 (0·2–1·2)	0·6 (0·2–1·2)	1·3 (-66·1–242·2)
Panama	0·5 (0·2–1·1)	0·5 (0·2–1·1)	1·9 (-67–256·9)	0·5 (0·2–1·1)	0·5 (0·2–1)	1·2 (-65·4–232·5)	0·5 (0·2–1·1)	0·5 (0·2–1·1)	1·5 (-66·2–244)
Venezuela (Bolivarian Republic of)	0·7 (0·2–1·5)	0·7 (0·3–1·5)	-2·9 (-68·5–238·7)	1·7 (0·6–3·5)	1·6 (0·6–3·2)	-7·2 (-67·6–203·1)	1·2 (0·4–2·5)	1·1 (0·4–2·3)	-7·3 (-68·4–208·6)
Tropical Latin America	0·4 (0·1–0·9)	0·4 (0·2–0·9)	-3·8 (-68·6–242·4)	0·6 (0·2–1·2)	0·6 (0·2–1·3)	8·9 (-62·4–263)	0·5 (0·2–1)	0·5 (0·2–1·1)	3·4 (-65·1–253·6)
Brazil	0·4 (0·1–0·9)	0·4 (0·1–0·8)	-4·6 (-69·1–237·3)	0·5 (0·2–1·1)	0·6 (0·2–1·2)	8·9 (-62·8–260·2)	0·5 (0·2–1)	0·5 (0·2–1)	3 (-65·7–249·4)
Paraguay	1·2 (0·4–2·6)	1·2 (0·4–2·5)	-1·7 (-67·8–242·5)	1·8 (0·7–3·8)	1·8 (0·7–3·7)	-0·9 (-65·4–223·4)	1·5 (0·6–3·2)	1·5 (0·6–3·1)	-1·4 (-66·5–230·1)
North Africa and Middle East	0·6 (0·2–1·3)	0·5 (0·2–1·1)	-13 (-67·9–173·4)	1·6 (0·7–3·1)	1·4 (0·7–2·6)	-8·9 (-62·2–156·9)	1·1 (0·5–2·2)	1 (0·5–1·9)	-9·3 (-63·2–163·7)
Afghanistan	0·3 (0·1–0·7)	0·3 (0·1–0·7)	-8·7 (-70·5–220·2)	2·4 (0·9–5)	2·7 (1·2–5·2)	14·5 (-59–268·1)	1·2 (0·5–2·5)	1·5 (0·7–2·9)	25·3 (-56–316)
Algeria	0·5 (0·2–1·1)	0·5 (0·2–1·1)	-0·5 (-67·8–248·8)	2·8 (1·1–5·7)	2·7 (1·1–5·3)	-3·4 (-65·8–212·7)	1·6 (0·6–3·4)	1·6 (0·7–3·2)	-2·2 (-66–217)
Bahrain	0·5 (0·2–1·2)	0·5 (0·2–1)	-11·3 (-71·3–209·8)	1·1 (0·4–2·3)	1 (0·4–2)	-10·7 (-69·2–191·8)	0·9 (0·3–1·8)	0·8 (0·3–1·6)	-8·4 (-68·9–204·7)
Egypt	0·2 (0·1–0·5)	0·2 (0·1–0·5)	-5·3 (-69·4–231·7)	0·7 (0·2–1·4)	0·6 (0·2–1·3)	-8 (-68·4–201·7)	0·5 (0·2–1)	0·4 (0·2–0·9)	-7·3 (-68·7–208·9)
Iran (Islamic Republic of)	0·5 (0·2–1)	0·4 (0·2–0·9)	-13·4 (-72–202·5)	1 (0·4–2·1)	0·9 (0·4–1·8)	-12·4 (-69·8–186·4)	0·8 (0·3–1·6)	0·7 (0·3–1·4)	-12·9 (-70·6–190·6)
Iraq	0·3 (0·1–0·7)	0·3 (0·1–0·6)	-11 (-71·2–211·6)	0·6 (0·2–1·2)	0·5 (0·2–1·1)	-8·8 (-68·7–199·6)	0·5 (0·2–1)	0·4 (0·2–0·9)	-9·7 (-69·7–203·2)
Jordan	0·3 (0·1–0·6)	0·2 (0·1–0·5)	-21·4 (-74·6–174·7)	0·5 (0·2–1·1)	0·4 (0·1–0·8)	-26·8 (-74·9–140·5)	0·4 (0·1–0·9)	0·3 (0·1–0·6)	-24·5 (-74·7–153)

	Female			Male			Both		
	Age-standardized prevalence (%)		Change (%)	Age-standardized prevalence (%)		Change (%)	Age-standardized prevalence (%)		Change (%)
	1990	2023		1990	2023		1990	2023	
Kuwait	0·3 (0·1–0·7)	0·3 (0·1–0·6)	-4·3 (-69·1–234·8)	0·6 (0·2–1·2)	0·6 (0·2–1·2)	-4·8 (-67·3–215·7)	0·5 (0·2–1)	0·5 (0·2–1)	-3 (-67·2–226·8)
Lebanon	0·6 (0·2–1·3)	0·6 (0·2–1·2)	-2·8 (-68·5–239·7)	0·8 (0·3–1·8)	0·8 (0·3–1·6)	-8·5 (-68·5–201·1)	0·7 (0·3–1·5)	0·7 (0·3–1·4)	-6·6 (-68·7–215·1)
Libya	0·4 (0·1–0·8)	0·3 (0·1–0·7)	-5·1 (-69·3–231·6)	0·8 (0·3–1·7)	0·8 (0·3–1·6)	-3·5 (-66·8–216·3)	0·6 (0·2–1·3)	0·6 (0·2–1·2)	-5·8 (-68·2–213·9)
Morocco	0·3 (0·1–0·7)	0·3 (0·1–0·6)	-6·7 (-69·9–226·6)	0·7 (0·3–1·6)	0·8 (0·3–1·6)	5·9 (-62·8–249)	0·5 (0·2–1·1)	0·5 (0·2–1·1)	3·4 (-64·5–245·1)
Oman	0·6 (0·2–1·3)	0·6 (0·2–1·2)	0·2 (-67·5–250)	0·9 (0·3–1·9)	0·8 (0·3–1·7)	-8·7 (-68·6–199·3)	0·8 (0·3–1·6)	0·7 (0·3–1·5)	-6·6 (-68·4–211·4)
Palestine	0·4 (0·1–0·9)	0·3 (0·1–0·7)	-16·7 (-73·1–191·6)	1·2 (0·4–2·4)	1·1 (0·4–2·2)	-8·6 (-68·4–198·7)	0·8 (0·3–1·6)	0·7 (0·3–1·5)	-9·1 (-69·1–202·5)
Qatar	0·3 (0·1–0·7)	0·3 (0·1–0·6)	-6·4 (-69·7–228)	1·1 (0·4–2·2)	1 (0·4–2·1)	-2·9 (-66·4–217)	0·8 (0·3–1·7)	0·8 (0·3–1·6)	-6·3 (-67·8–208·2)
Saudi Arabia	0·5 (0·2–1)	0·5 (0·2–1·1)	10·2 (-64·3–285·2)	0·7 (0·3–1·4)	0·7 (0·3–1·4)	2·4 (-64·8–235·9)	0·6 (0·2–1·2)	0·6 (0·2–1·3)	5·8 (-64·3–253·8)
Sudan	0·3 (0·1–0·6)	0·3 (0·1–0·6)	-9·7 (-70·8–216·1)	2·9 (1·1–6)	3·3 (1·5–6·3)	13·9 (-59–270·5)	1·6 (0·6–3·3)	1·8 (0·8–3·5)	14·3 (-59·4–274·4)
Syrian Arab Republic	0·3 (0·1–0·6)	0·3 (0·1–0·7)	34·9 (-56·4–372·8)	0·3 (0·1–0·5)	0·4 (0·2–0·8)	61·6 (-44·7–431·9)	0·3 (0·1–0·5)	0·4 (0·1–0·8)	47·2 (-50·9–399·4)
Tunisia	1 (0·4–2·2)	0·6 (0·2–1·3)	-39·3 (-80–108·6)	2·1 (0·8–4·3)	1·4 (0·5–2·8)	-34·1 (-76·7–111·8)	1·6 (0·6–3·3)	1 (0·4–2)	-36·5 (-78·1–108·4)
Türkiye	0·6 (0·2–1·3)	0·6 (0·2–1·3)	3·2 (-66·6–260·5)	0·7 (0·3–1·5)	0·7 (0·3–1·4)	-5·4 (-67·5–210·1)	0·7 (0·2–1·4)	0·6 (0·2–1·4)	-1·6 (-67·1–231·5)
United Arab Emirates	0·3 (0·1–0·6)	0·2 (0·1–0·5)	-11·5 (-71·4–209·7)	0·4 (0·2–0·9)	0·4 (0·1–0·8)	-14·5 (-70·8–180·8)	0·4 (0·1–0·8)	0·3 (0·1–0·7)	-13·5 (-70·8–188·2)
Yemen	6·8 (3·1–12·7)	4·2 (2·6–6·9)	-39·1 (-71·1–61·3)	17 (9–28·3)	10·3 (7·1–14·3)	-39·6 (-67·1–23·6)	11·9 (6–20·5)	7·2 (4·9–10·5)	-39·6 (-68·1–31·8)
South Asia	13·3 (6·7–22·8)	9·7 (5·6–15·7)	-27·4 (-67·1–68·2)	26·4 (14·9–40·8)	19·5 (14·6–26·2)	-26·3 (-57·2–39·4)	20·1 (10·9–32·2)	14·6 (10·2–20·9)	-27·3 (-60·6–43·6)
Bangladesh	25·7 (13·9–40·4)	26·3 (17·7–36·9)	2·5 (-42·8–100·5)	20·4 (10·7–33·4)	20·9 (17·1–25·7)	2·5 (-40·3–96·2)	22·8 (11·9–36·6)	23·4 (18·3–30·1)	2·8 (-40·5–97·8)
Bhutan	10·4 (4·4–20·2)	9·3 (5·1–15·1)	-11·1 (-61·4–149·4)	21·4 (10·4–37·1)	20·4 (12·8–30·7)	-4·4 (-53·2–116·1)	16·3 (7·6–29·2)	15·1 (9·3–23·2)	-7·5 (-56·3–119·3)

	Female			Male			Both		
	Age-standardized prevalence (%)		Change (%)	Age-standardized prevalence (%)		Change (%)	Age-standardized prevalence (%)		Change (%)
	1990	2023		1990	2023		1990	2023	
India	13·1 (6·5–22·6)	8·5 (4·7–14·3)	-35·1 (-72·1–58·8)	28·4 (16·2–43·3)	19·8 (14·7–26·5)	-30·3 (-59·7–28·9)	21·1 (11·5–33·5)	14·2 (9·8–20·4)	-32·4 (-63·3–33·4)
Nepal	7·3 (3·2–14)	5·7 (3–9·6)	-21·9 (-66·1–112·6)	35·3 (20·5–52·2)	30·6 (22·4–40·2)	-13·3 (-46·9–55·6)	21·2 (12–32·6)	17·5 (12·4–23·9)	-17·2 (-50·9–52·9)
Pakistan	4·9 (1·9–10)	4·8 (2–9·5)	-2·7 (-65·2–223·7)	14·1 (6·6–25·6)	13·6 (7·7–23·1)	-3·5 (-58·5–163·8)	9·7 (4·3–18·3)	9·3 (5–16·1)	-4·3 (-61·1–170·3)
**Southeast Asia, East Asia, and Oceania**	1 (0·4–2·1)	1 (0·5–2·1)	2·6 (-61·4–221·1)	1·3 (0·5–2·6)	1·7 (0·7–3·1)	23·2 (-54·1–273)	1·2 (0·5–2·4)	1·4 (0·6–2·6)	14·4 (-56·7–247·9)
East Asia	0·4 (0·1–0·9)	0·6 (0·2–1·2)	41·2 (-54·2–395)	0·9 (0·3–1·8)	1·1 (0·4–2·3)	28·9 (-55·6–323·5)	0·6 (0·2–1·4)	0·8 (0·3–1·8)	32·6 (-55·2–343·9)
China	0·4 (0·1–0·9)	0·6 (0·2–1·2)	42·5 (-53·8–399·3)	0·9 (0·3–1·8)	1·1 (0·4–2·3)	29·7 (-55·3–326·5)	0·6 (0·2–1·4)	0·9 (0·3–1·8)	33·5 (-54·9–347·1)
Democratic People's Republic of Korea	0·2 (0·1–0·4)	0·2 (0·1–0·4)	0·1 (-67·7–250·2)	0·2 (0·1–0·4)	0·2 (0·1–0·4)	-0·6 (-66·1–226·6)	0·2 (0·1–0·4)	0·2 (0·1–0·4)	-0·3 (-66·9–238·6)
Taiwan	0·5 (0·2–1)	0·5 (0·2–1·1)	9·3 (-64·6–281·9)	0·7 (0·3–1·6)	0·8 (0·3–1·6)	3·9 (-64·3–240·3)	0·6 (0·2–1·3)	0·6 (0·2–1·3)	5·4 (-64·6–253·2)
Oceania	6·9 (3–14·6)	9·9 (5·5–17·5)	43·3 (-44·7–308·1)	5·3 (2·4–11)	5·5 (2·6–10·8)	3 (-61–194·1)	6·1 (2·7–12·9)	7·7 (4·1–14·1)	26 (-50·7–253·7)
American Samoa	2·2 (0·8–4·7)	2·3 (0·9–4·7)	2·6 (-65·6–253·8)	4·2 (1·6–8·4)	4·2 (1·7–8·3)	-0·3 (-63·5–216·4)	3·2 (1·2–6·6)	3·2 (1·3–6·5)	0·8 (-64·2–230)
Cook Islands	2·3 (0·8–4·9)	2·2 (0·9–4·7)	-4 (-67·9–229·8)	3·6 (1·4–7·4)	3·7 (1·5–7·4)	1·6 (-63·3–223·9)	3 (1·1–6·2)	2·9 (1·2–5·9)	-2·5 (-65·8–220·1)
Fiji	1·8 (0·6–3·8)	1·8 (0·7–3·9)	3 (-65·9–257)	2·3 (0·9–4·8)	2·2 (0·9–4·5)	-3·5 (-65·9–213·9)	2 (0·8–4·3)	2 (0·8–4·2)	-0·6 (-65·9–232·6)
Guam	2·6 (1–5·6)	3·6 (1·8–6·4)	39·5 (-48·6–346·3)	7·5 (3·3–14·3)	9·1 (5·4–14·5)	21·7 (-45·7–203·4)	5·2 (2·3–10·2)	6·4 (3·8–10·5)	22·7 (-47·3–220·8)
Kiribati	3·3 (1·3–7)	5·9 (3–10·4)	76·9 (-33·7–455·2)	9 (3·9–17·1)	12·2 (6·6–20·1)	35 (-42–259·3)	6·1 (2·6–12)	9 (4·8–15·2)	47·4 (-39·1–311·6)
Marshall Islands	3·9 (1·6–8)	7·2 (3·9–12·2)	84·7 (-26–447·8)	9·7 (4·7–17·3)	12·5 (7·1–19·8)	28·6 (-43·3–223)	6·9 (3·1–12·7)	9·9 (5·5–15·8)	44·2 (-38·1–278·7)
Micronesia (Federated States of)	4 (1·5–8·1)	4·1 (1·8–8·1)	2·4 (-63·5–225·8)	6·9 (2·9–13·1)	6·9 (3·2–12·8)	0·2 (-60·7–184·8)	5·5 (2·2–10·7)	5·6 (2·5–10·5)	1·3 (-61·6–199·9)
Nauru	0·6 (0·2–1·4)	0·6 (0·2–1·3)	0·9 (-67·2–255)	0·7 (0·3–1·5)	0·7 (0·3–1·5)	1·1 (-65·2–235·6)	0·7 (0·2–1·4)	0·7 (0·3–1·4)	1·5 (-66–246·2)

	Female			Male			Both		
	Age-standardized prevalence (%)		Change (%)	Age-standardized prevalence (%)		Change (%)	Age-standardized prevalence (%)		Change (%)
	1990	2023		1990	2023		1990	2023	
Niue	1·4 (0·5–3)	1·3 (0·5–2·8)	-3·5 (-68·3–237·7)	1·5 (0·6–3·2)	1·8 (0·8–3·5)	15·9 (-58·9–272·1)	1·5 (0·5–3·1)	1·5 (0·6–3·2)	5·2 (-63·9–250·8)
Northern Mariana Islands	3·9 (1·5–7·9)	4·1 (1·8–8)	6·3 (-62·1–240·8)	6·8 (2·8–12·9)	6·9 (3·2–12·8)	2·7 (-59·6–195·8)	5·3 (2·2–10·5)	5·6 (2·5–10·5)	4·5 (-60·4–212·4)
Palau	18·8 (8·3–34·2)	25·5 (13·8–40·8)	35·9 (-40–261·4)	19·1 (8·7–33·9)	23·6 (13·5–36·9)	24 (-43·9–218·2)	18·9 (8·5–34·1)	24·4 (13·6–38·5)	29·1 (-42·2–238·9)
Papua New Guinea	10 (4·1–19·6)	12·5 (6·7–20·9)	24·9 (-50·4–261·5)	6·6 (2·8–12·7)	6·1 (2·7–11·6)	-6·8 (-64·2–173)	8·3 (3·4–16·2)	9·3 (4·8–16·2)	12·8 (-54·3–223·8)
Samoa	0·9 (0·3–2)	1 (0·4–2)	3·7 (-66·2–266·1)	1·4 (0·5–2·9)	1·4 (0·6–2·9)	5·2 (-63·5–245·2)	1·2 (0·4–2·5)	1·2 (0·5–2·5)	3·4 (-65–249·5)
Solomon Islands	2·8 (1–5·9)	2·8 (1·1–5·8)	1·2 (-66–246·8)	3·8 (1·5–7·7)	3·8 (1·6–7·6)	0·4 (-63·9–220)	3·3 (1·2–6·8)	3·3 (1·3–6·7)	0·4 (-64·9–229·5)
Tokelau	1·7 (0·6–3·7)	1·8 (0·7–3·8)	5·3 (-65·2–265·8)	2·3 (0·9–4·8)	2·4 (1–5)	6·3 (-62·2–243·9)	2 (0·7–4·2)	2·1 (0·8–4·4)	6·6 (-63·3–256)
Tonga	0·5 (0·2–1·2)	0·5 (0·2–1)	-13·9 (-72·1–200·7)	0·8 (0·3–1·6)	0·7 (0·3–1·4)	-15·6 (-71–176·4)	0·7 (0·2–1·4)	0·5 (0·2–1·1)	-16·1 (-71·9–181·8)
Tuvalu	0·6 (0·2–1·4)	0·5 (0·2–1·2)	-11·8 (-71·4–208)	0·9 (0·3–1·8)	0·7 (0·3–1·5)	-13·8 (-70·3–182·5)	0·7 (0·3–1·6)	0·6 (0·2–1·4)	-11·7 (-70·4–198)
Vanuatu	2·3 (0·8–4·9)	2·4 (0·9–5)	3·9 (-65·1–259·1)	4·1 (1·6–8·3)	4·1 (1·7–8·2)	1·1 (-63·1–219·3)	3·2 (1·2–6·6)	3·2 (1·3–6·6)	1·6 (-64–231·6)
Southeast Asia	2·9 (1·2–5·7)	2·1 (1–4·1)	-24·7 (-69·7–125·7)	2·8 (1·2–5·4)	2·8 (1·3–4·9)	-3 (-61·3–170·7)	2·9 (1·2–5·6)	2·5 (1·2–4·6)	-13·6 (-65·7–150·8)
Cambodia	13·3 (7–21·8)	8·9 (5·2–14·2)	-33·3 (-66·1–51)	2·1 (0·8–4·3)	1·8 (0·8–3·5)	-13·5 (-69·8–179·8)	8·4 (4·4–14·1)	5·8 (4–8·4)	-30·9 (-61·6–42·6)
Indonesia	2·3 (0·9–4·6)	1·2 (0·5–2·4)	-48·8 (-82·1–61·6)	0·4 (0·1–0·8)	0·5 (0·2–1·1)	33 (-54·4–336·2)	1·4 (0·5–2·8)	0·9 (0·3–1·8)	-37·4 (-78·1–98·3)
Lao People's Democratic Republic	6 (2·4–12·2)	6·3 (3·1–11·4)	4·3 (-62·3–222·1)	0·6 (0·2–1·2)	0·6 (0·2–1·1)	-3·3 (-66·9–217·8)	3·4 (1·4–6·9)	3·4 (1·7–6·2)	0·6 (-63·7–211·7)
Malaysia	1·5 (0·5–3·1)	1·7 (0·6–3·5)	14 (-62·3–294·5)	2 (0·8–4·1)	2·9 (1·2–5·8)	45·5 (-48·8–368·9)	1·7 (0·6–3·6)	2·3 (1–4·7)	34·1 (-53·8–342·5)
Maldives	3·4 (1·3–7·1)	3·1 (1·3–6·1)	-7·5 (-68·3–204·3)	5 (2·2–9·7)	5·2 (2·6–9·3)	3·8 (-60·7–203·9)	4·2 (1·8–8·5)	4·4 (2·1–8·1)	4 (-61·5–211·1)
Mauritius	2·9 (1·1–6·1)	2·4 (0·9–4·9)	-18·5 (-72–176·5)	1·5 (0·6–3·2)	1·6 (0·6–3·2)	2·4 (-64·4–234·1)	2·3 (0·8–4·8)	2 (0·8–4·2)	-12·4 (-69·7–193·1)
Myanmar	8·1 (3·1–15·9)	7·6 (3·2–14·5)	-5·3 (-65·7–194·8)	17·6 (8·3–31·2)	18·8 (9·6–31)	7 (-54·4–176·5)	12·8 (5·7–23·6)	13·1 (6·4–22·4)	2·4 (-58·3–174·7)

	Female			Male			Both		
	Age-standardized prevalence (%)		Change (%)	Age-standardized prevalence (%)		Change (%)	Age-standardized prevalence (%)		Change (%)
	1990	2023		1990	2023		1990	2023	
Philippines	1·3 (0·5–2·8)	1 (0·4–2·2)	-20·5 (-74–178·2)	2·6 (1–5·2)	2·6 (1·1–5·2)	2·2 (-63·6–231·5)	1·9 (0·7–4)	1·8 (0·8–3·6)	-5·8 (-67·7–208)
Seychelles	1·9 (0·7–4·1)	1·7 (0·6–3·5)	-14·2 (-71·1–194·8)	1·5 (0·6–3·1)	1·4 (0·6–3)	-3·9 (-66·6–214·2)	1·8 (0·6–3·8)	1·6 (0·6–3·3)	-10·6 (-69·4–200·8)
Sri Lanka	4·6 (1·8–9·5)	4·3 (1·7–8·6)	-7·5 (-68–201·1)	11·9 (5·2–22·4)	12·4 (5·8–21·9)	4·6 (-59·2–196)	8·3 (3·5–15·8)	8·2 (3·7–14·9)	-0·9 (-62·4–188·8)
Thailand	2·7 (1·1–5·6)	2·5 (1–5·1)	-7·9 (-68·3–201)	1·1 (0·4–2·3)	1·2 (0·5–2·4)	6·7 (-63–250·5)	2 (0·8–4·1)	1·9 (0·8–3·9)	-4 (-66·8–213·4)
Timor-Leste	2·5 (0·9–5·2)	2 (0·8–4·1)	-20·3 (-73·2–170·9)	1·2 (0·5–2·6)	1·3 (0·5–2·7)	5·8 (-63·4–250·2)	1·8 (0·7–3·9)	1·6 (0·6–3·4)	-11·4 (-69·8–196)
Viet Nam	1·2 (0·4–2·6)	1 (0·4–2·1)	-19·2 (-73–176·6)	0·5 (0·2–1)	0·4 (0·2–0·8)	-14·8 (-70·8–179·7)	0·9 (0·3–2)	0·7 (0·3–1·6)	-19·5 (-72·9–172·2)
**Sub-Saharan Africa**	1·1 (0·4–2·2)	1 (0·4–1·9)	-8·5 (-67·6–206·1)	1·4 (0·6–2·8)	1·3 (0·6–2·4)	-7 (-64·1–177·8)	1·2 (0·5–2·5)	1·1 (0·5–2·2)	-8 (-65·8–186·2)
Central Sub-Saharan Africa	1 (0·4–2·1)	0·6 (0·2–1·3)	-34·9 (-78·4–126·7)	0·9 (0·3–1·9)	1·1 (0·4–2·2)	15·4 (-59·5–281)	1 (0·4–2)	0·9 (0·3–1·8)	-11 (-69·4–198·2)
Angola	0·3 (0·1–0·8)	0·3 (0·1–0·7)	-1·6 (-68·2–244·2)	0·6 (0·2–1·2)	0·6 (0·2–1·2)	1 (-65·3–235)	0·5 (0·2–1)	0·5 (0·2–1)	-1·6 (-67–232·3)
Central African Republic	1 (0·3–2·1)	1 (0·4–2)	0·4 (-67·1–252·9)	0·6 (0·2–1·3)	0·6 (0·3–1·3)	1 (-65·3–233·5)	0·8 (0·3–1·7)	0·8 (0·3–1·7)	0·1 (-66·6–244·4)
Congo	0·9 (0·3–1·9)	0·9 (0·3–1·9)	-0·1 (-67·4–250·3)	0·5 (0·2–1)	0·5 (0·2–1)	7 (-63·4–256·1)	0·7 (0·2–1·5)	0·7 (0·3–1·5)	0·3 (-66·7–247)
Democratic Republic of the Congo	1·2 (0·4–2·5)	0·7 (0·3–1·5)	-37·9 (-79·8–114)	1·1 (0·4–2·3)	1·3 (0·6–2·7)	20·7 (-58·1–294·2)	1·1 (0·4–2·4)	1 (0·4–2·1)	-9·9 (-69·5–197·3)
Equatorial Guinea	0·7 (0·2–1·5)	0·6 (0·2–1·2)	-16·2 (-72·8–192·1)	0·6 (0·2–1·3)	0·6 (0·2–1·3)	2·8 (-64·8–237)	0·6 (0·2–1·4)	0·6 (0·2–1·3)	-4·6 (-68·2–224·4)
Gabon	0·4 (0·1–0·8)	0·3 (0·1–0·7)	-6·2 (-69·6–229·2)	0·3 (0·1–0·7)	0·4 (0·2–0·8)	16·6 (-60·1–283·6)	0·4 (0·1–0·8)	0·4 (0·1–0·8)	2·2 (-66–247·7)
Eastern Sub-Saharan Africa	1·4 (0·5–2·9)	1·3 (0·6–2·6)	-1·6 (-62·8–218·8)	2·3 (1–4·4)	2·1 (1·1–3·8)	-6·6 (-62–159·9)	1·8 (0·7–3·7)	1·7 (0·9–3·2)	-4·9 (-62·5–181·9)
Burundi	2·3 (0·8–4·8)	2 (0·8–4·1)	-10·1 (-70·2–209·4)	3·6 (1·4–7·2)	2·6 (1·1–5)	-27·8 (-74·4–127·6)	2·9 (1·1–5·9)	2·3 (1–4·6)	-18·6 (-71·9–165·5)
Comoros	3·8 (1·4–7·8)	2·8 (1·1–5·7)	-25·5 (-74·4–144·7)	5·3 (2·2–10·4)	4·3 (1·9–7·9)	-18·4 (-69·7–144·4)	4·5 (1·8–9)	3·5 (1·5–6·8)	-21 (-71·7–149·1)
Djibouti	1·1 (0·4–2·5)	1·1 (0·4–2·3)	-4 (-68·6–234·2)	1·5 (0·6–3·2)	1·4 (0·6–2·9)	-7·8 (-68–201·2)	1·3 (0·5–2·9)	1·3 (0·5–2·6)	-5·8 (-68–215·7)

	Female			Male			Both		
	Age-standardized prevalence (%)		Change (%)	Age-standardized prevalence (%)		Change (%)	Age-standardized prevalence (%)		Change (%)
	1990	2023		1990	2023		1990	2023	
Eritrea	0·3 (0·1–0·6)	0·3 (0·1–0·7)	3·9 (-66·4–263·7)	3·3 (1·3–6·7)	2·8 (1·1–5·6)	-15·6 (-70·1–171·2)	1·7 (0·6–3·4)	1·5 (0·6–3·1)	-7·9 (-67·6–199·5)
Ethiopia	0·5 (0·2–1)	0·5 (0·2–1)	-3·5 (-68·6–240·7)	2·1 (0·8–4·3)	1·9 (0·8–3·7)	-10·4 (-68·1–195·3)	1·3 (0·5–2·7)	1·2 (0·5–2·4)	-10·6 (-68·2–194·8)
Kenya	1·1 (0·4–2·4)	0·9 (0·3–1·9)	-18·6 (-73·4–186·4)	1·3 (0·5–2·6)	1·2 (0·5–2·5)	-4·9 (-67·2–210·4)	1·2 (0·4–2·5)	1 (0·4–2·2)	-11·4 (-70·2–199·2)
Madagascar	6·9 (2·9–13·7)	8·2 (4·5–13·7)	19·3 (-50·2–240·4)	14·7 (7·2–25·8)	13·7 (7·7–21·9)	-7·4 (-58·5–133·5)	10·8 (5–19·6)	10·9 (6·1–17·7)	0·9 (-56–164·5)
Malawi	1·3 (0·5–2·8)	1 (0·4–2·1)	-24 (-75–163·3)	0·5 (0·2–1·2)	0·5 (0·2–1·1)	-4·7 (-67·3–213·5)	0·9 (0·3–2)	0·8 (0·3–1·6)	-18·1 (-72·7–181·3)
Mozambique	1·6 (0·6–3·4)	0·9 (0·3–1·9)	-41·7 (-80·7–101·4)	1·1 (0·4–2·3)	0·9 (0·4–2)	-13 (-69·8–183·5)	1·3 (0·5–2·9)	0·9 (0·4–1·9)	-30·7 (-76·6–134·3)
Rwanda	2·3 (0·9–4·9)	1·9 (0·8–3·9)	-17·9 (-72·4–175·2)	0·3 (0·1–0·7)	0·3 (0·1–0·6)	-17·6 (-71·8–170·9)	1·4 (0·5–3)	1·2 (0·5–2·4)	-17·8 (-72·2–172·6)
Somalia	1 (0·4–2·2)	1 (0·4–2)	-4·3 (-68·7–233)	1·2 (0·5–2·6)	1·1 (0·4–2·3)	-9·9 (-68·8–194·2)	1·1 (0·4–2·4)	1 (0·4–2·2)	-7·4 (-68·8–210·5)
South Sudan	1·1 (0·4–2·3)	1 (0·4–2·2)	-3·5 (-68·4–236)	1·4 (0·5–3)	1·3 (0·5–2·7)	-7·3 (-67·9–202·5)	1·3 (0·5–2·7)	1·2 (0·5–2·5)	-7 (-68·5–210·5)
Uganda	1·1 (0·4–2·4)	1 (0·4–2·2)	-7·8 (-69·9–221·9)	0·6 (0·2–1·4)	0·7 (0·3–1·5)	8·8 (-62·9–258·7)	0·9 (0·3–1·9)	0·9 (0·4–1·9)	0·1 (-66·8–242·4)
United Republic of Tanzania	0·7 (0·2–1·5)	0·6 (0·2–1·3)	-10·3 (-70·8–213·8)	0·8 (0·3–1·8)	0·8 (0·3–1·6)	-5·9 (-67·7–212·2)	0·7 (0·3–1·6)	0·7 (0·3–1·5)	-7·6 (-69–215·3)
Zambia	0·8 (0·3–1·7)	0·8 (0·3–1·8)	3·6 (-66·2–263·8)	0·6 (0·2–1·2)	0·6 (0·3–1·3)	13·7 (-61–274·4)	0·7 (0·2–1·5)	0·7 (0·3–1·6)	8·9 (-63·7–272·3)
Southern Sub-Saharan Africa	1·3 (0·5–2·7)	1·2 (0·5–2·5)	-5·7 (-68–221)	1·2 (0·5–2·6)	0·9 (0·3–1·8)	-30·4 (-75·7–125·5)	1·3 (0·5–2·7)	1·1 (0·4–2·3)	-15·7 (-71·2–180·8)
Botswana	5·5 (2·2–11·2)	5·5 (2·7–9·9)	-1 (-62·8–205·3)	2·3 (0·9–4·8)	2·1 (0·8–4·3)	-9·3 (-68·2–195·2)	4·1 (1·6–8·3)	3·9 (1·9–7·3)	-3·3 (-63·9–192·9)
Eswatini	0·4 (0·2–1)	0·5 (0·2–1·2)	20·9 (-60·7–328·1)	0·7 (0·3–1·6)	0·7 (0·3–1·5)	-2·6 (-66·5–223·2)	0·6 (0·2–1·3)	0·6 (0·2–1·3)	8·6 (-63·6–270·9)
Lesotho	0·5 (0·2–1·2)	0·5 (0·2–1·1)	-1·2 (-67·7–245)	1·1 (0·4–2·2)	0·9 (0·4–1·8)	-17·4 (-71·5–173·7)	0·8 (0·3–1·6)	0·7 (0·3–1·5)	-4·9 (-68·1–220·9)
Namibia	1 (0·3–2·1)	1 (0·4–2·1)	2 (-66·3–255·9)	0·9 (0·3–1·8)	0·8 (0·3–1·6)	-11 (-69·5–195·8)	0·9 (0·3–2)	0·9 (0·4–1·9)	-2·1 (-67·1–233·7)

	Female			Male			Both		
	Age-standardized prevalence (%)		Change (%)	Age-standardized prevalence (%)		Change (%)	Age-standardized prevalence (%)		Change (%)
	1990	2023		1990	2023		1990	2023	
South Africa	1·3 (0·5–2·8)	1·1 (0·4–2·4)	-14·6 (-71·6–196·7)	1·4 (0·5–3)	0·9 (0·4–1·9)	-35·1 (-77·3–109·1)	1·4 (0·5–3)	1·1 (0·4–2·3)	-23·2 (-73·8–156·6)
Zimbabwe	0·7 (0·2–1·5)	1 (0·4–2)	37·2 (-55–379·7)	0·5 (0·2–1·1)	0·5 (0·2–1)	-7·6 (-68·3–204·3)	0·6 (0·2–1·3)	0·8 (0·3–1·6)	22·4 (-59·2–316·8)
Western Sub-Saharan Africa	0·7 (0·2–1·5)	0·6 (0·2–1·3)	-6·2 (-69–227)	0·8 (0·3–1·6)	0·8 (0·3–1·5)	-3·8 (-66·6–211·1)	0·7 (0·3–1·5)	0·7 (0·3–1·4)	-5·9 (-68–215·1)
Benin	1·1 (0·4–2·3)	1·1 (0·4–2·2)	2·1 (-66·6–252·5)	1·3 (0·5–2·6)	1·4 (0·6–2·8)	12·9 (-61·1–277·8)	1·1 (0·4–2·4)	1·3 (0·5–2·5)	9·7 (-62·9–275·5)
Burkina Faso	2·1 (0·8–4·4)	1·8 (0·7–3·5)	-15·4 (-71·4–187·9)	1·5 (0·6–3·2)	1·2 (0·5–2·4)	-21·2 (-72·6–155·1)	1·8 (0·7–3·9)	1·5 (0·6–3)	-18·7 (-72·3–169·4)
Cabo Verde	0·6 (0·2–1·2)	0·4 (0·2–0·9)	-27·3 (-76·4–154·3)	0·4 (0·1–0·8)	0·3 (0·1–0·7)	-16·8 (-71·6–173·3)	0·5 (0·2–1·1)	0·4 (0·1–0·8)	-24·6 (-75–158·2)
Cameroon	1·2 (0·5–2·6)	1·3 (0·5–2·6)	1·3 (-66·1–250·1)	0·6 (0·2–1·3)	0·6 (0·2–1·3)	-0·2 (-65·9–228·9)	0·9 (0·4–2)	0·9 (0·4–1·9)	0·7 (-65·9–238·2)
Chad	1 (0·4–2·3)	1·1 (0·4–2·3)	4·5 (-65·6–260·3)	1 (0·4–2)	1 (0·4–1·9)	-0·5 (-65·3–222·8)	1 (0·4–2·1)	1 (0·4–2·1)	1·6 (-65·6–240·3)
Côte d'Ivoire	0·5 (0·2–1·1)	0·4 (0·2–0·9)	-16·2 (-72·8–192·5)	0·4 (0·2–0·9)	0·4 (0·2–0·8)	-2·4 (-66·6–220·8)	0·5 (0·2–1)	0·4 (0·2–0·9)	-8·7 (-69·6–209·9)
Gambia	0·6 (0·2–1·4)	0·6 (0·2–1·2)	-12·5 (-71·4–205·2)	0·6 (0·2–1·3)	0·6 (0·2–1·2)	-11 (-69·7–194·5)	0·6 (0·2–1·4)	0·6 (0·2–1·2)	-10·9 (-70·2–203·6)
Ghana	0·5 (0·2–1·1)	0·5 (0·2–1·1)	8·3 (-64·9–280·2)	0·8 (0·3–1·6)	0·7 (0·3–1·5)	-3·2 (-66·9–218·3)	0·6 (0·2–1·3)	0·6 (0·2–1·3)	0·9 (-66·3–241·4)
Guinea	0·5 (0·2–1·1)	0·4 (0·2–0·9)	-11 (-71·1–210·6)	0·8 (0·3–1·7)	0·8 (0·3–1·7)	-0·6 (-65·8–225·6)	0·6 (0·2–1·4)	0·6 (0·2–1·3)	-5·1 (-68·1–218·7)
Guinea-Bissau	0·5 (0·2–1·2)	0·5 (0·2–1)	-9·4 (-70·6–216)	0·5 (0·2–1)	0·4 (0·2–0·9)	-12·7 (-70·1–186·3)	0·5 (0·2–1·1)	0·5 (0·2–1)	-10·1 (-70·1–204·6)
Liberia	0·6 (0·2–1·2)	0·5 (0·2–1·1)	-6·2 (-69·5–227·9)	1 (0·4–2·2)	1·1 (0·4–2·2)	3·9 (-64·4–240·6)	0·8 (0·3–1·7)	0·8 (0·3–1·7)	1·1 (-65·9–240·8)
Mali	0·6 (0·2–1·2)	0·5 (0·2–1·1)	-8·3 (-70·2–220·1)	1·9 (0·7–4)	1·9 (0·8–3·7)	-2·1 (-65·7–219·6)	1·2 (0·5–2·5)	1·2 (0·5–2·4)	-0·9 (-65·5–224·5)
Mauritania	0·9 (0·3–1·9)	0·9 (0·3–1·9)	1·6 (-66·9–254·5)	1·1 (0·4–2·2)	1·1 (0·4–2·3)	4·6 (-63·9–242·7)	1 (0·4–2·1)	1 (0·4–2·1)	2·6 (-65·5–246)
Niger	1 (0·4–2·3)	1·2 (0·5–2·4)	11·5 (-63·7–284·3)	2·5 (1–5·2)	2·6 (1·1–4·9)	2·5 (-63·3–228·3)	1·8 (0·7–3·7)	1·9 (0·8–3·6)	2·7 (-64·3–237·1)
Nigeria	0·4 (0·1–0·9)	0·4 (0·1–0·8)	-4·5 (-69–240·6)	0·5 (0·2–1·1)	0·5 (0·2–0·9)	-12·9 (-70·2–190·5)	0·5 (0·2–1)	0·4 (0·2–0·9)	-13·1 (-70·9–196·9)

	Female			Male			Both		
	Age-standardized prevalence (%)		Change (%)	Age-standardized prevalence (%)		Change (%)	Age-standardized prevalence (%)		Change (%)
	1990	2023		1990	2023		1990	2023	
Sao Tome and Principe	0·5 (0·2–1)	0·4 (0·2–1)	-0·4 (-67·7–249·7)	0·6 (0·2–1·3)	0·7 (0·3–1·5)	17 (-59·7–288·5)	0·5 (0·2–1·1)	0·6 (0·2–1·2)	10·4 (-62·9–276·4)
Senegal	0·8 (0·3–1·7)	0·8 (0·3–1·7)	0·9 (-67·1–252·8)	0·4 (0·1–0·8)	0·3 (0·1–0·6)	-15·7 (-71·1–179)	0·6 (0·2–1·2)	0·6 (0·2–1·2)	-3·5 (-68·1–231·2)
Sierra Leone	1·5 (0·5–3·2)	1·3 (0·5–2·7)	-14·1 (-71·5–197·1)	1·2 (0·4–2·5)	1 (0·4–2·1)	-13·1 (-70–184·2)	1·3 (0·5–2·8)	1·1 (0·5–2·4)	-13·8 (-70·9–190·8)
Togo	1·1 (0·4–2·4)	1 (0·4–2·2)	-9·8 (-70·3–215·7)	1 (0·4–2)	0·9 (0·3–1·8)	-11·4 (-69·4–191·7)	1·1 (0·4–2·3)	1 (0·4–2)	-9·9 (-69·7–207·8)

Appendix Figure 2·3: Prevalence of chewing tobacco use by age and sex, among ages 15+, global and South Asia, 2023

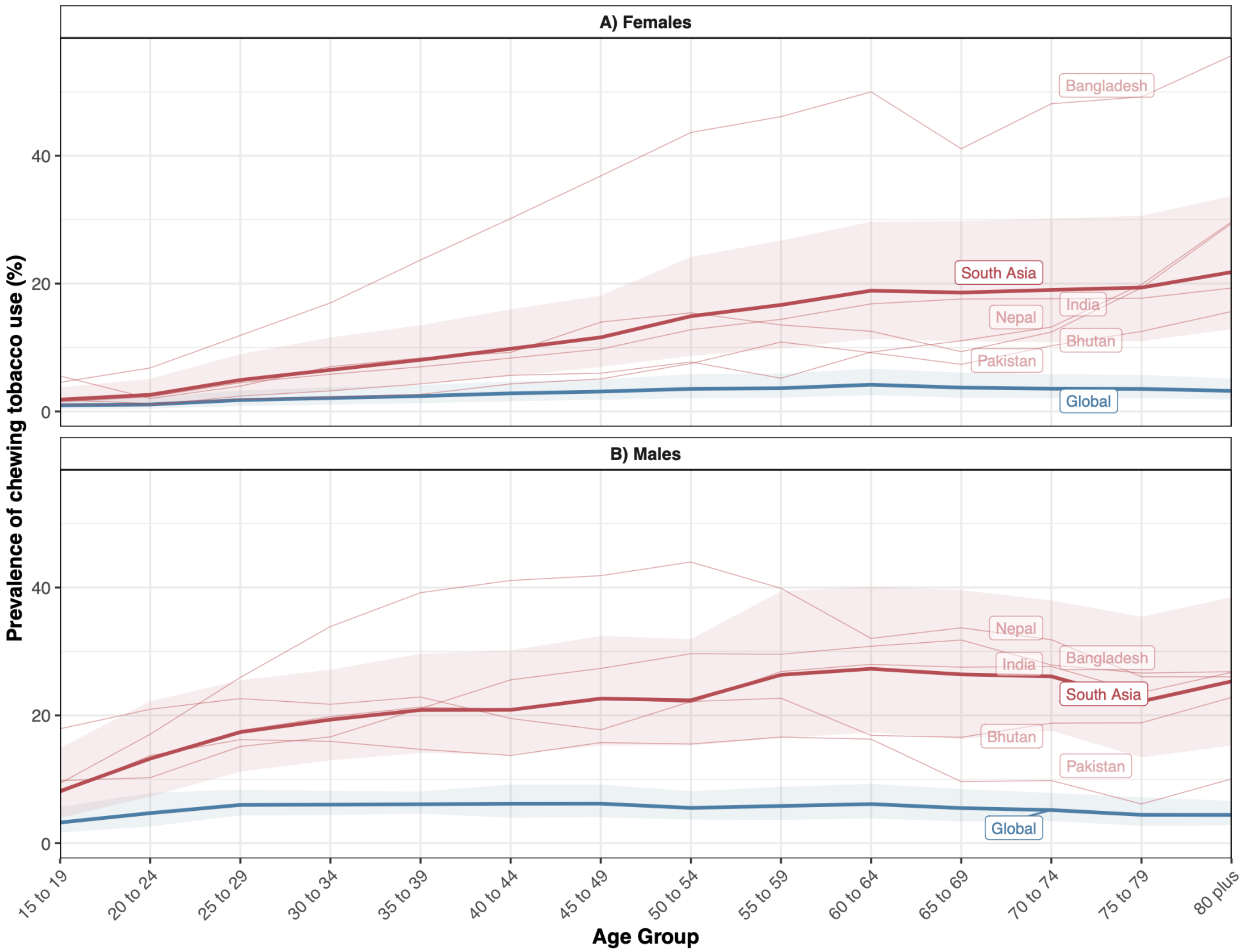

Appendix Figure 2·4: Number of new users of chewing tobacco by region, among ages 15+, comparing 1990 and 2023

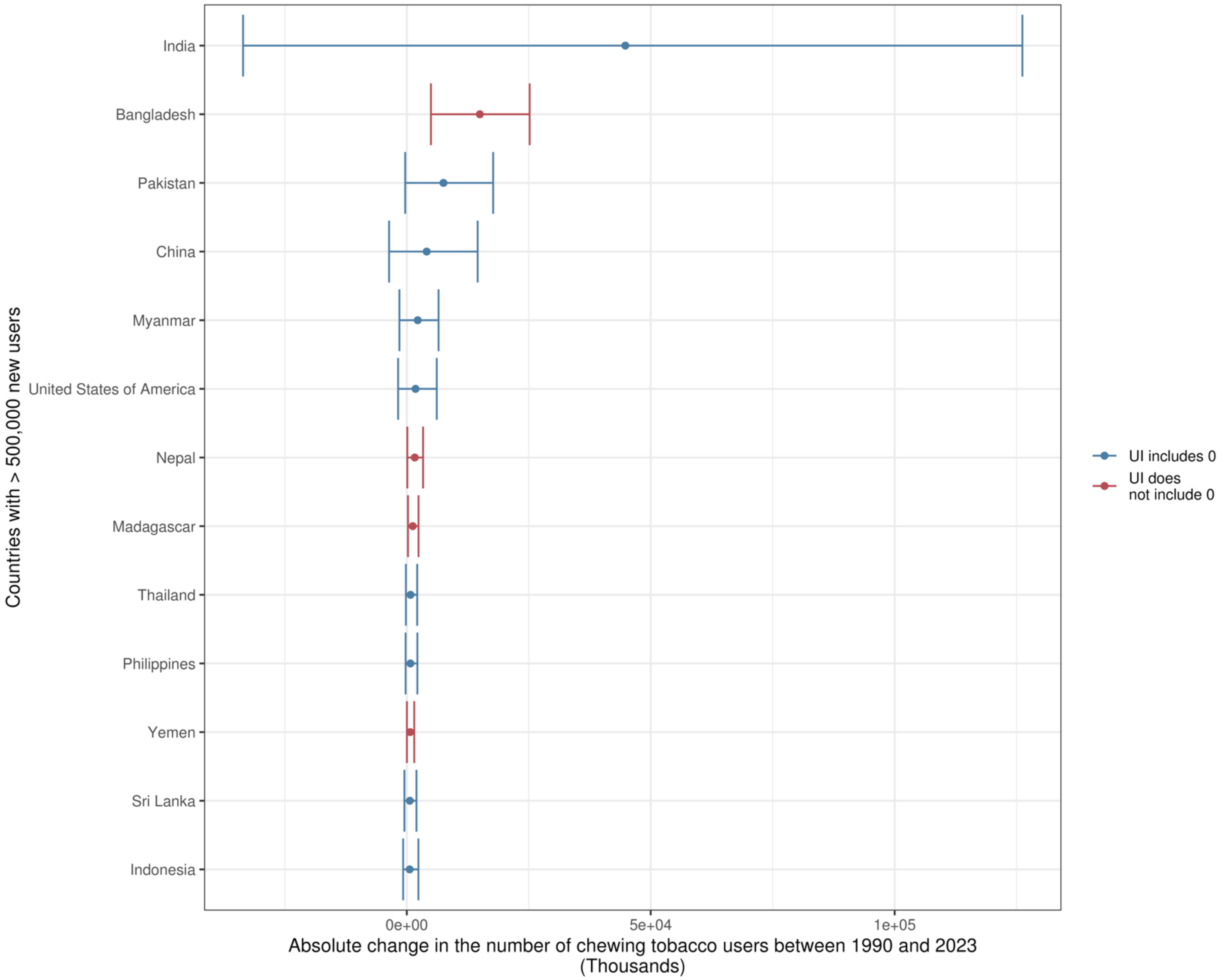

Appendix Table 2·6: Number of deaths attributable to chewing tobacco use in 1990 and 2023, among ages 15+

	Female			Male			Both		
	Number of deaths		Change (%)	Number of deaths		Change (%)	Number of deaths		Change (%)
	1990	2023		1990	2023		1990	2023	
Global	42800 (21800–78600)	99700 (53200–164000)	133 (19·8–317)	64700 (33500–108000)	152000 (96200–235000)	134 (38·7–321)	108000 (55400–177000)	251000 (149000–389000)	134 (31·9–312)
**Central Europe, Eastern Europe, and Central Asia**	550 (174–1300)	450 (140–1070)	-18·1 (-71·2–168)	765 (290–1680)	865 (296–1860)	13 (-59·4–238)	1320 (453–3000)	1320 (434–2920)	0 (-64·2–210)
Central Asia	57·5 (18·7–137)	60 (18·5–140)	4·2 (-64–233)	260 (101–554)	294 (106–622)	12·9 (-56·6–206)	318 (119–685)	354 (124–760)	11·3 (-57·9–210)
Armenia	1·4 (0·447–3·39)	1·26 (0·394–2·88)	-10·6 (-69·7–171)	1·74 (0·642–3·86)	1·54 (0·486–3·5)	-11·6 (-67·7–167)	3·14 (1·07–7·33)	2·79 (0·877–6·43)	-11·1 (-69·1–176)
Azerbaijan	2·79 (0·913–6·47)	2·9 (0·828–7·01)	4·1 (-64·5–240)	6·82 (2·44–15·5)	8·24 (2·88–19)	20·9 (-57·4–263)	9·61 (3·39–22·5)	11·1 (3·68–25·9)	16 (-58·1–261)
Georgia	5·85 (1·88–13·9)	4·9 (1·55–11·8)	-16·3 (-69·6–159)	5·34 (1·89–12)	4·5 (1·47–9·95)	-15·6 (-69·1–154)	11·2 (3·75–25·9)	9·4 (3·03–21·8)	-16 (-69·8–163)
Kazakhstan	11·3 (3·76–26·7)	7·46 (2·32–16·9)	-33·8 (-77·3–116)	25·3 (9·75–54·8)	20 (6·61–44·6)	-21·1 (-71·5–135)	36·6 (13·6–81·6)	27·4 (8·66–61·5)	-25 (-73·8–129)
Kyrgyzstan	8·79 (2·82–20·6)	3·62 (1·18–8·31)	-58·8 (-85·6–35)	87·9 (34·8–179)	60 (23·8–118)	-31·8 (-73·1–75·7)	96·7 (37·6–201)	63·6 (24·7–127)	-34·3 (-74·3–72·2)
Mongolia	1·47 (0·525–3·14)	1·16 (0·351–2·57)	-20·5 (-73·3–147)	3·65 (1·19–8·49)	3·62 (1·23–7·91)	-0·7 (-64·8–227)	5·11 (1·77–12)	4·79 (1·56–10·3)	-6·4 (-67·5–210)
Tajikistan	7·41 (2·52–16·5)	14·8 (4·05–34·2)	99·7 (-29·4–565)	5·41 (1·97–12·4)	8·42 (2·85–19·7)	55·5 (-45·5–363)	12·8 (4·48–28·8)	23·2 (6·81–54·6)	81 (-37·2–457)
Turkmenistan	1·77 (0·629–4·23)	2·64 (0·806–5·98)	48·9 (-51·6–371)	2·57 (0·975–5·48)	4·24 (1·4–9·71)	64·9 (-42·7–393)	4·35 (1·6–9·71)	6·88 (2·21–15·8)	58·4 (-46·8–388)
Uzbekistan	16·8 (5·59–39·5)	21·2 (6·52–48·2)	26·3 (-57·1–294)	122 (47·7–258)	184 (63–394)	50·9 (-43·2–329)	138 (53·4–296)	205 (69·4–440)	47·9 (-45·2–326)
Central Europe	150 (47·9–358)	166 (51·9–387)	10 (-61–268)	213 (78·6–485)	284 (96·2–623)	33·5 (-51·7–306)	363 (121–845)	450 (146–1020)	23·8 (-55·6–291)
Albania	1·95 (0·61–4·37)	2·99 (0·835–6·92)	53·2 (-46·8–396)	3·42 (1·22–7·68)	7·15 (2·23–16·5)	109 (-29–494)	5·37 (1·88–12·4)	10·1 (3·1–23·6)	88·8 (-36·6–453)
Bosnia and Herzegovina	3·23 (0·999–7·52)	5·52 (1·71–12·7)	71·2 (-40·9–450)	4·56 (1·63–10·6)	7·43 (2·53–16·5)	63·1 (-41–400)	7·78 (2·62–18·3)	13 (4·34–28·7)	66·5 (-38·6–433)

	Female			Male			Both		
	Number of deaths		Change (%)	Number of deaths		Change (%)	Number of deaths		Change (%)
	1990	2023		1990	2023		1990	2023	
Bulgaria	22·2 (6·99–52·2)	29·5 (9·42–68)	32·5 (-53–339)	36·9 (12·3–84·7)	45·2 (15·3–101)	22·5 (-55·8–275)	59·2 (19·4–137)	74·7 (24·8–169)	26·2 (-54·2–297)
Croatia	4·63 (1·45–10·8)	3·19 (1–7·29)	-31·1 (-75·9–128)	6·54 (2·5–14·4)	5·39 (1·84–12)	-17·6 (-70·1–149)	11·2 (3·99–25·6)	8·58 (2·76–19·5)	-23·2 (-72·7–143)
Czechia	17·2 (5·32–41)	10·4 (3·36–24·1)	-39·7 (-78·7–97·6)	20·5 (7·39–46·2)	20·4 (6·93–44·4)	-0·6 (-62·8–208)	37·8 (12·4–86·4)	30·8 (10·2–68·4)	-18·4 (-70–161)
Hungary	12·1 (3·85–28·7)	6·79 (2·13–15·8)	-43·9 (-80·2–81·8)	17·1 (6·39–37·9)	10·9 (3·8–23·9)	-36·4 (-77·1–99·5)	29·2 (10·3–67·8)	17·7 (5·91–39·7)	-39·5 (-78·7–92·5)
Montenegro	0·613 (0·201–1·47)	1·17 (0·36–2·63)	91 (-35–465)	0·842 (0·284–1·94)	1·59 (0·54–3·5)	89 (-29·5–477)	1·45 (0·486–3·46)	2·76 (0·928–6·18)	89·8 (-30·6–487)
North Macedonia	2 (0·666–4·7)	3·91 (1·16–9·18)	95·4 (-31·9–525)	2·95 (1·01–6·76)	5·49 (1·82–11·9)	86 (-29·1–491)	4·95 (1·64–11·5)	9·4 (3·1–21)	89·8 (-31·1–497)
Poland	40 (12·8–94·1)	53·1 (16·6–125)	32·9 (-51·4–346)	47·9 (18·4–107)	85 (29·7–186)	77·3 (-35·3–446)	87·9 (30·9–203)	138 (45·7–311)	57·1 (-42·3–409)
Romania	24 (7·49–56·7)	23·7 (7·28–55·7)	-1·2 (-64·5–224)	39 (14·2–89·1)	55 (18·9–122)	41 (-48·7–337)	63 (20·8–147)	78·7 (25·8–179)	24·9 (-55·2–292)
Serbia	14·9 (4·54–36·6)	18 (5·3–41·1)	21 (-58·9–288)	20·6 (6·81–47·2)	25·8 (8·16–57·7)	25·2 (-56·5–298)	35·5 (11·6–84·7)	43·8 (13·4–99·8)	23·5 (-57·4–267)
Slovakia	3·54 (1·14–8·81)	2·43 (0·767–5·43)	-31·4 (-76·4–108)	6·18 (2·29–13·7)	5·5 (2·03–12·2)	-10·9 (-68·4–179)	9·72 (3·56–22·5)	7·93 (2·78–17·7)	-18·4 (-71·3–168)
Slovenia	1·68 (0·516–4·11)	2·38 (0·757–5·53)	42 (-50·6–343)	2·65 (0·983–5·94)	4·82 (1·71–10·7)	81·6 (-31·3–468)	4·33 (1·56–10·2)	7·2 (2·37–16·1)	66·3 (-38·7–414)
Eastern Europe	342 (106–808)	225 (70·6–532)	-34·2 (-76·8–116)	292 (110–649)	287 (97·5–631)	-1·8 (-64·3–205)	634 (207–1460)	512 (164–1160)	-19·3 (-70·6–161)
Belarus	9·6 (3·04–22·5)	7·89 (2·57–18·2)	-17·9 (-71·7–167)	11·6 (4·34–25·7)	15·7 (5·69–34·9)	36 (-50–345)	21·2 (7·2–49·4)	23·6 (7·79–52·7)	11·6 (-60–269)
Estonia	1·85 (0·594–4·37)	0·738 (0·224–1·71)	-60 (-86·1–32)	1·71 (0·642–3·76)	2·57 (0·931–5·61)	50·5 (-41·6–361)	3·55 (1·18–8·24)	3·31 (1·16–7·3)	-6·9 (-64·7–196)
Latvia	3·84 (1·22–9·22)	2·91 (0·907–6·71)	-24·3 (-73·7–140)	2·92 (1·09–6·6)	2·48 (0·825–5·47)	-15·2 (-70·3–160)	6·76 (2·22–15·9)	5·39 (1·75–12·2)	-20·4 (-72·1–153)
Lithuania	2·26 (0·736–5·47)	2·33 (0·728–5·41)	3 (-64·5–233)	2·62 (0·978–5·71)	3·03 (1·05–6·74)	15·6 (-57·3–269)	4·89 (1·76–11·2)	5·36 (1·74–12·3)	9·8 (-60·8–253)

	Female			Male			Both		
	Number of deaths		Change (%)	Number of deaths		Change (%)	Number of deaths		Change (%)
	1990	2023		1990	2023		1990	2023	
Republic of Moldova	3·13 (0·969–7·09)	1·97 (0·617–4·65)	-36·9 (-77·8–110)	5·76 (2·21–12·8)	6·32 (2·07–13·7)	9·7 (-60·9–229)	8·89 (3·17–20)	8·29 (2·67–18·2)	-6·7 (-67·1–192)
Russian Federation	251 (78·5–586)	179 (55·8–426)	-28·6 (-74·6–130)	197 (75·4–436)	203 (68·6–446)	3·1 (-62·4–220)	448 (146–1040)	383 (122–870)	-14·6 (-68·8–178)
Ukraine	70·2 (21·7–166)	29·7 (9·42–66·9)	-57·6 (-85·4–36·4)	70·3 (26–158)	53·3 (18·2–120)	-24·2 (-72·7–130)	140 (45·9–322)	83 (27·2–187)	-40·9 (-79·3–84·3)
**High-income**	755 (250–1740)	862 (276–2010)	14·2 (-59·2–263)	4160 (2550–6450)	4370 (2840–6980)	4·9 (-35·3–72·8)	4920 (2800–8160)	5230 (3340–8770)	6·4 (-37–90·9)
Australasia	12 (3·86–28)	14·4 (4·57–33·5)	20·5 (-57·3–290)	36·9 (13·9–79·2)	50·1 (18–111)	35·8 (-49·3–306)	48·9 (18·4–107)	64·5 (22·5–145)	32·1 (-51·4–304)
Australia	9·88 (3·18–23·1)	11·6 (3·65–27·1)	17·5 (-58–277)	31·2 (11·8–66·6)	42·2 (15·2–93·3)	35·4 (-49·5–301)	41·1 (15·5–89·5)	53·9 (18·8–121)	31·1 (-51·7–298)
New Zealand	2·1 (0·672–4·99)	2·83 (0·887–6·57)	34·6 (-53·1–332)	5·7 (2·16–12·5)	7·86 (2·77–17·5)	37·9 (-48·1–319)	7·8 (2·89–17·5)	10·7 (3·66–24)	37 (-49·3–332)
High-income Asia Pacific	146 (46·3–364)	194 (57·7–448)	32·4 (-51·5–319)	473 (175–1080)	675 (229–1500)	42·8 (-47·4–327)	619 (216–1450)	869 (290–1960)	40·3 (-48·7–333)
Brunei Darussalam	0·315 (0·112–0·778)	0·662 (0·216–1·51)	110 (-29·3–575)	0·517 (0·184–1·27)	1·07 (0·367–2·41)	107 (-28·7–581)	0·832 (0·29–2·04)	1·73 (0·584–3·92)	108 (-27·5–535)
Japan	102 (31·9–249)	159 (47·2–364)	55·3 (-42·4–386)	347 (129–778)	552 (188–1230)	59·1 (-41·9–372)	449 (159–1030)	711 (238–1610)	58·2 (-42·2–384)
Republic of Korea	42·1 (12·9–110)	32·2 (9·89–77·4)	-23·6 (-72·6–142)	119 (41·2–283)	113 (37·2–239)	-5·1 (-66·5–182)	162 (54·2–393)	145 (47·4–318)	-9·9 (-67·8–176)
Singapore	1·54 (0·489–3·61)	1·92 (0·599–4·35)	24·9 (-54·9–295)	5·89 (2·23–12·6)	8·63 (3·1–19·2)	46·6 (-41·8–350)	7·42 (2·78–16·2)	10·5 (3·68–23·6)	42·1 (-45–339)
High-income North America	361 (122–785)	436 (140–1010)	20·9 (-57·6–267)	3320 (2240–4890)	3240 (2030–4970)	-2·5 (-34·4–45·1)	3680 (2360–5600)	3670 (2340–5590)	-0·2 (-35·5–60·6)
Canada	29·9 (9·71–70·9)	43·6 (14·1–99·1)	45·9 (-48·9–363)	203 (82·4–388)	183 (65·8–384)	-9·8 (-64·5–154)	232 (93·3–459)	226 (80·1–477)	-2·6 (-62·4–184)
Greenland	0·0809 (0·028–0·181)	0·0708 (0·0224–0·166)	-12·5 (-70·4–205)	0·744 (0·289–1·64)	0·794 (0·294–1·74)	6·7 (-61·4–227)	0·825 (0·317–1·79)	0·865 (0·317–1·9)	4·8 (-62–218)
United States of America	331 (112–720)	393 (124–915)	18·6 (-58·3–254)	3110 (2150–4500)	3050 (1890–4690)	-2 (-32·5–46·5)	3450 (2270–5180)	3440 (2160–5290)	0 (-33·9–53·3)

	Female			Male			Both		
	Number of deaths		Change (%)	Number of deaths		Change (%)	Number of deaths		Change (%)
	1990	2023		1990	2023		1990	2023	
Southern Latin America	22·8 (7·37–54·5)	21 (6·56–47·7)	-8·1 (-67·9–204)	30·4 (11·5–67·8)	24·8 (8·57–54·6)	-18·3 (-70·7–155)	53·2 (19·4–122)	45·8 (14·9–103)	-14 (-69·5–178)
Argentina	15 (4·86–36·1)	13·1 (4·12–29·4)	-13·2 (-69·2–193)	18·4 (6·89–41·3)	15·2 (5·35–33·2)	-17 (-70·1–159)	33·4 (12·1–77·1)	28·3 (9·14–62·7)	-15·3 (-70·2–174)
Chile	5·18 (1·71–12·3)	5·78 (1·82–13·2)	11·5 (-60·8–262)	8·86 (3·35–19·6)	7·43 (2·47–16·6)	-16·1 (-69·8–160)	14 (5·06–31·9)	13·2 (4·26–30)	-5·9 (-66·6–197)
Uruguay	2·59 (0·835–6·14)	2·12 (0·631–4·84)	-18 (-71·7–157)	3·18 (1·21–6·75)	2·16 (0·755–4·76)	-32·2 (-75·4–116)	5·77 (2·1–13·2)	4·28 (1·38–9·86)	-25·8 (-73·2–135)
Western Europe	213 (66·8–519)	197 (62·9–457)	-7·6 (-67·1–208)	305 (114–669)	384 (135–851)	25·8 (-54·7–279)	518 (187–1190)	581 (194–1330)	12·1 (-60·4–253)
Andorra	0·00829 (0·00248–0·0201)	0·021 (0·00632–0·0495)	154 (-18·7–750)	0·0242 (0·00849–0·0531)	0·0642 (0·0216–0·141)	165 (-10–773)	0·0325 (0·0113–0·0742)	0·0852 (0·0274–0·191)	162 (-10·8–726)
Austria	4·7 (1·45–11·5)	3·87 (1·26–8·83)	-17·7 (-70·9–169)	6·31 (2·35–14)	11·1 (3·92–24·4)	76·2 (-36·3–423)	11 (3·89–25·7)	15 (5·12–33·4)	36·1 (-51·1–323)
Belgium	5·38 (1·71–13·1)	3·81 (1·22–8·82)	-29·2 (-74·7–133)	6·27 (2·34–13·8)	5·61 (1·98–12·8)	-10·5 (-68·5–178)	11·7 (4·14–27·2)	9·42 (3·16–22)	-19·1 (-71·4–163)
Cyprus	0·385 (0·129–0·923)	0·309 (0·0926–0·685)	-19·7 (-74·9–159)	0·59 (0·181–1·38)	0·606 (0·208–1·39)	2·8 (-62–238)	0·975 (0·316–2·34)	0·916 (0·3–2·05)	-6·1 (-67·3–192)
Denmark	3·75 (1·17–8·97)	4·96 (1·56–11·5)	32·3 (-52·4–328)	9·29 (3·52–20·8)	12·1 (4·37–26·2)	30·7 (-52·4–274)	13 (4·66–29·8)	17·1 (5·81–37·9)	31·1 (-52·2–292)
Finland	2·46 (0·772–6·05)	2·53 (0·807–5·87)	2·6 (-63·1–226)	2·33 (0·846–5·31)	4·05 (1·42–8·95)	74·2 (-36·4–420)	4·79 (1·58–11·3)	6·58 (2·21–14·9)	37·4 (-50·2–324)
France	23·1 (7·39–55·6)	21·5 (6·88–50·3)	-6·9 (-67–210)	47·1 (17·4–97)	37·9 (13·2–85·4)	-19·6 (-71·4–145)	70·2 (25·7–151)	59·4 (19·9–137)	-15·4 (-70·3–169)
Germany	50·6 (15·8–124)	51·4 (16·8–120)	1·7 (-63·7–237)	58·5 (22–129)	114 (39·3–253)	94·1 (-29·5–506)	109 (37·9–253)	165 (54·4–378)	51·2 (-45·6–388)
Greece	8·56 (2·64–20·6)	6·78 (2·1–15·6)	-20·8 (-72·6–163)	9·81 (3·32–22·7)	8·69 (2·89–20·5)	-11·4 (-68·4–169)	18·4 (6·07–43·4)	15·5 (5·08–35·8)	-15·8 (-70·3–167)
Iceland	0·115 (0·0363–0·277)	0·2 (0·0631–0·459)	74·2 (-39·7–499)	0·353 (0·132–0·784)	0·998 (0·369–2·17)	183 (8·4–735)	0·468 (0·169–1·07)	1·2 (0·437–2·63)	156 (-4–679)
Ireland	1·41 (0·448–3·34)	1·25 (0·391–2·97)	-10·8 (-67·5–199)	1·76 (0·65–3·9)	2·08 (0·745–4·64)	18·2 (-55·9–257)	3·17 (1·16–7·27)	3·34 (1·12–7·58)	5·3 (-61·5–234)

Return to Table of Contents

	Female			Male			Both		
	Number of deaths		Change (%)	Number of deaths		Change (%)	Number of deaths		Change (%)
	1990	2023		1990	2023		1990	2023	
Israel	1·08 (0·335–2·57)	1·65 (0·53–3·83)	53·4 (-46·7–392)	1·76 (0·64–3·97)	2·85 (0·972–6·41)	61·8 (-39·5–382)	2·84 (0·957–6·58)	4·5 (1·5–10·3)	58·6 (-42–402)
Italy	29 (8·96–69·3)	27·9 (8·58–64·8)	-3·8 (-66–218)	47·3 (17·7–106)	49·1 (17·2–108)	3·9 (-62·1–208)	76·3 (27·3–176)	77 (26·2–176)	1 (-63·6–219)
Luxembourg	0·282 (0·0898–0·668)	0·167 (0·0525–0·386)	-40·7 (-79·2–91)	0·302 (0·109–0·69)	0·261 (0·0954–0·577)	-13·5 (-67·4–159)	0·584 (0·204–1·35)	0·428 (0·142–0·978)	-26·6 (-73·5–129)
Malta	0·139 (0·0434–0·332)	0·129 (0·0421–0·308)	-6·8 (-66·4–206)	0·184 (0·067–0·417)	0·207 (0·0718–0·467)	12·6 (-57·5–248)	0·323 (0·111–0·743)	0·337 (0·114–0·783)	4·3 (-61–232)
Monaco	0·0463 (0·0144–0·108)	0·0265 (0·00771–0·0706)	-42·8 (-83·9–90·6)	0·0591 (0·0207–0·14)	0·0492 (0·0173–0·109)	-16·9 (-74·9–152)	0·105 (0·0353–0·254)	0·0757 (0·025–0·184)	-28·3 (-76·6–131)
Netherlands	5·74 (1·82–13·8)	6·68 (2·14–15·6)	16·3 (-58·7–284)	6·41 (2·41–14·4)	10·1 (3·54–22·5)	57·1 (-42·3–371)	12·2 (4·34–27·8)	16·8 (5·65–38·4)	37·8 (-49·7–333)
Norway	3·52 (1·1–8·43)	3·58 (1·12–8·36)	1·6 (-63·8–237)	7·43 (2·71–16·7)	15 (5·64–32·5)	101 (-23·2–498)	11 (3·77–25·6)	18·5 (6·66–40·9)	69·3 (-35·6–422)
Portugal	10·1 (3·09–24)	5·73 (1·77–13)	-43·1 (-79·8–82·4)	11·6 (4·2–26·6)	8·84 (3·08–19·6)	-23·8 (-71·8–140)	21·7 (7·04–50·7)	14·6 (4·78–33·3)	-32·8 (-75·8–115)
San Marino	0·00976 (0·0032–0·0231)	0·0137 (0·00384–0·0328)	40·6 (-56·2–367)	0·0175 (0·00564–0·0415)	0·0223 (0·00752–0·0568)	27·1 (-55·6–309)	0·0273 (0·00905–0·0654)	0·036 (0·0119–0·085)	32 (-53·9–317)
Spain	19·9 (6·14–48·5)	15·1 (4·75–34·8)	-24·2 (-72·5–153)	27·9 (10·3–58·4)	23 (8·02–51)	-17·7 (-71–154)	47·8 (17·4–108)	38 (12·6–87·3)	-20·4 (-72·2–154)
Sweden	4·95 (1·55–12)	9·86 (3·23–22·7)	99·2 (-28·5–547)	21·3 (8·04–46·8)	32·5 (11·9–70·1)	52·5 (-42·7–343)	26·3 (9·85–58·8)	42·4 (14·9–93·6)	61·3 (-40·5–378)
Switzerland	3·25 (1·03–7·77)	2·91 (0·924–6·77)	-10·3 (-67·8–205)	4·8 (1·79–10·3)	5·46 (1·95–12·1)	13·8 (-59·8–233)	8·05 (2·92–18·1)	8·38 (2·84–19·1)	4·1 (-63–225)
United Kingdom	34·2 (10·9–80·8)	26·1 (8·3–60·8)	-23·7 (-73–150)	33·6 (12·9–73·8)	39·6 (14–88)	17·9 (-56·6–265)	67·8 (24·3–156)	65·8 (22·5–151)	-3·1 (-64·9–207)
**Latin America and Caribbean**	264 (85·6–625)	436 (140–1010)	64·8 (-41·7–438)	405 (154–898)	784 (274–1700)	93·5 (-29·7–496)	670 (236–1530)	1220 (406–2730)	82·2 (-34·6–477)
Andean Latin America	8·71 (2·85–20·8)	14 (4·22–31·6)	61 (-47·8–413)	10·8 (3·7–26·9)	21·6 (6·47–47·5)	99·7 (-28·4–545)	19·5 (6·45–48)	35·6 (10·6–80·3)	82·4 (-38·8–476)
Bolivia (Plurinational State of)	1·75 (0·573–3·97)	2·79 (0·892–6·38)	58·8 (-47·1–422)	3·23 (0·953–8·27)	7·7 (2·64–18·3)	138 (-30·5–727)	4·99 (1·49–12·2)	10·5 (3·43–24·7)	110 (-34·5–593)

Return to Table of Contents

	Female			Male			Both		
	Number of deaths		Change (%)	Number of deaths		Change (%)	Number of deaths		Change (%)
	1990	2023		1990	2023		1990	2023	
Ecuador	1·7 (0·544–4·07)	2·87 (0·873–6·46)	68·5 (-40·7–450)	1·82 (0·638–4·14)	2·92 (0·962–6·53)	60·4 (-40·9–396)	3·52 (1·14–8·25)	5·78 (1·85–13·4)	64·3 (-40·8–415)
Peru	5·25 (1·75–12·8)	8·37 (2·38–19·6)	59·3 (-55–416)	5·76 (1·96–13·8)	11 (3·2–23·8)	90·3 (-34·4–498)	11 (3·74–26·9)	19·3 (5·49–42·3)	75·5 (-43·2–455)
Caribbean	22·9 (7·63–51·6)	43·3 (13·7–101)	89·3 (-37·3–477)	30·1 (11·2–70·4)	62·5 (21·9–140)	108 (-23·6–521)	53 (18–123)	106 (36–239)	99·7 (-28·3–520)
Antigua and Barbuda	0·0386 (0·012–0·091)	0·0362 (0·0107–0·0863)	-6·1 (-67·4–214)	0·0464 (0·0175–0·105)	0·0585 (0·0196–0·131)	26·1 (-54·7–278)	0·085 (0·0288–0·198)	0·0948 (0·0299–0·217)	11·5 (-60·3–250)
Bahamas	0·099 (0·0326–0·227)	0·183 (0·0523–0·419)	85·3 (-32·5–537)	0·183 (0·0696–0·377)	0·383 (0·138–0·828)	110 (-21·9–570)	0·282 (0·106–0·626)	0·566 (0·183–1·26)	101 (-26·2–552)
Barbados	0·28 (0·091–0·669)	0·264 (0·0736–0·591)	-6 (-68·4–201)	0·159 (0·0612–0·347)	0·227 (0·0749–0·5)	42·6 (-48·2–340)	0·439 (0·151–1·03)	0·491 (0·15–1·09)	11·6 (-58·4–259)
Belize	0·0384 (0·0124–0·0899)	0·0807 (0·0239–0·189)	110 (-27·1–590)	0·0469 (0·0166–0·106)	0·124 (0·0408–0·278)	164 (-7·6–677)	0·0853 (0·0279–0·198)	0·204 (0·0625–0·469)	140 (-17·9–649)
Bermuda	0·0286 (0·00962–0·0689)	0·0335 (0·0105–0·0801)	17 (-59·2–279)	0·049 (0·0194–0·101)	0·0577 (0·0206–0·124)	17·9 (-57·7–262)	0·0776 (0·0295–0·167)	0·0913 (0·0313–0·203)	17·6 (-57·3–271)
Cuba	4·91 (1·6–11·4)	6·88 (2·24–15·8)	40·2 (-49·7–355)	6·92 (2·6–14·7)	12·8 (4·64–30·4)	84·6 (-32·9–469)	11·8 (4·37–26·7)	19·7 (7·06–46·7)	66·2 (-38·5–440)
Dominica	0·0382 (0·0121–0·0885)	0·0389 (0·0119–0·096)	1·8 (-65·9–246)	0·0321 (0·012–0·0737)	0·0465 (0·017–0·108)	44·9 (-46·1–345)	0·0703 (0·0237–0·167)	0·0854 (0·0284–0·202)	21·5 (-54·8–299)
Dominican Republic	2·84 (0·92–6·91)	5·74 (1·74–12·9)	102 (-26·2–532)	6·05 (2·25–12·8)	16 (5·41–36·4)	165 (6·9–697)	8·89 (3·21–19·7)	21·8 (7–49·4)	145 (-1·3–632)
Grenada	0·0847 (0·0278–0·193)	0·0798 (0·0237–0·191)	-5·8 (-65·5–205)	0·123 (0·0466–0·276)	0·15 (0·0537–0·336)	21·9 (-53·8–261)	0·208 (0·0737–0·473)	0·23 (0·0796–0·524)	10·6 (-57·9–242)
Guyana	0·424 (0·129–0·985)	0·365 (0·112–0·843)	-13·9 (-71·3–170)	0·569 (0·186–1·25)	0·517 (0·172–1·21)	-9·2 (-68·1–152)	0·993 (0·321–2·25)	0·882 (0·284–2·03)	-11·2 (-67·4–158)
Haiti	9·43 (3·2–20·9)	23·9 (7·61–56·2)	154 (-17·8–702)	9·94 (3·11–24·3)	24 (7·76–52·7)	141 (-17·6–687)	19·4 (6·53–43)	47·9 (16·4–107)	147 (-14·8–692)
Jamaica	1·38 (0·445–3·36)	1·59 (0·439–3·63)	14·6 (-59·1–269)	1·29 (0·448–3·05)	2·15 (0·717–4·89)	66·1 (-42·2–397)	2·68 (0·878–6·4)	3·73 (1·18–8·61)	39·5 (-50·6–339)
Puerto Rico	1·46 (0·485–3·44)	1·3 (0·424–3·01)	-10·8 (-69·7–185)	2·33 (0·85–4·84)	1·77 (0·63–3·9)	-24·2 (-72·9–128)	3·78 (1·36–8·23)	3·06 (1·02–6·88)	-19 (-71·7–148)

	Female			Male			Both		
	Number of deaths		Change (%)	Number of deaths		Change (%)	Number of deaths		Change (%)
	1990	2023		1990	2023		1990	2023	
Saint Kitts and Nevis	0·0461 (0·0145–0·107)	0·0266 (0·00795–0·0626)	-42·3 (-79·7–92·1)	0·0342 (0·0121–0·076)	0·0268 (0·00849–0·0594)	-21·5 (-71·7–139)	0·0803 (0·0261–0·186)	0·0534 (0·0162–0·123)	-33·5 (-75·6–113)
Saint Lucia	0·0931 (0·0303–0·225)	0·0883 (0·027–0·206)	-5·2 (-68·1–238)	0·105 (0·0402–0·214)	0·152 (0·0516–0·358)	44·9 (-51·8–346)	0·198 (0·0704–0·456)	0·24 (0·0746–0·564)	21·4 (-58·9–298)
Saint Vincent and the Grenadines	0·0498 (0·0161–0·115)	0·0499 (0·0152–0·114)	0·1 (-64–220)	0·0819 (0·0317–0·167)	0·149 (0·0478–0·345)	82·4 (-40·2–466)	0·132 (0·0496–0·298)	0·199 (0·0629–0·453)	51·3 (-48·6–384)
Suriname	0·158 (0·0515–0·386)	0·321 (0·0963–0·792)	103 (-32·8–565)	0·198 (0·072–0·447)	0·406 (0·132–0·929)	105 (-25·6–481)	0·356 (0·124–0·835)	0·726 (0·228–1·72)	104 (-29–521)
Trinidad and Tobago	0·709 (0·228–1·66)	0·912 (0·275–2·09)	28·6 (-55·3–292)	0·891 (0·32–1·99)	1·32 (0·45–2·94)	48·6 (-44·6–373)	1·6 (0·535–3·7)	2·24 (0·715–5·07)	39·7 (-50·5–354)
United States Virgin Islands	0·0248 (0·00814–0·0567)	0·0318 (0·00949–0·0728)	28 (-55·1–300)	0·0382 (0·0146–0·0812)	0·0576 (0·0212–0·124)	50·9 (-44·7–364)	0·063 (0·0228–0·141)	0·0894 (0·0305–0·193)	41·9 (-46·8–331)
Central Latin America	56·8 (18·4–134)	105 (31·7–253)	84·9 (-35·9–503)	73·7 (28·1–164)	157 (50·8–342)	113 (-25·5–566)	131 (47·6–301)	262 (82·8–595)	101 (-30·6–551)
Colombia	14·2 (4·6–33·2)	19·3 (6·11–44·2)	36·1 (-52·3–345)	14·4 (5·44–31·8)	19·8 (6·72–43·9)	37·7 (-51–311)	28·6 (10·3–65·2)	39·1 (12·8–88·4)	36·9 (-52–328)
Costa Rica	0·558 (0·181–1·33)	1·42 (0·468–3·28)	154 (-12–719)	0·817 (0·297–1·81)	2·04 (0·711–4·37)	150 (-9·5–627)	1·37 (0·497–3·15)	3·46 (1·19–7·83)	152 (-11·2–666)
El Salvador	2·09 (0·684–5·01)	2·97 (0·865–6·85)	42·2 (-50·8–368)	2·38 (0·838–5·39)	2·79 (0·919–6·07)	17·2 (-56·2–273)	4·46 (1·53–10·4)	5·75 (1·79–12·9)	28·9 (-54·1–309)
Guatemala	1·87 (0·62–4·37)	4·31 (1·3–9·97)	131 (-23–642)	1·73 (0·67–3·79)	3·53 (1·15–7·94)	104 (-26·4–538)	3·6 (1·32–8·16)	7·84 (2·43–18)	118 (-23·7–590)
Honduras	1·18 (0·376–2·62)	3·47 (1·17–8·58)	194 (-2·8–843)	1·76 (0·619–4·29)	5·05 (1·78–12·8)	188 (-6·6–868)	2·94 (0·985–6·91)	8·52 (3·05–19·9)	190 (-0·8–797)
Mexico	18·8 (6–43·9)	26·8 (8·4–62·9)	42·6 (-51·7–378)	17·3 (6·49–39)	29 (10·3–61·4)	67 (-36·8–407)	36·2 (12·5–84·2)	55·8 (18·7–126)	54·3 (-45·1–396)
Nicaragua	1·06 (0·352–2·54)	1·67 (0·54–4·19)	57·1 (-44·8–405)	1·08 (0·362–2·57)	1·87 (0·62–4·09)	73·8 (-37·1–436)	2·14 (0·708–5·12)	3·54 (1·14–8·46)	65·5 (-39·8–427)
Panama	1·16 (0·381–2·75)	2·59 (0·812–5·87)	123 (-19·7–627)	1·25 (0·456–2·88)	2·67 (0·928–5·92)	114 (-19·9–544)	2·41 (0·802–5·61)	5·26 (1·74–11·7)	118 (-20·5–576)
Venezuela (Bolivarian Republic of)	15·9 (5·16–36·9)	42·5 (11·5–105)	167 (-10·4–789)	33 (12·7–72·4)	90·4 (28·4–200)	174 (-3·1–755)	48·9 (18·4–110)	133 (38·7–298)	172 (-4·1–797)

	Female			Male			Both		
	Number of deaths		Change (%)	Number of deaths		Change (%)	Number of deaths		Change (%)
	1990	2023		1990	2023		1990	2023	
Tropical Latin America	176 (56·9–417)	273 (88·3–632)	55·3 (-43·2–419)	291 (111–637)	543 (193–1180)	86·9 (-30·9–472)	467 (163–1060)	816 (277–1810)	75 (-35·3–453)
Brazil	169 (54·6–400)	262 (84·4–604)	55·3 (-43–419)	277 (106–606)	514 (182–1120)	85·3 (-31·3–468)	446 (156–1010)	776 (263–1720)	73·9 (-35·4–451)
Paraguay	7·08 (2·44–16·5)	11·1 (3·47–24·6)	56·8 (-44·9–413)	13·2 (4·94–30·3)	29 (10·5–61·3)	119 (-21·6–536)	20·3 (7·14–47·5)	40·1 (13·9–85·9)	97·2 (-30·1–493)
North Africa and Middle East	629 (233–1290)	687 (354–1330)	9·2 (-48·2–135)	1460 (632–3000)	1740 (882–3200)	19·2 (-38·5–155)	2090 (876–4160)	2420 (1270–4440)	16·2 (-38·2–139)
Afghanistan	23·6 (7·64–53·5)	22·9 (7·52–54·6)	-3·1 (-69·1–206)	46·1 (15–112)	51·9 (17·4–125)	12·6 (-62·6–307)	69·8 (22·7–165)	74·8 (26·8–172)	7·3 (-65·8–265)
Algeria	32·9 (10·7–76·8)	43·4 (13·2–106)	31·9 (-58–291)	142 (47·6–329)	249 (80·5–586)	75·2 (-40·4–438)	175 (58·5–401)	292 (99·2–679)	67 (-42·7–408)
Bahrain	0·242 (0·0814–0·581)	0·237 (0·0674–0·574)	-2·3 (-68·8–211)	0·555 (0·191–1·28)	1·05 (0·35–2·32)	88·6 (-37·9–464)	0·798 (0·275–1·87)	1·28 (0·423–2·83)	61 (-45·5–394)
Egypt	27·8 (8·5–69·9)	41·1 (12·8–99·7)	47·6 (-59·1–371)	122 (38·2–293)	183 (57·4–424)	49·6 (-53·3–418)	150 (48·2–354)	224 (72–518)	49·2 (-51–390)
Iran (Islamic Republic of)	48·1 (15·8–113)	37·8 (12·3–96·9)	-21·4 (-73·7–139)	98·1 (31·3–237)	82·1 (27·1–192)	-16·4 (-73·1–173)	146 (47·1–349)	120 (40·1–284)	-18 (-72·8–167)
Iraq	9·58 (3·16–23)	15·2 (4·92–36·6)	59·1 (-50–403)	16·2 (5·06–39·7)	32·8 (10·6–86·7)	102 (-38·3–644)	25·8 (8·31–62·3)	48·1 (15·8–121)	86·3 (-39·5–555)
Jordan	1·26 (0·406–3·07)	2·32 (0·669–5·68)	84·2 (-39·6–506)	2·33 (0·687–5·74)	4·11 (1·36–9·66)	76·2 (-50·7–516)	3·6 (1·16–8·8)	6·44 (2·06–15·5)	79 (-44·8–509)
Kuwait	0·225 (0·0767–0·517)	0·397 (0·123–0·934)	76·6 (-39·9–480)	0·561 (0·212–1·22)	1·32 (0·46–2·98)	136 (-14·9–579)	0·786 (0·291–1·76)	1·72 (0·587–3·94)	119 (-21·9–556)
Lebanon	1·65 (0·458–4·04)	2·23 (0·664–5·05)	35·4 (-59·3–390)	3·35 (0·963–8·15)	5·2 (1·74–11·4)	55·2 (-56·7–497)	5 (1·55–12·5)	7·44 (2·46–16·9)	48·7 (-51·4–433)
Libya	2·55 (0·833–5·88)	4·81 (1·42–10·9)	88·6 (-46·5–527)	5·9 (1·84–14·4)	8·86 (2·61–20·7)	50·3 (-53·8–394)	8·45 (2·71–20·1)	13·7 (4·75–30·3)	61·9 (-45·8–423)
Morocco	27·2 (8·65–64)	40·5 (12–95·5)	49·1 (-50·3–376)	27·8 (8·09–69·3)	40·5 (11·7–100)	45·8 (-52·9–375)	55 (17·3–130)	81 (26·6–187)	47·4 (-50·3–373)
Oman	0·91 (0·289–2·19)	1·21 (0·375–2·81)	33·3 (-60·7–323)	2·22 (0·703–5·25)	2·84 (0·88–6·62)	28·2 (-54·5–365)	3·12 (1·01–7·33)	4·05 (1·33–9·22)	29·7 (-55·9–330)

	Female			Male			Both		
	Number of deaths		Change (%)	Number of deaths		Change (%)	Number of deaths		Change (%)
	1990	2023		1990	2023		1990	2023	
Palestine	2·13 (0·679–5·44)	1·58 (0·488–3·76)	-26 (-76·8–148)	3·78 (1·14–8·94)	2·99 (1–6·75)	-20·7 (-74·1–160)	5·91 (1·89–14·9)	4·57 (1·5–10·4)	-22·6 (-74·3–153)
Qatar	0·0457 (0·0153–0·104)	0·0834 (0·0245–0·21)	82·6 (-45·4–455)	0·14 (0·0436–0·336)	0·377 (0·122–0·874)	169 (-18·4–815)	0·186 (0·06–0·453)	0·46 (0·153–1·03)	148 (-23·4–668)
Saudi Arabia	7·86 (2·64–18·2)	8·62 (2·39–21·1)	9·7 (-63·3–260)	12·3 (3·81–29·2)	13·4 (4·29–29)	8·3 (-68·1–259)	20·2 (6·59–48·3)	22 (7·08–50·4)	8·9 (-65·6–254)
Sudan	24·5 (7·65–56·5)	27·9 (8·47–70·9)	14·2 (-64–280)	172 (50·3–426)	216 (67·9–537)	25·9 (-56·7–281)	196 (59·6–472)	244 (76·4–590)	24·5 (-56·9–278)
Syrian Arab Republic	3·28 (1·02–8·05)	4·57 (1·25–12·2)	39·4 (-58·4–374)	4·34 (1·3–10·6)	5·96 (1·84–13·5)	37·3 (-54–394)	7·62 (2·48–18·5)	10·5 (3·25–25·2)	38·2 (-57–365)
Tunisia	33·1 (10·8–71·5)	37·2 (11·6–80·2)	12·5 (-61·5–256)	58·3 (19·1–131)	65·4 (22·8–153)	12·2 (-62–275)	91·4 (31·1–212)	103 (36·7–229)	12·3 (-59·5–241)
Türkiye	67·9 (22·8–159)	67·3 (18·9–166)	-0·9 (-70·7–252)	72·4 (23·6–171)	74·6 (24·5–173)	3 (-67–252)	140 (47–336)	142 (43·4–337)	1·1 (-67·6–228)
United Arab Emirates	0·139 (0·0417–0·36)	0·263 (0·0777–0·624)	89 (-42·7–684)	0·589 (0·149–1·71)	1·78 (0·573–4·22)	203 (-2·6–1140)	0·729 (0·206–2·07)	2·05 (0·669–4·79)	181 (-9·5–1080)
Yemen	314 (117–619)	326 (156–613)	4 (-50·7–147)	665 (331–1180)	692 (318–1300)	4·1 (-53–130)	979 (468–1800)	1020 (513–1860)	4·1 (-50·1–135)
South Asia	31300 (16600–55100)	83600 (45600–137000)	167 (37·4–376)	48500 (26000–76200)	127000 (82000–188000)	161 (51·1–358)	79800 (43300–127000)	210000 (128000–318000)	163 (51·8–357)
Bangladesh	5780 (3120–9240)	18500 (10800–27600)	220 (87·4–412)	4600 (2290–7110)	14400 (10200–19700)	212 (97·6–461)	10400 (5700–16000)	32900 (22100–45100)	217 (104–423)
Bhutan	12·5 (5·44–23·8)	27·7 (13·5–48·2)	121 (1–396)	15·5 (6·55–29)	35·9 (19·8–60)	132 (22·7–369)	28 (12·9–48·8)	63·6 (34·9–106)	127 (20·6–338)
India	23400 (12100–43000)	59400 (30100–101000)	154 (25·1–379)	40900 (21900–64300)	104000 (65400–158000)	154 (46·6–353)	64300 (34500–104000)	163000 (97600–255000)	154 (41·8–347)
Nepal	345 (151–660)	766 (339–1460)	122 (-2·8–392)	744 (373–1200)	2040 (1320–3080)	174 (55·2–398)	1090 (546–1810)	2810 (1760–4300)	158 (48·3–374)
Pakistan	1760 (761–3390)	4890 (2140–9110)	178 (15·5–618)	2310 (1110–4410)	6370 (3260–12000)	175 (12·5–490)	4070 (2000–7630)	11300 (5520–20200)	176 (22·1–527)
**Southeast Asia, East Asia, and Oceania**	8150 (3040–16500)	11300 (4800–22400)	38·1 (-43·4–260)	8200 (3160–18500)	14700 (5710–30800)	79 (-31·8–387)	16300 (6180–34600)	25900 (10300–52700)	58·6 (-34–332)

	Female			Male			Both		
	Number of deaths		Change (%)	Number of deaths		Change (%)	Number of deaths		Change (%)
	1990	2023		1990	2023		1990	2023	
East Asia	1820 (595–4170)	2030 (638–4660)	12·1 (-63·9–256)	4510 (1670–10500)	6950 (2410–15000)	53·8 (-45·7–366)	6330 (2330–14900)	8980 (3050–19500)	41·9 (-49·7–335)
China	1790 (586–4110)	1990 (624–4540)	11·4 (-64·1–253)	4450 (1650–10300)	6820 (2360–14700)	53 (-46·1–362)	6240 (2300–14700)	8810 (2990–19100)	41·1 (-50–332)
Democratic People's Republic of Korea	13·1 (4·45–29·4)	29·6 (8·97–79)	126 (-24·2–573)	14 (4·66–33·3)	28·3 (8·94–65·7)	103 (-36·6–589)	27·1 (8·74–61·1)	57·9 (18·4–131)	114 (-26·5–580)
Taiwan	15·3 (4·82–36·3)	13·6 (4·27–30·5)	-10·7 (-67·3–180)	46·7 (17·6–98·5)	102 (38·3–224)	119 (-18·4–564)	61·9 (23·1–136)	116 (42·1–258)	86·8 (-30–477)
Oceania	14·6 (5·13–35·7)	61·9 (24·5–127)	323 (54–1120)	24·4 (9·56–56·5)	61·2 (21·2–143)	150 (-10·6–680)	39·1 (14·8–83·2)	123 (44·1–255)	215 (21·9–800)
American Samoa	0·0439 (0·015–0·099)	0·0733 (0·0232–0·175)	67 (-50–431)	0·149 (0·0502–0·35)	0·219 (0·078–0·52)	47·3 (-50·6–414)	0·193 (0·064–0·448)	0·293 (0·105–0·672)	51·8 (-47·4–396)
Cook Islands	0·0209 (0·00705–0·0469)	0·0175 (0·00563–0·0408)	-16·6 (-73·4–166)	0·0618 (0·0189–0·152)	0·073 (0·0273–0·164)	18·2 (-62·1–273)	0·0827 (0·0268–0·196)	0·0905 (0·033–0·204)	9·4 (-63·9–243)
Fiji	0·792 (0·279–1·8)	1·83 (0·546–4·06)	131 (-25·2–605)	1·96 (0·643–4·48)	3·48 (1·15–7·82)	77·7 (-35·6–455)	2·75 (0·942–6·25)	5·31 (1·72–11·5)	93 (-29·5–517)
Guam	0·0628 (0·0208–0·139)	0·232 (0·0924–0·495)	269 (42·7–1010)	0·46 (0·19–0·945)	1·56 (0·805–2·77)	240 (46·8–727)	0·523 (0·217–1·08)	1·8 (0·915–3·26)	243 (44·1–752)
Kiribati	0·0727 (0·0232–0·154)	0·213 (0·0674–0·496)	194 (-7·8–798)	0·547 (0·212–1·2)	1·11 (0·459–2·39)	103 (-21·8–465)	0·62 (0·235–1·33)	1·32 (0·534–2·76)	113 (-16·5–480)
Marshall Islands	0·0582 (0·0182–0·141)	0·178 (0·0666–0·427)	206 (1–789)	0·238 (0·09–0·511)	0·366 (0·152–0·794)	53·8 (-44·3–375)	0·296 (0·113–0·662)	0·544 (0·224–1·15)	83·8 (-28·9–459)
Micronesia (Federated States of)	0·171 (0·0571–0·4)	0·235 (0·0726–0·534)	37·2 (-57·2–318)	0·84 (0·312–1·84)	0·748 (0·276–1·74)	-10·9 (-67·7–189)	1·01 (0·364–2·24)	0·983 (0·365–2·2)	-2·8 (-65–200)
Nauru	0·00859 (0·00233–0·0212)	0·0189 (0·00564–0·0441)	120 (-33–641)	0·0148 (0·00398–0·0386)	0·0202 (0·00694–0·0473)	36·5 (-57·7–362)	0·0234 (0·00724–0·0618)	0·0391 (0·014–0·0875)	67·3 (-43·6–455)
Niue	0·00371 (0·00122–0·00829)	0·00325 (0·00104–0·00761)	-12·5 (-71·7–196)	0·00479 (0·00148–0·011)	0·00397 (0·00131–0·0086)	-17·2 (-71·2–161)	0·0085 (0·00275–0·0204)	0·00721 (0·00252–0·0166)	-15·1 (-70·3–171)
Northern Mariana Islands	0·0488 (0·017–0·112)	0·125 (0·0448–0·262)	155 (-14·3–704)	0·21 (0·0761–0·469)	0·513 (0·197–1·1)	144 (-11·8–606)	0·259 (0·0921–0·583)	0·638 (0·242–1·36)	146 (-10·3–624)
Palau	0·397 (0·181–0·715)	0·935 (0·455–1·67)	136 (5·5–441)	0·761 (0·337–1·44)	1·42 (0·687–2·66)	86·7 (-19·9–335)	1·16 (0·544–2·17)	2·36 (1·16–4·02)	104 (-8·1–344)

	Female			Male			Both		
	Number of deaths		Change (%)	Number of deaths		Change (%)	Number of deaths		Change (%)
	1990	2023		1990	2023		1990	2023	
Papua New Guinea	11·1 (3·8–28·2)	50·7 (20·3–107)	359 (67·8–1310)	13·8 (4·93–31·8)	37·6 (11·7–89·3)	173 (-5·9–887)	24·8 (9·27–53·7)	88·3 (32·4–185)	256 (33–905)
Samoa	0·131 (0·0398–0·303)	0·272 (0·0848–0·682)	107 (-40·3–588)	0·242 (0·0877–0·55)	0·43 (0·137–1·05)	77·6 (-41·1–555)	0·373 (0·126–0·849)	0·701 (0·234–1·71)	87·8 (-36·3–512)
Solomon Islands	0·629 (0·18–1·49)	3·63 (1·13–7·97)	477 (90·5–1800)	2·83 (0·962–6·37)	9·06 (3·34–20·3)	220 (25·2–905)	3·46 (1·22–7·84)	12·7 (4·46–26·9)	267 (40·6–1040)
Tokelau	0·00218 (0·000679–0·00492)	0·00276 (0·000838–0·00673)	26·8 (-60·4–329)	0·00566 (0·00188–0·0134)	0·00547 (0·00186–0·0122)	-3·4 (-66–238)	0·00784 (0·00254–0·0187)	0·00824 (0·00287–0·0192)	5 (-62·4–244)
Tonga	0·0649 (0·0209–0·143)	0·122 (0·0378–0·278)	88·3 (-43·9–511)	0·112 (0·0399–0·267)	0·133 (0·0427–0·318)	18·5 (-62·3–281)	0·177 (0·0589–0·399)	0·255 (0·0879–0·592)	44·1 (-51·1–348)
Tuvalu	0·0196 (0·00623–0·0448)	0·0236 (0·00749–0·0549)	20·4 (-66·4–292)	0·0381 (0·0123–0·0929)	0·0421 (0·0128–0·0935)	10·4 (-61·2–296)	0·0578 (0·0187–0·141)	0·0657 (0·0221–0·143)	13·8 (-60·8–273)
Vanuatu	0·152 (0·0483–0·35)	0·703 (0·22–1·73)	361 (46·6–1330)	0·694 (0·229–1·68)	1·84 (0·627–4·16)	165 (-5·9–774)	0·846 (0·275–2·1)	2·54 (0·885–5·75)	200 (5–873)
Southeast Asia	6320 (2450–12500)	9150 (4090–17700)	45 (-36·4–255)	3660 (1450–8140)	7660 (3240–15400)	110 (-10·1–440)	9970 (3870–19500)	16800 (7580–32500)	68·6 (-26·7–311)
Cambodia	391 (183–718)	988 (482–1800)	153 (13–466)	41 (15·1–105)	83·6 (31–204)	104 (-30·9–569)	432 (207–771)	1070 (576–1890)	148 (37–420)
Indonesia	2340 (865–4800)	2530 (844–5600)	7·8 (-60·6–192)	324 (110–764)	639 (215–1510)	97·3 (-28·7–542)	2670 (988–5440)	3160 (1070–7050)	18·6 (-56–219)
Lao People's Democratic Republic	109 (38·8–237)	243 (105–459)	123 (-14·1–479)	9·41 (2·94–24·3)	18·1 (5·92–43·3)	92·7 (-39·9–579)	118 (42·4–252)	262 (110–501)	121 (-14·2–484)
Malaysia	65·2 (21·3–152)	145 (53·2–338)	123 (-25·4–585)	68·1 (23·9–157)	245 (89·2–534)	260 (28–1080)	133 (44·8–314)	390 (143–861)	193 (3·8–807)
Maldives	2·71 (0·928–6·02)	4·4 (1·5–9·21)	62·5 (-41–392)	3·75 (1·52–8·05)	5·68 (2·49–11·4)	51·4 (-35·9–260)	6·46 (2·51–13·8)	10·1 (4·01–19)	56·1 (-38·3–294)
Mauritius	10·5 (3·8–21·3)	16·8 (5·83–36·7)	60·3 (-37·4–407)	4·43 (1·72–9·59)	8·72 (3·07–19·1)	97 (-22·8–478)	14·9 (5·39–31)	25·5 (8·9–55·3)	71·2 (-32·5–425)
Myanmar	1350 (501–2720)	2110 (729–4350)	56·6 (-44·3–343)	1880 (753–3970)	3730 (1650–7200)	98·5 (-15–380)	3230 (1250–6450)	5840 (2370–11000)	81 (-23·7–357)
Philippines	180 (65·6–391)	388 (128–851)	116 (-24·6–526)	287 (101–667)	799 (316–1710)	179 (11·6–727)	467 (164–1070)	1190 (446–2430)	154 (-2·2–601)

	Female			Male			Both		
	Number of deaths		Change (%)	Number of deaths		Change (%)	Number of deaths		Change (%)
	1990	2023		1990	2023		1990	2023	
Seychelles	0·794 (0·267–1·63)	0·8 (0·286–1·83)	0·7 (-65–202)	0·459 (0·173–1·06)	0·847 (0·301–1·84)	84·5 (-38·3–487)	1·25 (0·457–2·67)	1·65 (0·599–3·65)	31·4 (-50·9–288)
Sri Lanka	204 (75·8–423)	522 (174–1090)	156 (-5·2–604)	575 (242–1090)	1260 (564–2290)	120 (-3–403)	779 (321–1510)	1780 (752–3420)	129 (-4·6–439)
Thailand	795 (314–1600)	1210 (459–2410)	52·6 (-42–294)	268 (97–645)	569 (217–1260)	112 (-24·8–557)	1060 (411–2120)	1780 (676–3580)	67·6 (-34·7–345)
Timor-Leste	5·52 (2·01–11·9)	16 (5·24–36·1)	190 (-5·4–788)	1·87 (0·637–4·74)	5·09 (1·59–11·4)	172 (-6·6–817)	7·39 (2·68–16·3)	21·1 (7·09–44·5)	186 (-2·7–754)
Viet Nam	849 (291–1920)	966 (329–2150)	13·8 (-55·4–233)	191 (63·9–430)	288 (105–639)	50·9 (-45–372)	1040 (352–2340)	1250 (438–2940)	20·6 (-52·8–255)
**Sub-Saharan Africa**	1120 (394–2310)	2430 (873–5500)	117 (-19–513)	1210 (510–2540)	2530 (1080–5450)	109 (-17·6–450)	2330 (898–4930)	4950 (1940–10400)	113 (-16·7–450)
Central Sub-Saharan Africa	97·4 (33·6–216)	195 (61·4–470)	100 (-29·4–533)	69·6 (21·5–162)	190 (66–468)	173 (-11·1–810)	167 (57·3–367)	385 (130–878)	131 (-16·1–638)
Angola	5·61 (1·84–12·2)	16·6 (5·35–38·6)	195 (-2·1–830)	7·74 (2·38–19·2)	21·1 (6·43–52·2)	173 (-7·7–855)	13·4 (4·45–32)	37·7 (11·9–84·9)	182 (5·1–842)
Central African Republic	6·35 (2·09–14·7)	15·1 (4·25–35)	138 (-24·8–683)	2·73 (0·759–6·51)	5·74 (1·92–13·2)	110 (-29·4–635)	9·08 (3·01–21·6)	20·9 (6·58–46·1)	130 (-21·8–647)
Congo	4·44 (1·52–9·77)	10·6 (3·47–24·9)	138 (-18·9–712)	1·43 (0·461–3·28)	4·55 (1·49–11)	218 (7·1–1020)	5·87 (2·01–12·9)	15·1 (5·25–35·9)	158 (-6·5–750)
Democratic Republic of the Congo	79·4 (27·1–174)	150 (44–368)	88·8 (-33·1–484)	56·9 (17·8–132)	157 (56·2–392)	176 (-11–804)	136 (47·2–294)	307 (102–713)	125 (-20·1–616)
Equatorial Guinea	0·809 (0·27–1·77)	1·43 (0·473–3·1)	76·6 (-43·1–498)	0·514 (0·162–1·26)	1·17 (0·382–3·02)	128 (-27·9–748)	1·32 (0·444–3·04)	2·6 (0·856–5·96)	96·4 (-30·8–560)
Gabon	0·845 (0·285–1·93)	1·42 (0·446–3·29)	68·5 (-44·7–454)	0·302 (0·0999–0·737)	0·715 (0·246–1·64)	136 (-19·3–732)	1·15 (0·372–2·68)	2·14 (0·674–5·11)	86·3 (-35·2–506)
Eastern Sub-Saharan Africa	441 (171–910)	979 (393–2130)	122 (-12·6–487)	779 (344–1570)	1610 (736–3350)	106 (-15·4–396)	1220 (526–2460)	2580 (1120–5230)	112 (-15·3–407)
Burundi	17 (6·02–37·5)	34·5 (12–70·7)	103 (-30·6–588)	36 (13·5–74·6)	70·9 (27·9–177)	96·9 (-31·1–514)	53 (19·5–109)	105 (38·4–240)	99 (-30·7–520)
Comoros	2·58 (0·986–5·32)	3·54 (1·35–7·78)	37·2 (-48·4–288)	3·44 (1·39–7·3)	5·55 (2·22–11·3)	61·5 (-37·5–353)	6·02 (2·4–12·6)	9·09 (3·47–17·9)	51·1 (-38·6–288)

	Female			Male			Both		
	Number of deaths		Change (%)	Number of deaths		Change (%)	Number of deaths		Change (%)
	1990	2023		1990	2023		1990	2023	
Djibouti	0·49 (0·162–1·11)	1·6 (0·545–3·49)	226 (11·4–860)	0·845 (0·336–1·85)	3·19 (1·07–6·92)	277 (44·6–1040)	1·34 (0·507–3·15)	4·79 (1·61–10·2)	258 (31·4–973)
Eritrea	1·68 (0·53–3·83)	4·13 (1·34–10·5)	146 (-18–681)	16·1 (5·59–37·3)	37·9 (13·9–84·4)	135 (-16·8–619)	17·8 (6·34–41)	42 (15·5–94·2)	136 (-15·9–600)
Ethiopia	18·9 (5·78–46·7)	67·7 (23·9–143)	258 (8·7–1150)	116 (42·3–260)	362 (142–860)	211 (3·6–933)	135 (49·3–311)	430 (163–1000)	218 (7·3–871)
Kenya	35·9 (13·1–76·4)	87·6 (30·6–195)	144 (-10·7–640)	41·9 (16·2–94·2)	99·9 (34·8–232)	139 (-16–588)	77·7 (30·7–168)	187 (63·2–411)	141 (-13·7–651)
Madagascar	151 (63·2–289)	358 (169–697)	137 (4–464)	417 (194–795)	730 (388–1340)	75 (-14·2–292)	568 (263–1080)	1090 (577–1900)	91·4 (-10–308)
Malawi	33·3 (11·7–72·3)	54·8 (19·8–127)	64·7 (-38·1–382)	14·7 (5·22–35·5)	25·9 (8·47–60·8)	75·6 (-38·5–480)	48 (17·2–105)	80·7 (28·3–188)	68·1 (-36·6–420)
Mozambique	29·1 (10–65·4)	46·4 (14·1–106)	59·5 (-46·1–379)	30 (10·1–72·2)	51·8 (17·4–123)	72·4 (-42·8–399)	59·1 (20·2–129)	98·1 (32·7–218)	66·1 (-40·2–379)
Rwanda	41·2 (14·3–86·6)	65·7 (23–137)	59·4 (-39·8–395)	6·89 (2·26–16)	12·4 (4·44–28)	79·5 (-37·4–505)	48·1 (17·2–103)	78 (27·5–160)	62·3 (-37·3–389)
Somalia	5·34 (1·67–13·4)	20·9 (7·04–48·1)	292 (31·4–1200)	7·2 (2·48–16·1)	22·3 (7·2–55·7)	210 (4–971)	12·5 (4·38–30·2)	43·2 (14·5–98·2)	244 (19·6–1080)
South Sudan	11·6 (4·13–24·4)	22·6 (7·9–50)	95·4 (-32·2–464)	23·8 (8·53–56·7)	32·9 (11·4–75·3)	38·6 (-51·9–348)	35·3 (12·9–82·3)	55·5 (18·9–122)	57·2 (-45·3–381)
Uganda	48·6 (17·2–105)	106 (35·6–252)	118 (-18·9–616)	18·3 (6·5–39·7)	44·5 (15·4–105)	143 (-19·6–668)	66·9 (24·5–132)	151 (52·5–357)	125 (-19·1–610)
United Republic of Tanzania	31·8 (11–72·3)	66·3 (21·1–151)	108 (-27·6–544)	36·5 (12·8–83·3)	82·1 (29·1–185)	125 (-21·1–577)	68·3 (24·7–155)	148 (50·5–328)	117 (-22·6–591)
Zambia	12 (3·5–30·5)	37·9 (12·4–87·4)	215 (-1·3–961)	9·18 (3·28–20·8)	23·8 (7·88–55·2)	160 (-7–813)	21·2 (7·01–47·4)	61·8 (21·4–137)	191 (-1·5–799)
Southern Sub-Saharan Africa	241 (86–496)	528 (173–1200)	119 (-20·6–542)	60·7 (23·4–139)	106 (37·2–229)	74·6 (-37·9–435)	302 (107–633)	634 (210–1450)	110 (-22·9–502)
Botswana	14 (5·53–28)	26·2 (10·2–54·3)	87·2 (-25·9–401)	3·64 (1·31–8·2)	9·39 (3·53–20·8)	158 (-7·7–676)	17·7 (7·06–35·8)	35·6 (13·7–73·2)	102 (-17·7–446)
Eswatini	1·23 (0·414–2·61)	2·78 (0·909–6·12)	126 (-27·5–646)	1·79 (0·659–4·17)	2·15 (0·742–5·17)	19·8 (-58·6–280)	3·02 (1·11–6·96)	4·93 (1·71–10·5)	63 (-40·9–409)

	Female			Male			Both		
	Number of deaths		Change (%)	Number of deaths		Change (%)	Number of deaths		Change (%)
	1990	2023		1990	2023		1990	2023	
Lesotho	7·77 (2·54–17·1)	13·3 (4·18–32·8)	70·7 (-40·1–433)	2·67 (0·947–6·19)	3·99 (1·3–8·65)	49·6 (-50–398)	10·4 (3·54–22·9)	17·3 (5·83–40·4)	65·3 (-43–414)
Namibia	6·03 (2·1–13·2)	12·6 (3·89–26·5)	108 (-24·8–501)	2·32 (0·756–5·58)	3·83 (1·35–8·71)	65·4 (-44·1–436)	8·34 (2·91–18·7)	16·4 (5·35–33·3)	96·5 (-27·3–463)
South Africa	192 (67–392)	410 (132–947)	113 (-23·3–511)	43·9 (16·9–100)	76·4 (26·5–165)	73·9 (-40·3–444)	236 (83·8–493)	486 (158–1100)	106 (-24·2–504)
Zimbabwe	20 (6·44–41·5)	63·4 (20·6–135)	217 (11·2–878)	6·38 (2·26–14·5)	10·3 (3·67–25·9)	60·8 (-43–437)	26·3 (8·84–56·1)	73·6 (24·5–153)	180 (-1·9–754)
Western Sub-Saharan Africa	338 (109–735)	727 (253–1640)	115 (-29·7–572)	300 (109–695)	623 (241–1470)	108 (-29·2–556)	637 (219–1480)	1350 (488–2960)	112 (-20·5–545)
Benin	11·6 (3·72–25·4)	22·6 (7·61–53·6)	94·5 (-35·3–508)	6·35 (2·19–15·6)	13 (4·37–30·3)	105 (-35·9–551)	18 (5·91–38·8)	35·6 (11·9–81·9)	98 (-25·1–501)
Burkina Faso	16 (5·37–34·7)	34·2 (11·8–71·6)	113 (-31·9–569)	20·2 (7·37–46·7)	31·4 (11·4–74·2)	55·4 (-49·1–425)	36·3 (13–81·2)	65·6 (24·2–140)	80·8 (-36·4–453)
Cabo Verde	0·541 (0·189–1·19)	0·552 (0·166–1·25)	1·9 (-67·2–230)	0·278 (0·0952–0·684)	0·427 (0·138–0·926)	53·7 (-48·5–401)	0·819 (0·272–1·83)	0·978 (0·32–2·08)	19·5 (-57·9–291)
Cameroon	54 (17·5–142)	161 (58·9–353)	199 (-3·4–771)	14·8 (5·16–32·3)	40·5 (13·5–97·6)	174 (-17·2–829)	68·8 (22·5–173)	202 (73·7–460)	193 (-1·4–758)
Chad	23·7 (7·7–50·3)	50·2 (16·1–119)	111 (-28–593)	25·2 (9·16–59·9)	61·5 (23·4–142)	144 (-17·1–661)	49 (16·6–105)	112 (39·9–248)	128 (-14·1–611)
Côte d'Ivoire	12·6 (4·25–29·8)	31·1 (10·4–68·3)	147 (-19–686)	5·44 (1·72–12·3)	17·4 (6·02–42·2)	220 (7·1–957)	18 (5·94–42·7)	48·5 (16·1–111)	169 (-8–755)
Gambia	0·921 (0·313–2·08)	3·96 (1·17–9·37)	329 (24·2–1330)	0·646 (0·201–1·6)	1·78 (0·605–4·49)	175 (-12·7–852)	1·57 (0·502–3·73)	5·74 (1·92–13·6)	266 (22·1–1100)
Ghana	21·1 (6·63–47·9)	52·5 (16·1–128)	149 (-16·5–818)	38·8 (11·8–92·6)	84·2 (26·3–210)	117 (-25·8–620)	59·9 (19·4–142)	137 (43–329)	128 (-17·2–656)
Guinea	14·7 (4·65–32)	24·5 (7·86–57)	66·3 (-46·3–439)	13·8 (4·33–35·9)	20·9 (7·11–51·5)	51·8 (-48·7–434)	28·5 (9·71–64·2)	45·4 (15·5–101)	59·3 (-42–423)
Guinea-Bissau	1·88 (0·601–4·18)	4·15 (1·32–10)	120 (-28·2–619)	1·2 (0·383–2·9)	1·96 (0·613–4·62)	62·8 (-44·9–439)	3·08 (0·982–7·19)	6·11 (2·06–14·3)	98 (-29·5–500)
Liberia	4·46 (1·46–9·86)	8·47 (2·64–21·4)	90 (-42·5–495)	6·12 (1·94–15·8)	10·4 (3·51–24·3)	69·4 (-47·2–489)	10·6 (3·45–25·6)	18·8 (6·5–44·2)	78·1 (-41·4–507)

	Female			Male			Both		
	Number of deaths		Change (%)	Number of deaths		Change (%)	Number of deaths		Change (%)
	1990	2023		1990	2023		1990	2023	
Mali	13·4 (4·16–29·3)	28·1 (8·58–67·9)	110 (-32·1–565)	33·8 (13·9–76·7)	77·3 (30·4–178)	129 (-18·6–548)	47·2 (18·1–113)	105 (38·9–243)	123 (-15·9–548)
Mauritania	4·25 (1·42–9·65)	7·89 (2·46–19·8)	85·8 (-40·3–471)	3·39 (1·22–8·1)	6·87 (2·26–17·5)	103 (-38·8–550)	7·64 (2·57–18)	14·8 (4·93–34·1)	93·4 (-34·3–465)
Niger	12·2 (3·69–28)	38·4 (11·2–98·5)	215 (-6·2–915)	38·5 (15·1–88·5)	105 (39·4–221)	172 (-3·6–718)	50·7 (18·8–116)	143 (54·8–296)	182 (-4·7–719)
Nigeria	105 (32–236)	163 (54·8–397)	55·7 (-52·8–423)	66·7 (22·7–155)	108 (36·3–281)	61·7 (-49·7–483)	171 (55·4–393)	271 (95·6–624)	58·1 (-45·9–400)
Sao Tome and Principe	0·13 (0·0422–0·283)	0·142 (0·0418–0·355)	9·8 (-66·3–255)	0·117 (0·0371–0·277)	0·264 (0·0871–0·622)	126 (-26·8–613)	0·246 (0·0788–0·547)	0·406 (0·137–0·912)	64·9 (-46·4–413)
Senegal	14·3 (4·55–32·9)	43·8 (14·3–99·8)	207 (4–858)	5·12 (1·66–13)	10·7 (3·71–25·8)	109 (-32·5–583)	19·4 (6·12–45·1)	54·5 (17·8–124)	181 (1·5–794)
Sierra Leone	18·3 (6·46–44·2)	25·6 (8·26–56·2)	39·9 (-52·9–349)	13·7 (4·31–30·8)	19·5 (6·29–46·1)	42·2 (-52·3–372)	32 (10·7–76·2)	45·1 (15–98·1)	40·9 (-48·9–341)
Togo	8·48 (2·85–18·2)	25·8 (8·29–63·5)	204 (0·1–804)	5·56 (1·75–13·4)	12·4 (3·76–31)	124 (-22·7–616)	14 (4·61–32·5)	38·2 (12·3–89·7)	172 (-4–769)

Appendix Table 2·7: Age-standardized death rate attributable to chewing tobacco use in 1990 and 2023, among ages 15+

	Female			Male			Both		
	Age-standardized death rate (per 100,000)		Change (%)	Age-standardized death rate (per 100,000)		Change (%)	Age-standardized death rate (per 100,000)		Change (%)
	1990	2023		1990	2023		1990	2023	
Global	2·9 (1·5–5·3)	2·8 (1·5–4·7)	-1·2 (-49·4–77·3)	5·2 (2·7–8·7)	5 (3·2–7·7)	-3·1 (-42·3–73·3)	4 (2–6·4)	3·9 (2·3–6)	-2·5 (-44·3–73·5)
**Central Europe, Eastern Europe, and Central Asia**	0·3 (0·1–0·7)	0·1 (0–0·3)	-46·6 (-81·3–74·3)	0·7 (0·2–1·5)	0·5 (0·2–1)	-31·2 (-75·1–108·5)	0·5 (0·1–1)	0·3 (0·1–0·6)	-35·9 (-76·4–104·9)
Central Asia	0·3 (0·1–0·8)	0·2 (0·1–0·4)	-42·3 (-80–85·2)	2·1 (0·8–4·6)	1·1 (0·4–2·3)	-48·9 (-80·7–42·1)	1·2 (0·4–2·2)	0·6 (0·2–1·2)	-47·4 (-78·8–60)
Armenia	0·1 (0–0·4)	0·1 (0–0·1)	-62·3 (-87·3–15·9)	0·2 (0·1–0·5)	0·1 (0–0·2)	-57·1 (-84·4–29·1)	0·2 (0·1–0·4)	0·1 (0–0·2)	-59·7 (-85·7–26·3)
Azerbaijan	0·1 (0–0·3)	0·1 (0–0·2)	-43·1 (-80·4–83·1)	0·6 (0·2–1·5)	0·3 (0·1–0·7)	-55·1 (-84·7–35·3)	0·4 (0·1–0·7)	0·2 (0·1–0·4)	-52·1 (-80·3–75·1)
Georgia	0·2 (0·1–0·6)	0·2 (0·1–0·4)	-30·7 (-75·1–116·5)	0·4 (0·1–0·8)	0·3 (0·1–0·6)	-19·7 (-70·2–143·4)	0·3 (0·1–0·7)	0·2 (0·1–0·5)	-24·2 (-72·7–134·8)
Kazakhstan	0·2 (0·1–0·5)	0·1 (0–0·2)	-55·6 (-84·9–44·3)	0·9 (0·4–2·1)	0·4 (0·1–0·9)	-54·7 (-83·4–35·7)	0·6 (0·2–1)	0·3 (0·1–0·5)	-54·7 (-83·6–43·1)
Kyrgyzstan	0·8 (0·2–1·8)	0·2 (0·1–0·5)	-72·9 (-90·5–-11·3)	11·9 (4·6–24·5)	4 (1·5–8·2)	-66·3 (-86·6–-9·6)	6·1 (1·9–10)	2·1 (0·7–3·6)	-66·4 (-85·6–-2·4)
Mongolia	0·4 (0·1–0·8)	0·1 (0–0·3)	-65 (-88·2–8·8)	1·5 (0·5–3·5)	0·6 (0·2–1·3)	-61 (-85·7–28·5)	0·9 (0·3–1·9)	0·4 (0·1–0·7)	-62·4 (-86·7–29·3)
Tajikistan	0·7 (0·2–1·6)	0·8 (0·2–1·8)	10·4 (-61·6–272·3)	0·7 (0·2–1·6)	0·5 (0·2–1·1)	-31·5 (-76·2–107·1)	0·7 (0·2–1·6)	0·6 (0·2–1·5)	-10·7 (-68·1–184·9)
Turkmenistan	0·2 (0·1–0·6)	0·2 (0·1–0·4)	-29·8 (-76·7–121·1)	0·5 (0·2–1·1)	0·3 (0·1–0·8)	-38·5 (-79–81·4)	0·4 (0·1–0·8)	0·2 (0·1–0·5)	-34·6 (-77·5–109·4)
Uzbekistan	0·4 (0·1–1)	0·2 (0·1–0·5)	-44·5 (-81·3–73·8)	3·7 (1·4–8)	2 (0·7–4·4)	-44·9 (-79·5–58·7)	2 (0·7–3·9)	1·1 (0·4–2·3)	-44·5 (-78·6–70·2)
Central Europe	0·3 (0·1–0·6)	0·2 (0–0·4)	-40·6 (-79–99·4)	0·5 (0·2–1·2)	0·4 (0·1–1)	-17·5 (-70–150·5)	0·4 (0·1–0·9)	0·3 (0·1–0·6)	-25·5 (-73·2–137·2)
Albania	0·3 (0·1–0·6)	0·2 (0–0·4)	-32·3 (-76·7–118·7)	0·5 (0·2–1·1)	0·6 (0·2–1·3)	14·4 (-60·8–230·6)	0·4 (0·1–0·9)	0·4 (0·1–0·8)	-3·7 (-68·2–177·1)
Bosnia and Herzegovina	0·3 (0·1–0·6)	0·2 (0·1–0·5)	-23 (-72·8–148·7)	0·4 (0·1–1)	0·4 (0·1–0·9)	-8·5 (-67·2–181·1)	0·4 (0·1–0·8)	0·3 (0·1–0·6)	-14·7 (-68·6–172·9)
Bulgaria	0·5 (0·2–1·2)	0·4 (0·1–1)	-11·8 (-68·4–192·9)	1·1 (0·4–2·6)	1·1 (0·4–2·5)	-0·7 (-64–204·8)	0·8 (0·2–1·8)	0·8 (0·2–1·6)	-4·8 (-65·4–198·9)

	Female			Male			Both		
	Age-standardized death rate (per 100,000)		Change (%)	Age-standardized death rate (per 100,000)		Change (%)	Age-standardized death rate (per 100,000)		Change (%)
	1990	2023		1990	2023		1990	2023	
Croatia	0·2 (0·1–0·4)	0·1 (0–0·2)	-59·7 (-86–34·6)	0·4 (0·2–0·9)	0·2 (0·1–0·5)	-45·7 (-80·1–65·8)	0·3 (0·1–0·6)	0·1 (0–0·3)	-50·5 (-82·2–57)
Czechia	0·3 (0·1–0·7)	0·1 (0–0·2)	-64·8 (-87·5–16·5)	0·6 (0·2–1·4)	0·3 (0·1–0·7)	-48·4 (-80·6–59·3)	0·5 (0·1–1)	0·2 (0·1–0·4)	-53·4 (-82·3–54·2)
Hungary	0·2 (0·1–0·5)	0·1 (0–0·2)	-63·1 (-86·9–20·3)	0·4 (0·2–1)	0·2 (0·1–0·4)	-53·1 (-83–47·6)	0·3 (0·1–0·7)	0·1 (0–0·3)	-56·4 (-84·6–38·8)
Montenegro	0·3 (0·1–0·8)	0·3 (0·1–0·6)	-21 (-72·8–137·7)	0·5 (0·2–1·2)	0·5 (0·2–1·1)	-10·4 (-67·1–170·5)	0·4 (0·1–1)	0·4 (0·1–0·8)	-14·7 (-68·5–165)
North Macedonia	0·4 (0·1–0·9)	0·3 (0·1–0·7)	-19 (-72·2–160·6)	0·6 (0·2–1·4)	0·6 (0·2–1·2)	-9 (-65·2–190·6)	0·5 (0·2–1·2)	0·4 (0·1–1)	-13·1 (-68·2–173·7)
Poland	0·2 (0·1–0·5)	0·1 (0–0·3)	-33·8 (-75·8–125·6)	0·4 (0·2–0·9)	0·4 (0·1–0·9)	-2·3 (-64·1–202·3)	0·3 (0·1–0·7)	0·3 (0·1–0·6)	-13·9 (-68·1–181·1)
Romania	0·2 (0·1–0·6)	0·1 (0–0·3)	-46·7 (-80·9–74·2)	0·5 (0·2–1·2)	0·5 (0·2–1·2)	2·9 (-62·3–219·9)	0·4 (0·1–0·8)	0·3 (0·1–0·7)	-14·6 (-70·1–163·6)
Serbia	0·5 (0·1–1·1)	0·3 (0·1–0·7)	-35·8 (-78–104·7)	0·7 (0·2–1·7)	0·6 (0·2–1·3)	-25 (-74–132·9)	0·6 (0·2–1·4)	0·4 (0·1–0·9)	-28·9 (-75·6–112·5)
Slovakia	0·1 (0–0·4)	0·1 (0–0·1)	-62·1 (-87–16·4)	0·4 (0·1–0·8)	0·2 (0·1–0·4)	-47·6 (-81·5–63·8)	0·3 (0·1–0·6)	0·1 (0–0·3)	-51·8 (-83·2–57·6)
Slovenia	0·2 (0–0·4)	0·1 (0–0·2)	-42·9 (-80·2–81·4)	0·4 (0·2–1)	0·3 (0·1–0·8)	-21·1 (-69·7–148·1)	0·3 (0·1–0·6)	0·2 (0·1–0·5)	-25·5 (-71·7–135·9)
Eastern Europe	0·3 (0·1–0·6)	0·1 (0–0·3)	-52·4 (-83·2–55·7)	0·5 (0·2–1·1)	0·3 (0·1–0·7)	-39·2 (-77·8–88·5)	0·4 (0·1–0·8)	0·2 (0·1–0·5)	-44·5 (-79·4–83·4)
Belarus	0·2 (0·1–0·4)	0·1 (0–0·2)	-41·7 (-79·9–92·1)	0·4 (0·1–0·8)	0·4 (0·1–0·8)	-1·2 (-63·7–221·4)	0·3 (0·1–0·6)	0·2 (0·1–0·5)	-15·9 (-69·6–179·2)
Estonia	0·2 (0·1–0·4)	0·1 (0–0·1)	-72 (-90·2–-7)	0·4 (0·1–0·8)	0·4 (0·1–0·8)	2·4 (-60·4–214·1)	0·3 (0·1–0·6)	0·2 (0·1–0·4)	-24·8 (-70·9–142·2)
Latvia	0·2 (0·1–0·5)	0·1 (0–0·3)	-47·6 (-81·8–65·7)	0·4 (0·1–0·8)	0·2 (0·1–0·5)	-30·5 (-75·3–112·6)	0·3 (0·1–0·6)	0·2 (0·1–0·4)	-37·8 (-78–97·5)
Lithuania	0·1 (0–0·3)	0·1 (0–0·2)	-38·5 (-78·7–98·9)	0·2 (0·1–0·5)	0·2 (0·1–0·4)	-11·8 (-67·5–180·7)	0·2 (0·1–0·4)	0·1 (0–0·3)	-21·8 (-71·9–151·2)
Republic of Moldova	0·2 (0·1–0·4)	0·1 (0–0·2)	-64 (-87·4–20·9)	0·5 (0·2–1·1)	0·3 (0·1–0·7)	-33·5 (-76·2–101·3)	0·3 (0·1–0·7)	0·2 (0·1–0·4)	-42·2 (-79·9–79·8)
Russian Federation	0·3 (0·1–0·7)	0·2 (0–0·4)	-51·5 (-82·8–56·2)	0·6 (0·2–1·2)	0·3 (0·1–0·7)	-44·2 (-79·5–72·8)	0·4 (0·1–0·9)	0·2 (0·1–0·5)	-47·1 (-79·9–78)

	Female			Male			Both		
	Age-standardized death rate (per 100,000)		Change (%)	Age-standardized death rate (per 100,000)		Change (%)	Age-standardized death rate (per 100,000)		Change (%)
	1990	2023		1990	2023		1990	2023	
Ukraine	0·2 (0·1–0·5)	0·1 (0–0·2)	-61·7 (-87–24·5)	0·4 (0·1–0·9)	0·3 (0·1–0·6)	-38 (-77·7–87·5)	0·3 (0·1–0·7)	0·2 (0·1–0·3)	-47·1 (-81·3–65·7)
**High-income**	0·1 (0–0·3)	0·1 (0–0·2)	-45·9 (-80·8–76·2)	1·3 (0·8–2)	0·6 (0·4–1)	-54·6 (-71·6–-27·2)	0·7 (0·3–1)	0·3 (0·2–0·5)	-53·3 (-69·1–-2·9)
Australasia	0·1 (0–0·3)	0·1 (0–0·1)	-57·5 (-85–36·8)	0·6 (0·2–1·2)	0·3 (0·1–0·6)	-55·5 (-83·2–32·6)	0·3 (0·1–0·7)	0·2 (0·1–0·3)	-55·9 (-82·3–45·2)
Australia	0·1 (0–0·3)	0 (0–0·1)	-60·1 (-85·8–27·8)	0·6 (0·2–1·3)	0·3 (0·1–0·6)	-56·4 (-83·6–29·4)	0·3 (0·1–0·7)	0·1 (0·1–0·3)	-57·3 (-82·7–40·1)
New Zealand	0·1 (0–0·3)	0·1 (0–0·2)	-42·7 (-80·3–83·4)	0·5 (0·2–1·1)	0·3 (0·1–0·6)	-49·3 (-80·8–54·5)	0·3 (0·1–0·6)	0·2 (0·1–0·4)	-47·5 (-79·2–78·3)
High-income Asia Pacific	0·2 (0·1–0·5)	0·1 (0–0·2)	-61·6 (-86·1–28)	0·9 (0·3–2·1)	0·4 (0·1–0·9)	-53·5 (-82·8–38·9)	0·5 (0·2–1·1)	0·2 (0·1–0·5)	-55 (-82·5–46·4)
Brunei Darussalam	1 (0·4–2·5)	0·5 (0·2–1·2)	-48·3 (-82·6–63·4)	1·5 (0·5–3·7)	0·8 (0·3–1·8)	-46·9 (-81·7–78)	1·3 (0·4–3·1)	0·7 (0·2–1·5)	-47·5 (-81·7–59·7)
Japan	0·2 (0–0·4)	0·1 (0–0·2)	-51·3 (-82–62·5)	0·8 (0·3–1·8)	0·4 (0·2–1)	-43·5 (-79·4–71·1)	0·5 (0·1–0·9)	0·3 (0·1–0·5)	-45·2 (-78·4–82·8)
Republic of Korea	0·4 (0·1–1·1)	0·1 (0–0·2)	-82·4 (-93·6–-44·6)	1·9 (0·7–4·6)	0·4 (0·1–0·8)	-79·7 (-92·8–-38·3)	1·2 (0·3–2·3)	0·2 (0·1–0·4)	-80·1 (-92·2–-32·4)
Singapore	0·2 (0·1–0·5)	0·1 (0–0·1)	-72·7 (-90·1–-13·7)	0·9 (0·3–1·9)	0·3 (0·1–0·6)	-68·8 (-87·5–-3·7)	0·5 (0·2–1·1)	0·2 (0·1–0·4)	-69 (-87·5–-0·6)
High-income North America	0·2 (0·1–0·5)	0·1 (0–0·3)	-33·5 (-76·8–105·5)	3·5 (2·3–5·1)	1·5 (0·9–2·3)	-57·1 (-71·1–-36)	1·8 (0·9–2·2)	0·8 (0·5–1·2)	-54·9 (-66·5–-16·2)
Canada	0·2 (0·1–0·5)	0·1 (0–0·3)	-41·8 (-79·7–84·1)	2·2 (0·9–4·4)	0·7 (0·3–1·5)	-68·1 (-87·5–-10·4)	1·2 (0·4–2)	0·4 (0·1–0·8)	-65·7 (-85·2–11·4)
Greenland	1 (0·3–2·2)	0·4 (0·1–1)	-56·1 (-85–48·2)	9·3 (3·6–20·5)	3·8 (1·4–8·5)	-59·2 (-85·4–22·5)	5·6 (1·6–9·4)	2·2 (0·8–4·7)	-60·2 (-82·3–47·2)
United States of America	0·2 (0·1–0·5)	0·1 (0–0·3)	-32·3 (-76·3–105·9)	3·6 (2·4–5·2)	1·6 (1–2·5)	-55·8 (-69·6–-34·1)	1·9 (1–2·2)	0·9 (0·5–1·2)	-53·5 (-64·9–-17)
Southern Latin America	0·1 (0–0·3)	0 (0–0·1)	-63 (-87·1–21·9)	0·2 (0·1–0·5)	0·1 (0–0·2)	-61·9 (-86·3–19)	0·2 (0·1–0·4)	0·1 (0–0·1)	-62·3 (-86·7–21·4)
Argentina	0·1 (0–0·3)	0 (0–0·1)	-63·1 (-86·9–22·9)	0·2 (0·1–0·4)	0·1 (0–0·2)	-58 (-84·8–30·8)	0·2 (0·1–0·4)	0·1 (0–0·1)	-60·2 (-86·1–27·4)
Chile	0·1 (0–0·3)	0 (0–0·1)	-64·3 (-87·4–15)	0·3 (0·1–0·7)	0·1 (0–0·2)	-71·3 (-89·6–-10·7)	0·2 (0·1–0·5)	0·1 (0–0·2)	-68·9 (-88·8–-0·4)
Uruguay	0·2 (0·1–0·4)	0·1 (0–0·2)	-55 (-84·5–41·8)	0·3 (0·1–0·6)	0·1 (0–0·3)	-53·1 (-82·9–49·8)	0·2 (0·1–0·5)	0·1 (0–0·2)	-54 (-83·3–45·6)

	Female			Male			Both		
	Age-standardized death rate (per 100,000)		Change (%)	Age-standardized death rate (per 100,000)		Change (%)	Age-standardized death rate (per 100,000)		Change (%)
	1990	2023		1990	2023		1990	2023	
Western Europe	0·1 (0–0·2)	0 (0–0·1)	-48·4 (-81·7–73·6)	0·2 (0·1–0·4)	0·1 (0–0·3)	-36·4 (-77·2–91·4)	0·1 (0–0·3)	0·1 (0–0·2)	-39·6 (-78·1–93·5)
Andorra	0 (0–0·1)	0 (0–0·1)	-40·7 (-81–98)	0·1 (0–0·3)	0·1 (0–0·2)	-16 (-72·2–175·4)	0·1 (0–0·2)	0·1 (0–0·1)	-23 (-74·7–141·6)
Austria	0·1 (0–0·2)	0 (0–0·1)	-47·7 (-81·6–72)	0·2 (0·1–0·5)	0·2 (0·1–0·4)	-14·4 (-69–154·3)	0·1 (0–0·3)	0·1 (0–0·2)	-22·5 (-72–144·2)
Belgium	0·1 (0–0·2)	0 (0–0·1)	-57·8 (-85·1–40·2)	0·2 (0·1–0·3)	0·1 (0–0·2)	-52 (-83·1–49·2)	0·1 (0–0·2)	0·1 (0–0·1)	-53·8 (-83·2–53·5)
Cyprus	0·1 (0–0·3)	0 (0–0·1)	-67·2 (-89·7–4·8)	0·3 (0·1–0·6)	0·1 (0–0·2)	-58·1 (-84·6–38)	0·2 (0·1–0·4)	0·1 (0–0·2)	-61·4 (-87–19·6)
Denmark	0·1 (0–0·2)	0·1 (0–0·2)	-15·4 (-69·5–174·9)	0·4 (0·2–0·9)	0·3 (0·1–0·6)	-28·7 (-74·1–105·1)	0·3 (0·1–0·5)	0·2 (0·1–0·4)	-25·7 (-69·6–147·3)
Finland	0·1 (0–0·2)	0 (0–0·1)	-47·8 (-81·3–66·8)	0·1 (0–0·3)	0·1 (0–0·2)	-14·4 (-68·6–158·3)	0·1 (0–0·2)	0·1 (0–0·2)	-26·3 (-72·4–136·3)
France	0·1 (0–0·1)	0 (0–0·1)	-46·7 (-81–77·8)	0·2 (0·1–0·4)	0·1 (0–0·2)	-57·8 (-85–28·8)	0·1 (0–0·2)	0·1 (0–0·1)	-55·4 (-83·9–45·4)
Germany	0·1 (0–0·2)	0·1 (0–0·1)	-33·4 (-76·3–120·4)	0·2 (0·1–0·4)	0·2 (0·1–0·4)	-3·5 (-64·7–200·5)	0·1 (0–0·3)	0·1 (0–0·2)	-11·8 (-67·3–190·1)
Greece	0·1 (0–0·4)	0 (0–0·1)	-68 (-88·9–7·1)	0·2 (0·1–0·5)	0·1 (0–0·2)	-57·4 (-84·7–31·5)	0·2 (0·1–0·4)	0·1 (0–0·2)	-61·8 (-86·5–21·6)
Iceland	0·1 (0–0·2)	0·1 (0–0·2)	-19·1 (-71·8–180·7)	0·4 (0·2–0·9)	0·5 (0·2–1)	15·8 (-55·7–242·1)	0·3 (0·1–0·5)	0·3 (0·1–0·6)	11·8 (-56·6–253·2)
Ireland	0·1 (0–0·2)	0 (0–0·1)	-59·2 (-85·2–38·3)	0·1 (0·1–0·3)	0·1 (0–0·2)	-49·4 (-80·9–52·8)	0·1 (0–0·3)	0·1 (0–0·1)	-53·1 (-82·6–50·7)
Israel	0·1 (0–0·1)	0 (0–0·1)	-54·7 (-84·3–47·3)	0·1 (0–0·3)	0·1 (0–0·2)	-43 (-78·7–70·1)	0·1 (0–0·2)	0 (0–0·1)	-46·7 (-80·7–67·3)
Italy	0·1 (0–0·2)	0 (0–0·1)	-55·3 (-84·3–50·2)	0·2 (0·1–0·4)	0·1 (0–0·2)	-48·2 (-80·9–52·9)	0·1 (0–0·3)	0·1 (0–0·1)	-50 (-81·8–59·9)
Luxembourg	0·1 (0–0·3)	0 (0–0·1)	-71·1 (-89·9–-6·3)	0·2 (0·1–0·5)	0·1 (0–0·2)	-63·6 (-86·2–9·4)	0·2 (0·1–0·4)	0·1 (0–0·1)	-65·8 (-87·6–8)
Malta	0·1 (0–0·2)	0 (0–0·1)	-66·9 (-88·1–10)	0·2 (0·1–0·4)	0·1 (0–0·1)	-59·3 (-84·8–24·8)	0·1 (0–0·3)	0 (0–0·1)	-61 (-85·7–22·4)
Monaco	0·1 (0–0·3)	0·1 (0–0·1)	-58·5 (-87·7–38·3)	0·3 (0·1–0·7)	0·2 (0·1–0·3)	-45·2 (-83·1–65)	0·2 (0·1–0·5)	0·1 (0–0·2)	-48·6 (-82·7–66·6)
Netherlands	0·1 (0–0·2)	0 (0–0·1)	-38·6 (-78·1–103·3)	0·1 (0–0·3)	0·1 (0–0·2)	-31·2 (-74·7–106·8)	0·1 (0–0·2)	0·1 (0–0·1)	-33·7 (-75–113·3)
Norway	0·1 (0–0·3)	0·1 (0–0·2)	-31·4 (-75·5–128·3)	0·4 (0·1–0·8)	0·4 (0·2–0·9)	16·5 (-55·7–246·1)	0·2 (0·1–0·5)	0·3 (0·1–0·5)	6·8 (-58·3–239·2)

	Female			Male			Both		
	Age-standardized death rate (per 100,000)		Change (%)	Age-standardized death rate (per 100,000)		Change (%)	Age-standardized death rate (per 100,000)		Change (%)
	1990	2023		1990	2023		1990	2023	
Portugal	0·2 (0·1–0·4)	0 (0–0·1)	-77·3 (-92–-26·3)	0·3 (0·1–0·7)	0·1 (0–0·3)	-63·1 (-86·4–16·5)	0·2 (0·1–0·5)	0·1 (0–0·2)	-68·7 (-88·5–1·1)
San Marino	0·1 (0–0·2)	0 (0–0·1)	-57·1 (-86·7–44·4)	0·2 (0·1–0·5)	0·1 (0–0·2)	-60·2 (-86·2–24·8)	0·1 (0–0·3)	0·1 (0–0·1)	-59·3 (-85·1–35)
Spain	0·1 (0–0·2)	0 (0–0·1)	-69 (-88·8–4·5)	0·2 (0·1–0·4)	0·1 (0–0·2)	-62·6 (-86·9–15·6)	0·1 (0–0·3)	0 (0–0·1)	-64·6 (-87·5–13·5)
Sweden	0·1 (0–0·2)	0·1 (0–0·2)	28·9 (-53·6–315·8)	0·5 (0·2–1·1)	0·5 (0·2–1)	-4·9 (-64–176·4)	0·3 (0·1–0·6)	0·3 (0·1–0·6)	1·1 (-62·2–198·4)
Switzerland	0·1 (0–0·1)	0 (0–0·1)	-52·2 (-82·8–62·6)	0·2 (0·1–0·4)	0·1 (0–0·2)	-46·1 (-80·9–58·6)	0·1 (0–0·2)	0·1 (0–0·1)	-47·5 (-80·7–68·1)
United Kingdom	0·1 (0–0·2)	0 (0–0·1)	-48·7 (-81·8–71·3)	0·1 (0·1–0·3)	0·1 (0–0·2)	-34·7 (-75·8–102·3)	0·1 (0–0·2)	0·1 (0–0·1)	-39·9 (-77·8–92·2)
**Latin America and Caribbean**	0·4 (0·1–0·8)	0·2 (0·1–0·4)	-52·1 (-83·1–56)	0·6 (0·2–1·3)	0·4 (0·1–0·8)	-35·3 (-76·4–99·4)	0·5 (0·2–1·1)	0·3 (0·1–0·6)	-42 (-79·5–80·7)
Andean Latin America	0·1 (0–0·3)	0·1 (0–0·1)	-48·6 (-83·2–64)	0·2 (0·1–0·4)	0·1 (0–0·2)	-33·1 (-76·1–113·8)	0·1 (0–0·3)	0·1 (0–0·2)	-39·7 (-80–88·2)
Bolivia (Plurinational State of)	0·2 (0·1–0·4)	0·1 (0–0·2)	-51 (-84–58·2)	0·4 (0·1–0·9)	0·3 (0·1–0·7)	-21·2 (-76·6–175·4)	0·3 (0·1–0·6)	0·2 (0·1–0·4)	-30·2 (-79·3–118·5)
Ecuador	0·1 (0–0·2)	0 (0–0·1)	-54 (-83·8–49·2)	0·1 (0–0·2)	0·1 (0–0·1)	-49·9 (-81·5–54·2)	0·1 (0–0·2)	0 (0–0·1)	-52 (-82·8–49·5)
Peru	0·1 (0–0·3)	0·1 (0–0·1)	-45·8 (-84·7–75·8)	0·1 (0–0·3)	0·1 (0–0·2)	-33·3 (-77·1–109·6)	0·1 (0–0·3)	0·1 (0–0·2)	-38·8 (-80·3–91·9)
Caribbean	0·2 (0·1–0·5)	0·2 (0·1–0·5)	-13·8 (-71·6–162·4)	0·3 (0·1–0·8)	0·3 (0·1–0·7)	2·4 (-62·2–206·5)	0·3 (0·1–0·6)	0·3 (0·1–0·6)	-4·6 (-65·8–196)
Antigua and Barbuda	0·2 (0·1–0·4)	0·1 (0–0·2)	-54 (-84·1–54·5)	0·3 (0·1–0·7)	0·2 (0·1–0·4)	-46·1 (-80·7–60·4)	0·2 (0·1–0·5)	0·1 (0–0·3)	-48·9 (-82–60·4)
Bahamas	0·2 (0·1–0·4)	0·1 (0–0·3)	-28·9 (-74·3–145·1)	0·4 (0·1–0·7)	0·3 (0·1–0·6)	-26 (-72·5–137·6)	0·3 (0·1–0·6)	0·2 (0·1–0·4)	-27·2 (-73·4–137)
Barbados	0·2 (0·1–0·5)	0·1 (0–0·3)	-45 (-81·5–75)	0·2 (0·1–0·4)	0·1 (0–0·3)	-25·2 (-73–130·3)	0·2 (0·1–0·5)	0·1 (0–0·3)	-36·3 (-76·7–99·2)
Belize	0·1 (0–0·3)	0·1 (0–0·2)	-32 (-76·5–123)	0·1 (0·1–0·3)	0·1 (0–0·3)	-19·8 (-72–136·6)	0·1 (0–0·3)	0·1 (0–0·2)	-25·8 (-74·5–133·3)
Bermuda	0·1 (0–0·3)	0·1 (0–0·1)	-55 (-84·2–47·8)	0·3 (0·1–0·5)	0·1 (0–0·3)	-45·6 (-80·4–67)	0·2 (0·1–0·4)	0·1 (0–0·2)	-48·8 (-81–64·6)
Cuba	0·1 (0–0·3)	0·1 (0–0·2)	-36 (-77–107·3)	0·2 (0·1–0·4)	0·2 (0·1–0·5)	2 (-62·9–216)	0·2 (0·1–0·4)	0·1 (0–0·3)	-14 (-68·7–175·7)

	Female			Male			Both		
	Age-standardized death rate (per 100,000)		Change (%)	Age-standardized death rate (per 100,000)		Change (%)	Age-standardized death rate (per 100,000)		Change (%)
	1990	2023		1990	2023		1990	2023	
Dominica	0·1 (0–0·3)	0·1 (0–0·2)	-25·5 (-75–155·2)	0·2 (0·1–0·4)	0·1 (0·1–0·3)	-12·5 (-67·4–168·1)	0·1 (0–0·3)	0·1 (0–0·3)	-17·3 (-69–173·4)
Dominican Republic	0·2 (0·1–0·5)	0·2 (0–0·3)	-24·4 (-72·6–136·9)	0·4 (0·2–0·9)	0·5 (0·2–1·1)	9·7 (-55·8–230·7)	0·3 (0·1–0·7)	0·3 (0·1–0·7)	-0·9 (-61–192·2)
Grenada	0·3 (0·1–0·6)	0·1 (0–0·3)	-49·1 (-81·2–65)	0·6 (0·2–1·2)	0·3 (0·1–0·6)	-49·4 (-80·8–51·3)	0·4 (0·1–0·9)	0·2 (0·1–0·5)	-48·3 (-79·3–67·5)
Guyana	0·3 (0·1–0·8)	0·1 (0–0·3)	-54·9 (-84·9–40·8)	0·4 (0·1–1)	0·2 (0·1–0·5)	-51·2 (-82·7–35)	0·4 (0·1–0·9)	0·2 (0·1–0·4)	-52·8 (-82·8–37·9)
Haiti	0·8 (0·3–1·8)	0·9 (0·3–2·1)	9·7 (-62·7–242)	0·9 (0·3–2·1)	0·9 (0·3–2)	6·9 (-63·8–249·8)	0·8 (0·3–1·9)	0·9 (0·3–2·1)	8·4 (-62·7–248)
Jamaica	0·2 (0·1–0·4)	0·1 (0–0·3)	-37·1 (-77·8–105·9)	0·2 (0·1–0·5)	0·2 (0·1–0·5)	-8·1 (-68–175·1)	0·2 (0·1–0·5)	0·2 (0–0·4)	-21·5 (-72·7–145·9)
Puerto Rico	0·1 (0–0·2)	0 (0–0·1)	-65·2 (-88–11·1)	0·2 (0·1–0·4)	0·1 (0–0·2)	-62·6 (-86·6–11·6)	0·1 (0–0·3)	0 (0–0·1)	-63·6 (-87·3–11·9)
Saint Kitts and Nevis	0·3 (0·1–0·6)	0·1 (0–0·3)	-52·2 (-83·1–59·3)	0·3 (0·1–0·7)	0·2 (0–0·3)	-48·4 (-81·6–56·1)	0·3 (0·1–0·7)	0·1 (0–0·3)	-50·3 (-81·9–58·9)
Saint Lucia	0·2 (0·1–0·6)	0·1 (0–0·2)	-61·9 (-87·1–34·9)	0·4 (0·1–0·7)	0·2 (0·1–0·4)	-50·4 (-83·3–51·2)	0·3 (0·1–0·7)	0·1 (0–0·3)	-54·8 (-84·6–47·7)
Saint Vincent and the Grenadines	0·2 (0–0·4)	0·1 (0–0·2)	-46·6 (-80·7–69·3)	0·4 (0·1–0·7)	0·3 (0·1–0·6)	-27·2 (-76·2–127·7)	0·3 (0·1–0·6)	0·2 (0·1–0·4)	-31·9 (-76·1–127·4)
Suriname	0·2 (0·1–0·4)	0·1 (0–0·3)	-23·7 (-74·7–151·8)	0·2 (0·1–0·5)	0·2 (0·1–0·4)	-12·3 (-68·4–151)	0·2 (0·1–0·5)	0·2 (0·1–0·4)	-17·8 (-71·5–151·5)
Trinidad and Tobago	0·3 (0·1–0·7)	0·2 (0–0·3)	-46·5 (-81·2–64)	0·4 (0·1–0·8)	0·2 (0·1–0·5)	-34·9 (-76·2–106·1)	0·3 (0·1–0·8)	0·2 (0·1–0·4)	-40 (-78·8–95)
United States Virgin Islands	0·1 (0–0·3)	0 (0–0·1)	-69·9 (-89·3–-3·8)	0·2 (0·1–0·4)	0·1 (0–0·2)	-40·9 (-78·5–82·5)	0·2 (0·1–0·3)	0·1 (0–0·2)	-53·4 (-82·6–44·4)
Central Latin America	0·2 (0·1–0·5)	0·1 (0–0·2)	-47·8 (-81·9–70·3)	0·3 (0·1–0·6)	0·2 (0·1–0·4)	-30·8 (-75·9–116·8)	0·2 (0·1–0·5)	0·1 (0–0·3)	-38·5 (-78·9–98·1)
Colombia	0·2 (0·1–0·5)	0·1 (0–0·2)	-65·8 (-88–11·8)	0·2 (0·1–0·5)	0·1 (0–0·2)	-55·9 (-84·3–31·8)	0·2 (0·1–0·5)	0·1 (0–0·2)	-60·9 (-86·4–20·8)
Costa Rica	0·1 (0–0·2)	0·1 (0–0·1)	-37·2 (-78·2–103·2)	0·2 (0·1–0·3)	0·1 (0–0·2)	-28·7 (-74·1–106·6)	0·1 (0–0·3)	0·1 (0–0·2)	-31·8 (-76·4–104·3)
El Salvador	0·2 (0·1–0·4)	0·1 (0–0·3)	-37·7 (-78·5–106·5)	0·2 (0·1–0·5)	0·2 (0·1–0·4)	-30 (-73·6–126)	0·2 (0·1–0·5)	0·1 (0–0·3)	-34·1 (-76·9–105·2)

	Female			Male			Both		
	Age-standardized death rate (per 100,000)		Change (%)	Age-standardized death rate (per 100,000)		Change (%)	Age-standardized death rate (per 100,000)		Change (%)
	1990	2023		1990	2023		1990	2023	
Guatemala	0·2 (0·1–0·4)	0·1 (0–0·3)	-32·9 (-77·5–112·4)	0·2 (0·1–0·4)	0·1 (0–0·2)	-32·2 (-75·6–111·5)	0·2 (0·1–0·4)	0·1 (0–0·3)	-32·5 (-76·5–110·7)
Honduras	0·2 (0·1–0·5)	0·2 (0·1–0·4)	-18·9 (-73·2–161·3)	0·3 (0·1–0·7)	0·2 (0·1–0·6)	-12·7 (-70·4–194)	0·2 (0·1–0·6)	0·2 (0·1–0·5)	-15·7 (-70·9–156·7)
Mexico	0·1 (0–0·3)	0·1 (0–0·1)	-58·3 (-85·9–39·7)	0·1 (0–0·3)	0·1 (0–0·1)	-47·3 (-80–60·1)	0·1 (0–0·3)	0·1 (0–0·1)	-53 (-83·4–50·7)
Nicaragua	0·2 (0·1–0·5)	0·1 (0–0·2)	-50·5 (-82·5–62·8)	0·2 (0·1–0·5)	0·1 (0–0·3)	-41·8 (-79·3–75·9)	0·2 (0·1–0·5)	0·1 (0–0·3)	-45·9 (-79·7–77·2)
Panama	0·2 (0·1–0·6)	0·1 (0–0·3)	-42·7 (-79·5–88·7)	0·3 (0·1–0·6)	0·2 (0·1–0·4)	-31·6 (-74·4–106·5)	0·3 (0·1–0·6)	0·2 (0·1–0·4)	-37·1 (-77·1–95·1)
Venezuela (Bolivarian Republic of)	0·6 (0·2–1·3)	0·4 (0·1–1)	-28·3 (-75·9–141·6)	1·1 (0·4–2·5)	1 (0·3–2·2)	-15·7 (-70·6–162·3)	0·9 (0·3–1·9)	0·7 (0·2–1·5)	-21·2 (-72·4–162)
Tropical Latin America	0·6 (0·2–1·5)	0·3 (0·1–0·6)	-59·4 (-85·2–34·3)	1·2 (0·4–2·6)	0·7 (0·2–1·5)	-42 (-78·5–77·9)	0·9 (0·3–2)	0·5 (0·1–1)	-48·6 (-81·4–58·5)
Brazil	0·6 (0·2–1·5)	0·2 (0·1–0·6)	-59·8 (-85·2–33·5)	1·2 (0·4–2·5)	0·7 (0·2–1·4)	-42·8 (-78·7–75·5)	0·9 (0·3–1·9)	0·4 (0·1–0·9)	-49·3 (-81·6–56·7)
Paraguay	0·9 (0·3–2·1)	0·6 (0·2–1·2)	-39·8 (-78·9–96·6)	1·9 (0·7–4·5)	1·7 (0·6–3·6)	-11 (-67·8–158·2)	1·4 (0·5–3·3)	1·1 (0·4–2·3)	-20·6 (-72·4–131·3)
North Africa and Middle East	1·4 (0·5–2·8)	0·5 (0·2–0·9)	-64·7 (-83·3–-24·3)	2·9 (1·2–5·9)	1·2 (0·6–2·2)	-57·8 (-78·1–-7·2)	2·1 (0·9–4·2)	0·9 (0·4–1·5)	-59·6 (-78·8–-19·8)
Afghanistan	1·4 (0·4–3·1)	0·8 (0·2–1·8)	-45·5 (-82·3–67·5)	2·1 (0·7–5)	1·1 (0·3–2·6)	-45·9 (-81·2–85·4)	1·7 (0·6–4·1)	0·9 (0·3–2·1)	-45·1 (-82·2–72·9)
Algeria	1·1 (0·3–2·5)	0·4 (0·1–0·9)	-66·4 (-89·5–1·4)	3·9 (1·3–9)	2 (0·6–4·8)	-49·3 (-82·9–57·5)	2·5 (0·8–5·7)	1·2 (0·4–2·7)	-52·9 (-84·1–42·6)
Bahrain	0·7 (0·2–1·7)	0·1 (0–0·3)	-81·6 (-94·3–-42·5)	1·3 (0·4–3)	0·4 (0·1–0·9)	-70·1 (-89·8–-5·1)	1·1 (0·3–2·4)	0·3 (0·1–0·6)	-72·1 (-91·3–-21·2)
Egypt	0·5 (0·1–1·1)	0·3 (0·1–0·7)	-37·5 (-83–105·6)	2·2 (0·7–5·3)	1·4 (0·4–3·2)	-35·8 (-79·5–119·7)	1·3 (0·4–3)	0·9 (0·3–1·9)	-35·5 (-78·8–106)
Iran (Islamic Republic of)	0·8 (0·3–2)	0·2 (0·1–0·4)	-80·3 (-93·5–-40·4)	1·4 (0·4–3·3)	0·4 (0·1–0·8)	-74·7 (-91·6–-20)	1·1 (0·4–2·7)	0·3 (0·1–0·6)	-76·8 (-92·3–-27)
Iraq	0·4 (0·1–0·8)	0·2 (0·1–0·4)	-46·4 (-83·3–63·7)	0·6 (0·2–1·6)	0·4 (0·1–1·1)	-32·2 (-79·6–147·3)	0·5 (0·2–1·2)	0·3 (0·1–0·7)	-37 (-79·6–106·5)
Jordan	0·3 (0·1–0·9)	0·1 (0–0·3)	-69·7 (-90·2–0·9)	0·6 (0·2–1·5)	0·2 (0·1–0·4)	-71·8 (-91·9–-2·5)	0·5 (0·2–1·1)	0·1 (0–0·3)	-70·9 (-90·8–-1·6)

	Female			Male			Both		
	Age-standardized death rate (per 100,000)		Change (%)	Age-standardized death rate (per 100,000)		Change (%)	Age-standardized death rate (per 100,000)		Change (%)
	1990	2023		1990	2023		1990	2023	
Kuwait	0·2 (0·1–0·6)	0·1 (0–0·2)	-66·9 (-88·8–6·3)	0·4 (0·2–1)	0·2 (0·1–0·4)	-61·7 (-86·2–8·2)	0·4 (0·1–0·8)	0·1 (0–0·3)	-62·1 (-87·2–6·9)
Lebanon	0·2 (0·1–0·5)	0·1 (0–0·2)	-58·2 (-87·4–48·1)	0·5 (0·1–1·1)	0·3 (0·1–0·6)	-44·3 (-84·6–114·9)	0·3 (0·1–0·8)	0·2 (0·1–0·4)	-48·7 (-83·9–77·2)
Libya	0·4 (0·1–1)	0·3 (0·1–0·7)	-30·9 (-80·7–128·6)	1 (0·3–2·4)	0·5 (0·1–1·2)	-49·2 (-84·1–69·7)	0·7 (0·2–1·7)	0·4 (0·1–0·9)	-44·7 (-80·9–85)
Morocco	0·7 (0·2–1·6)	0·4 (0·1–0·9)	-44·3 (-81·4–80·8)	0·6 (0·2–1·6)	0·4 (0·1–0·9)	-43·1 (-81·6–88·9)	0·7 (0·2–1·5)	0·4 (0·1–0·9)	-43·8 (-81–82·6)
Oman	0·5 (0·2–1·2)	0·2 (0·1–0·5)	-52·5 (-85·9–49·1)	1·1 (0·4–2·6)	0·5 (0·2–1·2)	-53·1 (-83·4–62·1)	0·9 (0·2–1·8)	0·4 (0·1–0·8)	-52·7 (-84·2–52·5)
Palestine	0·8 (0·2–1·9)	0·2 (0·1–0·5)	-73·4 (-91·7–-10·7)	1·5 (0·4–3·5)	0·4 (0·1–1)	-71·9 (-90·7–-9·2)	1·1 (0·3–2·7)	0·3 (0·1–0·7)	-72·1 (-90·7–-11·1)
Qatar	0·3 (0·1–0·7)	0·1 (0–0·2)	-78·5 (-93·7–-32)	0·2 (0·1–0·6)	0·1 (0–0·2)	-64·5 (-88·6–17)	0·3 (0·1–0·7)	0·1 (0–0·2)	-68·9 (-90·9–-11·5)
Saudi Arabia	0·7 (0·2–1·6)	0·3 (0·1–0·8)	-53·8 (-84·8–53·6)	0·8 (0·2–1·9)	0·3 (0·1–0·7)	-57·9 (-86·5–37·4)	0·8 (0·2–1·8)	0·3 (0·1–0·8)	-56·3 (-85·4–35·9)
Sudan	1·1 (0·4–2·5)	0·5 (0·1–1·2)	-58·9 (-87·1–30·5)	5·6 (1·6–13·9)	2·9 (0·9–7·1)	-48·9 (-82·5–50·9)	3·3 (1–8·3)	1·7 (0·5–4)	-49·6 (-83·1–47·7)
Syrian Arab Republic	0·2 (0·1–0·5)	0·1 (0–0·4)	-40·3 (-82·2–108·6)	0·3 (0·1–0·7)	0·1 (0–0·3)	-45·9 (-82·1–92·1)	0·3 (0·1–0·6)	0·1 (0–0·4)	-43·6 (-82·3–88·3)
Tunisia	3 (1–6·5)	0·9 (0·3–1·9)	-70·5 (-89·9–-8·4)	5·6 (1·8–13)	1·7 (0·6–4)	-69·7 (-89·6–1·8)	4·3 (1·4–9·1)	1·3 (0·5–2·8)	-70·2 (-88·5–-1·9)
Türkiye	0·6 (0·2–1·5)	0·2 (0·1–0·5)	-71 (-91·4–2·7)	0·7 (0·2–1·7)	0·3 (0·1–0·6)	-63·5 (-88·4–25·3)	0·7 (0·2–1·6)	0·2 (0·1–0·5)	-67·1 (-89·6–2·3)
United Arab Emirates	0·2 (0·1–0·4)	0·1 (0–0·2)	-55·8 (-86·9–77·1)	0·5 (0·1–1·6)	0·3 (0·1–0·6)	-52·9 (-84·9–107·6)	0·4 (0·1–1)	0·2 (0·1–0·4)	-51·3 (-82·6–137·7)
Yemen	24·5 (9·2–49·1)	8·2 (3·7–16·4)	-66·5 (-85·3–-18)	55 (26·8–101)	17·5 (7·5–34·6)	-68·3 (-86·3–-28·7)	39·7 (18·1–71)	12·8 (5·8–23·3)	-67·7 (-85·1–-25·1)
South Asia	18·9 (10·1–33·3)	16·5 (9–26·8)	-12·8 (-56·3–55·7)	24 (12·9–38·3)	24·9 (15·8–37·6)	3·7 (-40·2–85·4)	21·6 (11·8–34·9)	20·7 (12·4–31·5)	-3·8 (-45·1–67·1)
Bangladesh	43·4 (23·6–68·7)	55·1 (30·5–82·2)	26·8 (-29·2–106·9)	25 (12·4–38·6)	30·2 (20·8–41·5)	20·8 (-25·5–113·3)	33·9 (17·6–50·8)	42·9 (26·2–57·8)	26·5 (-24·2–107·7)
Bhutan	18·7 (7·9–36·2)	12·7 (6·2–22·4)	-32·3 (-69·5–47·1)	19·5 (8·2–35·2)	15·1 (8·1–25·4)	-22·6 (-60·4–61·7)	19·1 (9–32·7)	13·9 (7·6–23·5)	-27·2 (-61·7–38·9)

	Female			Male			Both		
	Age-standardized death rate (per 100,000)		Change (%)	Age-standardized death rate (per 100,000)		Change (%)	Age-standardized death rate (per 100,000)		Change (%)
	1990	2023		1990	2023		1990	2023	
India	17·1 (8·8–31·3)	14·1 (7·1–23·9)	-18 (-61–56·3)	26 (14–41·6)	26 (16·3–39·6)	0·2 (-42·3–80·8)	21·7 (11·7–35·5)	20·1 (11·7–30·8)	-7·4 (-49·4–63·2)
Nepal	14·9 (6·6–28·8)	9·6 (4·2–17·7)	-35·5 (-71·2–36·9)	23·4 (11·6–39·4)	23·1 (14·5–35·4)	-1·3 (-45·2–87·6)	19·1 (9·7–32·9)	16·1 (9·7–24·8)	-15·8 (-52·2–55·9)
Pakistan	12·3 (5·4–23·9)	12 (5·2–22·6)	-2·8 (-60–151·3)	10·5 (5–20·1)	11·9 (6·1–22·5)	13·8 (-54·1–153·4)	11·4 (5·3–21)	12 (5·7–21·3)	5·3 (-51·9–147·6)
**Southeast Asia, East Asia, and Oceania**	2·4 (0·9–4·9)	1 (0·4–2)	-59·6 (-83·1–5·3)	2·3 (0·8–5·3)	1·4 (0·6–3)	-37·9 (-76·4–70·2)	2·4 (0·9–5·2)	1·2 (0·5–2·5)	-48·9 (-79·4–36·3)
East Asia	0·6 (0·2–1·5)	0·2 (0·1–0·5)	-64·3 (-88·3–14·2)	1·6 (0·6–3·7)	0·9 (0·3–1·9)	-44·5 (-80·1–68·9)	1·1 (0·4–2·6)	0·6 (0·2–1·2)	-50·3 (-82·3–52·7)
China	0·7 (0·2–1·5)	0·2 (0·1–0·5)	-64·5 (-88·4–13·5)	1·6 (0·6–3·8)	0·9 (0·3–2)	-45·1 (-80·3–67)	1·2 (0·4–2·6)	0·6 (0·2–1·2)	-50·7 (-82·5–51·2)
Democratic People's Republic of Korea	0·3 (0·1–0·6)	0·2 (0·1–0·6)	-11·8 (-70·4–161·1)	0·4 (0·1–1)	0·3 (0·1–0·8)	-18·4 (-74·1–178·1)	0·3 (0·1–0·7)	0·3 (0·1–0·6)	-15·7 (-70·2–174·8)
Taiwan	0·3 (0·1–0·8)	0·1 (0–0·2)	-76·7 (-91·4–-26·8)	0·8 (0·3–1·8)	0·7 (0·3–1·6)	-10·4 (-66·3–178·9)	0·6 (0·2–1·3)	0·4 (0·1–0·9)	-31·6 (-75·1–111·5)
Oceania	1·2 (0·4–2·7)	1·6 (0·6–3·4)	40 (-51·6–301·2)	1·9 (0·7–4·3)	1·7 (0·6–3·9)	-11·4 (-68–180·5)	1·5 (0·6–3·3)	1·6 (0·6–3·5)	7·6 (-58·6–214·7)
American Samoa	0·5 (0·2–1·1)	0·4 (0·1–1)	-16·9 (-75·1–167·4)	1·4 (0·5–3·3)	1·2 (0·4–2·9)	-14·1 (-71·7–201·7)	1 (0·3–2·3)	0·8 (0·3–1·8)	-14·9 (-71·4–169)
Cook Islands	0·5 (0·1–1)	0·3 (0·1–0·6)	-43·5 (-81·6–80·2)	1·2 (0·4–2·9)	1·1 (0·4–2·6)	-4·4 (-69·2–202)	0·8 (0·3–1·9)	0·7 (0·2–1·5)	-18·5 (-72·8–156·7)
Fiji	0·5 (0·2–1·2)	0·6 (0·2–1·3)	11·1 (-63·9–239·9)	1·3 (0·4–3)	1·2 (0·4–2·6)	-9·3 (-66·6–188·3)	0·9 (0·3–2)	0·9 (0·3–1·8)	-3·5 (-65·7–198·9)
Guam	0·2 (0·1–0·4)	0·3 (0·1–0·7)	77·2 (-30·8–456)	1·1 (0·5–2·4)	2·3 (1·2–4·1)	104·4 (-9·6–402·7)	0·7 (0·3–1·5)	1·3 (0·7–2·4)	90·2 (-17·9–387·4)
Kiribati	0·4 (0·1–0·9)	0·6 (0·2–1·3)	30·3 (-60·4–320·9)	3·1 (1·2–6·8)	3·1 (1·2–6·9)	-0·2 (-62·3–178·5)	1·7 (0·6–3·7)	1·8 (0·7–3·7)	3·6 (-60·9–190)
Marshall Islands	0·8 (0·3–2)	1·6 (0·6–3·9)	88 (-38·5–447·6)	2·6 (1–5·7)	2·8 (1·1–6·2)	8·2 (-62·8–255·2)	1·7 (0·6–4·1)	2·2 (0·9–4·7)	27 (-50·9–296·5)
Micronesia (Federated States of)	0·9 (0·3–2·1)	0·7 (0·2–1·7)	-18·5 (-74·7–157·2)	4 (1·5–8·9)	2·4 (0·9–5·6)	-40·9 (-78·5–93)	2·5 (0·8–5·3)	1·6 (0·6–3·4)	-36·9 (-77·5–95·3)
Nauru	0·7 (0·2–1·7)	0·8 (0·2–2)	20·1 (-63·4–313)	1·1 (0·3–3)	1 (0·3–2·4)	-10·5 (-72·4–192·4)	0·9 (0·3–2·3)	0·9 (0·3–2·1)	0·5 (-65–247·8)

	Female			Male			Both		
	Age-standardized death rate (per 100,000)		Change (%)	Age-standardized death rate (per 100,000)		Change (%)	Age-standardized death rate (per 100,000)		Change (%)
	1990	2023		1990	2023		1990	2023	
Niue	0·4 (0·1–0·9)	0·4 (0·1–0·9)	-3·8 (-69·1–224·7)	0·7 (0·2–1·6)	0·6 (0·2–1·3)	-13·6 (-69·9–172·2)	0·6 (0·2–1·3)	0·5 (0·2–1·1)	-10·5 (-68·4–187·3)
Northern Mariana Islands	0·7 (0·2–1·5)	0·7 (0·3–1·5)	7 (-64·8–249·4)	1·9 (0·7–4·3)	2·5 (0·9–5·3)	31·1 (-53·6–284·7)	1·3 (0·5–3·1)	1·6 (0·6–3·5)	23·8 (-56·8–255·5)
Palau	10·9 (4·9–19·9)	12·2 (5·8–21·7)	12 (-50·3–159)	20·1 (8·6–39·4)	15·8 (7·4–30·3)	-21·3 (-67–84·5)	15·7 (7·1–29·6)	14·2 (6·9–24·7)	-9·5 (-59·7–105·4)
Papua New Guinea	1·4 (0·5–3·3)	1·9 (0·7–4)	32·5 (-54·9–319·7)	1·7 (0·6–3·8)	1·4 (0·4–3·5)	-15·3 (-71·7–206·8)	1·5 (0·6–3·3)	1·6 (0·6–3·5)	5·6 (-61–215·8)
Samoa	0·4 (0·1–0·9)	0·4 (0·1–1·1)	11·2 (-67·9–270·9)	0·7 (0·3–1·6)	0·7 (0·2–1·7)	-3·1 (-68–259·7)	0·6 (0·2–1·2)	0·6 (0·2–1·4)	0·7 (-65·8–229·8)
Solomon Islands	1·4 (0·4–3·2)	2 (0·6–4·5)	50·2 (-51·4–392·5)	4·5 (1·5–10·2)	4·8 (1·7–10·6)	5 (-59·2–225·4)	3 (1·1–7·2)	3·4 (1·2–7·3)	14·1 (-58·7–233·2)
Tokelau	0·4 (0·1–0·9)	0·4 (0·1–1)	1·6 (-68·3–244·5)	1·2 (0·4–2·8)	0·9 (0·3–2·1)	-23·4 (-72·9–167·3)	0·8 (0·3–1·9)	0·7 (0·2–1·5)	-15·9 (-69·8–175·7)
Tonga	0·3 (0·1–0·7)	0·4 (0·1–0·9)	17·7 (-65–284·5)	0·6 (0·2–1·4)	0·5 (0·2–1·2)	-16·3 (-73·5–174)	0·5 (0·1–1)	0·4 (0·1–1)	-4·7 (-67·9–196)
Tuvalu	0·8 (0·2–1·7)	0·7 (0·2–1·6)	-12·6 (-75·7–187·3)	1·9 (0·6–4·7)	1·5 (0·5–3·3)	-23·1 (-73–177·7)	1·3 (0·4–3)	1·1 (0·3–2·2)	-16·3 (-72–174·1)
Vanuatu	0·6 (0·2–1·4)	0·8 (0·3–2·1)	31·8 (-58·7–322·7)	2·1 (0·7–5·2)	2·1 (0·7–4·8)	-2·2 (-65·5–218·8)	1·4 (0·5–3·6)	1·5 (0·5–3·3)	4·1 (-65·3–225)
Southeast Asia	8 (3·1–15·7)	3·7 (1·6–7·1)	-53·9 (-79·7–13·6)	4·5 (1·7–10·2)	3·3 (1·4–6·7)	-27·9 (-69·6–90·9)	6·3 (2·5–12·8)	3·5 (1·6–7)	-44·9 (-76–34·7)
Cambodia	30·1 (14·1–52·6)	20·3 (9·7–37·3)	-32·6 (-69·4–49·2)	3·4 (1·3–8·8)	2 (0·7–5·1)	-40·6 (-80·3–101·1)	18 (8·9–33·8)	11·5 (6·9–23·4)	-35·9 (-63·4–45·3)
Indonesia	8·7 (3·2–17·5)	3·5 (1·2–7·6)	-60 (-85·2–7·3)	1·4 (0·5–3·3)	0·9 (0·3–2·1)	-34·1 (-76·3–111·6)	5·1 (2–11)	2·2 (0·8–5·1)	-57·2 (-83·4–15·5)
Lao People's Democratic Republic	15·9 (5·7–34·3)	14·7 (6·1–28·1)	-7·7 (-63·9–139·4)	1·6 (0·5–4·1)	1·2 (0·4–2·9)	-24·4 (-76·4–167·6)	9·1 (3·3–19·6)	7·9 (3·4–16)	-12·7 (-65·7–136·8)
Malaysia	2·3 (0·8–5·4)	1·5 (0·5–3·4)	-37·6 (-79·1–93·8)	2·3 (0·8–5·3)	2·2 (0·8–4·7)	-6·5 (-67·6–208·3)	2·3 (0·8–5·5)	1·8 (0·7–4)	-21·4 (-72·8–143)
Maldives	14·4 (5–31·2)	6 (2–12·6)	-58·1 (-84·8–24·7)	9·8 (3·8–20·9)	5·1 (2·1–10·5)	-48·1 (-79–24·6)	12·1 (4·2–23·7)	5·5 (2·1–10·8)	-54·8 (-80·6–29)

	Female			Male			Both		
	Age-standardized death rate (per 100,000)		Change (%)	Age-standardized death rate (per 100,000)		Change (%)	Age-standardized death rate (per 100,000)		Change (%)
	1990	2023		1990	2023		1990	2023	
Mauritius	4·4 (1·6–8·9)	2·3 (0·8–5·2)	-46 (-79·1–71·8)	2·2 (0·8–4·8)	1·5 (0·5–3·4)	-29·5 (-72·1–108·5)	3·3 (1·4–7·6)	2 (0·7–4·5)	-40·5 (-77·8–75·1)
Myanmar	18·6 (6·9–38·2)	10·9 (3·7–22·4)	-41·3 (-79–73·3)	21 (8·4–46)	20·7 (9–40·2)	-1·7 (-58·6–144·5)	19·8 (7·8–40·6)	15·5 (6·1–29·4)	-21·5 (-67·6–100·5)
Philippines	2·2 (0·8–4·7)	1·4 (0·4–3)	-36·7 (-77·9–83·6)	3·6 (1·3–8·5)	3·8 (1·5–8·1)	5·8 (-58·6–202·1)	2·9 (1–6·5)	2·6 (0·9–4·7)	-9·8 (-68·2–123·1)
Seychelles	3·2 (1·1–6·6)	1·7 (0·6–3·8)	-48·2 (-82·1–55·5)	2·8 (1·1–6·5)	2·1 (0·7–4·6)	-26·3 (-75·5–137·4)	3 (1·2–6·8)	1·9 (0·7–4·4)	-37·2 (-76·9–79·7)
Sri Lanka	6·6 (2·4–13·7)	4·5 (1·5–9·3)	-32 (-75–91·5)	17 (7–32·4)	13·3 (5·9–24·2)	-21·8 (-65·9–82·7)	11·8 (4·7–22·9)	8·6 (3·6–16·3)	-26·4 (-70·3–75·1)
Thailand	7·4 (2·9–14·8)	2·5 (1–5)	-65·5 (-86·9–-11·2)	2·9 (1–6·8)	1·5 (0·6–3·4)	-45·9 (-80·3–67·9)	5·2 (2·1–10·9)	2·1 (0·8–4·3)	-59·9 (-85–2)
Timor-Leste	10·4 (3·8–22·1)	6 (2–13·7)	-42·4 (-80·9–81·7)	2·7 (0·9–7)	1·7 (0·5–3·8)	-36·5 (-79·1–104·7)	6·5 (2·4–14·3)	3·8 (1·3–8)	-40·8 (-80·4–72·6)
Viet Nam	5·1 (1·8–11·5)	2·1 (0·7–4·6)	-59·3 (-84·2–18·4)	1·9 (0·6–4·3)	0·9 (0·3–2)	-52·7 (-82·9–49·8)	3·6 (1·4–9·2)	1·5 (0·6–3·9)	-58·5 (-84·1–21·2)
**Sub-Saharan Africa**	1·6 (0·6–3·3)	1·3 (0·5–3)	-18·9 (-70·5–128·7)	1·6 (0·7–3·4)	1·4 (0·6–3·1)	-13 (-66·6–138·3)	1·6 (0·6–3·6)	1·4 (0·5–2·9)	-16 (-67·6–121·1)
Central Sub-Saharan Africa	1·5 (0·5–3·3)	0·9 (0·3–2·2)	-37·7 (-78·1–103·8)	1 (0·3–2·4)	1 (0·3–2·4)	-0·4 (-67·1–243·3)	1·3 (0·4–2·9)	1 (0·3–2·3)	-23·1 (-71·9–153)
Angola	0·5 (0·2–1·1)	0·4 (0·1–0·9)	-29·9 (-76·7–127·4)	0·6 (0·2–1·5)	0·5 (0·2–1·2)	-16·7 (-72–185·5)	0·6 (0·2–1·3)	0·4 (0·1–1)	-23·6 (-72·1–149·5)
Central African Republic	1·8 (0·6–4·1)	1·8 (0·5–4·2)	0·8 (-68·1–233·8)	0·8 (0·2–2)	0·8 (0·3–1·9)	-10 (-70·5–210)	1·3 (0·5–3·2)	1·3 (0·4–3·1)	-2·9 (-66·2–220·1)
Congo	1·4 (0·5–3)	1·3 (0·4–3)	-7·8 (-68·2–220·3)	0·4 (0·1–1·1)	0·5 (0·2–1·2)	12·5 (-61·5–291·9)	0·9 (0·3–2·2)	0·9 (0·3–2·2)	-3·9 (-64·7–222·7)
Democratic Republic of the Congo	1·9 (0·6–4·1)	1·1 (0·3–2·7)	-42·2 (-79·7–84·6)	1·2 (0·4–2·9)	1·3 (0·4–3·1)	4·8 (-65·6–255·8)	1·6 (0·5–3·4)	1·2 (0·4–2·8)	-24·3 (-72·6–152·5)
Equatorial Guinea	1·5 (0·5–3·2)	0·8 (0·2–1·6)	-48·3 (-83·3–74)	1 (0·3–2·5)	0·7 (0·2–1·8)	-29 (-77·4–148·7)	1·2 (0·4–2·9)	0·7 (0·2–1·7)	-41·6 (-78·6–95·3)
Gabon	0·4 (0·1–1)	0·3 (0·1–0·8)	-25·3 (-75·2–150·8)	0·2 (0·1–0·4)	0·2 (0·1–0·4)	-7·7 (-68·3–220·2)	0·3 (0·1–0·8)	0·3 (0·1–0·6)	-19·5 (-73·5–152·3)

	Female			Male			Both		
	Age-standardized death rate (per 100,000)		Change (%)	Age-standardized death rate (per 100,000)		Change (%)	Age-standardized death rate (per 100,000)		Change (%)
	1990	2023		1990	2023		1990	2023	
Eastern Sub-Saharan Africa	1·6 (0·6–3·3)	1·3 (0·5–2·9)	-17·8 (-68·9–128·2)	2·7 (1·2–5·5)	2·3 (1–4·9)	-14·9 (-65·3–112·4)	2·2 (0·9–4·4)	1·8 (0·7–3·7)	-15·9 (-67·3–104·7)
Burundi	1·7 (0·6–3·8)	1·7 (0·6–3·6)	0·8 (-65·3–248·4)	4·6 (1·7–9·5)	3·4 (1·3–8·2)	-26·3 (-74·3–131·5)	3·1 (1·1–6·1)	2·5 (0·9–5·8)	-16·9 (-69·6–173·5)
Comoros	3·1 (1·2–6·4)	1·6 (0·6–3·6)	-47·8 (-80·3–45·6)	4·5 (1·8–9·6)	2·9 (1·1–5·8)	-36·5 (-75·4–79·3)	3·8 (1·5–7·9)	2·2 (0·9–4·4)	-40·8 (-76·3–50·6)
Djibouti	1 (0·3–2·3)	0·7 (0·2–1·5)	-35 (-77·7–97·5)	1·5 (0·6–3·3)	1 (0·3–2·3)	-30·4 (-73·1–112·3)	1·3 (0·5–3)	0·9 (0·3–1·9)	-31·7 (-75–104)
Eritrea	0·3 (0·1–0·8)	0·3 (0·1–0·8)	-11·8 (-71·5–178·1)	3·9 (1·3–9)	3·2 (1·1–7·1)	-17·9 (-71·2–159·3)	2 (0·6–4·2)	1·8 (0·6–3·5)	-12·7 (-69·6–166·1)
Ethiopia	0·3 (0·1–0·8)	0·4 (0·1–0·9)	16·8 (-65–307·2)	1·5 (0·5–3·4)	2·1 (0·8–4·9)	35·6 (-55·5–356·2)	0·9 (0·3–2·3)	1·2 (0·5–2·9)	32·5 (-56·7–290·5)
Kenya	1·2 (0·4–2·6)	0·9 (0·3–2)	-26·3 (-72·1–120·3)	1·5 (0·6–3·4)	1·2 (0·4–2·7)	-23 (-72·4–124·1)	1·3 (0·5–2·9)	1 (0·3–2·2)	-24·5 (-72·3–134·6)
Madagascar	7·5 (3–14·7)	6·4 (2·7–13·2)	-15·1 (-64·5–115·2)	21·7 (10–41·5)	15 (7·5–28·2)	-31 (-67·3–54)	14·5 (6·6–27·8)	10·6 (5·3–19·3)	-27·3 (-67·2–54·2)
Malawi	2·3 (0·8–5·1)	1·7 (0·6–4)	-27·1 (-72·5–116·5)	1·2 (0·4–3)	1·1 (0·4–2·6)	-11 (-67·7–196·7)	1·8 (0·6–4)	1·4 (0·5–3·4)	-21·6 (-70·3–143·9)
Mozambique	1·3 (0·4–2·9)	1 (0·3–2·2)	-22·6 (-73·6–134·5)	1·5 (0·5–3·6)	1·4 (0·5–3·3)	-5·9 (-68·1–172·9)	1·4 (0·5–3)	1·2 (0·4–2·6)	-14 (-68·9–142)
Rwanda	3·3 (1·1–6·9)	2·2 (0·7–4·6)	-34·6 (-75·6–104·2)	0·8 (0·3–1·9)	0·7 (0·2–1·6)	-17·6 (-72·2–177·1)	2·1 (0·8–4·9)	1·5 (0·5–3·2)	-31·4 (-74·2–108)
Somalia	0·6 (0·2–1·5)	0·6 (0·2–1·4)	6·1 (-65·1–257·2)	0·8 (0·3–1·8)	0·8 (0·3–2)	1 (-65·8–248·5)	0·7 (0·2–1·6)	0·7 (0·2–1·6)	2·9 (-63·8–250)
South Sudan	1·6 (0·5–3·3)	1·5 (0·5–3·5)	-1·2 (-65·5–190·5)	2·4 (0·9–5·7)	2·1 (0·7–4·6)	-15 (-70–170)	2 (0·7–4·8)	1·8 (0·6–4)	-11·1 (-69·5–164·6)
Uganda	2·2 (0·8–4·7)	1·7 (0·6–4·1)	-21·5 (-71·3–153·9)	0·8 (0·3–1·7)	0·7 (0·3–1·8)	-1·3 (-67·1–219·3)	1·5 (0·6–3·1)	1·3 (0·4–3·1)	-16·3 (-69–175·6)
United Republic of Tanzania	0·8 (0·3–1·8)	0·6 (0·2–1·4)	-22·1 (-72·9–140)	0·9 (0·3–2·1)	0·8 (0·3–1·9)	-8·2 (-67·5–177·2)	0·8 (0·3–1·9)	0·7 (0·2–1·6)	-14·8 (-69·4–170·2)
Zambia	1·6 (0·5–4·1)	1·8 (0·6–4·1)	10·2 (-65·4–268·4)	1·1 (0·4–2·4)	1·2 (0·4–2·8)	12 (-60·4–284·8)	1·3 (0·4–3)	1·5 (0·5–3·3)	11·3 (-60·6–248)

	Female			Male			Both		
	Age-standardized death rate (per 100,000)		Change (%)	Age-standardized death rate (per 100,000)		Change (%)	Age-standardized death rate (per 100,000)		Change (%)
	1990	2023		1990	2023		1990	2023	
Southern Sub-Saharan Africa	2·4 (0·9–4·9)	2·1 (0·7–4·7)	-13·3 (-68·4–153·8)	0·8 (0·3–1·7)	0·6 (0·2–1·2)	-24·4 (-72·3–135·1)	1·6 (0·7–3·8)	1·4 (0·5–3·5)	-16 (-68·6–147·2)
Botswana	6·2 (2·5–12·5)	3·6 (1·4–7·4)	-41·8 (-77·3–56·7)	1·9 (0·7–4·3)	1·7 (0·6–3·7)	-9·2 (-68·8–176·2)	4·2 (1·8–9)	2·7 (1·1–5·9)	-35·9 (-73·8–78·8)
Eswatini	1·1 (0·4–2·2)	0·9 (0·3–2)	-13·8 (-72·5–183·9)	2 (0·7–4·6)	1·1 (0·4–2·7)	-42·2 (-79·9–78·9)	1·5 (0·5–3·4)	1 (0·4–2·2)	-30·5 (-75·1–114·1)
Lesotho	2·5 (0·8–5·6)	2·5 (0·8–6·1)	-2·5 (-65·8–204·5)	1·3 (0·4–3)	1·2 (0·4–2·6)	-7·1 (-69·5–203·5)	2 (0·7–4·8)	1·9 (0·7–5)	-7·8 (-65·7–211·4)
Namibia	2·8 (1–6·2)	1·9 (0·6–4·1)	-31·5 (-75·2–95·8)	1·1 (0·4–2·7)	0·7 (0·3–1·7)	-34·6 (-78·4–110·3)	2 (0·8–5·2)	1·4 (0·5–3·1)	-32·6 (-75·6–91·8)
South Africa	2·5 (0·9–5·1)	2·1 (0·7–4·8)	-17·5 (-70·2–136)	0·7 (0·3–1·7)	0·5 (0·2–1·2)	-27·5 (-74·3–130·6)	1·7 (0·7–4)	1·3 (0·5–3·6)	-19·9 (-69·8–139)
Zimbabwe	1·2 (0·4–2·6)	1·8 (0·6–3·8)	45·4 (-48·6–344·6)	0·5 (0·2–1·1)	0·4 (0·1–1·1)	-13·1 (-68·8–193·4)	0·9 (0·3–2·1)	1·2 (0·4–2·7)	32·2 (-54·2–291·8)
Western Sub-Saharan Africa	1·3 (0·4–2·7)	1·1 (0·4–2·5)	-12·9 (-71·6–171·4)	1 (0·4–2·4)	0·9 (0·3–2·2)	-10·4 (-70·1–191·5)	1·1 (0·4–2·8)	1 (0·4–2·2)	-11·5 (-68·3–164)
Benin	1·5 (0·5–3·4)	1·1 (0·4–2·5)	-30·7 (-76·9–114·8)	0·9 (0·3–2·3)	0·6 (0·2–1·5)	-32·6 (-79·2–116·5)	1·3 (0·4–2·7)	0·9 (0·3–2·1)	-32·2 (-73·8–112·1)
Burkina Faso	1 (0·3–2·3)	0·8 (0·3–1·8)	-18·1 (-73·7–162·2)	1·4 (0·5–3·2)	0·9 (0·3–2·2)	-34·2 (-78·4–121·7)	1·2 (0·4–2·7)	0·9 (0·3–1·9)	-26·7 (-74·1–122·2)
Cabo Verde	0·6 (0·2–1·2)	0·3 (0·1–0·6)	-50·8 (-84·2–63·7)	0·4 (0·1–1)	0·3 (0·1–0·7)	-25·7 (-75·5–142·3)	0·5 (0·2–1·1)	0·3 (0·1–0·7)	-41 (-79·1–97·1)
Cameroon	4·7 (1·5–12·3)	4·7 (1·7–10·5)	-0·8 (-67·4–187·1)	1·1 (0·4–2·3)	1 (0·3–2·4)	-5·4 (-71·7–227·7)	3 (1–7·5)	2·9 (1·1–6·7)	-3·6 (-65·7–184·3)
Chad	2·8 (0·9–6)	3·3 (1·1–7·6)	15·7 (-61·5–262·7)	3·3 (1·2–8·2)	3·6 (1·3–8·3)	7·3 (-63·2–232·3)	3·1 (1·1–6·6)	3·4 (1·3–7·6)	11·6 (-57·9–245·2)
Côte d'Ivoire	1·4 (0·5–3·5)	0·9 (0·3–2·1)	-35·5 (-78–105·9)	0·4 (0·1–1)	0·4 (0·1–1)	-5·1 (-68·5–213·8)	0·9 (0·3–2·2)	0·7 (0·2–1·5)	-29·1 (-75·2–139·4)
Gambia	0·9 (0·3–2)	1 (0·3–2·4)	14·9 (-66·3–283·9)	0·5 (0·2–1·3)	0·5 (0·2–1·3)	-5·3 (-69·7–234)	0·7 (0·2–1·7)	0·8 (0·3–1·8)	8·2 (-64·1–251·1)

	Female			Male			Both		
	Age-standardized death rate (per 100,000)		Change (%)	Age-standardized death rate (per 100,000)		Change (%)	Age-standardized death rate (per 100,000)		Change (%)
	1990	2023		1990	2023		1990	2023	
Ghana	1 (0·3–2·3)	0·8 (0·2–1·9)	-21·5 (-73·4–182·9)	2·2 (0·7–5·3)	1·7 (0·5–4·2)	-23·4 (-73·8–153·3)	1·6 (0·5–3·6)	1·2 (0·4–2·8)	-22·5 (-72·6–154)
Guinea	1·5 (0·5–3·2)	1·3 (0·4–3)	-12·7 (-71·4–175·3)	1·2 (0·4–3·2)	1 (0·3–2·4)	-20·1 (-72·9–181·5)	1·4 (0·5–3)	1·1 (0·4–2·5)	-15·8 (-69·6–173·7)
Guinea-Bissau	1·6 (0·5–3·4)	1·7 (0·5–4·2)	9·5 (-64·7–244·9)	0·9 (0·3–2·1)	0·9 (0·3–2)	0·9 (-66·6–233·3)	1·2 (0·4–2·8)	1·3 (0·5–3·1)	6·1 (-60·7–238·7)
Liberia	1·3 (0·4–2·8)	1·2 (0·4–2·9)	-9 (-72·5–180·4)	1·5 (0·5–3·8)	1·2 (0·4–2·8)	-19·5 (-74·5–182·5)	1·4 (0·5–3·3)	1·2 (0·4–2·8)	-14·6 (-72–189)
Mali	1·2 (0·4–2·6)	1·1 (0·3–2·6)	-10·8 (-71·2–179·4)	2·3 (0·9–5·3)	2·1 (0·8–5)	-8·5 (-67·7–163)	1·7 (0·7–4·3)	1·6 (0·6–3·8)	-8·4 (-64·4–167·5)
Mauritania	1·3 (0·4–2·9)	1·1 (0·3–2·7)	-15·7 (-73·2–157·4)	1·1 (0·4–2·6)	0·9 (0·3–2·2)	-17·5 (-74·8–168·8)	1·2 (0·4–2·8)	1 (0·3–2·3)	-16·4 (-72·2–139·4)
Niger	1·5 (0·5–3·6)	1·5 (0·4–4·1)	1 (-70–224·6)	4·2 (1·6–9·9)	3·6 (1·3–7·8)	-13·9 (-70·3–165·9)	2·8 (1·1–6·6)	2·5 (1–5·6)	-9·8 (-68·2–157·4)
Nigeria	0·8 (0·2–1·7)	0·5 (0·2–1·3)	-29·7 (-79–126·1)	0·4 (0·1–0·9)	0·3 (0·1–0·8)	-17·6 (-74·4–207·4)	0·6 (0·2–1·5)	0·4 (0·2–1)	-23·6 (-77·7–108·6)
Sao Tome and Principe	0·5 (0·2–1·1)	0·3 (0·1–0·8)	-36·4 (-80·4–103·6)	0·6 (0·2–1·4)	0·7 (0·2–1·6)	17·9 (-62·1–274·5)	0·5 (0·2–1·2)	0·5 (0·2–1·1)	-6·7 (-70·8–176·2)
Senegal	1·5 (0·5–3·4)	1·5 (0·5–3·4)	-0·7 (-66·5–211)	0·5 (0·2–1·2)	0·4 (0·1–0·9)	-18·1 (-73·6–168·7)	1 (0·3–2·4)	0·9 (0·3–2·2)	-6·1 (-65·6–200·2)
Sierra Leone	2·7 (0·9–6·6)	2·2 (0·7–5)	-17·3 (-72·3–169·2)	1·8 (0·6–4·1)	1·6 (0·5–3·7)	-14·5 (-71·1–191·3)	2·3 (0·8–5·5)	1·9 (0·6–4·3)	-16·6 (-69·1–171·6)
Togo	2·1 (0·7–4·4)	1·7 (0·6–4·1)	-17·6 (-73·1–139·2)	1·4 (0·4–3·4)	1 (0·3–2·4)	-31·1 (-76·6–124·5)	1·8 (0·6–4·1)	1·4 (0·5–3·3)	-22·9 (-72·9–143·1)

Appendix Table 2·8: Number of YLL attributable to chewing tobacco use in 1990 and 2023, among ages 15+

	Female			Male			Both		
	Number of YLLs (thousands)		Change (%)	Number of YLLs (thousands)		Change (%)	Number of YLLs (thousands)		Change (%)
	1990	2023		1990	2023		1990	2023	
Global	1030 (530–1900)	2180 (1160–3620)	112 (9·4–276)	1830 (948–3030)	3980 (2510–6030)	118 (30·4–280)	2860 (1490–4720)	6170 (3760–9370)	116 (21·6–276)
**Central Europe, Eastern Europe, and Central Asia**	9·9 (3·16–23·4)	7·42 (2·29–17·5)	-25·1 (-74·1–142)	20·3 (8–43·8)	22·1 (7·78–47·3)	8·7 (-60·7–218)	30·2 (11·1–67·7)	29·5 (9·87–64·9)	-2·3 (-65–195)
Central Asia	1·3 (0·441–3·09)	1·36 (0·416–3·17)	4·3 (-64·3–230)	7·57 (3·06–15·9)	8·59 (3·19–18)	13·4 (-55·8–200)	8·88 (3·47–18·8)	9·95 (3·59–21·2)	12·1 (-57–204)
Armenia	0·0272 (0·00874–0·0659)	0·0185 (0·00576–0·0431)	-32·1 (-77·6–112)	0·0449 (0·0169–0·0975)	0·0331 (0·0109–0·0755)	-26·3 (-72·4–125)	0·0722 (0·0263–0·165)	0·0516 (0·0163–0·116)	-28·5 (-74·8–121)
Azerbaijan	0·0621 (0·021–0·143)	0·0611 (0·0177–0·147)	-1·6 (-67–223)	0·167 (0·0598–0·378)	0·192 (0·0661–0·435)	14·9 (-58·8–242)	0·229 (0·0831–0·536)	0·253 (0·083–0·581)	10·4 (-60–240)
Georgia	0·119 (0·0382–0·284)	0·0761 (0·0242–0·182)	-36·1 (-77·2–96·9)	0·148 (0·0546–0·33)	0·104 (0·0343–0·232)	-29·6 (-74·1–111)	0·267 (0·0894–0·619)	0·181 (0·0582–0·412)	-32·5 (-76·2–107)
Kazakhstan	0·238 (0·0842–0·565)	0·148 (0·0467–0·33)	-37·7 (-78·5–102)	0·673 (0·257–1·44)	0·506 (0·171–1·14)	-24·8 (-73·2–124)	0·911 (0·341–2·01)	0·654 (0·214–1·47)	-28·2 (-74·8–118)
Kyrgyzstan	0·18 (0·0587–0·423)	0·084 (0·0272–0·196)	-53·3 (-83·6–50·2)	2·37 (0·977–4·78)	1·77 (0·765–3·36)	-25·5 (-70·3–83·6)	2·55 (1·03–5·21)	1·85 (0·787–3·55)	-27·4 (-71·2–81·1)
Mongolia	0·0344 (0·0125–0·0741)	0·029 (0·00888–0·0634)	-15·8 (-71·5–166)	0·0779 (0·0259–0·183)	0·0973 (0·0327–0·211)	25 (-56·9–298)	0·112 (0·0403–0·266)	0·126 (0·0409–0·273)	12·5 (-60·6–271)
Tajikistan	0·185 (0·0654–0·41)	0·372 (0·101–0·85)	101 (-29·4–561)	0·138 (0·0509–0·318)	0·213 (0·0735–0·501)	54·2 (-45·5–357)	0·324 (0·115–0·725)	0·586 (0·176–1·36)	80·9 (-38·5–442)
Turkmenistan	0·0441 (0·0156–0·105)	0·0656 (0·0195–0·149)	48·7 (-52·2–373)	0·0728 (0·0279–0·155)	0·125 (0·0423–0·292)	71·6 (-38·9–424)	0·117 (0·0435–0·259)	0·191 (0·0618–0·44)	63 (-44·2–409)
Uzbekistan	0·414 (0·146–0·971)	0·506 (0·154–1·13)	22·1 (-58·7–278)	3·88 (1·54–8·08)	5·55 (1·92–11·8)	43·2 (-45·3–302)	4·29 (1·68–9·01)	6·05 (2·07–13)	41·1 (-46·9–301)
Central Europe	2·59 (0·824–6·14)	2·42 (0·754–5·6)	-6·5 (-67–212)	5·17 (1·97–11·7)	6·41 (2·21–14)	24·1 (-55·1–280)	7·75 (2·82–17·9)	8·83 (2·94–19·8)	13·9 (-59–261)
Albania	0·0365 (0·0117–0·0823)	0·0444 (0·0125–0·0991)	21·8 (-57·4–286)	0·101 (0·0377–0·223)	0·147 (0·0477–0·329)	45·8 (-47·8–321)	0·138 (0·0488–0·317)	0·192 (0·0602–0·43)	39·4 (-51–309)
Bosnia and Herzegovina	0·0651 (0·0202–0·151)	0·0823 (0·0259–0·187)	26·3 (-56·9–322)	0·124 (0·046–0·285)	0·157 (0·0534–0·351)	27 (-54·8–296)	0·189 (0·0645–0·45)	0·24 (0·0791–0·534)	26·7 (-53·9–313)

	Female			Male			Both		
	Number of YLLs (thousands)		Change (%)	Number of YLLs (thousands)		Change (%)	Number of YLLs (thousands)		Change (%)
	1990	2023		1990	2023		1990	2023	
Bulgaria	0·397 (0·124–0·93)	0·427 (0·135–0·99)	7·7 (-62·1–256)	0·832 (0·29–1·9)	0·937 (0·317–2·05)	12·7 (-59·3–240)	1·23 (0·404–2·83)	1·36 (0·453–3·06)	11·1 (-59·9–247)
Croatia	0·0798 (0·0251–0·185)	0·0438 (0·0138–0·1)	-45·1 (-81·2–80·4)	0·163 (0·0622–0·347)	0·108 (0·0368–0·236)	-33·6 (-76·1–100)	0·243 (0·0911–0·539)	0·152 (0·0509–0·339)	-37·3 (-77·7–96)
Czechia	0·269 (0·0842–0·636)	0·153 (0·0491–0·358)	-43·2 (-79·9–87·1)	0·443 (0·169–0·971)	0·445 (0·154–0·973)	0·5 (-62·4–214)	0·713 (0·25–1·63)	0·598 (0·204–1·33)	-16 (-68·9–168)
Hungary	0·209 (0·0671–0·497)	0·107 (0·0334–0·245)	-48·7 (-81·8–68·9)	0·438 (0·164–0·937)	0·256 (0·0897–0·562)	-41·5 (-79–82·6)	0·648 (0·236–1·47)	0·364 (0·124–0·808)	-43·9 (-80·2–79·5)
Montenegro	0·0111 (0·00372–0·0269)	0·0181 (0·00576–0·0413)	62·5 (-45·7–386)	0·021 (0·00776–0·0468)	0·035 (0·0117–0·0788)	66·7 (-38·4–409)	0·0321 (0·0114–0·0755)	0·0531 (0·0175–0·119)	65·3 (-40·8–417)
North Macedonia	0·0391 (0·013–0·092)	0·0585 (0·0174–0·135)	49·7 (-47·7–383)	0·0661 (0·0224–0·152)	0·106 (0·0356–0·23)	60·9 (-39·3–403)	0·105 (0·0353–0·245)	0·165 (0·0547–0·359)	56·8 (-43·8–393)
Poland	0·663 (0·212–1·56)	0·789 (0·245–1·81)	19 (-56·5–304)	1·23 (0·476–2·68)	2·07 (0·728–4·48)	68·6 (-38·9–415)	1·89 (0·707–4·29)	2·86 (0·978–6·33)	51·2 (-44·8–382)
Romania	0·423 (0·134–0·996)	0·33 (0·101–0·766)	-22·1 (-71·9–155)	0·958 (0·363–2·17)	1·29 (0·449–2·86)	34·7 (-50·8–324)	1·38 (0·494–3·2)	1·62 (0·547–3·64)	17·3 (-57·2–274)
Serbia	0·264 (0·0848–0·652)	0·261 (0·0773–0·601)	-1·1 (-67–223)	0·485 (0·167–1·12)	0·527 (0·181–1·17)	8·6 (-62·9–242)	0·749 (0·263–1·79)	0·788 (0·252–1·73)	5·2 (-63·9–217)
Slovakia	0·0599 (0·0195–0·149)	0·038 (0·0121–0·0861)	-36·6 (-78·5–95·6)	0·16 (0·0617–0·341)	0·135 (0·0495–0·299)	-15·5 (-69·5–163)	0·22 (0·0816–0·49)	0·173 (0·0613–0·387)	-21·2 (-72·3–155)
Slovenia	0·0263 (0·00821–0·0636)	0·0289 (0·00914–0·067)	10 (-62·1–250)	0·0642 (0·0236–0·138)	0·0966 (0·0345–0·213)	50·5 (-43–371)	0·0904 (0·0333–0·207)	0·125 (0·0434–0·278)	38·7 (-48·4–330)
Eastern Europe	6·01 (1·89–14·1)	3·64 (1·14–8·57)	-39·4 (-78·8–95·7)	7·6 (2·94–16·5)	7·12 (2·44–15·5)	-6·4 (-66·2–193)	13·6 (4·84–31·1)	10·8 (3·48–24)	-21 (-71·4–155)
Belarus	0·176 (0·0573–0·414)	0·128 (0·0419–0·292)	-27·7 (-75·5–139)	0·326 (0·124–0·712)	0·436 (0·157–0·963)	33·7 (-51–336)	0·502 (0·181–1·17)	0·563 (0·197–1·25)	12·2 (-59·4–272)
Estonia	0·0312 (0·01–0·0738)	0·0123 (0·0037–0·0279)	-60·8 (-86·2–29·4)	0·0437 (0·0163–0·0951)	0·074 (0·027–0·161)	69·4 (-34·3–414)	0·0749 (0·027–0·17)	0·0863 (0·0307–0·189)	15·2 (-56–263)
Latvia	0·0613 (0·0197–0·147)	0·0377 (0·0116–0·0865)	-38·5 (-78·8–95·2)	0·0683 (0·0255–0·152)	0·0529 (0·0182–0·117)	-22·5 (-72·8–139)	0·13 (0·0455–0·306)	0·0906 (0·0292–0·205)	-30·1 (-75·5–121)
Lithuania	0·0408 (0·0131–0·0978)	0·0323 (0·0101–0·0746)	-20·9 (-72·8–153)	0·0677 (0·0253–0·144)	0·0715 (0·025–0·159)	5·7 (-60·7–238)	0·108 (0·0397–0·246)	0·104 (0·035–0·236)	-4·3 (-65·6–207)

	Female			Male			Both		
	Number of YLLs (thousands)		Change (%)	Number of YLLs (thousands)		Change (%)	Number of YLLs (thousands)		Change (%)
	1990	2023		1990	2023		1990	2023	
Republic of Moldova	0·0652 (0·0203–0·149)	0·0353 (0·0109–0·0826)	-45·8 (-81·2–81·1)	0·148 (0·0572–0·325)	0·157 (0·0516–0·34)	6 (-62·2–222)	0·214 (0·08–0·481)	0·193 (0·0627–0·419)	-9·8 (-68·6–183)
Russian Federation	4·43 (1·4–10·3)	2·89 (0·903–6·83)	-34·8 (-76·9–108)	5·06 (1·98–10·9)	4·88 (1·66–10·7)	-3·6 (-65·1–202)	9·49 (3·38–21·6)	7·77 (2·44–17·4)	-18·1 (-70·1–165)
Ukraine	1·21 (0·38–2·85)	0·507 (0·157–1·15)	-58 (-85·9–39·5)	1·88 (0·7–4·15)	1·44 (0·49–3·19)	-23·4 (-72·7–132)	3·09 (1·07–7·06)	1·95 (0·648–4·33)	-36·9 (-77·6–96·4)
**High-income**	11·8 (3·94–27·7)	12·3 (3·95–28·8)	4·1 (-63–239)	83·3 (50·2–130)	86·4 (54–144)	3·8 (-41·3–87·8)	95·1 (55·6–157)	98·8 (62·3–169)	3·8 (-43·2–101)
Australasia	0·185 (0·0593–0·433)	0·201 (0·0632–0·47)	8·7 (-61·6–252)	0·764 (0·285–1·61)	0·914 (0·331–1·99)	19·6 (-55·2–255)	0·949 (0·352–2·04)	1·11 (0·394–2·46)	17·5 (-56·4–255)
Australia	0·15 (0·0486–0·349)	0·159 (0·0502–0·37)	5·8 (-62·3–238)	0·65 (0·242–1·36)	0·776 (0·283–1·69)	19·4 (-55·2–251)	0·8 (0·297–1·71)	0·935 (0·33–2·06)	16·9 (-56·6–250)
New Zealand	0·0348 (0·0112–0·082)	0·0422 (0·013–0·0963)	21 (-58·7–291)	0·114 (0·0428–0·248)	0·137 (0·0499–0·303)	20·7 (-55–275)	0·149 (0·0556–0·33)	0·18 (0·0628–0·399)	20·7 (-56–281)
High-income Asia Pacific	2·65 (0·847–6·6)	2·7 (0·881–6·2)	1·7 (-63·3–226)	10·5 (3·87–23·8)	11·8 (4·09–25·9)	13·1 (-58·6–236)	13·1 (4·81–30·5)	14·5 (4·91–32·3)	10·8 (-59·6–238)
Brunei Darussalam	0·00784 (0·00276–0·0192)	0·0159 (0·0051–0·0364)	102 (-32·9–557)	0·0138 (0·005–0·0338)	0·0296 (0·0102–0·0675)	115 (-25·7–578)	0·0216 (0·0076–0·0529)	0·0455 (0·0156–0·101)	111 (-28–546)
Japan	1·72 (0·54–4·15)	2·14 (0·692–4·9)	24·8 (-53·7–309)	7·24 (2·72–16·1)	9·34 (3·24–20·5)	29 (-53·1–286)	8·96 (3·32–20·3)	11·5 (3·89–25·6)	28·2 (-53·5–291)
Republic of Korea	0·889 (0·28–2·31)	0·496 (0·163–1·17)	-44·2 (-80–77·7)	3·06 (1·08–7·2)	2·27 (0·762–4·78)	-25·7 (-73·5–121)	3·95 (1·35–9·5)	2·77 (0·915–5·91)	-29·9 (-74·7–115)
Singapore	0·036 (0·0123–0·0828)	0·0397 (0·0126–0·0908)	10 (-60·6–247)	0·161 (0·0618–0·344)	0·204 (0·0745–0·453)	26·1 (-51–282)	0·198 (0·0745–0·427)	0·243 (0·0865–0·544)	23·2 (-52·8–276)
High-income North America	5·39 (1·86–11·8)	6·38 (2·04–14·8)	18·3 (-58·7–269)	64·6 (42·1–98)	65·8 (41·7–101)	1·9 (-36·1–63·4)	70 (44·3–106)	72·2 (45·1–113)	3·1 (-36·5–74·6)
Canada	0·466 (0·16–1·08)	0·662 (0·211–1·51)	42 (-50·5–347)	4·03 (1·63–7·52)	3·35 (1·2–7·08)	-17·1 (-67·9–136)	4·5 (1·8–8·59)	4·01 (1·42–8·48)	-10·9 (-65·9–159)
Greenland	0·00178 (0·000618–0·00402)	0·0014 (0·000457–0·00317)	-21·3 (-73·3–172)	0·021 (0·00811–0·0454)	0·0183 (0·00677–0·0395)	-12·8 (-68·2–166)	0·0228 (0·00875–0·0495)	0·0197 (0·00735–0·0429)	-13·5 (-68·2–162)
United States of America	4·92 (1·7–10·7)	5·71 (1·83–13·2)	16·1 (-59·5–257)	60·5 (40–92)	62·4 (38·7–96)	3·1 (-34·8–62·5)	65·5 (42·3–100)	68·1 (42·7–105)	4·1 (-34·9–72·9)

	Female			Male			Both		
	Number of YLLs (thousands)		Change (%)	Number of YLLs (thousands)		Change (%)	Number of YLLs (thousands)		Change (%)
	1990	2023		1990	2023		1990	2023	
Southern Latin America	0·453 (0·146–1·08)	0·354 (0·11–0·804)	-21·8 (-72·7–156)	0·74 (0·278–1·65)	0·536 (0·187–1·17)	-27·6 (-74·1–126)	1·19 (0·439–2·73)	0·89 (0·293–1·98)	-25·4 (-73·6–140)
Argentina	0·306 (0·0994–0·73)	0·229 (0·0719–0·518)	-25·3 (-73·3–145)	0·458 (0·171–1·03)	0·337 (0·119–0·737)	-26·3 (-73·5–130)	0·764 (0·279–1·75)	0·566 (0·188–1·25)	-25·9 (-73·8–138)
Chile	0·101 (0·0333–0·241)	0·0925 (0·0288–0·212)	-8·8 (-68·1–196)	0·209 (0·0795–0·459)	0·151 (0·0512–0·338)	-27·7 (-73·9–124)	0·31 (0·114–0·701)	0·243 (0·0792–0·545)	-21·6 (-72·1–146)
Uruguay	0·0452 (0·0145–0·106)	0·0327 (0·00967–0·0759)	-27·7 (-75–128)	0·0741 (0·0283–0·154)	0·0478 (0·0167–0·104)	-35·5 (-76·6–105)	0·119 (0·0444–0·267)	0·0805 (0·0267–0·182)	-32·5 (-75·7–113)
Western Europe	3·17 (1·02–7·58)	2·7 (0·856–6·29)	-14·6 (-69·5–187)	6·72 (2·5–14·2)	7·35 (2·6–16·1)	9·3 (-60·7–227)	9·89 (3·64–21·9)	10 (3·47–22·6)	1·6 (-64·1–215)
Andorra	0·000142 (4·56e-05–0·000343)	0·000302 (9·23e-05–0·000707)	113 (-31·5–621)	0·000579 (0·000205–0·00126)	0·00145 (0·000475–0·00301)	151 (-13·9–695)	0·00072 (0·000261–0·00157)	0·00175 (0·000583–0·00377)	143 (-15·6–635)
Austria	0·0681 (0·0216–0·165)	0·0539 (0·0173–0·123)	-20·8 (-71·9–162)	0·142 (0·0526–0·304)	0·205 (0·0733–0·442)	44·7 (-47·7–329)	0·21 (0·0768–0·473)	0·259 (0·09–0·569)	23·5 (-55·6–280)
Belgium	0·0796 (0·0255–0·192)	0·0528 (0·0169–0·122)	-33·6 (-76·4–119)	0·137 (0·0505–0·29)	0·111 (0·0397–0·252)	-18·6 (-71·4–153)	0·216 (0·0782–0·488)	0·164 (0·0566–0·377)	-24·1 (-73·3–143)
Cyprus	0·00577 (0·00194–0·0139)	0·00422 (0·00125–0·00934)	-26·9 (-76·9–130)	0·00966 (0·00296–0·0232)	0·00976 (0·00324–0·0227)	1 (-64·8–230)	0·0154 (0·00504–0·0375)	0·014 (0·00448–0·0317)	-9·4 (-69–182)
Denmark	0·0565 (0·0182–0·135)	0·0676 (0·0212–0·157)	19·7 (-56·7–289)	0·164 (0·0621–0·363)	0·227 (0·0825–0·489)	38·4 (-49·4–296)	0·22 (0·082–0·496)	0·295 (0·104–0·648)	33·6 (-51·1–295)
Finland	0·0392 (0·0124–0·0954)	0·035 (0·011–0·0818)	-10·7 (-67·8–184)	0·0564 (0·0209–0·128)	0·0933 (0·0337–0·203)	65·4 (-39·7–399)	0·0956 (0·034–0·225)	0·128 (0·0443–0·285)	34·2 (-51–311)
France	0·331 (0·108–0·791)	0·3 (0·095–0·702)	-9·2 (-67·6–201)	1·14 (0·419–2·46)	0·776 (0·274–1·73)	-32·2 (-76–108)	1·48 (0·537–3·04)	1·08 (0·367–2·44)	-27 (-74·4–130)
Germany	0·753 (0·24–1·82)	0·726 (0·233–1·67)	-3·6 (-65·4–216)	1·3 (0·486–2·75)	2·1 (0·728–4·62)	60·9 (-41·7–400)	2·06 (0·76–4·61)	2·82 (0·955–6·36)	37·3 (-50·7–336)
Greece	0·125 (0·0392–0·3)	0·0844 (0·0268–0·193)	-32·8 (-76·6–125)	0·165 (0·0598–0·375)	0·137 (0·0464–0·318)	-16·6 (-70·2–154)	0·29 (0·0959–0·677)	0·222 (0·0734–0·512)	-23·6 (-72·8–144)
Iceland	0·0018 (0·00059–0·00423)	0·00287 (0·000916–0·00661)	59·6 (-44·1–451)	0·00672 (0·00252–0·0148)	0·0188 (0·00731–0·0411)	179 (7·1–734)	0·00852 (0·00315–0·0192)	0·0217 (0·0082–0·0482)	154 (-4·9–679)
Ireland	0·0232 (0·00771–0·0543)	0·0194 (0·00629–0·0451)	-16·4 (-69·6–185)	0·0368 (0·0134–0·0798)	0·0415 (0·0151–0·0908)	12·7 (-58·1–240)	0·06 (0·0219–0·135)	0·0609 (0·0209–0·136)	1·4 (-62·9–219)

	Female			Male			Both		
	Number of YLLs (thousands)		Change (%)	Number of YLLs (thousands)		Change (%)	Number of YLLs (thousands)		Change (%)
	1990	2023		1990	2023		1990	2023	
Israel	0·0189 (0·00592–0·0449)	0·0233 (0·00744–0·0548)	23·5 (-57·1–301)	0·0356 (0·0129–0·0804)	0·0558 (0·0195–0·125)	56·7 (-41·9–369)	0·0545 (0·0196–0·125)	0·0791 (0·0265–0·18)	45·2 (-46·9–356)
Italy	0·435 (0·137–1·03)	0·349 (0·111–0·823)	-19·7 (-71·6–167)	0·986 (0·367–2·15)	0·843 (0·303–1·84)	-14·5 (-68·6–152)	1·42 (0·517–3·21)	1·19 (0·408–2·68)	-16·1 (-69·6–161)
Luxembourg	0·00417 (0·00135–0·00989)	0·00223 (0·000701–0·00519)	-46·4 (-81·3–72·8)	0·0065 (0·00238–0·0143)	0·00526 (0·00197–0·0117)	-19 (-69·7–142)	0·0107 (0·00386–0·0245)	0·0075 (0·00269–0·0169)	-29·7 (-74·8–117)
Malta	0·00236 (0·000739–0·00557)	0·00186 (0·000604–0·00441)	-20·9 (-71·7–164)	0·00387 (0·00142–0·00862)	0·00389 (0·00138–0·00872)	0·5 (-62·2–214)	0·00623 (0·00225–0·0141)	0·00576 (0·00195–0·0131)	-7·6 (-65·6–197)
Monaco	0·000617 (0·000201–0·00141)	0·000353 (0·000104–0·000921)	-42·8 (-83–92)	0·00107 (0·000403–0·00253)	0·000999 (0·000347–0·00206)	-6·7 (-71·6–195)	0·00169 (0·000596–0·00401)	0·00135 (0·000455–0·00303)	-19·9 (-74·4–147)
Netherlands	0·0881 (0·0284–0·211)	0·0963 (0·0304–0·224)	9·3 (-61·1–260)	0·134 (0·0501–0·29)	0·187 (0·0673–0·416)	40 (-48·6–316)	0·222 (0·0809–0·495)	0·283 (0·0967–0·643)	27·8 (-53·3–294)
Norway	0·0541 (0·017–0·131)	0·055 (0·0172–0·129)	1·6 (-63·4–240)	0·152 (0·0566–0·34)	0·315 (0·121–0·685)	107 (-21·9–512)	0·207 (0·0752–0·476)	0·37 (0·138–0·815)	79 (-32·4–447)
Portugal	0·153 (0·0477–0·363)	0·0725 (0·0229–0·164)	-52·8 (-83·2–53·4)	0·24 (0·0904–0·541)	0·176 (0·0621–0·39)	-26·6 (-73–132)	0·393 (0·137–0·911)	0·249 (0·0852–0·558)	-36·8 (-77·2–102)
San Marino	0·000145 (4·9e-05–0·000339)	0·000182 (5·45e-05–0·00043)	25·9 (-60·8–325)	0·000338 (0·000116–0·000815)	0·000392 (0·000134–0·000932)	15·7 (-60·4–261)	0·000483 (0·000167–0·00116)	0·000574 (0·000197–0·00134)	18·8 (-59·4–273)
Spain	0·287 (0·0905–0·695)	0·198 (0·0641–0·457)	-30·8 (-75·1–133)	0·624 (0·23–1·27)	0·442 (0·154–0·971)	-29·1 (-75–121)	0·91 (0·335–1·97)	0·64 (0·22–1·44)	-29·6 (-75·5–124)
Sweden	0·0721 (0·023–0·174)	0·133 (0·0428–0·308)	84·4 (-33–496)	0·562 (0·212–1·21)	0·704 (0·266–1·5)	25·2 (-52–262)	0·634 (0·239–1·39)	0·837 (0·308–1·81)	32 (-50·4–285)
Switzerland	0·0461 (0·0147–0·11)	0·0383 (0·0124–0·0897)	-16·8 (-69·9–181)	0·0994 (0·0369–0·21)	0·0999 (0·0362–0·22)	0·5 (-64·5–198)	0·146 (0·0529–0·316)	0·138 (0·0483–0·31)	-5 (-66·1–194)
United Kingdom	0·519 (0·173–1·22)	0·383 (0·122–0·885)	-26·1 (-73·6–147)	0·708 (0·269–1·54)	0·79 (0·283–1·76)	11·6 (-59·2–242)	1·23 (0·459–2·78)	1·17 (0·4–2·67)	-4·3 (-65–200)
**Latin America and Caribbean**	5·63 (1·86–13·2)	8·1 (2·6–18·6)	43·9 (-49·5–368)	9·93 (3·79–21·9)	17·7 (6·29–38·1)	78·5 (-35·9–451)	15·6 (5·71–35·4)	25·8 (8·58–57)	66 (-40·9–423)
Andean Latin America	0·249 (0·0806–0·594)	0·329 (0·103–0·754)	32 (-57·3–330)	0·334 (0·114–0·83)	0·563 (0·168–1·24)	68·2 (-40–442)	0·583 (0·194–1·44)	0·891 (0·269–2·02)	52·7 (-48·3–386)
Bolivia (Plurinational State of)	0·0533 (0·0169–0·12)	0·0792 (0·0266–0·182)	48·7 (-50·5–409)	0·0946 (0·0262–0·238)	0·185 (0·0647–0·443)	95·4 (-43·3–601)	0·148 (0·0448–0·372)	0·264 (0·0877–0·621)	78·6 (-43·4–505)

	Female			Male			Both		
	Number of YLLs (thousands)		Change (%)	Number of YLLs (thousands)		Change (%)	Number of YLLs (thousands)		Change (%)
	1990	2023		1990	2023		1990	2023	
Ecuador	0·0425 (0·0136–0·102)	0·0565 (0·0173–0·129)	32·8 (-52·7–343)	0·053 (0·0185–0·121)	0·0712 (0·0235–0·157)	34·3 (-50·3–320)	0·0955 (0·0314–0·222)	0·128 (0·0408–0·292)	33·6 (-51·4–324)
Peru	0·153 (0·0505–0·369)	0·193 (0·0563–0·428)	26 (-62·9–305)	0·187 (0·0644–0·452)	0·306 (0·088–0·668)	64 (-43·3–416)	0·34 (0·118–0·826)	0·499 (0·143–1·12)	46·9 (-51·5–360)
Caribbean	0·576 (0·195–1·29)	1·06 (0·332–2·45)	83·8 (-39·7–450)	0·857 (0·319–2)	1·78 (0·633–4)	108 (-23·4–525)	1·43 (0·489–3·29)	2·84 (0·984–6·32)	98·3 (-28·5–504)
Antigua and Barbuda	0·000744 (0·000236–0·00174)	0·000714 (0·000211–0·00165)	-4 (-66·1–215)	0·00124 (0·00048–0·0028)	0·00159 (0·000544–0·00356)	28·4 (-55·3–282)	0·00198 (0·000707–0·00458)	0·00231 (0·000744–0·00518)	16·3 (-58·9–259)
Bahamas	0·00229 (0·000769–0·00519)	0·00403 (0·00116–0·0092)	76 (-35·9–493)	0·00608 (0·00232–0·0127)	0·012 (0·00439–0·0259)	97·1 (-27·1–533)	0·00837 (0·00316–0·0181)	0·016 (0·00532–0·0351)	91·3 (-29·4–518)
Barbados	0·00486 (0·00161–0·0114)	0·00474 (0·00133–0·0105)	-2·4 (-67·2–209)	0·00348 (0·00135–0·00745)	0·00522 (0·00172–0·0114)	49·7 (-45·1–359)	0·00834 (0·00299–0·0192)	0·00996 (0·00312–0·0218)	19·4 (-55·3–277)
Belize	0·000878 (0·000278–0·00205)	0·00186 (0·00055–0·00438)	112 (-27·4–609)	0·00128 (0·000464–0·00288)	0·0034 (0·00115–0·00757)	165 (-6·3–689)	0·00216 (0·000734–0·00494)	0·00526 (0·00171–0·012)	143 (-16·4–657)
Bermuda	0·000512 (0·000179–0·00122)	0·000498 (0·000156–0·0012)	-2·9 (-65·7–218)	0·00135 (0·000537–0·00277)	0·00137 (0·00049–0·00287)	1·1 (-64·2–214)	0·00187 (0·000717–0·00392)	0·00187 (0·00065–0·00405)	0 (-64·1–217)
Cuba	0·107 (0·0359–0·244)	0·12 (0·0379–0·273)	12 (-59·6–261)	0·167 (0·0621–0·353)	0·295 (0·11–0·685)	76·7 (-35·3–448)	0·274 (0·1–0·611)	0·415 (0·153–0·989)	51·5 (-44–386)
Dominica	0·00064 (0·000205–0·00152)	0·000597 (0·000185–0·00146)	-6·7 (-68·6–223)	0·000783 (0·000298–0·00178)	0·0011 (0·000418–0·00247)	40·5 (-47·2–335)	0·00142 (5e-04–0·00334)	0·0017 (0·000607–0·00407)	19·2 (-55·8–286)
Dominican Republic	0·0757 (0·0246–0·184)	0·123 (0·0374–0·268)	62·5 (-40·6–418)	0·198 (0·073–0·41)	0·488 (0·168–1·11)	146 (-1·7–637)	0·274 (0·0992–0·595)	0·611 (0·203–1·37)	123 (-11·1–569)
Grenada	0·00184 (0·000599–0·00411)	0·00162 (0·000475–0·00385)	-12·2 (-66·9–185)	0·00328 (0·00127–0·00717)	0·00437 (0·00162–0·00974)	33·4 (-49·9–293)	0·00512 (0·00188–0·0118)	0·00599 (0·00213–0·0134)	17 (-55·4–255)
Guyana	0·0104 (0·0032–0·0239)	0·0081 (0·00244–0·0187)	-22·4 (-74–154)	0·0167 (0·00553–0·0366)	0·0146 (0·00491–0·0343)	-12·4 (-69–152)	0·0271 (0·00887–0·0613)	0·0227 (0·00729–0·0535)	-16·2 (-69·1–145)
Haiti	0·277 (0·0933–0·616)	0·684 (0·216–1·59)	147 (-20·9–703)	0·3 (0·0937–0·733)	0·745 (0·24–1·64)	149 (-15·2–711)	0·576 (0·198–1·28)	1·43 (0·498–3·15)	148 (-14·3–692)
Jamaica	0·0261 (0·00838–0·0627)	0·029 (0·00794–0·0659)	11·3 (-60·8–268)	0·0321 (0·0114–0·0741)	0·0564 (0·0196–0·13)	75·7 (-36·8–430)	0·0582 (0·019–0·136)	0·0854 (0·0276–0·199)	46·8 (-48·2–370)
Puerto Rico	0·0248 (0·0087–0·0579)	0·0184 (0·00599–0·0421)	-25·5 (-74·5–139)	0·0579 (0·0211–0·12)	0·0382 (0·0136–0·0834)	-34·1 (-76·4–97·5)	0·0827 (0·0295–0·177)	0·0566 (0·0198–0·125)	-31·5 (-76·1–110)

	Female			Male			Both		
	Number of YLLs (thousands)		Change (%)	Number of YLLs (thousands)		Change (%)	Number of YLLs (thousands)		Change (%)
	1990	2023		1990	2023		1990	2023	
Saint Kitts and Nevis	0·000763 (0·000242–0·00178)	0·000467 (0·000138–0·00109)	-38·7 (-78·3–105)	0·000746 (0·000274–0·00165)	0·000663 (0·00022–0·00144)	-11·1 (-67·9–173)	0·00151 (0·00049–0·00345)	0·00113 (0·000342–0·00259)	-25·1 (-72·4–138)
Saint Lucia	0·00186 (0·000624–0·00453)	0·00161 (0·000483–0·00367)	-13·1 (-71·2–200)	0·00285 (0·00109–0·00578)	0·00414 (0·00144–0·00952)	45·1 (-53·6–347)	0·00471 (0·00177–0·0106)	0·00575 (0·0019–0·0133)	22·1 (-60·4–292)
Saint Vincent and the Grenadines	0·000922 (0·000298–0·00213)	0·000895 (0·00027–0·00199)	-2·8 (-65·7–203)	0·00223 (0·000865–0·00459)	0·00404 (0·00137–0·00923)	81·4 (-39·9–462)	0·00315 (0·00118–0·00689)	0·00494 (0·00156–0·0111)	56·8 (-47·4–399)
Suriname	0·00375 (0·00119–0·00904)	0·00693 (0·00208–0·0169)	85·2 (-38·7–502)	0·00627 (0·00228–0·0141)	0·0118 (0·00382–0·0267)	88·2 (-32·2–430)	0·01 (0·00358–0·0232)	0·0187 (0·00589–0·0438)	87 (-34·4–456)
Trinidad and Tobago	0·017 (0·00535–0·0401)	0·0182 (0·0055–0·0418)	7·5 (-63–228)	0·0258 (0·00934–0·0571)	0·0355 (0·0124–0·0787)	37·5 (-48·2–339)	0·0428 (0·0147–0·098)	0·0537 (0·0172–0·12)	25·6 (-55·1–308)
United States Virgin Islands	0·000598 (0·000194–0·00135)	0·000524 (0·000161–0·00118)	-12·3 (-69–178)	0·0011 (0·000427–0·00236)	0·00143 (0·000525–0·00317)	30 (-52·2–310)	0·0017 (0·000619–0·00377)	0·00195 (0·000719–0·00431)	15·1 (-56·9–259)
Central Latin America	1·3 (0·42–3·07)	2·06 (0·621–4·99)	58·9 (-45–415)	2·1 (0·8–4·64)	4·11 (1·35–8·83)	95·6 (-31·5–511)	3·4 (1·25–7·76)	6·17 (1·92–13·8)	81·6 (-38·1–483)
Colombia	0·364 (0·119–0·85)	0·383 (0·121–0·878)	5·4 (-63·2–244)	0·423 (0·159–0·934)	0·496 (0·169–1·08)	17·2 (-58·3–249)	0·787 (0·288–1·78)	0·88 (0·289–1·97)	11·8 (-61–247)
Costa Rica	0·0101 (0·00329–0·0238)	0·0212 (0·00704–0·0488)	110 (-27·8–580)	0·0167 (0·00612–0·0369)	0·0384 (0·0134–0·0815)	131 (-15·6–579)	0·0268 (0·00964–0·0612)	0·0597 (0·0205–0·133)	123 (-20·6–574)
El Salvador	0·0536 (0·0178–0·133)	0·0578 (0·0173–0·133)	7·9 (-62·3–260)	0·0759 (0·0267–0·175)	0·0706 (0·0232–0·154)	-7 (-64·4–185)	0·13 (0·0454–0·308)	0·128 (0·0412–0·288)	-0·8 (-64·6–207)
Guatemala	0·0479 (0·0164–0·112)	0·0863 (0·0264–0·198)	80·2 (-39·7–515)	0·0479 (0·0183–0·106)	0·0823 (0·0268–0·189)	71·8 (-36·7–435)	0·0958 (0·035–0·219)	0·169 (0·0533–0·384)	76 (-37·9–473)
Honduras	0·0264 (0·00832–0·0574)	0·0676 (0·0219–0·166)	156 (-15·4–730)	0·0467 (0·0165–0·111)	0·111 (0·0373–0·285)	138 (-27·1–706)	0·0731 (0·0251–0·176)	0·179 (0·0616–0·416)	145 (-18·1–656)
Mexico	0·377 (0·122–0·881)	0·501 (0·156–1·16)	32·8 (-55·1–343)	0·446 (0·167–0·996)	0·756 (0·267–1·6)	69·4 (-35·6–413)	0·823 (0·296–1·92)	1·26 (0·422–2·78)	52·6 (-45·1–389)
Nicaragua	0·0273 (0·00928–0·065)	0·0377 (0·012–0·0924)	38·2 (-51–331)	0·0327 (0·0112–0·0772)	0·0529 (0·0173–0·115)	61·7 (-41·9–407)	0·06 (0·0203–0·141)	0·0906 (0·0296–0·213)	51 (-45·7–388)
Panama	0·0237 (0·00769–0·0555)	0·0428 (0·0132–0·0963)	80·8 (-35·5–495)	0·0305 (0·0111–0·0697)	0·0576 (0·0198–0·13)	89 (-29·1–473)	0·0542 (0·0189–0·126)	0·1 (0·033–0·224)	85·4 (-32·4–483)
Venezuela (Bolivarian Republic of)	0·368 (0·123–0·862)	0·865 (0·237–2·17)	135 (-19·2–674)	0·979 (0·377–2·13)	2·44 (0·784–5·31)	149 (-11·8–670)	1·35 (0·509–3·02)	3·31 (0·985–7·29)	145 (-16·5–694)

Return to Table of Contents

	Female			Male			Both		
	Number of YLLs (thousands)		Change (%)	Number of YLLs (thousands)		Change (%)	Number of YLLs (thousands)		Change (%)
	1990	2023		1990	2023		1990	2023	
Tropical Latin America	3·51 (1·14–8·22)	4·65 (1·49–10·6)	32·6 (-51·6–343)	6·64 (2·53–14·5)	11·3 (4·04–24·3)	69·8 (-37·9–418)	10·1 (3·77–22·8)	15·9 (5·46–34·9)	56·9 (-42·4–394)
Brazil	3·35 (1·09–7·87)	4·42 (1·42–10·1)	31·9 (-51·5–342)	6·32 (2·41–13·7)	10·6 (3·78–22·8)	67·1 (-38·7–410)	9·67 (3·59–21·8)	15 (5·13–32·8)	54·9 (-42·9–388)
Paraguay	0·154 (0·0541–0·357)	0·228 (0·0714–0·501)	48·1 (-48·2–382)	0·316 (0·119–0·712)	0·705 (0·256–1·51)	123 (-19·8–556)	0·469 (0·173–1·08)	0·933 (0·332–2·01)	98·7 (-30·1–501)
North Africa and Middle East	13·5 (5·02–27·8)	13·8 (6·92–27·5)	3 (-52·3–131)	36·3 (15·8–76·2)	41·8 (21·6–80·5)	15·2 (-41·6–156)	49·7 (20·8–101)	55·6 (28·6–104)	11·9 (-41·4–142)
Afghanistan	0·51 (0·162–1·16)	0·537 (0·176–1·27)	5·4 (-67·4–238)	1·26 (0·413–3·02)	1·94 (0·666–4·74)	54·8 (-50–453)	1·76 (0·563–4·21)	2·48 (0·898–5·77)	40·5 (-54·4–389)
Algeria	0·712 (0·233–1·67)	0·935 (0·292–2·27)	31·2 (-58·5–295)	3·61 (1·24–8·31)	5·91 (2·01–13·7)	63·6 (-43·3–412)	4·32 (1·49–10)	6·85 (2·33–15·7)	58·3 (-44·9–382)
Bahrain	0·0053 (0·00181–0·0127)	0·00523 (0·00147–0·0129)	-1·5 (-67·6–209)	0·0153 (0·00533–0·0352)	0·0318 (0·0107–0·0692)	107 (-31·8–512)	0·0206 (0·00728–0·0477)	0·037 (0·0126–0·0824)	79·4 (-39·8–446)
Egypt	0·623 (0·188–1·58)	0·834 (0·261–1·97)	33·7 (-62–333)	2·47 (0·786–5·95)	3·54 (1·15–8·44)	43·6 (-54·9–393)	3·09 (1·01–7·26)	4·37 (1·42–10·1)	41·6 (-53·6–363)
Iran (Islamic Republic of)	1 (0·338–2·33)	0·697 (0·233–1·81)	-30·4 (-76·4–110)	2·37 (0·759–5·79)	1·93 (0·638–4·34)	-18·5 (-74·3–166)	3·37 (1·12–8·12)	2·63 (0·852–6·11)	-22·1 (-74·8–154)
Iraq	0·249 (0·0832–0·608)	0·418 (0·133–0·995)	67·7 (-48–438)	0·456 (0·143–1·13)	1 (0·325–2·72)	120 (-33·9–734)	0·705 (0·23–1·72)	1·42 (0·471–3·67)	102 (-36·7–613)
Jordan	0·0259 (0·00834–0·0625)	0·0459 (0·0139–0·107)	77·5 (-41·9–496)	0·0608 (0·0171–0·152)	0·0972 (0·0317–0·225)	59·9 (-55·3–468)	0·0866 (0·0275–0·216)	0·143 (0·0464–0·342)	65·2 (-48·7–452)
Kuwait	0·00505 (0·00175–0·0115)	0·00867 (0·00266–0·0202)	71·9 (-41–466)	0·0162 (0·00624–0·035)	0·0391 (0·0135–0·0873)	141 (-13·7–593)	0·0213 (0·00797–0·0468)	0·0478 (0·0163–0·107)	125 (-20·4–575)
Lebanon	0·0332 (0·0097–0·0791)	0·043 (0·0133–0·0963)	29·5 (-58·9–361)	0·0784 (0·0223–0·193)	0·116 (0·0379–0·264)	48·3 (-58·1–465)	0·112 (0·0345–0·275)	0·159 (0·0535–0·359)	42·7 (-54·2–409)
Libya	0·0529 (0·0173–0·124)	0·102 (0·0304–0·229)	92 (-44·3–552)	0·146 (0·048–0·355)	0·226 (0·0708–0·516)	55 (-52·7–392)	0·199 (0·0664–0·467)	0·327 (0·111–0·749)	64·9 (-45·7–423)
Morocco	0·622 (0·188–1·47)	0·886 (0·272–2·06)	42·5 (-52·3–353)	0·756 (0·219–1·92)	1·06 (0·313–2·6)	40·2 (-55·5–355)	1·38 (0·43–3·24)	1·95 (0·645–4·42)	41·2 (-51·5–346)
Oman	0·0205 (0·0067–0·0495)	0·0261 (0·00826–0·0621)	27·5 (-62·4–299)	0·0665 (0·0204–0·162)	0·0831 (0·0251–0·198)	24·9 (-57·8–348)	0·087 (0·0276–0·212)	0·109 (0·034–0·247)	25·5 (-57·1–316)

	Female			Male			Both		
	Number of YLLs (thousands)		Change (%)	Number of YLLs (thousands)		Change (%)	Number of YLLs (thousands)		Change (%)
	1990	2023		1990	2023		1990	2023	
Palestine	0·0371 (0·0121–0·0932)	0·0284 (0·00866–0·0674)	-23·5 (-75·5–157)	0·0971 (0·0284–0·236)	0·0742 (0·0253–0·169)	-23·6 (-74·9–169)	0·134 (0·0419–0·335)	0·103 (0·0336–0·234)	-23·6 (-74·7–150)
Qatar	0·0011 (0·000384–0·00251)	0·00202 (0·000619–0·00495)	83·3 (-44·1–448)	0·00619 (0·00185–0·0151)	0·0162 (0·00531–0·0379)	162 (-20·7–802)	0·00729 (0·00226–0·0177)	0·0182 (0·00612–0·0416)	150 (-23·2–735)
Saudi Arabia	0·178 (0·0611–0·416)	0·212 (0·0589–0·517)	18·8 (-59·9–297)	0·357 (0·109–0·862)	0·391 (0·125–0·857)	9·6 (-67·7–272)	0·535 (0·176–1·29)	0·603 (0·189–1·37)	12·7 (-65·1–278)
Sudan	0·553 (0·168–1·28)	0·61 (0·192–1·58)	10·4 (-64·2–270)	4·57 (1·36–11)	5·9 (1·9–14·7)	28·9 (-56·3–280)	5·13 (1·57–12·1)	6·51 (2·05–15·9)	26·9 (-56·6–278)
Syrian Arab Republic	0·0885 (0·0282–0·225)	0·0927 (0·026–0·243)	4·8 (-69·2–277)	0·135 (0·0401–0·336)	0·155 (0·0489–0·341)	14·9 (-62·6–336)	0·223 (0·0724–0·543)	0·248 (0·0774–0·567)	10·9 (-65·9–292)
Tunisia	0·59 (0·193–1·29)	0·579 (0·179–1·25)	-1·8 (-67·1–211)	1·23 (0·407–2·75)	1·33 (0·46–3·05)	7·6 (-63·6–254)	1·82 (0·616–4·25)	1·91 (0·675–4·35)	4·6 (-62·4–209)
Türkiye	1·5 (0·51–3·51)	1·2 (0·342–3)	-20 (-76·2–175)	2·02 (0·665–4·8)	1·76 (0·599–4·03)	-13 (-71·7–191)	3·53 (1·19–8·58)	2·96 (0·942–6·93)	-16 (-72·8–176)
United Arab Emirates	0·00353 (0·00104–0·00912)	0·00692 (0·00216–0·0166)	96·1 (-41·7–743)	0·0183 (0·00465–0·0527)	0·0493 (0·016–0·117)	170 (-16·3–1030)	0·0218 (0·00626–0·0614)	0·0563 (0·0184–0·133)	158 (-18·8–955)
Yemen	6·63 (2·5–13·2)	6·57 (3·21–12·5)	-1 (-52·6–125)	16·5 (8·12–29·3)	16·1 (7·81–28·5)	-2·5 (-55·6–116)	23·1 (11·1–42·1)	22·7 (11·6–40)	-2 (-51·3–114)
South Asia	788 (417–1410)	1860 (1010–3010)	136 (24·2–311)	1400 (746–2190)	3340 (2200–4840)	139 (41·6–312)	2190 (1220–3460)	5200 (3290–7720)	138 (38·4–307)
Bangladesh	143 (76–229)	427 (258–616)	199 (79·3–381)	127 (63·4–197)	391 (281–523)	207 (96–445)	270 (147–417)	818 (571–1090)	203 (100–411)
Bhutan	0·297 (0·134–0·565)	0·619 (0·314–1·03)	108 (-4–381)	0·448 (0·192–0·856)	1·01 (0·587–1·63)	126 (20·2–330)	0·745 (0·341–1·29)	1·63 (0·957–2·55)	119 (16·5–320)
India	595 (308–1100)	1300 (660–2220)	118 (8·9–307)	1180 (635–1860)	2700 (1710–4020)	129 (32·9–303)	1780 (976–2830)	4000 (2420–6090)	125 (28·1–299)
Nepal	7·33 (3·11–14)	14·4 (6·61–26·7)	97·1 (-7·2–320)	21·2 (11–34·3)	57·8 (39–82·2)	173 (60·7–361)	28·5 (14·2–46·3)	72·3 (47–107)	153 (54·2–344)
Pakistan	41·8 (18·3–82·5)	118 (51·1–216)	182 (18·9–625)	69·7 (33·4–132)	194 (101–362)	178 (19·2–479)	112 (54·6–205)	312 (159–563)	180 (21·7–522)
**Southeast Asia, East Asia, and Oceania**	173 (64·4–358)	222 (97·1–451)	28·2 (-47·9–234)	243 (97·1–551)	396 (160–813)	62·8 (-36·5–343)	417 (161–895)	618 (249–1240)	48·4 (-38·9–294)

	Female			Male			Both		
	Number of YLLs (thousands)		Change (%)	Number of YLLs (thousands)		Change (%)	Number of YLLs (thousands)		Change (%)
	1990	2023		1990	2023		1990	2023	
East Asia	43·3 (14·4–98·1)	36·8 (11·3–85·4)	-15 (-71·8–176)	132 (48·6–305)	166 (60·4–362)	26·4 (-54·7–288)	175 (64·9–405)	203 (71·8–444)	16·1 (-59·1–259)
China	42·6 (14·2–96·7)	35·9 (11–82·8)	-15·7 (-72–175)	130 (47·9–301)	163 (59·1–354)	25·3 (-55–284)	172 (64–399)	199 (70·1–434)	15·2 (-59·4–256)
Democratic People's Republic of Korea	0·295 (0·1–0·659)	0·589 (0·18–1·55)	99·8 (-33·1–503)	0·405 (0·138–0·964)	0·756 (0·244–1·75)	86·9 (-40–518)	0·699 (0·23–1·59)	1·34 (0·426–3·06)	92·3 (-33·1–523)
Taiwan	0·366 (0·125–0·864)	0·265 (0·0835–0·603)	-27·7 (-73·4–125)	1·42 (0·538–2·96)	2·9 (1·13–6·36)	104 (-24·5–513)	1·79 (0·668–3·82)	3·17 (1·21–6·99)	77 (-34·2–440)
Oceania	0·571 (0·2–1·43)	2·55 (1·03–5·16)	347 (69·7–1190)	0·933 (0·369–2·15)	2·33 (0·816–5·49)	150 (-9·2–668)	1·5 (0·571–3·2)	4·88 (1·83–10·1)	225 (25·6–768)
American Samoa	0·00159 (0·000548–0·00363)	0·00253 (0·00081–0·0058)	59·1 (-51·5–407)	0·006 (0·00204–0·0141)	0·00792 (0·00283–0·0187)	32·1 (-55·2–355)	0·00759 (0·00254–0·0176)	0·0105 (0·00379–0·024)	37·7 (-52·3–357)
Cook Islands	0·000815 (0·00028–0·00183)	0·000523 (0·000173–0·00119)	-35·9 (-79–109)	0·00246 (0·00074–0·00597)	0·00242 (0·00091–0·00564)	-1·4 (-67·9–218)	0·00327 (0·00106–0·00782)	0·00295 (0·00108–0·00675)	-10 (-69·7–184)
Fiji	0·0293 (0·0104–0·0686)	0·0625 (0·0187–0·138)	113 (-30·6–558)	0·0713 (0·0236–0·164)	0·115 (0·0391–0·256)	62 (-42–400)	0·101 (0·0346–0·229)	0·178 (0·0583–0·382)	76·9 (-35·8–459)
Guam	0·00232 (0·00077–0·00511)	0·00884 (0·00365–0·0182)	281 (49·6–1000)	0·0192 (0·00784–0·0389)	0·0619 (0·0327–0·108)	222 (42·8–648)	0·0216 (0·00877–0·0441)	0·0708 (0·0368–0·125)	228 (41·7–684)
Kiribati	0·00287 (0·000925–0·00605)	0·00915 (0·00296–0·0211)	218 (3·2–816)	0·0262 (0·0103–0·0561)	0·0533 (0·023–0·113)	104 (-18·8–453)	0·0291 (0·0111–0·062)	0·0624 (0·0263–0·13)	115 (-15–474)
Marshall Islands	0·00237 (0·000733–0·00605)	0·0074 (0·00282–0·0173)	212 (5·5–814)	0·0119 (0·0045–0·0256)	0·017 (0·00728–0·0357)	43·2 (-45·5–322)	0·0142 (0·00545–0·0304)	0·0244 (0·0105–0·0503)	71·3 (-32·5–403)
Micronesia (Federated States of)	0·00661 (0·00222–0·015)	0·00874 (0·00271–0·0196)	32·3 (-58·5–305)	0·0353 (0·0136–0·0745)	0·0301 (0·0115–0·071)	-14·6 (-68·9–167)	0·0419 (0·0157–0·0926)	0·0389 (0·0148–0·0876)	-7·2 (-66·6–180)
Nauru	0·000327 (8·64e-05–0·000813)	0·000673 (0·000201–0·00158)	106 (-37·1–622)	0·000617 (0·000161–0·00159)	0·000807 (0·000278–0·00191)	30·7 (-59·7–349)	0·000944 (0·000291–0·00248)	0·00148 (0·000532–0·00333)	56·8 (-47·5–430)
Niue	0·000111 (3·68e-05–0·000251)	0·000101 (3·28e-05–0·000234)	-9·4 (-70·7–207)	0·000184 (5·68e-05–0·000421)	0·000146 (4·77e-05–0·000314)	-20·5 (-72·7–151)	0·000295 (9·4e-05–0·000698)	0·000247 (8·42e-05–0·000559)	-16·4 (-70·9–165)
Northern Mariana Islands	0·00219 (0·00077–0·0051)	0·00472 (0·00174–0·00976)	116 (-25·2–553)	0·00944 (0·00348–0·021)	0·0196 (0·00757–0·041)	107 (-24·2–491)	0·0116 (0·00421–0·0258)	0·0243 (0·00944–0·0505)	109 (-23·1–499)

	Female			Male			Both		
	Number of YLLs (thousands)		Change (%)	Number of YLLs (thousands)		Change (%)	Number of YLLs (thousands)		Change (%)
	1990	2023		1990	2023		1990	2023	
Palau	0·0135 (0·00641–0·0241)	0·0294 (0·0152–0·051)	118 (3–371)	0·0259 (0·012–0·0476)	0·0477 (0·0241–0·0854)	83·9 (-17–315)	0·0394 (0·019–0·072)	0·0771 (0·0406–0·127)	95·4 (-9·1–309)
Papua New Guinea	0·441 (0·152–1·13)	2·15 (0·879–4·38)	386 (77·1–1350)	0·537 (0·19–1·26)	1·47 (0·461–3·51)	174 (-3·4–852)	0·978 (0·365–2·14)	3·62 (1·4–7·63)	270 (41·1–885)
Samoa	0·00388 (0·00117–0·00893)	0·00794 (0·00247–0·0198)	104 (-40·6–568)	0·00824 (0·00297–0·0191)	0·0136 (0·0044–0·0333)	64·6 (-44·8–509)	0·0121 (0·0041–0·0278)	0·0215 (0·00721–0·0522)	77·4 (-39–492)
Solomon Islands	0·0199 (0·00572–0·0483)	0·127 (0·0396–0·274)	535 (113–2020)	0·0889 (0·0306–0·201)	0·314 (0·115–0·705)	253 (37·2–1020)	0·109 (0·0392–0·243)	0·44 (0·154–0·939)	304 (54·7–1150)
Tokelau	5·8e-05 (1·85e-05–0·000129)	7·99e-05 (2·52e-05–0·000188)	37·7 (-56·9–373)	0·000176 (5·88e-05–0·000407)	0·000177 (5·96e-05–0·000392)	0·6 (-64·2–254)	0·000234 (7·76e-05–0·000564)	0·000257 (8·93e-05–0·00059)	9·8 (-60·2–262)
Tonga	0·00172 (0·000551–0·00377)	0·00308 (0·000966–0·00709)	78·9 (-46·6–476)	0·00312 (0·00111–0·00735)	0·00349 (0·00113–0·00831)	11·7 (-64·6–262)	0·00484 (0·00162–0·0109)	0·00657 (0·00226–0·0153)	35·6 (-53·4–322)
Tuvalu	0·000545 (0·000173–0·00126)	0·000638 (0·000204–0·00148)	17·2 (-67–280)	0·00111 (0·000354–0·00262)	0·00122 (0·000374–0·00276)	10·6 (-62·3–298)	0·00165 (0·000539–0·00411)	0·00186 (0·000621–0·00411)	12·7 (-61·6–268)
Vanuatu	0·00545 (0·00176–0·0124)	0·0258 (0·00796–0·0623)	373 (50·1–1360)	0·0275 (0·00911–0·0648)	0·073 (0·0249–0·164)	165 (-5·8–795)	0·033 (0·0107–0·08)	0·0988 (0·0344–0·223)	200 (5·8–877)
Southeast Asia	130 (50·4–258)	183 (81·5–360)	41·2 (-38·9–245)	111 (44·4–242)	227 (99·7–446)	105 (-11·1–411)	240 (93·6–474)	410 (178–777)	70·8 (-27·3–322)
Cambodia	9·47 (4·46–17·3)	18·6 (9·31–34·1)	96·8 (-10·8–342)	1·24 (0·45–3·18)	2·37 (0·916–5·78)	91·1 (-34·9–523)	10·7 (5·19–19·6)	21 (11·7–36·9)	96·1 (11·2–293)
Indonesia	46·8 (16·9–98·3)	47·9 (16·4–110)	2·4 (-63·6–189)	7·05 (2·35–16·6)	16 (5·41–37·6)	126 (-19·3–639)	53·8 (19·1–110)	63·8 (21·9–144)	18·6 (-57–238)
Lao People's Democratic Republic	2·42 (0·87–5·41)	5·92 (2·68–11)	145 (-6·2–525)	0·212 (0·065–0·556)	0·446 (0·148–1·05)	110 (-33·4–648)	2·63 (0·97–5·75)	6·37 (2·81–11·9)	142 (-7·4–526)
Malaysia	1·45 (0·483–3·43)	2·97 (1·08–6·85)	105 (-32·1–524)	1·93 (0·689–4·42)	6·81 (2·52–14·6)	253 (27·5–1040)	3·38 (1·16–8·07)	9·78 (3·54–21·4)	189 (2·2–788)
Maldives	0·0646 (0·0225–0·145)	0·0795 (0·0279–0·165)	23 (-54·9–275)	0·106 (0·0437–0·229)	0·146 (0·0657–0·288)	37·5 (-42·2–231)	0·171 (0·0678–0·375)	0·225 (0·0953–0·43)	32 (-47·9–227)
Mauritius	0·198 (0·0717–0·405)	0·289 (0·0982–0·633)	45·7 (-43·9–366)	0·121 (0·0477–0·261)	0·218 (0·0775–0·482)	80·5 (-29·8–427)	0·319 (0·114–0·669)	0·507 (0·175–1·11)	58·9 (-37·9–387)
Myanmar	32·6 (12·1–67·6)	53·5 (18·9–109)	64·3 (-42·4–349)	67·1 (26·8–139)	129 (59·4–246)	91·7 (-18·9–370)	99·7 (38·9–201)	182 (78·6–345)	82·8 (-22–346)

	Female			Male			Both		
	Number of YLLs (thousands)		Change (%)	Number of YLLs (thousands)		Change (%)	Number of YLLs (thousands)		Change (%)
	1990	2023		1990	2023		1990	2023	
Philippines	3·66 (1·29–8·02)	7·57 (2·46–16·9)	107 (-27·9–502)	7·18 (2·51–16·5)	20·2 (7·86–44)	181 (9·7–726)	10·8 (3·8–24·9)	27·7 (10·4–57·8)	156 (-2·6–631)
Seychelles	0·0126 (0·00433–0·0259)	0·0125 (0·00444–0·0281)	-1 (-66·5–195)	0·0117 (0·00443–0·0269)	0·0231 (0·00833–0·0499)	97·4 (-33·3–517)	0·0243 (0·00907–0·0525)	0·0356 (0·013–0·0774)	46·2 (-48·1–341)
Sri Lanka	4·62 (1·73–9·39)	9·78 (3·47–20)	111 (-20·6–460)	14·8 (6·4–27·9)	31·1 (14·5–55·4)	110 (-3·4–382)	19·4 (8·26–37·2)	40·8 (17·9–74·6)	111 (-8·6–381)
Thailand	14·6 (5·66–30)	20·4 (7·69–40·6)	39·6 (-46·7–266)	6·33 (2·23–15·1)	13·6 (5·11–29·8)	115 (-23·7–555)	21 (8·01–43·1)	34·1 (12·6–70·6)	62·5 (-37·7–350)
Timor-Leste	0·116 (0·0415–0·258)	0·306 (0·0995–0·685)	164 (-15·3–705)	0·0497 (0·0169–0·123)	0·124 (0·0396–0·284)	149 (-15·6–785)	0·166 (0·0598–0·377)	0·43 (0·144–0·919)	159 (-11·1–652)
Viet Nam	13·5 (4·57–30·8)	15·5 (5·24–33·8)	15·2 (-54·3–236)	4·39 (1·5–9·95)	7·39 (2·66–16·4)	68·4 (-38·4–423)	17·9 (6·04–40)	22·9 (7·98–51·7)	28·2 (-49·7–284)
**Sub-Saharan Africa**	27·9 (10·3–57·9)	60 (22·7–130)	115 (-16·4–492)	37·4 (16·2–78)	75·8 (33·7–163)	103 (-20–413)	65·3 (26·7–137)	136 (57·1–282)	108 (-18·8–414)
Central Sub-Saharan Africa	2·66 (0·923–5·91)	4·92 (1·52–12·3)	85·1 (-36·8–480)	2·24 (0·693–5·15)	6·07 (2·15–15·3)	171 (-12·3–792)	4·89 (1·68–10·5)	11 (3·8–24·6)	125 (-19·1–599)
Angola	0·132 (0·0429–0·29)	0·384 (0·124–0·865)	192 (-0·4–823)	0·268 (0·0806–0·653)	0·72 (0·218–1·79)	169 (-9·3–858)	0·399 (0·13–0·95)	1·1 (0·346–2·53)	176 (2·8–813)
Central African Republic	0·163 (0·0539–0·389)	0·392 (0·106–0·873)	141 (-27·8–690)	0·0808 (0·0224–0·195)	0·168 (0·0551–0·378)	107 (-31·1–636)	0·243 (0·0817–0·597)	0·559 (0·177–1·25)	130 (-21·7–641)
Congo	0·113 (0·039–0·246)	0·246 (0·078–0·564)	119 (-26·9–628)	0·0465 (0·0148–0·106)	0·143 (0·0463–0·34)	208 (2·4–968)	0·159 (0·0536–0·343)	0·389 (0·128–0·911)	145 (-9·4–706)
Democratic Republic of the Congo	2·21 (0·749–4·85)	3·83 (1·11–9·63)	73·4 (-41–431)	1·82 (0·567–4·2)	4·97 (1·76–12·5)	174 (-12·5–785)	4·03 (1·38–8·46)	8·81 (2·96–20·2)	119 (-23·4–581)
Equatorial Guinea	0·0198 (0·00661–0·0442)	0·0328 (0·0111–0·0725)	65·6 (-46·5–465)	0·0158 (0·00482–0·0378)	0·0409 (0·0131–0·107)	159 (-20·1–911)	0·0355 (0·0121–0·084)	0·0736 (0·0252–0·174)	107 (-29·2–604)
Gabon	0·0186 (0·00626–0·0418)	0·0299 (0·0094–0·0681)	61·2 (-47·3–432)	0·00958 (0·00316–0·0227)	0·0222 (0·00766–0·0523)	132 (-22·4–744)	0·0282 (0·00939–0·0637)	0·0521 (0·0168–0·121)	85·2 (-34·3–504)
Eastern Sub-Saharan Africa	13·7 (5·64–27·8)	30 (12·8–62)	120 (-11·7–464)	24·9 (11·1–50·4)	49·8 (24–104)	100 (-14·4–362)	38·6 (17·2–77·2)	79·9 (37·6–157)	107 (-14·2–381)
Burundi	0·567 (0·204–1·23)	1·2 (0·421–2·45)	113 (-25·8–602)	1·13 (0·428–2·3)	2·35 (0·916–5·76)	109 (-27·1–545)	1·69 (0·627–3·54)	3·56 (1·31–8·13)	110 (-24·2–537)

	Female			Male			Both		
	Number of YLLs (thousands)		Change (%)	Number of YLLs (thousands)		Change (%)	Number of YLLs (thousands)		Change (%)
	1990	2023		1990	2023		1990	2023	
Comoros	0·0666 (0·0259–0·137)	0·0856 (0·033–0·182)	28·5 (-51·4–230)	0·0922 (0·0377–0·192)	0·146 (0·0595–0·307)	58·7 (-38·1–337)	0·159 (0·0659–0·329)	0·232 (0·092–0·464)	46 (-42·5–272)
Djibouti	0·0167 (0·00545–0·0383)	0·0504 (0·0172–0·107)	202 (3·9–778)	0·0297 (0·012–0·0649)	0·102 (0·0344–0·221)	244 (30·3–913)	0·0464 (0·0179–0·108)	0·153 (0·0516–0·315)	229 (17·1–902)
Eritrea	0·0532 (0·0178–0·121)	0·126 (0·0413–0·308)	137 (-20·4–692)	0·543 (0·186–1·26)	1·18 (0·455–2·63)	117 (-21·5–578)	0·597 (0·21–1·37)	1·31 (0·509–2·92)	119 (-21·4–566)
Ethiopia	0·555 (0·173–1·37)	2·02 (0·719–4·47)	264 (10·2–1190)	3·62 (1·33–7·86)	10·5 (4·16–25·2)	192 (-2·9–871)	4·17 (1·55–9·52)	12·6 (4·9–29·4)	201 (2·9–827)
Kenya	1·05 (0·387–2·18)	2·38 (0·831–5·18)	127 (-22·2–606)	1·22 (0·474–2·74)	2·86 (0·988–6·67)	134 (-20·2–574)	2·27 (0·904–4·88)	5·24 (1·75–11·5)	131 (-20·2–620)
Madagascar	5·63 (2·4–10·6)	13·2 (6·7–25)	135 (9·6–436)	14 (6·6–26·3)	24·2 (13·4–42·9)	72·6 (-13·8–272)	19·7 (9·24–36·4)	37·4 (21–63·6)	90·4 (-8·8–307)
Malawi	0·938 (0·332–2·02)	1·48 (0·543–3·26)	57·5 (-40·5–362)	0·409 (0·142–0·984)	0·727 (0·237–1·67)	77·5 (-40·2–502)	1·35 (0·497–2·92)	2·2 (0·787–5)	63·6 (-39·9–395)
Mozambique	0·672 (0·233–1·49)	0·944 (0·303–2·09)	40·6 (-52·5–338)	0·781 (0·265–1·88)	1·28 (0·432–3·02)	63·4 (-47·6–371)	1·45 (0·489–3·18)	2·22 (0·742–4·9)	52·9 (-47·4–347)
Rwanda	1·32 (0·488–2·78)	1·94 (0·716–3·92)	47·1 (-43·9–344)	0·209 (0·0703–0·485)	0·324 (0·116–0·725)	54·6 (-46–431)	1·52 (0·562–3·25)	2·26 (0·841–4·54)	48·2 (-42–347)
Somalia	0·147 (0·0456–0·378)	0·547 (0·187–1·24)	272 (27·6–1090)	0·221 (0·0766–0·489)	0·627 (0·202–1·57)	184 (-3·4–871)	0·368 (0·129–0·901)	1·17 (0·408–2·73)	219 (8·2–972)
South Sudan	0·338 (0·124–0·712)	0·728 (0·253–1·59)	115 (-24·8–512)	0·738 (0·261–1·78)	1·02 (0·358–2·39)	37·8 (-52·4–351)	1·08 (0·412–2·51)	1·74 (0·607–3·85)	62·1 (-44·3–394)
Uganda	1·14 (0·422–2·42)	2·67 (0·948–6·24)	134 (-15·6–639)	0·566 (0·202–1·2)	1·54 (0·528–3·69)	172 (-10·2–735)	1·71 (0·669–3·41)	4·2 (1·5–9·62)	146 (-12·3–645)
United Republic of Tanzania	0·871 (0·3–1·96)	1·66 (0·539–3·79)	91 (-34·3–498)	1·07 (0·388–2·43)	2·22 (0·777–4·95)	108 (-27·9–526)	1·94 (0·709–4·39)	3·89 (1·32–8·6)	100 (-29·1–538)
Zambia	0·306 (0·088–0·746)	0·952 (0·312–2·21)	211 (-4·5–964)	0·249 (0·0901–0·563)	0·686 (0·225–1·57)	175 (-2·7–898)	0·556 (0·181–1·25)	1·64 (0·573–3·6)	195 (-0·9–819)
Southern Sub-Saharan Africa	4·17 (1·49–8·63)	8·82 (2·91–20·5)	112 (-23·5–517)	1·78 (0·676–4·02)	2·91 (1·03–6·25)	63 (-43·5–396)	5·95 (2·16–12·5)	11·7 (3·92–26·7)	97 (-27·1–465)
Botswana	0·315 (0·131–0·611)	0·554 (0·232–1·11)	76·1 (-31·5–383)	0·109 (0·0392–0·24)	0·277 (0·102–0·626)	155 (-4·8–678)	0·423 (0·174–0·863)	0·831 (0·344–1·66)	96·3 (-21·9–426)

	Female			Male			Both		
	Number of YLLs (thousands)		Change (%)	Number of YLLs (thousands)		Change (%)	Number of YLLs (thousands)		Change (%)
	1990	2023		1990	2023		1990	2023	
Eswatini	0·0276 (0·00956–0·0593)	0·0512 (0·0172–0·111)	85·2 (-41·9–525)	0·0544 (0·0201–0·126)	0·0527 (0·0181–0·126)	-3·1 (-66·5–219)	0·082 (0·0311–0·189)	0·104 (0·0352–0·222)	26·6 (-54·6–304)
Lesotho	0·127 (0·0414–0·279)	0·192 (0·0629–0·472)	51·9 (-47·5–371)	0·0758 (0·0265–0·172)	0·108 (0·0355–0·232)	42·6 (-50·9–361)	0·202 (0·0713–0·438)	0·301 (0·101–0·685)	48·4 (-50·9–361)
Namibia	0·119 (0·0419–0·255)	0·214 (0·0657–0·453)	79·1 (-35·3–429)	0·0784 (0·0261–0·187)	0·122 (0·0429–0·277)	56·1 (-48·6–389)	0·198 (0·0688–0·435)	0·336 (0·115–0·71)	70 (-38·2–383)
South Africa	3·29 (1·17–6·69)	6·75 (2·16–15·5)	105 (-26·4–509)	1·3 (0·493–2·91)	2·08 (0·711–4·43)	59·8 (-45·7–391)	4·59 (1·65–9·59)	8·83 (2·86–20·8)	92·3 (-28·9–475)
Zimbabwe	0·293 (0·098–0·611)	1·06 (0·344–2·31)	262 (27·1–1020)	0·167 (0·061–0·381)	0·271 (0·0962–0·688)	62·4 (-45·7–439)	0·46 (0·155–1·01)	1·33 (0·44–2·85)	190 (-0·2–794)
Western Sub-Saharan Africa	7·44 (2·37–16·2)	16·2 (5·6–36·7)	118 (-29·4–588)	8·46 (3·11–19·3)	16·9 (6·67–39·8)	100 (-32·6–529)	15·9 (5·51–37·3)	33·2 (12·2–71·7)	109 (-22–528)
Benin	0·247 (0·0791–0·533)	0·516 (0·175–1·19)	108 (-27·7–551)	0·2 (0·0703–0·493)	0·441 (0·155–1·04)	121 (-29·2–623)	0·447 (0·145–0·998)	0·957 (0·325–2·1)	114 (-20–534)
Burkina Faso	0·478 (0·166–1·05)	1·03 (0·369–2·18)	116 (-29·9–548)	0·575 (0·203–1·35)	0·872 (0·326–2·05)	51·5 (-51·7–408)	1·05 (0·356–2·34)	1·9 (0·718–4·06)	80·7 (-34·4–441)
Cabo Verde	0·00974 (0·00339–0·0211)	0·00987 (0·00312–0·0213)	1·3 (-66·2–234)	0·00609 (0·00212–0·0145)	0·0122 (0·00411–0·026)	100 (-31·1–546)	0·0158 (0·00526–0·0355)	0·0221 (0·00724–0·0485)	39·5 (-50·7–359)
Cameroon	1·12 (0·363–2·92)	3·26 (1·2–7·28)	190 (-8·6–791)	0·394 (0·135–0·866)	1·01 (0·346–2·46)	156 (-22·6–776)	1·52 (0·497–3·74)	4·27 (1·57–9·72)	181 (-6·5–731)
Chad	0·508 (0·163–1·08)	1·11 (0·358–2·63)	119 (-26·3–652)	0·531 (0·192–1·27)	1·25 (0·467–2·88)	135 (-18·8–641)	1·04 (0·347–2·21)	2·36 (0·83–5·33)	127 (-17·2–620)
Côte d'Ivoire	0·294 (0·0977–0·685)	0·659 (0·22–1·43)	124 (-26·6–596)	0·169 (0·0531–0·385)	0·505 (0·172–1·24)	198 (0–901)	0·464 (0·149–1·09)	1·16 (0·383–2·66)	151 (-10–702)
Gambia	0·0198 (0·00668–0·0437)	0·0752 (0·0232–0·178)	279 (11·3–1180)	0·018 (0·0057–0·0441)	0·0419 (0·0143–0·108)	133 (-28·2–680)	0·0379 (0·0121–0·0907)	0·117 (0·0407–0·281)	209 (4·2–946)
Ghana	0·592 (0·188–1·35)	1·34 (0·42–3·34)	127 (-22·7–717)	1·07 (0·325–2·58)	2·24 (0·711–5·49)	109 (-27·9–591)	1·67 (0·544–3·94)	3·59 (1·14–8·53)	115 (-22·6–607)
Guinea	0·292 (0·0937–0·623)	0·463 (0·151–1·06)	58·4 (-49·6–426)	0·366 (0·116–0·962)	0·528 (0·179–1·32)	44 (-52–415)	0·658 (0·22–1·53)	0·99 (0·338–2·27)	50·4 (-46·9–388)
Guinea-Bissau	0·0407 (0·0129–0·0907)	0·0882 (0·0294–0·209)	117 (-26·5–581)	0·0284 (0·00898–0·0693)	0·0474 (0·015–0·112)	66·8 (-43·9–460)	0·0691 (0·0222–0·165)	0·136 (0·0463–0·319)	96·2 (-30·4–499)

	Female			Male			Both		
	Number of YLLs (thousands)		Change (%)	Number of YLLs (thousands)		Change (%)	Number of YLLs (thousands)		Change (%)
	1990	2023		1990	2023		1990	2023	
Liberia	0·0953 (0·0312–0·212)	0·189 (0·0597–0·48)	98·2 (-39·8–556)	0·183 (0·0576–0·466)	0·318 (0·108–0·749)	74·1 (-46·9–508)	0·278 (0·0881–0·679)	0·507 (0·174–1·19)	82·4 (-39–520)
Mali	0·317 (0·0981–0·704)	0·617 (0·19–1·47)	94·7 (-33·9–541)	1·02 (0·42–2·29)	2·26 (0·928–5·07)	122 (-21·9–536)	1·34 (0·519–3·17)	2·88 (1·12–6·6)	115 (-22·8–515)
Mauritania	0·0944 (0·0313–0·215)	0·177 (0·0561–0·439)	87·5 (-39·8–442)	0·0936 (0·0336–0·221)	0·181 (0·0607–0·458)	93·6 (-41·9–526)	0·188 (0·0643–0·45)	0·358 (0·121–0·835)	90·5 (-38–451)
Niger	0·322 (0·102–0·753)	1·01 (0·309–2·56)	215 (-4·9–883)	1·07 (0·425–2·46)	2·86 (1·09–5·87)	167 (-6·1–691)	1·4 (0·522–3·22)	3·88 (1·52–7·8)	178 (-5·8–696)
Nigeria	2·05 (0·634–4·64)	3·63 (1·21–8·66)	77·4 (-45·8–480)	2·06 (0·708–4·85)	3·2 (1·07–8·48)	55·2 (-51·3–459)	4·1 (1·36–9·58)	6·82 (2·37–16·2)	66·2 (-41·3–449)
Sao Tome and Principe	0·00254 (0·000827–0·00551)	0·00342 (0·000989–0·00844)	34·6 (-58·9–342)	0·00287 (0·000914–0·00669)	0·0071 (0·00237–0·017)	147 (-21·7–692)	0·00541 (0·00173–0·0121)	0·0105 (0·00356–0·0235)	94·4 (-36·1–507)
Senegal	0·279 (0·0918–0·648)	0·806 (0·256–1·85)	189 (0·3–834)	0·129 (0·0422–0·324)	0·247 (0·0864–0·605)	90·9 (-39–539)	0·408 (0·13–0·922)	1·05 (0·348–2·43)	158 (-5·8–751)
Sierra Leone	0·457 (0·163–1·08)	0·629 (0·2–1·36)	37·5 (-53·3–338)	0·372 (0·119–0·846)	0·553 (0·18–1·3)	48·7 (-51·5–379)	0·829 (0·284–1·98)	1·18 (0·397–2·55)	42·5 (-47·5–334)
Togo	0·22 (0·0734–0·467)	0·627 (0·202–1·54)	185 (-5·3–783)	0·164 (0·0517–0·388)	0·358 (0·109–0·89)	118 (-25·4–598)	0·384 (0·126–0·896)	0·985 (0·313–2·31)	157 (-8·4–742)

Return to Table of Contents

Appendix Table 2·9: Age-standardized YLL rate attributable to chewing tobacco use in 1990 and 2023, among ages 15+

	Female			Male			Both		
	Age-standardized YLL rate (per 100,000)		Change (%)	Age-standardized YLL rate (per 100,000)		Change (%)	Age-standardized YLL rate (per 100,000)		Change (%)
	1990	2023		1990	2023		1990	2023	
Global	66·8 (34·4–123)	63·6 (33·8–105)	-4·8 (-51–69·4)	130 (67·2–215)	127 (80·7–192)	-1·9 (-41·1–71·1)	98·2 (50·4–159)	95·3 (57·8–143)	-3 (-44·9–70·6)
**Central Europe, Eastern Europe, and Central Asia**	4·8 (1·5–11·4)	2·6 (0·8–6·1)	-45·9 (-81·3–74)	15·4 (5·9–33·3)	11·4 (4–24·4)	-25·8 (-73·2–119)	9·8 (3·4–20·6)	6·7 (2·2–14·3)	-30·9 (-74·5–114·4)
Central Asia	6·8 (2·3–16·1)	3·8 (1·2–9)	-43·4 (-80·6–79·7)	50·9 (19·8–108)	27·4 (10·1–57·7)	-46·2 (-79·3–45·6)	28 (9·7–54·1)	15·3 (5·2–31)	-45·2 (-78·1–57·9)
Armenia	2·6 (0·8–6·3)	0·9 (0·3–2)	-67·2 (-89·2–2·3)	5·2 (2–11·6)	2·3 (0·7–5·2)	-56·6 (-84–31·5)	3·9 (1·3–8·7)	1·5 (0·5–3·3)	-61·1 (-86·1–21·1)
Azerbaijan	2·9 (1–6·7)	1·5 (0·4–3·6)	-49·6 (-83·1–64)	12·2 (4·4–27·7)	5·6 (1·9–12·8)	-54·6 (-84·1–36·1)	7·3 (2·3–14·8)	3·5 (1·1–7·6)	-52·8 (-81·6–60·1)
Georgia	4·6 (1·5–11)	2·8 (0·9–6·7)	-39 (-78·5–90·9)	8·4 (3–18·8)	6·4 (2·1–14·2)	-23·2 (-71·7–130·5)	6·4 (2·1–14·5)	4·5 (1·4–10·1)	-29·1 (-75–115·8)
Kazakhstan	4·3 (1·5–10·3)	1·8 (0·6–4)	-58·4 (-85·7–34·2)	19·1 (7·4–41·4)	8·5 (2·8–19·1)	-55·5 (-84–33·3)	11·4 (3·7–21·9)	5 (1·4–10·1)	-55·9 (-84·1–38·6)
Kyrgyzstan	14·8 (4·8–34·7)	4·1 (1·3–9·3)	-72·6 (-90·4–-11·5)	269 (107–546)	94·3 (38–184)	-64·9 (-86–-9·9)	137 (46·3–240)	47·9 (17·8–85·7)	-65 (-85–-4·2)
Mongolia	8·3 (3–17·8)	2·8 (0·9–6·2)	-65·9 (-88·5–9·2)	26·3 (8·6–61·6)	12·2 (4·1–26·8)	-53·5 (-83·5–52·3)	17·2 (5·5–37)	7·4 (2·2–14·5)	-57·2 (-85–42·1)
Tajikistan	16·9 (5·9–37·4)	17·2 (4·8–39·6)	2 (-64·5–240·6)	15·5 (5·7–35·5)	10·2 (3·5–23·9)	-34 (-77–98·6)	16·2 (5·7–36·4)	13·7 (4·1–32·4)	-15·4 (-70·5–162)
Turkmenistan	5·5 (1·9–13·1)	3·8 (1·1–8·5)	-31·6 (-78–116·2)	12·1 (4·6–25·8)	8 (2·6–18·3)	-34·2 (-77·1–97·4)	8·7 (3·1–18·4)	5·9 (1·8–13·1)	-32·1 (-76·5–113·8)
Uzbekistan	9·5 (3·3–22·3)	4·8 (1·5–10·9)	-49·2 (-82·9–57·7)	100 (39·2–212)	52·8 (18·2–114)	-47·3 (-80·1–49·4)	53·7 (19·4–106)	28·4 (9·3–58·8)	-47·1 (-79·6–56·4)
Central Europe	4·4 (1·4–10·4)	2·5 (0·8–5·8)	-42·2 (-79·6–94)	11·5 (4·4–26·1)	10 (3·4–21·8)	-13·5 (-68·4–165·8)	7·8 (2·7–17·6)	6·1 (2–13·4)	-21·8 (-71·4–151·5)
Albania	4·5 (1·4–10)	2·7 (0·8–6·1)	-39·2 (-78·8–94·6)	11·7 (4·3–26)	12 (4–26·8)	2·3 (-63·3–196·2)	8·2 (3–19)	7·2 (2·3–15·6)	-11·6 (-69·1–153·8)
Bosnia and Herzegovina	4·6 (1·4–10·6)	3·2 (1–7·3)	-29·3 (-75·3–131·3)	9·5 (3·4–22·2)	8·5 (2·9–19·1)	-9·8 (-68·5–178·1)	7 (2·3–16)	5·8 (1·9–12·8)	-17·2 (-69·2–167·9)
Bulgaria	8·5 (2·7–20)	6·9 (2·2–15·9)	-19·4 (-71·4–165·4)	22·2 (7·6–50·8)	22·4 (7·6–48·9)	1 (-63·5–206)	15·2 (4·9–34·2)	14·4 (4·6–31·3)	-5·5 (-65·6–194·7)

	Female			Male			Both		
	Age-standardized YLL rate (per 100,000)		Change (%)	Age-standardized YLL rate (per 100,000)		Change (%)	Age-standardized YLL rate (per 100,000)		Change (%)
	1990	2023		1990	2023		1990	2023	
Croatia	3 (1–7·1)	1·2 (0·4–2·6)	-62·1 (-87–24)	8·6 (3·3–18·6)	4·4 (1·5–9·6)	-48·4 (-81·2–57·3)	5·7 (2·1–12·2)	2·7 (0·9–5·8)	-52·3 (-82·7–52·3)
Czechia	4·6 (1·4–10·8)	1·7 (0·6–4)	-62 (-86·5–28·2)	11·9 (4·5–26·2)	7·1 (2·5–15·6)	-39·9 (-77·4–87·3)	8·1 (2·7–17·4)	4·4 (1·5–9·5)	-45·7 (-79·1–80·4)
Hungary	3·4 (1·1–8·2)	1·3 (0·4–3)	-61·8 (-86·3–27·6)	10·3 (3·9–22·2)	4·8 (1·7–10·5)	-53·6 (-83·3–45·2)	6·7 (2·4–14·8)	3 (1–6·4)	-55·8 (-84·3–42·2)
Montenegro	5·5 (1·8–13·3)	4·2 (1·4–9·8)	-23·1 (-74·1–135)	11·2 (3·9–25·4)	10·3 (3·5–23)	-7·8 (-65·5–181·2)	8·3 (2·8–19·4)	7·2 (2·4–16·1)	-13·1 (-68·3–175·3)
North Macedonia	6·5 (2·2–15·4)	4·8 (1·4–10·9)	-27·2 (-74·7–135·7)	11·6 (3·9–26·5)	10·4 (3·5–22·7)	-9·6 (-65·9–182·5)	9 (3–20·6)	7·6 (2·5–16·2)	-16·3 (-69·7–164·7)
Poland	3·6 (1·2–8·5)	2·5 (0·8–5·8)	-30·5 (-74·7–136·5)	9·4 (3·6–20·6)	9·9 (3·5–21·4)	5·6 (-61·5–223·5)	6·4 (2·3–14·1)	6 (2·1–13·2)	-5·1 (-64·8–206)
Romania	4·1 (1·3–9·6)	2 (0·6–4·6)	-50·9 (-82·4–60·5)	11 (4·1–25)	12·1 (4·2–26·9)	10·3 (-59·7–246·8)	7·4 (2·6–16·8)	6·9 (2·3–15)	-7·7 (-66·3–193·4)
Serbia	7·3 (2·3–18)	4·2 (1·3–9·6)	-42·3 (-80·6–87·4)	14·8 (4·9–33·8)	10·8 (3·7–24)	-26·9 (-75·3–124·7)	11 (3·7–25·6)	7·5 (2·4–16·3)	-31·8 (-76·8–108)
Slovakia	2·5 (0·8–6·1)	0·9 (0·3–2·2)	-61·8 (-87–20·8)	8·9 (3·4–19·2)	4·6 (1·7–10·2)	-48·2 (-81·4–62)	5·6 (2–11·9)	2·7 (0·9–5·9)	-51·1 (-82·7–60·1)
Slovenia	2·5 (0·8–6·1)	1·3 (0·4–3)	-48·7 (-82·3–66·7)	9·3 (3·4–20·3)	7·1 (2·5–15·5)	-23·7 (-70·7–139·7)	5·7 (2–12·3)	4·2 (1·4–8·9)	-27·1 (-71·7–134·3)
Eastern Europe	4·7 (1·5–11·1)	2·3 (0·7–5·4)	-51·2 (-83–56·4)	10·8 (4·1–23·8)	7 (2·4–15·3)	-35 (-76·4–103·1)	7·5 (2·5–16·3)	4·5 (1·4–9·7)	-40·5 (-77·9–97·1)
Belarus	3 (1–7·1)	1·7 (0·6–3·9)	-44·2 (-81·1–84·8)	9 (3·4–19·8)	9·4 (3·4–20·7)	4·1 (-61·7–238·2)	5·8 (2–12·9)	5·2 (1·8–11·1)	-10·3 (-67–200·5)
Estonia	3·2 (1–7·7)	1·1 (0·3–2·5)	-65·8 (-87·9–16·3)	8 (3–17·5)	10·6 (3·9–23)	32·3 (-48·8–304·9)	5·4 (1·9–11·8)	5·5 (2–12)	2·6 (-59·1–234·7)
Latvia	3·6 (1·1–8·5)	1·7 (0·5–3·9)	-51·8 (-83·4–52·7)	7·3 (2·7–16·3)	5·2 (1·8–11·6)	-28·4 (-74·6–120·7)	5·3 (1·8–12)	3·3 (1–7·3)	-37·1 (-77·5–100·5)
Lithuania	2·1 (0·7–5)	1·1 (0·4–2·6)	-45·6 (-81·2–73·7)	5·3 (2–11·4)	4·6 (1·6–10·3)	-13 (-67·5–178·6)	3·6 (1·3–7·8)	2·7 (0·9–6)	-23·3 (-71·8–148·3)
Republic of Moldova	3·7 (1·1–8·4)	1·3 (0·4–3·1)	-63·7 (-87·5–21·4)	11·9 (4·6–26·1)	8·5 (2·8–18·4)	-28·1 (-74·5–120·1)	7·5 (2·6–15·8)	4·7 (1·4–9·6)	-37 (-78·1–98)
Russian Federation	5·4 (1·7–12·5)	2·6 (0·8–6·2)	-50·8 (-82·7–56·2)	11·7 (4·5–25·9)	6·9 (2·4–15·2)	-40·7 (-78·4–85·2)	8·3 (2·7–17·8)	4·6 (1·4–10)	-44·3 (-78·9–86·6)

	Female			Male			Both		
	Age-standardized YLL rate (per 100,000)		Change (%)	Age-standardized YLL rate (per 100,000)		Change (%)	Age-standardized YLL rate (per 100,000)		Change (%)
	1990	2023		1990	2023		1990	2023	
Ukraine	3·7 (1·2–8·6)	1·5 (0·5–3·5)	-57·9 (-86·1–40·5)	9·9 (3·7–22)	6·9 (2·3–15·3)	-30·2 (-75·3–111·3)	6·5 (2·2–14·3)	4 (1·3–8·7)	-38·7 (-77·8–93·9)
**High-income**	2·3 (0·8–5·4)	1·4 (0·4–3·2)	-41·4 (-79·4–88·9)	24·2 (14·7–38)	12·9 (8–21·8)	-46·7 (-69·5–-5·6)	12·9 (6·6–18·9)	7 (4·2–11·7)	-45·8 (-68·4–14·5)
Australasia	2 (0·6–4·6)	0·9 (0·3–2)	-55·2 (-84·3–45·8)	10·7 (4–22·6)	5 (1·8–10·9)	-53 (-82·2–38·8)	6·3 (2·2–12·5)	2·9 (1–6·2)	-53·4 (-81·7–46·8)
Australia	1·9 (0·6–4·4)	0·8 (0·3–1·9)	-57·8 (-85–37)	10·9 (4·1–23)	5·1 (1·9–11)	-53·6 (-82·4–36·7)	6·3 (2·2–12·6)	2·9 (1–6·2)	-54·4 (-82·1–43·2)
New Zealand	2·2 (0·7–5·2)	1·3 (0·4–3)	-42 (-80·5–88)	9·5 (3·6–20·8)	4·8 (1·8–10·6)	-49·2 (-81·1–58·2)	5·8 (2–11·9)	3 (1–6·5)	-47·4 (-80–73·2)
High-income Asia Pacific	3·4 (1·1–8·4)	1·5 (0·5–3·4)	-55·8 (-84·3–47·5)	17·4 (6·4–39·9)	8·5 (3–18·5)	-51·3 (-82·1–44·5)	10·2 (3·4–21·7)	4·9 (1·6–10·4)	-52·1 (-81·6–53·7)
Brunei Darussalam	21·1 (7·5–52)	10·4 (3·4–23·8)	-50·8 (-83·6–58·5)	32·5 (11·7–79·7)	18·1 (6·2–41·1)	-44·2 (-80·9–82·3)	27·2 (9·5–66·1)	14·5 (4·8–32·3)	-46·7 (-81·8–60·6)
Japan	2·6 (0·8–6·3)	1·6 (0·5–3·7)	-37·7 (-77·3–108·3)	14·5 (5·4–32·4)	9·2 (3·2–20)	-36·9 (-77·1–90·3)	8·4 (2·8–17·3)	5·2 (1·7–11·3)	-37·4 (-75·8–105·5)
Republic of Korea	7·6 (2·4–19·9)	1·3 (0·4–3)	-83·3 (-94–-47·1)	36·6 (12·4–86·6)	7·2 (2·4–15·3)	-80·2 (-92·9–-41)	22 (6·2–45·3)	4·3 (1·3–8·4)	-80·6 (-92·4–-35·4)
Singapore	4·1 (1·4–9·6)	1·2 (0·4–2·8)	-70·4 (-89·5–-6·1)	20·4 (7·8–43·6)	6·3 (2·3–14·1)	-69 (-87·7–-5·1)	12·3 (4·4–25·2)	3·8 (1·3–8·3)	-68·7 (-87·6–-0·5)
High-income North America	3·5 (1·2–7·7)	2·5 (0·8–5·7)	-29·5 (-75·5–120·5)	64·2 (42·4–96·6)	31·9 (20·3–48·7)	-50·4 (-68·7–-22·2)	32·8 (18·3–43·4)	16·9 (10·2–25·5)	-48·4 (-66·4–-3·8)
Canada	3·5 (1·2–8)	2·3 (0·7–5·2)	-34·8 (-77·4–108·1)	41 (16·6–77·1)	13·9 (5–29·4)	-66·1 (-86·9–-4)	21·8 (7·8–37·4)	8 (2·7–16·3)	-63·5 (-85·1–13·6)
Greenland	16·7 (5·8–37·3)	7·1 (2·3–16·3)	-57·5 (-85·4–48·3)	182 (70·4–396)	72·7 (27·2–160)	-60 (-85·6–20·3)	108 (35·1–198)	41·9 (15·4–91·2)	-61·1 (-83·5–36·7)
United States of America	3·5 (1·2–7·6)	2·5 (0·8–5·7)	-28·8 (-75·3–123·2)	66·7 (44·6–100)	34·1 (21·2–52·3)	-48·9 (-67·5–-21·5)	33·9 (19·3–45·2)	18 (10·9–27)	-46·8 (-64·4–-2·5)
Southern Latin America	2·5 (0·8–6)	0·9 (0·3–2·1)	-63·2 (-87·1–20)	5 (1·9–11·2)	1·9 (0·6–4·1)	-63 (-86·8–14·9)	3·7 (1·3–8·4)	1·4 (0·4–3)	-63·1 (-86·9–18·2)
Argentina	2·5 (0·8–6)	0·9 (0·3–2·1)	-62·7 (-86·7–21·4)	4·4 (1·7–10)	1·8 (0·6–3·9)	-59·4 (-85·4–26·3)	3·4 (1·2–7·8)	1·3 (0·4–2·9)	-60·8 (-86·2–25·1)
Chile	2·5 (0·8–6)	0·9 (0·3–1·9)	-66·4 (-88·3–8·9)	6·5 (2·5–14·2)	1·8 (0·6–4)	-72·5 (-90·1–-15)	4·4 (1·6–9·7)	1·3 (0·4–2·9)	-70·6 (-89·4–-6·8)
Uruguay	3 (0·9–6·9)	1·4 (0·4–3·1)	-54 (-84·1–44·5)	6·2 (2·4–13)	3 (1·1–6·5)	-51·8 (-82·5–52·4)	4·5 (1·7–10)	2·1 (0·7–4·7)	-52·9 (-82·9–48·6)

	Female			Male			Both		
	Age-standardized YLL rate (per 100,000)		Change (%)	Age-standardized YLL rate (per 100,000)		Change (%)	Age-standardized YLL rate (per 100,000)		Change (%)
	1990	2023		1990	2023		1990	2023	
Western Europe	1·2 (0·4–3)	0·7 (0·2–1·5)	-45·8 (-80·6–80·9)	4·1 (1·5–8·6)	2·6 (0·9–5·6)	-36·9 (-77·3–88·6)	2·6 (0·9–5·5)	1·6 (0·5–3·5)	-38·7 (-77·8–92·3)
Andorra	0·7 (0·2–1·7)	0·5 (0·1–1·1)	-34·7 (-79·1–112·7)	2·7 (0·9–5·8)	2·5 (0·8–5·2)	-6·7 (-68·2–195·3)	1·8 (0·6–3·8)	1·5 (0·5–3·2)	-13·5 (-70·4–161·4)
Austria	1·2 (0·4–2·9)	0·7 (0·2–1·6)	-43·5 (-80–85·7)	4·5 (1·7–9·8)	3·5 (1·3–7·5)	-22·6 (-71·9–129·9)	2·8 (1–5·9)	2·1 (0·7–4·3)	-25·8 (-73–132·1)
Belgium	1·2 (0·4–2·8)	0·5 (0·2–1·2)	-55·2 (-84·1–49·7)	3·1 (1·2–6·6)	1·5 (0·6–3·5)	-50·5 (-82·6–53·8)	2·1 (0·7–4·6)	1 (0·4–2·3)	-51·6 (-82·6–57·3)
Cyprus	1·8 (0·6–4·4)	0·6 (0·2–1·2)	-69 (-90·2–-1·7)	3·9 (1·2–9·3)	1·6 (0·5–3·8)	-58·2 (-85·4–37·7)	2·8 (0·9–6·6)	1·1 (0·3–2·3)	-62 (-87·2–18·2)
Denmark	1·7 (0·5–3·9)	1·3 (0·4–3·1)	-20·1 (-71·1–161·2)	7 (2·6–15·5)	5·8 (2·1–12·4)	-17·5 (-69·8–136·3)	4·3 (1·4–8·7)	3·5 (1·2–7·5)	-17·5 (-67·4–163·7)
Finland	1·3 (0·4–3)	0·7 (0·2–1·6)	-44·5 (-80·1–81·1)	2·9 (1·1–6·5)	2·9 (1–6·2)	0·7 (-63·2–207·3)	2 (0·7–4·7)	1·8 (0·6–3·9)	-12·5 (-67·3–177·9)
France	0·9 (0·3–2)	0·5 (0·2–1·2)	-42·6 (-79·5–91·1)	4·8 (1·8–10·3)	1·9 (0·7–4·2)	-60·4 (-86–21·3)	2·8 (1–5·6)	1·2 (0·4–2·6)	-57·6 (-84·9–34·5)
Germany	1·3 (0·4–3·1)	0·9 (0·3–2·1)	-29·5 (-74·7–131·4)	3·8 (1·4–8)	3·5 (1·2–7·7)	-6·7 (-66–189·4)	2·4 (0·9–5·2)	2·2 (0·7–4·7)	-11·4 (-67·4–186·2)
Greece	2·1 (0·7–5·1)	0·7 (0·2–1·7)	-65·9 (-88·1–14·7)	3·6 (1·3–8·2)	1·7 (0·6–4)	-51·2 (-82·4–52·4)	2·8 (0·9–6·5)	1·2 (0·4–2·8)	-56·9 (-84·6–37·4)
Iceland	1·6 (0·5–3·7)	1·2 (0·4–2·8)	-22·4 (-72·7–167·6)	7·5 (2·8–16·6)	9·3 (3·6–20·2)	23·8 (-52·8–269·3)	4·5 (1·6–9·6)	5·4 (2–11·4)	18·4 (-54·7–272·2)
Ireland	1·4 (0·5–3·3)	0·6 (0·2–1·4)	-58·6 (-85–41·1)	2·9 (1·1–6·3)	1·5 (0·5–3·3)	-48·1 (-80·6–56·6)	2·1 (0·8–4·7)	1 (0·4–2·3)	-51·7 (-82·1–53·5)
Israel	1 (0·3–2·5)	0·4 (0·1–1)	-57·1 (-85·2–41·3)	2·3 (0·8–5·2)	1·4 (0·5–3·1)	-40·3 (-77·9–79·1)	1·7 (0·6–3·7)	0·9 (0·3–2)	-45·2 (-80–71·6)
Italy	1·2 (0·4–2·7)	0·5 (0·2–1·2)	-54·6 (-84–53·2)	3·8 (1·4–8·3)	1·9 (0·7–4·2)	-49·9 (-81·5–47·7)	2·4 (0·8–5·2)	1·2 (0·4–2·6)	-50·7 (-81·9–55·4)
Luxembourg	1·7 (0·6–4·1)	0·5 (0·2–1·2)	-71·7 (-90·1–-7·3)	4·2 (1·5–9·3)	1·5 (0·6–3·4)	-63·7 (-86·4–8·9)	2·9 (1–6·5)	1 (0·4–2·2)	-65·5 (-87·5–7·7)
Malta	1·4 (0·4–3·4)	0·5 (0·2–1·1)	-66·7 (-88·2–11·4)	3 (1·1–6·6)	1·2 (0·4–2·7)	-59·3 (-84·8–26·7)	2·2 (0·8–4·8)	0·9 (0·3–1·9)	-60·5 (-85·5–26·2)
Monaco	1·9 (0·6–4·5)	0·8 (0·3–2·2)	-56·2 (-87·4–51·3)	5·4 (2–12·8)	3·5 (1·2–7·1)	-36·1 (-80·1–102·5)	3·6 (1·2–8·2)	2·1 (0·7–4·6)	-40·6 (-81·1–90·8)
Netherlands	1 (0·3–2·5)	0·7 (0·2–1·5)	-37·2 (-77·5–105·3)	2·3 (0·9–5)	1·6 (0·6–3·6)	-30·6 (-74·4–105·9)	1·7 (0·6–3·6)	1·1 (0·4–2·5)	-32·5 (-74·7–112·1)

	Female			Male			Both		
	Age-standardized YLL rate (per 100,000)		Change (%)	Age-standardized YLL rate (per 100,000)		Change (%)	Age-standardized YLL rate (per 100,000)		Change (%)
	1990	2023		1990	2023		1990	2023	
Norway	1·9 (0·6–4·5)	1·4 (0·4–3·2)	-27·3 (-73·8–145)	7·6 (2·8–16·9)	9·5 (3·6–20·6)	24·9 (-52·9–269·3)	4·7 (1·7–10·3)	5·5 (2–11·8)	16·1 (-55·1–263·3)
Portugal	2·7 (0·8–6·3)	0·6 (0·2–1·4)	-76·4 (-91·7—24)	5·9 (2·2–13·3)	2·6 (0·9–5·6)	-56·6 (-84·1–37·3)	4·2 (1·4–9·5)	1·5 (0·5–3·4)	-63·4 (-86·6–19·8)
San Marino	1·2 (0·4–2·8)	0·5 (0·2–1·3)	-53·5 (-85–54·4)	3·6 (1·2–8·7)	1·5 (0·5–3·6)	-57·7 (-85·5–32·9)	2·4 (0·8–5·4)	1 (0·3–2·4)	-56·6 (-84·9–41·8)
Spain	1·2 (0·4–3)	0·4 (0·1–1)	-65·1 (-87·6–16·1)	3·9 (1·4–8)	1·4 (0·5–3·1)	-63·4 (-87·2–13·6)	2·5 (0·9–5·3)	0·9 (0·3–2)	-63·7 (-87·2–16·3)
Sweden	1·2 (0·4–2·8)	1·5 (0·5–3·5)	27·8 (-53·6–320·3)	13·9 (5·3–29·9)	11·6 (4·4–24·5)	-16·2 (-67·6–141·8)	7·4 (2·8–15·9)	6·6 (2·4–13·9)	-11 (-66·1–157·8)
Switzerland	1 (0·3–2·3)	0·5 (0·2–1·1)	-51·4 (-82·4–61·8)	3·3 (1·2–7)	1·7 (0·6–3·8)	-47·2 (-81·3–56·9)	2·1 (0·7–4·3)	1·1 (0·4–2·4)	-47·9 (-80·9–64·6)
United Kingdom	1·3 (0·5–3·1)	0·7 (0·2–1·6)	-45·9 (-80·7–82)	2·8 (1–6)	1·9 (0·7–4·3)	-29·7 (-74·1–115·5)	2 (0·7–4·5)	1·3 (0·5–3)	-34·9 (-75·9–105·5)
**Latin America and Caribbean**	6·7 (2·2–15·8)	3·2 (1–7·3)	-52·6 (-83·4–53·8)	12·8 (4·9–28·2)	8·2 (2·9–17·7)	-35·5 (-76·8–99·2)	9·7 (3·5–21·9)	5·6 (1·8–12·2)	-41·9 (-79·6–80·7)
Andean Latin America	2·6 (0·9–6·3)	1·3 (0·4–3·1)	-49·3 (-83·4–64·2)	3·8 (1·3–9·4)	2·5 (0·7–5·5)	-33·9 (-76·3–114)	3·2 (1·1–7·8)	1·9 (0·6–4·3)	-40·3 (-79·8–87·6)
Bolivia (Plurinational State of)	3·5 (1·2–8)	2 (0·7–4·5)	-44·4 (-81·3–86·9)	7·6 (2·2–19·5)	5·8 (2–13·8)	-24·1 (-77·6–166·6)	5·5 (1·6–13·3)	3·8 (1·2–8·6)	-30·3 (-78·7–126·7)
Ecuador	2 (0·6–4·7)	0·8 (0·3–1·9)	-57·8 (-85–40·3)	2·4 (0·9–5·5)	1·2 (0·4–2·6)	-51·2 (-82–52·7)	2·2 (0·7–5·1)	1 (0·3–2·3)	-54·4 (-83·5–43)
Peru	2·7 (0·9–6·5)	1·4 (0·4–3·1)	-48·2 (-84·8–69·1)	3·5 (1·2–8·4)	2·4 (0·7–5·2)	-32·1 (-76·7–112·1)	3·1 (1·1–7·5)	1·9 (0·5–4·2)	-39·1 (-79·9–90·6)
Caribbean	5·7 (1·9–12·7)	5·1 (1·6–11·9)	-9·8 (-70·4–169)	8·9 (3·3–20·8)	9·5 (3·4–21·2)	6·7 (-60·6–220·9)	7·2 (2·5–16·7)	7·2 (2·5–16)	0 (-63·9–205·8)
Antigua and Barbuda	3·5 (1·1–8·3)	1·6 (0·5–3·8)	-53·8 (-83·8–52·2)	7·4 (2·9–16·7)	4 (1·4–9)	-45·6 (-81·1–60·2)	5·4 (1·9–12·4)	2·8 (0·9–6·3)	-47·9 (-81·7–60·1)
Bahamas	3·5 (1·2–8·1)	2·4 (0·7–5·4)	-33·1 (-75·8–126)	10·1 (3·9–21)	7·5 (2·7–16·2)	-26 (-72·7–136·8)	6·7 (2·5–14·6)	4·8 (1·6–10·5)	-28·4 (-73·5–132·6)
Barbados	4 (1·4–9·4)	2·2 (0·6–5)	-44·5 (-81·4–74·3)	4·2 (1·6–8·8)	3·3 (1·1–7·2)	-21 (-71–141·2)	4·1 (1·5–9·5)	2·7 (0·9–6)	-33·2 (-75·2–107·7)
Belize	2·5 (0·8–5·8)	1·6 (0·5–3·8)	-36·2 (-78·1–112·2)	3·5 (1·3–7·9)	2·9 (1–6·4)	-18·8 (-71·5–140·1)	3 (1–7)	2·2 (0·7–5·1)	-26·8 (-74·9–127·5)
Bermuda	2·1 (0·7–5)	0·9 (0·3–2·2)	-56·1 (-84·3–43·5)	6·5 (2·6–13·2)	3·5 (1·3–7·4)	-45·2 (-80·5–70·5)	4·2 (1·6–8·7)	2·2 (0·8–4·7)	-48·1 (-80·9–68·3)

	Female			Male			Both		
	Age-standardized YLL rate (per 100,000)		Change (%)	Age-standardized YLL rate (per 100,000)		Change (%)	Age-standardized YLL rate (per 100,000)		Change (%)
	1990	2023		1990	2023		1990	2023	
Cuba	2·8 (0·9–6·4)	1·6 (0·5–3·6)	-43·7 (-79·8–82·1)	4·5 (1·7–9·5)	4·5 (1·7–10·5)	-0·6 (-63·5–210·3)	3·7 (1·3–8·1)	3 (1·1–7)	-17·7 (-70·1–161·5)
Dominica	2·2 (0·7–5·1)	1·5 (0·5–3·8)	-28·7 (-75·9–148)	3·8 (1·5–8·7)	3·4 (1·3–7·5)	-12·3 (-67·1–170)	3 (1–6·9)	2·5 (0·9–5·9)	-17 (-68·7–172·7)
Dominican Republic	4·9 (1·6–11·9)	3·2 (1–6·9)	-35 (-76·2–108·5)	12·3 (4·6–25·9)	13·1 (4·5–29·7)	6·3 (-57·7–221·5)	8·5 (3·1–18·8)	8·1 (2·7–18)	-5 (-62·7–182·4)
Grenada	6·2 (2–13·9)	3 (0·9–7)	-52·2 (-82·1–54·6)	14·1 (5·5–30·7)	8·3 (3·1–18·5)	-41·3 (-77·5–73·5)	10 (3·6–22·3)	5·7 (2–12·9)	-43·3 (-77·6–78·2)
Guyana	7·1 (2·2–16·5)	3 (0·9–6·8)	-58·5 (-86·1–34·2)	11·3 (3·7–24·9)	5·5 (1·8–12·8)	-51·6 (-83–37·3)	9·2 (3–20·8)	4·2 (1·3–9·8)	-54·4 (-83·2–32·6)
Haiti	20·5 (6·9–45·9)	21·1 (6·7–49·4)	2·8 (-66·8–229·7)	22·9 (7·2–55·9)	24·7 (8–54·2)	7·8 (-63–248·6)	21·7 (7·4–48·2)	22·8 (7·8–51·1)	5·4 (-63·7–236·2)
Jamaica	3·7 (1·2–8·9)	2·3 (0·6–5·3)	-37·4 (-77·9–109·8)	5·3 (1·9–12·3)	5·2 (1·8–12)	-2 (-64·8–195·5)	4·5 (1·5–10·5)	3·7 (1·2–8·7)	-16·7 (-70·9–166·8)
Puerto Rico	1·7 (0·6–3·9)	0·6 (0·2–1·3)	-66·5 (-88·4–6·8)	4·7 (1·7–9·8)	1·8 (0·7–3·9)	-61·6 (-86·2–14·8)	3·1 (1·1–6·6)	1·2 (0·4–2·5)	-63·1 (-87–13·5)
Saint Kitts and Nevis	5 (1·6–11·5)	2·2 (0·6–5·1)	-55·6 (-84·3–47·9)	6·5 (2·5–14·4)	3·4 (1·1–7·4)	-48 (-81·3–58·2)	5·7 (1·9–13·1)	2·8 (0·8–6·4)	-51·5 (-82·2–54·2)
Saint Lucia	5·1 (1·7–12·4)	1·8 (0·5–4)	-65·2 (-88·4–19·3)	9·5 (3·6–19·4)	4·9 (1·7–11·2)	-49·2 (-83·7–55·8)	7·2 (2·7–16·2)	3·3 (1·1–7·6)	-54·3 (-85·1–46·8)
Saint Vincent and the Grenadines	3 (1–6·9)	1·6 (0·5–3·5)	-46·2 (-81·2–67·2)	9·1 (3·6–18·8)	7·3 (2·4–16·4)	-20·3 (-73·8–147·9)	6 (2·2–12·9)	4·5 (1·4–10·1)	-25·1 (-74–147·8)
Suriname	3·7 (1·2–9)	2·8 (0·8–6·8)	-25·1 (-75·2–145·2)	6 (2·2–13·4)	5·3 (1·7–11·9)	-12 (-68·2–144·6)	4·8 (1·7–11·2)	4 (1·3–9·3)	-17·9 (-71·2–143·6)
Trinidad and Tobago	5·7 (1·8–13·4)	2·9 (0·9–6·5)	-50·1 (-82·6–54)	8·6 (3·1–19·2)	5·7 (2–12·7)	-33·9 (-75·3–110·2)	7·2 (2·4–16·5)	4·3 (1·4–9·5)	-40·4 (-78·7–94·4)
United States Virgin Islands	2·2 (0·7–5)	0·8 (0·2–1·7)	-64·7 (-87·4–12·5)	3·9 (1·5–8·4)	3 (1·1–6·6)	-23·7 (-72·4–141)	3 (1·1–6·8)	1·8 (0·7–4·1)	-38·9 (-76·8–94)
Central Latin America	3·9 (1·3–9·3)	2 (0·6–4·8)	-49·2 (-82·4–64·4)	6·5 (2·5–14·5)	4·5 (1·5–9·7)	-31 (-75·9–115·6)	5·2 (1·9–12)	3·2 (1–7·1)	-38·3 (-79·1–96·1)
Colombia	5 (1·6–11·6)	1·7 (0·5–3·8)	-66·3 (-88·2–10·4)	6 (2·3–13·2)	2·6 (0·9–5·7)	-56·8 (-84·6–28·6)	5·5 (2–12·5)	2·1 (0·7–4·7)	-61·3 (-86·6–19)
Costa Rica	1·6 (0·5–3·7)	0·9 (0·3–2·1)	-41·6 (-79·9–89·6)	2·8 (1–6·2)	1·9 (0·7–4·1)	-31·3 (-74·9–101·8)	2·2 (0·8–5)	1·4 (0·5–3·1)	-34·8 (-77·1–94·7)
El Salvador	4·2 (1·4–10·2)	2·2 (0·7–5·1)	-46·6 (-81·4–77·9)	6·4 (2·3–14·6)	3·9 (1·3–8·4)	-39·4 (-76·6–90·6)	5·2 (1·8–12·3)	3 (0·9–6·5)	-43·2 (-80·1–73·3)
Guatemala	3·6 (1·2–8·4)	2 (0·6–4·7)	-43 (-81–89·5)	3·6 (1·4–7·8)	2·1 (0·7–4·9)	-39·6 (-78·1–89·1)	3·6 (1·3–8·1)	2·1 (0·6–4·8)	-41·4 (-79·5–88·6)

	Female			Male			Both		
	Age-standardized YLL rate (per 100,000)		Change (%)	Age-standardized YLL rate (per 100,000)		Change (%)	Age-standardized YLL rate (per 100,000)		Change (%)
	1990	2023		1990	2023		1990	2023	
Honduras	3·6 (1·1–8)	2·7 (0·9–6·6)	-26·2 (-75·6–138·4)	6·1 (2·2–14·8)	4·6 (1·6–11·8)	-24·6 (-76·1–151·8)	4·8 (1·6–11·5)	3·6 (1·3–8·4)	-25·7 (-75·1–127)
Mexico	2·3 (0·7–5·3)	1 (0·3–2·2)	-57·9 (-85·8–39·9)	2·8 (1–6·3)	1·6 (0·6–3·4)	-42·8 (-78·2–73)	2·5 (0·9–5·9)	1·3 (0·4–2·8)	-49·9 (-82·1–59·6)
Nicaragua	4 (1·4–9·8)	1·9 (0·6–4·6)	-53·7 (-83·6–46·3)	5·2 (1·8–12·5)	3 (1–6·5)	-42·6 (-79·4–76·4)	4·6 (1·6–10·9)	2·4 (0·8–5·8)	-47·5 (-80·9–66·2)
Panama	4·4 (1·4–10·4)	2·4 (0·8–5·5)	-45 (-80·4–82·7)	5·5 (2–12·7)	3·7 (1·3–8·4)	-32·4 (-74·7–104·4)	5 (1·7–11·6)	3·1 (1–6·9)	-38·2 (-77·6–93·3)
Venezuela (Bolivarian Republic of)	10·7 (3·5–24·8)	7·6 (2·1–18·9)	-28·9 (-75·6–135·1)	27·7 (10·7–60·6)	23·2 (7·3–50·7)	-16·3 (-70·6–156·9)	19 (7·1–42·6)	15 (4·4–33)	-21·5 (-72·5–156·9)
Tropical Latin America	10·8 (3·5–25·3)	4·4 (1·4–10)	-59·4 (-85·2–34·7)	22·6 (8·6–49·4)	13·2 (4·7–28·5)	-41·3 (-78·5–79·2)	16·5 (5·8–36·7)	8·6 (2·9–18·3)	-47·8 (-81·1–61·4)
Brazil	10·6 (3·4–24·9)	4·2 (1·4–9·6)	-59·9 (-85·3–33·4)	22·1 (8·4–48·2)	12·7 (4·5–27·4)	-42·5 (-78·8–75·8)	16·2 (5·7–35·9)	8·3 (2·7–17·6)	-48·7 (-81·4–58·8)
Paraguay	18·3 (6·4–42·5)	10·7 (3·4–23·6)	-41·4 (-79·4–90·9)	41·3 (15·6–93·7)	37·3 (13·5–79·3)	-9·6 (-67·6–164·8)	29·8 (10·7–67·8)	23·9 (8·2–49·6)	-19·8 (-72·2–138·1)
North Africa and Middle East	25 (9·2–51·3)	8·5 (4·3–16·6)	-66 (-84–-25·1)	59·6 (25·7–123)	24·1 (12·2–45·6)	-59·5 (-79·3–-12·4)	42·7 (17·8–84·9)	16·7 (8·4–30)	-61 (-79·9–-20·1)
Afghanistan	24·7 (7·9–55·8)	13·5 (4·3–32·4)	-45·2 (-82·6–73·7)	49·4 (16·1–119)	29·1 (9·5–70·4)	-41 (-80·5–111·9)	36·3 (11·9–86·2)	21·4 (7·5–48·6)	-41·2 (-81·3–94·1)
Algeria	17·9 (5·9–42)	6·9 (2·1–16·7)	-61·7 (-87·9–15·9)	80·3 (27·3–186)	42·1 (13·8–98·7)	-47·5 (-81·7–63·1)	49·2 (16·5–113)	24·6 (8·3–56·4)	-50 (-82·6–52·3)
Bahrain	11·6 (3·9–27·7)	2·1 (0·6–5·2)	-81·6 (-94·2–-41·2)	22·3 (7·5–52·9)	6·9 (2·2–15·5)	-69·2 (-89·8–-6·2)	18·2 (5·8–40·6)	5·2 (1·5–10·6)	-71·2 (-90·7–-14·7)
Egypt	7·6 (2·3–19·2)	4·4 (1·4–10·7)	-41·8 (-83·8–85·9)	33 (10·3–78·9)	20·2 (6·2–46·1)	-38·6 (-80·9–111·3)	20·6 (6·5–47·9)	12·6 (3·9–28·1)	-38·7 (-80·3–95·8)
Iran (Islamic Republic of)	13·8 (4·5–32·4)	2·7 (0·9–6·9)	-80·8 (-93·5–-42·6)	25·7 (8·2–62)	6·8 (2·2–15·7)	-73·6 (-91·5–-12·7)	19·9 (6·4–48)	4·8 (1·6–11·1)	-76·1 (-92·2–-23·3)
Iraq	8 (2·7–19·5)	4·2 (1·3–10)	-48·2 (-83·8–62·8)	14·7 (4·6–36·1)	9·8 (3·2–25·9)	-33·4 (-79·7–145·4)	11·5 (3·6–27·2)	7·1 (2·3–17·3)	-38·4 (-80·2–113·6)
Jordan	5·7 (1·8–13·7)	1·7 (0·5–4·2)	-69·3 (-90–1·9)	10·9 (3·2–26·7)	3 (1–7·1)	-72 (-92·2–-2·2)	8·4 (2·7–20·2)	2·5 (0·8–5·8)	-70·9 (-91–-1·6)
Kuwait	4·1 (1·4–9·4)	1·3 (0·4–3)	-69·2 (-89·5–-0·2)	8·2 (3–18)	3 (1–6·6)	-63·8 (-86·9–3·9)	6·5 (2·3–14·4)	2·4 (0·8–5·1)	-64 (-87·7–3·3)

	Female			Male			Both		
	Age-standardized YLL rate (per 100,000)		Change (%)	Age-standardized YLL rate (per 100,000)		Change (%)	Age-standardized YLL rate (per 100,000)		Change (%)
	1990	2023		1990	2023		1990	2023	
Lebanon	4 (1·2–9·6)	1·8 (0·6–4·1)	-54·3 (-85·5–59·7)	9·8 (2·8–23·8)	5·8 (1·9–13·2)	-40·2 (-83·2–127·5)	6·8 (2·1–16·8)	3·8 (1·2–8·3)	-44·2 (-82·7–90·1)
Libya	8·2 (2·7–19·1)	5·4 (1·6–12·1)	-34·6 (-81·2–120·4)	19·5 (6·2–47·8)	10·2 (3·1–23·7)	-47·6 (-84–71·8)	14·3 (4·6–32·6)	7·9 (2·7–17·6)	-44·9 (-81·3–77·9)
Morocco	13 (4–30·6)	7·2 (2·2–16·8)	-44·4 (-81·1–76·8)	13·5 (4–34·2)	8 (2·3–19·7)	-41·1 (-80·9–90·2)	13·3 (4·1–30·9)	7·6 (2·5–17·2)	-42·6 (-80·5–85·3)
Oman	9·7 (3·1–23)	4 (1·2–9·3)	-58·5 (-87·8–33·4)	22·1 (7–52·5)	8·8 (2·8–20·6)	-60·2 (-85·8–43·1)	17·7 (5·2–37·5)	7·2 (2·1–14·5)	-59·6 (-86·5–31·3)
Palestine	12 (3·9–30·4)	3·2 (1–7·5)	-73·8 (-91·8–-11·7)	28·4 (8·5–67·3)	7·8 (2·6–17·5)	-72·6 (-91–-10·9)	20·1 (6·3–48·8)	5·5 (1·8–12·2)	-72·7 (-91–-11·7)
Qatar	4·9 (1·6–10·9)	1 (0·3–2·4)	-80·2 (-94·2–-38·8)	5·5 (1·7–13·2)	1·8 (0·6–4·3)	-66·8 (-89·6–9·4)	5·3 (1·8–13·4)	1·6 (0·5–3·4)	-70·2 (-91·4–-14·2)
Saudi Arabia	12·1 (4·1–27·8)	5·5 (1·5–13·4)	-54·5 (-84·7–50)	14·3 (4·4–33·7)	5·9 (1·9–12·6)	-59 (-87·3–35·2)	13·4 (4·3–31·8)	5·7 (1·9–13)	-57·2 (-86·3–37·2)
Sudan	19·6 (6·1–44·9)	8 (2·5–20·5)	-59 (-87–34·6)	126 (37–311)	65·4 (20·8–163)	-47·9 (-82·1–56)	71·8 (23·1–180)	37·1 (11·6–89·7)	-48·3 (-82·9–47)
Syrian Arab Republic	4·4 (1·4–11)	2·1 (0·6–5·5)	-53·6 (-86·3–57·6)	5·9 (1·8–14·4)	2·9 (0·9–6·6)	-50 (-83·5–84·4)	5·2 (1·7–12·6)	2·5 (0·8–5·9)	-52 (-85–65·1)
Tunisia	44·3 (14·6–95·7)	12·7 (3·9–27·4)	-71·3 (-90·3–-10·2)	88·9 (28·7–202)	30·1 (10·5–69·8)	-66·1 (-88·4–11·6)	66·8 (21·6–149)	21·3 (7·5–47·2)	-68·1 (-88·2–0·6)
Türkiye	12·1 (4·1–28·1)	3·2 (0·9–8)	-73·3 (-92–-8·4)	16·3 (5·3–38·6)	5·4 (1·8–12·7)	-66·8 (-89·3–11)	14·2 (4·8–34·3)	4·3 (1·3–10)	-69·6 (-90·3–-1·2)
United Arab Emirates	3·2 (1–8·2)	1·1 (0·3–2·7)	-66·8 (-90·1–36·7)	8·7 (2·2–24·9)	3·3 (1·1–7·4)	-61·5 (-87·5–67·1)	7·1 (1·7–17)	2·8 (0·8–5·7)	-60·7 (-86·7–78·7)
Yemen	439 (166–876)	138 (66·4–261)	-68·7 (-85·2–-25·9)	1070 (532–1920)	326 (150–622)	-69·5 (-86·3–-33)	755 (352–1360)	232 (115–412)	-69·3 (-85·2–-30·3)
South Asia	404 (214–715)	335 (182–544)	-17 (-56–45·5)	595 (318–933)	586 (386–858)	-1·6 (-42·4–71·7)	503 (275–802)	462 (286–689)	-8·2 (-47–56·4)
Bangladesh	922 (493–1490)	1040 (626–1520)	13 (-33·6–81·7)	620 (309–954)	741 (534–981)	19·5 (-22·8–111·5)	766 (416–1150)	894 (594–1180)	16·7 (-23–95·7)
Bhutan	359 (157–678)	266 (133–446)	-26 (-66·8–68)	444 (189–827)	389 (223–636)	-12·3 (-53·1–74·7)	405 (187–703)	330 (189–520)	-18·5 (-56·6–60·9)
India	373 (193–685)	284 (144–488)	-23·8 (-61·9–43)	636 (342–1000)	600 (380–900)	-5·7 (-45·3–66·8)	510 (274–820)	445 (263–673)	-12·8 (-51·3–52)
Nepal	260 (114–496)	163 (74·1–305)	-37·2 (-71·4–34·3)	568 (287–908)	607 (407–880)	6·8 (-37·8–87·3)	412 (211–692)	376 (241–561)	-8·8 (-45·7–62·4)

	Female			Male			Both		
	Age-standardized YLL rate (per 100,000)		Change (%)	Age-standardized YLL rate (per 100,000)		Change (%)	Age-standardized YLL rate (per 100,000)		Change (%)
	1990	2023		1990	2023		1990	2023	
Pakistan	245 (107–475)	254 (111–466)	3·4 (-56·7–165·2)	289 (140–547)	330 (170–618)	13·9 (-51·9–136·5)	269 (130–488)	293 (147–528)	9 (-51·2–149·5)
**Southeast Asia, East Asia, and Oceania**	44·6 (16·7–90·9)	19·5 (8·5–39·5)	-56·2 (-82·4–14·6)	56·5 (21·9–127)	37·6 (15·2–77·7)	-33·4 (-74·3–83·5)	50·6 (19·7–110)	28·6 (11·6–57·3)	-43·4 (-77·2–50·1)
East Asia	13·5 (4·5–30·9)	4·2 (1·3–9·8)	-68·7 (-89·7–0·5)	39·5 (14·6–92)	20·8 (7·5–45·4)	-47·3 (-81·1–61·8)	26·8 (9·7–61·5)	12·6 (4·4–27·1)	-52·9 (-83·5–45·3)
China	13·8 (4·6–31·6)	4·3 (1·3–9·9)	-69 (-89·8–-0·7)	40·3 (14·9–94)	21 (7·6–45·7)	-48 (-81·3–59·4)	27·4 (10–62·9)	12·8 (4·4–27·4)	-53·5 (-83·7–43·3)
Democratic People's Republic of Korea	4·7 (1·6–10·6)	4·3 (1·3–11·6)	-8·4 (-69·1–175)	8·8 (2·9–21·1)	7·3 (2·3–17·1)	-16·5 (-74·1–186)	6·7 (2·1–14·3)	5·8 (1·8–12·9)	-13·5 (-68·8–183·9)
Taiwan	6·9 (2·3–16·4)	1·7 (0·5–3·9)	-75·3 (-90·8–-21·9)	22·1 (8·4–46·2)	21·9 (8·6–48·1)	-0·9 (-63·1–201·1)	14·8 (5·5–32·1)	11·6 (4·4–25·3)	-21·3 (-71·2–139·8)
Oceania	39·5 (13·9–96·4)	61 (24·4–124)	54·6 (-43·2–348·1)	60·2 (23·6–139)	55·8 (19·4–131)	-7·3 (-67–187·1)	50·1 (19–106)	58·4 (21·1–121)	16·4 (-54·5–230·9)
American Samoa	14·8 (5·1–33·2)	13·7 (4·4–31·3)	-7·1 (-71·7–199·2)	47·9 (16·2–112)	41·4 (14·7–98·3)	-13·5 (-70·8–197)	31·6 (10·6–73·8)	27·8 (10–63·5)	-12·1 (-69·7–185·9)
Cook Islands	15·5 (5·3–34·5)	8 (2·7–18·4)	-48·1 (-82·3–70·7)	41·8 (12·7–102)	38·7 (14·5–93·4)	-7·6 (-69·4–193·6)	29·2 (9·5–69·6)	22·9 (8·4–53·3)	-21·7 (-73·4–142·7)
Fiji	16·2 (5·7–37)	18·8 (5·6–41·7)	16 (-62·3–257·5)	40·4 (13·3–92·5)	35·4 (11·9–78·9)	-12·5 (-68·4–172·8)	28·4 (9·7–64·4)	27·2 (8·8–57·7)	-4·4 (-65·7–198·1)
Guam	6·5 (2·1–14·3)	14 (5·9–28·2)	116·6 (-15·8–539·5)	42·2 (17·4–86·6)	95·6 (51·6–162)	126·7 (2·4–438·2)	25·8 (10·6–53)	55·1 (28·9–96·9)	113·4 (-4·2–428·6)
Kiribati	14·1 (4·5–29·7)	21·3 (6·8–49·4)	51·3 (-52·3–352·4)	126 (48·8–276)	132 (54·7–284)	4·4 (-59·4–193)	68·3 (25·8–146)	74·6 (30·3–154)	9·2 (-57·2–198·6)
Marshall Islands	27·2 (8·5–65·2)	57·7 (21·6–136)	112·5 (-29·6–503·7)	102 (38·7–220)	118 (50·4–252)	15·6 (-57·8–254·2)	65·8 (25–150)	88·7 (37·5–184)	34·9 (-47·6–301·2)
Micronesia (Federated States of)	29·3 (9·8–68·4)	25·2 (7·8–56·6)	-14·1 (-73·1–167·5)	141 (52·3–306)	85·3 (32–200)	-39·3 (-77·9–94·4)	85·7 (30·7–188)	55·6 (20·8–124)	-35·1 (-76·9–100·1)
Nauru	18·7 (5–45·8)	23·1 (6·9–54·1)	23·2 (-62·6–311·7)	31·4 (8·6–82·2)	29·1 (10–68)	-7·3 (-71·1–207·6)	25·2 (7·6–65·9)	26·1 (9·2–58)	3·5 (-64·7–248·9)
Niue	14·1 (4·7–32)	14·1 (4·6–32·7)	-0·5 (-67·9–236·4)	26 (8·1–59·1)	23·2 (7·5–50·3)	-10·9 (-69–181·4)	20 (6·4–47·2)	18·4 (6·2–41·2)	-7·9 (-67·9–186·2)

	Female			Male			Both		
	Age-standardized YLL rate (per 100,000)		Change (%)	Age-standardized YLL rate (per 100,000)		Change (%)	Age-standardized YLL rate (per 100,000)		Change (%)
	1990	2023		1990	2023		1990	2023	
Northern Mariana Islands	20·2 (6·9–46·1)	25·1 (9·3–51·5)	24·1 (-57·4–280·6)	64 (23·2–144)	91 (35·5–192)	42·1 (-49·1–308·3)	43·7 (16–102)	59·6 (23·7–125)	36·3 (-51·5–284·5)
Palau	324 (152–579)	375 (193–648)	15·8 (-46–143·6)	609 (276–1140)	510 (255–913)	-16·2 (-62·9–91·2)	472 (223–861)	449 (237–744)	-4·8 (-55·6–103·1)
Papua New Guinea	49 (16·9–125)	70·5 (28·3–148)	43·8 (-47·3–341·8)	54·8 (19·6–126)	48·1 (15–114)	-12·2 (-69·5–215·9)	52 (19·3–112)	58·9 (21·8–125)	13·3 (-57·1–219·2)
Samoa	10·8 (3·3–25·1)	12·4 (3·9–31·1)	14·8 (-66·3–274·8)	21·5 (7·8–49·1)	20·5 (6·6–50·4)	-4·7 (-68·1–253·2)	16·4 (5·5–37·2)	16·5 (5·5–40·2)	0·3 (-65·3–234·7)
Solomon Islands	36·6 (10·4–86·9)	61·2 (19·1–134)	67·2 (-44·6–453)	133 (45·5–299)	146 (53·9–327)	9·8 (-57·1–242·3)	86·5 (32–203)	104 (36·7–222)	20·7 (-55·8–258·6)
Tokelau	11·2 (3·6–25·1)	12·5 (4–29·5)	11·6 (-65–284·7)	38·7 (13–88·8)	30·5 (10·3–67·5)	-21·2 (-71·8–177·7)	24·7 (8–58·1)	21·6 (7·4–48·5)	-12·5 (-68·1–190·5)
Tonga	7·9 (2·5–17·3)	9·4 (2·9–21·5)	19·3 (-64·3–282·3)	14·7 (5·2–34·8)	12·1 (3·9–29)	-17·7 (-74–166·3)	11·2 (3·7–25·3)	10·6 (3·7–24·8)	-5·3 (-67·5–194·1)
Tuvalu	19 (6·1–43·6)	17·1 (5·4–39·8)	-10·4 (-75·1–190·6)	48·4 (15·5–116)	37 (11·2–82·7)	-23·6 (-73·8–175·2)	32·4 (10·2–77·6)	27·2 (8·8–57·9)	-16 (-71·4–174·3)
Vanuatu	19·5 (6·2–44·7)	28 (8·7–68·3)	43·5 (-54·2–342·2)	78·2 (25·9–188)	78 (26·6–176)	-0·3 (-64·5–230·3)	49·5 (16·6–126)	52·8 (18·3–119)	6·8 (-63·7–235·6)
Southeast Asia	145 (56·3–288)	68·9 (30·7–135)	-52·4 (-79·4–17·1)	114 (45·1–252)	86·9 (37·7–173)	-23·4 (-67·3–95·2)	130 (52·2–261)	77·9 (34·6–151)	-39·9 (-74·7–44·1)
Cambodia	576 (268–1060)	351 (174–645)	-39·1 (-72·3–34·7)	81 (29·8–207)	50 (18·9–122)	-38·3 (-79·1–102·3)	352 (175–655)	207 (125–400)	-41·2 (-64·8–26·4)
Indonesia	150 (55·3–309)	57·6 (19·4–130)	-61·6 (-86·1–5·5)	24·4 (8·3–57·7)	18·1 (6·1–42·7)	-25·7 (-73·3–140·4)	88·3 (34–187)	37·7 (13·5–89·4)	-57·3 (-83·9–18·2)
Lao People's Democratic Republic	317 (115–701)	315 (139–589)	-0·7 (-61·1–155·9)	31·1 (9·6–80·5)	25·2 (8·3–60)	-19 (-74·5–186·5)	180 (66·9–394)	170 (74·7–330)	-6 (-62·6–149·3)
Malaysia	45·3 (14·9–106)	26·9 (9·8–62·2)	-40·8 (-80·5–82·8)	55·7 (19·6–129)	55 (20·4–118)	-1·2 (-64·7–220·2)	50·5 (17·2–120)	41·7 (15–90·6)	-17·5 (-71·2–152·8)
Maldives	271 (93·1–600)	92·8 (31·7–194)	-65·8 (-87·6–3·8)	249 (101–537)	106 (46·1–212)	-57·4 (-82·1–0·4)	260 (97·9–542)	101 (40·2–191)	-61 (-84·3–1·4)
Mauritius	75 (27·2–153)	40·2 (13·7–88·2)	-46·5 (-79·5–72·4)	49·2 (19·1–107)	35·8 (12·6–79·2)	-27·3 (-71·4–114)	62·2 (24–139)	38 (13·7–85·8)	-38·9 (-76·8–82·1)

	Female			Male			Both		
	Age-standardized YLL rate (per 100,000)		Change (%)	Age-standardized YLL rate (per 100,000)		Change (%)	Age-standardized YLL rate (per 100,000)		Change (%)
	1990	2023		1990	2023		1990	2023	
Myanmar	380 (141–770)	254 (89·2–522)	-33·1 (-76·1–85·4)	686 (275–1440)	683 (312–1310)	-0·4 (-57·8–141·8)	529 (208–1070)	457 (193–860)	-13·6 (-63·9–109·6)
Philippines	37·4 (13·5–81·5)	24·2 (7·9–53·7)	-35·3 (-77·3–87·2)	70·8 (25–165)	74·2 (29·3–159)	4·8 (-58–214·5)	54·1 (18·9–123)	49·3 (17·3–95)	-8·7 (-66·8–140·3)
Seychelles	52·6 (18–108)	26·9 (9·5–60·4)	-48·8 (-82·7–53·1)	65·4 (24·8–151)	48·7 (17·4–106)	-25·6 (-75·1–136)	59 (23–132)	39 (14·7–86·8)	-33·8 (-76·6–94·9)
Sri Lanka	128 (47·6–263)	82·7 (29·4–169)	-35·1 (-75·7–78·5)	379 (162–718)	322 (150–576)	-14·9 (-61·3–93·5)	253 (106–486)	197 (84·9–352)	-22·3 (-67–77)
Thailand	123 (48·2–248)	44·1 (16·5–88·3)	-64·2 (-86·2–-7·4)	56·2 (20·2–135)	37·5 (14–83·2)	-33·2 (-75·7–104·1)	90·3 (36·6–191)	41 (15·3–87·3)	-54·6 (-83·2–19·6)
Timor-Leste	169 (61·5–361)	101 (33·1–230)	-39·9 (-80·4–89·4)	53·6 (18·3–137)	37·8 (12·1–86·2)	-29·4 (-76·2–135·8)	110 (40·8–246)	69·1 (23·5–149)	-37 (-78·8–84·4)
Viet Nam	81·1 (27·4–186)	34·6 (11·7–74·8)	-57·3 (-83·4–24)	37·9 (12·7–85·4)	20·4 (7·4–45·3)	-46·2 (-80·3–68·6)	60·9 (22·6–151)	27·6 (10·4–67·1)	-54·6 (-82·7–32·7)
**Sub-Saharan Africa**	34·2 (12·4–70·5)	27·7 (10·2–61·7)	-19 (-69·1–128·2)	43 (18·4–90·1)	36·6 (15·8–78·8)	-15 (-66·3–120·2)	38·5 (15·5–82·5)	32 (12·9–66·6)	-17 (-68–107·8)
Central Sub-Saharan Africa	31·1 (10·8–68·7)	19·6 (6·2–47·8)	-37·1 (-78–101·2)	24·8 (7·6–58·1)	24·8 (8·6–60·7)	-0·3 (-67·5–233·2)	28 (9·7–62)	22·1 (7·6–50·4)	-21 (-71·3–149·3)
Angola	9·6 (3·2–21)	6·9 (2·2–16)	-27·8 (-76·2–129·2)	14·9 (4·6–36·8)	12·6 (3·8–31·2)	-15·2 (-71·4–195·8)	12·3 (4·2–29·9)	9·6 (3–21·7)	-21·7 (-71·3–157)
Central African Republic	36·4 (12–84·3)	37·6 (10·4–86·8)	3·4 (-67·6–241·4)	18·8 (5·2–44·6)	16·8 (5·6–39)	-11 (-70·3–211·9)	27·9 (9·5–67·9)	27·5 (8·9–62·4)	-1·6 (-66·5–220·3)
Congo	27·2 (9·3–59·7)	24·4 (8–57·2)	-10 (-69·4–203·5)	11·6 (3·7–26·6)	12·4 (4·1–29·8)	7·5 (-63·9–273·7)	19·6 (7–45·2)	18·5 (6·6–45·5)	-5·7 (-65·9–206·5)
Democratic Republic of the Congo	38·1 (13·1–83·6)	23·2 (6·8–57·5)	-39 (-78·7–90·7)	29·6 (9·2–68·9)	30·9 (11–77·7)	4·4 (-66·3–243·4)	34 (11·7–73·2)	27·1 (9–62·2)	-20·3 (-71·8–152·5)
Equatorial Guinea	27·4 (9·2–60·1)	14·2 (4·7–31·1)	-48·2 (-83–79·3)	23·1 (7·2–56·6)	17·9 (5·8–45·7)	-22·4 (-75·5–185·4)	25·4 (8·6–59·7)	16·2 (5·3–37·1)	-36·3 (-78–110·5)
Gabon	8·8 (3–19·9)	6·6 (2–15)	-25·2 (-75·5–150·6)	4·7 (1·6–11·3)	4·5 (1·5–10·5)	-4·4 (-67·5–243·1)	6·8 (2·3–16·3)	5·6 (1·8–13·2)	-17·5 (-72–155·8)
Eastern Sub-Saharan Africa	41·9 (16·7–85·9)	35·2 (14·3–74·9)	-16 (-66·6–119·7)	77·3 (34·4–156)	63·8 (29·7–133)	-17·4 (-65·7–93·4)	59·1 (26·3–120)	49·2 (21·9–98·2)	-16·9 (-66·6–95·5)

	Female			Male			Both		
	Age-standardized YLL rate (per 100,000)		Change (%)	Age-standardized YLL rate (per 100,000)		Change (%)	Age-standardized YLL rate (per 100,000)		Change (%)
	1990	2023		1990	2023		1990	2023	
Burundi	49·6 (17·8–108)	49·3 (17·1–100)	-0·7 (-65·6–227·8)	128 (48·8–263)	96 (37·8–238)	-25·3 (-73·7–128·8)	87·1 (31·7–177)	72·8 (27·1–169)	-16·4 (-69·3–164·2)
Comoros	79·6 (31·2–163)	41·3 (16–87·5)	-48 (-80·6–32·9)	115 (46·9–240)	73·6 (30·2–153)	-36 (-74·9–76·7)	96·8 (40–201)	57·5 (22·7–115)	-40·6 (-76·6–49·6)
Djibouti	26 (8·6–58·8)	16·7 (5·7–36·3)	-35·8 (-78·1–88·6)	42·4 (16·8–93·2)	28·5 (9·5–61·8)	-32·9 (-74·1–104·5)	34·6 (13·1–81·7)	23 (7·8–49·6)	-33·5 (-75·5–100·4)
Eritrea	8·2 (2·6–18·7)	7·8 (2·6–19·4)	-4·4 (-68·3–209·5)	99·1 (34·2–228)	82·8 (30·6–183)	-16·5 (-70·3–156·7)	51·9 (17·2–112)	46·3 (16·2–95·7)	-10·7 (-68·1–166·4)
Ethiopia	7·3 (2·2–18)	9·9 (3·6–21)	35·6 (-58·2–374·9)	40·7 (14·8–90·4)	54·3 (21·5–129)	33·6 (-56·2–346·8)	23·8 (9·2–57·7)	32 (12·7–76·6)	34·4 (-56·1–304·6)
Kenya	29·4 (10·8–62·3)	21·2 (7·4–46·1)	-28 (-74·4–122·1)	38·1 (14·8–85·6)	28·2 (9·7–65·3)	-25·8 (-74–112·8)	33·7 (13·3–72·6)	24·6 (8·3–53·8)	-26·8 (-74·1–125·9)
Madagascar	242 (102–457)	203 (96·2–394)	-16·1 (-62·6–99·7)	657 (311–1240)	424 (225–778)	-35·6 (-67·8–39·4)	447 (211–844)	310 (165–543)	-30·6 (-66·8–46·6)
Malawi	58·5 (20·5–127)	42·1 (15·6–93·3)	-28 (-73·2–109·7)	29·3 (10·3–70·6)	25·8 (8·4–60·4)	-12 (-69·4–189·3)	44·4 (16·2–97·4)	34·3 (12·2–80·2)	-22·7 (-71–136·6)
Mozambique	26·9 (9·3–60·2)	19·2 (6–42·5)	-28·8 (-75·9–118·1)	34·7 (11·7–83·2)	31·1 (10·5–74·2)	-10·3 (-70·6–158·6)	30·5 (10·4–67·2)	24·7 (8·3–55·4)	-18·9 (-72–133·5)
Rwanda	94·5 (34·5–198)	58·8 (21·5–121)	-37·8 (-76·2–89·5)	19·2 (6·4–44·4)	14·4 (5·1–32·6)	-24·8 (-73·7–152·2)	58·5 (22·2–128)	37·6 (14·2–79·2)	-35·7 (-74·9–92·7)
Somalia	12·9 (4–32·6)	14·7 (5–33·4)	13·9 (-61·9–269·4)	19·8 (6·7–44·2)	19·6 (6·3–49)	-1·1 (-66·3–238·9)	16·4 (5·7–39·4)	17·1 (5·8–39·1)	4·3 (-63·8–252)
South Sudan	38·7 (14·3–81·5)	38·6 (13·5–85·2)	-0·1 (-65–187·2)	66·9 (23·7–160)	53·8 (18·6–124)	-19·6 (-71·9–158·2)	53·7 (20·7–128)	46 (15·9–102)	-14·3 (-71–155·2)
Uganda	45·7 (16·7–98·2)	38·4 (13·1–89·9)	-16 (-68·9–173·9)	20·6 (7·4–44·5)	21·8 (7·5–51·3)	5·5 (-64·9–226·5)	33·6 (12·9–67·5)	30·4 (11·1–73·4)	-9·4 (-66·9–184·2)
United Republic of Tanzania	19·6 (6·7–44·2)	14·1 (4·5–31·9)	-27·8 (-75·4–125·2)	24·2 (8·8–55·2)	20·2 (7·1–44·7)	-16·8 (-70·9–151·9)	21·8 (8–49·3)	17 (5·8–37·5)	-21·8 (-72·3–149·6)
Zambia	31·8 (9·3–80·1)	36·1 (11·8–83·2)	13·6 (-64·5–283·2)	23·3 (8·3–52·8)	27·1 (9–63·3)	16·5 (-58·4–310·1)	27·7 (9·1–61·2)	31·9 (11·2–71·6)	15·1 (-60·5–261·1)
Southern Sub-Saharan Africa	39·4 (14–81·7)	33·6 (11·1–77·4)	-14·8 (-69·1–149·1)	18·3 (7–41·6)	13·3 (4·7–28·7)	-27·2 (-74·6–123·6)	29·4 (11·3–66·5)	23·9 (8·8–60·6)	-18·7 (-69·1–137·5)

	Female			Male			Both		
	Age-standardized YLL rate (per 100,000)		Change (%)	Age-standardized YLL rate (per 100,000)		Change (%)	Age-standardized YLL rate (per 100,000)		Change (%)
	1990	2023		1990	2023		1990	2023	
Botswana	128 (53·3–250)	77·3 (32·5–154)	-39·8 (-76·4–66·5)	48 (17·4–107)	45·3 (16·9–101)	-5·6 (-65–182·7)	91·1 (37·4–188)	61·9 (26·5–128)	-32·1 (-72·8–79·7)
Eswatini	21·2 (7·3–45·4)	16·6 (5·6–36)	-21·6 (-75·5–165·8)	49·6 (18·2–116)	24·5 (8·5–59·4)	-50·5 (-83–59·9)	34 (12·9–78·9)	20·5 (6·8–42·9)	-39·9 (-78·9–86·4)
Lesotho	37·9 (12·4–83·5)	35·4 (11·5–86·7)	-6·6 (-67·3–191)	31·8 (11·3–72·3)	28 (9·1–60·1)	-11·9 (-69·9–184·6)	35·3 (12·5–80·5)	31·8 (12–80·9)	-9·9 (-68·4–194·8)
Namibia	48 (16·8–104)	31·5 (9·6–66·3)	-34·5 (-76·1–91·8)	30·5 (9·9–73·3)	18·9 (6·6–42·8)	-38·1 (-79–99·5)	39·6 (14·8–96·7)	25·4 (9·3–56·6)	-35·9 (-76–81·2)
South Africa	41·3 (14·6–83·7)	33·1 (10·6–75·9)	-19·8 (-71·1–135·7)	17·8 (6·8–40·4)	12·2 (4·2–26·2)	-31·5 (-76·8–114·1)	30·1 (11·6–69·2)	23·1 (8·4–59·7)	-23·4 (-70·8–134·2)
Zimbabwe	17·9 (6–37·4)	29·8 (9·7–64·9)	66·2 (-41·2–415·3)	10·7 (3·8–24·3)	9·7 (3·5–24·2)	-8·8 (-68·5–197·1)	14·4 (5·4–34·4)	20·4 (7·3–46·9)	41·4 (-51·7–328·7)
Western Sub-Saharan Africa	24·5 (7·9–53·6)	20·7 (7·2–46·9)	-15·6 (-72·6–162·9)	24·8 (9·1–57·4)	21·8 (8·5–51·5)	-12 (-70·1–176)	24·7 (8·8–59·1)	21·3 (7·7–46·5)	-13·9 (-68·5–154·2)
Benin	31·4 (10·1–67·6)	22·2 (7·6–51·7)	-29·4 (-75·7–121·1)	25·6 (9·1–63·3)	17·5 (5·9–40·8)	-31·5 (-78·3–120·8)	28·8 (9·4–63·6)	19·9 (6·9–45·3)	-30·6 (-73·2–109·5)
Burkina Faso	26·1 (8·8–56·4)	22·3 (7·9–47)	-14·5 (-72·3–159·3)	36·1 (13–84·3)	23·1 (8·5–54·2)	-36·2 (-79·5–112·8)	30·7 (10·9–69·5)	22·7 (8·5–48·6)	-26·2 (-73·9–121·5)
Cabo Verde	10·4 (3·6–22·2)	5·4 (1·7–11·7)	-47·6 (-82·5–72·5)	9·3 (3·3–22·4)	8 (2·7–17)	-14·3 (-71·3–174·5)	9·9 (3·3–22·2)	6·7 (2·2–15)	-32·1 (-76–125·8)
Cameroon	81·1 (26·3–213)	79·4 (29–173)	-2·1 (-68·5–187·4)	24·1 (8·4–52·8)	21·3 (7·1–51·5)	-11·9 (-73·3–199·4)	53·9 (17·5–134)	50·7 (18·7–116)	-5·9 (-68·1–178·9)
Chad	52·1 (16·9–109)	57·5 (18·4–138)	10·4 (-62·9–271·5)	59·5 (21·5–144)	61 (22·9–141)	2·5 (-64·9–221·5)	55·6 (18·9–119)	59·2 (21·4–134)	6·6 (-60·3–237·1)
Côte d'Ivoire	25·6 (8·6–60·8)	16·1 (5·4–35·2)	-37 (-79·3–96·9)	9·4 (3–21·5)	8·8 (3·1–21·4)	-6·1 (-68·4–208·6)	17·3 (5·7–40·5)	12·3 (4·2–29)	-28·9 (-74·7–135·2)
Gambia	16·8 (5·7–37·9)	18·8 (5·8–44·9)	11·5 (-66·6–278·1)	12·1 (3·8–29·8)	10·7 (3·7–27·5)	-11·1 (-72·4–203·6)	14·5 (4·6–34·5)	14·9 (5·2–35·9)	2·8 (-64·9–240·3)
Ghana	23·1 (7·2–52·3)	18·3 (5·6–45·1)	-20·8 (-73·1–189)	49·2 (15·1–118)	38·1 (11·9–94·4)	-22·7 (-73·5–159·3)	35·7 (11·4–83·2)	27·9 (8·6–64·7)	-21·8 (-72·2–154·6)
Guinea	25·9 (8·3–55·7)	22 (7·2–50·3)	-15·3 (-72·6–179·3)	30 (9·5–79·1)	22·8 (7·8–56·8)	-23·9 (-74·6–171·7)	27·8 (9·5–64·7)	22·4 (7·7–50·6)	-19·7 (-71·4–160)

	Female			Male			Both		
	Age-standardized YLL rate (per 100,000)		Change (%)	Age-standardized YLL rate (per 100,000)		Change (%)	Age-standardized YLL rate (per 100,000)		Change (%)
	1990	2023		1990	2023		1990	2023	
Guinea-Bissau	28 (9–62·3)	30·1 (9·8–71·8)	7·2 (-64·4–247·3)	18·2 (5·7–44·3)	17·7 (5·6–41·7)	-2·8 (-67·3–219·8)	23·4 (7·4–54·4)	24·2 (8·3–57·9)	3·1 (-62·2–222·9)
Liberia	24·5 (8–54·1)	22·1 (7–55·5)	-9·8 (-72·4–187·8)	37·6 (12–96·4)	28·7 (9·7–67·3)	-23·8 (-76·1–165·1)	31·1 (10·2–76·9)	25·4 (8·9–60·7)	-18·2 (-73·1–176·5)
Mali	23·1 (7·2–50·9)	19·6 (6–47)	-15·3 (-72·1–170·1)	63·4 (26–143)	56·7 (22·8–128)	-10·6 (-68·4–154·2)	42·3 (16·7–104)	37·8 (14·9–90·2)	-10·7 (-66·9–158·3)
Mauritania	25·4 (8·4–57·4)	21·2 (6·7–52·6)	-16·7 (-73·2–146·1)	25·9 (9·3–61·5)	21·2 (7–53·5)	-18·1 (-75·6–165·8)	25·6 (8·8–61·5)	21·2 (7·1–49·2)	-17·4 (-72·9–137·8)
Niger	31·2 (9·5–71·4)	31·2 (9·2–79·8)	-0·1 (-70·1–221·5)	98·7 (39·1–229)	86·8 (32·6–182)	-12·1 (-69·1–164)	64·2 (24·9–154)	58·3 (23·2–123)	-9·1 (-69·5–157·6)
Nigeria	13·9 (4·3–31·5)	10 (3·3–24·2)	-28·4 (-78·1–135·8)	10·9 (3·7–25·4)	8·8 (3–23)	-19·2 (-74·8–191·7)	12·4 (4·4–31·1)	9·4 (3·3–21·6)	-23·8 (-75·7–123·2)
Sao Tome and Principe	9·8 (3·2–21·3)	6·9 (2–17·1)	-29·3 (-78·2–129·6)	13 (4·2–30·6)	15·4 (5·1–36·4)	18·5 (-62·1–278·7)	11·4 (3·6–25·4)	11·2 (3·7–24·5)	-1·5 (-68·4–199·4)
Senegal	25·6 (8·3–59·5)	25·3 (8·1–57·8)	-1·4 (-66·5–216·4)	10·8 (3·5–27·1)	8·2 (2·9–19·9)	-24·1 (-75·5–150·7)	18·5 (5·9–42)	16·9 (5·6–39·8)	-8·9 (-66·1–206·9)
Sierra Leone	61·6 (21·9–146)	49·2 (15·5–108)	-20·1 (-72·6–154·5)	46·4 (14·7–105)	39·5 (12·7–92·7)	-14·8 (-71·8–179·2)	54·2 (18·3–130)	44·4 (14·9–96·5)	-18·1 (-69·9–153·9)
Togo	45·7 (15·3–97·5)	35·9 (11·6–88·6)	-21·5 (-74·2–136·8)	35·4 (11·1–85·5)	23·5 (7·1–58·6)	-33·7 (-77–112)	40·9 (13·6–95·7)	30 (9·9–72·1)	-26·7 (-73·6–138·1)

Appendix Table 2·10: Number of YLD attributable to chewing tobacco use in 1990 and 2023, among ages 15+

	Female			Male			Both		
	Number of YLDs (thousands)		Change (%)	Number of YLDs (thousands)		Change (%)	Number of YLDs (thousands)		Change (%)
	1990	2023		1990	2023		1990	2023	
Global	47·6 (19·1–96)	113 (56·1–223)	137 (20·6–374)	86·9 (41·9–158)	194 (108–346)	123 (34·2–273)	134 (60·3–254)	307 (164–568)	128 (29·3–306)
**Central Europe, Eastern Europe, and Central Asia**	0·67 (0·204–1·7)	0·773 (0·255–1·99)	15·4 (-61–278)	0·979 (0·324–2·36)	1·45 (0·524–3·52)	48·4 (-46–335)	1·65 (0·537–4·04)	2·23 (0·779–5·49)	35 (-52–313)
Central Asia	0·0945 (0·0288–0·241)	0·135 (0·0432–0·354)	42·5 (-51·4–359)	0·364 (0·125–0·838)	0·611 (0·229–1·45)	67·6 (-35–346)	0·459 (0·153–1·08)	0·746 (0·273–1·81)	62·5 (-38·7–349)
Armenia	0·00284 (0·000827–0·00711)	0·00322 (0·000998–0·00842)	13·1 (-60·8–282)	0·00259 (0·00082–0·00649)	0·00319 (0·00111–0·00782)	23 (-56·5–280)	0·00544 (0·00165–0·0134)	0·00641 (0·00213–0·0162)	17·8 (-58·5–280)
Azerbaijan	0·0049 (0·00153–0·0126)	0·00786 (0·00248–0·0206)	60·3 (-44–401)	0·00731 (0·0024–0·0181)	0·011 (0·00385–0·0272)	50·3 (-43·8–343)	0·0122 (0·0039–0·0308)	0·0188 (0·00635–0·048)	54·3 (-44·8–371)
Georgia	0·0094 (0·00285–0·0239)	0·00709 (0·00225–0·0186)	-24·6 (-73·8–142)	0·00637 (0·00204–0·0163)	0·00509 (0·00174–0·0132)	-20 (-71·1–148)	0·0158 (0·00495–0·0399)	0·0122 (0·00402–0·0318)	-22·7 (-72·7–145)
Kazakhstan	0·0173 (0·00514–0·0434)	0·0175 (0·00558–0·0455)	0·8 (-66–231)	0·0301 (0·00956–0·0741)	0·0302 (0·0103–0·0759)	0·5 (-63·9–217)	0·0474 (0·0147–0·117)	0·0477 (0·0158–0·122)	0·6 (-65–222)
Kyrgyzstan	0·0118 (0·00357–0·0291)	0·0105 (0·00332–0·027)	-11·3 (-69·1–184)	0·102 (0·0363–0·228)	0·129 (0·053–0·277)	26·5 (-49·8–224)	0·114 (0·0398–0·255)	0·139 (0·0559–0·302)	22·5 (-51·8–214)
Mongolia	0·00149 (0·00046–0·00377)	0·0023 (0·000745–0·00591)	54·8 (-47·3–413)	0·00229 (0·000741–0·00576)	0·00311 (0·00104–0·0078)	35·8 (-51·1–316)	0·00378 (0·00119–0·00953)	0·00542 (0·00178–0·0135)	43·3 (-49·8–353)
Tajikistan	0·0126 (0·00385–0·0323)	0·0256 (0·00825–0·0657)	103 (-30·3–545)	0·00777 (0·00252–0·0187)	0·0144 (0·00493–0·0371)	85·6 (-33·7–457)	0·0204 (0·00639–0·051)	0·04 (0·0132–0·103)	96·3 (-32·4–504)
Turkmenistan	0·00274 (0·000837–0·00685)	0·005 (0·00159–0·013)	82·8 (-37·3–510)	0·00318 (0·00103–0·00789)	0·00662 (0·00229–0·0172)	108 (-27·2–555)	0·00592 (0·00185–0·0147)	0·0116 (0·00391–0·0302)	96·3 (-32·5–540)
Uzbekistan	0·0314 (0·00977–0·0818)	0·0557 (0·0178–0·148)	77·3 (-39·6–457)	0·203 (0·0676–0·477)	0·408 (0·144–0·993)	101 (-22·4–475)	0·234 (0·0774–0·555)	0·464 (0·161–1·14)	98 (-25·4–476)
Central Europe	0·172 (0·052–0·426)	0·261 (0·0863–0·668)	51·6 (-47·5–400)	0·261 (0·0843–0·644)	0·431 (0·15–1·06)	65·1 (-40·7–406)	0·434 (0·137–1·07)	0·693 (0·236–1·72)	59·7 (-43·3–404)
Albania	0·00283 (0·000868–0·0072)	0·00441 (0·00143–0·0116)	55·8 (-43·3–417)	0·00698 (0·00225–0·0169)	0·00937 (0·00312–0·0235)	34·3 (-49·5–315)	0·00981 (0·00311–0·024)	0·0138 (0·00455–0·0356)	40·5 (-47·7–344)
Bosnia and Herzegovina	0·0062 (0·00188–0·0156)	0·00754 (0·00244–0·0202)	21·6 (-56·7–282)	0·00972 (0·00307–0·0234)	0·0131 (0·00443–0·0326)	34·7 (-50·8–311)	0·0159 (0·00495–0·039)	0·0206 (0·00685–0·0535)	29·6 (-53·8–294)

	Female			Male			Both		
	Number of YLDs (thousands)		Change (%)	Number of YLDs (thousands)		Change (%)	Number of YLDs (thousands)		Change (%)
	1990	2023		1990	2023		1990	2023	
Bulgaria	0·0293 (0·00884–0·0712)	0·043 (0·0135–0·112)	46·6 (-49·2–387)	0·0488 (0·0161–0·12)	0·0686 (0·0238–0·165)	40·4 (-49–335)	0·0782 (0·0252–0·192)	0·112 (0·0373–0·277)	42·7 (-49·9–349)
Croatia	0·0055 (0·00168–0·0134)	0·00516 (0·00172–0·0131)	-6·1 (-67·3–216)	0·00749 (0·00235–0·0185)	0·00895 (0·00298–0·0219)	19·4 (-57·1–263)	0·013 (0·00412–0·0317)	0·0141 (0·00472–0·0351)	8·6 (-61·3–242)
Czechia	0·0194 (0·00578–0·0471)	0·0255 (0·00827–0·0651)	31·2 (-53·4–331)	0·0244 (0·00766–0·0583)	0·0401 (0·0136–0·0962)	64·3 (-38·5–416)	0·0438 (0·0135–0·106)	0·0655 (0·0218–0·162)	49·6 (-46·1–380)
Hungary	0·0168 (0·00518–0·0421)	0·0142 (0·00454–0·0351)	-15·7 (-70·9–177)	0·0201 (0·00629–0·051)	0·017 (0·00582–0·0403)	-15·4 (-69·1–152)	0·0369 (0·0116–0·0931)	0·0312 (0·0104–0·0753)	-15·5 (-69·6–163)
Montenegro	0·000772 (0·000239–0·00207)	0·00126 (0·000403–0·00338)	62·7 (-43·3–439)	0·00119 (0·000383–0·00282)	0·00219 (0·000754–0·00525)	83·7 (-36–480)	0·00196 (0·00062–0·00489)	0·00344 (0·00116–0·00854)	75·5 (-39·2–471)
North Macedonia	0·00282 (0·000847–0·00716)	0·00441 (0·00142–0·012)	56·7 (-45·2–421)	0·00425 (0·00137–0·0103)	0·00852 (0·00285–0·0215)	101 (-30·9–536)	0·00706 (0·00226–0·0175)	0·0129 (0·00426–0·0336)	83·1 (-36·7–490)
Poland	0·039 (0·012–0·0972)	0·1 (0·0342–0·252)	157 (-8·6–762)	0·05 (0·0162–0·125)	0·143 (0·0496–0·344)	186 (1·5–794)	0·089 (0·0284–0·223)	0·243 (0·0838–0·593)	173 (-2·8–782)
Romania	0·0257 (0·00783–0·0657)	0·0247 (0·00814–0·06)	-3·9 (-65·5–228)	0·0511 (0·0161–0·124)	0·0658 (0·023–0·159)	28·6 (-52·9–289)	0·0768 (0·024–0·19)	0·0905 (0·0312–0·219)	17·7 (-56·9–266)
Serbia	0·0134 (0·00408–0·0337)	0·0168 (0·00537–0·0435)	25·6 (-56·5–319)	0·0217 (0·00686–0·0535)	0·0312 (0·0105–0·0754)	43·7 (-49·7–347)	0·0351 (0·011–0·0873)	0·048 (0·0159–0·117)	36·8 (-52·6–337)
Slovakia	0·0058 (0·00176–0·0144)	0·00589 (0·0019–0·0153)	1·5 (-63·7–214)	0·00842 (0·00274–0·02)	0·00902 (0·0031–0·0217)	7·2 (-62·1–239)	0·0142 (0·00448–0·0344)	0·0149 (0·005–0·0365)	4·9 (-62·2–234)
Slovenia	0·00191 (0·000594–0·00484)	0·0041 (0·00134–0·0103)	115 (-23·5–601)	0·00294 (0·000932–0·00722)	0·00818 (0·0027–0·0195)	178 (-2–771)	0·00485 (0·00151–0·0121)	0·0123 (0·00406–0·0302)	153 (-9–703)
Eastern Europe	0·403 (0·123–1·03)	0·377 (0·125–0·979)	-6·5 (-68·9–206)	0·353 (0·113–0·883)	0·411 (0·144–1·01)	16·3 (-58·9–266)	0·756 (0·24–1·91)	0·788 (0·269–1·96)	4·1 (-64·2–235)
Belarus	0·015 (0·00438–0·0384)	0·0136 (0·00433–0·0349)	-9·3 (-68·8–197)	0·0176 (0·00559–0·0424)	0·0222 (0·00765–0·0514)	26·4 (-55·3–301)	0·0326 (0·0101–0·0808)	0·0358 (0·0119–0·0857)	9·9 (-61·5–255)
Estonia	0·00224 (0·000701–0·00567)	0·00287 (0·000938–0·00732)	28·4 (-56·3–313)	0·00214 (0·000693–0·00535)	0·00519 (0·00182–0·0125)	143 (-9·9–624)	0·00437 (0·00138–0·011)	0·00806 (0·00276–0·0197)	84·2 (-33·4–474)
Latvia	0·00421 (0·00129–0·0108)	0·00305 (0·001–0·00797)	-27·7 (-74·9–138)	0·00349 (0·00113–0·00885)	0·00296 (0·00102–0·00719)	-15·1 (-68·9–158)	0·0077 (0·00242–0·0195)	0·00601 (0·00203–0·015)	-22 (-71·9–147)
Lithuania	0·00445 (0·00131–0·0115)	0·00444 (0·00143–0·0114)	-0·2 (-64·4–218)	0·00345 (0·00107–0·00887)	0·00449 (0·00154–0·0113)	30·3 (-53·2–291)	0·00789 (0·00242–0·0205)	0·00893 (0·00298–0·0229)	13·1 (-59·4–253)

	Female			Male			Both		
	Number of YLDs (thousands)		Change (%)	Number of YLDs (thousands)		Change (%)	Number of YLDs (thousands)		Change (%)
	1990	2023		1990	2023		1990	2023	
Republic of Moldova	0·0045 (0·00135–0·0119)	0·00426 (0·00135–0·0108)	-5·4 (-67·6–221)	0·00661 (0·00213–0·0166)	0·00889 (0·00306–0·022)	34·5 (-51–317)	0·0111 (0·00349–0·0282)	0·0132 (0·00442–0·0332)	18·3 (-57·9–279)
Russian Federation	0·281 (0·0864–0·716)	0·284 (0·0934–0·731)	1·1 (-66·1–234)	0·213 (0·0678–0·539)	0·28 (0·0983–0·693)	31·7 (-54·4–313)	0·494 (0·153–1·25)	0·564 (0·192–1·4)	14·3 (-61·1–271)
Ukraine	0·0913 (0·0279–0·242)	0·0643 (0·0213–0·168)	-29·6 (-77·2–127)	0·107 (0·0338–0·262)	0·0869 (0·0303–0·212)	-19·1 (-70·9–152)	0·199 (0·0619–0·494)	0·151 (0·0521–0·38)	-23·9 (-73·3–143)
**High-income**	1·61 (0·508–4·09)	2·88 (0·954–7·28)	79 (-39–462)	9·58 (5·11–17·6)	14·3 (7·3–26·5)	48·8 (-14·5–159)	11·2 (5·79–21·8)	17·1 (8·65–33·1)	53·2 (-17·1–190)
Australasia	0·0194 (0·00588–0·0502)	0·0384 (0·0122–0·0954)	98·1 (-33·3–546)	0·0661 (0·021–0·164)	0·13 (0·0452–0·318)	95·9 (-28·3–506)	0·0855 (0·0271–0·216)	0·168 (0·0576–0·414)	96·4 (-28·9–518)
Australia	0·0157 (0·00474–0·0408)	0·0311 (0·00977–0·077)	98·7 (-33–551)	0·0567 (0·0181–0·14)	0·111 (0·0381–0·273)	95·8 (-28·2–506)	0·0724 (0·023–0·183)	0·142 (0·048–0·35)	96·4 (-28·7–517)
New Zealand	0·0037 (0·00113–0·00938)	0·00722 (0·00236–0·0183)	95·2 (-34·4–545)	0·00937 (0·0029–0·0235)	0·0184 (0·00677–0·0441)	96·4 (-30·5–513)	0·0131 (0·00406–0·0333)	0·0256 (0·0092–0·0619)	96 (-31·3–521)
High-income Asia Pacific	0·325 (0·0974–0·844)	0·7 (0·231–1·77)	115 (-24·3–597)	0·929 (0·298–2·33)	1·83 (0·655–4·46)	97·4 (-28·9–496)	1·25 (0·395–3·2)	2·53 (0·89–6·21)	102 (-27·6–522)
Brunei Darussalam	0·000664 (0·000198–0·00169)	0·00187 (0·000614–0·00463)	181 (2·8–824)	0·00112 (0·000371–0·00268)	0·00284 (0·000957–0·00723)	153 (-7·4–685)	0·00179 (0·000572–0·00438)	0·00471 (0·00157–0·0118)	164 (-2·7–737)
Japan	0·24 (0·0721–0·624)	0·535 (0·176–1·36)	123 (-21·4–620)	0·708 (0·225–1·76)	1·4 (0·51–3·4)	98·1 (-27·9–505)	0·948 (0·298–2·41)	1·94 (0·69–4·71)	104 (-26·3–533)
Republic of Korea	0·0798 (0·0239–0·206)	0·146 (0·047–0·377)	83·1 (-35·4–499)	0·205 (0·0678–0·497)	0·373 (0·122–0·917)	81·7 (-34·7–439)	0·285 (0·0918–0·704)	0·519 (0·169–1·31)	82·1 (-35·4–458)
Singapore	0·00446 (0·00132–0·0116)	0·0166 (0·00529–0·0425)	273 (25·1–1150)	0·0147 (0·00468–0·0369)	0·0557 (0·0183–0·136)	277 (38–1050)	0·0192 (0·00606–0·0485)	0·0723 (0·0237–0·177)	276 (35·1–1070)
High-income North America	0·922 (0·297–2·26)	1·61 (0·537–4·08)	74·8 (-38·4–430)	8·07 (4·51–13·8)	11·3 (6·2–18·9)	39·6 (-13–123)	9 (4·89–16)	12·9 (6·73–23·1)	43·2 (-15·9–144)
Canada	0·0836 (0·0259–0·213)	0·167 (0·0524–0·436)	99·2 (-30·7–535)	0·512 (0·178–1·19)	0·536 (0·184–1·31)	4·6 (-62·3–194)	0·596 (0·204–1·39)	0·702 (0·241–1·75)	17·9 (-58·1–240)
Greenland	8·62e-05 (2·62e-05–0·000217)	0·000116 (3·74e-05–0·00029)	34·7 (-52·6–345)	0·00109 (0·000369–0·00262)	0·00123 (0·000439–0·00292)	13 (-56·2–230)	0·00117 (0·000395–0·00283)	0·00134 (0·000476–0·00322)	14·6 (-55·9–239)
United States of America	0·838 (0·269–2·05)	1·44 (0·484–3·66)	72·3 (-39·1–416)	7·56 (4·29–12·9)	10·7 (5·8–18·3)	42 (-10·1–124)	8·4 (4·64–14·6)	12·2 (6·49–21·7)	45 (-12·6–138)

	Female			Male			Both		
	Number of YLDs (thousands)		Change (%)	Number of YLDs (thousands)		Change (%)	Number of YLDs (thousands)		Change (%)
	1990	2023		1990	2023		1990	2023	
Southern Latin America	0·034 (0·0102–0·0885)	0·0474 (0·0152–0·124)	39·6 (-51·3–359)	0·0339 (0·011–0·0847)	0·0424 (0·0145–0·109)	25·1 (-55·8–290)	0·0679 (0·0213–0·173)	0·0898 (0·0299–0·232)	32·3 (-53·8–321)
Argentina	0·02 (0·00597–0·0522)	0·0264 (0·00843–0·0686)	31·8 (-54·3–339)	0·0171 (0·00572–0·0431)	0·0215 (0·00719–0·0545)	25·5 (-55·7–289)	0·0371 (0·0117–0·0964)	0·0479 (0·0158–0·124)	28·9 (-55·1–310)
Chile	0·0108 (0·00324–0·0271)	0·018 (0·00572–0·0473)	67·2 (-41·6–443)	0·0137 (0·00431–0·0338)	0·0182 (0·00646–0·0465)	33·4 (-51·6–309)	0·0244 (0·00755–0·0609)	0·0362 (0·0122–0·0948)	48·3 (-47·1–365)
Uruguay	0·0032 (0·000978–0·00819)	0·00305 (0·000986–0·00773)	-4·7 (-66·8–217)	0·00311 (0·000987–0·00778)	0·0027 (0·000877–0·00641)	-13·4 (-69·4–177)	0·00632 (0·00195–0·016)	0·00575 (0·00187–0·014)	-9 (-68·8–197)
Western Europe	0·309 (0·0933–0·802)	0·484 (0·156–1·24)	56·8 (-45·7–414)	0·48 (0·153–1·21)	0·983 (0·35–2·34)	105 (-28·7–531)	0·789 (0·248–2·01)	1·47 (0·504–3·6)	85·9 (-35·2–486)
Andorra	2·93e-05 (8·72e-06–7·76e-05)	6·88e-05 (2·18e-05–0·000179)	135 (-19·1–681)	6·03e-05 (1·98e-05–0·000147)	0·000162 (5·36e-05–0·000371)	169 (-4·1–739)	8·96e-05 (2·89e-05–0·000225)	0·000231 (7·54e-05–0·00055)	158 (-10·1–718)
Austria	0·00755 (0·00227–0·0188)	0·0144 (0·00465–0·0378)	90·1 (-35·2–521)	0·0112 (0·0035–0·0279)	0·0325 (0·0111–0·0778)	191 (4·5–791)	0·0187 (0·00584–0·0465)	0·0469 (0·0157–0·116)	150 (-11·5–700)
Belgium	0·0071 (0·00212–0·0179)	0·00782 (0·00249–0·0198)	10·1 (-62·2–258)	0·0094 (0·00304–0·0236)	0·0111 (0·0037–0·0263)	18·1 (-56·8–256)	0·0165 (0·00514–0·0412)	0·0189 (0·00615–0·0457)	14·7 (-59–257)
Cyprus	0·000346 (0·000104–0·000918)	0·000607 (0·000199–0·0016)	75·1 (-38·2–463)	0·00059 (0·000195–0·0015)	0·00115 (0·000377–0·00276)	95·7 (-30·6–491)	0·000936 (0·000301–0·00241)	0·00176 (0·000576–0·00433)	88·1 (-33·4–488)
Denmark	0·00614 (0·00188–0·0162)	0·00946 (0·00306–0·0243)	54 (-46·7–399)	0·0142 (0·00454–0·0348)	0·0227 (0·00776–0·0548)	59·6 (-42·4–388)	0·0203 (0·00647–0·051)	0·0321 (0·0108–0·0798)	57·9 (-43·6–388)
Finland	0·00441 (0·00137–0·0114)	0·00643 (0·00211–0·0164)	45·9 (-49·6–363)	0·0056 (0·00177–0·014)	0·0137 (0·00469–0·0334)	145 (-12·4–643)	0·01 (0·00312–0·0254)	0·0201 (0·00684–0·0502)	101 (-28·8–517)
France	0·0326 (0·00985–0·0836)	0·0565 (0·0184–0·148)	73·3 (-43·4–453)	0·0571 (0·0191–0·14)	0·0907 (0·0303–0·211)	59 (-44·3–365)	0·0897 (0·0293–0·225)	0·147 (0·0487–0·356)	64·2 (-45·2–398)
Germany	0·0852 (0·0262–0·216)	0·163 (0·052–0·416)	90·8 (-34·4–528)	0·0933 (0·0297–0·23)	0·307 (0·107–0·744)	229 (19·5–907)	0·178 (0·0561–0·447)	0·47 (0·158–1·17)	163 (-6·4–734)
Greece	0·00937 (0·00287–0·0246)	0·00994 (0·00319–0·0257)	6·1 (-62·5–231)	0·0125 (0·00393–0·0319)	0·0144 (0·00492–0·0344)	14·9 (-59·6–257)	0·0219 (0·00695–0·0564)	0·0243 (0·00812–0·0598)	11·1 (-61·3–251)
Iceland	0·00022 (6·79e-05–0·000567)	0·000478 (0·000152–0·00116)	117 (-24·9–614)	0·000675 (0·000208–0·00165)	0·0024 (0·000829–0·0058)	256 (27·5–990)	0·000895 (0·000276–0·00222)	0·00288 (0·000983–0·00697)	222 (15–898)
Ireland	0·00195 (0·00058–0·00509)	0·00279 (0·000911–0·00684)	42·8 (-50·9–366)	0·00268 (0·000853–0·00683)	0·00428 (0·00144–0·0098)	59·9 (-41·6–386)	0·00463 (0·00144–0·0119)	0·00707 (0·00234–0·0166)	52·7 (-45·5–387)

	Female			Male			Both		
	Number of YLDs (thousands)		Change (%)	Number of YLDs (thousands)		Change (%)	Number of YLDs (thousands)		Change (%)
	1990	2023		1990	2023		1990	2023	
Israel	0·00253 (0·000771–0·00653)	0·00539 (0·00169–0·014)	113 (-25·5–583)	0·00491 (0·00158–0·012)	0·01 (0·00343–0·025)	104 (-28–534)	0·00744 (0·00236–0·0185)	0·0154 (0·0051–0·0392)	107 (-27·1–547)
Italy	0·0322 (0·00959–0·0854)	0·0442 (0·0144–0·11)	37·3 (-52·4–352)	0·0554 (0·0181–0·137)	0·0923 (0·0337–0·223)	66·5 (-39·2–413)	0·0876 (0·0277–0·222)	0·136 (0·0483–0·328)	55·8 (-45·8–394)
Luxembourg	0·00028 (8·75e-05–0·000723)	0·000319 (0·000104–0·000813)	14·1 (-61·3–262)	0·000357 (0·000115–0·000905)	0·00051 (0·000171–0·00119)	42·8 (-49·8–337)	0·000637 (0·000204–0·00163)	0·000829 (0·000275–0·00199)	30·2 (-54·7–308)
Malta	0·000187 (5·61e-05–0·000499)	0·000293 (9·56e-05–0·000761)	56·8 (-43·8–416)	0·000301 (9·6e-05–0·000755)	0·000519 (0·000178–0·0012)	72·3 (-39–428)	0·000488 (0·000153–0·00124)	0·000812 (0·000272–0·00196)	66·3 (-40·9–432)
Monaco	4·68e-05 (1·42e-05–0·000124)	4·91e-05 (1·56e-05–0·000124)	4·8 (-63·8–253)	9·05e-05 (2·87e-05–0·000234)	0·000106 (3·41e-05–0·00024)	16·9 (-61·5–253)	0·000137 (4·4e-05–0·000355)	0·000155 (4·99e-05–0·000352)	12·7 (-62·4–253)
Netherlands	0·0108 (0·00326–0·0276)	0·014 (0·00451–0·0354)	29·7 (-54·8–324)	0·0126 (0·00404–0·0316)	0·02 (0·00688–0·0469)	58·5 (-43·4–381)	0·0234 (0·00734–0·0591)	0·034 (0·0115–0·0821)	45·2 (-49·7–356)
Norway	0·00607 (0·00181–0·0158)	0·0115 (0·00388–0·0286)	89·2 (-32·4–500)	0·0164 (0·00534–0·0405)	0·0547 (0·0203–0·129)	233 (23·1–897)	0·0225 (0·00718–0·0562)	0·0662 (0·0242–0·157)	194 (8·5–797)
Portugal	0·0121 (0·00369–0·0314)	0·0103 (0·00332–0·0267)	-14·8 (-69·2–188)	0·0124 (0·00392–0·0313)	0·0165 (0·0055–0·0384)	32·6 (-51·2–318)	0·0245 (0·00748–0·062)	0·0268 (0·00885–0·0653)	9·2 (-58·9–257)
San Marino	1·82e-05 (5·36e-06–4·7e-05)	4·02e-05 (1·31e-05–0·000104)	121 (-23·3–625)	3·57e-05 (1·17e-05–8·81e-05)	7·65e-05 (2·55e-05–0·000184)	115 (-23·8–548)	5·39e-05 (1·72e-05–0·000134)	0·000117 (3·86e-05–0·000288)	117 (-24·6–574)
Spain	0·0269 (0·00801–0·0709)	0·0352 (0·0114–0·09)	30·9 (-53–338)	0·04 (0·0132–0·103)	0·0624 (0·0211–0·146)	55·9 (-47·5–374)	0·0669 (0·0208–0·177)	0·0976 (0·0319–0·234)	45·8 (-49·4–368)
Sweden	0·0089 (0·00277–0·0231)	0·0316 (0·0102–0·0829)	255 (26·2–1030)	0·0645 (0·0213–0·156)	0·138 (0·0507–0·327)	114 (-19·9–554)	0·0734 (0·0241–0·179)	0·17 (0·0612–0·409)	131 (-14·4–611)
Switzerland	0·00464 (0·00141–0·012)	0·00677 (0·00221–0·017)	45·8 (-48·9–362)	0·0079 (0·00249–0·0196)	0·0131 (0·00453–0·0306)	66 (-40·9–399)	0·0125 (0·00399–0·0315)	0·0199 (0·00672–0·0475)	58·6 (-44–394)
United Kingdom	0·049 (0·0146–0·127)	0·0531 (0·0173–0·129)	8·4 (-62·8–254)	0·0576 (0·0183–0·147)	0·0732 (0·0257–0·173)	27 (-52·7–298)	0·107 (0·033–0·274)	0·126 (0·043–0·308)	18·5 (-57·3–279)
**Latin America and Caribbean**	0·328 (0·101–0·855)	0·71 (0·235–1·81)	116 (-23–601)	0·377 (0·125–0·933)	0·849 (0·295–2·09)	125 (-17·8–596)	0·705 (0·225–1·81)	1·56 (0·53–3·89)	121 (-20–599)
Andean Latin America	0·022 (0·00667–0·0569)	0·0357 (0·0114–0·0917)	62·8 (-43·6–424)	0·0209 (0·00675–0·0514)	0·039 (0·0131–0·0993)	86·3 (-32–471)	0·0429 (0·0135–0·108)	0·0748 (0·0245–0·192)	74·2 (-37·7–456)
Bolivia (Plurinational State of)	0·0029 (0·000873–0·00766)	0·0054 (0·00176–0·014)	86·1 (-34–527)	0·00355 (0·00114–0·00889)	0·00699 (0·00241–0·0174)	96·9 (-25·6–517)	0·00645 (0·00204–0·0166)	0·0124 (0·00411–0·0318)	92 (-30·2–521)

	Female			Male			Both		
	Number of YLDs (thousands)		Change (%)	Number of YLDs (thousands)		Change (%)	Number of YLDs (thousands)		Change (%)
	1990	2023		1990	2023		1990	2023	
Ecuador	0·0033 (0·000994–0·00872)	0·0072 (0·00228–0·0185)	118 (-26·8–621)	0·00283 (0·00093–0·00713)	0·00582 (0·00194–0·0152)	105 (-25·7–544)	0·00613 (0·00191–0·0159)	0·013 (0·00422–0·0336)	112 (-26·4–583)
Peru	0·0158 (0·00479–0·0405)	0·0231 (0·00746–0·0593)	46·9 (-48·7–367)	0·0146 (0·00468–0·0354)	0·0262 (0·0087–0·0666)	79·9 (-35·2–442)	0·0303 (0·00944–0·0759)	0·0494 (0·0162–0·127)	62·8 (-42–410)
Caribbean	0·024 (0·00733–0·0625)	0·0444 (0·0142–0·115)	85 (-36–509)	0·0293 (0·00944–0·0734)	0·0601 (0·0208–0·145)	105 (-25·9–526)	0·0533 (0·0169–0·138)	0·104 (0·0353–0·26)	95·9 (-30·4–511)
Antigua and Barbuda	4·41e-05 (1·34e-05–0·000113)	7·36e-05 (2·32e-05–0·000191)	66·7 (-42·1–436)	4·78e-05 (1·54e-05–0·000119)	8·04e-05 (2·72e-05–0·000199)	68·2 (-41–411)	9·19e-05 (2·93e-05–0·000232)	0·000154 (5·09e-05–0·000392)	67·5 (-41·5–420)
Bahamas	0·000128 (4·02e-05–0·000331)	0·000281 (9·18e-05–0·000712)	120 (-24·2–626)	0·000175 (5·73e-05–0·000441)	0·000383 (0·000129–0·000894)	119 (-23·3–604)	0·000303 (9·69e-05–0·000781)	0·000663 (0·000219–0·00164)	119 (-24·2–616)
Barbados	0·000269 (8·26e-05–0·000697)	0·00041 (0·000128–0·00105)	52·1 (-46·2–394)	0·000127 (4·07e-05–0·000324)	0·000222 (7·55e-05–0·000533)	74·9 (-36·9–454)	0·000397 (0·000124–0·00103)	0·000632 (0·000204–0·00159)	59·4 (-43–413)
Belize	6·67e-05 (2·01e-05–0·000175)	0·000187 (6e-05–0·000495)	180 (-5·2–818)	5·86e-05 (1·9e-05–0·000145)	0·00017 (5·74e-05–0·000423)	190 (8·2–785)	0·000125 (4·02e-05–0·000326)	0·000357 (0·000117–0·00092)	185 (0·6–800)
Bermuda	4·57e-05 (1·43e-05–0·000118)	6·91e-05 (2·23e-05–0·000182)	51·3 (-48–397)	5·26e-05 (1·74e-05–0·000127)	8·1e-05 (2·7e-05–0·000186)	53·9 (-44·9–381)	9·83e-05 (3·08e-05–0·000246)	0·00015 (4·94e-05–0·000357)	52·7 (-46·4–387)
Cuba	0·00785 (0·00242–0·0202)	0·0126 (0·00406–0·0322)	60·9 (-45·1–426)	0·00786 (0·00253–0·0194)	0·0153 (0·00525–0·037)	94 (-29–495)	0·0157 (0·00483–0·0399)	0·0279 (0·00926–0·0694)	77·5 (-36·8–462)
Dominica	3·78e-05 (1·16e-05–9·72e-05)	4·54e-05 (1·49e-05–0·000119)	19·9 (-57–295)	3·46e-05 (1·13e-05–8·5e-05)	4·97e-05 (1·72e-05–0·000122)	43·5 (-45·9–356)	7·25e-05 (2·3e-05–0·000182)	9·51e-05 (3·23e-05–0·000241)	31·2 (-51·5–324)
Dominican Republic	0·00384 (0·00119–0·00982)	0·00796 (0·00253–0·0201)	107 (-28·1–592)	0·00767 (0·00256–0·0186)	0·0186 (0·00662–0·0446)	143 (-5·4–580)	0·0115 (0·0037–0·0285)	0·0266 (0·00926–0·0639)	131 (-12·9–585)
Grenada	9·81e-05 (3·06e-05–0·000263)	0·000135 (4·35e-05–0·000351)	38 (-51·7–355)	0·000101 (3·24e-05–0·000255)	0·000181 (6·02e-05–0·000463)	79·9 (-35·7–455)	0·000199 (6·38e-05–0·000518)	0·000316 (0·000105–0·000822)	59·2 (-44·1–409)
Guyana	0·000358 (0·000106–0·000915)	0·000414 (0·000133–0·00108)	15·7 (-59·1–287)	0·000399 (0·000124–0·00102)	0·000458 (0·000158–0·00114)	14·7 (-57·9–259)	0·000757 (0·000234–0·00194)	0·000872 (0·000292–0·00222)	15·2 (-58·2–272)
Haiti	0·00482 (0·00146–0·013)	0·0122 (0·00384–0·0313)	152 (-14·6–720)	0·0063 (0·00205–0·0162)	0·0146 (0·00511–0·0365)	131 (-16·3–611)	0·0111 (0·00353–0·0292)	0·0267 (0·00895–0·0681)	140 (-16·8–651)
Jamaica	0·00152 (0·000456–0·00402)	0·00225 (0·000711–0·00585)	48·4 (-48·5–393)	0·00143 (0·00046–0·00358)	0·00231 (0·000779–0·00574)	61·5 (-40·2–415)	0·00295 (0·000923–0·00759)	0·00456 (0·00149–0·0115)	54·8 (-44–398)
Puerto Rico	0·00262 (0·000803–0·00671)	0·00393 (0·00126–0·0103)	50 (-47·7–387)	0·00248 (0·000819–0·00627)	0·00311 (0·00104–0·00766)	25·5 (-56·2–276)	0·0051 (0·00159–0·0132)	0·00704 (0·00236–0·018)	38·1 (-52·6–332)

	Female			Male			Both		
	Number of YLDs (thousands)		Change (%)	Number of YLDs (thousands)		Change (%)	Number of YLDs (thousands)		Change (%)
	1990	2023		1990	2023		1990	2023	
Saint Kitts and Nevis	3·04e-05 (9·07e-06–7·86e-05)	2·92e-05 (9·58e-06–7·75e-05)	-3·9 (-67·6–214)	2·1e-05 (6·83e-06–5·24e-05)	2·76e-05 (9·41e-06–6·8e-05)	31·2 (-52·1–299)	5·14e-05 (1·6e-05–0·000133)	5·68e-05 (1·9e-05–0·000146)	10·5 (-61·1–252)
Saint Lucia	0·000102 (3·12e-05–0·00026)	0·000146 (4·75e-05–0·000379)	42·4 (-50–374)	9·1e-05 (2·96e-05–0·000231)	0·000167 (5·82e-05–0·000409)	83·7 (-35–466)	0·000193 (6·18e-05–0·000494)	0·000313 (0·000106–0·000787)	61·9 (-42·7–425)
Saint Vincent and the Grenadines	5·32e-05 (1·59e-05–0·000134)	7·32e-05 (2·38e-05–0·000193)	37·6 (-51·3–350)	7·83e-05 (2·55e-05–0·000196)	0·000145 (4·85e-05–0·000339)	84·8 (-34·5–458)	0·000132 (4·17e-05–0·000334)	0·000218 (7·3e-05–0·000532)	65·7 (-40·9–414)
Suriname	0·000218 (6·61e-05–0·000561)	0·000461 (0·000147–0·00119)	112 (-27·2–578)	0·00027 (8·55e-05–0·000659)	0·000498 (0·000171–0·0012)	84·5 (-30·7–458)	0·000488 (0·000152–0·00121)	0·000959 (0·000315–0·00247)	96·6 (-28·5–514)
Trinidad and Tobago	0·00103 (0·000313–0·00267)	0·00157 (0·000502–0·00412)	52·6 (-44·6–403)	0·00109 (0·00036–0·00274)	0·00167 (0·000569–0·00416)	53·8 (-43·9–380)	0·00212 (0·000664–0·0054)	0·00325 (0·00111–0·00828)	53·2 (-44·8–391)
United States Virgin Islands	3·99e-05 (1·23e-05–0·000104)	6·7e-05 (2·12e-05–0·000172)	67·8 (-42–444)	3·99e-05 (1·28e-05–0·000102)	8·03e-05 (2·76e-05–0·000192)	101 (-28·1–536)	7·99e-05 (2·55e-05–0·000206)	0·000147 (4·88e-05–0·000373)	84·5 (-34·8–494)
Central Latin America	0·106 (0·0321–0·272)	0·232 (0·0757–0·594)	119 (-22·2–601)	0·105 (0·0339–0·26)	0·215 (0·0755–0·537)	106 (-23·9–536)	0·21 (0·0669–0·533)	0·447 (0·152–1·13)	112 (-23–569)
Colombia	0·03 (0·00905–0·0749)	0·0597 (0·0191–0·153)	99·1 (-31–568)	0·0225 (0·00724–0·0555)	0·0436 (0·0151–0·109)	94·2 (-27–487)	0·0525 (0·0166–0·13)	0·103 (0·0343–0·261)	97 (-29–531)
Costa Rica	0·00119 (0·000365–0·00306)	0·00302 (0·000965–0·00778)	154 (-10·8–724)	0·00116 (0·000373–0·00288)	0·00291 (0·00101–0·00706)	150 (-13–651)	0·00235 (0·000745–0·00602)	0·00593 (0·00201–0·015)	152 (-11·8–680)
El Salvador	0·00382 (0·00115–0·0101)	0·00563 (0·00181–0·0141)	47·5 (-48·6–392)	0·00322 (0·00102–0·00803)	0·00384 (0·0013–0·00942)	19·4 (-55·7–263)	0·00704 (0·00216–0·0182)	0·00948 (0·00311–0·0238)	34·6 (-52·3–339)
Guatemala	0·00286 (0·000876–0·00722)	0·00721 (0·00229–0·0182)	152 (-14·3–753)	0·00216 (0·000695–0·00533)	0·0051 (0·00174–0·0128)	136 (-15·2–617)	0·00502 (0·00158–0·0125)	0·0123 (0·00403–0·0314)	145 (-12·9–674)
Honduras	0·00209 (0·000652–0·00544)	0·00615 (0·002–0·0158)	194 (0·6–867)	0·003 (0·000957–0·00752)	0·00768 (0·00268–0·0191)	156 (-8·5–648)	0·0051 (0·00161–0·013)	0·0138 (0·00468–0·0357)	171 (-4·7–741)
Mexico	0·0353 (0·0106–0·0907)	0·0781 (0·0259–0·198)	122 (-22·3–607)	0·0263 (0·00851–0·065)	0·0634 (0·0221–0·157)	141 (-14–634)	0·0616 (0·019–0·157)	0·142 (0·0482–0·358)	130 (-18·6–619)
Nicaragua	0·00239 (0·000729–0·00626)	0·00542 (0·00172–0·0139)	127 (-20·8–641)	0·00201 (0·000635–0·00488)	0·00432 (0·00146–0·0107)	115 (-19·9–575)	0·0044 (0·00137–0·0111)	0·00974 (0·00318–0·0248)	121 (-22·2–603)
Panama	0·00225 (0·000679–0·00577)	0·00547 (0·00177–0·0138)	144 (-14·8–698)	0·00175 (0·000565–0·00436)	0·00383 (0·0013–0·00957)	119 (-21·9–578)	0·00399 (0·00126–0·0101)	0·0093 (0·00311–0·0234)	133 (-18·3–642)
Venezuela (Bolivarian Republic of)	0·0261 (0·00794–0·0674)	0·061 (0·02–0·153)	133 (-16·8–660)	0·0424 (0·0138–0·105)	0·0808 (0·0282–0·191)	90·3 (-26·5–470)	0·0686 (0·0218–0·172)	0·142 (0·0479–0·348)	107 (-22·9–544)

	Female			Male			Both		
	Number of YLDs (thousands)		Change (%)	Number of YLDs (thousands)		Change (%)	Number of YLDs (thousands)		Change (%)
	1990	2023		1990	2023		1990	2023	
Tropical Latin America	0·176 (0·0545–0·46)	0·398 (0·134–1)	126 (-19·4–636)	0·222 (0·0743–0·543)	0·535 (0·184–1·29)	140 (-12·9–640)	0·399 (0·128–1·02)	0·933 (0·317–2·27)	134 (-15·5–642)
Brazil	0·167 (0·0516–0·436)	0·381 (0·128–0·957)	128 (-18·5–645)	0·209 (0·0697–0·509)	0·506 (0·174–1·22)	142 (-12·2–647)	0·376 (0·121–0·959)	0·887 (0·302–2·16)	136 (-14·8–648)
Paraguay	0·00909 (0·00283–0·0236)	0·0169 (0·00555–0·0431)	86·1 (-32·1–486)	0·0134 (0·0045–0·0332)	0·0286 (0·00989–0·0695)	113 (-22·1–536)	0·0225 (0·00736–0·0568)	0·0455 (0·0153–0·113)	102 (-25·9–529)
North Africa and Middle East	0·6 (0·204–1·41)	1·1 (0·45–2·56)	83 (-18·9–351)	1·48 (0·539–3·27)	3·1 (1·39–6·74)	110 (2·4–366)	2·08 (0·744–4·67)	4·2 (1·84–9·34)	102 (-3·4–354)
Afghanistan	0·0118 (0·00371–0·0306)	0·0239 (0·00797–0·0622)	102 (-30·2–576)	0·0567 (0·018–0·135)	0·24 (0·0938–0·564)	323 (55–1080)	0·0685 (0·0216–0·164)	0·264 (0·102–0·626)	285 (43·9–993)
Algeria	0·0351 (0·0105–0·0881)	0·0767 (0·0249–0·199)	118 (-19·1–589)	0·193 (0·0649–0·467)	0·441 (0·164–1·03)	128 (-13·1–508)	0·228 (0·076–0·555)	0·517 (0·188–1·23)	126 (-14·2–517)
Bahrain	0·000562 (0·000174–0·00142)	0·00149 (0·000479–0·00376)	165 (-9·6–709)	0·00169 (0·000541–0·00398)	0·00543 (0·00181–0·0134)	220 (12·5–880)	0·00226 (0·000714–0·00537)	0·00691 (0·0023–0·0175)	206 (5·4–838)
Egypt	0·042 (0·0126–0·106)	0·0709 (0·0225–0·188)	68·9 (-39·3–452)	0·123 (0·0394–0·304)	0·246 (0·0819–0·625)	100 (-27·7–513)	0·165 (0·0517–0·401)	0·317 (0·104–0·82)	92·2 (-31–496)
Iran (Islamic Republic of)	0·0681 (0·0205–0·174)	0·117 (0·0383–0·307)	72·4 (-39·6–444)	0·13 (0·0417–0·319)	0·24 (0·0821–0·591)	85·6 (-34·3–467)	0·198 (0·0634–0·492)	0·358 (0·122–0·905)	81 (-36·1–459)
Iraq	0·0188 (0·00567–0·0478)	0·0424 (0·0141–0·109)	126 (-22·1–609)	0·0263 (0·00834–0·0652)	0·0693 (0·0237–0·172)	163 (-3·6–691)	0·0451 (0·0141–0·113)	0·112 (0·0377–0·282)	148 (-11·2–656)
Jordan	0·00267 (0·000809–0·00654)	0·00739 (0·00231–0·0186)	176 (-3·4–816)	0·0044 (0·00142–0·0113)	0·013 (0·00442–0·0331)	195 (3–850)	0·00708 (0·00221–0·018)	0·0204 (0·00671–0·0516)	188 (2·5–823)
Kuwait	0·00102 (0·000301–0·0025)	0·0032 (0·00104–0·00825)	213 (10·7–908)	0·00274 (0·000872–0·00663)	0·0113 (0·00386–0·0288)	314 (48·6–1140)	0·00377 (0·00117–0·00914)	0·0145 (0·00493–0·0372)	286 (37·5–1080)
Lebanon	0·00484 (0·00147–0·0121)	0·00854 (0·00285–0·022)	76·4 (-37·2–477)	0·00703 (0·00223–0·0175)	0·0143 (0·00482–0·036)	103 (-25·7–500)	0·0119 (0·00367–0·0297)	0·0228 (0·0077–0·0584)	92·2 (-30·1–495)
Libya	0·00364 (0·00107–0·00914)	0·00927 (0·00303–0·024)	155 (-12·2–727)	0·00843 (0·00271–0·0219)	0·0251 (0·00885–0·0625)	198 (9·1–844)	0·0121 (0·00378–0·031)	0·0344 (0·0118–0·0867)	185 (2·4–811)
Morocco	0·0208 (0·00607–0·0532)	0·0327 (0·0104–0·0835)	56·8 (-44·9–416)	0·0438 (0·014–0·11)	0·072 (0·0253–0·172)	64·5 (-36·2–402)	0·0646 (0·0204–0·163)	0·105 (0·0355–0·258)	62 (-40·2–404)
Oman	0·00182 (0·000548–0·00469)	0·00535 (0·00173–0·0137)	194 (2·4–823)	0·00417 (0·00133–0·0102)	0·0126 (0·00415–0·0313)	202 (12·1–797)	0·00599 (0·00188–0·0149)	0·0179 (0·00591–0·0454)	199 (8·5–790)

	Female			Male			Both		
	Number of YLDs (thousands)		Change (%)	Number of YLDs (thousands)		Change (%)	Number of YLDs (thousands)		Change (%)
	1990	2023		1990	2023		1990	2023	
Palestine	0·00218 (0·000653–0·00534)	0·00515 (0·00176–0·0137)	137 (-16·2–642)	0·00478 (0·00149–0·0116)	0·0135 (0·00463–0·0341)	183 (2·7–723)	0·00696 (0·00216–0·0173)	0·0187 (0·0064–0·0476)	169 (-3·2–699)
Qatar	0·000194 (5·52e-05–0·00049)	0·00139 (0·000465–0·00352)	617 (159–2260)	0·00136 (0·000424–0·00311)	0·00901 (0·00308–0·0225)	561 (146–1830)	0·00156 (0·000482–0·00358)	0·0104 (0·00354–0·026)	568 (147–1870)
Saudi Arabia	0·0111 (0·00339–0·0273)	0·0308 (0·00989–0·0792)	178 (-5·5–786)	0·0211 (0·0067–0·0494)	0·0606 (0·0199–0·152)	188 (1·3–770)	0·0321 (0·01–0·0767)	0·0914 (0·03–0·232)	185 (0·7–781)
Sudan	0·0148 (0·00444–0·0381)	0·0292 (0·00932–0·0769)	96·9 (-29·1–517)	0·178 (0·0567–0·431)	0·469 (0·182–1·11)	164 (1·7–619)	0·193 (0·0615–0·468)	0·498 (0·192–1·18)	159 (0–599)
Syrian Arab Republic	0·00781 (0·00227–0·0194)	0·0156 (0·00501–0·0397)	99·8 (-29–549)	0·00806 (0·00247–0·0202)	0·0193 (0·00684–0·0494)	139 (-14·9–664)	0·0159 (0·00474–0·0398)	0·0349 (0·0118–0·0883)	120 (-22·9–592)
Tunisia	0·0321 (0·00995–0·0782)	0·0486 (0·0157–0·122)	51·8 (-45·4–381)	0·0623 (0·0208–0·142)	0·0953 (0·0332–0·221)	53 (-43–348)	0·0943 (0·031–0·219)	0·144 (0·0489–0·342)	52·6 (-44·4–356)
Türkiye	0·101 (0·0296–0·253)	0·182 (0·058–0·461)	81·1 (-37·2–482)	0·107 (0·0343–0·25)	0·169 (0·0572–0·422)	57·5 (-42·5–386)	0·208 (0·0642–0·503)	0·351 (0·116–0·882)	68·9 (-39·4–434)
United Arab Emirates	0·000736 (0·000218–0·00192)	0·00348 (0·00113–0·00887)	373 (61·7–1450)	0·00275 (0·000859–0·00669)	0·0168 (0·00528–0·0424)	511 (118–1880)	0·00349 (0·00108–0·00859)	0·0203 (0·00647–0·0516)	481 (106–1820)
Yemen	0·219 (0·0847–0·457)	0·382 (0·187–0·716)	74·3 (-8·5–264)	0·491 (0·213–0·929)	0·859 (0·454–1·42)	75 (2·5–216)	0·71 (0·295–1·38)	1·24 (0·627–2·07)	74·8 (1·3–224)
South Asia	31·1 (13·4–60·9)	81·2 (43·8–146)	161 (46·6–383)	63·2 (30·3–113)	145 (85·3–236)	129 (45·3–279)	94·3 (43·4–171)	226 (131–380)	140 (46–309)
Bangladesh	5·95 (2·8–10·5)	18·4 (10·9–29·4)	209 (106–375)	5·19 (2·36–9·41)	14·6 (9·14–21)	182 (85·3–381)	11·1 (5·18–20)	33 (20·6–50)	196 (99·5–375)
Bhutan	0·0119 (0·00429–0·0251)	0·027 (0·0128–0·0529)	128 (16·8–400)	0·0234 (0·00956–0·0454)	0·0494 (0·0255–0·0859)	111 (20·4–291)	0·0352 (0·0138–0·0703)	0·0764 (0·0389–0·136)	117 (19·8–324)
India	23·3 (9·78–46·4)	57·4 (29·4–106)	146 (29·5–384)	53·3 (26–95·9)	118 (68·3–193)	122 (37·6–267)	76·6 (35·3–139)	175 (99·2–298)	129 (34·4–301)
Nepal	0·339 (0·122–0·719)	0·915 (0·412–1·86)	170 (38·2–480)	1·17 (0·566–2·05)	3·16 (1·74–5·12)	170 (81·9–314)	1·51 (0·685–2·76)	4·07 (2·19–6·85)	170 (72·2–337)
Pakistan	1·51 (0·552–3·46)	4·48 (1·69–9·83)	196 (22·8–647)	3·52 (1·36–7·05)	8·82 (4·2–17·1)	150 (16·7–466)	5·04 (1·92–10·4)	13·3 (6·2–27)	164 (18·7–518)
**Southeast Asia, East Asia, and Oceania**	11·9 (4·04–27·2)	23·1 (9·17–55·6)	94·1 (-18·6–416)	9·68 (3·46–22·3)	25·7 (9·94–60·3)	166 (5·9–599)	21·6 (7·53–49·5)	48·8 (19·8–117)	126 (-7·5–500)

	Female			Male			Both		
	Number of YLDs (thousands)		Change (%)	Number of YLDs (thousands)		Change (%)	Number of YLDs (thousands)		Change (%)
	1990	2023		1990	2023		1990	2023	
East Asia	2·07 (0·628–5·36)	5·51 (1·8–14·6)	166 (-8·8–759)	4·17 (1·39–10·3)	12·7 (4·35–32·1)	205 (7·7–857)	6·23 (2·04–15·7)	18·2 (6·14–46·7)	192 (2·3–824)
China	2·01 (0·61–5·21)	5·39 (1·76–14·3)	169 (-8·1–766)	4·07 (1·36–10·1)	12·5 (4·27–31·6)	206 (8·3–862)	6·08 (1·99–15·3)	17·9 (6·03–45·9)	194 (2·9–830)
Democratic People's Republic of Korea	0·0225 (0·00689–0·0562)	0·042 (0·0136–0·111)	86·5 (-33–498)	0·0202 (0·00648–0·0505)	0·0421 (0·0139–0·107)	109 (-26·5–557)	0·0427 (0·0135–0·106)	0·0841 (0·0276–0·218)	96·9 (-30·2–530)
Taiwan	0·0363 (0·0111–0·0928)	0·0701 (0·0228–0·181)	92·8 (-30·5–520)	0·0727 (0·0228–0·177)	0·174 (0·0586–0·414)	140 (-13·5–623)	0·109 (0·0344–0·27)	0·244 (0·0816–0·586)	124 (-18·3–588)
Oceania	0·094 (0·0331–0·208)	0·301 (0·132–0·604)	220 (43·2–693)	0·0687 (0·0231–0·152)	0·167 (0·0628–0·389)	143 (-1·9–531)	0·163 (0·056–0·359)	0·469 (0·199–1)	188 (25·1–597)
American Samoa	0·00027 (8·28e-05–0·000665)	0·000381 (0·000125–0·000988)	40·8 (-48·3–345)	0·000488 (0·000164–0·00117)	0·000641 (0·000233–0·00156)	31·4 (-50–276)	0·000758 (0·000242–0·00184)	0·00102 (0·00036–0·00252)	34·7 (-49·8–299)
Cook Islands	0·000144 (4·44e-05–0·000361)	0·000159 (5·13e-05–0·000418)	9·8 (-60·6–246)	0·000213 (6·92e-05–0·000518)	0·000201 (7·08e-05–0·000492)	-5·7 (-63·8–165)	0·000357 (0·000114–0·000879)	0·000359 (0·000125–0·000913)	0·6 (-62·2–198)
Fiji	0·00458 (0·00141–0·0116)	0·0071 (0·00236–0·0185)	55·1 (-43·7–388)	0·00601 (0·00196–0·015)	0·00884 (0·00319–0·0218)	47·1 (-44·9–332)	0·0106 (0·00336–0·0263)	0·0159 (0·00559–0·0403)	50·6 (-44·3–355)
Guam	0·000923 (0·000285–0·00232)	0·00208 (0·000832–0·00489)	126 (-10·9–530)	0·00258 (0·000938–0·00562)	0·00488 (0·00235–0·00964)	89·2 (-8·7–306)	0·0035 (0·00123–0·00792)	0·00696 (0·00315–0·0143)	98·9 (-7·1–367)
Kiribati	0·000694 (0·000215–0·00171)	0·00178 (0·000689–0·00394)	156 (6·4–581)	0·00209 (0·000756–0·00458)	0·00443 (0·00193–0·00891)	112 (-1·8–404)	0·00278 (0·00097–0·00624)	0·0062 (0·00265–0·013)	123 (-0·4–453)
Marshall Islands	0·000362 (0·000118–0·000856)	0·000739 (0·000312–0·00161)	104 (-9·9–412)	0·000752 (0·000294–0·00153)	0·00112 (0·000513–0·00233)	48·9 (-28·7–211)	0·00111 (0·000419–0·00238)	0·00186 (0·000813–0·00392)	66·8 (-21·4–270)
Micronesia (Federated States of)	0·00103 (0·000341–0·0024)	0·00116 (0·000398–0·00285)	12·3 (-56·9–215)	0·00177 (0·000627–0·00388)	0·00213 (0·000792–0·00496)	20·2 (-51·9–201)	0·0028 (0·000969–0·0063)	0·00329 (0·0012–0·0078)	17·3 (-53·6–204)
Nauru	2·15e-05 (6·61e-06–5·61e-05)	3·33e-05 (1·07e-05–8·69e-05)	54·6 (-43–410)	2·29e-05 (7·43e-06–5·6e-05)	3·09e-05 (1·07e-05–7·81e-05)	35·4 (-50·1–324)	4·44e-05 (1·42e-05–0·000112)	6·42e-05 (2·2e-05–0·000164)	44·7 (-47·1–369)
Niue	1·33e-05 (3·99e-06–3·4e-05)	1·06e-05 (3·49e-06–2·73e-05)	-20·7 (-71·1–166)	1·25e-05 (4·07e-06–3·08e-05)	1·07e-05 (3·66e-06–2·68e-05)	-14 (-68·3–149)	2·58e-05 (8·03e-06–6·44e-05)	2·13e-05 (7·15e-06–5·43e-05)	-17·4 (-69·7–157)
Northern Mariana Islands	0·000432 (0·000133–0·00104)	0·000605 (0·000218–0·00147)	40·3 (-47·2–303)	0·000814 (0·000284–0·00183)	0·00106 (0·000386–0·00241)	30 (-45·7–234)	0·00125 (0·000414–0·00285)	0·00166 (0·000604–0·0039)	33·6 (-46·4–254)

	Female			Male			Both		
	Number of YLDs (thousands)		Change (%)	Number of YLDs (thousands)		Change (%)	Number of YLDs (thousands)		Change (%)
	1990	2023		1990	2023		1990	2023	
Palau	0·00125 (0·000476–0·00256)	0·00269 (0·00128–0·00515)	115 (6·1–374)	0·00144 (0·000559–0·00284)	0·00299 (0·00147–0·00585)	107 (5·7–335)	0·00269 (0·00103–0·00541)	0·00567 (0·00282–0·0109)	111 (6·4–352)
Papua New Guinea	0·0744 (0·0268–0·16)	0·261 (0·116–0·517)	251 (58·3–765)	0·0415 (0·014–0·0912)	0·116 (0·0413–0·27)	179 (10·8–619)	0·116 (0·0404–0·254)	0·376 (0·162–0·788)	225 (41·6–683)
Samoa	0·000597 (0·000181–0·00152)	0·000962 (0·000313–0·00251)	61·2 (-45·2–412)	0·000794 (0·000256–0·00197)	0·0013 (0·000438–0·00331)	63·3 (-38·8–383)	0·00139 (0·000439–0·00349)	0·00226 (0·000758–0·00588)	62·4 (-42·1–393)
Solomon Islands	0·00227 (0·000709–0·00546)	0·00735 (0·00251–0·0183)	223 (19·8–885)	0·00358 (0·00115–0·00854)	0·0113 (0·00412–0·027)	215 (21·7–768)	0·00585 (0·00188–0·014)	0·0186 (0·00663–0·0463)	218 (21·4–811)
Tokelau	1·17e-05 (3·5e-06–2·95e-05)	1·36e-05 (4·42e-06–3·44e-05)	16 (-58·8–274)	1·37e-05 (4·44e-06–3·32e-05)	1·7e-05 (5·98e-06–4·14e-05)	24·2 (-52·8–260)	2·54e-05 (8·01e-06–6·27e-05)	3·05e-05 (1·05e-05–7·81e-05)	20·4 (-55·7–263)
Tonga	0·000205 (6·15e-05–0·000521)	0·000227 (7·33e-05–0·000582)	10·9 (-61·7–254)	0·00026 (8·26e-05–0·000673)	0·000285 (9·71e-05–0·000718)	9·6 (-59·5–234)	0·000465 (0·000145–0·00119)	0·000512 (0·000167–0·00131)	10·1 (-61·3–243)
Tuvalu	3·1e-05 (9·36e-06–7·95e-05)	3·86e-05 (1·24e-05–0·000103)	24·6 (-54·9–303)	4·03e-05 (1·3e-05–9·87e-05)	6·01e-05 (2·04e-05–0·000153)	49·1 (-45·8–351)	7·13e-05 (2·24e-05–0·000178)	9·87e-05 (3·27e-05–0·000257)	38·4 (-49·9–325)
Vanuatu	0·000985 (0·000304–0·00245)	0·00279 (0·000905–0·00712)	183 (7–825)	0·00209 (0·000698–0·00494)	0·0054 (0·00194–0·0132)	159 (1·9–651)	0·00308 (0·000996–0·00738)	0·00819 (0·00284–0·0205)	166 (3·5–703)
Southeast Asia	9·74 (3·38–21·8)	17·3 (7·02–40·2)	77·5 (-21·5–348)	5·45 (1·99–12)	12·9 (5·45–28·1)	136 (1·7–455)	15·2 (5·42–33·8)	30·2 (12·5–68·4)	98·7 (-14·2–389)
Cambodia	0·636 (0·277–1·18)	1·45 (0·687–2·6)	128 (22–391)	0·0652 (0·0221–0·154)	0·153 (0·0545–0·359)	134 (-12·2–561)	0·701 (0·304–1·32)	1·6 (0·825–2·77)	128 (33·6–377)
Indonesia	3·65 (1·26–8·51)	4·22 (1·46–10·4)	15·4 (-55·3–231)	0·464 (0·152–1·19)	1·22 (0·419–3·16)	163 (-3·5–686)	4·12 (1·42–9·69)	5·44 (1·87–13·7)	32·1 (-48·9–283)
Lao People's Democratic Republic	0·131 (0·0454–0·294)	0·273 (0·121–0·618)	108 (-13·3–422)	0·0122 (0·00384–0·0294)	0·0234 (0·00789–0·0579)	92·4 (-28·4–473)	0·143 (0·0495–0·324)	0·296 (0·128–0·676)	107 (-12·9–418)
Malaysia	0·141 (0·0436–0·338)	0·453 (0·147–1·15)	222 (14–912)	0·153 (0·0498–0·37)	0·725 (0·275–1·74)	374 (81·1–1210)	0·294 (0·0939–0·702)	1·18 (0·432–2·87)	301 (47·1–1080)
Maldives	0·00347 (0·00115–0·00806)	0·00879 (0·00311–0·0209)	153 (-2·1–565)	0·00677 (0·00248–0·0148)	0·0186 (0·00773–0·0398)	175 (17·4–599)	0·0102 (0·00362–0·0229)	0·0274 (0·011–0·0595)	167 (14·2–618)
Mauritius	0·0255 (0·00825–0·058)	0·0456 (0·0153–0·114)	79·1 (-36·6–435)	0·00864 (0·00285–0·0213)	0·0181 (0·00629–0·0445)	109 (-20·4–519)	0·0341 (0·0111–0·0788)	0·0637 (0·0217–0·159)	86·8 (-32·8–457)
Myanmar	1·88 (0·649–4·18)	3·08 (1·17–7)	63·9 (-28·9–313)	2·46 (0·922–5·07)	5·21 (2·27–10·3)	112 (-4·6–378)	4·34 (1·56–9·18)	8·29 (3·48–17·2)	91·1 (-14–357)

	Female			Male			Both		
	Number of YLDs (thousands)		Change (%)	Number of YLDs (thousands)		Change (%)	Number of YLDs (thousands)		Change (%)
	1990	2023		1990	2023		1990	2023	
Philippines	0·379 (0·115–0·952)	0·96 (0·325–2·43)	153 (-8·9–676)	0·589 (0·193–1·44)	1·58 (0·608–3·78)	168 (3·3–606)	0·968 (0·305–2·41)	2·54 (0·94–6·16)	162 (-0·7–632)
Seychelles	0·00131 (0·000423–0·00314)	0·00206 (0·000705–0·00516)	57·1 (-41·2–362)	0·000687 (0·000218–0·00167)	0·00152 (0·000518–0·00363)	122 (-23·5–593)	0·002 (0·000646–0·00482)	0·00358 (0·00122–0·00877)	79·2 (-34·6–444)
Sri Lanka	0·493 (0·166–1·15)	1·2 (0·419–2·83)	143 (-6–588)	1·05 (0·413–2·22)	2·18 (0·946–4·27)	107 (-5·2–358)	1·55 (0·58–3·36)	3·38 (1·38–7·16)	119 (-3·6–417)
Thailand	1·38 (0·471–3·2)	3·7 (1·32–8·57)	167 (1·5–637)	0·432 (0·142–1·07)	1·3 (0·457–3·18)	200 (8–768)	1·82 (0·612–4·23)	5 (1·76–11·7)	175 (3–667)
Timor-Leste	0·00647 (0·0021–0·0158)	0·0168 (0·00571–0·0423)	160 (-1·6–664)	0·00314 (0·000994–0·00786)	0·0105 (0·00364–0·0263)	235 (24–909)	0·00961 (0·00311–0·0237)	0·0273 (0·00929–0·0686)	184 (6·9–744)
Viet Nam	0·991 (0·323–2·42)	1·86 (0·616–4·42)	87·7 (-28·6–462)	0·195 (0·0619–0·47)	0·429 (0·143–1·08)	120 (-21·6–566)	1·19 (0·39–2·89)	2·29 (0·756–5·5)	93·1 (-28·3–478)
**Sub-Saharan Africa**	1·37 (0·444–3·36)	3·17 (1·2–7·83)	131 (-10·1–568)	1·62 (0·583–3·65)	3·7 (1·55–8·19)	129 (-5·9–463)	2·99 (1·03–7·01)	6·87 (2·77–16)	130 (-7·8–497)
Central Sub-Saharan Africa	0·119 (0·0379–0·293)	0·212 (0·0695–0·551)	79 (-36·8–441)	0·0957 (0·0306–0·23)	0·276 (0·0981–0·672)	189 (4·4–759)	0·214 (0·0681–0·523)	0·489 (0·166–1·22)	128 (-17·1–585)
Angola	0·0069 (0·00212–0·018)	0·0254 (0·0081–0·0645)	268 (32·4–1070)	0·00937 (0·00298–0·0226)	0·0269 (0·00908–0·0679)	187 (3·8–769)	0·0163 (0·00512–0·0405)	0·0523 (0·0171–0·132)	221 (13·3–920)
Central African Republic	0·00597 (0·00186–0·0148)	0·0132 (0·00441–0·0345)	122 (-21·6–585)	0·00395 (0·00128–0·00967)	0·00946 (0·00322–0·0233)	139 (-10·9–615)	0·00992 (0·00314–0·0246)	0·0227 (0·00765–0·0585)	129 (-17·7–598)
Congo	0·00593 (0·00185–0·0155)	0·0126 (0·00403–0·0326)	112 (-25·1–607)	0·00267 (0·000856–0·0065)	0·00703 (0·00248–0·0176)	163 (0–725)	0·0086 (0·00272–0·022)	0·0196 (0·00654–0·0506)	128 (-19·2–644)
Democratic Republic of the Congo	0·098 (0·0315–0·241)	0·158 (0·0519–0·406)	60·8 (-43·7–378)	0·0786 (0·0251–0·188)	0·231 (0·0822–0·56)	193 (6·7–767)	0·177 (0·0567–0·43)	0·388 (0·133–0·968)	120 (-19·6–552)
Equatorial Guinea	0·000751 (0·000242–0·00189)	0·00166 (0·000536–0·00431)	121 (-22·9–604)	0·000516 (0·000169–0·00127)	0·0012 (0·000411–0·00291)	133 (-12·8–584)	0·00127 (0·00041–0·00315)	0·00286 (0·000944–0·00726)	126 (-18·5–612)
Gabon	0·00109 (0·000336–0·00272)	0·00197 (0·000623–0·00505)	79·9 (-36·7–472)	0·000523 (0·000167–0·0013)	0·0012 (0·000398–0·00306)	129 (-16–601)	0·00162 (0·000504–0·00401)	0·00317 (0·00102–0·008)	95·7 (-29·8–520)
Eastern Sub-Saharan Africa	0·559 (0·18–1·35)	1·36 (0·561–3·22)	143 (1·2–536)	1·02 (0·384–2·22)	2·35 (1·05–4·91)	131 (0·4–439)	1·58 (0·564–3·55)	3·71 (1·6–8·04)	135 (0·7–467)
Burundi	0·0255 (0·00786–0·062)	0·0447 (0·0151–0·105)	75 (-37·3–406)	0·0501 (0·0174–0·116)	0·0891 (0·0327–0·206)	77·8 (-31·6–367)	0·0756 (0·0253–0·178)	0·134 (0·0478–0·309)	76·8 (-34·2–381)

	Female			Male			Both		
	Number of YLDs (thousands)		Change (%)	Number of YLDs (thousands)		Change (%)	Number of YLDs (thousands)		Change (%)
	1990	2023		1990	2023		1990	2023	
Comoros	0·00513 (0·0017–0·012)	0·00899 (0·00305–0·0219)	75·4 (-33·1–396)	0·00815 (0·00308–0·018)	0·0164 (0·00633–0·0365)	101 (-15·7–376)	0·0133 (0·00483–0·0299)	0·0254 (0·00945–0·0578)	91·3 (-21·7–371)
Djibouti	0·000852 (0·000259–0·00211)	0·00353 (0·00118–0·00889)	314 (46·6–1230)	0·00123 (0·000409–0·00303)	0·00502 (0·00162–0·0117)	309 (56·6–1080)	0·00208 (0·000664–0·00507)	0·00855 (0·00281–0·0205)	311 (48–1130)
Eritrea	0·00224 (0·000684–0·00575)	0·00534 (0·00171–0·0138)	139 (-15·9–654)	0·0217 (0·00739–0·0521)	0·0459 (0·0159–0·106)	111 (-19·4–497)	0·0239 (0·00808–0·0581)	0·0512 (0·0176–0·12)	114 (-19·7–515)
Ethiopia	0·0381 (0·0119–0·0967)	0·0779 (0·0251–0·2)	105 (-27·5–577)	0·182 (0·0607–0·425)	0·368 (0·134–0·842)	102 (-24·2–427)	0·22 (0·0719–0·518)	0·446 (0·16–1·02)	103 (-25·6–445)
Kenya	0·0461 (0·0147–0·117)	0·128 (0·042–0·321)	178 (-3·3–794)	0·0556 (0·0184–0·14)	0·16 (0·0554–0·379)	187 (4·1–754)	0·102 (0·0333–0·257)	0·288 (0·0974–0·697)	183 (0·4–774)
Madagascar	0·18 (0·062–0·405)	0·584 (0·272–1·2)	224 (48·2–678)	0·499 (0·205–0·996)	1·24 (0·589–2·41)	148 (25–428)	0·679 (0·267–1·41)	1·82 (0·878–3·58)	168 (31–485)
Malawi	0·029 (0·00929–0·0695)	0·048 (0·0156–0·118)	65·7 (-39·4–410)	0·0144 (0·00459–0·0357)	0·025 (0·00853–0·0605)	73·9 (-39–464)	0·0434 (0·0139–0·105)	0·073 (0·0241–0·179)	68·4 (-38·8–423)
Mozambique	0·0553 (0·0175–0·136)	0·0759 (0·0245–0·199)	37·2 (-49·5–310)	0·0547 (0·0177–0·135)	0·0996 (0·0345–0·25)	82 (-34·7–441)	0·11 (0·0354–0·271)	0·175 (0·0592–0·449)	59·5 (-43·1–384)
Rwanda	0·0479 (0·015–0·117)	0·0755 (0·0258–0·185)	57·7 (-43·4–358)	0·00645 (0·00215–0·0165)	0·0108 (0·00358–0·0266)	66·7 (-39·7–410)	0·0543 (0·0171–0·134)	0·0862 (0·0294–0·213)	58·7 (-43·1–363)
Somalia	0·0141 (0·00435–0·0357)	0·0388 (0·0126–0·101)	176 (-2·5–821)	0·0183 (0·00605–0·0458)	0·0453 (0·0152–0·114)	148 (-10·1–658)	0·0323 (0·0105–0·0812)	0·0841 (0·0276–0·218)	160 (-8·5–733)
South Sudan	0·0109 (0·00343–0·0266)	0·021 (0·00696–0·0513)	92·4 (-34·1–512)	0·0196 (0·00639–0·0461)	0·0287 (0·00961–0·0693)	46·3 (-47·5–355)	0·0305 (0·00983–0·0726)	0·0497 (0·0166–0·119)	62·8 (-43·5–417)
Uganda	0·0509 (0·0163–0·128)	0·122 (0·041–0·308)	140 (-11·3–650)	0·0257 (0·00815–0·0631)	0·0597 (0·0209–0·143)	133 (-17·7–635)	0·0766 (0·0249–0·189)	0·182 (0·0612–0·452)	137 (-15·9–639)
United Republic of Tanzania	0·0395 (0·0123–0·0977)	0·0904 (0·0291–0·232)	129 (-19·1–615)	0·0498 (0·0166–0·123)	0·124 (0·0415–0·306)	148 (-5·9–667)	0·0893 (0·0288–0·218)	0·214 (0·07–0·533)	140 (-11·8–645)
Zambia	0·0126 (0·00397–0·0325)	0·0339 (0·0114–0·0859)	168 (-4–762)	0·0096 (0·00311–0·0238)	0·0284 (0·00956–0·0685)	196 (6·4–820)	0·0222 (0·00715–0·0564)	0·0623 (0·0211–0·154)	180 (1–787)
Southern Sub-Saharan Africa	0·247 (0·0805–0·603)	0·527 (0·183–1·32)	113 (-20·8–539)	0·0834 (0·0283–0·204)	0·126 (0·0429–0·308)	50·8 (-46·2–358)	0·331 (0·109–0·823)	0·652 (0·226–1·63)	97·3 (-27·2–497)
Botswana	0·0183 (0·00637–0·0411)	0·0388 (0·0162–0·0861)	112 (-11·2–414)	0·00503 (0·00169–0·0123)	0·00954 (0·00321–0·0233)	89·5 (-28·2–427)	0·0234 (0·00806–0·052)	0·0483 (0·0199–0·109)	107 (-14·3–403)

	Female			Male			Both		
	Number of YLDs (thousands)		Change (%)	Number of YLDs (thousands)		Change (%)	Number of YLDs (thousands)		Change (%)
	1990	2023		1990	2023		1990	2023	
Eswatini	0·000947 (0·000298–0·00238)	0·00258 (0·000868–0·00668)	172 (-0·2–766)	0·00122 (0·000395–0·00307)	0·00238 (0·000816–0·00571)	94·5 (-27·5–528)	0·00217 (0·000703–0·00553)	0·00496 (0·00168–0·0123)	128 (-16·7–627)
Lesotho	0·00364 (0·00115–0·00946)	0·00658 (0·00215–0·0168)	80·8 (-33·9–450)	0·00244 (0·000816–0·00613)	0·00378 (0·00131–0·00913)	54·6 (-44·3–371)	0·00608 (0·00199–0·0158)	0·0104 (0·00347–0·0257)	70·3 (-39·1–423)
Namibia	0·00482 (0·00154–0·0121)	0·0132 (0·00427–0·0337)	174 (-2·7–721)	0·00255 (0·000807–0·00633)	0·00509 (0·00173–0·0125)	100 (-29·1–526)	0·00737 (0·00243–0·0184)	0·0183 (0·00603–0·0462)	148 (-11·2–662)
South Africa	0·199 (0·0635–0·489)	0·388 (0·133–0·96)	95·4 (-29·1–495)	0·0645 (0·0218–0·158)	0·0898 (0·0307–0·219)	39·4 (-50·1–323)	0·263 (0·0863–0·66)	0·478 (0·166–1·2)	81·7 (-34·3–456)
Zimbabwe	0·0209 (0·00666–0·051)	0·0775 (0·0249–0·192)	271 (37·8–1040)	0·00771 (0·00249–0·0188)	0·0152 (0·00514–0·0381)	96·7 (-31·9–502)	0·0286 (0·00913–0·0696)	0·0927 (0·0299–0·231)	224 (17·4–897)
Western Sub-Saharan Africa	0·445 (0·139–1·12)	1·07 (0·352–2·72)	140 (-13·9–652)	0·42 (0·138–1·02)	0·955 (0·331–2·3)	127 (-15·2–550)	0·865 (0·277–2·13)	2·02 (0·683–5·02)	134 (-14·5–605)
Benin	0·0164 (0·00524–0·0413)	0·0485 (0·0164–0·123)	195 (6·4–764)	0·00987 (0·00322–0·0241)	0·0313 (0·0112–0·0738)	217 (22–769)	0·0263 (0·00853–0·0654)	0·0798 (0·0275–0·197)	203 (10–761)
Burkina Faso	0·0476 (0·0151–0·115)	0·101 (0·0344–0·253)	112 (-20·3–514)	0·0375 (0·0126–0·0894)	0·0701 (0·0236–0·166)	86·8 (-28–418)	0·0851 (0·0273–0·204)	0·171 (0·0579–0·419)	101 (-24·5–460)
Cabo Verde	0·00108 (0·000327–0·00275)	0·00133 (0·000434–0·00342)	22·7 (-57·2–288)	0·000554 (0·000176–0·00138)	0·000794 (0·000261–0·00195)	43·3 (-48·5–342)	0·00164 (0·000505–0·00412)	0·00212 (0·000694–0·00537)	29·7 (-54·5–305)
Cameroon	0·0512 (0·0165–0·121)	0·16 (0·055–0·383)	212 (16·3–764)	0·0183 (0·00585–0·0462)	0·064 (0·0217–0·157)	249 (30·6–911)	0·0695 (0·0229–0·168)	0·224 (0·0768–0·546)	222 (20·6–803)
Chad	0·024 (0·00759–0·0595)	0·0513 (0·0172–0·13)	114 (-21·9–550)	0·0235 (0·00787–0·0574)	0·054 (0·019–0·131)	130 (-13·9–533)	0·0475 (0·0157–0·115)	0·105 (0·0363–0·261)	122 (-17·8–537)
Côte d'Ivoire	0·0145 (0·00454–0·0374)	0·0421 (0·0138–0·11)	189 (6·1–824)	0·0128 (0·00419–0·0314)	0·0369 (0·0123–0·089)	188 (4·1–795)	0·0273 (0·00876–0·069)	0·0789 (0·0261–0·199)	189 (4·9–811)
Gambia	0·00229 (0·000705–0·00575)	0·00663 (0·00213–0·0174)	190 (4·5–817)	0·00188 (0·000595–0·00461)	0·00448 (0·00152–0·0112)	138 (-14–614)	0·00416 (0·00129–0·0104)	0·0111 (0·00364–0·0286)	167 (-1·1–724)
Ghana	0·027 (0·00825–0·0696)	0·0753 (0·0242–0·198)	179 (-2·9–813)	0·0418 (0·0137–0·102)	0·108 (0·0374–0·267)	158 (-4·6–684)	0·0688 (0·0219–0·171)	0·183 (0·0618–0·462)	166 (-4·3–734)
Guinea	0·0132 (0·00416–0·0334)	0·0221 (0·00699–0·0566)	67·5 (-39·5–441)	0·0146 (0·00474–0·0349)	0·0314 (0·0105–0·0792)	115 (-21·7–524)	0·0278 (0·00891–0·0684)	0·0535 (0·0174–0·136)	92·4 (-30·5–486)
Guinea-Bissau	0·00213 (0·000663–0·00544)	0·00377 (0·00123–0·00976)	77·3 (-37·2–459)	0·00174 (0·000558–0·00437)	0·00263 (0·000898–0·00675)	51·2 (-41·8–371)	0·00386 (0·00123–0·00981)	0·0064 (0·00213–0·0165)	65·6 (-39·3–419)

	Female			Male			Both		
	Number of YLDs (thousands)		Change (%)	Number of YLDs (thousands)		Change (%)	Number of YLDs (thousands)		Change (%)
	1990	2023		1990	2023		1990	2023	
Liberia	0·00509 (0·00159–0·0128)	0·0105 (0·00344–0·0271)	107 (-27–546)	0·00753 (0·00244–0·0186)	0·0182 (0·00627–0·0448)	142 (-8·6–634)	0·0126 (0·00403–0·0314)	0·0288 (0·00971–0·0719)	128 (-16·3–599)
Mali	0·0182 (0·00576–0·0465)	0·0371 (0·0117–0·0954)	104 (-24·1–568)	0·0407 (0·0141–0·0965)	0·1 (0·0363–0·23)	146 (-0·4–548)	0·0589 (0·0199–0·142)	0·137 (0·0481–0·328)	133 (-8·3–547)
Mauritania	0·00797 (0·00246–0·0201)	0·015 (0·00496–0·0398)	88·8 (-34·4–498)	0·00622 (0·00198–0·0145)	0·012 (0·00409–0·0298)	92·9 (-29·4–486)	0·0142 (0·0045–0·0345)	0·027 (0·00902–0·0702)	90·6 (-32·1–494)
Niger	0·0212 (0·00657–0·0532)	0·0715 (0·0237–0·18)	238 (19·1–959)	0·0492 (0·0168–0·111)	0·137 (0·0506–0·304)	179 (7·4–628)	0·0704 (0·0234–0·164)	0·209 (0·0744–0·486)	196 (11·1–703)
Nigeria	0·132 (0·0411–0·341)	0·273 (0·0885–0·713)	107 (-27·4–555)	0·117 (0·038–0·299)	0·214 (0·0732–0·537)	82·1 (-35·6–444)	0·249 (0·08–0·642)	0·487 (0·161–1·25)	95·5 (-31·3–501)
Sao Tome and Principe	0·00023 (7·11e-05–0·000583)	0·000397 (0·000127–0·00103)	72·7 (-39·8–440)	0·000263 (8·33e-05–0·000655)	0·00062 (0·000209–0·00157)	136 (-13·6–623)	0·000493 (0·000153–0·00126)	0·00102 (0·000338–0·0026)	106 (-25·3–540)
Senegal	0·025 (0·00763–0·0619)	0·0722 (0·0231–0·188)	189 (3·3–810)	0·00934 (0·00299–0·0225)	0·0196 (0·00651–0·0493)	110 (-23·8–542)	0·0343 (0·0106–0·0844)	0·0918 (0·0297–0·238)	168 (-3·9–729)
Sierra Leone	0·0224 (0·00711–0·0564)	0·0352 (0·0114–0·0885)	57·1 (-42·9–386)	0·0182 (0·00595–0·0442)	0·0268 (0·0093–0·0653)	46·8 (-43·9–333)	0·0406 (0·013–0·101)	0·0619 (0·0207–0·153)	52·5 (-43·8–362)
Togo	0·0136 (0·00429–0·0342)	0·0429 (0·014–0·108)	214 (11·3–857)	0·00878 (0·00283–0·0209)	0·0228 (0·00781–0·0574)	160 (-1·5–697)	0·0224 (0·00716–0·0551)	0·0657 (0·0218–0·166)	193 (5·2–788)

Return to Table of Contents

Appendix Table 2·11: Age-standardized YLD rate attributable to chewing tobacco use in 1990 and 2023, among ages 15+

	Female			Male			Both		
	Age-standardized YLD rate (per 100,000)		Change (%)	Age-standardized YLD rate (per 100,000)		Change (%)	Age-standardized YLD rate (per 100,000)		Change (%)
	1990	2023		1990	2023		1990	2023	
Global	3 (1·2–6·1)	3·3 (1·6–6·6)	9·6 (-44·3–119·3)	6·2 (3–11)	6·3 (3·5–11·2)	2 (-37·7–69)	4·6 (2–8·4)	4·8 (2·5–8·8)	4·5 (-39·7–87·9)
**Central Europe, Eastern Europe, and Central Asia**	0·3 (0·1–0·9)	0·3 (0·1–0·8)	-8·9 (-69·1–197·3)	0·7 (0·2–1·7)	0·8 (0·3–1·9)	8·7 (-60·2–219·1)	0·5 (0·2–1·2)	0·5 (0·2–1·3)	2·8 (-62·5–220)
Central Asia	0·5 (0·1–1·2)	0·4 (0·1–1)	-21·9 (-73·3–151·5)	2·2 (0·7–5)	1·8 (0·7–4·4)	-16·2 (-68–126·8)	1·3 (0·4–2·9)	1·1 (0·4–2·6)	-16·4 (-67·7–140·2)
Armenia	0·2 (0·1–0·6)	0·2 (0·1–0·5)	-28·5 (-75·1–141·2)	0·3 (0·1–0·7)	0·2 (0·1–0·6)	-18·6 (-71·7–152·4)	0·3 (0·1–0·6)	0·2 (0·1–0·5)	-23·8 (-73·3–144·5)
Azerbaijan	0·2 (0·1–0·6)	0·2 (0·1–0·5)	-18·3 (-71·6–157·4)	0·5 (0·2–1·1)	0·3 (0·1–0·8)	-33·1 (-75·5–100·8)	0·3 (0·1–0·8)	0·2 (0·1–0·6)	-27·5 (-73·3–131·1)
Georgia	0·4 (0·1–1)	0·3 (0·1–0·9)	-8·6 (-68·1–193·3)	0·3 (0·1–0·9)	0·3 (0·1–0·8)	-5·2 (-65·7–192·2)	0·4 (0·1–0·9)	0·3 (0·1–0·9)	-7·2 (-67·2–191·1)
Kazakhstan	0·3 (0·1–0·8)	0·2 (0·1–0·5)	-31 (-76·8–126·7)	0·7 (0·2–1·9)	0·5 (0·2–1·2)	-36 (-77·1–104·8)	0·5 (0·1–1·2)	0·3 (0·1–0·8)	-34·3 (-76·8–113·9)
Kyrgyzstan	0·9 (0·3–2·3)	0·4 (0·1–1·1)	-51·9 (-83·4–54·3)	10·4 (3·7–23·2)	6·2 (2·5–13·3)	-40·6 (-76·1–50·5)	5·5 (1·7–11·1)	3·2 (1·2–6·6)	-41 (-75·3–57·7)
Mongolia	0·3 (0·1–0·8)	0·2 (0·1–0·5)	-33·8 (-77·8–120·7)	0·7 (0·2–1·7)	0·4 (0·1–1)	-44·6 (-80·3–69·5)	0·5 (0·1–1·2)	0·3 (0·1–0·7)	-41·6 (-79·3–87·5)
Tajikistan	1·1 (0·3–2·8)	1 (0·3–2·5)	-8·3 (-68·2–188·7)	0·7 (0·2–1·8)	0·5 (0·2–1·4)	-26·6 (-74·2–118·6)	0·9 (0·3–2·3)	0·8 (0·3–2)	-16·2 (-71·2–155·7)
Turkmenistan	0·3 (0·1–0·8)	0·3 (0·1–0·7)	-14·2 (-70·6–185·4)	0·5 (0·2–1·2)	0·4 (0·1–1)	-17·3 (-71·1–157·4)	0·4 (0·1–1)	0·3 (0·1–0·9)	-15·3 (-70·3–176·9)
Uzbekistan	0·7 (0·2–1·7)	0·5 (0·1–1·2)	-28·7 (-75·8–125·1)	4·6 (1·6–10·9)	3·5 (1·2–8·7)	-23·3 (-71–119·5)	2·6 (0·8–5·9)	2 (0·7–4·8)	-23·5 (-71–126·8)
Central Europe	0·3 (0·1–0·7)	0·3 (0·1–0·8)	8·6 (-62·3–256)	0·6 (0·2–1·4)	0·7 (0·2–1·7)	17·4 (-58·1–258·7)	0·4 (0·1–1)	0·5 (0·2–1·2)	14·2 (-59·4–261·3)
Albania	0·3 (0·1–0·8)	0·3 (0·1–0·8)	0·5 (-63·2–232·7)	0·7 (0·2–1·8)	0·8 (0·3–2)	10·5 (-58·3–238·9)	0·5 (0·2–1·3)	0·5 (0·2–1·4)	5·3 (-61·3–223·2)
Bosnia and Herzegovina	0·4 (0·1–1)	0·4 (0·1–1)	-5·4 (-66·2–193·1)	0·7 (0·2–1·7)	0·7 (0·3–1·9)	5 (-61·3–218·7)	0·5 (0·2–1·3)	0·5 (0·2–1·4)	0·4 (-63·5–208·5)

	Female			Male			Both		
	Age-standardized YLD rate (per 100,000)		Change (%)	Age-standardized YLD rate (per 100,000)		Change (%)	Age-standardized YLD rate (per 100,000)		Change (%)
	1990	2023		1990	2023		1990	2023	
Bulgaria	0·6 (0·2–1·6)	0·8 (0·3–2·1)	24 (-57·5–312·8)	1·3 (0·4–3·1)	1·6 (0·6–4)	28·3 (-52·7–298·1)	1 (0·3–2·3)	1·2 (0·4–2·9)	26·3 (-55–298·5)
Croatia	0·2 (0·1–0·5)	0·2 (0·1–0·5)	-20 (-71·8–167)	0·4 (0·1–1)	0·4 (0·1–0·9)	-9 (-67·6–176·9)	0·3 (0·1–0·7)	0·3 (0·1–0·6)	-13·1 (-68·8–174·9)
Czechia	0·3 (0·1–0·8)	0·3 (0·1–0·9)	-2·9 (-65·2–221·7)	0·6 (0·2–1·5)	0·6 (0·2–1·6)	1·1 (-62·2–219·7)	0·5 (0·1–1·1)	0·5 (0·2–1·2)	0·2 (-62·8–231·7)
Hungary	0·3 (0·1–0·7)	0·2 (0·1–0·5)	-30·9 (-76·1–128·7)	0·5 (0·1–1·2)	0·3 (0·1–0·8)	-31·3 (-74·8–105·4)	0·4 (0·1–0·9)	0·3 (0·1–0·6)	-31·1 (-75·4–114·3)
Montenegro	0·3 (0·1–0·9)	0·3 (0·1–0·9)	-1·8 (-65·8–217)	0·6 (0·2–1·5)	0·7 (0·2–1·6)	7 (-62·3–239·5)	0·5 (0·1–1·2)	0·5 (0·2–1·2)	3·6 (-63·7–234·6)
North Macedonia	0·4 (0·1–1)	0·4 (0·1–1·1)	-3·2 (-66·3–220·9)	0·7 (0·2–1·6)	0·8 (0·3–2·1)	22 (-57·6–281·9)	0·5 (0·2–1·3)	0·6 (0·2–1·6)	12·2 (-61·6–254·3)
Poland	0·2 (0·1–0·6)	0·4 (0·1–0·9)	63·7 (-42–442·5)	0·4 (0·1–0·9)	0·7 (0·2–1·6)	83·7 (-35·5–474·9)	0·3 (0·1–0·7)	0·5 (0·2–1·2)	75·7 (-38–464·5)
Romania	0·2 (0·1–0·6)	0·2 (0·1–0·5)	-24·5 (-72·9–154·3)	0·6 (0·2–1·4)	0·6 (0·2–1·5)	7·9 (-60·8–231·1)	0·4 (0·1–1)	0·4 (0·1–0·9)	-2·7 (-65–201)
Serbia	0·3 (0·1–0·9)	0·3 (0·1–0·8)	-10·7 (-69–196·8)	0·6 (0·2–1·5)	0·6 (0·2–1·5)	0·2 (-64·7–209·3)	0·5 (0·1–1·2)	0·5 (0·2–1·1)	-3·3 (-66·4–208·3)
Slovakia	0·2 (0·1–0·6)	0·2 (0·1–0·4)	-30·7 (-75·2–113·4)	0·5 (0·2–1·1)	0·3 (0·1–0·8)	-33·2 (-76·4–112·4)	0·4 (0·1–0·8)	0·2 (0·1–0·6)	-32·2 (-75·4–117·4)
Slovenia	0·2 (0·1–0·5)	0·2 (0·1–0·6)	28·4 (-54·1–319·4)	0·4 (0·1–1·1)	0·6 (0·2–1·4)	40·6 (-50·5–343·8)	0·3 (0·1–0·7)	0·4 (0·1–1)	39·2 (-48·3–357·3)
Eastern Europe	0·3 (0·1–0·9)	0·3 (0·1–0·7)	-17·8 (-72·5–167·3)	0·5 (0·2–1·2)	0·4 (0·1–1)	-14·8 (-69·8–166)	0·4 (0·1–1)	0·3 (0·1–0·8)	-16·1 (-70·6–173·2)
Belarus	0·3 (0·1–0·7)	0·2 (0·1–0·5)	-23 (-73·5–148·4)	0·5 (0·2–1·2)	0·5 (0·2–1·1)	-0·1 (-64·5–220·6)	0·4 (0·1–0·9)	0·3 (0·1–0·8)	-9·6 (-67·8–193·8)
Estonia	0·3 (0·1–0·6)	0·3 (0·1–0·8)	28·8 (-55·5–310·6)	0·4 (0·1–1)	0·8 (0·3–1·8)	96·7 (-26·4–491·8)	0·3 (0·1–0·8)	0·5 (0·2–1·3)	68·3 (-37·1–441·5)
Latvia	0·3 (0·1–0·7)	0·2 (0·1–0·5)	-25·9 (-74·3–141·4)	0·4 (0·1–0·9)	0·3 (0·1–0·7)	-18·6 (-70·4–146·2)	0·3 (0·1–0·8)	0·2 (0·1–0·6)	-22 (-71·8–146·9)
Lithuania	0·2 (0·1–0·6)	0·2 (0·1–0·5)	-14·3 (-69·2–179·9)	0·3 (0·1–0·7)	0·3 (0·1–0·8)	11·2 (-60·1–237)	0·3 (0·1–0·6)	0·2 (0·1–0·6)	-1·7 (-64·9–209)

	Female			Male			Both		
	Age-standardized YLD rate (per 100,000)		Change (%)	Age-standardized YLD rate (per 100,000)		Change (%)	Age-standardized YLD rate (per 100,000)		Change (%)
	1990	2023		1990	2023		1990	2023	
Republic of Moldova	0·2 (0·1–0·7)	0·2 (0·1–0·5)	-20·9 (-73·2–167·1)	0·5 (0·2–1·3)	0·5 (0·2–1·2)	-4·8 (-65·2–192·7)	0·4 (0·1–0·9)	0·3 (0·1–0·8)	-10·2 (-68·3–185·1)
Russian Federation	0·4 (0·1–0·9)	0·3 (0·1–0·8)	-16·9 (-71·9–172·8)	0·5 (0·1–1·1)	0·4 (0·1–1)	-12 (-69·6–177·6)	0·4 (0·1–1)	0·3 (0·1–0·8)	-14·4 (-70·4–180)
Ukraine	0·3 (0·1–0·8)	0·2 (0·1–0·6)	-25 (-75·2–134·4)	0·6 (0·2–1·4)	0·4 (0·2–1)	-24·7 (-72·9–133·5)	0·4 (0·1–1)	0·3 (0·1–0·8)	-24·7 (-72·7–143·4)
**High-income**	0·3 (0·1–0·8)	0·4 (0·1–1)	13·1 (-61·4–255·7)	2·8 (1·5–5)	2·2 (1·1–4·2)	-20·9 (-54·5–34·4)	1·5 (0·7–2·6)	1·3 (0·6–2·5)	-16·4 (-50·6–77·9)
Australasia	0·2 (0·1–0·5)	0·2 (0·1–0·5)	-7·5 (-68·7–202·2)	0·9 (0·3–2·3)	0·7 (0·3–1·8)	-22·4 (-71·4–140·7)	0·6 (0·2–1·3)	0·5 (0·2–1·1)	-19·7 (-69·2–167·2)
Australia	0·2 (0·1–0·5)	0·2 (0·1–0·5)	-10·2 (-69·6–193·6)	1 (0·3–2·4)	0·7 (0·3–1·8)	-23·5 (-71·8–137·7)	0·6 (0·2–1·3)	0·5 (0·1–1·1)	-21·4 (-69·6–161·9)
New Zealand	0·2 (0·1–0·6)	0·3 (0·1–0·6)	6·4 (-64·4–252·7)	0·8 (0·2–2)	0·7 (0·2–1·6)	-16·3 (-70·5–161·1)	0·5 (0·1–1·2)	0·5 (0·2–1·1)	-10·2 (-67–198·3)
High-income Asia Pacific	0·4 (0·1–1·1)	0·4 (0·1–1·1)	7·4 (-62·2–243·3)	1·5 (0·5–3·8)	1·3 (0·5–3·2)	-14·1 (-68·7–156·7)	1 (0·3–2·3)	0·9 (0·3–2·1)	-9·4 (-65·7–189·8)
Brunei Darussalam	1·6 (0·5–4·2)	1·2 (0·4–3)	-25 (-72·4–145)	2·7 (0·9–6·4)	1·9 (0·6–4·8)	-29·2 (-74·3–115)	2·2 (0·7–5·3)	1·6 (0·5–3·9)	-28 (-74·1–121·4)
Japan	0·4 (0·1–1)	0·5 (0·1–1·2)	22·7 (-56·8–290·3)	1·4 (0·4–3·5)	1·4 (0·5–3·3)	-3·5 (-64·8–190·9)	0·9 (0·3–2·1)	0·9 (0·3–2·1)	1·8 (-61·2–228)
Republic of Korea	0·6 (0·2–1·7)	0·4 (0·1–1·1)	-36·5 (-77·7–105·1)	2·4 (0·8–5·7)	1·2 (0·4–2·9)	-50·4 (-82·3–48·7)	1·5 (0·4–3·2)	0·8 (0·2–1·9)	-47·2 (-79·4–76·7)
Singapore	0·5 (0·1–1·3)	0·5 (0·2–1·3)	3·6 (-65·2–252·4)	2·1 (0·7–5·2)	1·8 (0·6–4·4)	-13·5 (-68·3–161·8)	1·3 (0·4–3)	1·2 (0·4–2·8)	-8·9 (-65·8–196·3)
High-income North America	0·6 (0·2–1·5)	0·7 (0·2–1·8)	14·6 (-60–252·7)	8 (4·5–13·5)	5·7 (3·1–9·7)	-28·7 (-55·1–13)	4·2 (2–6·5)	3·2 (1·6–5·5)	-24·2 (-51·9–44·6)
Canada	0·6 (0·2–1·6)	0·6 (0·2–1·7)	0·5 (-65·1–220·4)	5·2 (1·8–12·1)	2·3 (0·8–5·5)	-56·6 (-84·3–22)	2·9 (0·9–6)	1·4 (0·5–3·5)	-49·9 (-80·7–56·9)
Greenland	0·7 (0·2–1·8)	0·5 (0·2–1·3)	-25·7 (-73·7–146·8)	8·4 (2·9–20·3)	4·8 (1·7–11·6)	-42·3 (-77·6–65·9)	5 (1·4–10·4)	2·8 (1–6·7)	-43·1 (-74·9–91·7)
United States of America	0·6 (0·2–1·5)	0·7 (0·2–1·8)	16·5 (-59·3–256·4)	8·3 (4·7–14·2)	6·1 (3·3–10·5)	-26·2 (-52·8–15·2)	4·3 (2·1–6·6)	3·4 (1·7–5·8)	-21·7 (-49·3–44·9)

Return to Table of Contents

	Female			Male			Both		
	Age-standardized YLD rate (per 100,000)		Change (%)	Age-standardized YLD rate (per 100,000)		Change (%)	Age-standardized YLD rate (per 100,000)		Change (%)
	1990	2023		1990	2023		1990	2023	
Southern Latin America	0·2 (0·1–0·5)	0·1 (0–0·4)	-26·5 (-74·4–141·7)	0·2 (0·1–0·6)	0·1 (0·1–0·4)	-33·9 (-76·7–105·2)	0·2 (0·1–0·5)	0·1 (0–0·4)	-30·4 (-75·7–122·4)
Argentina	0·2 (0–0·4)	0·1 (0–0·3)	-27·9 (-75·1–140·9)	0·2 (0·1–0·4)	0·1 (0–0·3)	-29·4 (-75·1–120·1)	0·2 (0·1–0·4)	0·1 (0–0·3)	-28·6 (-75·2–126·5)
Chile	0·3 (0·1–0·6)	0·2 (0·1–0·5)	-26·8 (-74·2–138·3)	0·4 (0·1–1)	0·2 (0·1–0·6)	-45·2 (-80·1–68·3)	0·3 (0·1–0·8)	0·2 (0·1–0·5)	-37·7 (-77·5–98·6)
Uruguay	0·2 (0·1–0·6)	0·2 (0–0·4)	-31·5 (-76·1–126·7)	0·3 (0·1–0·7)	0·2 (0·1–0·4)	-34·1 (-76·7–111·4)	0·2 (0·1–0·6)	0·2 (0·1–0·4)	-32·9 (-76·9–119·1)
Western Europe	0·1 (0–0·3)	0·1 (0–0·4)	4·1 (-64–241·1)	0·3 (0·1–0·7)	0·3 (0·1–0·8)	17·5 (-59–260·6)	0·2 (0·1–0·5)	0·2 (0·1–0·6)	13·6 (-59·8–262·7)
Andorra	0·1 (0–0·4)	0·1 (0–0·3)	-17·9 (-71·6–170·4)	0·3 (0·1–0·7)	0·3 (0·1–0·7)	0·7 (-64·5–212·1)	0·2 (0·1–0·5)	0·2 (0·1–0·5)	-6 (-67·2–199)
Austria	0·2 (0–0·4)	0·2 (0·1–0·5)	35·5 (-53·3–344·6)	0·4 (0·1–0·9)	0·6 (0·2–1·3)	53·9 (-45·3–376)	0·2 (0·1–0·6)	0·4 (0·1–0·9)	50·2 (-45·8–391·8)
Belgium	0·1 (0–0·3)	0·1 (0–0·2)	-21·3 (-72·6–154·2)	0·2 (0·1–0·5)	0·2 (0·1–0·4)	-27·3 (-73·6–118·8)	0·2 (0–0·4)	0·1 (0–0·3)	-24·9 (-72·7–138·7)
Cyprus	0·1 (0–0·3)	0·1 (0–0·2)	-22 (-72·4–150·7)	0·2 (0·1–0·5)	0·2 (0·1–0·4)	-16·7 (-70·5–151·2)	0·2 (0·1–0·4)	0·1 (0–0·3)	-19·1 (-70·9–155·7)
Denmark	0·2 (0·1–0·5)	0·2 (0·1–0·6)	13·7 (-60·7–266·4)	0·6 (0·2–1·5)	0·6 (0·2–1·4)	-4·3 (-65·3–192·8)	0·4 (0·1–0·9)	0·4 (0·1–0·9)	0·6 (-60·8–242·1)
Finland	0·1 (0–0·4)	0·2 (0–0·4)	3·1 (-64·5–232·8)	0·3 (0·1–0·7)	0·4 (0·2–1·1)	50·8 (-45·6–357·8)	0·2 (0·1–0·5)	0·3 (0·1–0·7)	35·6 (-49·3–337·8)
France	0·1 (0–0·2)	0·1 (0–0·3)	11·8 (-63·3–260·4)	0·2 (0·1–0·6)	0·2 (0·1–0·5)	-7·9 (-67·5–169·7)	0·2 (0·1–0·4)	0·2 (0·1–0·4)	-2 (-66·6–202·2)
Germany	0·2 (0–0·4)	0·2 (0·1–0·6)	39·8 (-51·9–358)	0·3 (0·1–0·7)	0·5 (0·2–1·2)	80·1 (-34·2–447·5)	0·2 (0·1–0·5)	0·4 (0·1–0·8)	66·2 (-39·8–431·3)
Greece	0·2 (0·1–0·4)	0·1 (0–0·3)	-32 (-76–112·7)	0·3 (0·1–0·7)	0·2 (0·1–0·4)	-28·7 (-74·6–123·1)	0·2 (0·1–0·5)	0·1 (0–0·4)	-30·1 (-75·4–120·2)
Iceland	0·2 (0·1–0·5)	0·2 (0·1–0·6)	13·1 (-60·8–271)	0·7 (0·2–1·8)	1·2 (0·4–2·9)	60·4 (-42·6–390·3)	0·5 (0·1–1·1)	0·7 (0·2–1·7)	53·5 (-43·9–387·7)
Ireland	0·1 (0–0·3)	0·1 (0–0·2)	-25·1 (-74·3–147·6)	0·2 (0·1–0·5)	0·2 (0·1–0·4)	-23·8 (-72·2–132·1)	0·2 (0·1–0·4)	0·1 (0–0·3)	-24·4 (-72·5–146·5)

	Female			Male			Both		
	Age-standardized YLD rate (per 100,000)		Change (%)	Age-standardized YLD rate (per 100,000)		Change (%)	Age-standardized YLD rate (per 100,000)		Change (%)
	1990	2023		1990	2023		1990	2023	
Israel	0·1 (0–0·4)	0·1 (0–0·3)	-16·8 (-70·8–164·6)	0·3 (0·1–0·8)	0·2 (0·1–0·6)	-22·4 (-72·5–141·9)	0·2 (0·1–0·5)	0·2 (0·1–0·4)	-20·2 (-71·7–149·7)
Italy	0·1 (0–0·3)	0·1 (0–0·2)	-9·4 (-68·5–194·2)	0·2 (0·1–0·5)	0·2 (0·1–0·5)	0·3 (-64·1–210·5)	0·2 (0–0·4)	0·1 (0·1–0·4)	-2·5 (-66·1–212·1)
Luxembourg	0·1 (0–0·3)	0·1 (0–0·2)	-34·7 (-77·5–106·1)	0·2 (0·1–0·6)	0·1 (0–0·3)	-34·7 (-77–101·2)	0·2 (0·1–0·4)	0·1 (0–0·3)	-34·1 (-76·6–111·4)
Malta	0·1 (0–0·3)	0·1 (0–0·2)	-25·9 (-73·1–148)	0·2 (0·1–0·6)	0·2 (0·1–0·4)	-30·2 (-75·2–111·1)	0·2 (0·1–0·4)	0·1 (0–0·3)	-27 (-73·7–133·4)
Monaco	0·2 (0·1–0·4)	0·1 (0–0·4)	-18·9 (-72·1–173·3)	0·5 (0·1–1·2)	0·4 (0·1–0·8)	-23·3 (-74·7–130·6)	0·3 (0·1–0·8)	0·2 (0·1–0·6)	-20·8 (-73·2–150·4)
Netherlands	0·1 (0–0·3)	0·1 (0–0·3)	-17 (-71·2–172·5)	0·2 (0·1–0·5)	0·2 (0·1–0·4)	-18·6 (-71·1–146·4)	0·2 (0·1–0·4)	0·1 (0–0·3)	-17·8 (-70·9–164·4)
Norway	0·2 (0·1–0·6)	0·3 (0·1–0·8)	31·7 (-52·8–315·1)	0·8 (0·3–2)	1·7 (0·6–3·9)	100·4 (-26·1–503·1)	0·5 (0·2–1·3)	1 (0·4–2·3)	87 (-29·8–486·6)
Portugal	0·2 (0·1–0·6)	0·1 (0–0·3)	-48·2 (-81·2–75·5)	0·3 (0·1–0·8)	0·2 (0·1–0·5)	-20·3 (-70·6–150·2)	0·3 (0·1–0·6)	0·2 (0·1–0·4)	-33 (-75–120·5)
San Marino	0·2 (0–0·4)	0·1 (0–0·4)	-10·3 (-68·6–195·4)	0·4 (0·1–0·9)	0·3 (0·1–0·7)	-20·1 (-71·8–143·2)	0·3 (0·1–0·6)	0·2 (0·1–0·5)	-17·1 (-70·6–165·9)
Spain	0·1 (0–0·3)	0·1 (0–0·2)	-27 (-73·6–143·7)	0·2 (0·1–0·6)	0·2 (0·1–0·5)	-19·8 (-72·9–146)	0·2 (0·1–0·5)	0·1 (0–0·3)	-22·1 (-73·1–149·1)
Sweden	0·2 (0–0·4)	0·4 (0·1–1)	147 (-11·6–685·7)	1·6 (0·5–3·9)	2·2 (0·8–5·3)	41·1 (-46·9–330)	0·9 (0·3–2·1)	1·3 (0·5–3·1)	53·8 (-42·5–372·6)
Switzerland	0·1 (0–0·3)	0·1 (0–0·3)	-7·3 (-67·2–195)	0·3 (0·1–0·6)	0·2 (0·1–0·6)	-9·5 (-67·6–172)	0·2 (0·1–0·4)	0·2 (0·1–0·4)	-8·4 (-66·5–195·4)
United Kingdom	0·1 (0–0·4)	0·1 (0–0·3)	-20·3 (-72·6–161·3)	0·2 (0·1–0·6)	0·2 (0·1–0·4)	-20·2 (-70·1–149·5)	0·2 (0·1–0·5)	0·1 (0–0·3)	-20·1 (-70·6–160·1)
**Latin America and Caribbean**	0·4 (0·1–1)	0·3 (0·1–0·7)	-24·2 (-73·2–143·4)	0·5 (0·2–1·2)	0·4 (0·1–1)	-16·1 (-69·5–157·6)	0·4 (0·1–1·1)	0·3 (0·1–0·8)	-19·9 (-71·4–149·1)
Andean Latin America	0·2 (0·1–0·6)	0·1 (0–0·4)	-34·6 (-77·3–110·1)	0·2 (0·1–0·6)	0·2 (0·1–0·4)	-26·1 (-73·2–128·3)	0·2 (0·1–0·6)	0·2 (0·1–0·4)	-30·3 (-75·1–120·7)
Bolivia (Plurinational State of)	0·2 (0·1–0·5)	0·1 (0–0·4)	-28·7 (-74·5–138)	0·3 (0·1–0·7)	0·2 (0·1–0·5)	-25·6 (-72·4–133·6)	0·2 (0·1–0·6)	0·2 (0·1–0·4)	-26·7 (-73·8–133·3)

	Female			Male			Both		
	Age-standardized YLD rate (per 100,000)		Change (%)	Age-standardized YLD rate (per 100,000)		Change (%)	Age-standardized YLD rate (per 100,000)		Change (%)
	1990	2023		1990	2023		1990	2023	
Ecuador	0·1 (0–0·4)	0·1 (0–0·3)	-27·1 (-75·7–138·2)	0·1 (0–0·3)	0·1 (0–0·3)	-24·9 (-73–134·9)	0·1 (0–0·4)	0·1 (0–0·3)	-26 (-74·4–137)
Peru	0·3 (0·1–0·7)	0·2 (0·1–0·4)	-36·6 (-77·7–101·4)	0·3 (0·1–0·7)	0·2 (0·1–0·5)	-25·1 (-72·8–127·4)	0·3 (0·1–0·7)	0·2 (0·1–0·5)	-30·8 (-75·2–116·5)
Caribbean	0·2 (0·1–0·6)	0·2 (0·1–0·6)	-7·6 (-68–204·5)	0·3 (0·1–0·7)	0·3 (0·1–0·8)	8·8 (-60·9–233·1)	0·3 (0·1–0·7)	0·3 (0·1–0·7)	1·4 (-64·1–218)
Antigua and Barbuda	0·2 (0·1–0·5)	0·2 (0·1–0·5)	-15·3 (-70·5–173·3)	0·3 (0·1–0·7)	0·2 (0·1–0·5)	-19·9 (-71·9–142·2)	0·2 (0·1–0·6)	0·2 (0·1–0·5)	-17·6 (-71·3–156·7)
Bahamas	0·2 (0·1–0·5)	0·2 (0·1–0·4)	-13·7 (-70·2–185·5)	0·3 (0·1–0·7)	0·2 (0·1–0·6)	-11 (-68·7–185·1)	0·2 (0·1–0·6)	0·2 (0·1–0·5)	-12·3 (-69·9–187·9)
Barbados	0·2 (0·1–0·6)	0·2 (0·1–0·5)	-12·5 (-69·2–182)	0·1 (0–0·4)	0·1 (0·1–0·4)	1·9 (-63·7–221·8)	0·2 (0·1–0·5)	0·2 (0·1–0·4)	-7·4 (-67·5–193·3)
Belize	0·2 (0·1–0·4)	0·1 (0–0·4)	-11·6 (-70·2–189·8)	0·2 (0–0·4)	0·1 (0–0·4)	-5·7 (-65·3–187·1)	0·2 (0·1–0·4)	0·1 (0–0·4)	-8·7 (-67·8–188·8)
Bermuda	0·2 (0·1–0·5)	0·2 (0–0·4)	-17·3 (-71·5–167·6)	0·3 (0·1–0·6)	0·2 (0·1–0·5)	-12·5 (-68·6–176·4)	0·2 (0·1–0·5)	0·2 (0·1–0·4)	-14·7 (-69·8–174)
Cuba	0·2 (0·1–0·5)	0·2 (0·1–0·5)	-9·3 (-68·9–196·2)	0·2 (0·1–0·5)	0·2 (0·1–0·6)	15·2 (-58·2–252·7)	0·2 (0·1–0·5)	0·2 (0·1–0·5)	2·9 (-63·7–223)
Dominica	0·1 (0–0·3)	0·1 (0–0·3)	-2·7 (-65·5–219·5)	0·2 (0·1–0·4)	0·2 (0·1–0·4)	-2·2 (-63·1–210)	0·1 (0–0·4)	0·1 (0–0·4)	-1·9 (-63·9–214·6)
Dominican Republic	0·2 (0·1–0·6)	0·2 (0·1–0·5)	-14·6 (-70·3–183·5)	0·4 (0·1–1·1)	0·5 (0·2–1·2)	9·8 (-58·4–220·2)	0·3 (0·1–0·9)	0·3 (0·1–0·8)	1·7 (-62·5–211·3)
Grenada	0·3 (0·1–0·9)	0·3 (0·1–0·7)	-21·2 (-72·3–160·1)	0·4 (0·1–1)	0·3 (0·1–0·9)	-16·3 (-70–158·2)	0·4 (0·1–1)	0·3 (0·1–0·8)	-17·8 (-71–164·4)
Guyana	0·2 (0·1–0·6)	0·1 (0–0·4)	-32·6 (-76·4–125·5)	0·3 (0·1–0·7)	0·2 (0·1–0·4)	-31 (-74·6–117·3)	0·2 (0·1–0·6)	0·2 (0·1–0·4)	-31·8 (-75·5–120·7)
Haiti	0·4 (0·1–1)	0·4 (0·1–0·9)	1·2 (-65·6–226·8)	0·5 (0·2–1·3)	0·5 (0·2–1·2)	-0·5 (-64·3–205·7)	0·4 (0·1–1·1)	0·4 (0·1–1·1)	0·3 (-65·8–209·9)
Jamaica	0·2 (0·1–0·6)	0·2 (0·1–0·5)	-10·8 (-69–193·1)	0·2 (0·1–0·6)	0·2 (0·1–0·5)	-4·1 (-64·7–205·5)	0·2 (0·1–0·6)	0·2 (0·1–0·5)	-7·3 (-66·5–197·2)
Puerto Rico	0·2 (0·1–0·5)	0·1 (0–0·4)	-21·6 (-72·8–156·4)	0·2 (0·1–0·5)	0·1 (0–0·4)	-26·7 (-74·9–118·6)	0·2 (0·1–0·5)	0·1 (0–0·4)	-24·2 (-74·2–139·9)

	Female			Male			Both		
	Age-standardized YLD rate (per 100,000)		Change (%)	Age-standardized YLD rate (per 100,000)		Change (%)	Age-standardized YLD rate (per 100,000)		Change (%)
	1990	2023		1990	2023		1990	2023	
Saint Kitts and Nevis	0·2 (0·1–0·5)	0·1 (0–0·4)	-30·9 (-76·6–127·3)	0·2 (0·1–0·4)	0·1 (0–0·4)	-17·4 (-69·8–152·9)	0·2 (0·1–0·5)	0·1 (0–0·4)	-24·8 (-73·6–139·8)
Saint Lucia	0·3 (0·1–0·7)	0·2 (0·1–0·4)	-37·9 (-78·1–106·9)	0·3 (0·1–0·7)	0·2 (0·1–0·5)	-29·8 (-75·1–114·4)	0·3 (0·1–0·7)	0·2 (0·1–0·5)	-33·7 (-76·6–114)
Saint Vincent and the Grenadines	0·2 (0·1–0·4)	0·1 (0–0·4)	-18·3 (-71·2–167·2)	0·3 (0·1–0·7)	0·3 (0·1–0·6)	-8·9 (-68·2–174·5)	0·2 (0·1–0·6)	0·2 (0·1–0·5)	-11·4 (-68·5–179·1)
Suriname	0·2 (0·1–0·5)	0·2 (0·1–0·5)	-7·7 (-67·7–198·4)	0·2 (0·1–0·6)	0·2 (0·1–0·6)	-4·7 (-64·4–186·7)	0·2 (0·1–0·6)	0·2 (0·1–0·5)	-6·4 (-66·1–191)
Trinidad and Tobago	0·3 (0·1–0·8)	0·2 (0·1–0·6)	-19·4 (-71–163·6)	0·3 (0·1–0·8)	0·3 (0·1–0·7)	-17·1 (-70–158)	0·3 (0·1–0·8)	0·3 (0·1–0·7)	-18·2 (-70·6–160·6)
United States Virgin Islands	0·1 (0–0·3)	0·1 (0–0·3)	-10·7 (-68·6–184·7)	0·1 (0–0·4)	0·2 (0·1–0·4)	19·4 (-57–275·2)	0·1 (0–0·3)	0·1 (0–0·3)	4·7 (-62·7–233·1)
Central Latin America	0·3 (0·1–0·7)	0·2 (0·1–0·6)	-22·4 (-72·6–146·2)	0·3 (0·1–0·8)	0·2 (0·1–0·6)	-21·9 (-71·5–140·4)	0·3 (0·1–0·8)	0·2 (0·1–0·6)	-22·2 (-72·1–143·3)
Colombia	0·4 (0·1–0·9)	0·3 (0·1–0·7)	-26·1 (-74·3–144·9)	0·3 (0·1–0·7)	0·2 (0·1–0·6)	-23·3 (-71·7–129·2)	0·3 (0·1–0·8)	0·2 (0·1–0·6)	-24·9 (-73·1–137·7)
Costa Rica	0·2 (0·1–0·4)	0·1 (0–0·3)	-19·5 (-71·7–159·7)	0·2 (0·1–0·5)	0·1 (0·1–0·4)	-20·1 (-72·2–139·7)	0·2 (0·1–0·4)	0·1 (0–0·4)	-19·8 (-72·1–147·8)
El Salvador	0·3 (0·1–0·7)	0·2 (0·1–0·5)	-22·2 (-73·3–159·4)	0·3 (0·1–0·7)	0·2 (0·1–0·5)	-25·2 (-72·3–129·1)	0·3 (0·1–0·7)	0·2 (0·1–0·5)	-23·5 (-73·1–148·9)
Guatemala	0·2 (0·1–0·5)	0·2 (0–0·4)	-21·3 (-73·2–163·1)	0·2 (0–0·4)	0·1 (0–0·3)	-19·5 (-71·1–145)	0·2 (0·1–0·4)	0·1 (0–0·4)	-20·3 (-71·5–150·6)
Honduras	0·2 (0·1–0·6)	0·2 (0·1–0·6)	-12·9 (-69·9–188·3)	0·4 (0·1–1)	0·3 (0·1–0·8)	-20·7 (-71·7–132·9)	0·3 (0·1–0·8)	0·3 (0·1–0·7)	-17·8 (-71·2–153·8)
Mexico	0·2 (0·1–0·5)	0·1 (0–0·4)	-22·1 (-73–148)	0·2 (0–0·4)	0·1 (0–0·3)	-10·2 (-68·3–171·3)	0·2 (0·1–0·4)	0·1 (0–0·4)	-17 (-70·9–158·6)
Nicaragua	0·3 (0·1–0·8)	0·2 (0·1–0·6)	-18·2 (-71·9–164·3)	0·3 (0·1–0·7)	0·2 (0·1–0·6)	-20·7 (-71·1–147·8)	0·3 (0·1–0·8)	0·2 (0·1–0·6)	-19·4 (-71·5–156)
Panama	0·4 (0·1–1)	0·3 (0·1–0·8)	-16·6 (-71–172·2)	0·3 (0·1–0·8)	0·2 (0·1–0·6)	-16·9 (-70·7–155·1)	0·3 (0·1–0·9)	0·3 (0·1–0·7)	-16·5 (-70·7–165·9)
Venezuela (Bolivarian Republic of)	0·7 (0·2–1·7)	0·5 (0·2–1·3)	-21·2 (-72·1–152·7)	1·1 (0·4–2·8)	0·8 (0·3–1·9)	-29·8 (-73·5–110·9)	0·9 (0·3–2·2)	0·6 (0·2–1·6)	-27·1 (-73–130)

	Female			Male			Both		
	Age-standardized YLD rate (per 100,000)		Change (%)	Age-standardized YLD rate (per 100,000)		Change (%)	Age-standardized YLD rate (per 100,000)		Change (%)
	1990	2023		1990	2023		1990	2023	
Tropical Latin America	0·5 (0·2–1·4)	0·4 (0·1–0·9)	-28·4 (-74·5–130·1)	0·8 (0·3–2)	0·7 (0·2–1·6)	-18·5 (-70·3–149·9)	0·7 (0·2–1·7)	0·5 (0·2–1·2)	-22·9 (-72·5–138·7)
Brazil	0·5 (0·2–1·4)	0·4 (0·1–0·9)	-28·5 (-74·5–130·5)	0·8 (0·3–1·9)	0·6 (0·2–1·5)	-18·5 (-70·3–149·5)	0·6 (0·2–1·6)	0·5 (0·2–1·2)	-22·9 (-72·5–138·4)
Paraguay	1 (0·3–2·7)	0·8 (0·3–2)	-25·2 (-72·6–135·4)	1·8 (0·6–4·4)	1·5 (0·5–3·8)	-13·1 (-68·4–160·1)	1·4 (0·5–3·5)	1·2 (0·4–2·8)	-17·8 (-70·2–154·4)
North Africa and Middle East	0·9 (0·3–2)	0·6 (0·2–1·3)	-33·3 (-69·4–57·8)	2 (0·7–4·4)	1·5 (0·7–3·3)	-23·8 (-61·7–63·5)	1·4 (0·5–3·2)	1·1 (0·5–2·3)	-25·9 (-63·7–60·5)
Afghanistan	0·5 (0·1–1·2)	0·4 (0·1–1)	-19·8 (-71·5–166)	2·3 (0·7–5·4)	2·7 (1–6·4)	18·3 (-55·5–224·9)	1·3 (0·4–3·1)	1·5 (0·6–3·7)	16·8 (-54·6–233·9)
Algeria	0·7 (0·2–1·7)	0·5 (0·2–1·4)	-23·9 (-71·9–144)	3·7 (1·3–9)	3 (1·1–7)	-19·9 (-69·6–116·9)	2·2 (0·7–5·3)	1·8 (0·6–4·2)	-20·4 (-69·7–121·7)
Bahrain	0·6 (0·2–1·6)	0·4 (0·1–1)	-37·7 (-78·7–92·4)	1·2 (0·4–2·9)	0·8 (0·3–1·9)	-35 (-76·8–97·7)	1 (0·3–2·3)	0·6 (0·2–1·6)	-34 (-77·1–100·4)
Egypt	0·3 (0·1–0·8)	0·2 (0·1–0·7)	-24·6 (-72·6–147·1)	1·1 (0·4–2·7)	0·9 (0·3–2·4)	-13·1 (-68·5–163·4)	0·7 (0·2–1·8)	0·6 (0·2–1·5)	-15·1 (-70–159·3)
Iran (Islamic Republic of)	0·6 (0·2–1·6)	0·4 (0·1–1)	-41·8 (-79·6–82·2)	1·1 (0·4–2·7)	0·8 (0·3–1·9)	-30·8 (-75·6–110·8)	0·9 (0·3–2·2)	0·6 (0·2–1·4)	-34·9 (-77·4–97·7)
Iraq	0·5 (0·2–1·3)	0·4 (0·1–0·9)	-30·2 (-75·7–119·6)	0·6 (0·2–1·6)	0·5 (0·2–1·4)	-15·2 (-69·1–157)	0·6 (0·2–1·4)	0·5 (0·2–1·1)	-21·5 (-72·2–138·1)
Jordan	0·4 (0·1–1)	0·2 (0·1–0·5)	-44·9 (-81–81·9)	0·5 (0·2–1·3)	0·3 (0·1–0·8)	-42·6 (-79·4–82·5)	0·4 (0·1–1·1)	0·3 (0·1–0·6)	-43·2 (-80–82·6)
Kuwait	0·4 (0·1–1)	0·3 (0·1–0·8)	-24·9 (-74·2–145·5)	0·8 (0·2–1·9)	0·6 (0·2–1·5)	-20·7 (-72·4–139·6)	0·6 (0·2–1·5)	0·5 (0·2–1·2)	-19·4 (-72·6–143·1)
Lebanon	0·5 (0·2–1·3)	0·4 (0·1–1)	-26·3 (-73·9–141·7)	0·8 (0·3–2·1)	0·7 (0·2–1·7)	-17 (-69·5–146·7)	0·7 (0·2–1·7)	0·5 (0·2–1·3)	-20·4 (-71·5–141·6)
Libya	0·4 (0·1–1·1)	0·4 (0·1–1)	-10 (-68–195·8)	0·9 (0·3–2·2)	1 (0·3–2·4)	13·1 (-59·2–257·9)	0·7 (0·2–1·7)	0·7 (0·2–1·8)	4·2 (-62·6–233·3)
Morocco	0·3 (0·1–0·8)	0·2 (0·1–0·6)	-26·6 (-73·8–138·7)	0·6 (0·2–1·6)	0·5 (0·2–1·2)	-19 (-69·4–148·7)	0·5 (0·2–1·2)	0·4 (0·1–0·9)	-21·1 (-71·1–146·9)
Oman	0·6 (0·2–1·6)	0·5 (0·2–1·3)	-15·6 (-70·6–164·7)	0·8 (0·3–2·1)	0·7 (0·2–1·8)	-13·1 (-68·1–164·6)	0·8 (0·2–1·9)	0·7 (0·2–1·6)	-13·6 (-69·1–161·7)

	Female			Male			Both		
	Age-standardized YLD rate (per 100,000)		Change (%)	Age-standardized YLD rate (per 100,000)		Change (%)	Age-standardized YLD rate (per 100,000)		Change (%)
	1990	2023		1990	2023		1990	2023	
Palestine	0·5 (0·2–1·3)	0·4 (0·1–1)	-27·6 (-74·5–128·9)	1·2 (0·4–2·9)	0·9 (0·3–2·3)	-22·2 (-71·1–128·2)	0·9 (0·3–2·1)	0·7 (0·2–1·7)	-23·1 (-72·1–133·2)
Qatar	0·3 (0·1–0·9)	0·3 (0·1–0·7)	-22·2 (-72·5–148·9)	0·7 (0·2–1·6)	0·6 (0·2–1·5)	-13·2 (-67·6–161·5)	0·6 (0·2–1·4)	0·5 (0·2–1·2)	-14·8 (-69·8–150·5)
Saudi Arabia	0·5 (0·1–1·2)	0·5 (0·2–1·2)	3·2 (-63·6–232·5)	0·5 (0·2–1·3)	0·5 (0·2–1·3)	-0·8 (-65·1–206·8)	0·5 (0·2–1·2)	0·5 (0·2–1·3)	1·2 (-64·1–218·3)
Sudan	0·4 (0·1–1)	0·3 (0·1–0·8)	-21·4 (-71·9–147·1)	4·2 (1·3–10·3)	4·5 (1·7–10·6)	6 (-58·7–182·5)	2·3 (0·8–5·8)	2·4 (0·9–5·8)	6·2 (-60·1–173·3)
Syrian Arab Republic	0·3 (0·1–0·7)	0·2 (0·1–0·6)	-12·1 (-68·4–181·1)	0·3 (0·1–0·7)	0·3 (0·1–0·8)	14·8 (-59·3–263·3)	0·3 (0·1–0·7)	0·3 (0·1–0·7)	0·9 (-63·9–214·5)
Tunisia	1·9 (0·6–4·6)	1 (0·3–2·5)	-47 (-81–67·7)	3·4 (1·1–7·7)	2·1 (0·7–4·8)	-38·5 (-77·1–78·2)	2·6 (0·9–6)	1·5 (0·5–3·6)	-42 (-79–72)
Türkiye	0·7 (0·2–1·7)	0·5 (0·2–1·3)	-25·3 (-74·1–138·6)	0·7 (0·2–1·7)	0·5 (0·2–1·3)	-29·8 (-74·4–116·1)	0·7 (0·2–1·7)	0·5 (0·2–1·3)	-27·6 (-74–126)
United Arab Emirates	0·3 (0·1–0·8)	0·2 (0·1–0·5)	-38·9 (-78·5–102·1)	0·5 (0·2–1·4)	0·4 (0·1–1)	-30·8 (-74·8–118·1)	0·5 (0·1–1·1)	0·3 (0·1–0·8)	-30·3 (-74·1–128)
Yemen	11·2 (4·3–23)	5·9 (2·8–10·4)	-47·2 (-73–8·6)	24·9 (10·8–46·4)	13·5 (6·9–23·6)	-45·8 (-69·4–11·5)	18 (7·5–34·6)	9·7 (5·3–16·2)	-46·3 (-70–7·2)
South Asia	14·6 (6·3–28·5)	14·2 (7·6–25·6)	-3·2 (-45·4–76)	26 (12·4–45·8)	25·4 (15–42·1)	-2·3 (-38·9–64·1)	20·6 (9·3–37·4)	19·9 (11·4–33·5)	-3·3 (-42·1–64·5)
Bangladesh	36·4 (17·5–63·6)	41·2 (24·5–65·2)	13·4 (-23–71·4)	25·5 (11·5–45·9)	28·1 (17·4–41)	10·5 (-27·3–88·5)	30·7 (14·3–53·7)	34·8 (21·3–52·1)	13·2 (-23·5–78·8)
Bhutan	13 (4·9–27)	11·6 (5·5–22·7)	-10·7 (-52·1–89·4)	22 (8·9–41·9)	19·7 (10·1–34·5)	-10·6 (-49·3–68·1)	17·9 (6·9–34·5)	15·8 (7·9–28·5)	-11·5 (-50·3–72·4)
India	13·2 (5·5–26·2)	12·2 (6·3–22·6)	-7·6 (-50·7–78·2)	27·7 (13·4–48·6)	26·3 (15·2–43·7)	-5·1 (-41·8–57·3)	20·8 (9·5–37·2)	19·4 (10·8–32·5)	-6·7 (-46·3–60·4)
Nepal	11·1 (4·3–22·8)	10·2 (4·5–20·6)	-8·6 (-52·5–95·3)	30 (14·4–52·8)	33·6 (18·5–54·6)	11·9 (-25·6–76·3)	20·5 (9·3–38·2)	21·4 (11·6–36·5)	4·6 (-34·9–75·1)
Pakistan	8·6 (3·2–19·2)	9·7 (3·7–20·9)	13 (-52·5–182·6)	14·3 (5·6–28·5)	14·9 (7–29)	4·3 (-51–137·9)	11·6 (4·5–24)	12·4 (5·6–25·4)	6·7 (-52·3–149·2)
**Southeast Asia, East Asia, and Oceania**	2·9 (1–6·6)	2·1 (0·8–5)	-28·5 (-69·7–89·8)	2·2 (0·8–5·2)	2·5 (1–5·9)	13·1 (-55·2–199·5)	2·6 (0·9–6)	2·3 (0·9–5·5)	-10·2 (-63·7–134·2)

	Female			Male			Both		
	Age-standardized YLD rate (per 100,000)		Change (%)	Age-standardized YLD rate (per 100,000)		Change (%)	Age-standardized YLD rate (per 100,000)		Change (%)
	1990	2023		1990	2023		1990	2023	
East Asia	0·6 (0·2–1·5)	0·7 (0·2–1·8)	17·3 (-59·9–272·1)	1·2 (0·4–2·9)	1·6 (0·6–4·2)	39·6 (-50·8–331·5)	0·9 (0·3–2·2)	1·2 (0·4–3)	31·8 (-54–310·6)
China	0·6 (0·2–1·5)	0·7 (0·2–1·8)	18·1 (-59·6–274·4)	1·2 (0·4–3)	1·7 (0·6–4·2)	40·1 (-50·6–333·5)	0·9 (0·3–2·2)	1·2 (0·4–3)	32·5 (-53·7–312·7)
Democratic People's Republic of Korea	0·3 (0·1–0·8)	0·3 (0·1–0·8)	-1 (-64·1–223·9)	0·4 (0·1–1)	0·4 (0·1–1·1)	-0·8 (-65·4–211·8)	0·4 (0·1–0·9)	0·4 (0·1–0·9)	-0·9 (-64–223·6)
Taiwan	0·6 (0·2–1·6)	0·5 (0·2–1·3)	-21·1 (-71·5–151·7)	1·2 (0·4–2·8)	1·3 (0·5–3·1)	15·6 (-58–256·3)	0·9 (0·3–2·2)	0·9 (0·3–2·1)	0·8 (-63·5–209·2)
Oceania	5·3 (1·9–12)	6·6 (2·8–13·5)	22·7 (-45·7–203·5)	3·7 (1·3–8·6)	3·7 (1·4–8·7)	-1·9 (-61·1–164·5)	4·5 (1·5–10·2)	5·1 (2·1–11·1)	12·3 (-51·6–179)
American Samoa	2·1 (0·7–5·3)	2·1 (0·7–5·4)	-1·1 (-63·9–217)	3·6 (1·2–8·7)	3·5 (1·3–8·4)	-4·6 (-63·6–172·4)	2·9 (0·9–7)	2·8 (1–6·9)	-3·4 (-64·2–187·6)
Cook Islands	2·6 (0·8–6·5)	2·7 (0·9–7·1)	4·3 (-62·5–233·3)	3·6 (1·2–8·8)	3·5 (1·2–8·5)	-4 (-63·5–171·3)	3·1 (1–7·7)	3·1 (1·1–7·8)	-1·6 (-63·3–192·4)
Fiji	2·2 (0·7–5·5)	2·1 (0·7–5·5)	-2·9 (-65–204·5)	3·1 (1–7·8)	2·7 (1–6·6)	-14·8 (-68–152·6)	2·7 (0·8–6·6)	2·4 (0·8–6)	-10 (-66·8–173·7)
Guam	2·3 (0·7–5·7)	3·4 (1·4–7·8)	50·9 (-40·5–334·4)	5·1 (1·9–11·3)	7·6 (3·7–14·7)	49 (-27·4–232·3)	3·8 (1·3–8·7)	5·5 (2·6–11)	45·3 (-32·8–244·6)
Kiribati	3·1 (1–7·8)	3·9 (1·5–8·9)	26·9 (-47·9–252·1)	9·7 (3·4–21·7)	10·4 (4·4–21·9)	7·6 (-50·9–165·5)	6·3 (2·2–14·3)	7·1 (3–15·2)	12·4 (-50·1–188·9)
Marshall Islands	3 (1–7·4)	5·2 (2·2–11·4)	73 (-26·6–338·9)	5·6 (2·2–11·8)	7·4 (3·3–15·7)	32·3 (-37·8–192·2)	4·3 (1·6–9·6)	6·3 (2·7–13·6)	45·8 (-33·5–236·8)
Micronesia (Federated States of)	3·6 (1·2–8·5)	3·1 (1·1–7·8)	-12·1 (-66·3–163)	6·3 (2·2–14·1)	5·8 (2·1–13·5)	-7·8 (-64·2–143·2)	4·9 (1·7–11·3)	4·5 (1·6–10·6)	-9·3 (-65·5–148·6)
Nauru	1 (0·3–2·7)	1 (0·3–2·7)	-0·9 (-64·5–223·1)	1·1 (0·4–2·8)	1·1 (0·4–2·8)	-4·6 (-64·5–200·1)	1·1 (0·3–2·7)	1·1 (0·4–2·7)	-2·9 (-64·5–214·4)
Niue	1·7 (0·5–4·4)	1·5 (0·5–3·9)	-13·4 (-68·4–189·3)	1·8 (0·6–4·3)	1·7 (0·6–4·2)	-2·8 (-63·9–185·6)	1·7 (0·5–4·3)	1·6 (0·5–4)	-8·4 (-66·3–186·6)
Northern Mariana Islands	3 (0·9–7·5)	3·3 (1·2–8)	9·5 (-59·3–219·8)	4·8 (1·6–11)	5·2 (1·9–11·8)	8·9 (-55·6–182·6)	4 (1·3–9·4)	4·3 (1·6–10·1)	8·5 (-57·2–190·8)
Palau	28·4 (10·8–58·4)	35 (16·9–67·2)	23·2 (-39·2–177·2)	32·6 (12·6–64·9)	32·8 (16·2–64·5)	0·7 (-49·8–115·1)	30·6 (11·7–61·9)	33·8 (16·8–65·1)	10·6 (-44·6–141·3)

	Female			Male			Both		
	Age-standardized YLD rate (per 100,000)		Change (%)	Age-standardized YLD rate (per 100,000)		Change (%)	Age-standardized YLD rate (per 100,000)		Change (%)
	1990	2023		1990	2023		1990	2023	
Papua New Guinea	6·7 (2·4–14·8)	7·6 (3·3–15·3)	13·2 (-49·3–175·3)	3·4 (1·1–7·7)	3·4 (1·2–8)	-1·5 (-61·1–164·9)	5 (1·7–11·1)	5·4 (2·3–11·7)	8·1 (-52·8–165·2)
Samoa	1·6 (0·5–4·1)	1·5 (0·5–3·9)	-6·2 (-68·1–196·8)	2 (0·6–5)	2 (0·7–5·1)	1 (-62·3–202·1)	1·8 (0·6–4·6)	1·8 (0·6–4·6)	-2·7 (-65·2–197·9)
Solomon Islands	3·3 (1–8·2)	3·2 (1·1–8·1)	-1·6 (-64·2–201·6)	4·8 (1·6–11·4)	5 (1·8–12)	4 (-60·4–190·4)	4·1 (1·3–10)	4·1 (1·4–10·3)	1·5 (-62·3–190·2)
Tokelau	2·3 (0·7–5·8)	2·2 (0·7–5·6)	-5 (-66·4–208)	3 (1–7·2)	2·9 (1–7·1)	-1·9 (-62·7–181)	2·6 (0·8–6·4)	2·6 (0·9–6·5)	-2·9 (-64·3–191·1)
Tonga	0·9 (0·3–2·2)	0·7 (0·2–1·7)	-22·7 (-73·3–149·3)	1·2 (0·4–3)	1 (0·3–2·5)	-16·4 (-69·4–157·6)	1 (0·3–2·6)	0·8 (0·3–2·1)	-20 (-72–151·4)
Tuvalu	1 (0·3–2·7)	1 (0·3–2·7)	-2 (-64·7–218)	1·7 (0·5–4·1)	1·7 (0·6–4·3)	0·6 (-63·8–202·4)	1·3 (0·4–3·3)	1·4 (0·4–3·4)	2·3 (-63·7–208·6)
Vanuatu	2·7 (0·8–6·7)	2·8 (0·9–7·1)	3·4 (-61·8–233·5)	5·5 (1·8–13·1)	5·6 (2–13·8)	3·2 (-59·9–199·7)	4·1 (1·3–10·1)	4·2 (1·5–10·5)	2·3 (-61·7–202·2)
Southeast Asia	10·3 (3·6–23·1)	6·5 (2·6–15·2)	-36·9 (-72·1–59·1)	5·7 (2·1–12·8)	5·2 (2·2–11·6)	-8·2 (-61–119·6)	8·1 (3–18·5)	5·9 (2·5–13·8)	-27·1 (-68·4–80·3)
Cambodia	36·1 (16·1–65·7)	26·2 (12·4–47·2)	-27·4 (-61·1–55·6)	4·6 (1·6–11)	3·4 (1·2–7·9)	-26 (-72·2–108·1)	21·8 (9·9–41·8)	15·3 (8·6–29)	-29·9 (-56·4–52·9)
Indonesia	11·2 (4–25·4)	5·1 (1·8–12·5)	-54 (-81·9–28·2)	1·6 (0·5–4·1)	1·5 (0·5–3·9)	-6·7 (-66–175·3)	6·5 (2·4–15·4)	3·3 (1·2–8·6)	-49 (-79·6–46·4)
Lao People's Democratic Republic	16 (5·6–35·7)	14·1 (6·1–32·1)	-11·8 (-63·1–120)	1·7 (0·6–4·2)	1·4 (0·5–3·4)	-20·5 (-70·4–135·2)	9·2 (3·2–20·8)	7·7 (3·3–18)	-15·9 (-64·1–113·2)
Malaysia	3·9 (1·2–9·3)	3·9 (1·3–9·9)	0·3 (-64·3–213·8)	4·1 (1·4–10)	5·9 (2·2–14·1)	42 (-46·2–295·7)	4 (1·3–9·6)	4·9 (1·8–11·9)	23·2 (-55·3–261·9)
Maldives	12·5 (4·2–29)	7·7 (2·7–18·1)	-38·3 (-75·8–64·1)	15·7 (5·7–34·4)	10·7 (4·4–22·9)	-31·8 (-70–79)	14·2 (5–32)	9·6 (3·8–21·1)	-32·1 (-71·6–74·4)
Mauritius	9·2 (3–20·9)	6·4 (2·1–15·9)	-31 (-75·6–103·7)	3·6 (1·2–8·8)	3·1 (1·1–7·6)	-13·9 (-67·6–159·3)	6·4 (2·3–15·9)	4·7 (1·7–12·4)	-26·1 (-74·2–110·1)
Myanmar	19·9 (6·9–44)	14·3 (5·4–32·7)	-28·1 (-68·9–79·8)	24·2 (9–50·5)	27·3 (11·8–54·7)	12·9 (-49·4–157·1)	22 (8–47·4)	20·5 (8·6–42·6)	-7 (-58·8–120·3)
Philippines	3·4 (1–8·6)	2·9 (1–7·5)	-13·5 (-68·7–162·2)	5·7 (1·9–13·6)	6·1 (2·4–14·5)	6·8 (-58–171·4)	4·6 (1·4–11)	4·5 (1·6–10·2)	-0·4 (-63·7–157·1)
Seychelles	5·5 (1·8–13·3)	4·5 (1·5–11·4)	-18·2 (-69·4–142·9)	3·8 (1·2–9·3)	3·4 (1·2–8·1)	-11·3 (-69·2–176·5)	4·7 (1·6–11·9)	3·9 (1·4–10·1)	-16·9 (-69·5–153·3)

	Female			Male			Both		
	Age-standardized YLD rate (per 100,000)		Change (%)	Age-standardized YLD rate (per 100,000)		Change (%)	Age-standardized YLD rate (per 100,000)		Change (%)
	1990	2023		1990	2023		1990	2023	
Sri Lanka	12 (4–28)	10·4 (3·6–24·4)	-13·7 (-66·6–144·5)	25·5 (10–53·4)	23 (9·9–45·2)	-10 (-59–101·2)	18·8 (7–40·7)	16·3 (6·6–34·2)	-12·8 (-62·5–105)
Thailand	11 (3·8–25·5)	8 (2·9–18·6)	-27·4 (-72–96·4)	4 (1·3–9·9)	3·6 (1·3–8·8)	-11·7 (-68·1–154·4)	7·6 (2·7–18·2)	5·9 (2·1–14·2)	-22·4 (-70·8–108·9)
Timor-Leste	7·3 (2·4–17·8)	5·3 (1·8–13·2)	-27·6 (-72·4–112·6)	3 (1–7·4)	3·2 (1·1–8)	7·8 (-60–217·2)	5·1 (1·7–12·5)	4·2 (1·5–10·6)	-16·7 (-68·7–140·1)
Viet Nam	5·9 (1·9–14·4)	4·2 (1·4–10·1)	-28·6 (-72·5–113·5)	1·7 (0·5–4)	1·2 (0·4–3·1)	-26 (-73·5–125·4)	3·9 (1·4–10·6)	2·7 (1–7·3)	-29·8 (-73·3–109·4)
**Sub-Saharan Africa**	1·7 (0·6–4·2)	1·5 (0·5–3·7)	-13·1 (-66·7–156·7)	1·9 (0·7–4·3)	1·8 (0·7–3·9)	-7·2 (-62·3–129·7)	1·8 (0·6–4·3)	1·6 (0·6–3·8)	-10·1 (-64·7–138·5)
Central Sub-Saharan Africa	1·3 (0·4–3·3)	0·8 (0·3–2·2)	-36·2 (-77·4–95·7)	1·1 (0·4–2·7)	1·1 (0·4–2·8)	0·6 (-63–200·3)	1·2 (0·4–3)	1 (0·3–2·5)	-19·6 (-71·1–142·7)
Angola	0·5 (0·1–1·2)	0·4 (0·1–1·1)	-6·8 (-66·3–199·6)	0·5 (0·2–1·3)	0·5 (0·2–1·2)	-12·4 (-68·7–169·9)	0·5 (0·2–1·3)	0·5 (0·2–1·2)	-10·1 (-68·1–188·5)
Central African Republic	1·3 (0·4–3·2)	1·2 (0·4–3·3)	-2·1 (-65·8–206·8)	0·9 (0·3–2·2)	0·9 (0·3–2·3)	-1·8 (-63·9–196·8)	1·1 (0·4–2·8)	1·1 (0·4–2·9)	-2·1 (-64·9–203·1)
Congo	1·4 (0·4–3·6)	1·2 (0·4–3)	-15·2 (-69·5–179·3)	0·7 (0·2–1·7)	0·6 (0·2–1·5)	-13·2 (-67·3–170·1)	1 (0·3–2·8)	0·9 (0·3–2·3)	-15·2 (-69·6–171·5)
Democratic Republic of the Congo	1·6 (0·5–3·9)	1 (0·3–2·4)	-39·7 (-78·9–81·9)	1·4 (0·4–3·3)	1·4 (0·5–3·5)	5·5 (-61·1–212·8)	1·5 (0·5–3·6)	1·2 (0·4–3)	-18·9 (-70·9–139·7)
Equatorial Guinea	1 (0·3–2·5)	0·7 (0·2–1·8)	-27 (-74·1–133·2)	0·8 (0·3–2)	0·5 (0·2–1·3)	-32·1 (-75·2–102·2)	0·9 (0·3–2·3)	0·6 (0·2–1·6)	-30·9 (-73·5–132·1)
Gabon	0·5 (0·2–1·3)	0·4 (0·1–1·1)	-15·3 (-70–169·6)	0·3 (0·1–0·6)	0·2 (0·1–0·6)	-4·7 (-65·3–191·9)	0·4 (0·1–1)	0·3 (0·1–0·9)	-11 (-69·4–171·1)
Eastern Sub-Saharan Africa	1·8 (0·6–4·3)	1·6 (0·6–3·8)	-10 (-63·5–144)	3·2 (1·2–7·1)	2·9 (1·3–6·3)	-8·8 (-61–114)	2·5 (0·9–5·6)	2·2 (1–5)	-9·2 (-62·2–118·8)
Burundi	2·3 (0·7–5·7)	1·9 (0·6–4·5)	-20·3 (-70·8–135·5)	5·9 (2·1–13·7)	3·8 (1·4–8·9)	-35·4 (-74·9–68·5)	4 (1·3–9·3)	2·8 (1–6·8)	-29·4 (-72·4–98·6)
Comoros	6 (2–14)	4·1 (1·4–10)	-31 (-73·6–93·4)	10·2 (3·8–22·4)	8·1 (3·1–18·1)	-20·2 (-66·1–90)	8 (2·9–18)	6·1 (2·3–13·8)	-23·6 (-68·8–84·9)
Djibouti	1·4 (0·4–3·6)	1·2 (0·4–3·2)	-12·2 (-68·8–181·8)	1·8 (0·6–4·5)	1·5 (0·5–3·5)	-19·3 (-69·8–134·8)	1·6 (0·5–4·1)	1·4 (0·5–3·4)	-16·1 (-69·9–153·6)

	Female			Male			Both		
	Age-standardized YLD rate (per 100,000)		Change (%)	Age-standardized YLD rate (per 100,000)		Change (%)	Age-standardized YLD rate (per 100,000)		Change (%)
	1990	2023		1990	2023		1990	2023	
Eritrea	0·4 (0·1–0·9)	0·3 (0·1–0·9)	-2·7 (-65·2–212·9)	4·3 (1·5–10·3)	3·4 (1·2–8)	-20·7 (-69·8–127·3)	2·2 (0·7–5)	1·9 (0·6–4·1)	-14·7 (-68·4–145·6)
Ethiopia	0·5 (0·2–1·3)	0·4 (0·1–1)	-21·9 (-72·7–158·9)	2·1 (0·7–4·9)	1·9 (0·7–4·2)	-10·7 (-66·8–134·9)	1·3 (0·4–3·2)	1·1 (0·4–2·7)	-12·6 (-68·6–130·9)
Kenya	1·3 (0·4–3·4)	1·2 (0·4–2·9)	-10·3 (-68·5–185·4)	1·7 (0·6–4·4)	1·6 (0·6–3·9)	-6·6 (-65·9–176)	1·5 (0·5–3·9)	1·4 (0·5–3·4)	-8·2 (-67·4–181·2)
Madagascar	7·6 (2·6–17·5)	8·4 (3·8–18)	10·7 (-49·8–159)	23·1 (9·4–45·9)	20·6 (9·5–41·1)	-10·7 (-56·3–87·8)	15·2 (6–31·6)	14·3 (6·6–29·2)	-5·9 (-55·3–99·9)
Malawi	1·8 (0·6–4·4)	1·4 (0·4–3·5)	-24·4 (-72·3–133)	1·1 (0·4–2·7)	0·9 (0·3–2·2)	-15·8 (-70–168·6)	1·5 (0·5–3·6)	1·2 (0·4–2·9)	-21·2 (-71–144)
Mozambique	2·2 (0·7–5·4)	1·5 (0·5–3·8)	-33·6 (-75·5–100·6)	2·5 (0·8–6·2)	2·3 (0·8–5·8)	-8·2 (-67·2–174·1)	2·3 (0·8–5·8)	1·8 (0·6–4·7)	-20·9 (-72–138·1)
Rwanda	3·6 (1·1–8·7)	2·3 (0·8–5·7)	-35·3 (-76·7–88·1)	0·6 (0·2–1·7)	0·5 (0·2–1·3)	-18·9 (-71·1–145·1)	2·2 (0·7–5·6)	1·5 (0·5–3·8)	-32·9 (-75·9–94)
Somalia	1·2 (0·4–3)	1 (0·3–2·7)	-12·5 (-68·8–183·1)	1·6 (0·6–4·1)	1·4 (0·5–3·4)	-15·5 (-69·4–158·7)	1·4 (0·5–3·6)	1·2 (0·4–3·1)	-14·5 (-70–168·1)
South Sudan	1·3 (0·4–3·2)	1·2 (0·4–2·9)	-9·3 (-68·4–190·6)	1·8 (0·6–4·3)	1·6 (0·5–3·8)	-13·4 (-69·1–167·1)	1·6 (0·5–3·8)	1·4 (0·5–3·3)	-12·9 (-69·8–172·4)
Uganda	2 (0·7–5·1)	1·8 (0·6–4·6)	-10·6 (-66·8–176)	0·9 (0·3–2·3)	0·9 (0·3–2·1)	-7·1 (-67·2–185·5)	1·5 (0·5–3·8)	1·4 (0·5–3·6)	-9·3 (-66·9–187·4)
United Republic of Tanzania	0·9 (0·3–2·2)	0·8 (0·2–1·9)	-15·9 (-70·1–162·9)	1·2 (0·4–2·8)	1·1 (0·4–2·8)	-2·7 (-63·2–202)	1 (0·3–2·5)	0·9 (0·3–2·3)	-8·6 (-66·5–183·2)
Zambia	1·3 (0·4–3·3)	1·3 (0·4–3·3)	-1·3 (-65–211·4)	0·9 (0·3–2·3)	1·2 (0·4–2·9)	26·9 (-54·4–293)	1·1 (0·4–2·8)	1·2 (0·4–3·1)	10·1 (-60·3–248·5)
Southern Sub-Saharan Africa	2·3 (0·7–5·5)	2 (0·7–5)	-12·2 (-67·4–160·6)	0·8 (0·3–2)	0·6 (0·2–1·4)	-26·3 (-73·9–126·1)	1·6 (0·6–4·2)	1·3 (0·5–3·7)	-15·9 (-68·2–157·1)
Botswana	7·4 (2·6–16·7)	5·4 (2·3–12·1)	-26·6 (-68·9–78·2)	2·2 (0·8–5·6)	1·6 (0·5–3·8)	-30 (-73·6–94·6)	5 (1·8–11·4)	3·6 (1·5–8·5)	-28·8 (-69·1–78·3)
Eswatini	0·8 (0·2–1·9)	0·8 (0·3–2·2)	9·9 (-59·4–251·1)	1·2 (0·4–3·1)	1·1 (0·4–2·6)	-12·1 (-67·6–177·3)	1 (0·3–2·5)	1 (0·3–2·4)	-1·8 (-64·8–207·4)
Lesotho	1·1 (0·3–2·7)	1·2 (0·4–3·1)	14·1 (-58–244·8)	1 (0·3–2·6)	1 (0·3–2·3)	-6·3 (-66·3–185)	1 (0·3–2·7)	1·1 (0·4–2·9)	4·3 (-60·5–238·4)
Namibia	1·9 (0·6–4·9)	2 (0·6–5)	1·9 (-63·6–204·3)	1·1 (0·3–2·6)	0·9 (0·3–2·1)	-20·2 (-71·5–147·8)	1·5 (0·5–4·1)	1·4 (0·5–4)	-5·8 (-65·4–191·6)

	Female			Male			Both		
	Age-standardized YLD rate (per 100,000)		Change (%)	Age-standardized YLD rate (per 100,000)		Change (%)	Age-standardized YLD rate (per 100,000)		Change (%)
	1990	2023		1990	2023		1990	2023	
South Africa	2·4 (0·8–5·9)	1·9 (0·6–4·6)	-22·2 (-71·5–134)	0·8 (0·3–2)	0·5 (0·2–1·3)	-32·4 (-76·1–107·9)	1·6 (0·6–4·5)	1·2 (0·5–3·4)	-24·8 (-72·1–130·6)
Zimbabwe	1·2 (0·4–3)	2·2 (0·7–5·4)	76·7 (-34·3–439·7)	0·5 (0·2–1·2)	0·5 (0·2–1·3)	7·2 (-62·1–228·7)	0·9 (0·3–2·3)	1·4 (0·5–3·8)	60·8 (-40·6–403·2)
Western Sub-Saharan Africa	1·4 (0·5–3·6)	1·3 (0·4–3·4)	-6·7 (-66·5–191·3)	1·2 (0·4–3)	1·2 (0·4–2·9)	-3·7 (-64–177·3)	1·3 (0·4–3·4)	1·3 (0·4–3·2)	-5·1 (-65·7–182·7)
Benin	2·1 (0·7–5·3)	2·1 (0·7–5·4)	1·6 (-63·6–196·1)	1·2 (0·4–3)	1·2 (0·4–2·8)	-2·7 (-63·7–168·3)	1·7 (0·5–4·2)	1·7 (0·6–4·2)	-1·2 (-63·6–193·6)
Burkina Faso	2·6 (0·8–6·3)	2·3 (0·8–5·8)	-12 (-66·6–154·4)	2·4 (0·8–5·7)	1·9 (0·6–4·5)	-21·7 (-69·7–117·7)	2·5 (0·8–6·1)	2·1 (0·7–5·2)	-16·4 (-68·4–132)
Cabo Verde	1·2 (0·3–2·9)	0·7 (0·2–1·9)	-36 (-77·4–103·4)	0·8 (0·3–2)	0·6 (0·2–1·4)	-29·5 (-74·7–117·4)	1 (0·3–2·5)	0·6 (0·2–1·7)	-34·5 (-76·7–107·1)
Cameroon	3·6 (1·2–8·5)	3·9 (1·4–9·1)	5·6 (-60·2–187·2)	1·2 (0·4–3)	1·3 (0·5–3·3)	13·6 (-57·4–229)	2·5 (0·8–5·8)	2·6 (0·9–6·3)	5·8 (-59·3–197·8)
Chad	2·4 (0·8–5·9)	2·5 (0·8–6·4)	5·4 (-60·8–211·2)	2·7 (0·9–6·5)	2·7 (0·9–6·4)	-1·7 (-62·5–166·3)	2·5 (0·8–6·2)	2·6 (0·9–6·4)	2 (-61·2–188·1)
Côte d'Ivoire	1·2 (0·4–3)	1 (0·3–2·7)	-12·9 (-67·9–178·7)	0·8 (0·2–1·9)	0·7 (0·2–1·6)	-12·6 (-68·1–171·3)	1 (0·3–2·4)	0·8 (0·3–2·2)	-13·3 (-67·7–181·5)
Gambia	1·9 (0·6–4·8)	1·6 (0·5–4·2)	-15·2 (-69·4–169)	1·3 (0·4–3·1)	1·1 (0·4–2·6)	-15·8 (-69·1–153·9)	1·6 (0·5–4)	1·4 (0·4–3·5)	-15 (-67·9–166·2)
Ghana	1·1 (0·3–2·8)	1 (0·3–2·8)	-3 (-66·2–219·4)	1·9 (0·6–4·8)	1·9 (0·7–4·7)	-2·6 (-63·9–194·4)	1·5 (0·5–3·7)	1·5 (0·5–3·6)	-2·5 (-65·3–201·7)
Guinea	1·2 (0·4–2·9)	1 (0·3–2·7)	-9·7 (-66·9–190·3)	1·2 (0·4–2·9)	1·3 (0·4–3·3)	8·8 (-60·5–215·1)	1·2 (0·4–2·9)	1·2 (0·4–3)	-0·8 (-63·7–202·8)
Guinea-Bissau	1·4 (0·4–3·6)	1·2 (0·4–3·3)	-11·8 (-68·7–176·3)	1·1 (0·3–2·7)	0·9 (0·3–2·4)	-15 (-67·1–161·7)	1·3 (0·4–3·2)	1·1 (0·4–2·9)	-13·3 (-67·5–176)
Liberia	1·3 (0·4–3·2)	1·3 (0·4–3·3)	-2·3 (-65·6–205·3)	1·6 (0·5–3·9)	1·6 (0·6–4·1)	5 (-61·5–219·2)	1·4 (0·5–3·6)	1·5 (0·5–3·7)	1·9 (-63·5–214·6)
Mali	1·3 (0·4–3·4)	1·2 (0·4–3)	-12·2 (-67·8–188·9)	2·6 (0·9–6·2)	2·5 (0·9–5·9)	-3·1 (-61–156)	1·9 (0·7–4·8)	1·8 (0·7–4·5)	-5·3 (-62·5–166·6)
Mauritania	2·1 (0·6–5·3)	1·8 (0·6–4·6)	-15·8 (-70·5–166)	1·7 (0·5–3·9)	1·3 (0·5–3·4)	-19·2 (-70·3–147·8)	1·9 (0·6–4·7)	1·6 (0·5–4)	-17·2 (-70·7–155·1)

	Female			Male			Both		
	Age-standardized YLD rate (per 100,000)		Change (%)	Age-standardized YLD rate (per 100,000)		Change (%)	Age-standardized YLD rate (per 100,000)		Change (%)
	1990	2023		1990	2023		1990	2023	
Niger	1·9 (0·6–4·8)	2·1 (0·7–5·2)	6·5 (-61·9–239·1)	4·4 (1·5–10)	4·1 (1·5–9·2)	-7·6 (-64·4–138·8)	3·1 (1·1–7·6)	3 (1·1–7·3)	-3·2 (-63·8–161·8)
Nigeria	0·9 (0·3–2·2)	0·7 (0·2–1·9)	-17·7 (-71–161·7)	0·6 (0·2–1·6)	0·5 (0·2–1·4)	-11 (-68·4–166·2)	0·7 (0·3–2)	0·6 (0·2–1·6)	-13·7 (-71·6–149)
Sao Tome and Principe	0·9 (0·3–2·2)	0·8 (0·3–2·1)	-10·4 (-68·7–178·8)	1·2 (0·4–3)	1·3 (0·5–3·4)	13·8 (-58·1–251·3)	1 (0·3–2·6)	1·1 (0·4–2·7)	4 (-62·9–217·7)
Senegal	2·3 (0·7–5·7)	2·2 (0·7–5·9)	-1·8 (-64·7–206·8)	0·8 (0·2–1·9)	0·6 (0·2–1·6)	-18·7 (-70·4–146·6)	1·6 (0·5–3·8)	1·4 (0·5–3·9)	-7·2 (-65·2–197·6)
Sierra Leone	3 (0·9–7·5)	2·8 (0·9–6·9)	-6·5 (-66–190·1)	2·3 (0·8–5·6)	1·9 (0·7–4·8)	-15·6 (-67·8–147·6)	2·6 (0·8–6·5)	2·4 (0·8–5·8)	-10·6 (-67·1–170·6)
Togo	2·8 (0·9–7)	2·5 (0·8–6·3)	-10·1 (-68–171·4)	1·9 (0·6–4·5)	1·5 (0·5–3·8)	-18·9 (-69·6–146·2)	2·3 (0·8–5·9)	2 (0·7–5·2)	-13·7 (-68·5–163·6)

Appendix Table 2·12: Number of DALYs attributable to chewing tobacco use in 1990 and 2023, among ages 15+

	Female			Male			Both		
	Number of DALYs (thousands)		Change (%)	Number of DALYs (thousands)		Change (%)	Number of DALYs (thousands)		Change (%)
	1990	2023		1990	2023		1990	2023	
Global	1080 (550–1990)	2300 (1220–3790)	113 (10·6–282)	1920 (989–3180)	4180 (2670–6320)	118 (31·4–279)	3000 (1550–5000)	6480 (3960–9910)	116 (22·1–278)
**Central Europe, Eastern Europe, and Central Asia**	10·6 (3·36–24·9)	8·19 (2·54–19·4)	-22·5 (-73·1–152)	21·3 (8·33–46·2)	23·6 (8·26–51·2)	10·6 (-60·1–224)	31·9 (11·7–72·3)	31·8 (10·5–70·8)	-0·4 (-64·4–201)
Central Asia	1·4 (0·469–3·35)	1·5 (0·46–3·52)	6·9 (-63·5–238)	7·93 (3·19–16·5)	9·2 (3·4–19·4)	15·9 (-54·8–207)	9·33 (3·64–19·9)	10·7 (3·83–22·8)	14·6 (-56·1–211)
Armenia	0·0301 (0·00959–0·0742)	0·0217 (0·00683–0·052)	-27·8 (-76·3–127)	0·0475 (0·0177–0·103)	0·0363 (0·0119–0·0833)	-23·6 (-71·7–135)	0·0776 (0·028–0·179)	0·058 (0·0183–0·134)	-25·2 (-73·6–133)
Azerbaijan	0·067 (0·0223–0·154)	0·0689 (0·0199–0·167)	2·9 (-65·2–235)	0·175 (0·0623–0·397)	0·203 (0·0694–0·463)	16·4 (-58·2–246)	0·242 (0·0872–0·568)	0·272 (0·0884–0·63)	12·6 (-59·3–246)
Georgia	0·129 (0·0411–0·309)	0·0832 (0·0266–0·199)	-35·3 (-77–99·7)	0·155 (0·0565–0·347)	0·11 (0·0357–0·245)	-29·2 (-74·1–113)	0·283 (0·0942–0·659)	0·193 (0·0618–0·442)	-32 (-76–110)
Kazakhstan	0·255 (0·0891–0·61)	0·166 (0·0524–0·379)	-35·1 (-77·6–111)	0·703 (0·267–1·52)	0·536 (0·181–1·22)	-23·7 (-72·8–128)	0·958 (0·356–2·13)	0·702 (0·226–1·6)	-26·8 (-74·4–124)
Kyrgyzstan	0·192 (0·0624–0·453)	0·0945 (0·0307–0·222)	-50·8 (-82·8–59·7)	2·48 (1·01–5)	1·9 (0·818–3·66)	-23·3 (-69·5–90·1)	2·67 (1·07–5·47)	1·99 (0·843–3·87)	-25·3 (-70·3–85·9)
Mongolia	0·0359 (0·013–0·078)	0·0313 (0·00956–0·0687)	-12·9 (-70·2–177)	0·0802 (0·0265–0·189)	0·1 (0·0336–0·218)	25·3 (-56·6–300)	0·116 (0·0415–0·276)	0·132 (0·0424–0·284)	13·5 (-60·2–271)
Tajikistan	0·198 (0·0686–0·442)	0·398 (0·11–0·918)	101 (-29·2–558)	0·146 (0·0533–0·339)	0·228 (0·0776–0·53)	55·8 (-45·2–361)	0·344 (0·12–0·778)	0·626 (0·187–1·47)	81·8 (-38·2–444)
Turkmenistan	0·0469 (0·0165–0·112)	0·0706 (0·0211–0·162)	50·7 (-51·5–382)	0·076 (0·029–0·162)	0·132 (0·0447–0·306)	73·2 (-38·2–431)	0·123 (0·0455–0·274)	0·202 (0·0658–0·465)	64·6 (-43·7–416)
Uzbekistan	0·446 (0·153–1·06)	0·562 (0·172–1·28)	26 (-57·4–290)	4·08 (1·6–8·46)	5·96 (2·07–12·8)	46·1 (-44·1–310)	4·52 (1·76–9·52)	6·52 (2·25–14)	44·1 (-45·7–309)
Central Europe	2·76 (0·878–6·56)	2·68 (0·83–6·25)	-2·9 (-65·6–225)	5·43 (2·05–12·3)	6·84 (2·35–15·2)	26 (-54·4–286)	8·19 (2·96–19·1)	9·52 (3·15–21·6)	16·3 (-58·2–268)
Albania	0·0393 (0·0126–0·0891)	0·0488 (0·014–0·109)	24·2 (-55·9–294)	0·108 (0·0398–0·24)	0·157 (0·0503–0·354)	45 (-47·8–320)	0·147 (0·0521–0·343)	0·206 (0·0649–0·466)	39·5 (-50·7–311)
Bosnia and Herzegovina	0·0713 (0·0222–0·163)	0·0898 (0·0281–0·206)	25·9 (-56·5–317)	0·134 (0·0485–0·315)	0·17 (0·0572–0·384)	27·5 (-54·5–297)	0·205 (0·0697–0·49)	0·26 (0·0851–0·588)	27 (-53·9–315)

	Female			Male			Both		
	Number of DALYs (thousands)		Change (%)	Number of DALYs (thousands)		Change (%)	Number of DALYs (thousands)		Change (%)
	1990	2023		1990	2023		1990	2023	
Bulgaria	0·426 (0·133–1)	0·47 (0·149–1·1)	10·3 (-61·2–265)	0·88 (0·304–2·01)	1·01 (0·336–2·24)	14·2 (-58·8–245)	1·31 (0·43–3·02)	1·48 (0·485–3·34)	13 (-59·3–253)
Croatia	0·0853 (0·0269–0·198)	0·049 (0·0154–0·113)	-42·6 (-80·3–89·2)	0·17 (0·0647–0·367)	0·117 (0·0398–0·26)	-31·2 (-75·4–107)	0·256 (0·0956–0·575)	0·166 (0·0552–0·377)	-35 (-77–104)
Czechia	0·289 (0·0908–0·682)	0·179 (0·0568–0·421)	-38·2 (-78–105)	0·468 (0·176–1·04)	0·486 (0·167–1·07)	3·8 (-61·3–224)	0·757 (0·263–1·75)	0·664 (0·226–1·49)	-12·2 (-67·7–181)
Hungary	0·226 (0·0726–0·532)	0·121 (0·0378–0·278)	-46·3 (-80·9–77·6)	0·458 (0·17–0·993)	0·273 (0·0947–0·61)	-40·4 (-78·6–86·6)	0·684 (0·247–1·58)	0·395 (0·133–0·896)	-42·3 (-79·6–84·2)
Montenegro	0·0119 (0·00395–0·0289)	0·0194 (0·00617–0·0442)	62·5 (-45·7–390)	0·0222 (0·00807–0·05)	0·0372 (0·0125–0·0837)	67·7 (-37·9–411)	0·0341 (0·012–0·0802)	0·0565 (0·0185–0·126)	65·8 (-40·7–419)
North Macedonia	0·0419 (0·0139–0·0996)	0·0629 (0·0189–0·147)	50·2 (-47·5–385)	0·0703 (0·0238–0·163)	0·115 (0·0382–0·25)	63·3 (-38·6–407)	0·112 (0·0376–0·262)	0·178 (0·059–0·393)	58·4 (-43·1–398)
Poland	0·702 (0·224–1·65)	0·89 (0·278–2·06)	26·7 (-54·1–332)	1·28 (0·492–2·83)	2·22 (0·776–4·81)	73·2 (-37·5–430)	1·98 (0·736–4·53)	3·11 (1·06–6·92)	56·7 (-43·2–400)
Romania	0·449 (0·143–1·06)	0·354 (0·108–0·826)	-21·1 (-71·5–159)	1·01 (0·38–2·3)	1·36 (0·469–3·04)	34·4 (-50·9–322)	1·46 (0·517–3·39)	1·71 (0·573–3·89)	17·3 (-57·2–274)
Serbia	0·277 (0·0879–0·688)	0·278 (0·0828–0·645)	0·2 (-66·4–228)	0·507 (0·176–1·17)	0·559 (0·189–1·24)	10·1 (-61·9–242)	0·784 (0·273–1·87)	0·836 (0·264–1·85)	6·6 (-63·3–223)
Slovakia	0·0657 (0·0212–0·164)	0·0439 (0·014–0·102)	-33·3 (-77·3–107)	0·168 (0·0645–0·364)	0·144 (0·0525–0·321)	-14·3 (-69·1–167)	0·234 (0·086–0·534)	0·188 (0·0663–0·425)	-19·6 (-71·8–160)
Slovenia	0·0282 (0·00884–0·0673)	0·033 (0·0105–0·0779)	17·1 (-59·4–274)	0·0671 (0·0246–0·147)	0·105 (0·0372–0·23)	56·1 (-41·1–389)	0·0953 (0·0349–0·222)	0·138 (0·0475–0·307)	44·6 (-46·4–349)
Eastern Europe	6·41 (2·02–15·2)	4·02 (1·25–9·63)	-37·4 (-78·2–104)	7·95 (3·06–17·5)	7·53 (2·58–16·5)	-5·4 (-65·9–196)	14·4 (5·05–33·3)	11·5 (3·68–25·9)	-19·6 (-71·1–160)
Belarus	0·191 (0·0622–0·452)	0·141 (0·0455–0·33)	-26·2 (-75–143)	0·343 (0·129–0·76)	0·458 (0·165–1·02)	33·4 (-51·2–335)	0·535 (0·19–1·25)	0·599 (0·206–1·35)	12 (-59·6–270)
Estonia	0·0335 (0·0108–0·0799)	0·0151 (0·00461–0·035)	-54·8 (-84·5–50·8)	0·0458 (0·017–0·1)	0·0792 (0·0288–0·173)	72·8 (-33·1–425)	0·0793 (0·0284–0·182)	0·0944 (0·0336–0·209)	19 (-54·6–274)
Latvia	0·0655 (0·021–0·157)	0·0407 (0·0126–0·0951)	-37·8 (-78·6–98·1)	0·0718 (0·0268–0·161)	0·0559 (0·0191–0·125)	-22·2 (-72·6–140)	0·137 (0·0476–0·326)	0·0966 (0·031–0·22)	-29·6 (-75·3–123)
Lithuania	0·0453 (0·0144–0·109)	0·0367 (0·0114–0·0856)	-18·9 (-72–161)	0·0711 (0·0265–0·155)	0·076 (0·0265–0·17)	6·9 (-60·4–241)	0·116 (0·0422–0·266)	0·113 (0·0376–0·258)	-3·1 (-65·1–210)

	Female			Male			Both		
	Number of DALYs (thousands)		Change (%)	Number of DALYs (thousands)		Change (%)	Number of DALYs (thousands)		Change (%)
	1990	2023		1990	2023		1990	2023	
Republic of Moldova	0·0697 (0·0217–0·161)	0·0396 (0·012–0·0935)	-43·2 (-80·4–91)	0·155 (0·0594–0·343)	0·166 (0·0547–0·361)	7·3 (-61·8–227)	0·225 (0·0838–0·508)	0·206 (0·067–0·455)	-8·4 (-68·1–188)
Russian Federation	4·71 (1·48–11)	3·17 (0·985–7·67)	-32·6 (-76·2–116)	5·28 (2·05–11·5)	5·16 (1·75–11·4)	-2·2 (-64·7–206)	9·98 (3·51–23)	8·33 (2·6–18·8)	-16·5 (-69·7–171)
Ukraine	1·3 (0·409–3·03)	0·571 (0·175–1·33)	-56 (-85·2–45·5)	1·99 (0·733–4·46)	1·53 (0·52–3·44)	-23·1 (-72·6–133)	3·29 (1·12–7·57)	2·1 (0·697–4·71)	-36·1 (-77·3–99·2)
**High-income**	13·5 (4·46–31·5)	15·2 (4·83–35·4)	13·1 (-60·1–264)	92·9 (55·6–148)	101 (62·6–171)	8·4 (-38·1–96·2)	106 (62·3–176)	116 (72·4–199)	9 (-40·4–110)
Australasia	0·205 (0·0654–0·496)	0·24 (0·0747–0·556)	17·1 (-58·9–280)	0·83 (0·308–1·81)	1·04 (0·376–2·32)	25·7 (-53·3–277)	1·03 (0·38–2·3)	1·28 (0·446–2·9)	24 (-54·5–278)
Australia	0·166 (0·0531–0·4)	0·19 (0·0597–0·445)	14·6 (-59·5–269)	0·707 (0·262–1·53)	0·887 (0·318–1·97)	25·6 (-53·4–273)	0·873 (0·321–1·93)	1·08 (0·375–2·43)	23·5 (-54·6–274)
New Zealand	0·0385 (0·0123–0·092)	0·0494 (0·0154–0·115)	28·2 (-56·2–316)	0·123 (0·0459–0·273)	0·156 (0·0565–0·351)	26·4 (-53·2–292)	0·162 (0·0598–0·365)	0·205 (0·0711–0·467)	26·8 (-53·9–304)
High-income Asia Pacific	2·98 (0·947–7·3)	3·4 (1·11–7·95)	14·1 (-58·9–272)	11·4 (4·16–26·2)	13·7 (4·7–30·8)	20 (-56·5–259)	14·4 (5·2–33·7)	17·1 (5·67–39·1)	18·7 (-57–265)
Brunei Darussalam	0·0085 (0·00296–0·0208)	0·0177 (0·00573–0·0414)	109 (-30·5–575)	0·0149 (0·00531–0·0365)	0·0325 (0·0112–0·0737)	118 (-24·5–592)	0·0234 (0·00817–0·0573)	0·0502 (0·0171–0·114)	115 (-26·4–559)
Japan	1·96 (0·613–4·8)	2·68 (0·874–6·22)	36·8 (-50·1–356)	7·95 (2·94–17·9)	10·7 (3·71–24·3)	35·2 (-50·8–308)	9·91 (3·62–22·7)	13·4 (4·48–30·8)	35·5 (-50·7–319)
Republic of Korea	0·969 (0·306–2·52)	0·642 (0·212–1·54)	-33·7 (-76–109)	3·26 (1·14–7·74)	2·64 (0·865–5·67)	-19 (-70·8–137)	4·23 (1·43–10·2)	3·29 (1·08–7·2)	-22·3 (-72·2–138)
Singapore	0·0405 (0·0134–0·0945)	0·0563 (0·0174–0·134)	38·9 (-51·2–347)	0·176 (0·0668–0·387)	0·259 (0·0942–0·597)	47·1 (-43·4–341)	0·217 (0·0812–0·486)	0·316 (0·111–0·73)	45·6 (-45·1–339)
High-income North America	6·31 (2·17–14·1)	7·99 (2·53–18·7)	26·6 (-56·4–293)	72·7 (47·9–110)	77·1 (47·8–120)	6 (-33·4–70·3)	79 (50·6–120)	85·1 (52·1–135)	7·7 (-33·4–81·3)
Canada	0·55 (0·183–1·32)	0·829 (0·263–1·91)	50·7 (-47·6–377)	4·55 (1·81–8·98)	3·88 (1·39–8·43)	-14·6 (-66·7–145)	5·1 (2·01–10·3)	4·71 (1·67–10·4)	-7·6 (-64·7–170)
Greenland	0·00187 (0·000648–0·00423)	0·00152 (0·000496–0·00338)	-18·7 (-72·2–179)	0·0221 (0·0085–0·0482)	0·0195 (0·00728–0·0424)	-11·5 (-67·2–169)	0·0239 (0·0092–0·052)	0·021 (0·00787–0·0459)	-12·1 (-67·3–164)
United States of America	5·76 (1·99–12·8)	7·16 (2·27–16·8)	24·3 (-56·7–281)	68·1 (45·5–103)	73·2 (45·2–114)	7·4 (-31·9–69·8)	73·9 (48·1–112)	80·3 (49·3–127)	8·8 (-31·9–79·9)

Return to Table of Contents

	Female			Male			Both		
	Number of DALYs (thousands)		Change (%)	Number of DALYs (thousands)		Change (%)	Number of DALYs (thousands)		Change (%)
	1990	2023		1990	2023		1990	2023	
Southern Latin America	0·487 (0·157–1·17)	0·401 (0·124–0·922)	-17·5 (-71·1–171)	0·774 (0·29–1·74)	0·578 (0·201–1·29)	-25·3 (-73·4–133)	1·26 (0·462–2·9)	0·98 (0·315–2·23)	-22·3 (-72·5–150)
Argentina	0·326 (0·106–0·782)	0·255 (0·0792–0·584)	-21·8 (-72·1–157)	0·475 (0·177–1·07)	0·359 (0·126–0·796)	-24·4 (-72·9–136)	0·801 (0·291–1·85)	0·614 (0·201–1·39)	-23·3 (-73–146)
Chile	0·112 (0·0365–0·269)	0·11 (0·0343–0·261)	-1·5 (-65·8–220)	0·222 (0·0841–0·493)	0·169 (0·0567–0·38)	-24 (-72·7–136)	0·334 (0·121–0·764)	0·279 (0·0901–0·633)	-16·5 (-70·3–163)
Uruguay	0·0484 (0·0155–0·114)	0·0358 (0·0106–0·0836)	-26·1 (-74·5–134)	0·0772 (0·0294–0·163)	0·0505 (0·0176–0·111)	-34·6 (-76·2–108)	0·126 (0·0466–0·286)	0·0863 (0·0284–0·196)	-31·3 (-75·4–117)
Western Europe	3·47 (1·11–8·47)	3·19 (1–7·42)	-8·3 (-67·2–208)	7·2 (2·68–15·6)	8·33 (2·94–18·8)	15·6 (-58·6–249)	10·7 (3·89–24·4)	11·5 (3·93–26·5)	7·9 (-61·8–236)
Andorra	0·000171 (5·29e-05–0·000422)	0·000371 (0·000114–0·000898)	117 (-29·3–570)	0·000639 (0·000228–0·0014)	0·00161 (0·000535–0·00345)	152 (-13–684)	0·00081 (0·00029–0·00185)	0·00198 (0·00066–0·00438)	145 (-13·9–643)
Austria	0·0756 (0·0241–0·184)	0·0683 (0·0216–0·157)	-9·7 (-68·2–198)	0·153 (0·0564–0·337)	0·238 (0·0838–0·528)	55·4 (-44–367)	0·229 (0·0833–0·532)	0·306 (0·105–0·696)	33·9 (-51·7–314)
Belgium	0·0867 (0·0278–0·211)	0·0606 (0·0192–0·142)	-30 (-75·2–131)	0·146 (0·0536–0·318)	0·122 (0·0434–0·282)	-16·2 (-70·6–161)	0·233 (0·084–0·539)	0·183 (0·0624–0·429)	-21·4 (-72·3–152)
Cyprus	0·00612 (0·00206–0·0149)	0·00483 (0·00142–0·0108)	-21·1 (-74·7–151)	0·0103 (0·00317–0·0247)	0·0109 (0·00358–0·0255)	6·5 (-63·1–247)	0·0164 (0·00535–0·0399)	0·0157 (0·00497–0·0357)	-3·8 (-67·2–192)
Denmark	0·0626 (0·0201–0·151)	0·0771 (0·0241–0·181)	23 (-55·7–298)	0·178 (0·067–0·399)	0·25 (0·0903–0·548)	40·1 (-48·7–304)	0·241 (0·0895–0·547)	0·327 (0·114–0·733)	35·6 (-50·5–302)
Finland	0·0436 (0·0138–0·106)	0·0414 (0·0129–0·0979)	-5 (-65·7–203)	0·062 (0·023–0·143)	0·107 (0·0384–0·238)	72·5 (-36·9–419)	0·106 (0·037–0·25)	0·148 (0·0498–0·339)	40·5 (-48·7–330)
France	0·363 (0·118–0·868)	0·357 (0·113–0·847)	-1·8 (-65·6–227)	1·2 (0·442–2·57)	0·867 (0·301–1·97)	-27·9 (-74·9–123)	1·56 (0·567–3·25)	1·22 (0·416–2·83)	-21·8 (-73–145)
Germany	0·838 (0·267–2·06)	0·888 (0·279–2·03)	6 (-62·3–254)	1·4 (0·519–3·02)	2·4 (0·83–5·44)	72·1 (-38·5–438)	2·23 (0·818–5·17)	3·29 (1·1–7·63)	47·3 (-47·5–370)
Greece	0·135 (0·0423–0·324)	0·0943 (0·03–0·218)	-30·1 (-75·7–135)	0·177 (0·0637–0·407)	0·152 (0·0509–0·356)	-14·3 (-69·6–161)	0·312 (0·103–0·731)	0·246 (0·0809–0·575)	-21·1 (-72·1–152)
Iceland	0·00202 (0·000661–0·00488)	0·00335 (0·00106–0·00774)	65·9 (-42·1–470)	0·0074 (0·00274–0·0165)	0·0212 (0·00811–0·0468)	186 (8·2–751)	0·00942 (0·00346–0·0215)	0·0245 (0·00914–0·0544)	161 (-3·4–695)
Ireland	0·0251 (0·00818–0·0592)	0·0222 (0·00707–0·0531)	-11·8 (-68–199)	0·0395 (0·0144–0·0883)	0·0458 (0·0165–0·102)	15·9 (-57·2–249)	0·0646 (0·0234–0·149)	0·0679 (0·0232–0·155)	5·1 (-62–229)

Return to Table of Contents

	Female			Male			Both		
	Number of DALYs (thousands)		Change (%)	Number of DALYs (thousands)		Change (%)	Number of DALYs (thousands)		Change (%)
	1990	2023		1990	2023		1990	2023	
Israel	0·0214 (0·00671–0·0514)	0·0287 (0·0092–0·0674)	34·1 (-53·3–339)	0·0405 (0·0146–0·0926)	0·0658 (0·0226–0·153)	62·4 (-40–389)	0·0619 (0·0219–0·143)	0·0945 (0·0318–0·222)	52·6 (-44·3–381)
Italy	0·467 (0·146–1·12)	0·393 (0·126–0·928)	-15·8 (-70·2–181)	1·04 (0·387–2·31)	0·936 (0·33–2·09)	-10·2 (-67·4–169)	1·51 (0·546–3·48)	1·33 (0·453–3·06)	-11·9 (-68·2–177)
Luxembourg	0·00445 (0·00144–0·0107)	0·00255 (0·000792–0·00601)	-42·6 (-80–85·8)	0·00686 (0·0025–0·0154)	0·00577 (0·00214–0·0131)	-15·8 (-68·8–151)	0·0113 (0·00407–0·0261)	0·00833 (0·00295–0·0192)	-26·4 (-73·7–127)
Malta	0·00254 (0·000797–0·00596)	0·00216 (0·000694–0·00509)	-15·2 (-69·6–181)	0·00418 (0·00152–0·00937)	0·00441 (0·00156–0·0101)	5·7 (-60·3–231)	0·00672 (0·00241–0·0153)	0·00657 (0·00223–0·0153)	-2·2 (-63·6–215)
Monaco	0·000664 (0·000212–0·00153)	0·000402 (0·000123–0·00103)	-39·4 (-81·4–104)	0·00116 (0·000433–0·00277)	0·0011 (0·000397–0·00228)	-4·8 (-70·7–201)	0·00182 (0·000634–0·00436)	0·00151 (0·000527–0·00335)	-17·4 (-73·6–153)
Netherlands	0·0989 (0·0319–0·24)	0·11 (0·0346–0·259)	11·5 (-60·6–267)	0·146 (0·0548–0·326)	0·207 (0·0735–0·471)	41·6 (-48·3–323)	0·245 (0·0894–0·568)	0·317 (0·108–0·736)	29·4 (-53–301)
Norway	0·0602 (0·0189–0·145)	0·0665 (0·0208–0·156)	10·5 (-60·2–272)	0·169 (0·0621–0·383)	0·37 (0·14–0·836)	119 (-17·1–548)	0·229 (0·0828–0·534)	0·436 (0·159–0·993)	90·4 (-27·9–480)
Portugal	0·165 (0·0515–0·395)	0·0828 (0·0263–0·189)	-50 (-82·2–62·9)	0·252 (0·095–0·579)	0·193 (0·0674–0·435)	-23·7 (-72·1–142)	0·418 (0·145–0·975)	0·275 (0·0925–0·633)	-34·1 (-76·2–111)
San Marino	0·000163 (5·49e-05–0·000398)	0·000223 (6·78e-05–0·000523)	36·5 (-55·8–359)	0·000374 (0·000128–0·000906)	0·000468 (0·000161–0·00112)	25·1 (-55·9–283)	0·000537 (0·000181–0·00132)	0·000691 (0·000236–0·00161)	28·6 (-55·6–299)
Spain	0·313 (0·0987–0·767)	0·234 (0·0755–0·546)	-25·5 (-73·1–148)	0·664 (0·245–1·38)	0·504 (0·174–1·15)	-24 (-73·3–137)	0·977 (0·356–2·18)	0·738 (0·246–1·71)	-24·5 (-73·6–141)
Sweden	0·081 (0·0259–0·198)	0·165 (0·0518–0·38)	103 (-26·9–560)	0·627 (0·235–1·37)	0·842 (0·317–1·85)	34·4 (-49·3–287)	0·708 (0·264–1·57)	1·01 (0·366–2·25)	42·3 (-46·7–313)
Switzerland	0·0507 (0·0162–0·124)	0·0451 (0·0144–0·106)	-11·1 (-67·8–198)	0·107 (0·0397–0·232)	0·113 (0·0406–0·255)	5·3 (-62·8–212)	0·158 (0·0571–0·354)	0·158 (0·0548–0·365)	0·1 (-64·2–209)
United Kingdom	0·568 (0·186–1·35)	0·436 (0·135–1·01)	-23·1 (-72·6–156)	0·766 (0·289–1·69)	0·863 (0·3–1·96)	12·8 (-58·7–247)	1·33 (0·493–3·06)	1·3 (0·44–3)	-2·5 (-64·4–207)
**Latin America and Caribbean**	5·96 (1·96–14)	8·81 (2·8–20·5)	47·9 (-48·2–383)	10·3 (3·91–22·9)	18·6 (6·57–40)	80·2 (-35·4–457)	16·3 (5·93–37·3)	27·4 (8·99–60·7)	68·4 (-40·2–431)
Andean Latin America	0·271 (0·0873–0·654)	0·364 (0·114–0·844)	34·5 (-56·1–338)	0·355 (0·121–0·872)	0·602 (0·181–1·34)	69·2 (-39–445)	0·626 (0·208–1·55)	0·966 (0·292–2·18)	54·2 (-47·5–388)
Bolivia (Plurinational State of)	0·0562 (0·018–0·126)	0·0846 (0·0284–0·192)	50·6 (-49·7–415)	0·0982 (0·0274–0·252)	0·192 (0·0672–0·461)	95·4 (-43–588)	0·154 (0·0469–0·389)	0·276 (0·0912–0·651)	79·1 (-42·4–504)

	Female			Male			Both		
	Number of DALYs (thousands)		Change (%)	Number of DALYs (thousands)		Change (%)	Number of DALYs (thousands)		Change (%)
	1990	2023		1990	2023		1990	2023	
Ecuador	0·0458 (0·0147–0·11)	0·0637 (0·0195–0·147)	39 (-50·4–362)	0·0559 (0·0192–0·127)	0·077 (0·0254–0·171)	37·9 (-48·9–330)	0·102 (0·033–0·238)	0·141 (0·0453–0·322)	38·4 (-49·5–342)
Peru	0·169 (0·0552–0·413)	0·216 (0·0635–0·485)	27·9 (-62·1–314)	0·201 (0·0691–0·483)	0·333 (0·0946–0·726)	65·2 (-42·8–414)	0·37 (0·127–0·887)	0·549 (0·158–1·23)	48·2 (-50·5–365)
Caribbean	0·6 (0·202–1·35)	1·1 (0·346–2·55)	83·9 (-40·1–452)	0·887 (0·328–2·07)	1·84 (0·654–4·15)	108 (-23·5–526)	1·49 (0·503–3·41)	2·95 (1·02–6·59)	98·2 (-28·6–505)
Antigua and Barbuda	0·000788 (0·00025–0·00186)	0·000788 (0·000234–0·00184)	0 (-64·8–230)	0·00129 (0·000496–0·00292)	0·00167 (0·000567–0·00374)	29·9 (-54·8–285)	0·00208 (0·000733–0·00481)	0·00246 (0·000782–0·00558)	18·5 (-58·2–267)
Bahamas	0·00242 (0·000808–0·00555)	0·00431 (0·00122–0·00985)	78·3 (-35·4–502)	0·00625 (0·00238–0·0131)	0·0124 (0·00449–0·0268)	97·7 (-26·9–535)	0·00867 (0·00326–0·019)	0·0167 (0·00546–0·0366)	92·3 (-29·2–520)
Barbados	0·00513 (0·0017–0·0121)	0·00515 (0·00147–0·0116)	0·5 (-66·3–220)	0·00361 (0·00139–0·00778)	0·00544 (0·00179–0·012)	50·6 (-44·8–362)	0·00874 (0·00311–0·0202)	0·0106 (0·00331–0·0236)	21·2 (-54·7–284)
Belize	0·000945 (3e-04–0·00224)	0·00205 (0·000611–0·00479)	116 (-25·4–625)	0·00134 (0·000479–0·00303)	0·00357 (0·0012–0·008)	166 (-5·8–694)	0·00229 (0·000767–0·00528)	0·00562 (0·0018–0·013)	146 (-15·4–666)
Bermuda	0·000558 (0·000193–0·00134)	0·000567 (0·000177–0·00136)	1·6 (-63·7–233)	0·00141 (0·000556–0·00289)	0·00145 (0·000517–0·00309)	3·1 (-63·5–220)	0·00196 (0·000752–0·0042)	0·00202 (0·000698–0·00444)	2·7 (-63·3–225)
Cuba	0·115 (0·0381–0·267)	0·132 (0·0421–0·302)	15·4 (-58·3–272)	0·175 (0·0649–0·377)	0·311 (0·116–0·724)	77·5 (-34·8–451)	0·29 (0·106–0·653)	0·443 (0·162–1·05)	52·9 (-43·8–391)
Dominica	0·000678 (0·000216–0·00161)	0·000643 (0·000198–0·00157)	-5·2 (-67·7–226)	0·000817 (0·00031–0·00186)	0·00115 (0·000436–0·00259)	40·6 (-47·1–335)	0·0015 (0·000519–0·00354)	0·00179 (0·000631–0·00431)	19·8 (-55·7–286)
Dominican Republic	0·0795 (0·0255–0·192)	0·131 (0·0397–0·287)	64·7 (-39·6–419)	0·206 (0·0755–0·428)	0·506 (0·174–1·15)	146 (-1·9–633)	0·286 (0·102–0·62)	0·637 (0·212–1·43)	123 (-11·3–570)
Grenada	0·00194 (0·00063–0·00436)	0·00175 (0·000518–0·00419)	-9·6 (-66·2–193)	0·00338 (0·0013–0·00744)	0·00455 (0·00167–0·0102)	34·8 (-49·5–298)	0·00532 (0·00194–0·0122)	0·00631 (0·00223–0·0142)	18·6 (-55–262)
Guyana	0·0108 (0·00331–0·0248)	0·00852 (0·00257–0·0198)	-21·1 (-73·5–159)	0·0171 (0·00565–0·0377)	0·0151 (0·00506–0·0355)	-11·7 (-68·6–155)	0·0279 (0·00912–0·0632)	0·0236 (0·00759–0·0554)	-15·4 (-68·8–148)
Haiti	0·281 (0·0947–0·627)	0·696 (0·22–1·62)	147 (-20·7–694)	0·306 (0·0958–0·753)	0·76 (0·246–1·68)	148 (-14·8–709)	0·587 (0·202–1·31)	1·46 (0·508–3·2)	148 (-14–688)
Jamaica	0·0276 (0·00888–0·0668)	0·0313 (0·00865–0·0707)	13·3 (-59·6–276)	0·0335 (0·0118–0·0773)	0·0587 (0·0203–0·135)	75·1 (-37·3–430)	0·0611 (0·02–0·143)	0·0899 (0·0287–0·211)	47·2 (-48–371)
Puerto Rico	0·0274 (0·00953–0·0653)	0·0224 (0·00723–0·0524)	-18·3 (-72·3–163)	0·0604 (0·0219–0·127)	0·0413 (0·0147–0·0919)	-31·7 (-75·7–105)	0·0878 (0·0313–0·192)	0·0636 (0·022–0·144)	-27·5 (-74·8–122)

	Female			Male			Both		
	Number of DALYs (thousands)		Change (%)	Number of DALYs (thousands)		Change (%)	Number of DALYs (thousands)		Change (%)
	1990	2023		1990	2023		1990	2023	
Saint Kitts and Nevis	0·000793 (0·000251–0·00184)	0·000496 (0·000147–0·00116)	-37·4 (-77·8–110)	0·000767 (0·00028–0·0017)	0·000691 (0·000228–0·00151)	-9·9 (-67·5–177)	0·00156 (0·000506–0·00359)	0·00119 (0·000362–0·00274)	-23·9 (-72–141)
Saint Lucia	0·00196 (0·000654–0·00478)	0·00176 (0·00053–0·00406)	-10·2 (-70–209)	0·00294 (0·00112–0·00602)	0·00431 (0·00149–0·00995)	46·3 (-53·1–350)	0·0049 (0·00183–0·0111)	0·00607 (0·00198–0·0141)	23·7 (-59·6–298)
Saint Vincent and the Grenadines	0·000975 (0·000315–0·00227)	0·000969 (0·00029–0·00216)	-0·6 (-64·9–212)	0·00231 (0·000892–0·00476)	0·00419 (0·0014–0·00954)	81·5 (-39·8–460)	0·00328 (0·00123–0·0072)	0·00515 (0·00161–0·0117)	57·1 (-47–399)
Suriname	0·00396 (0·00125–0·0097)	0·0074 (0·00223–0·0182)	86·6 (-38·1–504)	0·00654 (0·00238–0·0147)	0·0123 (0·00397–0·0279)	88 (-31·8–428)	0·0105 (0·00373–0·0244)	0·0197 (0·00616–0·0462)	87·5 (-34·2–458)
Trinidad and Tobago	0·018 (0·00566–0·0427)	0·0198 (0·00602–0·0459)	10·1 (-61·8–238)	0·0269 (0·0097–0·0596)	0·0371 (0·013–0·0833)	38·1 (-48–341)	0·0449 (0·0154–0·103)	0·0569 (0·0182–0·129)	26·9 (-54·7–312)
United States Virgin Islands	0·000638 (0·000207–0·00145)	0·000591 (0·000182–0·00136)	-7·3 (-67·1–193)	0·00114 (0·000439–0·00247)	0·00151 (0·000553–0·00334)	32·5 (-51–318)	0·00178 (0·000645–0·00399)	0·0021 (0·000765–0·00463)	18·2 (-56·1–270)
Central Latin America	1·4 (0·451–3·35)	2·29 (0·692–5·51)	63·5 (-43·2–432)	2·2 (0·835–4·91)	4·32 (1·41–9·33)	96·1 (-31·4–512)	3·61 (1·31–8·32)	6·62 (2·05–14·8)	83·4 (-37·2–489)
Colombia	0·394 (0·128–0·922)	0·443 (0·139–1·02)	12·6 (-60·4–271)	0·446 (0·166–0·995)	0·54 (0·18–1·2)	21·1 (-57·1–263)	0·839 (0·303–1·92)	0·983 (0·319–2·24)	17·1 (-58·9–267)
Costa Rica	0·0113 (0·00363–0·0271)	0·0243 (0·00788–0·0572)	114 (-26–592)	0·0178 (0·0065–0·0403)	0·0413 (0·0143–0·0893)	132 (-15·4–578)	0·0291 (0·0104–0·0676)	0·0656 (0·0222–0·147)	125 (-20·1–584)
El Salvador	0·0574 (0·0189–0·142)	0·0635 (0·0191–0·146)	10·5 (-61·2–269)	0·0791 (0·0278–0·183)	0·0744 (0·0243–0·162)	-5·9 (-64–190)	0·137 (0·0476–0·325)	0·138 (0·0445–0·313)	1 (-63·9–211)
Guatemala	0·0508 (0·0171–0·12)	0·0935 (0·028–0·216)	84·2 (-38·4–530)	0·05 (0·019–0·112)	0·0874 (0·028–0·201)	74·6 (-35·7–444)	0·101 (0·0365–0·233)	0·181 (0·0562–0·414)	79·5 (-36·6–485)
Honduras	0·0285 (0·00903–0·0615)	0·0737 (0·0237–0·18)	159 (-13·5–742)	0·0497 (0·0175–0·12)	0·119 (0·0402–0·302)	139 (-25·3–687)	0·0781 (0·0267–0·186)	0·193 (0·0663–0·45)	146 (-18·1–640)
Mexico	0·412 (0·133–0·984)	0·579 (0·177–1·36)	40·4 (-52·1–365)	0·472 (0·175–1·07)	0·819 (0·285–1·77)	73·3 (-34·3–428)	0·885 (0·316–2·09)	1·4 (0·463–3·13)	58 (-43·1–408)
Nicaragua	0·0297 (0·00996–0·0705)	0·0431 (0·0137–0·107)	45·3 (-48·7–351)	0·0347 (0·012–0·0825)	0·0572 (0·0189–0·125)	64·8 (-39·7–415)	0·0644 (0·0214–0·153)	0·1 (0·0328–0·238)	55·8 (-44·1–403)
Panama	0·0259 (0·0084–0·0616)	0·0483 (0·0148–0·109)	86·2 (-33·3–514)	0·0322 (0·0117–0·0744)	0·0614 (0·0211–0·139)	90·6 (-28·4–478)	0·0582 (0·02–0·136)	0·11 (0·0358–0·246)	88·6 (-31·7–496)
Venezuela (Bolivarian Republic of)	0·394 (0·13–0·938)	0·926 (0·257–2·32)	135 (-18·8–673)	1·02 (0·391–2·23)	2·52 (0·809–5·52)	147 (-12·4–660)	1·42 (0·531–3·18)	3·45 (1·03–7·6)	144 (-17·1–684)

	Female			Male			Both		
	Number of DALYs (thousands)		Change (%)	Number of DALYs (thousands)		Change (%)	Number of DALYs (thousands)		Change (%)
	1990	2023		1990	2023		1990	2023	
Tropical Latin America	3·68 (1·2–8·68)	5·05 (1·62–11·6)	37·1 (-50·1–357)	6·86 (2·61–15)	11·8 (4·22–25·5)	72·1 (-37·3–425)	10·5 (3·89–24)	16·8 (5·76–37·1)	59·8 (-41·6–404)
Brazil	3·52 (1·14–8·29)	4·8 (1·54–11)	36·5 (-50–358)	6·53 (2·48–14·3)	11·1 (3·95–23·9)	69·5 (-38·1–418)	10 (3·71–22·8)	15·9 (5·41–34·9)	57·9 (-42·1–398)
Paraguay	0·163 (0·057–0·381)	0·245 (0·0769–0·539)	50·2 (-47·7–388)	0·329 (0·124–0·747)	0·734 (0·266–1·57)	123 (-20·1–554)	0·492 (0·179–1·14)	0·978 (0·347–2·11)	98·9 (-30·3–501)
North Africa and Middle East	14·1 (5·21–28·8)	14·9 (7·57–30·1)	6·4 (-50·1–136)	37·7 (16·4–79·4)	44·9 (23–85·5)	19 (-39·2–161)	51·8 (21·6–106)	59·8 (30·5–112)	15·5 (-39·3–147)
Afghanistan	0·521 (0·165–1·19)	0·561 (0·185–1·33)	7·6 (-66·1–244)	1·31 (0·428–3·17)	2·18 (0·768–5·22)	66·4 (-46·1–468)	1·83 (0·588–4·39)	2·74 (1·01–6·31)	49·7 (-50·4–405)
Algeria	0·748 (0·244–1·74)	1·01 (0·316–2·4)	35·3 (-56·8–303)	3·81 (1·32–8·81)	6·35 (2·16–14·5)	66·9 (-41·9–426)	4·55 (1·57–10·6)	7·36 (2·56–16·8)	61·7 (-43·6–387)
Bahrain	0·00587 (0·00195–0·0143)	0·00671 (0·00199–0·0166)	14·4 (-61·6–258)	0·017 (0·00587–0·0393)	0·0372 (0·0124–0·0831)	119 (-26·1–544)	0·0229 (0·00786–0·0533)	0·044 (0·0143–0·0975)	91·9 (-34·8–484)
Egypt	0·665 (0·201–1·64)	0·905 (0·286–2·17)	36 (-60·9–325)	2·59 (0·828–6·27)	3·79 (1·23–9·07)	46·3 (-53·7–392)	3·25 (1·06–7·65)	4·69 (1·55–10·9)	44·2 (-51·6–364)
Iran (Islamic Republic of)	1·07 (0·358–2·51)	0·814 (0·274–2·08)	-23·8 (-74–128)	2·5 (0·8–6·11)	2·17 (0·697–4·89)	-13·1 (-72·4–180)	3·57 (1·17–8·62)	2·98 (0·954–6·88)	-16·3 (-73–172)
Iraq	0·268 (0·089–0·656)	0·461 (0·146–1·1)	71·8 (-45·7–445)	0·482 (0·153–1·19)	1·07 (0·351–2·88)	123 (-32·3–726)	0·75 (0·244–1·81)	1·53 (0·51–3·9)	104 (-35·1–613)
Jordan	0·0285 (0·00917–0·0696)	0·0533 (0·0163–0·126)	86·8 (-37·4–508)	0·0652 (0·0187–0·161)	0·11 (0·0356–0·258)	69 (-51·9–481)	0·0937 (0·0298–0·233)	0·163 (0·0522–0·387)	74·4 (-45·4–476)
Kuwait	0·00607 (0·00207–0·0139)	0·0119 (0·00364–0·0284)	95·6 (-32·3–534)	0·0189 (0·00703–0·0425)	0·0504 (0·017–0·114)	166 (-4·2–665)	0·025 (0·00911–0·0572)	0·0623 (0·0207–0·142)	149 (-11·1–645)
Lebanon	0·038 (0·0114–0·0928)	0·0515 (0·016–0·118)	35·5 (-57·3–358)	0·0854 (0·0247–0·212)	0·131 (0·043–0·297)	52·8 (-55·2–445)	0·123 (0·0382–0·304)	0·182 (0·0616–0·417)	47·5 (-52·9–425)
Libya	0·0565 (0·0183–0·129)	0·111 (0·0343–0·25)	96 (-42·4–576)	0·154 (0·0508–0·371)	0·251 (0·0798–0·573)	62·8 (-48·8–402)	0·211 (0·0702–0·497)	0·362 (0·124–0·838)	71·7 (-42·9–438)
Morocco	0·642 (0·197–1·52)	0·918 (0·282–2·15)	43 (-52·1–351)	0·8 (0·234–1·99)	1·13 (0·339–2·71)	41·5 (-54·6–343)	1·44 (0·451–3·36)	2·05 (0·682–4·69)	42·2 (-50·9–343)
Oman	0·0223 (0·0073–0·0535)	0·0315 (0·00997–0·0745)	41·1 (-56·4–329)	0·0707 (0·022–0·17)	0·0957 (0·0297–0·225)	35·3 (-54–376)	0·093 (0·0295–0·227)	0·127 (0·0403–0·291)	36·7 (-52–341)

	Female			Male			Both		
	Number of DALYs (thousands)		Change (%)	Number of DALYs (thousands)		Change (%)	Number of DALYs (thousands)		Change (%)
	1990	2023		1990	2023		1990	2023	
Palestine	0·0393 (0·0128–0·0989)	0·0335 (0·0102–0·0816)	-14·7 (-72·5–179)	0·102 (0·0298–0·244)	0·0877 (0·0294–0·204)	-13·9 (-71·4–197)	0·141 (0·0445–0·347)	0·121 (0·0401–0·283)	-14·1 (-71·7–175)
Qatar	0·00129 (0·000431–0·003)	0·00341 (0·00108–0·0083)	163 (-14·8–678)	0·00755 (0·00229–0·0183)	0·0252 (0·00879–0·0604)	234 (11·7–962)	0·00885 (0·00286–0·0215)	0·0286 (0·00974–0·0673)	224 (8·4–881)
Saudi Arabia	0·19 (0·0646–0·448)	0·243 (0·0685–0·597)	28·1 (-55·7–326)	0·378 (0·116–0·906)	0·452 (0·142–1·01)	19·5 (-63·7–301)	0·568 (0·188–1·36)	0·695 (0·219–1·61)	22·4 (-60·7–304)
Sudan	0·568 (0·174–1·31)	0·64 (0·206–1·64)	12·7 (-63·1–280)	4·75 (1·43–11·6)	6·37 (2·06–15·6)	34 (-52–298)	5·32 (1·65–12·6)	7·01 (2·27–16·7)	31·7 (-53–289)
Syrian Arab Republic	0·0963 (0·0305–0·244)	0·108 (0·031–0·28)	12·4 (-65·6–278)	0·143 (0·0426–0·353)	0·174 (0·0546–0·387)	21·9 (-59·3–353)	0·239 (0·0775–0·585)	0·282 (0·0899–0·656)	18·1 (-63·1–309)
Tunisia	0·622 (0·205–1·35)	0·628 (0·194–1·37)	1 (-65·7–215)	1·3 (0·428–2·87)	1·42 (0·496–3·26)	9·8 (-62·6–257)	1·92 (0·647–4·45)	2·05 (0·724–4·66)	7 (-61·4–216)
Türkiye	1·6 (0·542–3·77)	1·38 (0·392–3·44)	-13·7 (-73·8–191)	2·13 (0·701–5·06)	1·93 (0·658–4·41)	-9·4 (-69·8–199)	3·73 (1·25–9·08)	3·31 (1·03–7·67)	-11·3 (-70·7–189)
United Arab Emirates	0·00426 (0·00132–0·0111)	0·0104 (0·00335–0·0252)	144 (-25·3–835)	0·021 (0·00549–0·0598)	0·0662 (0·0214–0·155)	215 (-0·8–1160)	0·0253 (0·00742–0·0708)	0·0765 (0·025–0·177)	203 (-3·4–1080)
Yemen	6·85 (2·58–13·5)	6·95 (3·43–13·1)	1·4 (-50·2–128)	17 (8·44–30·4)	17 (8·28–29·8)	-0·2 (-53·9–117)	23·8 (11·4–43·5)	23·9 (12·3–42)	0·3 (-49·5–117)
South Asia	819 (433–1460)	1940 (1060–3130)	137 (24·3–313)	1460 (787–2280)	3490 (2310–5090)	138 (42·5–309)	2280 (1260–3610)	5430 (3420–8090)	138 (39·2–306)
Bangladesh	149 (79·9–239)	446 (270–643)	199 (80·1–379)	133 (65·9–204)	405 (296–537)	206 (95·7–443)	281 (154–436)	851 (598–1140)	202 (101–409)
Bhutan	0·309 (0·14–0·591)	0·646 (0·328–1·09)	109 (-3–382)	0·471 (0·205–0·892)	1·06 (0·624–1·72)	125 (21·3–325)	0·78 (0·361–1·35)	1·71 (0·992–2·68)	119 (16·9–319)
India	619 (317–1140)	1360 (693–2310)	119 (9·4–310)	1230 (660–1920)	2820 (1810–4210)	128 (33·5–301)	1850 (1010–2950)	4180 (2520–6390)	125 (28·8–297)
Nepal	7·66 (3·24–14·5)	15·4 (7·1–28·3)	100 (-4·8–326)	22·4 (11·5–35·7)	61 (41–88)	172 (62·4–362)	30 (15–48·6)	76·3 (49·6–113)	154 (55·3–340)
Pakistan	43·4 (18·9–85·7)	123 (52·9–224)	183 (19–625)	73·2 (34·9–140)	203 (106–381)	177 (18·9–474)	117 (57·2–217)	325 (166–591)	179 (21·5–523)
**Southeast Asia, East Asia, and Oceania**	185 (68·5–385)	246 (107–505)	32·5 (-46·1–244)	253 (101–573)	422 (168–866)	66·8 (-34·7–354)	438 (169–945)	667 (265–1360)	52·3 (-37·2–305)

	Female			Male			Both		
	Number of DALYs (thousands)		Change (%)	Number of DALYs (thousands)		Change (%)	Number of DALYs (thousands)		Change (%)
	1990	2023		1990	2023		1990	2023	
East Asia	45·3 (15·1–103)	42·3 (12·9–98·6)	-6·8 (-69·1–202)	136 (50·1–316)	179 (64·7–390)	31·9 (-52·7–307)	181 (67–424)	221 (76·7–487)	22·2 (-56·9–280)
China	44·6 (14·8–101)	41·3 (12·6–96·2)	-7·4 (-69·3–200)	134 (49·3–312)	175 (63·2–382)	30·9 (-53–303)	178 (66–418)	216 (74·9–476)	21·3 (-57·2–277)
Democratic People's Republic of Korea	0·317 (0·106–0·702)	0·631 (0·194–1·64)	98·9 (-33·3–503)	0·425 (0·145–1·02)	0·798 (0·255–1·86)	87·9 (-39·1–520)	0·742 (0·243–1·71)	1·43 (0·454–3·24)	92·6 (-32·9–508)
Taiwan	0·403 (0·134–0·957)	0·335 (0·102–0·781)	-16·8 (-69–160)	1·5 (0·562–3·16)	3·08 (1·17–6·82)	106 (-23·9–518)	1·9 (0·704–4·14)	3·41 (1·26–7·65)	79·7 (-33·2–447)
Oceania	0·665 (0·238–1·64)	2·85 (1·17–5·58)	329 (69·7–1050)	1 (0·394–2·3)	2·5 (0·871–5·81)	150 (-9·2–654)	1·67 (0·629–3·54)	5·35 (1·98–11)	221 (27–740)
American Samoa	0·00186 (0·000628–0·00425)	0·00292 (0·000942–0·00672)	56·5 (-50·2–393)	0·00648 (0·0022–0·0152)	0·00856 (0·00308–0·0203)	32 (-55–337)	0·00835 (0·0028–0·0194)	0·0115 (0·00417–0·0263)	37·5 (-51·1–341)
Cook Islands	0·00096 (0·000316–0·00218)	0·000682 (0·000227–0·0016)	-29 (-76·6–131)	0·00267 (0·000832–0·0064)	0·00262 (0·000982–0·0062)	-1·8 (-67·7–217)	0·00363 (0·00118–0·00864)	0·00331 (0·00122–0·00768)	-9 (-69·1–182)
Fiji	0·0339 (0·012–0·0798)	0·0696 (0·021–0·156)	105 (-30·6–536)	0·0773 (0·0257–0·174)	0·124 (0·0424–0·276)	60·8 (-40·9–393)	0·111 (0·0381–0·256)	0·194 (0·0627–0·423)	74·4 (-35·5–448)
Guam	0·00324 (0·00113–0·00727)	0·0109 (0·00442–0·0229)	237 (36–837)	0·0218 (0·00896–0·0444)	0·0668 (0·0346–0·118)	206 (37·9–599)	0·0251 (0·0102–0·0517)	0·0777 (0·0398–0·142)	210 (35·1–632)
Kiribati	0·00357 (0·00116–0·00759)	0·0109 (0·00365–0·0246)	206 (3·9–755)	0·0283 (0·0111–0·0608)	0·0577 (0·0248–0·122)	104 (-17·6–443)	0·0318 (0·0121–0·0685)	0·0687 (0·0289–0·143)	116 (-13·2–466)
Marshall Islands	0·00273 (0·000863–0·0067)	0·00814 (0·00316–0·019)	198 (3·8–739)	0·0126 (0·00485–0·0269)	0·0181 (0·00786–0·0381)	43·5 (-44·3–309)	0·0153 (0·00588–0·0324)	0·0262 (0·0115–0·0542)	71 (-32·3–383)
Micronesia (Federated States of)	0·00764 (0·00259–0·0174)	0·0099 (0·00313–0·0226)	29·6 (-57·6–288)	0·037 (0·0143–0·0792)	0·0322 (0·0123–0·0757)	-12·9 (-68·2–169)	0·0447 (0·0167–0·0985)	0·0421 (0·0161–0·0943)	-5·7 (-65·7–182)
Nauru	0·000348 (9·97e-05–0·000869)	0·000706 (0·000215–0·00163)	103 (-36·9–551)	0·00064 (0·00017–0·00166)	0·000838 (0·00029–0·00199)	30·9 (-59·5–342)	0·000988 (0·000306–0·00264)	0·00154 (0·000555–0·00349)	56·2 (-47·3–414)
Niue	0·000125 (4·11e-05–0·000277)	0·000111 (3·79e-05–0·000255)	-10·7 (-70·4–200)	0·000197 (6·08e-05–0·000454)	0·000157 (5·15e-05–0·000343)	-20·1 (-72·1–145)	0·000321 (0·000102–0·000765)	0·000268 (9·2e-05–0·000615)	-16·4 (-70·6–160)
Northern Mariana Islands	0·00262 (0·000923–0·00606)	0·00533 (0·00199–0·0112)	103 (-26·7–514)	0·0102 (0·00381–0·0225)	0·0206 (0·00798–0·0435)	101 (-25·9–478)	0·0129 (0·00467–0·0287)	0·026 (0·0101–0·0548)	102 (-25·1–473)

	Female			Male			Both		
	Number of DALYs (thousands)		Change (%)	Number of DALYs (thousands)		Change (%)	Number of DALYs (thousands)		Change (%)
	1990	2023		1990	2023		1990	2023	
Palau	0·0148 (0·00698–0·0265)	0·0321 (0·0166–0·0554)	117 (3·1–360)	0·0274 (0·0126–0·0502)	0·0506 (0·0253–0·0911)	85·1 (-16–316)	0·0421 (0·0201–0·0776)	0·0828 (0·0431–0·137)	96·4 (-8·6–311)
Papua New Guinea	0·516 (0·181–1·29)	2·41 (0·997–4·78)	367 (82·5–1160)	0·578 (0·206–1·33)	1·59 (0·496–3·78)	175 (-1·9–808)	1·09 (0·409–2·33)	4 (1·52–8·24)	265 (41·3–847)
Samoa	0·00448 (0·00135–0·0102)	0·0089 (0·0028–0·0219)	98·7 (-40·1–535)	0·00903 (0·00323–0·0207)	0·0149 (0·00482–0·0361)	64·5 (-44·7–494)	0·0135 (0·00454–0·0309)	0·0238 (0·00798–0·0576)	75·8 (-39·1–473)
Solomon Islands	0·0222 (0·00668–0·0535)	0·134 (0·0421–0·292)	503 (104–1910)	0·0925 (0·0316–0·209)	0·325 (0·119–0·727)	251 (37·6–1010)	0·115 (0·0407–0·259)	0·459 (0·159–0·978)	300 (54·4–1130)
Tokelau	6·97e-05 (2·13e-05–0·000157)	9·35e-05 (3·08e-05–0·000218)	34·1 (-55·9–346)	0·00019 (6·32e-05–0·000437)	0·000194 (6·56e-05–0·000435)	2·3 (-62·9–252)	0·000259 (8·54e-05–0·000625)	0·000287 (1e-04–0·00067)	10·8 (-60–253)
Tonga	0·00193 (0·000619–0·00425)	0·00331 (0·00105–0·00767)	71·7 (-47·4–456)	0·00338 (0·0012–0·00815)	0·00377 (0·00124–0·00898)	11·5 (-63·8–250)	0·00531 (0·00176–0·0122)	0·00708 (0·0024–0·0164)	33·4 (-52·9–314)
Tuvalu	0·000576 (0·000183–0·00135)	0·000677 (0·000221–0·00157)	17·6 (-66–285)	0·00115 (0·000366–0·00275)	0·00128 (0·000394–0·00289)	11·9 (-61·3–298)	0·00172 (0·000561–0·00429)	0·00196 (0·000656–0·00434)	13·8 (-60·7–271)
Vanuatu	0·00644 (0·00199–0·0147)	0·0286 (0·00908–0·0697)	344 (44·3–1230)	0·0296 (0·00985–0·07)	0·0784 (0·0269–0·177)	165 (-5·2–794)	0·036 (0·0118–0·0873)	0·107 (0·0373–0·243)	197 (5·8–846)
Southeast Asia	139 (54·3–279)	200 (89·7–401)	43·8 (-38·6–251)	116 (46·4–255)	240 (104–473)	107 (-10·7–412)	256 (98·8–507)	441 (190–844)	72·4 (-26·4–325)
Cambodia	10·1 (4·81–18·4)	20·1 (10–36·4)	98·8 (-7·1–345)	1·3 (0·48–3·35)	2·52 (0·974–6·12)	93·2 (-34–521)	11·4 (5·46–20·8)	22·6 (12·6–39·4)	98·1 (13·2–295)
Indonesia	50·4 (18·3–105)	52·1 (18–121)	3·3 (-62·9–192)	7·52 (2·51–17·8)	17·2 (5·85–40·6)	129 (-18–632)	57·9 (20·6–118)	69·3 (23·9–158)	19·6 (-55·9–239)
Lao People's Democratic Republic	2·55 (0·926–5·63)	6·2 (2·81–11·6)	143 (-5·7–521)	0·224 (0·0695–0·586)	0·469 (0·157–1·11)	109 (-32·5–640)	2·78 (1·03–5·98)	6·67 (2·94–12·6)	140 (-7·9–515)
Malaysia	1·59 (0·541–3·83)	3·42 (1·23–7·87)	115 (-28–554)	2·08 (0·742–4·72)	7·54 (2·76–16·3)	262 (31·4–1050)	3·67 (1·24–8·8)	11 (3·92–24·2)	198 (5·5–811)
Maldives	0·0681 (0·0238–0·152)	0·0883 (0·031–0·186)	29·6 (-51·9–290)	0·113 (0·0464–0·242)	0·164 (0·0744–0·323)	45·7 (-38·3–252)	0·181 (0·0715–0·397)	0·253 (0·107–0·483)	39·7 (-44·5–246)
Mauritius	0·224 (0·0801–0·46)	0·335 (0·111–0·742)	49·5 (-42·6–375)	0·13 (0·05–0·277)	0·236 (0·0835–0·521)	82·4 (-28·5–433)	0·353 (0·125–0·749)	0·571 (0·195–1·25)	61·5 (-37·1–393)
Myanmar	34·4 (12·9–71·5)	56·6 (20–115)	64·3 (-41·5–340)	69·6 (27·7–145)	134 (61·8–256)	92·4 (-17·9–369)	104 (40·5–210)	191 (81·9–358)	83·1 (-21·7–344)

	Female			Male			Both		
	Number of DALYs (thousands)		Change (%)	Number of DALYs (thousands)		Change (%)	Number of DALYs (thousands)		Change (%)
	1990	2023		1990	2023		1990	2023	
Philippines	4·04 (1·41–8·94)	8·53 (2·83–19·3)	111 (-26·3–512)	7·77 (2·74–17·6)	21·7 (8·44–47·4)	180 (9·2–709)	11·8 (4·14–26·7)	30·3 (11·3–63·6)	156 (-2·9–626)
Seychelles	0·014 (0·00471–0·0292)	0·0146 (0·00515–0·0328)	4·4 (-63·6–209)	0·0124 (0·00465–0·0286)	0·0246 (0·00878–0·0538)	98·7 (-32·6–527)	0·0263 (0·00973–0·0575)	0·0392 (0·0141–0·0867)	48·7 (-46·6–351)
Sri Lanka	5·12 (1·9–10·5)	11 (3·81–22·9)	114 (-19·6–472)	15·8 (6·83–30·2)	33·2 (15·5–59·3)	110 (-3·3–375)	20·9 (8·87–40·7)	44·2 (19·3–81·2)	111 (-8·3–383)
Thailand	16 (6·2–32·2)	24·1 (8·95–49·7)	50·6 (-42–298)	6·76 (2·37–16·1)	14·9 (5·57–33)	121 (-21·9–563)	22·8 (8·65–47·2)	39·1 (14·4–82·7)	71·5 (-35–377)
Timor-Leste	0·122 (0·0438–0·269)	0·323 (0·105–0·719)	164 (-14·9–702)	0·0528 (0·0186–0·132)	0·134 (0·0435–0·305)	154 (-13–791)	0·175 (0·0632–0·399)	0·457 (0·154–0·977)	161 (-8·7–659)
Viet Nam	14·5 (4·89–33·6)	17·4 (5·85–38·2)	20·1 (-52·7–247)	4·59 (1·55–10·4)	7·82 (2·81–17·3)	70·6 (-37·5–427)	19·1 (6·39–42·7)	25·2 (8·76–56·9)	32·3 (-48·4–297)
**Sub-Saharan Africa**	29·3 (10·8–61)	63·2 (23·8–138)	116 (-15·4–495)	39 (17·1–82·3)	79·5 (35·4–170)	104 (-19·6–415)	68·3 (27·6–145)	143 (59·6–296)	109 (-18·3–417)
Central Sub-Saharan Africa	2·77 (0·962–6·25)	5·13 (1·6–12·8)	84·9 (-36·2–482)	2·33 (0·718–5·39)	6·34 (2·24–15·8)	172 (-11·7–792)	5·11 (1·75–11·1)	11·5 (4–25·7)	125 (-18·9–585)
Angola	0·139 (0·0457–0·31)	0·41 (0·132–0·93)	196 (1·6–836)	0·277 (0·0841–0·686)	0·747 (0·228–1·85)	169 (-8·9–851)	0·416 (0·138–0·996)	1·16 (0·364–2·64)	178 (3·3–803)
Central African Republic	0·169 (0·057–0·404)	0·405 (0·111–0·908)	140 (-27·5–686)	0·0848 (0·0239–0·202)	0·177 (0·0595–0·398)	109 (-29·7–636)	0·253 (0·0849–0·624)	0·582 (0·187–1·29)	130 (-20·6–638)
Congo	0·118 (0·0413–0·261)	0·259 (0·0837–0·597)	118 (-27–610)	0·0492 (0·0157–0·11)	0·15 (0·049–0·356)	206 (3·4–963)	0·168 (0·0563–0·368)	0·409 (0·135–0·961)	144 (-9–708)
Democratic Republic of the Congo	2·31 (0·799–5·09)	3·99 (1·17–10)	72·8 (-40·6–432)	1·89 (0·59–4·4)	5·2 (1·9–13·1)	175 (-11·8–788)	4·2 (1·44–8·93)	9·19 (3·14–21)	119 (-22·7–570)
Equatorial Guinea	0·0205 (0·00686–0·0461)	0·0344 (0·0116–0·0764)	67·6 (-45·8–472)	0·0163 (0·00499–0·0397)	0·0421 (0·0137–0·11)	159 (-19·8–896)	0·0368 (0·0125–0·0869)	0·0765 (0·026–0·181)	108 (-28·8–605)
Gabon	0·0197 (0·00669–0·0448)	0·0319 (0·01–0·0724)	62·2 (-46·4–419)	0·0101 (0·00332–0·0241)	0·0234 (0·00809–0·055)	132 (-22·1–724)	0·0298 (0·00981–0·0675)	0·0553 (0·0179–0·128)	85·7 (-33·5–506)
Eastern Sub-Saharan Africa	14·2 (5·79–29·1)	31·4 (13·3–65)	121 (-10·2–465)	25·9 (11·5–52·6)	52·2 (25·1–108)	101 (-15–367)	40·2 (17·8–80·7)	83·6 (39·2–165)	108 (-13·8–385)
Burundi	0·592 (0·213–1·29)	1·25 (0·442–2·53)	111 (-26–595)	1·18 (0·442–2·43)	2·44 (0·946–5·96)	108 (-27–525)	1·77 (0·647–3·66)	3·69 (1·36–8·43)	109 (-25·3–517)

	Female			Male			Both		
	Number of DALYs (thousands)		Change (%)	Number of DALYs (thousands)		Change (%)	Number of DALYs (thousands)		Change (%)
	1990	2023		1990	2023		1990	2023	
Comoros	0·0717 (0·0276–0·15)	0·0946 (0·0364–0·202)	31·9 (-49·8–242)	0·1 (0·0421–0·211)	0·163 (0·0675–0·34)	62·2 (-36·6–340)	0·172 (0·0706–0·363)	0·257 (0·101–0·518)	49·5 (-41·7–282)
Djibouti	0·0176 (0·00571–0·0402)	0·0539 (0·0181–0·115)	207 (6·2–799)	0·0309 (0·0124–0·0674)	0·107 (0·036–0·232)	247 (31–921)	0·0485 (0·0186–0·114)	0·161 (0·0544–0·337)	232 (18·7–913)
Eritrea	0·0555 (0·0185–0·127)	0·132 (0·0433–0·319)	137 (-20·2–691)	0·565 (0·194–1·31)	1·23 (0·471–2·74)	117 (-21·5–571)	0·62 (0·219–1·41)	1·36 (0·523–3·04)	119 (-21·5–558)
Ethiopia	0·593 (0·184–1·43)	2·1 (0·745–4·64)	254 (8·7–1150)	3·8 (1·42–8·29)	10·9 (4·31–26)	187 (-3·6–846)	4·39 (1·66–9·91)	13 (5·04–30·5)	196 (1·7–805)
Kenya	1·09 (0·403–2·29)	2·51 (0·876–5·42)	129 (-20·2–614)	1·28 (0·504–2·89)	3·02 (1·02–7·04)	136 (-18·1–583)	2·37 (0·949–5·14)	5·53 (1·86–12·2)	133 (-18·9–626)
Madagascar	5·82 (2·48–11)	13·8 (6·91–26·1)	138 (10·5–443)	14·5 (6·84–27·1)	25·4 (14·4–45·1)	75·2 (-12·7–278)	20·3 (9·51–37·7)	39·2 (21·9–67·2)	93 (-7·4–311)
Malawi	0·967 (0·342–2·08)	1·53 (0·564–3·37)	57·8 (-40·2–363)	0·424 (0·147–1·01)	0·752 (0·247–1·73)	77·4 (-40·2–500)	1·39 (0·512–3·05)	2·28 (0·811–5·19)	63·8 (-39·5–398)
Mozambique	0·727 (0·251–1·62)	1·02 (0·329–2·26)	40·3 (-51·7–333)	0·836 (0·283–2·02)	1·38 (0·466–3·26)	64·7 (-46·1–368)	1·56 (0·526–3·44)	2·4 (0·795–5·34)	53·3 (-47·1–348)
Rwanda	1·36 (0·504–2·88)	2·01 (0·746–4·06)	47·5 (-43·2–344)	0·216 (0·0724–0·501)	0·335 (0·12–0·748)	55 (-45·7–430)	1·58 (0·582–3·39)	2·35 (0·875–4·75)	48·5 (-41·6–350)
Somalia	0·161 (0·0498–0·412)	0·585 (0·2–1·32)	264 (24–1070)	0·239 (0·0835–0·536)	0·673 (0·218–1·66)	181 (-3·2–855)	0·4 (0·143–0·989)	1·26 (0·434–2·92)	214 (7·9–949)
South Sudan	0·349 (0·128–0·743)	0·749 (0·261–1·64)	115 (-24·4–513)	0·758 (0·268–1·84)	1·05 (0·369–2·45)	38 (-52·2–351)	1·11 (0·422–2·59)	1·79 (0·624–3·96)	62·2 (-44·3–395)
Uganda	1·19 (0·439–2·5)	2·79 (0·987–6·55)	134 (-15·3–623)	0·592 (0·214–1·24)	1·6 (0·546–3·84)	170 (-10·3–730)	1·78 (0·692–3·6)	4·39 (1·56–10·1)	146 (-11·6–641)
United Republic of Tanzania	0·91 (0·312–2·05)	1·75 (0·57–4·02)	92·6 (-33·6–502)	1·12 (0·402–2·58)	2·35 (0·823–5·27)	110 (-26·1–532)	2·03 (0·741–4·6)	4·1 (1·39–9·11)	102 (-28–542)
Zambia	0·319 (0·0928–0·769)	0·986 (0·324–2·29)	209 (-3·8–948)	0·259 (0·0942–0·594)	0·714 (0·237–1·64)	176 (-1·7–884)	0·578 (0·192–1·31)	1·7 (0·594–3·74)	194 (-0·4–809)
Southern Sub-Saharan Africa	4·42 (1·55–9·18)	9·35 (3·1–21·9)	112 (-23·5–511)	1·87 (0·709–4·23)	3·03 (1·07–6·52)	62·4 (-43·3–393)	6·28 (2·26–13·4)	12·4 (4·15–28·3)	97 (-27·1–463)
Botswana	0·333 (0·136–0·657)	0·593 (0·248–1·17)	78 (-29·6–382)	0·114 (0·0415–0·255)	0·286 (0·106–0·648)	152 (-5·3–665)	0·447 (0·182–0·918)	0·879 (0·362–1·76)	96·8 (-22·6–419)

	Female			Male			Both		
	Number of DALYs (thousands)		Change (%)	Number of DALYs (thousands)		Change (%)	Number of DALYs (thousands)		Change (%)
	1990	2023		1990	2023		1990	2023	
Eswatini	0·0286 (0·00987–0·0618)	0·0537 (0·0181–0·118)	88 (-39·9–534)	0·0556 (0·0206–0·129)	0·0551 (0·0188–0·131)	-1 (-65·4–227)	0·0842 (0·0318–0·195)	0·109 (0·0369–0·232)	29·2 (-53·5–312)
Lesotho	0·13 (0·0427–0·288)	0·199 (0·0651–0·482)	52·7 (-46·6–370)	0·0783 (0·0273–0·178)	0·112 (0·0366–0·241)	42·9 (-50·3–360)	0·209 (0·0731–0·454)	0·311 (0·105–0·712)	49·1 (-50·2–358)
Namibia	0·124 (0·0435–0·267)	0·227 (0·0708–0·488)	82·8 (-32·7–428)	0·0809 (0·0269–0·194)	0·127 (0·0451–0·289)	57·4 (-47·8–392)	0·205 (0·0709–0·454)	0·354 (0·121–0·751)	72·8 (-36·2–388)
South Africa	3·49 (1·22–7·23)	7·14 (2·3–16·4)	105 (-26·1–505)	1·36 (0·518–3·07)	2·17 (0·741–4·64)	58·8 (-45·7–384)	4·85 (1·74–10·1)	9·3 (3·03–22·1)	91·8 (-29·5–470)
Zimbabwe	0·314 (0·103–0·66)	1·14 (0·371–2·45)	263 (27·2–1020)	0·175 (0·0632–0·394)	0·286 (0·101–0·728)	63·9 (-44·7–442)	0·488 (0·165–1·08)	1·42 (0·47–3·08)	192 (1·1–804)
Western Sub-Saharan Africa	7·88 (2·52–17·2)	17·3 (5·95–39·4)	120 (-28·2–593)	8·88 (3·26–20·5)	17·9 (7·04–41·9)	101 (-31·4–530)	16·8 (5·81–39)	35·2 (12·9–76·7)	110 (-21–531)
Benin	0·264 (0·0842–0·566)	0·565 (0·192–1·3)	114 (-24·8–558)	0·21 (0·0736–0·521)	0·473 (0·164–1·11)	126 (-27·1–624)	0·473 (0·153–1·06)	1·04 (0·354–2·26)	119 (-17·9–542)
Burkina Faso	0·525 (0·181–1·15)	1·13 (0·403–2·41)	116 (-27–542)	0·613 (0·217–1·45)	0·942 (0·354–2·21)	53·7 (-50·8–408)	1·14 (0·385–2·52)	2·07 (0·781–4·44)	82·2 (-31·8–444)
Cabo Verde	0·0108 (0·00367–0·0235)	0·0112 (0·00356–0·0245)	3·5 (-64·6–240)	0·00665 (0·00227–0·0162)	0·013 (0·00428–0·0281)	95·7 (-32–522)	0·0175 (0·00574–0·0397)	0·0242 (0·00793–0·0532)	38·5 (-51·3–357)
Cameroon	1·17 (0·382–3·03)	3·42 (1·27–7·74)	191 (-5·9–789)	0·412 (0·141–0·918)	1·07 (0·369–2·6)	160 (-19·2–783)	1·59 (0·52–3·9)	4·49 (1·66–10·3)	183 (-4·3–734)
Chad	0·532 (0·172–1·12)	1·16 (0·372–2·76)	119 (-25·2–633)	0·554 (0·2–1·32)	1·3 (0·488–2·99)	135 (-18·7–627)	1·09 (0·362–2·31)	2·47 (0·861–5·58)	127 (-16·5–602)
Côte d'Ivoire	0·309 (0·102–0·711)	0·701 (0·234–1·53)	127 (-24·9–595)	0·182 (0·0573–0·423)	0·541 (0·186–1·33)	198 (0·1–884)	0·491 (0·158–1·16)	1·24 (0·41–2·86)	153 (-8–705)
Gambia	0·0221 (0·00754–0·0478)	0·0818 (0·0254–0·194)	270 (12–1140)	0·0199 (0·00634–0·0491)	0·0464 (0·0159–0·12)	133 (-25·5–677)	0·042 (0·0134–0·0991)	0·128 (0·0446–0·308)	205 (6–928)
Ghana	0·619 (0·195–1·41)	1·42 (0·445–3·51)	129 (-21·2–724)	1·12 (0·339–2·7)	2·35 (0·752–5·7)	111 (-27·2–593)	1·73 (0·566–4·09)	3·77 (1·21–8·94)	117 (-21·6–610)
Guinea	0·305 (0·0989–0·656)	0·485 (0·16–1·12)	58·8 (-48·6–427)	0·381 (0·122–0·991)	0·559 (0·19–1·39)	46·8 (-51–423)	0·686 (0·229–1·59)	1·04 (0·356–2·4)	52·1 (-46·4–393)
Guinea-Bissau	0·0428 (0·0136–0·0959)	0·092 (0·0307–0·217)	115 (-26·5–576)	0·0301 (0·00957–0·0743)	0·05 (0·0159–0·118)	65·9 (-43·5–457)	0·073 (0·0235–0·173)	0·142 (0·0485–0·336)	94·6 (-30·3–496)

	Female			Male			Both		
	Number of DALYs (thousands)		Change (%)	Number of DALYs (thousands)		Change (%)	Number of DALYs (thousands)		Change (%)
	1990	2023		1990	2023		1990	2023	
Liberia	0·1 (0·0328–0·224)	0·199 (0·0635–0·507)	98·6 (-38·9–563)	0·19 (0·06–0·489)	0·336 (0·115–0·791)	76·8 (-45·1–506)	0·29 (0·0921–0·715)	0·535 (0·184–1·26)	84·3 (-37·4–519)
Mali	0·335 (0·104–0·739)	0·654 (0·202–1·54)	95·2 (-33–543)	1·06 (0·436–2·4)	2·37 (0·972–5·27)	123 (-20·9–534)	1·4 (0·539–3·28)	3·02 (1·17–6·92)	116 (-21·8–516)
Mauritania	0·102 (0·0332–0·233)	0·192 (0·0606–0·477)	87·6 (-39·6–447)	0·0998 (0·0356–0·239)	0·193 (0·0646–0·485)	93·5 (-40·8–512)	0·202 (0·0688–0·485)	0·385 (0·131–0·894)	90·5 (-37·1–452)
Niger	0·343 (0·109–0·8)	1·09 (0·333–2·72)	216 (-1·4–889)	1·12 (0·447–2·57)	3 (1·16–6·18)	167 (-5·7–684)	1·47 (0·548–3·36)	4·09 (1·61–8·14)	179 (-4·9–696)
Nigeria	2·18 (0·667–4·94)	3·9 (1·3–9·31)	79·2 (-44·7–463)	2·18 (0·748–5·15)	3·41 (1·15–8·98)	56·7 (-50·1–457)	4·35 (1·44–10·1)	7·31 (2·53–17·3)	67·9 (-40·7–444)
Sao Tome and Principe	0·00277 (0·000898–0·006)	0·00381 (0·00112–0·00943)	37·8 (-56·9–353)	0·00313 (0·001–0·00742)	0·00771 (0·00258–0·0184)	146 (-19·6–666)	0·0059 (0·00189–0·0133)	0·0115 (0·00389–0·026)	95·4 (-35·1–498)
Senegal	0·304 (0·0978–0·709)	0·878 (0·277–2·03)	189 (1·2–829)	0·138 (0·046–0·351)	0·266 (0·0931–0·658)	92·2 (-38·2–539)	0·442 (0·141–0·996)	1·14 (0·378–2·65)	159 (-6–743)
Sierra Leone	0·48 (0·17–1·14)	0·664 (0·212–1·46)	38·4 (-52·2–330)	0·39 (0·126–0·889)	0·58 (0·189–1·37)	48·7 (-50·9–373)	0·87 (0·295–2·06)	1·24 (0·418–2·7)	43 (-47·2–334)
Togo	0·234 (0·0765–0·497)	0·67 (0·216–1·63)	187 (-3·6–795)	0·173 (0·0547–0·413)	0·38 (0·115–0·937)	120 (-24·4–593)	0·406 (0·133–0·942)	1·05 (0·335–2·48)	159 (-7·1–742)

Return to Table of Contents

Appendix Table 2·13: Age-standardized DALY rate attributable to chewing tobacco use in 1990 and 2023, among ages 15+

	Female			Male			Both		
	Age-standardized DALY rate (per 100,000)		Change (%)	Age-standardized DALY rate (per 100,000)		Change (%)	Age-standardized DALY rate (per 100,000)		Change (%)
	1990	2023		1990	2023		1990	2023	
Global	69·8 (35·7–129)	66·9 (35·6–110)	-4·2 (-50·4–72·1)	136 (70·4–226)	134 (85·8–202)	-1·7 (-40·7–70·2)	103 (52·2–169)	100 (60·8–152)	-2·6 (-44·7–71·4)
**Central Europe, Eastern Europe, and Central Asia**	5·2 (1·6–12·2)	2·9 (0·9–6·9)	-43·5 (-80·4–83·2)	16·1 (6·1–35·2)	12·2 (4·3–26·5)	-24·3 (-72·6–123·8)	10·3 (3·5–22)	7·3 (2·4–15·7)	-29·2 (-73·8–120·3)
Central Asia	7·3 (2·4–17·4)	4·2 (1·3–9·9)	-42 (-80·2–84)	53·1 (20·6–112)	29·2 (10·7–61·7)	-44·9 (-78·8–49)	29·3 (10·1–57)	16·4 (5·5–33·3)	-43·9 (-77·6–61·6)
Armenia	2·9 (0·9–7)	1 (0·3–2·5)	-63·8 (-88·1–15·2)	5·5 (2–12·1)	2·5 (0·8–5·7)	-54·7 (-83·3–38·6)	4·1 (1·4–9·3)	1·7 (0·5–3·9)	-58·7 (-85·3–29·9)
Azerbaijan	3·1 (1–7·2)	1·6 (0·5–4)	-47·5 (-82·3–71·1)	12·7 (4·6–28·9)	5·9 (2–13·7)	-53·8 (-83·8–38·8)	7·7 (2·4–15·6)	3·7 (1·2–8·2)	-51·7 (-81·3–63·4)
Georgia	5 (1·6–12·1)	3·2 (1–7·6)	-36·7 (-77·6–99·5)	8·7 (3·1–19·7)	6·8 (2·2–15)	-22·5 (-71·5–133·3)	6·7 (2·2–15·5)	4·9 (1·5–10·9)	-27·9 (-74·5–120·8)
Kazakhstan	4·6 (1·6–11·1)	2 (0·6–4·6)	-56·6 (-85·1–40·7)	19·9 (7·6–43·3)	9 (2·9–20·3)	-54·7 (-83·8–35·7)	11·9 (3·8–23·1)	5·3 (1·5–10·9)	-54·9 (-83·8–42·2)
Kyrgyzstan	15·7 (5·1–37)	4·5 (1·5–10·4)	-71·3 (-90–-7)	279 (111–569)	100 (40·5–197)	-64 (-85·6–-7·9)	142 (48·1–251)	51·1 (19–92·2)	-64 (-84·6–-2)
Mongolia	8·6 (3·1–18·6)	3 (0·9–6·7)	-64·8 (-88–13·6)	27 (8·8–63·4)	12·6 (4·2–27·7)	-53·3 (-83·3–53·1)	17·7 (5·6–38·2)	7·7 (2·3–15·1)	-56·7 (-84·8–43·5)
Tajikistan	18 (6·2–40·2)	18·2 (5·1–42·2)	1·4 (-64·4–238·2)	16·2 (5·9–37·5)	10·8 (3·6–25·1)	-33·7 (-76·9–100·5)	17·1 (6–38·9)	14·5 (4·3–34·4)	-15·5 (-70·6–161·2)
Turkmenistan	5·8 (2–13·9)	4 (1·2–9·1)	-30·7 (-77·7–120·6)	12·6 (4·8–27)	8·4 (2·8–19·1)	-33·6 (-76·8–99·8)	9·1 (3·2–19·4)	6·3 (2–13·8)	-31·4 (-76·3–116·7)
Uzbekistan	10·2 (3·5–24·1)	5·3 (1·6–12·1)	-47·9 (-82·4–61·7)	105 (40·8–221)	56·3 (19·4–121)	-46·2 (-79·7–52·6)	56·3 (20·3–112)	30·4 (10–63)	-46 (-79·2–59·6)
Central Europe	4·7 (1·5–11·1)	2·8 (0·9–6·6)	-39 (-78·4–105·7)	12·1 (4·5–27·6)	10·6 (3·7–23·6)	-12 (-67·9–170·4)	8·3 (2·9–18·7)	6·6 (2·2–14·6)	-20 (-70·7–157·4)
Albania	4·8 (1·5–10·8)	3 (0·9–6·8)	-36·7 (-77·6–102·7)	12·4 (4·5–27·9)	12·8 (4·2–29)	2·7 (-62·9–197·6)	8·7 (3·1–20·3)	7·8 (2·4–16·9)	-10·6 (-68·6–157·9)
Bosnia and Herzegovina	4·9 (1·5–11·5)	3·6 (1·1–8·2)	-27·4 (-74·7–140·4)	10·2 (3·6–24·1)	9·3 (3·1–21·1)	-8·8 (-67·6–182)	7·5 (2·5–17·4)	6·3 (2–14·2)	-15·9 (-68·7–174·9)
Bulgaria	9·2 (2·9–21·5)	7·7 (2·4–17·8)	-16·3 (-70·5–176)	23·5 (8–53·9)	24·1 (8–53·5)	2·5 (-63–209·4)	16·1 (5·2–36·4)	15·6 (5–34·3)	-3·6 (-65–201·3)

	Female			Male			Both		
	Age-standardized DALY rate (per 100,000)		Change (%)	Age-standardized DALY rate (per 100,000)		Change (%)	Age-standardized DALY rate (per 100,000)		Change (%)
	1990	2023		1990	2023		1990	2023	
Croatia	3·3 (1–7·6)	1·3 (0·4–3·1)	-59·3 (-85·9–34·1)	9 (3·4–19·6)	4·8 (1·6–10·6)	-46·6 (-80·7–62·6)	6 (2·2–13·1)	3 (1–6·5)	-50·3 (-82·1–59·1)
Czechia	4·9 (1·5–11·6)	2·1 (0·7–4·8)	-57·8 (-85–43)	12·5 (4·6–27·9)	7·8 (2·7–17·2)	-37·8 (-76·7–93·8)	8·5 (2·8–18·7)	4·9 (1·6–10·6)	-43·1 (-78·3–89)
Hungary	3·7 (1·2–8·8)	1·5 (0·5–3·5)	-59·4 (-85·4–36·1)	10·8 (4–23·4)	5·1 (1·8–11·4)	-52·6 (-83–48·6)	7·1 (2·5–15·8)	3·2 (1·1–7·1)	-54·4 (-83·8–46·6)
Montenegro	5·9 (1·9–14·2)	4·6 (1·5–10·6)	-21·8 (-73·8–140·9)	11·8 (4·1–27)	11 (3·7–24·8)	-7 (-65–182·4)	8·8 (2·9–20·5)	7·7 (2·5–17·3)	-12·1 (-68–178·1)
North Macedonia	6·9 (2·3–16·5)	5·1 (1·6–12)	-25·8 (-74·1–141·2)	12·2 (4·2–28·2)	11·3 (3·8–24·7)	-7·9 (-65·5–187·5)	9·6 (3·1–22)	8·2 (2·7–17·8)	-14·6 (-69–170)
Poland	3·8 (1·2–9)	2·9 (0·9–6·6)	-25 (-72·9–156·6)	9·7 (3·8–21·6)	10·6 (3·7–23)	8·6 (-60·6–232·8)	6·7 (2·4–14·9)	6·6 (2·2–14·4)	-1·5 (-63·7–217·2)
Romania	4·3 (1·4–10·2)	2·2 (0·7–5·1)	-49·4 (-81·8–65·9)	11·6 (4·3–26·4)	12·8 (4·4–28·6)	10·2 (-59·8–245·7)	7·9 (2·7–17·8)	7·3 (2·4–16)	-7·4 (-66·2–193·9)
Serbia	7·7 (2·4–19)	4·5 (1·4–10·4)	-40·8 (-80–92·8)	15·4 (5·2–35·4)	11·4 (3·9–25·4)	-25·8 (-74·7–126)	11·5 (3·8–26·8)	8 (2·5–17·3)	-30·6 (-76·4–112·5)
Slovakia	2·7 (0·9–6·8)	1·1 (0·4–2·6)	-59 (-86–29·3)	9·4 (3·6–20·5)	4·9 (1·8–11)	-47·4 (-81·1–64·2)	5·9 (2·1–13)	3 (1–6·5)	-49·9 (-82·3–63·1)
Slovenia	2·7 (0·8–6·5)	1·5 (0·5–3·6)	-43·2 (-80·3–85·5)	9·7 (3·6–21·6)	7·7 (2·7–16·8)	-20·8 (-69·8–148·7)	6·1 (2·1–13·2)	4·6 (1·5–9·8)	-23·8 (-70·5–145·3)
Eastern Europe	5 (1·6–11·9)	2·6 (0·8–6·2)	-49 (-82·3–65·2)	11·3 (4·3–25·2)	7·4 (2·5–16·3)	-34·1 (-76·2–106)	7·9 (2·6–17·4)	4·8 (1·5–10·4)	-39·2 (-77·6–101·3)
Belarus	3·3 (1·1–7·8)	1·9 (0·6–4·5)	-42·4 (-80·5–90·6)	9·5 (3·6–21·2)	9·8 (3·5–21·9)	3·9 (-61·9–238)	6·1 (2·1–13·8)	5·5 (1·9–12)	-10·3 (-67·1–200·6)
Estonia	3·5 (1·1–8·3)	1·4 (0·4–3·3)	-58·9 (-85·9–39·4)	8·4 (3·1–18·4)	11·4 (4·1–24·7)	35·3 (-47·7–313·5)	5·7 (2–12·6)	6·1 (2·2–13·4)	6·2 (-57·8–246·1)
Latvia	3·8 (1·2–9·2)	1·9 (0·6–4·5)	-50 (-82·9–59·1)	7·7 (2·9–17·3)	5·5 (1·9–12·3)	-28 (-74·4–121·6)	5·6 (1·9–12·8)	3·6 (1·1–7·9)	-36·2 (-77·2–103·6)
Lithuania	2·3 (0·7–5·6)	1·3 (0·4–3·1)	-42·4 (-80·1–85·8)	5·6 (2·1–12·3)	4·9 (1·7–11)	-11·8 (-67·2–181·3)	3·8 (1·3–8·4)	3 (1–6·7)	-21·9 (-71·3–152·3)
Republic of Moldova	3·9 (1·2–9)	1·5 (0·5–3·6)	-61 (-86·7–30·5)	12·4 (4·8–27·6)	9 (3–19·7)	-27·1 (-74·2–123·5)	7·8 (2·7–16·7)	5 (1·5–10·4)	-35·7 (-77·7–102·5)
Russian Federation	5·7 (1·8–13·4)	2·9 (0·9–7·1)	-48·7 (-81·9–64·4)	12·2 (4·6–27·1)	7·3 (2·5–16·2)	-39·7 (-78·1–88·7)	8·7 (2·8–18·8)	5 (1·5–10·8)	-42·9 (-78·5–91·6)

	Female			Male			Both		
	Age-standardized DALY rate (per 100,000)		Change (%)	Age-standardized DALY rate (per 100,000)		Change (%)	Age-standardized DALY rate (per 100,000)		Change (%)
	1990	2023		1990	2023		1990	2023	
Ukraine	4 (1·3–9·2)	1·8 (0·5–4·1)	-55·3 (-85·1–47·7)	10·5 (3·9–23·6)	7·3 (2·5–16·5)	-29·9 (-75·2–112·7)	6·9 (2·3–15·3)	4·3 (1·4–9·4)	-37·9 (-77·6–96·9)
**High-income**	2·7 (0·9–6·2)	1·7 (0·6–4)	-34·6 (-77·1–110·2)	27 (16·4–42·4)	15·1 (9·4–26)	-44 (-67·8–-0·8)	14·4 (7·5–21·1)	8·3 (4·9–13·9)	-42·7 (-66·6–21·3)
Australasia	2·2 (0·7–5·3)	1·1 (0·3–2·5)	-50·6 (-82·8–61·9)	11·6 (4·3–25·4)	5·7 (2·1–12·7)	-50·5 (-81·5–47·5)	6·8 (2·3–14·1)	3·4 (1·2–7·4)	-50·6 (-80·9–57·3)
Australia	2·1 (0·7–5·1)	1 (0·3–2·3)	-53·2 (-83·6–52·9)	11·9 (4·4–25·8)	5·8 (2·1–12·8)	-51·1 (-81·7–44·1)	6·9 (2·4–14·2)	3·3 (1·1–7·3)	-51·7 (-81·2–53)
New Zealand	2·5 (0·8–5·9)	1·5 (0·5–3·6)	-37·2 (-78·7–104·6)	10·3 (3·8–22·9)	5·5 (2–12·3)	-46·7 (-80·2–65·7)	6·3 (2·2–13·2)	3·5 (1·2–7·7)	-44·4 (-78·9–85)
High-income Asia Pacific	3·8 (1·2–9·3)	1·9 (0·6–4·5)	-48·8 (-81·8–72·4)	19 (6·9–43·8)	9·8 (3·4–21·9)	-48·3 (-81·1–54·7)	11·2 (3·7–24)	5·8 (1·9–12·6)	-48·4 (-80·2–67·4)
Brunei Darussalam	22·7 (7·9–55·8)	11·6 (3·8–27)	-49 (-82·8–63·7)	35·2 (12·5–86·1)	20 (6·9–45·2)	-43 (-80·3–87)	29·4 (10·2–71·4)	16·1 (5·4–36·2)	-45·3 (-81·3–65)
Japan	3 (0·9–7·3)	2·1 (0·7–4·8)	-30 (-74·6–132·6)	15·9 (5·9–36)	10·5 (3·7–23·5)	-33·9 (-76–99)	9·3 (3·1–19·4)	6·1 (2–13·6)	-33·7 (-74·4–118·1)
Republic of Korea	8·3 (2·6–21·5)	1·7 (0·6–4)	-79·6 (-92·8–-36·1)	39 (13·1–93·1)	8·4 (2·7–18)	-78·4 (-92·2–-35·6)	23·5 (6·6–48·4)	5·1 (1·5–10·3)	-78·5 (-91·6–-28)
Singapore	4·6 (1·5–10·9)	1·7 (0·5–4·2)	-62·4 (-86·9–22·2)	22·5 (8·5–49·8)	8·1 (2·9–18·7)	-63·8 (-85·8–9·3)	13·5 (4·8–28·8)	5 (1·7–11·3)	-63 (-85·4–15·9)
High-income North America	4·1 (1·4–9·3)	3·2 (1–7·4)	-22·9 (-73·5–138·1)	72·2 (48·5–109)	37·6 (23·3–58·2)	-48 (-67·1–-18·3)	36·9 (20·5–49·1)	20·1 (11·7–31)	-45·6 (-64·7–1·9)
Canada	4·1 (1·4–9·9)	2·9 (0·9–6·8)	-29·3 (-75·4–126·4)	46·2 (18·4–92·1)	16·2 (5·8–35·1)	-65 (-86·5–-0·2)	24·7 (8·7–45)	9·4 (3·2–20·1)	-61·9 (-84·4–19·8)
Greenland	17·4 (6–39·1)	7·6 (2·4–17·3)	-56·2 (-84·9–51·8)	190 (73·5–414)	77·5 (29·1–171)	-59·2 (-85·1–21·2)	113 (36·6–207)	44·7 (16·4–97·2)	-60·3 (-83–38·8)
United States of America	4·1 (1·4–9·2)	3·2 (1–7·5)	-22 (-72·9–140·9)	75 (50·8–112)	40·2 (25–62·3)	-46·4 (-65·8–-17·3)	38·2 (21·8–51)	21·4 (12·5–33)	-44 (-62·5–2·2)
Southern Latin America	2·7 (0·9–6·5)	1·1 (0·3–2·4)	-60·6 (-86·2–28·9)	5·2 (2–11·8)	2 (0·7–4·5)	-61·8 (-86·4–19)	3·9 (1·4–8·9)	1·5 (0·5–3·4)	-61·4 (-86·3–23·9)
Argentina	2·7 (0·9–6·4)	1 (0·3–2·4)	-60·6 (-86–29·1)	4·6 (1·7–10·4)	1·9 (0·7–4·3)	-58·3 (-85·1–29·8)	3·6 (1·3–8·3)	1·5 (0·5–3·3)	-59·3 (-85·7–30)
Chile	2·8 (0·9–6·7)	1 (0·3–2·5)	-62·8 (-87·1–20·4)	6·9 (2·6–15·2)	2 (0·7–4·5)	-70·9 (-89·6–-9·8)	4·7 (1·7–10·5)	1·5 (0·5–3·3)	-68·4 (-88·6–0·8)

	Female			Male			Both		
	Age-standardized DALY rate (per 100,000)		Change (%)	Age-standardized DALY rate (per 100,000)		Change (%)	Age-standardized DALY rate (per 100,000)		Change (%)
	1990	2023		1990	2023		1990	2023	
Uruguay	3·2 (1–7·4)	1·5 (0·4–3·5)	-52·4 (-83·5–51)	6·5 (2·5–13·8)	3·2 (1·1–7)	-51·1 (-82·2–54·7)	4·8 (1·7–10·7)	2·3 (0·8–5·2)	-51·9 (-82·6–52·2)
Western Europe	1·4 (0·4–3·3)	0·8 (0·3–1·9)	-40·9 (-78·9–97·6)	4·4 (1·6–9·5)	2·9 (1–6·5)	-33·3 (-76·2–101)	2·8 (1–6·1)	1·8 (0·6–4·1)	-34·8 (-76·5–105·9)
Andorra	0·9 (0·3–2·1)	0·6 (0·2–1·4)	-31·9 (-77·9–110·9)	3 (1·1–6·5)	2·8 (0·9–5·9)	-6 (-67·9–191·2)	2 (0·7–4·5)	1·7 (0·6–3·8)	-12·6 (-69·4–165·7)
Austria	1·4 (0·4–3·3)	0·9 (0·3–2·1)	-34·8 (-77·1–118·3)	4·9 (1·8–10·8)	4·1 (1·4–9)	-17 (-70·1–149·3)	3 (1–6·6)	2·4 (0·8–5·3)	-19·5 (-70·7–152·3)
Belgium	1·3 (0·4–3·1)	0·6 (0·2–1·5)	-52·1 (-83·1–59·8)	3·3 (1·2–7·3)	1·7 (0·6–3·9)	-49 (-82·1–58·8)	2·3 (0·8–5·1)	1·2 (0·4–2·7)	-49·7 (-81·9–63·9)
Cyprus	1·9 (0·7–4·7)	0·6 (0·2–1·4)	-66·3 (-89·1–8·5)	4·1 (1·3–9·9)	1·8 (0·6–4·2)	-56 (-84·7–43·5)	3 (0·9–7)	1·2 (0·4–2·6)	-59·7 (-86·4–22·7)
Denmark	1·9 (0·6–4·4)	1·5 (0·5–3·6)	-16·7 (-70–173·7)	7·6 (2·9–17)	6·3 (2·3–13·9)	-16·5 (-69·4–141)	4·7 (1·6–9·5)	3·9 (1·4–8·6)	-16 (-66·8–169·6)
Finland	1·4 (0·4–3·4)	0·8 (0·3–2)	-39·5 (-78·3–98·3)	3·2 (1·2–7·3)	3·3 (1·2–7·3)	5·3 (-61·5–220·3)	2·2 (0·8–5·2)	2·1 (0·7–4·7)	-7·8 (-65·4–192)
France	1 (0·3–2·3)	0·6 (0·2–1·4)	-37·2 (-77·9–108·6)	5 (1·9–10·7)	2·1 (0·7–4·8)	-58 (-85·3–30)	2·9 (1–5·9)	1·3 (0·4–3)	-54·5 (-84–45·3)
Germany	1·4 (0·4–3·5)	1·1 (0·3–2·6)	-21·8 (-72·2–160·9)	4 (1·5–8·8)	4 (1·4–9)	-0·8 (-64·2–209·4)	2·7 (0·9–5·8)	2·5 (0·8–5·6)	-5·3 (-65·3–207·8)
Greece	2·3 (0·7–5·6)	0·8 (0·3–1·9)	-63·4 (-87·3–22)	3·8 (1·4–8·8)	1·9 (0·7–4·5)	-49·6 (-81·9–57·5)	3 (1–7)	1·4 (0·4–3·1)	-55 (-84–43·7)
Iceland	1·8 (0·6–4·4)	1·5 (0·5–3·4)	-18·5 (-71·4–180)	8·2 (3·1–18·5)	10·5 (4–23·1)	27·1 (-52·2–277·2)	5 (1·7–10·8)	6·1 (2·2–13·1)	21·7 (-53·8–280·7)
Ireland	1·5 (0·5–3·6)	0·7 (0·2–1·6)	-55·8 (-84–49·7)	3·1 (1·1–6·9)	1·7 (0·6–3·7)	-46·4 (-80·1–61·3)	2·3 (0·8–5·2)	1·2 (0·4–2·6)	-49·7 (-81·6–59·1)
Israel	1·2 (0·4–2·8)	0·6 (0·2–1·3)	-52·4 (-83·4–58)	2·6 (1–6)	1·6 (0·6–3·8)	-38·2 (-77·1–86·4)	1·9 (0·7–4·3)	1·1 (0·4–2·5)	-42·2 (-79–82·1)
Italy	1·3 (0·4–3)	0·6 (0·2–1·4)	-51·1 (-82·7–65·8)	4 (1·5–9)	2·1 (0·8–4·7)	-47·2 (-80·7–57·4)	2·6 (0·9–5·6)	1·3 (0·4–3)	-47·9 (-80·9–65·9)
Luxembourg	1·9 (0·6–4·5)	0·6 (0·2–1·4)	-69·2 (-89·3–1·1)	4·4 (1·6–10)	1·7 (0·6–3·8)	-62·2 (-86–12·9)	3·1 (1·1–6·9)	1·1 (0·4–2·5)	-63·8 (-86·9–13)
Malta	1·5 (0·5–3·6)	0·6 (0·2–1·3)	-63·6 (-87·1–20·7)	3·2 (1·2–7·2)	1·4 (0·5–3·1)	-57·2 (-84·1–33·3)	2·3 (0·8–5·2)	1 (0·3–2·2)	-58 (-84·5–34·8)
Monaco	2·1 (0·7–4·9)	1 (0·3–2·5)	-53·2 (-85·1–60·7)	5·9 (2·2–14)	3·8 (1·4–7·8)	-35·1 (-79·8–106·5)	3·9 (1·3–9)	2·4 (0·8–5·1)	-39 (-80·4–95·5)

	Female			Male			Both		
	Age-standardized DALY rate (per 100,000)		Change (%)	Age-standardized DALY rate (per 100,000)		Change (%)	Age-standardized DALY rate (per 100,000)		Change (%)
	1990	2023		1990	2023		1990	2023	
Netherlands	1·2 (0·4–2·8)	0·8 (0·2–1·8)	-34·8 (-77–113·1)	2·5 (0·9–5·6)	1·8 (0·6–4)	-29·5 (-74·2–110·3)	1·8 (0·6–4·1)	1·3 (0·4–2·9)	-31·1 (-74·3–117·8)
Norway	2·2 (0·7–5·2)	1·7 (0·5–4)	-20·6 (-71·4–169·3)	8·4 (3·1–19·1)	11·1 (4·2–25·1)	32·3 (-49·9–291·1)	5·2 (1·8–11·6)	6·4 (2·4–14·4)	23·3 (-52–284·7)
Portugal	2·9 (0·9–6·9)	0·7 (0·2–1·7)	-74·3 (-90·9–-16·6)	6·2 (2·3–14·2)	2·8 (1–6·3)	-54·9 (-83·5–43·3)	4·4 (1·5–10·1)	1·7 (0·6–3·9)	-61·6 (-86–25·6)
San Marino	1·3 (0·5–3·3)	0·7 (0·2–1·6)	-48·4 (-82·9–70·9)	4 (1·3–9·6)	1·8 (0·6–4·3)	-54·1 (-83·8–41·6)	2·6 (0·9–6·1)	1·2 (0·4–2·9)	-52·6 (-83·2–53·1)
Spain	1·4 (0·4–3·3)	0·5 (0·2–1·2)	-61·7 (-86·3–28·4)	4·2 (1·5–8·7)	1·6 (0·6–3·7)	-60·8 (-86·3–22·5)	2·7 (1–5·9)	1·1 (0·4–2·4)	-60·9 (-86·2–25·4)
Sweden	1·3 (0·4–3·2)	1·9 (0·6–4·3)	42·3 (-48·4–366·3)	15·5 (5·8–33·7)	13·9 (5·3–30·2)	-10·4 (-66–158)	8·3 (3·1–18)	7·9 (2·9–17·2)	-4·3 (-64–176·4)
Switzerland	1·1 (0·4–2·7)	0·6 (0·2–1·4)	-46·9 (-80·7–76·3)	3·6 (1·3–7·7)	2 (0·7–4·5)	-44·5 (-80·4–64·7)	2·3 (0·8–4·9)	1·3 (0·4–2·9)	-44·7 (-79·7–74·4)
United Kingdom	1·5 (0·5–3·5)	0·8 (0·3–1·9)	-43·4 (-79·9–89·4)	3 (1·1–6·6)	2·1 (0·7–4·8)	-28·9 (-73·9–118·7)	2·2 (0·8–4·9)	1·5 (0·5–3·3)	-33·7 (-75·5–110·7)
**Latin America and Caribbean**	7·1 (2·3–16·7)	3·5 (1·1–8·1)	-51·1 (-82·9–59·3)	13·2 (5–29·5)	8·6 (3–18·7)	-34·8 (-76·6–101·4)	10·1 (3·6–23·1)	6 (1·9–13)	-41 (-79·3–84)
Andean Latin America	2·9 (0·9–6·9)	1·5 (0·5–3·4)	-48·2 (-82·9–67·7)	4 (1·4–9·8)	2·7 (0·8–5·9)	-33·5 (-76–114·7)	3·4 (1·1–8·5)	2·1 (0·6–4·6)	-39·7 (-79·5–88·7)
Bolivia (Plurinational State of)	3·7 (1·2–8·5)	2·1 (0·7–4·8)	-43·6 (-80·9–90·1)	7·9 (2·3–20·4)	6 (2·1–14·4)	-24·1 (-77·5–161·3)	5·8 (1·7–13·9)	4 (1·3–9)	-30·2 (-78·3–122·1)
Ecuador	2·1 (0·7–5·1)	0·9 (0·3–2·2)	-55·7 (-84·2–47·4)	2·5 (0·9–5·8)	1·3 (0·4–2·8)	-49·9 (-81·5–56·4)	2·3 (0·8–5·5)	1·1 (0·4–2·5)	-52·7 (-82·8–49·3)
Peru	3 (1–7·3)	1·6 (0·5–3·5)	-47·2 (-84·3–72·1)	3·8 (1·3–9)	2·6 (0·7–5·6)	-31·6 (-76·4–112)	3·4 (1·1–8)	2·1 (0·6–4·6)	-38·4 (-79·5–93)
Caribbean	5·9 (2–13·2)	5·3 (1·7–12·3)	-9·8 (-70·6–170·5)	9·2 (3·4–21·6)	9·8 (3·5–22)	6·7 (-60·6–221·4)	7·5 (2·5–17·3)	7·5 (2·6–16·7)	0 (-64–206·2)
Antigua and Barbuda	3·7 (1·2–8·8)	1·8 (0·5–4·2)	-51·6 (-83·1–59·5)	7·7 (3–17·4)	4·3 (1·4–9·5)	-44·7 (-80·8–62·4)	5·6 (2–13)	3 (1–6·8)	-46·7 (-81·2–64·4)
Bahamas	3·7 (1·2–8·6)	2·5 (0·7–5·8)	-32·1 (-75·6–129·6)	10·4 (4–21·6)	7·7 (2·8–16·8)	-25·6 (-72·6–138)	7 (2·6–15·2)	5 (1·6–11)	-27·8 (-73·4–133·7)
Barbados	4·3 (1·4–10)	2·4 (0·7–5·5)	-42·7 (-80·7–80·9)	4·3 (1·7–9·2)	3·4 (1·1–7·6)	-20·3 (-70·7–143·5)	4·3 (1·6–10)	2·9 (0·9–6·5)	-32 (-74·9–112)

	Female			Male			Both		
	Age-standardized DALY rate (per 100,000)		Change (%)	Age-standardized DALY rate (per 100,000)		Change (%)	Age-standardized DALY rate (per 100,000)		Change (%)
	1990	2023		1990	2023		1990	2023	
Belize	2·7 (0·9–6·3)	1·7 (0·5–4·1)	-34·7 (-77·4–117·4)	3·7 (1·3–8·3)	3 (1–6·7)	-18·3 (-71·2–142·2)	3·2 (1·1–7·4)	2·4 (0·7–5·5)	-25·9 (-74·5–130·4)
Bermuda	2·3 (0·8–5·4)	1·1 (0·3–2·6)	-53 (-83·1–53·9)	6·7 (2·7–13·8)	3·8 (1·3–8)	-44 (-80–74·3)	4·4 (1·7–9·3)	2·4 (0·8–5·2)	-46·4 (-80·3–73·3)
Cuba	3 (1–7)	1·8 (0·6–4)	-41·4 (-78·9–90·2)	4·7 (1·7–10·1)	4·7 (1·7–11·1)	0·1 (-63·2–212·5)	3·9 (1·4–8·7)	3·2 (1·2–7·5)	-16·6 (-69·8–165·8)
Dominica	2·3 (0·7–5·4)	1·7 (0·5–4·1)	-27·2 (-75·2–149·9)	4 (1·5–9·1)	3·5 (1·3–7·8)	-11·9 (-66·9–171·3)	3·1 (1·1–7·2)	2·6 (0·9–6·2)	-16·3 (-68·6–174·6)
Dominican Republic	5·2 (1·7–12·4)	3·4 (1–7·4)	-34·1 (-75·7–108)	12·8 (4·7–27)	13·6 (4·7–30·8)	6·4 (-57·7–220·7)	8·8 (3·2–19·6)	8·4 (2·8–18·8)	-4·8 (-62·8–183·5)
Grenada	6·5 (2·1–14·7)	3·2 (0·9–7·6)	-50·7 (-81·5–60)	14·5 (5·6–32)	8·6 (3·2–19·4)	-40·6 (-77·2–75·6)	10·4 (3·7–23·4)	6 (2·1–13·6)	-42·4 (-77·4–81·6)
Guyana	7·4 (2·3–17)	3·1 (0·9–7·2)	-57·7 (-85·8–37·1)	11·6 (3·8–25·5)	5·7 (1·9–13·2)	-51·2 (-82·8–39·1)	9·4 (3·1–21·4)	4·4 (1·4–10·1)	-53·8 (-82·9–34·9)
Haiti	20·9 (7–46·7)	21·4 (6·9–50·4)	2·8 (-66·8–226·1)	23·4 (7·3–57·4)	25·2 (8·2–55·2)	7·6 (-63·1–247·8)	22·1 (7·5–49·4)	23·2 (8–52)	5·3 (-63·6–234·5)
Jamaica	3·9 (1·3–9·4)	2·5 (0·7–5·7)	-36 (-77·1–114·8)	5·5 (2–12·8)	5·4 (1·9–12·4)	-2·1 (-64·9–196·1)	4·7 (1·5–11)	3·9 (1·3–9·2)	-16·3 (-70·7–168·4)
Puerto Rico	1·8 (0·6–4·4)	0·7 (0·2–1·6)	-62·1 (-87–21·5)	4·9 (1·8–10·4)	2 (0·7–4·3)	-60·1 (-85·8–19)	3·3 (1·2–7·2)	1·3 (0·5–2·9)	-60·9 (-86·3–20)
Saint Kitts and Nevis	5·2 (1·6–12)	2·3 (0·7–5·5)	-54·6 (-83·9–51·6)	6·7 (2·5–14·8)	3·5 (1·1–7·8)	-47·2 (-81·1–60·9)	5·9 (2–13·6)	2·9 (0·9–6·8)	-50·7 (-81·9–57·1)
Saint Lucia	5·4 (1·8–13·1)	1·9 (0·6–4·5)	-63·8 (-87·9–23·8)	9·8 (3·7–20·2)	5·1 (1·8–11·7)	-48·6 (-83·5–57·5)	7·5 (2·8–17)	3·5 (1·1–8·1)	-53·6 (-84·7–49·4)
Saint Vincent and the Grenadines	3·1 (1–7·3)	1·7 (0·5–3·9)	-44·7 (-80·5–73·2)	9·4 (3·7–19·5)	7·5 (2·5–17)	-19·9 (-73·6–148·3)	6·2 (2·3–13·4)	4·7 (1·5–10·6)	-24·6 (-73·8–148·7)
Suriname	3·9 (1·2–9·6)	3 (0·9–7·3)	-24·2 (-74·8–146·5)	6·2 (2·3–14)	5·5 (1·8–12·5)	-11·7 (-67·9–145·1)	5·1 (1·8–11·8)	4·2 (1·3–9·8)	-17·4 (-71–145·6)
Trinidad and Tobago	6 (1·9–14·2)	3·1 (0·9–7·2)	-48·5 (-82–59·2)	8·9 (3·2–20)	6 (2–13·5)	-33·2 (-75·1–112·4)	7·5 (2·5–17·3)	4·5 (1·4–10·2)	-39·4 (-78·4–97·6)
United States Virgin Islands	2·3 (0·7–5·3)	0·9 (0·3–2)	-61·8 (-86·4–19·7)	4·1 (1·5–8·8)	3·2 (1·2–7)	-22·2 (-71·7–145·5)	3·1 (1·1–7·2)	2 (0·7–4·4)	-37·1 (-76·2–100·5)
Central Latin America	4·2 (1·4–10·1)	2·2 (0·7–5·3)	-47·4 (-81·7–71·2)	6·8 (2·6–15·3)	4·8 (1·5–10·3)	-30·6 (-75·7–116·7)	5·5 (2–12·8)	3·4 (1·1–7·7)	-37·5 (-78·8–99·1)

	Female			Male			Both		
	Age-standardized DALY rate (per 100,000)		Change (%)	Age-standardized DALY rate (per 100,000)		Change (%)	Age-standardized DALY rate (per 100,000)		Change (%)
	1990	2023		1990	2023		1990	2023	
Colombia	5·3 (1·7–12·5)	1·9 (0·6–4·4)	-63·6 (-87·1–20·6)	6·3 (2·4–14)	2·8 (0·9–6·2)	-55·2 (-84·1–34·3)	5·8 (2·1–13·4)	2·4 (0·8–5·4)	-59·2 (-85·8–26·7)
Costa Rica	1·8 (0·6–4·2)	1·1 (0·3–2·5)	-39·5 (-79–95·2)	3 (1·1–6·8)	2·1 (0·7–4·5)	-30·6 (-74·7–102·5)	2·4 (0·8–5·5)	1·6 (0·5–3·5)	-33·7 (-76·8–99·2)
El Salvador	4·5 (1·5–10·9)	2·4 (0·7–5·6)	-45·1 (-80·6–83·9)	6·7 (2·4–15·2)	4·1 (1·3–8·9)	-38·9 (-76·4–92·9)	5·5 (1·9–13)	3·2 (1–7·1)	-42·2 (-79·7–76·3)
Guatemala	3·8 (1·3–8·9)	2·2 (0·7–5·1)	-41·9 (-80·6–94)	3·7 (1·4–8·2)	2·3 (0·7–5·2)	-38·7 (-77·7–91·8)	3·7 (1·4–8·6)	2·2 (0·7–5·1)	-40·4 (-79·1–92·3)
Honduras	3·9 (1·2–8·4)	2·9 (0·9–7·2)	-25·4 (-75·3–140·3)	6·5 (2·3–15·7)	4·9 (1·7–12·5)	-24·4 (-75·7–146·7)	5·2 (1·7–12·3)	3·9 (1·3–9)	-25·2 (-75·1–125·1)
Mexico	2·5 (0·8–5·9)	1·1 (0·3–2·6)	-55·2 (-84·7–47·8)	3 (1·1–6·7)	1·7 (0·6–3·8)	-41·1 (-77·8–79·2)	2·7 (1–6·4)	1·4 (0·5–3·2)	-47·8 (-81·3–66·9)
Nicaragua	4·3 (1·4–10·4)	2·1 (0·7–5·3)	-51·2 (-82·8–52·8)	5·5 (1·9–13·2)	3·2 (1·1–7·1)	-41·5 (-78·7–79·2)	4·9 (1·6–11·8)	2·7 (0·9–6·3)	-45·8 (-80·3–72·4)
Panama	4·8 (1·6–11·5)	2·8 (0·8–6·2)	-42·7 (-79·6–90·6)	5·8 (2·1–13·5)	4 (1·4–9)	-31·6 (-74·3–106·9)	5·3 (1·8–12·5)	3·4 (1·1–7·6)	-36·8 (-77·3–98·8)
Venezuela (Bolivarian Republic of)	11·4 (3·7–26·8)	8·1 (2·2–20·2)	-28·5 (-75·3–136·2)	28·8 (11–63·2)	23·9 (7·5–52·8)	-16·8 (-70·7–154·7)	19·9 (7·4–44·7)	15·6 (4·5–34·4)	-21·7 (-72·6–155·1)
Tropical Latin America	11·3 (3·6–26·7)	4·7 (1·5–10·9)	-57·9 (-84·7–39·8)	23·4 (8·9–51·5)	13·9 (5–30·1)	-40·5 (-78·2–81·7)	17·2 (6–38·5)	9·1 (3–19·5)	-46·8 (-80·8–64·7)
Brazil	11·1 (3·6–26·2)	4·6 (1·5–10·5)	-58·5 (-84·8–38·3)	22·9 (8·7–50·2)	13·4 (4·7–28·9)	-41·6 (-78·6–78·4)	16·8 (5·9–37·6)	8·8 (2·9–18·7)	-47·7 (-81·1–62·1)
Paraguay	19·4 (6·7–45·3)	11·5 (3·6–25·4)	-40·6 (-79·1–93·8)	43·1 (16·2–98·3)	38·9 (14·1–82·5)	-9·8 (-67·7–164)	31·2 (11·1–71·1)	25 (8·5–52·1)	-19·7 (-72·3–138·3)
North Africa and Middle East	25·8 (9·5–53)	9·1 (4·6–18)	-64·9 (-83·3–-21·9)	61·6 (26·5–128)	25·6 (12·9–49)	-58·4 (-78·5–-9·8)	44·1 (18·3–88·4)	17·7 (8·9–32·2)	-59·8 (-79·1–-17·1)
Afghanistan	25·1 (8·1–57)	13·9 (4·5–33·1)	-44·7 (-82·3–74·7)	51·7 (17–126)	31·8 (10·9–75·1)	-38·4 (-79·3–114·9)	37·6 (12·3–89·6)	22·9 (8·2–51·7)	-39·1 (-80·2–100·4)
Algeria	18·6 (6·1–43·5)	7·4 (2·3–17·5)	-60·3 (-87·2–19·6)	84 (28·7–193)	45·1 (15–104)	-46·3 (-81·3–68)	51·4 (17·3–118)	26·3 (9·1–59·8)	-48·8 (-82·1–54·9)
Bahrain	12·3 (4·1–29·4)	2·5 (0·8–6·3)	-79·3 (-93·3–-32·2)	23·5 (7·9–56·1)	7·6 (2·4–17·3)	-67·5 (-88·9–-2·1)	19·1 (6·1–42·9)	5·9 (1·7–12·1)	-69·3 (-90–-8·5)
Egypt	7·9 (2·4–19·9)	4·7 (1·5–11·3)	-41·1 (-83·4–83·9)	34 (10·6–82)	21·2 (6·6–48·1)	-37·8 (-80·4–111·6)	21·3 (6·7–49·6)	13·2 (4·1–29·7)	-37·9 (-79·7–97·1)

	Female			Male			Both		
	Age-standardized DALY rate (per 100,000)		Change (%)	Age-standardized DALY rate (per 100,000)		Change (%)	Age-standardized DALY rate (per 100,000)		Change (%)
	1990	2023		1990	2023		1990	2023	
Iran (Islamic Republic of)	14·5 (4·7–34·1)	3 (1–7·8)	-79·1 (-92·9–-37·9)	26·8 (8·5–64·9)	7·6 (2·5–17·4)	-71·8 (-91–-8·1)	20·8 (6·7–50·2)	5·3 (1·7–12·2)	-74·4 (-91·7–-18)
Iraq	8·5 (2·8–20·9)	4·5 (1·4–10·7)	-47·1 (-83·2–66·2)	15·4 (4·8–37·6)	10·4 (3·4–27·2)	-32·6 (-79·2–145·6)	12·1 (3·8–28·4)	7·5 (2·4–18·2)	-37·6 (-79·8–114·2)
Jordan	6 (1·9–14·6)	1·9 (0·6–4·7)	-67·8 (-89·3–7·4)	11·4 (3·3–27·6)	3·3 (1·1–7·9)	-70·6 (-91·7–0)	8·9 (2·8–21·1)	2·7 (0·9–6·4)	-69·5 (-90·5–1)
Kuwait	4·5 (1·5–10·4)	1·5 (0·5–3·7)	-65·4 (-87·9–11·7)	8·9 (3·2–20)	3·6 (1·2–7·9)	-60·1 (-85·5–15·5)	7·1 (2·5–16·1)	2·8 (0·9–6·2)	-60·1 (-86·3–16·2)
Lebanon	4·5 (1·3–11)	2·2 (0·7–5·1)	-51·1 (-84·3–69·6)	10·6 (3·1–25·9)	6·5 (2·2–14·8)	-38·4 (-82·1–120·3)	7·5 (2·3–18·4)	4·3 (1·4–9·6)	-42·1 (-82–97·7)
Libya	8·6 (2·8–19·7)	5·8 (1·8–13·1)	-33·3 (-80·4–127·7)	20·3 (6·5–49·5)	11·2 (3·4–25·9)	-45 (-82·8–76·7)	14·9 (4·8–34·1)	8·6 (3–19·2)	-42·7 (-80·7–85·9)
Morocco	13·3 (4·2–31·2)	7·5 (2·3–17·4)	-43·9 (-80·9–77·1)	14·2 (4·2–35·8)	8·5 (2·5–20·7)	-40·1 (-80·5–89·1)	13·7 (4·3–31·9)	8 (2·6–18·1)	-41·9 (-80·1–85·1)
Oman	10·3 (3·2–24·7)	4·5 (1·4–10·6)	-55·9 (-86·7–38·4)	23 (7·3–54·2)	9·5 (3·1–21·9)	-58·4 (-85·2–47·9)	18·5 (5·4–39·5)	7·8 (2·3–16·1)	-57·7 (-85·8–35·4)
Palestine	12·6 (4·1–31·8)	3·5 (1·1–8·6)	-71·8 (-91·1–-7)	29·6 (8·9–69·1)	8·7 (2·9–19·9)	-70·6 (-90·3–-4·9)	21 (6·6–50·2)	6·2 (2–13·8)	-70·6 (-90·3–-4·4)
Qatar	5·2 (1·7–11·9)	1·2 (0·4–3)	-76·4 (-92·6–-27·2)	6·2 (1·9–14·9)	2·4 (0·8–5·6)	-61 (-87–23·2)	5·9 (2–14·6)	2·1 (0·7–4·5)	-64·7 (-89·1–-0·2)
Saudi Arabia	12·5 (4·2–29)	6 (1·7–14·4)	-52·3 (-83·7–57·2)	14·8 (4·6–34·8)	6·4 (2–13·8)	-56·9 (-86·6–39·8)	13·9 (4·5–32·7)	6·2 (2–14·3)	-55·1 (-85·6–42·7)
Sudan	20 (6·3–45·5)	8·3 (2·6–21·2)	-58·3 (-86·7–37·7)	130 (38·6–322)	69·9 (22·3–170)	-46·2 (-81·2–58·8)	74·1 (24·1–186)	39·5 (12·5–94·7)	-46·7 (-82·3–52·2)
Syrian Arab Republic	4·7 (1·5–11·6)	2·3 (0·6–6·1)	-51·1 (-85·2–65·6)	6·2 (1·8–14·9)	3·3 (1–7·4)	-47·1 (-82·4–89·8)	5·5 (1·8–13·3)	2·8 (0·9–6·6)	-49·3 (-84–72·2)
Tunisia	46·2 (15·3–100)	13·7 (4·2–30)	-70·3 (-89·8–-7·7)	92·3 (29·9–208)	32·2 (11·3–74·2)	-65·1 (-88–12·6)	69·4 (22·5–156)	22·8 (8–50·4)	-67·1 (-87·8–3·1)
Türkiye	12·7 (4·3–29·8)	3·7 (1·1–9·2)	-70·8 (-91·1–-1·9)	17 (5·5–40·3)	5·9 (2–13·6)	-65·2 (-88·5–14·1)	14·9 (5–35·9)	4·8 (1·5–11)	-67·7 (-89·5–5)
United Arab Emirates	3·5 (1·1–9)	1·2 (0·4–3·1)	-64·4 (-89·1–35·4)	9·2 (2·4–25·7)	3·7 (1·2–8·3)	-59·7 (-87–72·3)	7·6 (1·9–17·7)	3·1 (0·9–6·4)	-58·8 (-85·8–79·1)
Yemen	450 (170–892)	143 (69·9–269)	-68·1 (-85–-25)	1100 (546–1980)	340 (158–643)	-69 (-86–-32·3)	773 (359–1390)	241 (121–426)	-68·8 (-84·9–-29·1)
South Asia	419 (221–740)	349 (189–565)	-16·6 (-55·5–45·9)	621 (332–970)	611 (402–903)	-1·6 (-41·9–70·6)	524 (284–836)	482 (297–722)	-8 (-46·6–56)

	Female			Male			Both		
	Age-standardized DALY rate (per 100,000)		Change (%)	Age-standardized DALY rate (per 100,000)		Change (%)	Age-standardized DALY rate (per 100,000)		Change (%)
	1990	2023		1990	2023		1990	2023	
Bangladesh	958 (518–1540)	1080 (657–1580)	13 (-33·3–81·1)	645 (321–991)	769 (556–1010)	19·2 (-22·6–110·5)	797 (432–1200)	929 (623–1230)	16·6 (-22·9–95·3)
Bhutan	372 (163–703)	277 (140–469)	-25·5 (-66·3–70·1)	466 (198–860)	409 (235–675)	-12·3 (-52·9–72·9)	423 (194–734)	346 (196–549)	-18·2 (-56·2–60·9)
India	386 (198–707)	296 (151–504)	-23·2 (-61·5–44·4)	664 (358–1040)	626 (394–944)	-5·7 (-45–66·3)	531 (284–852)	464 (273–706)	-12·6 (-50·9–53·4)
Nepal	271 (118–516)	174 (79·5–325)	-36 (-70·4–36·1)	598 (301–950)	641 (427–942)	7·1 (-36·9–84·4)	432 (221–725)	397 (256–595)	-8·2 (-45–62·9)
Pakistan	254 (110–493)	264 (115–484)	3·7 (-56·3–165·7)	304 (146–580)	344 (177–651)	13·4 (-52·4–134·6)	280 (135–515)	305 (154–555)	8·9 (-51·3–150·4)
**Southeast Asia, East Asia, and Oceania**	47·5 (17·7–97·5)	21·6 (9·4–44·3)	-54·5 (-81·3–19·1)	58·7 (22·7–133)	40·1 (16–83)	-31·7 (-73·5–88·6)	53·2 (20·7–116)	30·9 (12·4–62·7)	-41·8 (-76·5–54·1)
East Asia	14·1 (4·6–32·2)	4·9 (1·5–11·6)	-65·1 (-88·4–9·9)	40·6 (15–95·1)	22·4 (8·1–49)	-44·8 (-80·2–70·3)	27·7 (10–64)	13·8 (4·7–29·8)	-50·2 (-82·5–54·7)
China	14·4 (4·7–32·8)	5 (1·5–11·6)	-65·4 (-88·5–9·1)	41·5 (15·3–97·1)	22·6 (8·1–49·3)	-45·4 (-80·4–68)	28·3 (10·3–65·5)	13·9 (4·7–30·1)	-50·7 (-82·7–52·7)
Democratic People's Republic of Korea	5·1 (1·7–11·4)	4·7 (1·4–12·2)	-7·9 (-69–178)	9·2 (3·1–22·3)	7·8 (2·5–18·1)	-15·8 (-73·6–187·4)	7·1 (2·2–15·3)	6·2 (1·9–13·8)	-12·8 (-68·5–179·2)
Taiwan	7·5 (2·4–18)	2·2 (0·7–5·2)	-70·8 (-89·1–-7·7)	23·3 (8·8–49·4)	23·3 (8·9–51·6)	-0·1 (-62·8–203·5)	15·7 (5·8–34·7)	12·5 (4·6–27·7)	-20·1 (-70·7–143·2)
Oceania	44·8 (16·2–108)	67·6 (27·3–133)	50·8 (-41·7–312)	63·9 (24·9–148)	59·5 (20·6–138)	-6·9 (-66·4–184·5)	54·7 (20·6–116)	63·4 (23·1–131)	16 (-53·7–222·9)
American Samoa	16·9 (5·7–38·4)	15·8 (5·1–36·3)	-6·3 (-70·5–200)	51·5 (17·4–121)	44·9 (16·1–107)	-12·9 (-70·2–190·2)	34·5 (11·6–81)	30·6 (11–69·7)	-11·4 (-68·5–179·3)
Cook Islands	18·1 (5·9–41·3)	10·7 (3·6–25·2)	-40·6 (-80·1–96·6)	45·5 (14·3–110)	42·2 (15·8–101)	-7·3 (-69·1–191·8)	32·3 (10·5–77·2)	25·9 (9·6–61·1)	-19·7 (-72·7–147·1)
Fiji	18·4 (6·5–42·5)	21 (6·3–47·1)	13·8 (-61·7–251·2)	43·6 (14·4–97·7)	38 (12·8–84·8)	-12·7 (-67·6–169·8)	31·1 (10·6–71)	29·6 (9·4–63·8)	-4·9 (-65·4–193·3)
Guam	8·7 (3–19·5)	17·4 (7·3–35·9)	99·6 (-20·6–469·3)	47·3 (19·4–98·3)	103 (55·7–179)	118·3 (-1·1–409·5)	29·6 (12–62)	60·6 (31·9–109)	104·7 (-8·7–399·4)
Kiribati	17·2 (5·6–36·6)	25·2 (8·3–57)	46·9 (-51·8–332·5)	136 (52·6–297)	142 (58·9–304)	4·6 (-59–188·7)	74·6 (28–159)	81·6 (33·1–169)	9·5 (-56·7–198·2)

	Female			Male			Both		
	Age-standardized DALY rate (per 100,000)		Change (%)	Age-standardized DALY rate (per 100,000)		Change (%)	Age-standardized DALY rate (per 100,000)		Change (%)
	1990	2023		1990	2023		1990	2023	
Marshall Islands	30·1 (9·6–71·5)	62·9 (24·1–148)	108·6 (-28·4–482·3)	108 (41·3–231)	126 (54·3–268)	16·4 (-56·8–251·1)	70·1 (26·7–160)	95 (40·9–197)	35·6 (-46·8–289·6)
Micronesia (Federated States of)	32·9 (11·1–76·2)	28·3 (8·9–64·8)	-13·8 (-72·6–163·3)	147 (54·6–324)	91 (34·4–213)	-38 (-77·3–93·9)	90·6 (32·4–200)	60·1 (22·5–133)	-33·7 (-76·2–102·2)
Nauru	19·8 (5·7–48·3)	24·1 (7·3–55·8)	21·9 (-62·4–291·6)	32·5 (9–85·7)	30·1 (10·4–70·7)	-7·2 (-71–202·8)	26·3 (8–68·6)	27·1 (9·5–60·7)	3·2 (-64·7–243·6)
Niue	15·9 (5·3–35·3)	15·6 (5·3–35·4)	-1·9 (-67·3–228·6)	27·8 (8·6–64·2)	24·9 (8·1–54·7)	-10·4 (-68·4–175·8)	21·7 (6·9–51·7)	20 (6·8–45·4)	-7·9 (-67·8–180·4)
Northern Mariana Islands	23·3 (8–53·3)	28·4 (10·7–59·6)	22·2 (-56·7–279)	68·8 (25·1–154)	96·2 (37·6–205)	39·8 (-49·5–296)	47·6 (17·4–112)	63·9 (25·3–136)	34 (-51·8–273·1)
Palau	352 (163–635)	410 (212–698)	16·4 (-45·6–145·7)	642 (291–1200)	543 (268–979)	-15·4 (-62·3–92·1)	502 (235–923)	483 (252–802)	-3·9 (-55·2–104·8)
Papua New Guinea	55·7 (19·5–140)	78·1 (32·1–159)	40·1 (-45·8–288·3)	58·2 (21·1–132)	51·5 (16–122)	-11·6 (-69·3–205·5)	57 (21·1–123)	64·3 (23·6–134)	12·8 (-56·8–210·4)
Samoa	12·4 (3·8–28·4)	13·9 (4·4–34·4)	12·1 (-65·8–259·3)	23·5 (8·5–53·7)	22·5 (7·3–54·8)	-4·2 (-67·8–246·9)	18·2 (6·1–41·1)	18·2 (6·1–44·4)	0 (-65·3–227)
Solomon Islands	39·9 (11·8–93·9)	64·5 (20·2–142)	61·5 (-46·8–433·9)	138 (46·7–310)	151 (55·4–337)	9·6 (-57·2–242·7)	90·6 (33–214)	108 (37·9–230)	19·8 (-55·7–253·6)
Tokelau	13·6 (4·2–30·4)	14·8 (4·9–34·3)	8·8 (-64·1–262·8)	41·7 (14–94·9)	33·4 (11·3–74·9)	-19·8 (-71–175)	27·3 (8·8–64·3)	24·2 (8·3–55·3)	-11·5 (-67·9–183·4)
Tonga	8·7 (2·8–19·2)	10·1 (3·2–23·3)	15·1 (-64·7–273·4)	15·9 (5·6–38·5)	13·1 (4·3–31·1)	-17·6 (-73·2–159·3)	12·2 (4·1–28)	11·4 (3·9–26·5)	-6·5 (-67·2–189·2)
Tuvalu	20·1 (6·4–46·5)	18·1 (5·8–42)	-10 (-74·3–194·8)	50·1 (16–121)	38·7 (11·8–86·3)	-22·8 (-73·2–176·4)	33·7 (10·7–81·1)	28·6 (9·2–60·9)	-15·2 (-71·1–176·3)
Vanuatu	22·2 (6·8–50·6)	30·8 (9·8–75·6)	38·7 (-55–324·1)	83·7 (27·8–199)	83·6 (28·7–189)	-0·1 (-64·2–232·2)	53·6 (18–136)	57 (19·8–129)	6·5 (-63·3–226·9)
Southeast Asia	155 (60·9–310)	75·4 (33·7–150)	-51·4 (-79·2–19·5)	119 (47·2–264)	92·2 (39·7–183)	-22·6 (-67–95·9)	138 (55·1–279)	83·7 (37·5–164)	-39·1 (-74·2–46·1)
Cambodia	612 (289–1100)	377 (187–688)	-38·4 (-71·1–33·4)	85·6 (31·8–220)	53·4 (20·3–130)	-37·6 (-78·7–101·5)	374 (183–687)	222 (135–427)	-40·5 (-64·2–25·7)

	Female			Male			Both		
	Age-standardized DALY rate (per 100,000)		Change (%)	Age-standardized DALY rate (per 100,000)		Change (%)	Age-standardized DALY rate (per 100,000)		Change (%)
	1990	2023		1990	2023		1990	2023	
Indonesia	161 (58·6–334)	62·7 (21·4–142)	-61·1 (-85·7–7·1)	26 (8·9–61·3)	19·6 (6·7–46·5)	-24·6 (-72·3–143·2)	94·8 (36·5–200)	41 (14·8–97·7)	-56·8 (-83·4–20·3)
Lao People's Democratic Republic	333 (123–727)	329 (146–621)	-1·2 (-61·1–153·7)	32·8 (10·3–84·8)	26·6 (8·8–63·3)	-19·1 (-74–184·4)	190 (70·8–409)	177 (78·3–346)	-6·5 (-62·9–145·6)
Malaysia	49·2 (16·6–118)	30·7 (11·1–71·1)	-37·5 (-79·3–92)	59·8 (21·1–138)	60·9 (22·1–132)	1·8 (-63·4–224)	54·5 (18·3–130)	46·6 (16·6–102)	-14·5 (-70·1–160·5)
Maldives	284 (97·3–628)	100 (34·5–212)	-64·6 (-87·1–7·1)	265 (107–571)	117 (50·6–233)	-55·9 (-81·4–4·1)	274 (103–572)	111 (44–211)	-59·5 (-83·7–5·3)
Mauritius	84·3 (30·3–172)	46·5 (15·4–103)	-44·8 (-78·9–76·3)	52·8 (20·2–114)	38·9 (13·7–85·8)	-26·3 (-70·9–117)	68·6 (26·4–155)	42·8 (15·2–97·5)	-37·7 (-76·6–86)
Myanmar	400 (150–813)	269 (94·3–551)	-32·9 (-75·6–84·9)	710 (284–1500)	710 (324–1370)	0 (-57·3–142·1)	551 (216–1120)	477 (201–893)	-13·4 (-63·9–108·8)
Philippines	40·8 (14·7–89·5)	27·1 (9–61·2)	-33·5 (-76·5–91·4)	76·6 (27·3–175)	80·3 (31·7–173)	4·9 (-58·1–209·8)	58·6 (20·3–132)	53·9 (18·9–105)	-8·1 (-66·7–140·5)
Seychelles	58·2 (19·7–122)	31·5 (11·1–70·9)	-45·9 (-81·2–60·6)	69·2 (26–160)	52·1 (18·5–115)	-24·8 (-74·5–139)	63·6 (24·5–144)	42·9 (15·9–97·4)	-32·5 (-76·1–100·3)
Sri Lanka	140 (51·5–290)	93·1 (32·3–194)	-33·3 (-75·1–84·3)	404 (172–773)	345 (161–617)	-14·6 (-61·1–92·4)	272 (113–529)	213 (91·6–384)	-21·7 (-66·7–79·9)
Thailand	134 (52·5–271)	52·1 (19·3–108)	-61·2 (-84·9–1·4)	60·2 (21·7–144)	41·1 (15·3–92)	-31·7 (-75·2–103·6)	97·9 (38·9–208)	46·9 (17·5–102)	-52·1 (-82·5–26·8)
Timor-Leste	176 (63·9–376)	107 (35–241)	-39·4 (-80·1–90·4)	56·6 (19·8–145)	41 (13·3–92·1)	-27·5 (-75·2–138·4)	115 (42·4–258)	73·4 (25–158)	-36·1 (-78·1–86·8)
Viet Nam	87 (29·4–202)	38·8 (13–85·8)	-55·4 (-82·8–29)	39·6 (13·1–89·1)	21·6 (7·9–48)	-45·4 (-79·9–70)	64·8 (23·8–161)	30·4 (11·4–74·3)	-53·1 (-82·2–37·9)
**Sub-Saharan Africa**	35·9 (12·9–74·7)	29·2 (10·7–65·4)	-18·8 (-68·8–129·2)	44·9 (19·2–95·1)	38·3 (16·6–82·2)	-14·7 (-66·2–120·8)	40·3 (16–86·3)	33·6 (13·4–70·1)	-16·7 (-67·8–109)
Central Sub-Saharan Africa	32·4 (11·2–72·4)	20·4 (6·5–49·9)	-37 (-77·9–102)	26 (8·1–60·1)	25·9 (8·8–63)	-0·3 (-67·4–232)	29·2 (10·2–65·5)	23·1 (7·9–52·8)	-21 (-71·1–144·4)
Angola	10·1 (3·3–22·3)	7·4 (2·4–17·2)	-26·8 (-75·7–132·8)	15·5 (4·8–38·3)	13·1 (4–32·2)	-15·1 (-71·3–193·5)	12·8 (4·3–31·3)	10·1 (3·2–22·8)	-21·2 (-71·1–157)

	Female			Male			Both		
	Age-standardized DALY rate (per 100,000)		Change (%)	Age-standardized DALY rate (per 100,000)		Change (%)	Age-standardized DALY rate (per 100,000)		Change (%)
	1990	2023		1990	2023		1990	2023	
Central African Republic	37·6 (12·5–87·2)	38·9 (10·9–89·5)	3·3 (-67·5–240·2)	19·8 (5·6–47·1)	17·7 (6–41·2)	-10·6 (-69·9–212·4)	29 (9·9–70·9)	28·6 (9·4–64·7)	-1·6 (-66–218·9)
Congo	28·6 (9·8–62·9)	25·6 (8·4–60·3)	-10·3 (-69·3–200·3)	12·3 (3·9–27·9)	13 (4·3–31·2)	6·4 (-63·8–270·4)	20·7 (7·4–48·2)	19·4 (6·9–47·8)	-6·2 (-65·7–204·2)
Democratic Republic of the Congo	39·6 (13·8–87·7)	24·2 (7·2–59·4)	-39·1 (-78·7–91·3)	31 (9·7–72·6)	32·4 (11·5–81·3)	4·4 (-66·2–242·4)	35·5 (12·3–77·3)	28·3 (9·6–65·1)	-20·2 (-71·4–148·7)
Equatorial Guinea	28·4 (9·5–62·5)	14·9 (4·9–33)	-47·5 (-82·8–79·3)	23·9 (7·5–59·2)	18·5 (6·1–46·8)	-22·7 (-75·3–181·3)	26·3 (8·9–61·8)	16·8 (5·5–38·8)	-36·1 (-77·8–111·3)
Gabon	9·3 (3·2–21·2)	7 (2·2–16)	-24·6 (-75·1–145·1)	5 (1·6–12)	4·7 (1·6–11)	-4·4 (-67·5–234·8)	7·2 (2·4–17·2)	5·9 (1·9–14)	-17·2 (-71·5–157·4)
Eastern Sub-Saharan Africa	43·6 (17·2–89·9)	36·8 (14·9–78·7)	-15·8 (-66–119·7)	80·5 (35·6–163)	66·8 (31–138)	-17·1 (-65·6–95)	61·6 (27·2–126)	51·4 (22·8–103)	-16·6 (-66·5–96)
Burundi	51·9 (18·6–113)	51·1 (18–104)	-1·6 (-65·8–223·8)	134 (50·5–279)	99·8 (39·1–247)	-25·7 (-73·6–121·8)	91·1 (32·8–184)	75·7 (28·1–174)	-17 (-69·6–155·9)
Comoros	85·6 (33·2–178)	45·5 (17·6–97)	-46·8 (-79·9–37·2)	125 (52·2–263)	81·7 (33·7–169)	-34·7 (-74·2–77·1)	105 (42·9–221)	63·6 (24·8–127)	-39·3 (-76·3–53·2)
Djibouti	27·4 (9–62·3)	17·9 (6·1–38·7)	-34·6 (-77·4–93·4)	44·2 (17·5–96·9)	29·9 (10–65·4)	-32·3 (-74–106·5)	36·2 (13·7–86·6)	24·4 (8·2–53)	-32·7 (-75·2–103·2)
Eritrea	8·6 (2·7–19·6)	8·2 (2·7–20·1)	-4·4 (-68·1–208·2)	103 (35·7–239)	86·2 (31·7–191)	-16·7 (-70·3–151)	54·1 (18–115)	48·2 (16·6–99·7)	-10·9 (-68·1–160·7)
Ethiopia	7·8 (2·4–19)	10·3 (3·7–21·9)	32 (-58·7–365·2)	42·8 (15·9–95·3)	56·2 (22·2–133)	31·4 (-56·3–334·4)	25·1 (9·9–60·1)	33·1 (13·1–79·4)	32 (-56·7–294·3)
Kenya	30·7 (11·3–65·5)	22·3 (7·8–48·2)	-27·3 (-73·7–123·1)	39·8 (15·8–90·6)	29·9 (10·2–69·1)	-25 (-73·3–116)	35·2 (14–77)	26 (8·8–57·2)	-26 (-73·6–128·5)
Madagascar	250 (105–475)	211 (99·8–411)	-15·3 (-61·9–100·5)	680 (323–1280)	444 (236–812)	-34·7 (-67·4–40·6)	462 (219–873)	324 (173–570)	-29·8 (-66·5–49·1)
Malawi	60·3 (21·1–130)	43·5 (16·2–96·7)	-27·9 (-73·1–110·6)	30·4 (10·7–73·6)	26·7 (8·8–62·6)	-12·1 (-69·3–188·6)	45·9 (16·8–102)	35·5 (12·6–83·3)	-22·7 (-70·9–137·2)
Mozambique	29·1 (10·1–65·1)	20·6 (6·6–46·3)	-29·2 (-75·6–115·7)	37·2 (12·5–89·6)	33·4 (11·3–79·1)	-10·1 (-70·4–155·4)	32·8 (11·1–72·9)	26·6 (8·8–59·6)	-19 (-72–131·2)

	Female			Male			Both		
	Age-standardized DALY rate (per 100,000)		Change (%)	Age-standardized DALY rate (per 100,000)		Change (%)	Age-standardized DALY rate (per 100,000)		Change (%)
	1990	2023		1990	2023		1990	2023	
Rwanda	98·1 (35·7–207)	61·1 (22·1–125)	-37·7 (-76–89)	19·8 (6·6–46·3)	14·9 (5·3–33·7)	-24·6 (-73·7–152·1)	60·7 (23–134)	39·1 (14·6–82·9)	-35·6 (-74·8–93·8)
Somalia	14·1 (4·4–35·5)	15·7 (5·4–36·1)	11·7 (-61·7–262)	21·4 (7·6–48·4)	20·9 (6·8–51·9)	-2·2 (-66–232·6)	17·8 (6·3–43·2)	18·4 (6·1–41·8)	2·8 (-64·3–244·1)
South Sudan	40 (14·7–85·3)	39·8 (13·9–87·5)	-0·4 (-64·9–187·7)	68·7 (24·2–166)	55·3 (19·2–127)	-19·4 (-71·8–158·5)	55·3 (21·2–132)	47·4 (16·3–105)	-14·3 (-71–155·7)
Uganda	47·8 (17·4–102)	40·2 (13·7–94·7)	-15·8 (-68·7–168)	21·6 (7·8–46·1)	22·7 (7·9–53·3)	5 (-64·9–224·2)	35·1 (13·4–71·7)	31·8 (11·5–76·9)	-9·4 (-66·6–181·3)
United Republic of Tanzania	20·5 (7–46)	14·9 (4·8–33·7)	-27·3 (-75·1–126·3)	25·4 (9·1–58·3)	21·3 (7·5–47·7)	-16·2 (-70·2–153·3)	22·8 (8·3–51·7)	18 (6·1–39·7)	-21·2 (-71·7–151·1)
Zambia	33·1 (9·9–82·4)	37·4 (12·2–86·6)	13 (-64·2–279·7)	24·2 (8·7–55·4)	28·3 (9·5–66·2)	16·9 (-57·9–308·3)	28·8 (9·5–63·9)	33·1 (11·6–74·6)	14·9 (-60·4–257·4)
Southern Sub-Saharan Africa	41·7 (14·6–87)	35·6 (11·8–82·6)	-14·6 (-69–147)	19·1 (7·3–43·7)	13·9 (4·8–29·9)	-27·2 (-74·4–123·2)	30·9 (11·8–70·7)	25·2 (9·3–64·2)	-18·5 (-69–136·6)
Botswana	136 (56·3–268)	82·7 (34·5–162)	-39·1 (-75·8–66·1)	50·3 (18·4–114)	46·9 (17·6–104)	-6·7 (-65·2–178·1)	96·1 (39·1–202)	65·5 (27·7–136)	-31·9 (-72·7–77·6)
Eswatini	21·9 (7·5–47·4)	17·4 (5·9–38·2)	-20·5 (-74·7–169·3)	50·8 (18·8–119)	25·6 (8·8–61·7)	-49·6 (-82·6–62·9)	35 (13·2–81·6)	21·4 (7·2–45·1)	-38·8 (-78·4–89·3)
Lesotho	38·9 (12·7–85·9)	36·6 (11·9–88·5)	-6 (-66·8–190·7)	32·8 (11·6–75)	29 (9·4–62·1)	-11·7 (-69·6–183·3)	36·4 (12·8–83·4)	32·9 (12·4–83·6)	-9·5 (-67·9–193·9)
Namibia	50 (17·4–109)	33·4 (10·4–71·3)	-33·1 (-75·2–93·3)	31·6 (10·5–76·3)	19·7 (7–44·8)	-37·5 (-78·6–100·7)	41·1 (15·4–100)	26·8 (9·8–60·2)	-34·8 (-75·4–82·4)
South Africa	43·7 (15·2–90·4)	35 (11·3–80·1)	-20 (-70·9–134·5)	18·6 (7·1–42·3)	12·7 (4·4–27·5)	-31·6 (-76·6–112·6)	31·8 (12·2–72·4)	24·3 (8·9–63·3)	-23·4 (-70·9–132·3)
Zimbabwe	19·1 (6·3–40·4)	32 (10·4–69)	66·9 (-41–416·1)	11·1 (4–25·2)	10·2 (3·6–25·5)	-8·1 (-68·1–198)	15·3 (5·7–36·8)	21·8 (7·8–50·8)	42·5 (-50·4–335·2)
Western Sub-Saharan Africa	26 (8·4–56·6)	22·1 (7·7–50·2)	-15·1 (-72·1–165)	26·1 (9·5–61)	23 (9–54·4)	-11·6 (-69·7–175·4)	26 (9·2–61·8)	22·5 (8·2–49·7)	-13·5 (-67·8–155·2)
Benin	33·5 (10·8–71·7)	24·3 (8·3–55·4)	-27·4 (-74·7–124·3)	26·8 (9·5–66·6)	18·7 (6·3–43·4)	-30·2 (-77·7–121·4)	30·4 (9·9–67·9)	21·6 (7·5–49·4)	-29 (-72·5–113)
Burkina Faso	28·7 (9·7–61·9)	24·6 (8·6–52·6)	-14·3 (-71·2–157·9)	38·6 (13·9–90·4)	25 (9·3–58·5)	-35·3 (-78·8–113)	33·2 (11·8–74·6)	24·8 (9·2–53·2)	-25·5 (-72·6–116·9)

	Female			Male			Both		
	Age-standardized DALY rate (per 100,000)		Change (%)	Age-standardized DALY rate (per 100,000)		Change (%)	Age-standardized DALY rate (per 100,000)		Change (%)
	1990	2023		1990	2023		1990	2023	
Cabo Verde	11·5 (3·9–24·9)	6·2 (2–13·7)	-46·4 (-81·7–75·6)	10·1 (3·5–24·7)	8·6 (2·8–18·3)	-15·5 (-71·4–167·2)	10·9 (3·6–24·9)	7·4 (2·4–16·4)	-32·3 (-76·1–125·8)
Cameroon	84·7 (27·7–221)	83·2 (30·5–184)	-1·7 (-67·6–191)	25·3 (8·8–56·2)	22·6 (7·6–54·8)	-10·7 (-72·3–199·5)	56·4 (18·3–140)	53·3 (19·9–123)	-5·4 (-67·4–180·9)
Chad	54·5 (17·8–114)	60 (19·2–144)	10·2 (-62·8–268·4)	62·2 (22·4–149)	63·7 (24·1–146)	2·4 (-64·7–214·8)	58·1 (19·7–124)	61·8 (22·2–140)	6·4 (-60·4–228·7)
Côte d'Ivoire	26·8 (8·9–62·9)	17·2 (5·8–37)	-36 (-79–95·1)	10·2 (3·2–23·7)	9·5 (3·3–22·9)	-6·5 (-68·4–204·3)	18·3 (5·9–42·9)	13·1 (4·5–31·1)	-28·1 (-74–136·5)
Gambia	18·8 (6·5–41·3)	20·4 (6·3–48·5)	8·8 (-66·9–265·2)	13·3 (4·2–33·4)	11·8 (4–30·3)	-11·5 (-72–200·8)	16·1 (5·1–37·7)	16·2 (5·7–39·3)	1 (-64·7–233)
Ghana	24·1 (7·6–54·7)	19·3 (5·9–48)	-20 (-72·6–191·2)	51·2 (15·8–124)	39·9 (12·7–98·3)	-21·9 (-73·1–160·7)	37·2 (11·9–86·4)	29·4 (9–67·9)	-21·1 (-71·9–155·9)
Guinea	27·1 (8·7–57·6)	23 (7·6–53)	-15·1 (-72·1–179·3)	31·2 (10–81·9)	24·2 (8·2–59·9)	-22·6 (-74·1–175·3)	29 (9·8–67)	23·5 (8·1–53·6)	-18·9 (-71·2–161·8)
Guinea-Bissau	29·5 (9·4–65·8)	31·3 (10·2–74·6)	6·3 (-64·3–243·7)	19·3 (6·1–47·4)	18·6 (5·9–44)	-3·5 (-67·2–218·1)	24·7 (7·8–57)	25·2 (8·7–60·8)	2·3 (-62·3–220·7)
Liberia	25·8 (8·4–57·2)	23·3 (7·5–58·4)	-9·4 (-71·8–192·5)	39·2 (12·5–101)	30·3 (10·4–71·4)	-22·6 (-75·7–165·7)	32·5 (10·7–81)	26·9 (9·4–64·5)	-17·3 (-72·2–173·9)
Mali	24·5 (7·6–53·5)	20·8 (6·4–49·3)	-15·2 (-71·6–171·8)	66 (27–150)	59·2 (23·7–133)	-10·3 (-68–153·5)	44·3 (17·4–107)	39·6 (15·5–94·5)	-10·5 (-66·5–158·7)
Mauritania	27·5 (9–62·5)	22·9 (7·1–57)	-16·6 (-73–148·6)	27·5 (9·8–65·9)	22·5 (7·5–56·5)	-18·2 (-75·2–159·7)	27·5 (9·4–66·3)	22·7 (7·6–52·6)	-17·4 (-72·9–138·8)
Niger	33·2 (10·3–75·5)	33·3 (10–85·2)	0·3 (-68·4–223·9)	103 (41–237)	90·9 (35·1–190)	-11·9 (-69·1–161·6)	67·3 (26·2–160)	61·3 (24·4–129)	-8·8 (-69·3–157·7)
Nigeria	14·8 (4·5–33·7)	10·7 (3·6–25·9)	-27·8 (-77·7–128·3)	11·5 (3·9–27)	9·4 (3·2–24·2)	-18·8 (-74·1–190)	13·1 (4·6–32·5)	10·1 (3·5–23)	-23·2 (-75·3–121)
Sao Tome and Principe	10·7 (3·5–23·1)	7·7 (2·3–19·1)	-27·7 (-77·2–135·6)	14·2 (4·6–33·9)	16·8 (5·6–39·7)	18·1 (-61·5–267·4)	12·4 (4–28)	12·3 (4–27·1)	-1 (-67·9–196·3)
Senegal	27·9 (8·9–65·1)	27·5 (8·8–63·3)	-1·4 (-66·2–214·4)	11·5 (3·8–29·4)	8·8 (3·1–21·6)	-23·7 (-75–150·9)	20·1 (6·4–44·9)	18·3 (6·2–43·4)	-8·8 (-66–204·5)
Sierra Leone	64·6 (22·9–153)	52 (16·4–114)	-19·5 (-72·4–149·2)	48·7 (15·6–110)	41·4 (13·4–97·6)	-14·9 (-71·7–175·3)	56·9 (19–136)	46·8 (15·7–102)	-17·8 (-69·6–153·9)

	Female			Male			Both		
	Age-standardized DALY rate (per 100,000)		Change (%)	Age-standardized DALY rate (per 100,000)		Change (%)	Age-standardized DALY rate (per 100,000)		Change (%)
	1990	2023		1990	2023		1990	2023	
Togo	48·5 (16–102)	38·4 (12·4–94·3)	-20·9 (-73·7–139·8)	37·3 (11·8–91·2)	25 (7·6–61·7)	-32·9 (-76·6–113·5)	43·2 (14·3–101)	32 (10·6–77·3)	-25·9 (-73·1–140·7)

Appendix Figure 2·5: DALY rates attributable to chewing tobacco use (per 100,000), by measure and region, among ages 15+, comparing 1990 and 2023

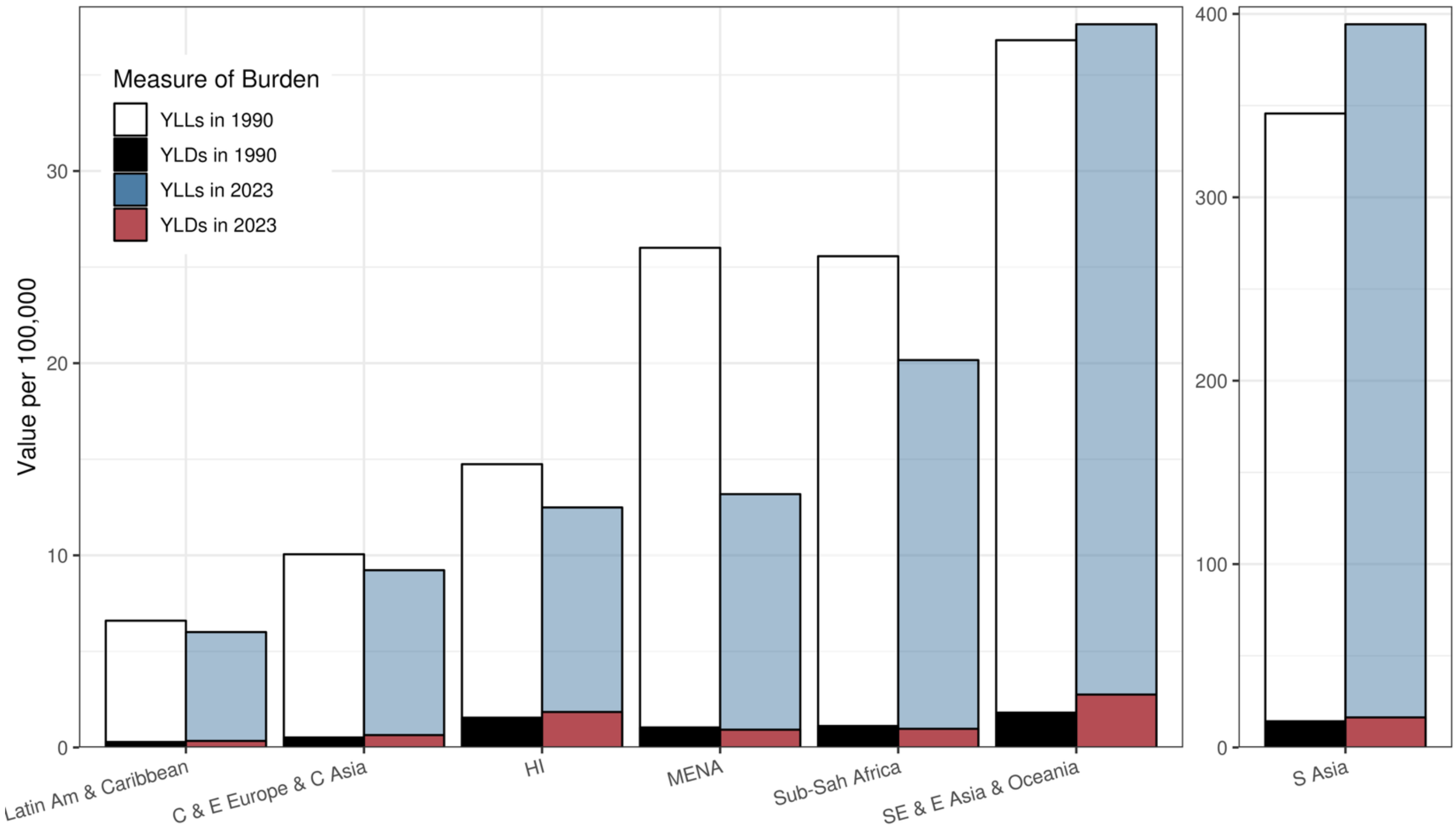

Return to Table of Contents

Appendix Table 2·14: Cause-specific DALY rates attributable to chewing tobacco use in 2023, among ages 15+, by super-region

	Cause	Male (per 100,000)	Female (per 100,000)	Both (per 100,000)
Global				
	Esophageal cancer	15·2 (9·1–25·1)	7·9 (3·9–14)	11·5 (7·8–20·2)
	Larynx cancer	10·7 (5·6–17·2)	1·3 (0·6–2·2)	6 (3·4–10·5)
	Lip and oral cavity cancer	24·7 (15·7–35·6)	10·1 (5·9–15·7)	17·4 (11·3–24·3)
	Nasopharynx cancer	3·8 (1·9–6·4)	1·3 (0·6–2·4)	2·6 (1·3–4·2)
	Other pharynx cancer	11·5 (5·9–18·5)	1·9 (0·9–3·5)	6·7 (3·8–11·6)
	Stroke	67·6 (36–116)	44·4 (20·8–78·8)	55·9 (29–92·9)
**Central Europe, Eastern Europe, and Central Asia**				
	Esophageal cancer	1 (0·4–2·1)	0·1 (0–0·3)	0·5 (0·2–1·2)
	Larynx cancer	0·9 (0·3–2·1)	0·1 (0–0·1)	0·5 (0·2–1·1)
	Lip and oral cavity cancer	1·9 (0·7–4)	0·3 (0·1–0·6)	1 (0·4–2·2)
	Nasopharynx cancer	0·2 (0·1–0·6)	0 (0–0·1)	0·1 (0–0·3)
	Other pharynx cancer	0·7 (0·2–1·5)	0·1 (0–0·1)	0·3 (0·1–0·8)
	Stroke	7·4 (2·5–16·5)	2·4 (0·7–5·7)	4·8 (1·5–10·5)
**High-income**				
	Esophageal cancer	3·2 (1·8–5·4)	0·2 (0·1–0·4)	1·6 (1–3·1)
	Larynx cancer	1 (0·5–1·8)	0 (0–0·1)	0·5 (0·3–1)
	Lip and oral cavity cancer	2·5 (1·5–3·9)	0·3 (0·1–0·6)	1·3 (0·8–2·1)
	Nasopharynx cancer	0·3 (0·1–0·6)	0 (0–0·1)	0·2 (0·1–0·3)
	Other pharynx cancer	0·9 (0·5–1·5)	0 (0–0·1)	0·5 (0·3–0·9)
	Stroke	7·3 (3·8–12·5)	1·2 (0·4–2·9)	4·2 (2–7·3)
**Latin America and Caribbean**				
	Esophageal cancer	1·2 (0·4–2·5)	0·2 (0·1–0·5)	0·7 (0·3–1·6)
	Larynx cancer	1 (0·3–2·2)	0·1 (0–0·2)	0·5 (0·2–1·3)
	Lip and oral cavity cancer	1·2 (0·5–2·6)	0·4 (0·1–0·8)	0·8 (0·3–1·6)
	Nasopharynx cancer	0·1 (0–0·3)	0 (0–0·1)	0·1 (0–0·2)
	Other pharynx cancer	0·4 (0·1–1)	0·1 (0–0·1)	0·2 (0·1–0·6)
	Stroke	4·8 (1·5–10·5)	2·7 (0·9–6·4)	3·7 (1·2–8·2)
North Africa and Middle East				
	Esophageal cancer	1·3 (0·6–2·3)	0·4 (0·2–0·8)	0·9 (0·5–1·7)
	Larynx cancer	2 (0·8–3·9)	0·2 (0·1–0·5)	1·1 (0·5–2·4)

	Cause	Male (per 100,000)	Female (per 100,000)	Both (per 100,000)
	Lip and oral cavity cancer	1 (0·5–2·1)	0·3 (0·1–0·6)	0·7 (0·3–1·3)
	Nasopharynx cancer	0·6 (0·2–1·3)	0·1 (0–0·3)	0·4 (0·1–0·8)
	Other pharynx cancer	0·2 (0·1–0·5)	0 (0–0·1)	0·1 (0·1–0·3)
	Stroke	20·5 (8·8–40·9)	8 (3·7–16)	14·5 (6·6–27·1)
South Asia				
	Esophageal cancer	64·7 (39·6–103)	42·3 (20·6–74·7)	53·7 (37–95·1)
	Larynx cancer	52·1 (27·4–84·5)	6·5 (2·9–10·7)	29·6 (16·7–52·3)
	Lip and oral cavity cancer	117 (73·8–168)	53·4 (31·8–82·3)	85·8 (55·8–121)
	Nasopharynx cancer	14·3 (7·4–23·8)	5·4 (2·3–9·6)	9·9 (5–15·8)
	Other pharynx cancer	58·1 (29·3–93·8)	10 (5–18·5)	34·4 (19·6–59·9)
	Stroke	304 (163–512)	232 (107–395)	269 (136–439)
**Southeast Asia, East Asia, and Oceania**				
	Esophageal cancer	5·4 (2–11·4)	1 (0·4–2·2)	3·2 (1·3–7·4)
	Larynx cancer	1·3 (0·4–2·4)	0·2 (0·1–0·4)	0·7 (0·3–1·6)
	Lip and oral cavity cancer	3·2 (1·4–6)	1·3 (0·6–2·6)	2·2 (1–4·1)
	Nasopharynx cancer	2 (0·7–4)	0·6 (0·2–1·3)	1·3 (0·5–2·7)
	Other pharynx cancer	0·5 (0·2–1)	0·1 (0–0·3)	0·3 (0·1–0·7)
	Stroke	27·8 (11·3–59·4)	18·3 (7·3–37·7)	23·1 (9·4–48·4)
**Sub-Saharan Africa**				
	Esophageal cancer	6·5 (2·7–13·3)	4·3 (1·6–9)	5·4 (2·7–11·7)
	Larynx cancer	2·1 (0·8–4·4)	0·3 (0·1–0·8)	1·2 (0·5–2·6)
	Lip and oral cavity cancer	3·6 (1·6–7·1)	2·4 (1–4·9)	3 (1·3–5·7)
	Nasopharynx cancer	1·4 (0·5–2·9)	0·8 (0·3–1·6)	1·1 (0·4–2·2)
	Other pharynx cancer	0·6 (0·2–1·2)	0·2 (0·1–0·4)	0·4 (0·2–0·9)
	Stroke	24·1 (9–55)	21·2 (7·2–49·4)	22·6 (8·8–49·3)

Appendix Table 2·15: Number and proportion of total DALYs for each cause attributable to chewing tobacco, among ages 15+, in 2023

	Cause	Male			Female			Both		
		Chewing tobacco (thousands)	Total (thousands)	Percent (%)	Chewing tobacco (thousands)	Total (thousands)	Percent (%)	Chewing tobacco (thousands)	Total (thousands)	Percent (%)
**Global**										
	Esophageal cancer	478 (286–793)	10100 (8840–11600)	4·7 (3–7·2)	268 (132–470)	3970 (3120–5040)	6·7 (3·9–10·7)	746 (448–1170)	14000 (12600–15800)	5·3 (3·4–8·1)
	Larynx cancer	341 (179–547)	3050 (2520–3790)	11·2 (6·3–16·7)	42·7 (18·7–73)	508 (392–694)	8·4 (4·3–13·5)	384 (195–609)	3560 (2990–4290)	10·8 (6–16·1)
	Lip and oral cavity cancer	780 (496–1120)	4200 (3460–5120)	18·6 (13·5–24)	339 (199–525)	2250 (1770–2960)	15·1 (9·7–22·3)	1120 (729–1580)	6450 (5570–7620)	17·3 (12·2–23·3)
	Nasopharynx cancer	119 (59·8–201)	1740 (1370–2150)	6·9 (3·8–11)	43·1 (19·4–78·4)	807 (604–1060)	5·3 (2·6–8·6)	163 (80·6–267)	2540 (2110–2990)	6·4 (3·5–10·3)
	Other pharynx cancer	368 (187–590)	2700 (2040–3480)	13·7 (8·1–20·2)	63·1 (31·5–118)	634 (472–915)	10 (5·2–15·2)	431 (220–671)	3330 (2680–4160)	13 (7·7–19·3)
	Stroke	2090 (1120–3600)	84200 (74700–95800)	2·5 (1·3–4·1)	1540 (720–2740)	69800 (61100–82000)	2·2 (1·1–3·7)	3630 (1890–6070)	154000 (139000–169000)	2·4 (1·2–3·8)
**Central Europe, Eastern Europe, and Central Asia**										
	Esophageal cancer	2·02 (0·713–4·28)	402 (376–429)	0·5 (0·2–1·1)	0·326 (0·107–0·759)	105 (96·4–112)	0·3 (0·1–0·7)	2·34 (0·819–5·02)	506 (479–535)	0·5 (0·2–1)
	Larynx cancer	1·93 (0·652–4·33)	305 (285–327)	0·6 (0·2–1·4)	0·131 (0·0406–0·311)	29·1 (26·6–32)	0·5 (0·1–1)	2·06 (0·694–4·66)	334 (313–357)	0·6 (0·2–1·4)
	Lip and oral cavity cancer	3·73 (1·35–8·03)	348 (320–377)	1·1 (0·4–2·2)	0·667 (0·251–1·53)	101 (91·1–112)	0·7 (0·2–1·5)	4·39 (1·6–9·58)	449 (419–477)	1 (0·4–2)
	Nasopharynx cancer	0·436 (0·152–0·999)	47 (41·4–52·6)	0·9 (0·3–2·1)	0·069 (0·0208–0·164)	16·4 (13·8–19·5)	0·4 (0·1–1)	0·505 (0·175–1·16)	63·4 (57·1–70·6)	0·8 (0·3–1·8)
	Other pharynx cancer	1·32 (0·428–2·96)	264 (245–286)	0·5 (0·2–1·1)	0·137 (0·0426–0·311)	38·4 (34·5–43·9)	0·4 (0·1–0·8)	1·45 (0·469–3·27)	302 (284–323)	0·5 (0·2–1·1)
	Stroke	14·1 (4·78–31·4)	6400 (6100–6700)	0·2 (0·1–0·5)	6·86 (2·12–16·5)	6100 (5570–6490)	0·1 (0–0·3)	21 (6·89–48)	12500 (11800–13100)	0·2 (0·1–0·4)
**High-income**										
	Esophageal cancer	21·3 (12·4–36·3)	1390 (1280–1480)	1·5 (0·9–2·6)	1·25 (0·409–2·88)	371 (330–411)	0·3 (0·1–0·8)	22·6 (13–38·9)	1760 (1630–1870)	1·3 (0·7–2·2)

Cause	Male			Female			Both		
	Chewing tobacco (thousands)	Total (thousands)	Percent (%)	Chewing tobacco (thousands)	Total (thousands)	Percent (%)	Chewing tobacco (thousands)	Total (thousands)	Percent (%)
Larynx cancer	6·94 (3·76–12·2)	349 (310–394)	2 (1·1–3·4)	0·33 (0·099–0·812)	63·5 (54·1–74·6)	0·5 (0·2–1·2)	7·27 (3·9–12·8)	413 (372–458)	1·8 (1–3·1)
Lip and oral cavity cancer	16 (10·1–25·1)	490 (449–534)	3·3 (2–5·3)	1·95 (0·695–4·47)	236 (199–271)	0·8 (0·3–1·8)	17·9 (10·9–29·3)	726 (666–793)	2·5 (1·5–4·1)
Nasopharynx cancer	1·81 (0·82–3·46)	99·8 (85·5–117)	1·8 (0·9–3·4)	0·163 (0·0456–0·405)	33·5 (26·8–40·4)	0·5 (0·1–1·2)	1·97 (0·907–3·91)	133 (117–155)	1·5 (0·7–2·8)
Other pharynx cancer	5·88 (3·13–10·1)	397 (362–429)	1·5 (0·8–2·6)	0·344 (0·104–0·791)	85·7 (74·5–97·5)	0·4 (0·1–0·9)	6·23 (3·32–10·8)	483 (444–519)	1·3 (0·7–2·3)
Stroke	48·8 (25·3–82·5)	7210 (6530–7770)	0·7 (0·3–1·1)	11·2 (3·55–26·9)	7040 (5920–7820)	0·2 (0–0·4)	60 (30·8–107)	14200 (12600–15500)	0·4 (0·2–0·7)
**Latin America and Caribbean**									
Esophageal cancer	2·56 (0·899–5·41)	396 (373–416)	0·6 (0·2–1·4)	0·552 (0·185–1·26)	108 (98·5–116)	0·5 (0·2–1·2)	3·11 (1·08–6·67)	504 (477–524)	0·6 (0·2–1·3)
Larynx cancer	2·12 (0·695–4·88)	222 (211–234)	1 (0·3–2·2)	0·247 (0·0784–0·592)	33·9 (30·6–37·5)	0·7 (0·2–1·7)	2·37 (0·775–5·49)	256 (243–270)	0·9 (0·3–2·1)
Lip and oral cavity cancer	2·69 (1·02–5·71)	187 (177–196)	1·4 (0·5–3)	0·894 (0·325–1·99)	80·7 (72·6–87·5)	1·1 (0·4–2·5)	3·59 (1·36–7·73)	267 (254–279)	1·3 (0·5–2·9)
Nasopharynx cancer	0·248 (0·0784–0·564)	30·6 (27·6–34·5)	0·8 (0·2–1·8)	0·0741 (0·0215–0·176)	12·5 (11–14·2)	0·6 (0·2–1·4)	0·322 (0·0999–0·749)	43·1 (39·6–47·1)	0·7 (0·2–1·7)
Other pharynx cancer	0·945 (0·316–2·14)	127 (121–133)	0·7 (0·2–1·7)	0·145 (0·0446–0·331)	24·8 (22–27·6)	0·6 (0·2–1·4)	1·09 (0·36–2·47)	152 (145–158)	0·7 (0·2–1·6)
Stroke	10 (3·2–22)	3470 (3260–3690)	0·3 (0·1–0·6)	6·9 (2·18–16·2)	3180 (2960–3400)	0·2 (0·1–0·5)	16·9 (5·37–38·1)	6650 (6260–7040)	0·3 (0·1–0·6)
**North Africa and Middle East**									
Esophageal cancer	2·4 (1·17–4·32)	136 (104–181)	1·8 (1–3)	0·671 (0·295–1·32)	90·6 (60·1–130)	0·7 (0·4–1·3)	3·07 (1·47–5·59)	226 (182–292)	1·4 (0·7–2·3)
Larynx cancer	3·78 (1·57–7·46)	186 (136–239)	2 (1–3·7)	0·362 (0·125–0·848)	39·2 (25·3–55·1)	0·9 (0·4–1·8)	4·14 (1·74–7·97)	225 (175–279)	1·8 (0·9–3·5)
Lip and oral cavity cancer	2·01 (0·981–4·1)	59·5 (42·8–81·5)	3·4 (1·8–6·1)	0·526 (0·241–1·09)	43 (31–60·6)	1·2 (0·6–2·4)	2·54 (1·24–5·15)	102 (81·5–130)	2·5 (1·3–4·6)
Nasopharynx cancer	1·27 (0·436–2·87)	59·6 (40·1–84·1)	2·1 (0·8–4·6)	0·226 (0·0721–0·542)	28·6 (19·4–43)	0·8 (0·3–1·8)	1·5 (0·523–3·32)	88·2 (65·9–115)	1·7 (0·6–3·7)

Cause	Male			Female			Both		
	Chewing tobacco (thousands)	Total (thousands)	Percent (%)	Chewing tobacco (thousands)	Total (thousands)	Percent (%)	Chewing tobacco (thousands)	Total (thousands)	Percent (%)
Other pharynx cancer	0·44 (0·172–0·939)	24·6 (15·2–35·7)	1·8 (0·8–3·4)	0·0889 (0·0309–0·203)	13·6 (9·07–20·3)	0·7 (0·3–1·4)	0·529 (0·205–1·12)	38·2 (27·6–52·4)	1·4 (0·6–2·7)
Stroke	35 (15–70·6)	4490 (3360–6110)	0·8 (0·4–1·4)	13·1 (5·87–26·5)	4410 (3420–5940)	0·3 (0·1–0·6)	48·1 (22·1–92·6)	8900 (7420–10800)	0·5 (0·3–1)
**South Asia**									
Esophageal cancer	376 (233–604)	1810 (1310–2470)	20·7 (13·5–29·5)	243 (118–431)	1540 (1040–2200)	15·7 (9·3–24)	619 (380–974)	3360 (2620–4290)	18·4 (11·5–26·7)
Larynx cancer	308 (163–497)	1160 (804–1680)	26·5 (16·1–38·4)	38·5 (17·2–63·7)	210 (136–323)	18·4 (10–28·9)	347 (177–547)	1370 (1010–1860)	25·3 (15·1–37·1)
Lip and oral cavity cancer	714 (453–1020)	2060 (1500–2750)	34·6 (25·8–45·2)	315 (187–485)	1310 (962–1840)	23·9 (15·4–34·9)	1030 (676–1450)	3370 (2720–4270)	30·5 (21·9–41·5)
Nasopharynx cancer	91·2 (47·4–152)	369 (229–529)	24·7 (14·3–36·5)	33·6 (14·7–60·9)	220 (141–327)	15·3 (7·7–25)	125 (63–202)	589 (423–781)	21·2 (12·1–31·7)
Other pharynx cancer	353 (178–568)	1520 (956–2190)	23·2 (13·8–34·4)	60·4 (29·8–112)	387 (253–614)	15·6 (8·2–24·9)	413 (212–642)	1910 (1370–2560)	21·7 (12·8–32·2)
Stroke	1650 (894–2740)	16100 (12800–20700)	10·2 (5·6–16)	1250 (582–2150)	15500 (11500–19900)	8·1 (4·1–13·4)	2900 (1480–4730)	31600 (25100–37400)	9·2 (4·9–14·8)
**Southeast Asia, East Asia, and Oceania**									
Esophageal cancer	59·4 (21·7–125)	5240 (4600–5970)	1·1 (0·4–2·4)	11·9 (4·6–24·9)	1240 (1010–1480)	1 (0·4–1·9)	71·3 (26·2–148)	6480 (5760–7300)	1·1 (0·4–2·3)
Larynx cancer	14 (4·71–26·8)	663 (541–845)	2·1 (0·8–4·2)	2·25 (0·875–5·03)	97·5 (67·1–131)	2·3 (1–4·9)	16·2 (5·7–31·7)	760 (629–948)	2·1 (0·8–4·3)
Lip and oral cavity cancer	33·9 (15·2–64·4)	847 (723–1000)	4 (1·8–7·2)	14·8 (6·49–29·4)	279 (226–346)	5·3 (2·4–9·9)	48·7 (21·9–88·5)	1130 (998–1290)	4·3 (2–7·9)
Nasopharynx cancer	20·8 (7·38–42·1)	985 (842–1190)	2·1 (0·8–4·4)	6·61 (2·36–14·2)	342 (250–425)	1·9 (0·7–3·9)	27·4 (10·1–57·3)	1330 (1150–1550)	2·1 (0·8–4·4)
Other pharynx cancer	5·55 (1·92–11·4)	306 (251–374)	1·8 (0·7–3·5)	1·4 (0·478–3·03)	55·5 (39·9–78·7)	2·5 (1–5·4)	6·95 (2·5–14·1)	362 (303–427)	1·9 (0·7–3·8)
Stroke	288 (117–616)	39900 (35500–44500)	0·7 (0·3–1·4)	209 (83·7–430)	26500 (22200–30200)	0·8 (0·3–1·6)	497 (202–1040)	66400 (60000–72600)	0·7 (0·3–1·5)
**Sub-Saharan Africa**									

Cause	Male			Female			Both		
	Chewing tobacco (thousands)	Total (thousands)	Percent (%)	Chewing tobacco (thousands)	Total (thousands)	Percent (%)	Chewing tobacco (thousands)	Total (thousands)	Percent (%)
Esophageal cancer	14·1 (5·75–29)	678 (481–954)	2·1 (1–3·9)	10·1 (3·98–20·8)	517 (342–800)	2 (0·8–3·9)	24·3 (11–45·7)	1190 (939–1580)	2 (0·9–3·8)
Larynx cancer	4·56 (1·77–9·52)	166 (118–236)	2·7 (1·2–5·3)	0·815 (0·277–1·92)	34·9 (23·1–50·5)	2·3 (0·9–4·7)	5·38 (2·2–10·7)	201 (152–269)	2·7 (1·2–5·2)
Lip and oral cavity cancer	8·03 (3·78–15·9)	209 (148–288)	3·8 (1·9–6·9)	5·86 (2·5–12)	197 (139–287)	3 (1·3–5·9)	13·9 (5·98–26·7)	407 (320–511)	3·4 (1·6–6·4)
Nasopharynx cancer	3·61 (1·35–7·57)	147 (84·2–209)	2·5 (1·1–5·1)	2·35 (0·856–4·82)	154 (102–229)	1·5 (0·6–3·2)	5·95 (2·26–12·2)	301 (219–391)	2 (0·8–4·1)
Other pharynx cancer	1·39 (0·499–2·84)	58·5 (37–85·6)	2·4 (1·1–4·4)	0·54 (0·191–1·16)	28·3 (18·5–44·1)	1·9 (0·8–3·8)	1·93 (0·677–3·82)	86·8 (62·7–117)	2·2 (1–4·2)
Stroke	47·8 (17·7–109)	6620 (4890–8790)	0·7 (0·3–1·5)	43·5 (14·7–101)	7040 (5260–9530)	0·6 (0·2–1·3)	91·2 (35·3–198)	13700 (10900–16800)	0·7 (0·2–1·4)

Return to Table of Contents

Appendix Figure 2·6: Cause-specific DALY rates attributable to chewing tobacco use in 2023, among ages 15+, by sex and South Asian nation

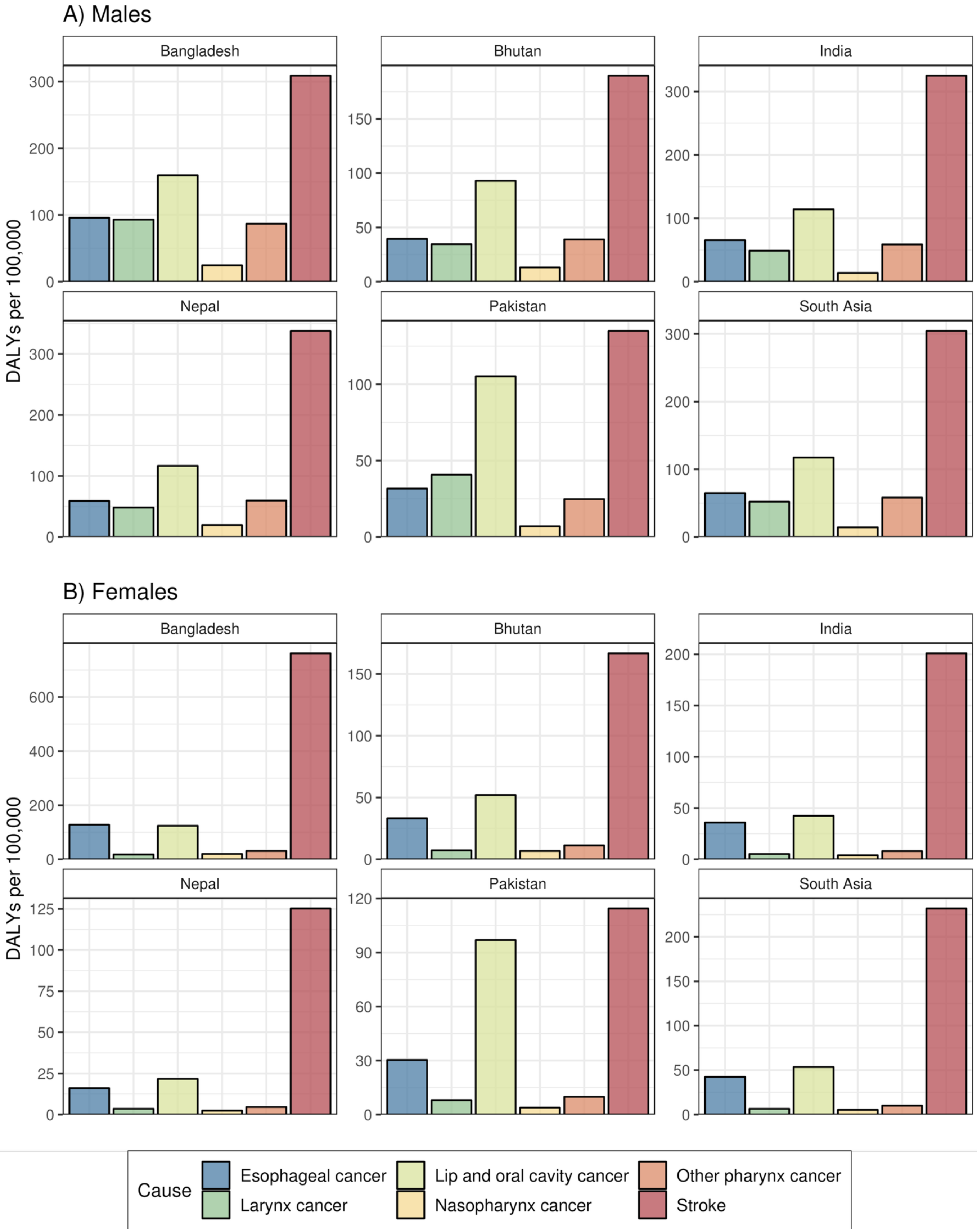

Appendix Figure 2·7: Age-standardized rate of DALYs attributable to chewing tobacco use, age 15+, by sex, 2023

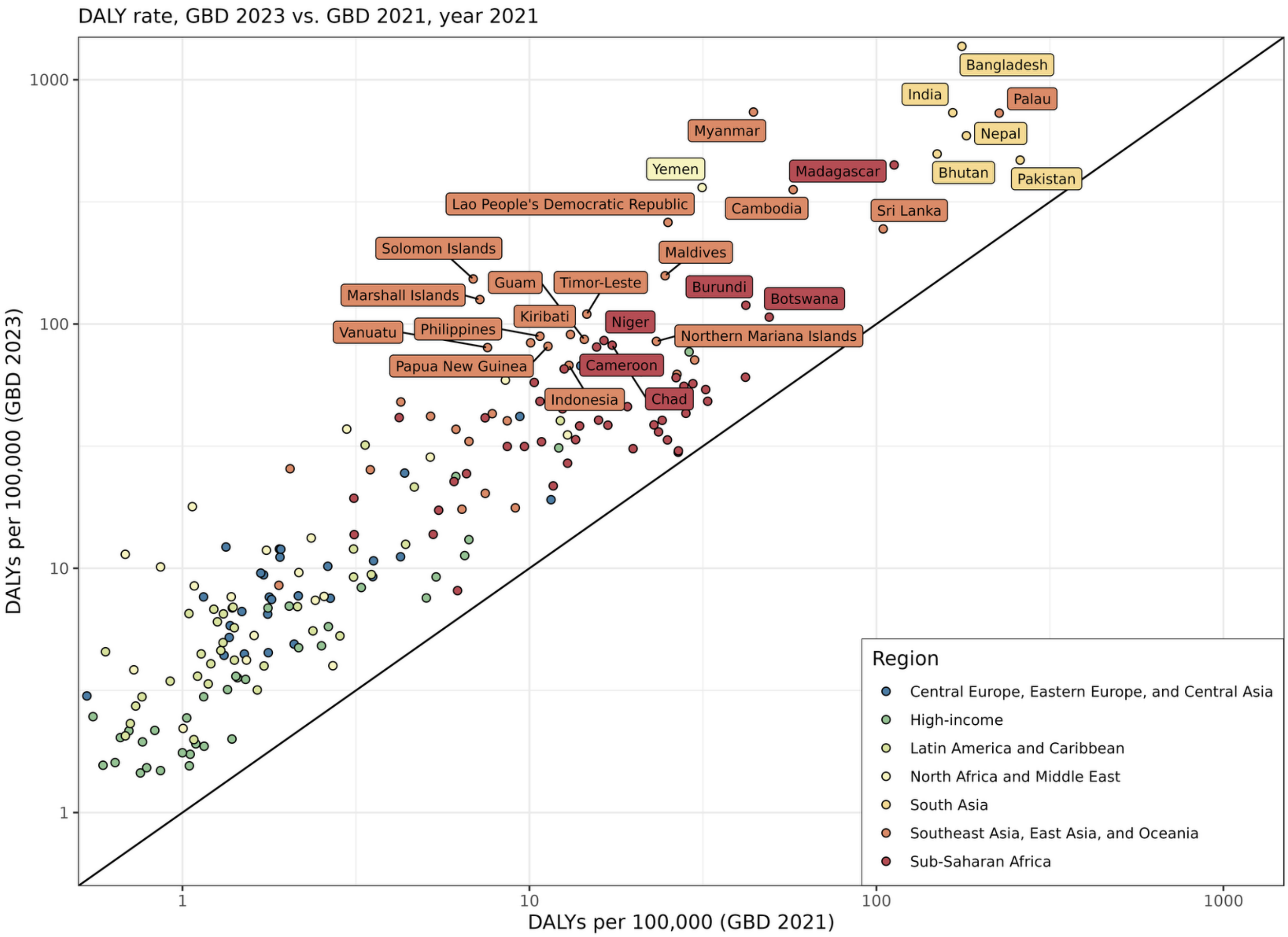

Return to Table of Contents

Appendix Figure 2·8: Ratio of DALYs attributable to chewing tobacco use to DALYs attributable to smoking, age 30+, by sex, 2023

Male

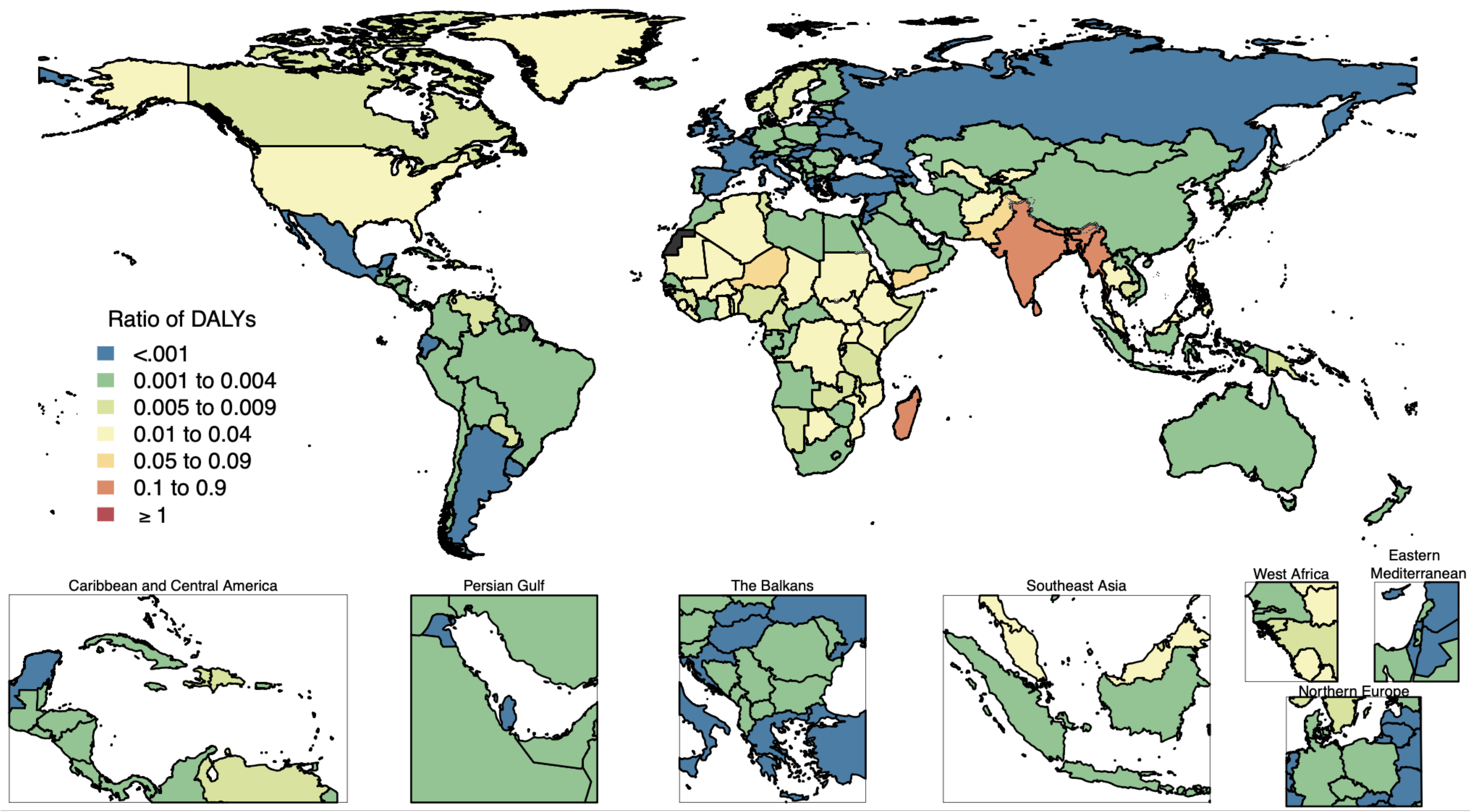

Female

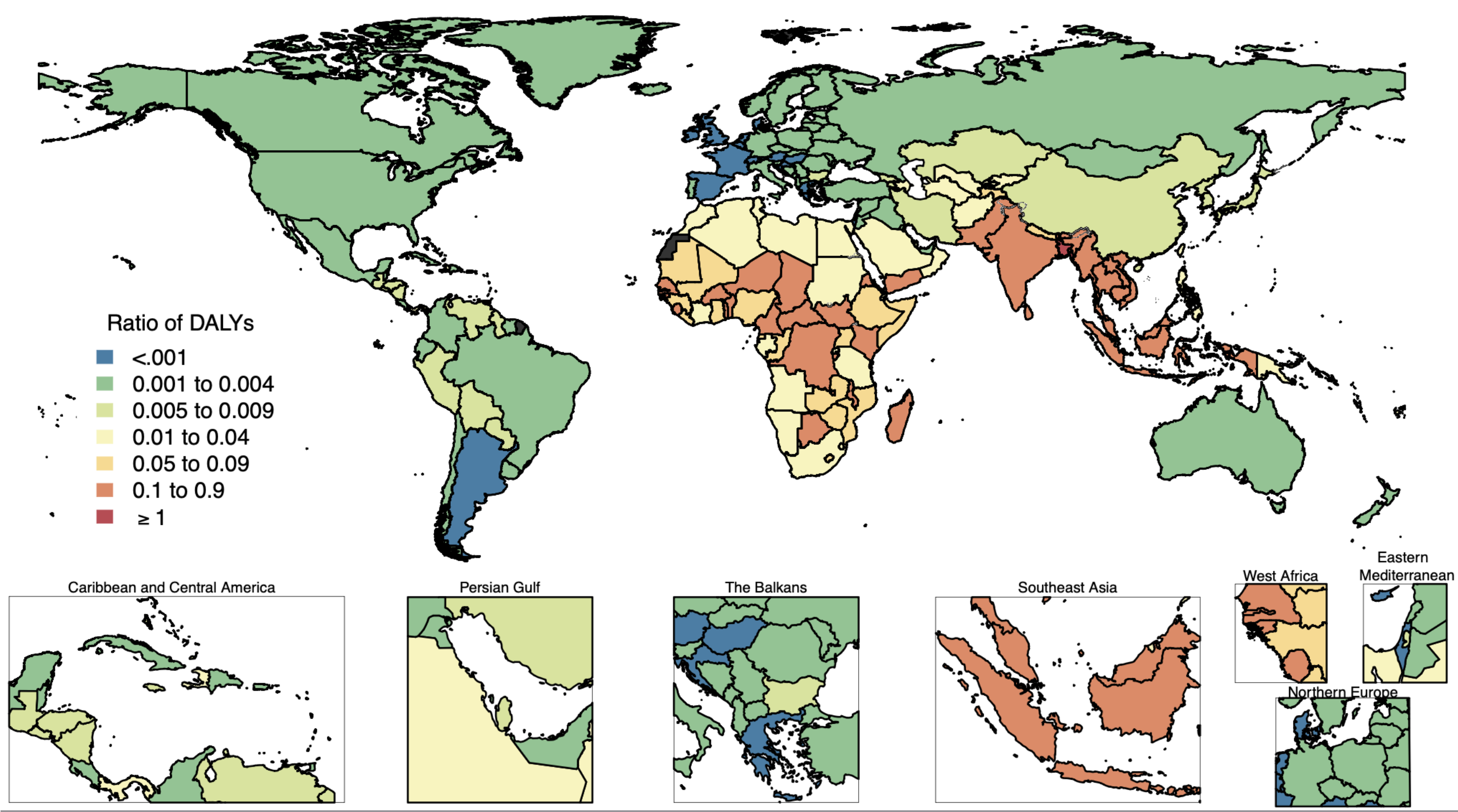

Supplement: Supplementary file 2 [file mmc2.pdf]
