# Supplementary material for: Global, regional, and national burden of exposure to chewing tobacco, 1990–2023: a systematic analysis of the Global Burden of Disease Study 2023
Source: Lancet Glob Health. 2026 Sep 30;14(11):None. doi: 10.1016/j.langlo.2026.104043 (PMC13755197; doi:10.1016/j.langlo.2026.104043)
Supplement: Supplementary file 3 [file mmc3.pdf]

THE LANCET
Global Health

Supplementary appendix 3

This appendix formed part of the original submission and has been peer reviewed.
We post it as supplied by the authors.

Supplement to: Global, regional, and national burden of exposure to chewing tobacco, 1990–2023: a systematic analysis of the Global Burden of Disease Study 2023. *Lancet Glob Health* 2026. https://doi.org/10.1016/j.langlo.2026.104043

Appendix 3: Authorship appendix to "Global, regional, and national burden of exposure to chewing tobacco, 1990-2023: a systematic analysis of the Global Burden of Disease Study 2023"

This appendix provides further authorship detail for "Global, regional, and national burden of exposure to chewing tobacco, 1990-2023: a systematic analysis of the Global Burden of Disease Study 2023"

Table of Contents

Appendix 3: Authorship appendix to "Global, regional, and national burden of exposure to chewing tobacco, 1990-2023: a systematic analysis of the Global Burden of Disease Study 2023" 1

GBD 2023 Chewing Tobacco Collaborators 2

Affiliations 5

Authors' Contributions 23

Managing the overall research enterprise 23

Writing the first draft of the manuscript 23

Primary responsibility for applying analytical methods to produce estimates 23

Primary responsibility for seeking, cataloguing, extracting, or cleaning data; designing or coding figures and tables 23

Providing data or critical feedback on data sources 23

Developing methods or computational machinery 25

Providing critical feedback on methods or results 25

Drafting the work or revising it critically for important intellectual content 28

Managing the estimation or publications process 30

GBD 2023 Chewing Tobacco Collaborators

Gabriela Fernanda Gil*, Jason A Anderson, Xiaochen Dai, Luisa S Flor**, Oliver J McElvaney, Brooks W Morgan, Erin C Mullany, Erin M O'Connell, Bhoomadevi A, Hasan Aalruz, Rashad Abdul-Ghani, Rizwan Suliankatchi Abdulkader, Parisa Abedi, Olugbenga Olusola Abiodun, Olumide Abiodun, Nagah M Abourashed, Dariush Abtahi, Fuad Hamdi A Abuadas, Mohammad H Abuadas, Rana Kamal Abu-Farha, Ahmed Abu-Zaid, Anirudh Balakrishna Acharya, Apurba Acharya, Swetha Acharya, Babatope Oluwadamilare Adebiyi, Miracle Ayomikun Adesina, Juliana Bunmi Adetunji, Usha Adiga, Aanuoluwapo Adeyimika Afolabi, Muhammad Sohail Afzal, Feleke Doyore Agide, Aqeel Ahmad, Khabir Ahmad, Khurshid Ahmad, Sajjad Ahmad, Ayman Ahmed, Md Faisal Ahmed, Naveed Ahmed, Shahzaib Ahmed, Sara Akeel, Akil Akhtar, Tomi Akinyemiju, Omar Al Omari, Mohammad Al Qadire, Yazan Al Thaher, Mohammad Ahmmad Mahmoud Al Zoubi, Timothy Olukunle Aladelusi, Mohammed Sultan Al-Ak'hali, Mohammad Khursheed Alam, Rasmieh Mustafa Al-Amer, Turki M Alanzi, Shams Moh'd Fares Khalaf Al-Anzi, Ahmad M Al-Bashaireh, Mohammed S Aldossary, Raouf Alebshehy, Aqeel M Alenazi, Fahad D Algahtani, Abdelazeem M Algammal, Khalid F Alhabib, Fadwa Naji Alhalaiqa, Amidu Alhassan, Ashraf Alhumaidi, Inayat Ali, Irfan Ali, Kamran Ali, Mohammad Daud Ali, Rafat Ali, Syed Shujait Ali, Syed Mohamed Aljunid, Sameer Abdulmalik Alkubati, Wesam Taher Almagharbeh, Mohammed J Almalki, Sabah Al-Marwani, Margret Beaula Alocious Sukumar, Mahmoud A Alomari, Jaber S Alqahtani, Mohammad R Alqudimat, Ahmad Rajeh Al-Qudimat, Ahmed Yaseen Alqutaibi, Sahel Majed Alrousan, Mohammed Amjed Alsaegh, Najim Z Alshahrani, Ali Salman Al-Shami, Zaid Altaany, Awais Altaf, Hassan Alwafi, Yaser Mohammed Al-Worafi, Hany Aly, Mohammad Sharif Ibrahim Alyahya, Abdulmohsen Hamdan Al-Zalabani, Karem H Alzoubi, Adel S Al-Zubairi, Revati Amin, Tarek Tawfik Amin, Etsay Woldu Anbesu, Syam S Andra, Abhishek Anil, Ah Sruthi Anil Kumar, Prapti Anjana, Kabilan Annadurai, Saeid Anvari, Razique Anwer, Gururaj Arakeri, Aleksandr Y Aravkin, Harshit Arora, Monika Arora, Umesh Raj Aryal, Mohammad Ashfaque, Tahira Ashraf, Muhammad Shahzad Aslam, Yuni Asri, Anil Raj Assariparambil, Omer Atac, Seyyed Shamsadin Athari, Samuel Babajide Atoyebi, Alok Atreya, Avinash Aujayeb, Manal Awad, Kemal Lemnuro Awol, Ahmad Ayed, Syed Babar Jamal Bacha, Ashish D Badiye, Riadh Badraoui, Youngoh Bae, Nasser Bagheri, Khlood K Baghlaf, Abdul Rasheed Bahar, Yasemin Bahar, Razieh Bahreini, Parvathy Balachandran, Raju Balaji, Yatan Pal Singh Balhara, Biswajit Banik, Hiba Jawdat Barqawi, Amadou Barrow, Fadi Basha, Md Abu Bashar, Almaz Nibret Belay, Uzma Iqbal Belgaumi, Abdulrahman Babatunde Bello, Ibrahim Olajide Bello, Kelemu Zelalem Berhanu, Pankaj Bhardwaj, Sonu Bhaskar, Ashok Kumar Bhati, Sudip Bhattacharya, Bijit Biswas, Eyob Ketema Bogale, Archith Boloor, Dejana Braithwaite, Zahid A Butt, Senthilkumar C, Sanjay C J, Zhong Cao, Felix Carvalho, Márcia Carvalho, Sandra Carvalho, Joao Mauricio Castaldelli-Maia, Maria Sofia Cattaruzza, Sandip Chakraborty, Rama Mohan Chandika, Vijay Kumar Chattu, Sirshendu Chaudhuri, Akhilanand Chaurasia, Snehal Prakash Chavhan, Meng Xuan Chen, Saravanan Chinnaiyan, Bryan Chong, Sonali Gajanan Choudhari, Dinh-Toi Chu, Isaac Sunday Chukwu, Sunghyun Chung, Alexandru Corlateanu, Claudia Cosma, Natalia Cruz-Martins, Omid Dadras, Namrata Dagli, Gloria Dalla Costa, Mouni Damineni, Lalit Dandona, Rakhi Dandona, Samuel Demissie Darcho, Yagnik Dave, Marco Del Riccio, Ismail Dergaa, Vinoth Gnana Chellaiyan Devanbu, Syed Masudur Rahman Dewan, Arkadeep Dhali, Amol S Dhane, Bibha Dhungel, Kimia Didehvar, Hoa Thi Do, Pranavi Dohare, Sushil Dohare, Cátia Domingues, Jasmin Dönicke, Ojas Prakashbhai Doshi, Jiang Du, Emeka W Dumbili, Senbagam Duraisamy, Siddhartha Dutta, Kristina Edvardsson, Michael Ekholuenetale, Suhaib El Khatib, Iman El Sayed, Mohamed Ahmed Eladl, Omar Abdelsadek Abdou Elmeligy, Ashraf A El-Metwally, Abdelgawad Salah Eltahawy, Collins Nduka Esomchi, Elochukwu Ezenwankwo, Devaraj Ezhilarasan, Adeniyi Francis Fagbamigbe, Ali Faramarzi, Mohammad Fareed, Carla Sofia E Sá Farinha, Munazza Fatima, Alireza Feizkhah, Feten Fekih-Romdhane, Nuno Ferreira, Anteneh Fikrie, Filippos T Filippidis, Florian Fischer, James L Fisher, Muktar A Gadanya, Márió Gajdács, Balasankar Ganesan, Balasubramanian Ganesh, Shivaprakash Gangachannaiah, Fernando Jr Barroga Garcia, Rupesh K Gautam, Haftay Gebremedhin, Mizan Habtemichael Gebresilassie, Khalid Yaser

Ghailan, Lobna Gharaibeh, Sailaja Ghimire, Sama Ghoba, Ehsan Gholami, Zainab Gholami, Thinh Gia Gia Nguyen, Artyom Urievich Gil, Gamze Gökulu, Pouya Goleij, Divya Gopinath, Michal Grivna, Bin Guan, Zenawi Hagos Gufue, Damitha Asanga Gunawardane, Sasidhar Gunturu, Anish Kumar Gupta, Bhawna Gupta, Himanshu Gupta, Prakash C Gupta, Rajeev Gupta, Sunanda Gupta, Yonas Deressa Guracho, Parham Habibzadeh, Yohannes Gebreegziabher Haile, Ferdous Hakim, Esam Halboub, Sajid Hameed, Nasrin Hanifi, Arief Hargono, Ahmed I Hasaballah, Faizul Hasan, Ikramul Hasan, Shamimul Hasan, Towhid Hasan, Nada Tawfig Hashim, Ahmad Shoeb Hashmi, Md Saquib Hasnain, Nageeb Hassan, Simon I Hay, Behzad Heibati, Mohammad Heidari, Zaw Myo Hein, Mohamed Fawzi Hemida, Brenda Yuliana Herrera-Serna, Yuta Hiraike, Ashley Mark Hopkins, Lubna Hossain, Sahadat Hossain, Mehdi Hosseinzade, Weijun Huang, Javid Hussain, Bing-Fang Hwang, Luigi Francesco Iannone, Segun Emmanuel Ibitoye, Olayinka Stephen Ilesanmi, Irena M Ilic, Milena D Ilic, Mohammad Tarique Imam, Mustapha Immurana, Lalu Muhammad Irham, CV Irshad, M Mofizul Islam, Farhad Islami, Juan S Izquierdo-Condoy, Jaison Jacob, Abhishek Jaiswal, Mihajlo Jakovljevic, Qazi Mohammad Sajid Jamal, Rajiv Janardhanan, Ruwan Duminda Jayasinghe, Achala Upendra Jayatilleke, Gwang Hun Jeong, Bijay Mukesh Jeswani, Zixiang Ji, Shuai Jin, Jobin Jose, Alex Joseph, Nitin Joseph, Charity Ehimwenma Joshua, Hema Shree K, Vaishali K, Billingsley Kaambwa, Zubair Kabir, Vidya Kadashetti, Dler Hussein Kadir, Pradnya Vishal Kakodkar, Ebbie Kalan, Mehnaz Kamal, Mona Kanaan, Jiseung Kang, Kehinde Kazeem Kanmodi, Surya Kant, Neeti Kapoor, Shalini Kapoor, Debasish Kar, Sujita Kumar Kar, Neila Karagezova, Mohmed Isaqali Karobari, Manoj Kumar Kashyap, Chukwudi Keke, Asaad Khalid, Maseer Khan, Moien Ab Khan, Muhammad Arslan Khan, Muhammad Umer Khan, Serab Khan, Sumaiya Khan, Zahid Khan, Zelal Kharaba, Sameer Uttamaro Khasbage, Samira Khoshvaght, Mahmood Khosrowjerdi, P Ratan Khuman, Min Seo Kim, Marta A Kisiel, Shivakumar KM, Prakash Babu Kodali, Oleksii Korzh, Archana Koul, Sindhura Lakshmi Koulmane Laxminarayana, Kewal Krishan, Abhishek Krishna, Deepak Krishnamurthy, Nitya Krishnasamy, Pranav S Kshtriya, Chong-Han Kua, Garry Kuan, Claudia E Kuehni, Omar Kujan, Mukhtar Kulimbet, Ashok Kumar, Dhasarathi Kumar, Diwakar Kumar, G Anil Kumar, Laksh Kumar, Mohan Kumar, Nithin Kumar, Pawan Kumar, Pradeep Kumar, Rakesh Kumar, Tushar Kumar, V Udaya Kumar, Om P Kurmi, Maria Dyah Kurniasari, Krishna Prasad Kurpad, Taeho Kwak, Chandrakant Lahariya, Hanpeng Lai, Timo Lajunen, Agung Dwi Laksono, Yilma Markos Larebo, Savita Lasrado, Munjae Lee, Seung Won Lee, Hui Li, Jiaying Li, Qiang Li, Si Li, Xingang Li, Zhengrui Li, Yanxue Lian, Daniel Lindholm, Bingsheng Liu, Gang Liu, Jue Liu, José Francisco López-Gil, Giancarlo Lucchetti, Abhilash Ludhiadch, Pincheng Luo, Hung N Luu, Miltiadis D Lytras, Monika Machoy-Rakoczy, Ahmed A Madfa, Samatar Abshir Mahamed, Mansour Adam Mahmoud, Kashish Malhotra, Lokesh Manjani, Farheen Mansoor, Miquel Martorell, Yasith Mathangasinghe, Medha Mathur, Neeta Mathur, Khurshid A Mattoo, Enkeleint A Mechili, John Robert Carabeo Medina, Jitendra Kumar Meena, Ravi Mehrotra, Vini Mehta, Satish Melwani, Aamir Raoof Memon, Godfred Antony Menezes, Ritesh G Menezes, Tarig H Merghani, Tomislav Mestrovic, Chamila Dinushi Kukulege Mettananda, Mohamed M M Metwally, Muhammad Agus Mikrajab, Giuseppe Minervini, Erkin M Mirrakhimov, Vinaytosh Mishra, Sanjeev Misra, Tridip Mitra, Farduus Ibraahim Mohamed, Nouh Saad Mohamed, Ibrahim Mohammadzadeh, Zaidan Mohammed, Syam Mohan, Nagaishwarya Moka, Ali H Mokdad, Hirbaye Mokona, Himel Mondal, Rafael Silveira Moreira, Rohith Motappa, Sumaira Mubarik, Amartya Mukhopadhyay, Malaisamy Muniyandi, Christopher J L Murray, Krishna Murti, Vignesh Murugan, David Musoke, Sathish Muthu, Karikalan Nagarajan, Kiran P P Nagdeo, Ganesh R Naik, Gopal Nambi, Vinay Nangia, Shumaila Nargus, Mohammad Zakaria Nassani, Zuhair S Natto, Biswa Prakash Nayak, Kinjal Nayak, Andrew Kamsoko Ndakotsu, Sabina Onyinye Nduaguba, Samata Nepal, Marie Ng, Cuong Tat Nguyen, Duy Cao Nguyen, Huong Lan Thi Nguyen, Trang Nguyen, Ali Nikoobar, Vikram Niranjan, Fred Nugen, Wahyu Pudji Nugraheni, Nazik Nurelhuda, Bogdan Oancea, Ramez M Odat, Fabio Massimo Oddi, Ismail A Odetokun, Olusegun Olatunji Ojedoyin, Osaretin Christabel Okonji, Andrew T Olagunju, Jeby Jose Olickal, Ahmed Omar Bali, Hope Onohuean, Miracle Chinonso Oparah, Michal Ordak, Augustus Osborne, Tope Oyelade, Mahesh P A, Alicia Padron-

Monedero, Raul Felipe Palma-Alvarez, Alok Kumar Panda, Deepshikha Pande Katare, Ashok Pandey, Sanjib Raj Raj Pandey, Ioannis Pantazopoulos, Shahina Pardhan, Tarang Parekh, Romil R Parikh, Arpit Parmar, Pragnesh Parmar, Sameena Parveen, Roberto Passera, Bhargav Umeshchandra Patel, Satyananda Patel, Shivani Patel, Vipin Patidar, Ashlesh Patil, Dimitrios Patoulias, Uttam Paudel, Shrikant Pawar, Saikiran Pendem, Prince Peprah, Gavin Pereira, Olumuyiwa James Peter, Tung Thanh Pham, Edoardo Pirera, Roman V Polibin, Ramesh Poluru, Thantrira Porntaveetus, Amit Porwal, Priyanka Porwal, Pranil Man Singh Pradhan, Elton Junio Sady Prates, Harsh Priya, Bharathi M Purohit, Jagadeesh Puvvula, Ibrahim Qattea, Raghu Anekal Radhakrishnan, Venkatraman Radhakrishnan, Yashpal Singh Raghav, Pracheth Raghuveer, Fakher Rahim, Sajjad Rahimi, Firman Suryadi Rahman, Md Ferdous Rahman, Mohammad Hifz Ur Rahman, Mosiur Rahman, Amita Rai, Sunil Kumar Raina, Jeffrey Pradeep Raj, Mahmoud Mohammed Ramadan, Majed Ramadan, Kadar Ramadhan, Shakthi Kumaran Ramasamy, Pramod W Ramteke, Lokesh Rana, Shailendra Singh Rana, Chhabi Lal Ranabhat, Kumuda Rao, Sowmya J Rao, Devarajan Rathish, Tapash Ranjan Rautray, Salman Rawaf, Abdul Rehman, Mina Rezaei, Nazila Rezaei, Ravi Rohilla, Susanne Röhr, Chanapong Rojanaworarit, Himanshu Sekhar Rout, Shubhanjali Roy, Rohini Ruhil, Chandan S N, Cameron John Sabet, Mamta Sachdeva Dhingra, Basema Ahmad Saddik, Mohammad Reza Saeb, Umar Saeed, Dominic Sagoe, Indranil Saha, Amirhossein Sahebkar, Vaibhav Sahni, Ambika Sahoo, Mirza Rizwan Sajid, Joseph W Sakshaug, Afeez Abolarinwa Salami, Mohamed A Saleh, Janmejaya Samal, Abdallah M Samy, Siddaram Shivaji Sarate, Aswini Saravanan, Yaser Sarikhani, Mohammad Sarmadi, Gargi Sachin Sarode, Sachin C Sarode, Mukesh Kumar Sathya Narayanan, Aanchal Satija, Shakta Mani Satyam, Ganesh Kumar Saya, Christophe Schinckus, Art Schuermans, Siddharthan Selvaraj, Mohammad H Semreen, Subramanian Senthilkumaran, Yashendra Sethi, Seyed Mohammad Seyed Alshohadaei, Ahmed Nabil Shaaban, Arash Shaban-Nejad, Muhammed Shabil, Sweni Shah, Sunder Sham, Muhammad Aaqib Shamim, Alfiya Shamsutdinova, Abhishek Shankar, Mohammed Shannawaz, Bhoopesh Kumar Sharma, Manoj Sharma, Ravi Kumar Sharma, Shreya Sharma, Vishal Sharma, Somia Shehzadi, Mahdi Sheikh, Mahabalesh Shetty, Pavanchand Shetty, Premalatha K Shetty, Hui-Zhong Shi, Arun Shirali, Shirindokht Shirazi, Kawkab Shishani, Aminu Shittu, Ivy Shiue, Ambreen Shoaib, Aman Shrestha, Sunil Shrestha, Dharmsheel Shrivastav, Kerem Shuval, Negussie Boti Sidamo, Abhinav Singh, Amit Singh, Bhim Pratap Singh, Brij Pal Singh, Jasvinder A Singh, Jawahar Singh, Poornima Suryanath Singh, Prashant Kumar Singh, Ragini D Singh, Surjit Singh, Virendra Singh, Gisha Sivan, Gowri Sivaramakrishnan, Valentin Yurievich Skryabin, Farrukh Sobia, Md Salman Sohel, Lencho Kajela Solbana, Solikhah Solikhah, Mansi Soni, Joan B Soriano, Michele Sorrentino, Chandrashekhar T Sreeramareddy, Vipul Srivastava, Paschalis Steiropoulos, Marjia Sultana, Zhong Sun, Shanthosh Priyan Sundaram, Johan Sundström, Jithin Surendran, Chandan Kumar Swain, Celine Tabche, Mohammad Tabish, Iman M Talaat, Avni Tambi, Mircea Tampa, Benjamin Kye Jyn Tan, Ingan Ukur Tarigan, Aigul Yelgondiyevna Tazhiyeva, Gebremaryam Temesgen, Reem Temsah, Masayuki Teramoto, Kavumpurathu Raman Thankappan, Rekha Thapar, Madi Tleshev, Thang Huu Tran, Domenico Trico, Aristidis Tsatsakis, Evangelia Eirini Tsermpini, Abdul Rohim Tualeka, Himayat Ullah, Brigid Unim, Bhaskaran Unnikrishnan, Abdulkadir Usman Sambo, Berna Uzun, Hande Uzunçıbuk, Rakesh V R, Sai Mahesh Vajjala, Zahir Vally, Jef Van Den Eynde, Joe Varghese, Siddhartha Alluri Varma, Sampara Vasishta, Vishnu Priya Veeraraghavan, Balachandar Vellingiri, U Venkatesh, Narayanaswamy Venketasubramanian, Akshaya Kumar Verma, Vignesh Vikram, David Villarreal-Zegarra, Liang Wang, Shu Wang, Yaojie Wang, Fei-Long Wei, Dakshitha Praneeth Wickramasinghe, Nuwan Darshana Wickramasinghe, Faustine Williams, Shulan Wu, Hong Xiao, Wanqing Xie, Zemenu Shiferaw Shiferaw Yadita, Kaiqi Yang, Renjulal Yesodharan, Arzu Yiğit, Vahit Yiğit, Biksegn Asrat Asrat Yirdaw, S Yogeshkumar, Dong Keon Yon, Naohiro Yonemoto, Chuanhua Yu, Monal Yuwanati, Muhammad Sohail Zafar, Emilia Zainal Abidin, Fathiah Zakham, Hamed Zandian, Michael Zastrozhin, Saad Zbiri, Mohammed G M Zeariya, Alemu Birara Zemariam, Eyael M Zeru, Haijun Zhang, Zhiqiang Zhang, Zhi-Jiang Zhang, Lei Zheng, Ming-Hua Zheng, Carl Zhou, Mohamed Ali Zoromba, Ahed H

Zyoud, Emmanuela Gakidou**

*Lead author
**Co-senior authors

Affiliations

Institute for Health Metrics and Evaluation (G F Gil MPH, J A Anderson BS, X Dai PhD, L S Flor PhD, B W Morgan MSPH, E C Mullany MBA, E M O'Connell BA, A Y Aravkin PhD, Prof. L Dandona MD, Prof. R Dandona PhD, S Ghoba MS, Prof. S I Hay DSc, T Mestrovic PhD, Prof. A H Mokdad PhD, Prof. C J L Murray DPhil, M Ng PhD, Prof. E Gakidou PhD), Department of Health Metrics Sciences, School of Medicine (X Dai PhD, L S Flor PhD, Prof. S I Hay DSc, Prof. A H Mokdad PhD, Prof. C J L Murray DPhil, Prof. E Gakidou PhD), Division of Pulmonary, Critical Care and Sleep Medicine (O J McElvaney MD), Department of Applied Mathematics (A Y Aravkin PhD), University of Washington, Seattle, WA, USA; Amity Institute of Public Health and Hospital Administration (Prof. B A PhD), Amity University Noida, Uttar Pradesh, India; Department of Nursing (H Aalruz PhD), Al Zaytoonah University of Jordan, Amman, Jordan; Department of Parasitology (Prof. R Abdul-Ghani PhD), Department of Biochemistry and Molecular Biology (Prof. A S Al-Zubairi PhD), Sana'a University, Sana'a, Yemen; Tropical Disease Research Center (Prof. R Abdul-Ghani PhD), University of Science and Technology (USTY), Sana'a, Yemen; National Institute of Epidemiology (R Abdulkader PhD), ICMR - National Institute for Research in Tuberculosis (K Nagarajan PhD), Indian Council of Medical Research, Chennai, India; Yale School of Medicine (P Abedi MD), Department of Genetics (S Pawar PhD), Yale University, New Haven, CT, USA; Neuroendocrine Unit (P Abedi MD), Nutrition Department (G Dalla Costa MD), T.h. Chan School of Public Health (P C Gupta DSc, P M S Pradhan MD), Harvard University, Boston, MA, USA; Department of Internal Medicine (O O Abiodun FWACP), Federal Medical Centre, Abuja, Nigeria; Department of Community Medicine (Prof. O Abiodun MPH), Babcock University, Ilishan-Remo, Nigeria; Zoology Department (N M Abourashed PhD), Benha University, Benha, Egypt; Department of Anesthesiology (D Abtahi MD, S Seyed Alshohadaei MD), Skull Base Research Center (I Mohammadzadeh MD), Social Determinants of Health Research Center (A Nikoobar MD), Shahid Beheshti University of Medical Sciences, Tehran, Iran; Community Health Nursing Department (F H A Abuadas PhD), Preventive Dentistry Department (Prof. M K Alam PhD), Jouf University, Sakaka, Saudi Arabia; Basic Nursing Department (M H Abuadas PhD), Yarmouk University, Amman, Jordan; Clinical Pharmacy and Therapeutics Department (Prof. R K Abu-Farha PhD), Applied Science Private University, Amman, Jordan; Department of Biochemistry and Molecular Medicine (A Abu-Zaid PhD), College of Pharmacy (R Temsah PharmD), Alfaisal University, Riyadh, Saudi Arabia; College of Graduate Health Sciences (A Abu-Zaid PhD), Center for Biomedical Informatics, Department of Pediatrics (Prof. A Shaban-Nejad PhD), University of Tennessee, Memphis, TN, USA; Department of Restorative Dentistry (A B Acharya PhD), College of Dental Medicine (M A Alsaegh PhD), Research Institute for Medical and Health Sciences (M A Alsaegh PhD), Department of Pharmacy Practice and Pharmacotherapeutics (Prof. K H Alzoubi PhD, Z Kharaba PhD), Dental Medicine (Prof. M Awad PhD), Clinical Sciences Department (H J Barqawi MPhil, Prof. I M Talaat PhD), Department of Basic Medical Sciences (M A Eladl PhD), Department of Clinical Sciences (Prof. M M Ramadan PhD), College of Medicine (Prof. M A Saleh PhD), College of Pharmacy (Prof. M H Semreen PhD), Research Institute of Medical & Health Sciences (Prof. M H Semreen PhD), University of Sharjah, Sharjah, United Arab Emirates; Institute of International Health (A Acharya MD), Institute of Public Health (F Fischer PhD), Charité Medical University Berlin, Berlin, Germany; Department of Oral Pathology and Microbiology (S

Acharya PhD), Jss Academy of Higher Education and Research, Mysuru, India; Department of Pediatrics (B O Adebiyi PhD), University of Calgary, Calgary, AB, Canada; School of Public Health (B O Adebiyi PhD), University of the Western Cape, Bellville, South Africa; Slum and Rural Health Initiative Research Academy (M A Adesina BPT), Slum and Rural Health Initiative, Ibadan, Nigeria; Department of Physiotherapy (M A Adesina BPT), Department of Oral and Maxillofacial Surgery (T O Aladelusi FWACS), Parasitology Unit (A B Bello PhD), Department of Epidemiology and Medical Statistics (A F Fagbamigbe PhD), Department of Health Promotion and Education (S Ibitoye PhD), University of Ibadan, Ibadan, Nigeria; Department of Biochemistry (J B Adetunji PhD), Osun State University, Osogbo, Nigeria; Apollo Institute of Medical Sciences & Research Chittoor (Prof. U Adiga PhD), Apollo Hospital, Chittoor, India; Technical Services Directorate (A A Afolabi MPH), Msi Nigeria Reproductive Choices, Abuja, Nigeria; Department of Life Sciences (M S Afzal PhD), University of Management and Technology, Lahore, Pakistan; Department of Public Health (F D Agide PhD), Wachemo University, Kampala, Uganda; College of Medicine (A Ahmad PhD, M Tabish MPharm, H Ullah FCPS), Shaqra University, Shaqra, Saudi Arabia; King Khaled Eye Specialist Hospital and Research Center, Riyadh, Saudi Arabia (Prof. K Ahmad PhD); Department of Health Informatics (K Ahmad PhD, Prof. Q Jamal PhD), Qassim University, Buraydah, Saudi Arabia; Department of Health and Biological Sciences (S Ahmad PhD), Abasyn University, Peshawar, Pakistan; Department of Natural Sciences (S Ahmad PhD), Lebanese American University, Beirut, Lebanon; Institute of Endemic Diseases (A Ahmed PhD), Department of Oral Rehabilitation (N T Hashim PhD), University of Khartoum, Khartoum, Sudan (Prof. T H Merghani PhD); Pan-African One Health Institute (paohi), Kigali, Rwanda (A Ahmed PhD); Department of Health Sciences and Informatics (M Ahmed BSc), Bangladesh Institute of Innovative Health Research, Dhaka, Bangladesh; Department of Assistance Medical Sciences, Applied College (N Ahmed PhD), Faculty of Nursing (W T Almagharbeh PhD), University of Tabuk, Tabuk, Saudi Arabia (S Ali PhD); Department of Medicine (S Ahmed MBBS), Fatima Memorial Hospital College of Medicine and Dentistry, Lahore, Pakistan; Faculty of Dentistry, Oral Diagnostic Sciences (S Akeel PhD), Pediatric Dentistry Department (K K Baghlaf PhD), Department of Dental Public Health (Prof. Z S Natto DrPH), King Abdulaziz University, Jeddah, Saudi Arabia; Emory University School of Medicine (A Akhtar PhD), Emory University, Atlanta, GA, USA; Department of Population Health Sciences (T Akinyemiju PhD), Duke Global Health Institute (T Akinyemiju PhD), Duke University, Durham, NC, USA; Fundamentals and Administration Department (Prof. O Al Omari PhD), Sultan Qaboos University, Muscat, Oman; Al al-Bayt University, Mafraq, Jordan (Prof. M Al Qadire PhD); Faculty of Pharmacy (Y Al Thaher PhD), Faculty of Nursing (A M Al-Bashaireh PhD), Philadelphia University, Amman, Jordan; School of Pharmacy (Y Al Thaher PhD), Cardiff University, Cardiff, UK; School of Public Health (M A M Al Zoubi PhD), University of Texas, Houston, TX, USA; Department of Oral and Maxillofacial Surgery (T O Aladelusi FWACS, A A Salami BDS), Department of Community Medicine (O S Ilesanmi PhD), University College Hospital, Ibadan, Ibadan, Nigeria; Department of Preventive Dental Sciences (Prof. M Al-Ak'hali PhD, A K Bhati MDS), College of Nursing and Health Sciences (M J Almalki PhD), Clinical Nutrition Department (R M Chandika PhD), Department of Public Health (S Dohare MD, K Y Ghailan PhD, F Sobia PhD, J Varghese PhD), Department of Maxillofacial Surgery and Diagnostic Sciences (E Halboub PhD), Health Research Center (Prof. A Khalid PhD), Epidemiology Program (M Khan MD), Department of Prosthetic Dental Sciences (K A Mattoo MD, A Porwal PhD), Maxillofacial Surgery and Diagnostic Sciences (S Parveen PhD), Department of Mathematics (Y Raghav PhD), Department of Clinical Practice (A Shoaib PhD), Jazan University, Jazan, Saudi Arabia; School of Nursing (R M Al-Amer PhD), Yarmouk University, Irbid, Jordan; School of Nursing and Midwifery (R M Al-Amer PhD), Western Sydney University, Sydney, NSW, Australia; Department of Health Information Management and

Technology (Prof. T M Alanzi PhD), Department of Pathology (Prof. R G Menezes MD), Imam Abdulrahman Bin Faisal University, Dammam, Saudi Arabia; School of Nursing (S M F K Al-Anzi MSc), University of British Columbia, Vancouver, BC, Canada; General Directorate of Research and Studies (M S Aldossary MClinDent), Ministry of Health, Riyadh, Saudi Arabia; Department of Health (R Alebshehy PhD), University of Bath, Bath, UK; Bielefeld University, Bielefeld, Germany (R Alebshehy PhD); Health and Rehabilitation Sciences Department (A M Alenazi PhD), College of Nursing (M Zoromba PhD), Prince Sattam Bin Abdulaziz University, Al-Kharj, Saudi Arabia; Department of Public Health (Prof. F D Algahtani PhD, M G M Zeariya PhD), Department of Medical Surgical Nursing (S A Alkubati PhD), Department of Biology (Prof. R Badraoui PhD), Department of Health Management (R Kumar PhD), College of Dentistry (Prof. A A Madfa PhD), University of Hail, Hail, Saudi Arabia; Department of Bacteriology, Immunology, and Mycology (Prof. A M Algammal PhD), Suez Canal University, Ismailia, Egypt; Department of Cardiac Sciences (Prof. K F Alhabib MD), Oral Medicine and Diagnostic Science (Prof. I Bello PhD), King Saud University, Riyadh, Saudi Arabia; College of Nursing (Prof. F N Alhalaiqa PhD), College of Dental Medicine (Prof. K Ali PhD), Qatar University, Doha, Qatar; Adult Health Department (A Alhassan MS), University of Cape Coast, Cape Coast, Ghana; Faculty of Dentistry (A Alhumaidi DDS), Ibn Al-Nafis University for Medical Sciences, Sana'a, Yemen; Department of Oral and Maxillofacial Surgery (A Alhumaidi DDS), Xi'an Jiaotong University, Xi'an, China; Department of Community Medicine (I Ali PhD), Fatima Jinnah Women University, Rawalpindi, Pakistan; Department of Social and Cultural Anthropology (I Ali PhD), University of Vienna, Austria, Vienna, Austria; Department of Statistics and Operations Research (I Ali PhD), Department of Periodontia and Community Dentistry (A Hashmi MDS), Aligarh Muslim University, Aligarh, India; Department of Pharmacy (M Ali PhD), Mohammed Al-Mana College for Medical Sciences, Dammam, Saudi Arabia; Department of Biosciences (R Ali PhD), Faculty of Dentistry (S Hasan MDS), Centre for Interdisciplinary Research in Basic Sciences (S Khan MSc), Jamia Millia Islamia, New Delhi, India; Center for Biotechnology and Microbiology (S Ali PhD), University of Swat, Swat, Pakistan; Department of Public Health and Community Medicine (Prof. S M Aljunid PhD, Prof. C T Sreeramareddy MD), International Medical University, Kuala Lumpur, Malaysia; International Centre for Casemix and Clinical Coding (Prof. S M Aljunid PhD), National University of Malaysia, Bandar Tun Razak, Malaysia; Independent Consultant, Amman, Jordan (S Al-Marwani MSc); SRM School of Public Health, Chennai, India (M Alocious Sukumar PhD); Department of Physical Therapy and Rehabilitation Sciences (Prof. M A Alomari PhD), Department of Rehabilitation Sciences and Physical Therapy (Prof. M A Alomari PhD), Faculty of Medicine (Prof. M S I Alyahya PhD, R M Odat MD), Jordan University of Science and Technology, Irbid, Jordan; Department of Respiratory Care (J S Alqahtani PhD), Prince Sultan Military College of Health Sciences, Dammam, Saudi Arabia; American University of the Middle East, Egaila, Kuwait (M R Alqudimat PhD); Surgical Research Section (A R Al-Qudimat MPH), Hamad Medical Corporation, Doha, Qatar; Department of Prosthodontics and Implant Dentistry (Prof. A Alqutaibi PhD), College of Medicine (Prof. A H Al-Zalabani PhD), Taibah University, Madinah, Saudi Arabia; Department of Prosthodontics and Implant Dentistry (Prof. A Alqutaibi PhD), Ibb University, Ibb, Yemen; Macro-Fiscal Policy Department (S M Alrousan PhD), Ministry of Finance, Dubai, United Arab Emirates; Department of Family and Community Medicine (N Z Alshahrani MD), University of Jeddah, Jeddah, Saudi Arabia; Department of Pharmacology (A S Al-Shami MS), Sana'a University, Sanaa, Yemen; Department of Basic Medical Sciences (A S Al-Shami MS), Universiti Malaysia Sarawak, Kota Samarahan, Malaysia; Department of Basic Sciences (Z Altaany PhD), Faculty of Medicine, Irbid, Jordan; Health Science Division (Z Altaany PhD), Higher Collage of Technology, Abu Dhabi, United Arab Emirates; Institute of Molecular Biology and Biotechnology (imbb) (A Altaf PhD, M Khan PhD), University Institute of Public Health (S

Nargus PhD), University Institute of Medical Lab Technology (S Shehzadi PhD), The University of Lahore, Lahore, Pakistan; Faculty of Health Sciences (A Altaf PhD), Equator University of Science and Technology, Uganda, Masaka, Uganda; Department of Clinical Pharmacology and Toxicology (H Alwafi PhD), Umm Al-Qura University, Makkah, Saudi Arabia; Fakeeh College for Medical Sciences (Prof. Y M Al-Worafi PhD), Fakeeh College for Medical Sciences, Jeddah, Saudi Arabia; College of Medical Sciences (Prof. Y M Al-Worafi PhD), Azal University for Human Development, Sana'a, Yemen; Department of Pediatrics (Prof. H Aly MD), Cleveland Clinic, Cleveland, OH, USA; Laboratory Medicine Department (Prof. A S Al-Zubairi PhD), Al-Baha University, Al-Aqiq, Saudi Arabia; Department of Physiotherapy (R Amin PhD, Prof. V K PhD), Department of Pharmacology (S Gangachannaiah MD), Centre for Toxicovigilance & Drug Safety (A K Gupta PharmD), Manipal College of Dental Sciences (Prof. R A Radhakrishnan PhD), Division of Clinical Pharmacology (J P Raj DM), Manipal College of Dental Sciences, Mangalore (Prof. P K Shetty MDS), Department of General Medicine (A Shirali MD), Kasturba Medical College Mangalore (Prof. B Unnikrishnan MD), Manipal Academy of Higher Education, Manipal, India; Kasr Alainy Faculty of Medicine (Prof. T T Amin MD), Cairo University, Cairo, Egypt; Bio-Medical Research Department (Prof. T T Amin MD), Armed Forces College of Medicine, Cairo, Egypt; Department of Public Health (E W Anbesu MPH), Samara University, Samara, Ethiopia; Department of Environmental Medicine and Public Health (S S Andra PhD), Icahn School of Medicine at Mount Sinai, New York City, NY, USA; Department of Pharmacology (A Anil MD), College of Nursing (J Jacob MSc), Department of Psychiatry (A Parmar DM), All India Institute of Medical Sciences, Bhubaneswar, India; Division of Medical Research (A Anil Kumar PhD, T Mitra MSc), Sri Ramaswamy Memorial Institute of Science and Technology, Chengalpattu, India; Ashok & Rita Patel Institute of Physiotherapy (P Anjana MPT), Charotar University of Science and Technology, Petlad, India; Department of Public Health (K Annadurai PhD), The Apollo University, Chittoor, India; Regenerative Medicine, Organ Procurement and Transplantation Multi-disciplinary Center (S Anvari MD), Department of Social Medicine and Epidemiology (A Feizkhah MD), Guilan University of Medical Sciences, Rasht, Iran; College of Medicine (R Anwer PhD), Imam Mohammad Ibn Saud Islamic University, Riyadh, Saudi Arabia; Department of Head and Neck Oncology (Prof. G Arakeri PhD), HCG Cancer Hospital, Bengaluru, India; Department Of Health Metrics Sciences, School Of Medicine (A Y Aravkin PhD, Prof. R Dandona PhD), University Of Washington, Seattle, WA, USA; Department of Neurosurgery (H Arora MBBS), University of Kentucky, Lexington, KY, USA; Health Promotion Division (M Arora PhD), Public Health Foundation of India, Delhi, India; Research Department (M Arora PhD), Health Related Information Dissemination Amongst Youth, New Delhi, India; Department of Research (U R Aryal PhD), Research Department (S Ghimire MPH, A Pandey MPH, U Paudel PhD), Nepal Health Research Council, Kathmandu, Nepal; Department of Biosciences (M Ashfaque PhD), Integral University, Lucknow, India; Department of Biostatistics (T Ashraf PhD), Sakarya University, Sakarya, Türkiye; School of Traditional Chinese Medicine (M Aslam PhD), Xiamen University Malaysia, Sepang, Malaysia; Nursing Department (Y Asri PhD), Faculty of Health Science (Y Asri PhD), Institute of Technology and Health Science Rs Dr Soepraoen, Malang, Indonesia; Department of Medical-Surgical Nursing (A Assariparambil PhD), Kasturba Medical College, Manipal (S Koulmane Laxminarayana MD), Department of Medical Imaging Technology (S Pendem PhD), Manipal College of Nursing (R Yesodharan MPhil), Manipal Academy of Higher Education, Udupi, India; Institute of Population and Social Research (O Atac PhD), Marmara University, Istanbul, Türkiye; Department of Public Health (O Atac PhD), Istanbul Medipol University, Istanbul, Türkiye; Department of Immunology (S Athari PhD), Department of Critical Care and Emergency Nursing (N Hanifi PhD), Zanjan University of Medical Sciences, Zanjan, Iran; School of Statistics and Actuarial Science (S B Atoyebi MSc), Wits Advanced Drug Delivery Platform Research

Unit (Prof. P Kumar PhD), Wits Infectious Diseases and Oncology Research Institute (Prof. P Kumar PhD), Pharmacy and Pharamacology (H Onohuean PhD), University of the Witwatersrand, Johannesburg, South Africa; Department of Forensic Medicine (A Atreya MD), Department of Community Medicine (S Nepal MD), Lumbini Medical College, Palpa, Nepal; Northumbria HealthCare Nhs Foundation Trust, Newcastle Upon Tyne, UK (A Aujayeb MBBS); Internal Medicine Department (K L Awol MD), Saint Paul's Hospital Millennium Medical College, Addis Ababa, Ethiopia; Bloomberg School of Public Health (K L Awol MD), School of Medicine (B M Jeswani MBBS), Health Policy and Management Department (S Wu MS), Department of Psychiatry (E M Zeru MPH), Department of International Health (H Zhang PhD), Johns Hopkins University, Baltimore, MD, USA; Faculty of Nursing (A Ayed PhD), Arab American University Palestine, Jenin, Palestine; Department of Biological Sciences (S B J Bacha PhD), National University of Medical Sciences (NUMS), Rawalpindi, Pakistan; Department of Forensic Science (A D Badiye PhD, N Kapoor PhD), Government Institute of Forensic Science Nagpur, Nagpur, India; Rashtrasant Tukadoji Maharaj Nagpur University, Nagpur, India (A D Badiye PhD); Faculty of Medicine of Tunis (Prof. R Badraoui PhD), University Tunis el Manar, Tunis, Tunisia; Department of Neurological Surgery (Y Bae MD), University of Ulsan, Seoul, South Korea; Health Research Institute (Prof. N Bagheri PhD), University of Canberra, Canberra, NSW, Australia; Department of Medicine (A Bahar MD, Y Bahar MD), Wayne State University, Detroit, MI, USA; College of Optometry (R Bahreini MS), Pacific University, Forest Grove, OR, USA; Amrita School of Dentistry (P Balachandran MDS), Amrita Vishwa Vidyapeetham, Kochi, India (Prof. K R Thankappan MD); Saveetha Medical College and Hospital (R Balaji PhD, Prof. M Karobari PhD), Department of Pharmacology (Prof. D Ezhilarasan PhD), Department of Oral Medicine and Periodontology (Prof. R D Jayasinghe MS), Department of Pathology (H K PhD), Department of Oral Biology (N Krishnasamy PhD), Saveetha Dental College and Hospitals (G Minervini PhD, M Yuwanati MDS), Department of Biochemistry (S Mohan PhD), Center for Global Health Research (Prof. A Sahebkar PhD), Centre of Molecular Medicine and Diagnostics (Prof. V Veeraraghavan PhD), Saveetha University, Chennai, India; National Drug Dependence Treatment Centre (Prof. Y Balhara MD), Centre for Community Medicine (A Jaiswal MD), Department of Preventive Oncology (J K Meena MD), Centre for Dental Education and Research (H Priya MDS, B M Purohit MDS), Department of Radiation Oncology (V Sahni MDS, A Shankar MD), Department of Laboratory Medicine (A Singh PhD), All India Institute of Medical Sciences, New Delhi, India; Institute of Health and Wellbeing (B Banik PhD), Federation University Australia, Melbourne, VIC, Australia; Manna Institute (B Banik PhD), University of New England, Armidale, NSW, Australia; Department of Public and Environmental Health (A Barrow MPH), University of the Gambia, Banjul, The Gambia; Department of Epidemiology (A Barrow MPH, D Braithwaite PhD), University of Florida, Gainesville, FL, USA; Department of Public Health (F Basha MD), Department of Chemistry (Prof. A H Zyoud PhD), An-Najah National University, Nablus, Palestine; Department of Community and Family Medicine (M Bashar MD), Department of Community Medicine and Family Medicine (U Venkatesh MD), All India Institute of Medical Sciences, Gorakhpur, India; College of Medicine and Health Sciences (A N Belay MSc, Y Guracho PhD, Z S Yadita MPH), Department of Health Promotion and Behavioural Science (E K Bogale MPH), Bahir Dar University, Bahir Dar, Ethiopia; Department of Oral Pathology and Microbiology (U I Belgaumi MD), Department of Oral and Maxillofacial Pathology and Microbiology (V Kadashetti MDS), Department of Public Health Dentistry (Prof. S KM PhD), Department of Periodontology (S A Varma MDS), Krishna Vishwa Vidyapeeth (Deemed to Be University), Karad, India; Department of Pathology (Prof. I Bello PhD), Department of Psychology (Prof. T Lajunen PhD), University of Helsinki, Helsinki, Finland; Education (Prof. K Berhanu PhD), University of Johannesburg, Johannesburg, South Africa; Institute of Behavioral Sciences (Prof. K

Berhanu PhD), Debre Markos University, Debre Markos, Ethiopia; National Institute for Implementation Research On Non Communicable Diseases (Prof. P Bhardwaj MD), Indian Council of Medical Research, Jodhpur, India; Global Health Neurology Lab (S Bhaskar MD), Nsw Brain Clot Bank, Sydney, NSW, Australia; Division of Cerebrovascular Medicine and Neurology (S Bhaskar MD), National Cerebral and Cardiovascular Center, Suita, Japan; Department of Community and Family Medicine (S Bhattacharya MD, B Biswas MD), Department of Physiology (H Mondal MD, S Sharma MA), College of Nursing (V Patidar MSc), All India Institute of Medical Sciences, Deoghar, India; Department of Internal Medicine (A Boloor MD), Department of Community Medicine (N Joseph MD, N Kumar MD, R Motappa MD, J Surendran MD, R Thapar MD), Department of Radiation Oncology (A Krishna MD), Kasturba Medical College (G A Menezes PhD), Manipal Academy of Higher Education, Mangalore, India; Cancer Population Sciences Program (D Braithwaite PhD), University of Florida Health Cancer Center, Gainesville, FL, USA; School of Public Health Sciences (Z A Butt PhD), University of Waterloo, Waterloo, ON, Canada; Al Shifa School of Public Health (Z A Butt PhD), Al Shifa Trust Eye Hospital, Rawalpindi, Pakistan; The Apollo University (S C MOT), Apollo Hospital, Chittor, India; Jss Dental College & Hospital (S C J MDS), Department of Respiratory Medicine (Prof. M P A DNB), Jagadguru Sri Shivarathreeswara Academy of Health Education and Research, Mysore, India; Heidelberg Institute of Global Health (Z Cao PhD), Heidelberg University, Heidelberg, Germany; Research Unit On Applied Molecular Biosciences (ucibio) (Prof. F Carvalho PhD), Associated Laboratory for Green Chemistry (laqv) (M Carvalho PhD), Institute for Research and Innovation in Health (i3S) (Prof. N Cruz-Martins PhD), University of Porto, Porto, Portugal; Faculty of Health Sciences (M Carvalho PhD), University Fernando Pessoa, Porto, Portugal; Department of Basic Psychology (Prof. S Carvalho PhD), Life and Health Sciences Research Institute (icvs) (Prof. N Cruz-Martins PhD), University of Minho, Braga, Portugal; Department of Psychiatry (Prof. J Castaldelli-Maia PhD), University of São Paulo, São Paulo, Brazil; Department of Public Health and Infectious Diseases (M S Cattaruzza PhD), La Sapienza University, Rome, Italy; State Disease Investigation Laboratory (S Chakraborty MVSc), Animal Resources Development Department, Agartala, India; Department of Public Health and Health Sciences (Prof. V Chattu PhD), Tennessee State University (tsu), Nashville, TN, USA; Department of Community Medicine (Prof. V Chattu PhD), Datta Meghe Institute of Medical Sciences, Sawangi, India; Department of Public Health (S Chaudhuri MD), Indian Institute of Public Health, Hyderabad, India; Department of Oral Medicine and Radiology (Prof. A Chaurasia MD), Department of Respiratory Medicine (Prof. S Kant MD), Department of Psychiatry (S K Kar MD), King George's Medical University, Lucknow, India; National Institute of Translational Virology and Aids Research (S P Chavhan MD), Indian Council of Medical Research, Pune, India; School of Dentistry (M Chen DDS), University of Michigan, Ann Arbor, MI, USA; SRM School of Public Health (S Chinnaiyan PhD), SRM Centre for Clinical Trials and Research (cctr) (D Kumar PhD), Sri Ramaswamy Memorial Institute of Science and Technology, Chennai, India; Department of Medicine (B Chong MBBS), Nus-ihme Global Burden of Disease Research Centre, Yong Loo Lin School of Medicine (M Ng PhD), Yong Loo Lin School of Medicine (B Tan MBBS, Prof. N Venketasubramanian MSc), Department of Dentistry (Prof. L Zheng MD), National University of Singapore, Singapore, Singapore; Department of Community Medicine (Prof. S G Choudhari MD), Jawaharlal Nehru Medical College, Wardha, India; The Interdisciplinary Research Group On Biomedicine and Health (D Chu PhD), Faculty of Applied Sciences (D Chu PhD), Vnu International School (vnuis), Hanoi, Viet Nam; Department of Paediatric Surgery (I S Chukwu BMedSc), Federal Medical Centre, Umuahia, Nigeria; Department of Health Behavior (S Chung MPH), Texas A&Mm University, College Station, TX, USA; Department of Respiratory Medicine and Allergology (Prof. A Corlateanu PhD), Nicolae Testemitanu State University of Medicine and Pharmacy, Chisinau, Moldova;

Department of Health Sciences (C Cosma MD, M Del Riccio MD), University of Florence, Florence, Italy; Independent Consultant, Darwin, SA, Australia (O Dadras PhD); Karnavati Scientific Research Centre (N Dagli MA), Karnavati Scientific Research Centre, Gandhinagar, India; Department of Dentistry (M Damineni DDS), Four States Dental Care, Joplin, MO, USA; Public Health Foundation of India, Gurugram, India (Prof. L Dandona MD, Prof. R Dandona PhD, G Kumar PhD); Department of Public Health (S D Darcho MPH), Haramaya University, Harar, Ethiopia; Faculty of Physiotherapy (Y Dave PhD), Marwadi University, Rajkot, India; Infectieziekten in de Eerste Lijn Programme (M Del Riccio MD), Netherlands Institute for Health Services Research, Utrecht, Netherlands; Department of Biological Sciences (I Dergaa PhD), University of Manouba, Manouba, Tunisia; Department of Social Sciences (I Dergaa PhD), University of Jendouba, El Kef, Tunisia; Chettinad Hospital & Research Institute (Prof. V Devanbu MD), Chettinad Academy of Research and Education, Chennai, India; Department of Pharmacy (S Dewan PhD), United International University, Dhaka, Bangladesh; Pharmacology Division (S Dewan PhD), Center for Life Sciences Research Bangladesh, Dhaka, Bangladesh; Sheffield Teaching Hospitals Nhs Foundation Trust, Sheffield, UK (A Dhali MBBS); Research and Development Cell (A S Dhane MBA), Department of Oral Pathology and Microbiology (Prof. G S Sarode PhD, Prof. S C Sarode PhD), Dr. D. Y. Patil Vidyapeeth, Pune (Deemed to Be University), Pune, India; Population Interventions Unit (B Dhungel DrPH), University of Melbourne, Melbourne, VIC, Australia; Department of Anesthesiology (K Didehvar MD), Rutgers University, Newark, NJ, USA; Institute of Health Economics and Technology (H T Do PhD), Institute of Health Economics and Technology, Hanoi, Viet Nam; Amity Institute of Psychology and Allied Sciences (P Dohare BA), Amity Institute of Public Health and Hospital Administration (A Koul MHA, R Ruhil PhD, M Shannawaz PhD, P S Singh PhD), Amity Institute of Forensic Sciences (B P Nayak PhD), Amity University, Noida, India; Laboratory of Drug Development and Technologies, Faculty of Pharmacy (C Domingues MSc), University of Coimbra, Coimbra, Portugal; Institute of Medical Biostatistics, Epidemiology, and Informatics (J Dönicke MSc), Johannes Gutenberg University Mainz, Mainz, Germany; Independent Consultant, Bridgewater, NJ, USA (O P Doshi MS); Department of Pathology (Prof. J Du PhD), China Medical University, Shenyang, China; School of Sociology (E W Dumbili PhD), University College Dublin, Dublin, Ireland; Faculty of Science and Humanities (S Duraisamy PhD), SRM Institute of Science and Technology, Kattankulathur, India; Department of Pharmacology (S Dutta MD), Department of Biochemistry (R D Singh PhD), All India Institute of Medical Sciences, Rajkot, India; School of Nursing and Midwifery (Prof. K Edvardsson PhD), La Trobe University, Bundoora, VIC, Australia; Faculty of Science and Health (M Ekholuenetale PhD), University of Portsmouth, Hampshire, UK; Department of Internal Medicine (S El Khatib MD), University of Colorado Denver, Pueblo, CO, USA; Biomedical Informatics and Medical Statistics Department (Prof. I El Sayed PhD), Pediatric Dentistry and Dental Public Health Department (Prof. O A A Elmeligy PhD), Faculty of Medicine (M Hemida MD), Department of Pathology (Prof. I M Talaat PhD), Alexandria University, Alexandria, Egypt; Department of Anatomy and Embryology (M A Eladl PhD), Department of Cardiology (Prof. M M Ramadan PhD), Faculty of Pharmacy (Prof. M A Saleh PhD), Faculty of Nursing (M Zoromba PhD), Mansoura University, Mansoura, Egypt; College of Public Health and Health Informatics (Prof. A A El-Metwally PhD), King Saud Bin Abdulaziz University for Health Sciences, Riyadh, Saudi Arabia; King Abdullah International Medical Research Center, Riyadh, Saudi Arabia (Prof. A A El-Metwally PhD); Faculty of Veterinary Medicine (Prof. A S Eltahawy PhD), Damanhour University, Damanhur, Egypt; Department of Anatomy (C N Esomchi MSc), State University of Medical and Applied Sciences, Igbo Eno, Nigeria; Department of Anatomy (C N Esomchi MSc), Alex Ekwueme Federal University Ndufu Alike Ikwo, Ikwo, Nigeria; Drexel Dornsife School of Public Health (E Ezenwankwo MPH), Drexel University, Philadelphia, PA, USA; Research Centre for

Healthcare and Community (A F Fagbamigbe PhD), Coventry University, Coventry, UK; Student Research Committee (A Faramarzi MD), Department of Otolaryngology (A Faramarzi MD), Health Policy Research Center (Y Sarikhani PhD), Shiraz University of Medical Sciences, Shiraz, Iran; College of Medicine (M Fareed PhD), AlMaarefa University, Riyadh, Saudi Arabia; Environmental Statistics Unit (C S E S Farinha PhD), National Institute of Statistics, Lisbon, Portugal; Ecological Economics and Environmental Management Department (C S E S Farinha PhD), Nova University of Lisbon, Lisbon, Portugal; Department of Geography and Geoinformatics (M Fatima PhD), The Islamia University of Bahawalpur, Bahawalpur, Pakistan; Department of Psychiatry Ibn Omrane (Prof. F Fekih-Romdhane MD), University Tunis el Manar, Manouba, Tunisia; Department of Social Sciences (Prof. N Ferreira PhD), University of Nicosia, Nicosia, Cyprus; Institute for Health Metrics and Evaluation (A Fikrie MPH), Department of Psychiatry (H Mokona MSc), Bule Hora University, Bule Hora, Ethiopia; School of Public Health (F Filippidis PhD), Department of Primary Care and Public Health (Prof. S Rawaf MD, C Tabche MSc), Imperial College London, London, UK; James Cancer Hospital at the Ohio State University (J L Fisher PhD), Center for Tobacco Research (C Keke PhD), Ohio State University, Columbus, OH, USA; Department of Community Medicine (Prof. M A Gadanya MD), Bayero University Kano, Kano, Nigeria; Department of Community Medicine (Prof. M A Gadanya MD), Aminu Kano Teaching Hospital, Kano, Nigeria; Department of Public Health (M Gajdács PhD), University of Szeged, Szeged, Hungary; School of American Education (B Ganesan PhD), Institute of Health & Management, Melbourne, VIC, Australia; Swinburne University of Technology (B Ganesan PhD), School of Engineering, Melbourne, VIC, Australia; Laboratory Sciences Division (B Ganesh PhD), National Institute of Epidemiology, Chennai, India; Department of Health Policy and Administration (Prof. F B Garcia PhD), National Institutes of Health (J C Medina MD), Department of Clinical Epidemiology (J C Medina MD), University of the Philippines Manila, Manila, Philippines; Amity Institute of Pharmacy (Prof. R K Gautam PhD), Amity University Uttar Pradesh, Noida, India; School of Public Health (H Gebremedhin MPH), Department of Public Health (Z Gufue MPH), Adigrat University, Adigrat, Ethiopia; College of Health Sciences (M H Gebresilassie MSc), Department of Psychiatry (H Mokona MSc), Addis Ababa University, Addis Ababa, Ethiopia; Faculty of Pharmacy (L Gharaibeh PhD), Amman Arab University, Amman, Jordan; Department of Electrical and Computer Engineering (E Gholami PhD), University of California Davis, Davis, CA, USA; Department of Physics (Z Gholami PhD), University of Zanjan, Zanjan, Iran; International University of Health and Welfare (T G Gia Nguyen MD), International University of Health and Welfare, Narita, Japan; Country Office (A U Gil PhD), World Health Organization (WHO), Astana, Kazakhstan; Department of Pediatrics (G Gökulu PhD), Mersin City Training and Research Hospital, Mersin, Türkiye; Department of Genetics (P Goleij MSc), Sana Institute of Higher Education, Sari, Iran; Universal Scientific Education and Research Network (usern) (P Goleij MSc), Kermanshah University of Medical Sciences, Kermanshah, Iran; College of Dentistry (D Gopinath PhD), College of Medicine (Z Hein PhD), Department of Pathological Sciences (A Rehman PhD), Department of Clinical Sciences (Prof. M S Zafar PhD), Ajman University, Ajman, United Arab Emirates (Prof. N Hassan PhD); Institute of Public Health (Prof. M Grivna PhD), Family Medicine Department (M A Khan MSc), United Arab Emirates University, Al Ain, United Arab Emirates; Department of Public Health and Preventive Medicine (Prof. M Grivna PhD), Charles University, Prague, Czech Republic; Tianjin Medical University General Hospital (B Guan MD), Tianjin Medical University, Tianjin, China; Cheeloo College of Medicine (B Guan MD), Shandong University, Jinan, China; Department of Epidemiology and Biostatistics (Z Gufue MPH), University of South Carolina, Columbia, SC, USA; Department of Community Medicine (Prof. D A Gunawardane MD), University of Peradeniya, Kandy, Sri Lanka; Department of Psychiatry (S Gunturu MD), Bronxcare Health System, Bronx, NY, USA; Department of Psychiatry (S Gunturu MD),

Icahn School of Medicine at Mount Sinai, New York, NY, USA; Department of Nephrology (A K Gupta PharmD), Max Super Speciality Hospital, New Delhi, India; Department of Public Health (B Gupta PhD), Torrens University Australia, Melbourne, VIC, Australia; College of Medicine and Public Health (H Gupta PhD), Flinders University, Melbourne, VIC, Australia; Department of Epidemiology (P C Gupta DSc), Healis Sekhsaria Institute for Public Health, Navi Mumbai, India; Department of Preventive Cardiology & Medicine (Prof. R Gupta MD), Eternal Heart Care Centre & Research Institute, Jaipur, India; Department of Medicine (Prof. R Gupta MD), Mahatma Gandhi University Medical Sciences, Jaipur, India; Community and Family Medicine (S Gupta MD), Central Armed Police Forces Institute of Medical Sciences (aiims-capfims), Delhi, India; Department of Medicine (P Habibzadeh MD), University of Pittsburgh Medical Center, Pittsburgh, PA, USA; Asrat Woldeyes College of Health Sciences (Y G Haile PhD), Debre Berhan University, Debre Berhan, Ethiopia; Research and Publication (F Hakim MPH), World Health Organization (WHO), Dhaka, Bangladesh; Department of Public Health (S Hameed PhD), Green International University, Lahore, Lahore, Pakistan; Department of Epidemiology Population Biostatistics and Health Promotion (A Hargono PhD), Department of Public Health (F S Rahman PhD), Universitas Airlangga (Airlangga University), Surabaya, Indonesia; Department of Zoology and Entomology (A I Hasaballah PhD, M G M Zeariya PhD), Al-Azhar University, Cairo, Egypt; Faculty of Nursing (F Hasan PhD), Center of Excellence in Precision Medicine and Digital Health (T Porntaveetus PhD), Chulalongkorn University, Bangkok, Thailand; Department of Pharmaceutical Technology (I Hasan MPharm), Institute of Nutrition and Food Science (M Sultana PhD), University of Dhaka, Dhaka, Bangladesh; Department of Food Technology and Nutrition Science (T Hasan PhD), Noakhali Science and Technology University, Noakhali, Bangladesh; Department of Periodontics (N T Hashim PhD), Rak Medical & Health Sciences University, Ras Al Khaimah, United Arab Emirates; Department of Pharmaceutical Technology (Prof. M S Hasnain PhD), Adamas University, Kolkata, India; Department of Medicine (B Heibati PhD), University of Alberta, Edmonton, AB, Canada; Community-Oriented Nursing Midwifery Research Center (M Heidari PhD), Shahrekord University of Medical Sciences, Shahrekord, Iran; Departamento de Salud Oral (Department of Oral Health) (B Y Herrera-Serna PhD), Universidad Autónoma de Manizales (Autonomous University of Manizales), Manizales, Colombia; Graduate School of Medicine (Y Hiraike PhD), University of Tokyo, Tokyo, Japan; College of Medicine and Public Health (A M Hopkins PhD, B Kaambwa PhD, G R Naik PhD), Health Economics Unit (B Kaambwa PhD), Flinders University, Adelaide, SA, Australia; Maternal and Child Health Division (mchd) (L Hossain MPH), International Centre for Diarrhoeal Disease Research, Bangladesh, Dhaka, Bangladesh; Department of Public Health and Informatics (S Hossain PhD), Jahangirnagar University, Dhaka, Bangladesh; Institute of Research and Development (Prof. M Hosseinzade PhD, S Khoshvaght MSc, A Omar Bali PhD), School of Engineering & Technology (S Khoshvaght MSc), Institute for Global Health Innovations (C T Nguyen MPH, D C Nguyen MD, H L T Nguyen MPH), Faculty of Medicine (T Nguyen PhD), Duy Tan University, Da Nang, Viet Nam; School of Computer Science and Engineering (Prof. M Hosseinzade PhD), Galgotias University, Greater Noida, India; Department of Otorhinolaryngology Head and Neck Surgery (W Huang PhD), School of Medicine (Z Li PhD), Shanghai Jiao Tong University, Shanghai, China; Department of Biological Sciences and Chemistry (dbsc) (Prof. J Hussain PhD), University of Nizwa, Nizwa, Oman; Department of Occupational Safety and Health (Prof. B Hwang PhD), China Medical University, Taiwan, Taichung, Taiwan; Department of Biomedical, Metabolic, and Neural Science (L F Iannone MD), University of Modena and Reggio Emilia, Modena, Italy; West Africa Rcc (O S Ilesanmi PhD), Africa Centre for Disease Control and Prevention, Abuja, Nigeria; Faculty of Medicine (I M Ilic PhD), University of Belgrade, Belgrade, Serbia; Faculty of Medical Sciences (Prof. M D Ilic PhD), University of Kragujevac, Kragujevac, Serbia;

Department of Clinical Pharmacy (M Imam PhD), Department of Pharmaceutical Chemistry (M Kamal PhD), Department of Health and Rehabilitation Sciences (Prof. G Nambi PhD), Prince Sattam Bin Abdulaziz University, Al Kharj, Saudi Arabia; Institute of Health Research (M Immurana PhD), University of Health and Allied Sciences, Ho, Ghana; Faculty of Pharmacy (L M Irham PhD), Faculty of Public Health (Prof. S Solikhah DrPH), Universitas Ahmad Dahlan, Yogyakarta, Indonesia; International Institute of Migration and Development, Trivandrum, India (C Irshad PhD); School of Psychology and Public Health (M Islam PhD), La Trobe University, Melbourne, VIC, Australia; Department of Surveillance and Health Equity Science (F Islami PhD), American Cancer Society, Atlanta, GA, USA; One Health Research Group (Prof. J S Izquierdo-Condoy MSc), Universidad de Las Américas, Quito, Ecuador; Section of Economic & Social Sciences, Humanities & Arts (Prof. M Jakovljevic PhD), The World Academy of Sciences Unesco-twas, Trieste, Italy; Shaanxi University of Technology, Hanzhong, China (Prof. M Jakovljevic PhD); Division of Medical Research (R Janardhanan PhD), SRM School of Public Health (Prof. A Joseph PhD, J Samal MPH), Sri Ramaswamy Memorial Institute of Science and Technology, Kattankulathur, India; Department of Oral Medicine and Periodontology (Prof. R D Jayasinghe MS), University of Peradeniya, Peradeniya, Sri Lanka; Postgraduate Institute of Medicine (A U Jayatilleke PhD), Department of Anatomy, Genetics and Biomedical Informatics (Y Mathangasinghe PhD), Department of Surgery (D P Wickramasinghe MD), University of Colombo, Colombo, Sri Lanka; Faculty of Graduate Studies (A U Jayatilleke PhD), Institute for Violence and Injury Prevention, Colombo, Sri Lanka; Seoul National University Hospital, Seoul, South Korea (G Jeong MD); Department of Public Health Sciences (G Jeong MD), Seoul National University, Seoul, South Korea; Department of Public Health (Z Ji MD), Shanghai City Huangpu District Center for Disease Control and Prevention (Shanghai City Huangpu District Health Supervision Bureau), Shanghai, China; School of Biology and Engineering (S Jin MD), Guizhou Medical University, Guiyang, China; Nitte Gulabi Shetty Memorial Institute of Pharmaceutical Sciences (J Jose PhD), Ab Shetty Memorial Institute of Dental Sciences (K Rao PhD), Ks Hegde Medical Academy (Prof. P Shetty MD), Nitte University, Mangalore, India; Department of Economics (C E Joshua BSc), National Open University, Benin City, Nigeria; Research Department (Z Kabir PhD), TobaccoFree Research Institute Ireland, Dublin, Ireland; School of Public Health (Z Kabir PhD), University College Cork, Cork, Ireland; Department of Statistics (Prof. D H Kadir PhD), Salahaddin University-Erbil, Erbil, Iraq; Department of Business Administrations (Prof. D H Kadir PhD), Cihan University-Erbil, Erbil, Iraq; Independent Consultant, Pune, India (P V Kakodkar MDS); Department of Behavioral and Community Health (E Kalan PhD), University of Maryland, College Park, MD, USA; Department of Health Sciences (Prof. M Kanaan PhD), University of York, York, UK; School of Health and Environmental Science (Prof. J Kang PhD), Department of Biomedical Informatics (T Kwak BS), University College (Prof. U Saeed PhD), Korea University, Seoul, South Korea; Department of Anesthesia, Critical Care and Pain Medicine (Prof. J Kang PhD), Massachusetts General Hospital, Boston, MA, USA (M Kim MD); Office of the Executive Director (Prof. K K Kanmodi MPH), Cephas Health Research Initiative Inc., Ibadan, Nigeria; Department of Public Health (Prof. K K Kanmodi MPH), Thomas Adewumi University, Oko, Nigeria; Department of Periodontics (Prof. S Kapoor PhD), Shree Guru Gobind Tricentenary University, Gurgaon, India; Community and Primary Care Research Group (D Kar MD), Plymouth University, Plymouth, UK; School of Dentistry (N Karagezova BSc), Science Department (A Shamsutdinova MD), Department of Science (A Y Tazhiyeva PhD), Department of Prosthetic Dentistry (M Tleshev MSc), Kazakh National Medical University, Almaty, Kazakhstan; Amity Stem Cell Institute (Prof. M K Kashyap PhD), Amity University Haryana, Gurugram, India; National Center for Research, Khartoum, Sudan (Prof. A Khalid PhD); Primary Care Department (M A Khan MSc), NHS North West London, London, UK; Department of Pharmacy (M

Khan MPhil), Minhaj University Lahore, Lahore, Pakistan; International Center for Chemical and Biological Sciences (S Khan PhD), International Center for Chemical and Biological Sciences, Karachi, Pakistan; Department of Cardiology (Z Khan MD), University of South Wales, Treforest, UK; Department of Cardiology (Z Khan MD), University of Buckingham, Buckingham, UK; Department of Pharmacology (S U Khasbage MD), All India Institute of Medical Sciences, Raipur, India; Research Department (M Khosrowjerdi PhD), University of Inland Norway, Elverum, Norway; Ashok & Rita Patel Institute of Physiotherapy (P Khuman PhD), Charotar University of Science and Technology, Changa, India; Cardiovascular Disease Initiative (M Kim MD), Broad Institute of Mit and Harvard, Cambridge, MA, USA; Department of Medical Sciences (M A Kisiel PhD), Uppsala University Hospital, Uppsala, Sweden; Department of Public Health and Community Medicine (P Kodali PhD), Central University of Kerala, Kasaragod, India; Center for Tobacco Control Research and Education (P Kodali PhD), Department of Bioengineering and Therapeutical Sciences (Prof. M Zastrozhin PhD), University of California San Francisco, San Francisco, CA, USA; Department of General Practice and Family Medicine (Prof. O Korzh DSc), Kharkiv National Medical University, Kharkiv, Ukraine; Department of Anthropology (Prof. K Krishan PhD), Department of Forensic Science (Prof. V Sharma PhD), Panjab University, Chandigarh, India; Manikaka Topawala Institute of Nursing (D Krishnamurthy PhD), Ashok & Rita Patel Institute of Physiotherapy (B U Patel MPT), Department of Neuro Physiotherapy (M Soni PhD), Charotar University of Science and Technology, Anand, India; Parul Institute of Public Health (P S Kshtriya MPH), Parul University, Vadodara, India; School of Applied Science (C Kua PhD), Republic Polytechnic, Singapore, Singapore; School of Health Sciences (Prof. G Kuan PhD), Universiti Sains Malaysia, Kubang Kerian, Malaysia; School of Physical Education (Prof. G Kuan PhD), Henan University, Keifeng, China; Institute of Social and Preventive Medicine (Prof. C E Kuehni MD), Ispm University of Bern, Bern, Switzerland; University Children's Hospital, Inselspital (Prof. C E Kuehni MD), Department of Emergency Medicine (Prof. I Pantazopoulos PhD), University of Bern, Bern, Switzerland; Dental School (O Kujan PhD), Marshall Centre for Interventions in Infectious Disease (Prof. L Wang PhD), The University of Western Australia, Perth, WA, Australia; Department of Research Management (M Kulimbet MSc), Research Institute of Cardiology and Internal Diseases, Almaty, Kazakhstan; Health Research Institute (M Kulimbet MSc), Al Farabi Kazakh National University, Almaty, Kazakhstan; Department of Biochemistry (A Kumar PhD), Department of Dentistry (Prof. A Singh MDS), All India Institute of Medical Sciences, Bhopal, India; Center for Policy Research (D Kumar PhD), Indian Institute of Science Bangalore, Bangalore, India; Shaheed Mohtarma Benazir Bhutto Medical College Lyari (L Kumar MBBS), Dow University of Health Sciences, Karachi, Pakistan; Department of Community Medicine (M Kumar MD), ESIC Medical College and Hospital, Faridabad, India; Department of Community Medicine & School of Public Health (P Kumar PhD), Post Graduate Institute of Medical Education and Research, Chandigarh, India; Department of Anaesthesiology (T Kumar DNB), Rajendra Institute of Medical Sciences, Ranchi, India; Department of Pharmacy Practice (V Kumar PhD), National Institute of Pharmaceutical Education and Research (niper), Hajipur, Hajipur, India; Lincoln Institute of Rural and Coastal Health (O P Kurmi PhD), University of Lincoln, Lincoln, UK; Department of Medicine (O P Kurmi PhD), Department of Psychiatry and Behavioural Neurosciences (Prof. A T Olagunju PhD), McMaster University, Hamilton, ON, Canada; Faculty of Medicine and Health Science (M Kurniasari PhD), Universitas Kristen Satya Wacana (Satya Wacana Christian University), Salatiga, Indonesia; School of Nursing (M Kurniasari PhD), Taipei Medical University, Taipei, Taiwan; Division of Cardiology (K Kurpad MD), University of Texas, Galveston, TX, USA; Division of Evidence Synthesis (C Lahariya MD), Foundation for People-centric Health Systems, New Delhi, India; Division of Lifestyle Medicine (C Lahariya MD), Centre for Health: the Specialty Practice,

New Delhi, India; Department of Occupational and Environmental Health (H Lai PhD), School of Public Health (H Zhang PhD), Huazhong University of Science and Technology, Wuhan, China; Department of Respiratory and Critical Care Medicine (H Lai PhD), Northern Jiangsu People's Hospital, Yangzhou, China; Department of Psychology (Prof. T Lajunen PhD), Norwegian University of Science and Technology, Trondheim, Norway; Department of Public Health and Nutrition (A D Laksono DrPH), National Research and Innovation Agency Republic of Indonesia, Central Jakarta, Indonesia; Health Policy Department (A D Laksono DrPH), Airlangga Centre for Health Policy, Surabaya, Indonesia; Department of Public Health (Y M Larebo PhD), Wachemo University, Hossana Town, Ethiopia; Department of Otorhinolaryngology (Prof. S Lasrado MS), Father Muller Medical College, Mangalore, India; Department of Biohealth Industry (M Lee PhD), Yonsei University, Seoul, South Korea; Department of Precision Medicine (Prof. S Lee MD), Sungkyunkwan University, Suwon-si, South Korea; Department of Rheumatology and Immunology (H Li MD), The People's Hospital of Baoan Shenzhen, Shenzhen, China; The Nethersole School of Nursing (J Li PhD), The Chinese University of Hong Kong, Hong Kong, China; Beijing Anzhen Hospital (Q Li MD), Department of Neurosurgery (S Wang MD), Capital Medical University, Beijing, China; Department of Pulmonary Critical Care Medicine (S Li MD), Mayo Clinic, Phoenix, AZ, USA; Nutrition and Health Innovation Research Institute (X Li PhD), School of Medical and Health Sciences (X Li PhD), Edith Cowan University, Joondalup, WA, Australia; Discipline of Physiology (Y Lian MA), School of Medicine (P Luo MD), National University of Ireland - Galway, Galway, Ireland; Department of Medical Sciences (D Lindholm MD, Prof. J Sundström PhD), Uppsala University, Uppsala, Sweden; Department of General Surgery (B Liu MB), The First Affiliated Hospital of Anhui Medical University, Hefei, China; School of Life Sciences (G Liu PhD), University of Technology Sydney, Sydney, NSW, Australia; Department of Epidemiology and Biostatistics (Prof. J Liu PhD), Peking University, Beijing, China; School of Medicine (J López-Gil PhD), Universidad Espíritu Santo, Samborondón, Ecuador; Faculty of Health Sciences (J López-Gil PhD), Universidad Autónoma de Chile, Santiago, Chile; School of Medicine (Prof. G Lucchetti PhD), Federal University of Juiz de Fora, Juiz De Fora, Brazil; Department of Community and Family Medicine (A Ludhiadch PhD), Department of Dentistry (S S Rana MDS), Department of Psychiatry (J Singh MD), All India Institute of Medical Sciences, Bathinda, India; Dr. Mary and Ron Neal Cancer Center (Prof. H N Luu MD), Houston Methodist Research Institute, Houston, TX, USA; College of Engineering (Prof. M D Lytras PhD), Effat University, Jeddah, Saudi Arabia; Management of Information Systems Department (Prof. M D Lytras PhD), The American College of Greece, Aghia Paraskevi, Greece; Department of Periodontology (Prof. M Machoy-Rakoczy PhD), Pomeranian Medical University, Szczecin, Poland; Thamar University, Dhamar, Yemen (Prof. A A Madfa PhD); Department of One Health in Tropical Infectiousness Diseases (S A Mahamed MSc), Jigjiga University, Jigjiga, Ethiopia; College of Health Science (S A Mahamed MSc), Amoud University, Borama, Somalia; Department of Clinical and Hospital Pharmacy (M A Mahmoud PhD), Ashok and Rita Patel Institute of Physiotherapy, Al-Madinah Al-Munawarrah, Saudi Arabia; Rama Medical College Hospital and Research Centre, Uttar Pradesh, India (K Malhotra MBBS); Institute of Applied Health Research (K Malhotra MBBS), University of Birmingham, Birmingham, UK; Internal Medicine Department (L Manjani MD), MedStar Health, Washington, DC, USA; International Center for Chemical and Biological Sciences (F Mansoor MS), University of Karachi, Karachi, Pakistan; Department of Nutrition and Dietetics (M Martorell PhD), Centre for Healthy Living (M Martorell PhD), University of Concepción, Concepción, Chile; Department of Anatomy and Developmental Biology (Y Mathangasinghe PhD), Monash University, Clayton, VIC, Australia; Department of Community Medicine (M Mathur MD), Geetanjali Medical College and Hospital in Udaipur India, Udaipur, India; Department of Community Medicine (N Mathur MD), Apollo Institute of Medical Sciences and Research, Hyderabad, India;

Department of Healthcare (Prof. E A Mechili PhD), University of Vlora, Vlora City, Albania; Clinic of Social and Family Medicine (Prof. E A Mechili PhD), Department of Medicine (Prof. A Tsatsakis DSc), University of Crete, Heraklion, Greece; Centre for Health Innovation and Policy, Noida, India (Prof. R Mehrotra PhD); Department of Dental Research Cell (Prof. V Mehta PhD), Dr. D. Y. Patil University, Pune, India; Turning Point, Eastern Health Clinical School (S Melwani PhD), Monash University, Melbourne, VIC, Australia; Institute of Physiotherapy and Rehabilitation Sciences (A R Memon MPhil), Peoples University of Medical & Health Sciences for Women, Nawabshah (Shaheed Benazirabad), Pakistan; Institute for Health and Sport (A R Memon MPhil), Victoria University, Melbourne, VIC, Australia; Department of Medical Microbiology and Immunology (G A Menezes PhD), Trinity Medical Sciences University, St. Vincent, Saint Vincent and the Grenadines; Rak Medical and Health Sciences University (Prof. T H Merghani PhD), A.C.S. Medical College and Hospital, Rak, United Arab Emirates; University Centre Varazdin (T Mestrovic PhD), University North, Varazdin, Croatia; Department of Pharmacology (Prof. C D K Mettananda PhD), University of Kelaniya, Ragama, Sri Lanka; Clinical Medicine Department (Prof. C D K Mettananda PhD), Colombo North Teaching Hospital, Ragama, Sri Lanka; Department of Pathology (Prof. M M M Metwally PhD), Zagazig University, Zagazig, Egypt; Research Center for Public Health and Nutrition (M Mikrajab MPH), National Research and Innovation Agency, Bogor District, Indonesia; Multidisciplinary Department of Medical-Surgical and Dental Specialties (G Minervini PhD), University of Campania Luigi Vanvitelli, Naples, Italy; Internal Medicine (Prof. E M Mirrakhimov MD), Kyrgyz State Medical Academy, Bishkek, Kyrgyzstan; Department of Atherosclerosis and Coronary Heart Disease (Prof. E M Mirrakhimov MD), National Center of Cardiology and Internal Disease, Bishkek, Kyrgyzstan; Thumbay College of Management and Ai in Healthcare (V Mishra PhD), Gulf Medical University, Ajman, United Arab Emirates; Research and Development Department (V Mishra PhD), Panacea Institute of Interdisciplinary Research and Education, Varanasi, India; Department of Surgical Oncology (Prof. S Misra MCh), Department of Pharmacology (A Saravanan MD, M Shamim MBBS, S Singh MD), All India Institute of Medical Sciences, Jodhpur, India; Graduate School (F I Mohamed MPH), University of Hargeisa, Hargeisa, Somalia; Molecular Biology Unit (N S Mohamed MSc), Bio-Statistical and Molecular Biology Department (N S Mohamed MSc), Sirius Training and Research Centre, Khartoum, Sudan; Department of Psychological Science (Z Mohammed MSc), University of Vermont, Burlington, VT, USA; School of Health Sciences (S Mohan PhD), University of Petroleum and Energy Studies, Dehradun, India; Arh Hematology Oncology (N Moka MD), Appalachian Regional Healthcare, Middlesboro, KY, USA; Department of Hematology Oncology (N Moka MD), Lincoln Memorial University, Harrogate, TN, USA; Department of Public Health (Prof. R S Moreira PhD), Oswaldo Cruz Foundation, Recife, Brazil; Department of Public Health (Prof. R S Moreira PhD), Federal University of Pernambuco, Recife, Brazil; Unit of Pharmacotherapy, Epidemiology and Economics (Prof. S Mubarik PhD), University of Groningen (Rijksuniversiteit Groningen), Groningen, Netherlands; Department of Epidemiology and Biostatistics (Prof. S Mubarik PhD, Prof. C Yu PhD), School of Public Health (Prof. Z Zhang PhD), Wuhan University, Wuhan, China; Department of Medicine (A Mukhopadhyay MD), National University Health System, Singapore, Singapore; Department of Health Economics (M Muniyandi PhD), Department of Epidemiology (M Sathya Narayanan MBBS), National Institute for Research in Tuberculosis, Chennai, India; Pharmacy Practice (K Murti PhD), Pharmaceutical Analysis (A Rai PhD), National Institute of Pharmaceutical Education and Research, Hajipur, Hajipur, India; Community Medicine (V Murugan MD), Indira Gandhi Medical College and Research Institute, Puducherry, India (A Saravanan MD); School of Public Health (D Musoke PhD), Makerere University, Kampala, Uganda; Department of Research Methods (S Muthu PhD), Orthopaedic Research Group, Coimbatore, India; Central Research Laboratory

(S Muthu PhD), Vinayaka Mission's Research Foundation (Deemed to Be University), Puducherry, India; Academy of Scientific and Innovative Research (AcSIR), Ghaziabad, India (K Nagarajan PhD); Department of Epidemiology, Nyu School of Global Public Health (K P Nagdeo DDS), New York University, New York, NY, USA; Department of Computer Science and It (G R Naik PhD), Torrens University, Adelaide, SA, Australia; Suraj Eye Institute, Nagpur, India (V Nangia PhD); Department of Restorative and Prosthetic Dental Sciences (M Nassani PhD), Dar Al Uloom University, Riyadh, Saudi Arabia; Kent State University (K Nayak MPH), Kent State University, Kent, OH, USA; Internal Medicine Department (A K Ndakotsu MD), MedStar Health, Baltimore, MD, USA; Department of Medicine (C J Sabet MA), Georgetown University, Washington, DC, USA (A K Ndakotsu MD); School of Pharmacy (S O Nduaguba PhD), West Virginia University, Morgantown, WV, USA; Faculty of Public Health (T Nguyen PhD), Vnu University of Medicine and Pharmacy, Hanoi, Viet Nam; School of Medicine (V Niranjan PhD), University of Limerick, Limerick, Ireland; Department of Radiology (F Nugen PhD), Mayo Clinic, Rochester, MN, USA; School of Information (F Nugen PhD), University of California Berkeley, Berkeley, CA, USA; Research Center for Public Health and Nutrition (W P Nugraheni PhD), National Health Research Institutes, Jakarta, Indonesia; School of Health Sciences (N Nurelhuda PhD), Hamdan Bin Mohammed Smart University, Dubai, United Arab Emirates; Dalla Lana School of Public Health (N Nurelhuda PhD), University of Toronto, Toronto, ON, Canada; Department of Applied Economics and Quantitative Analysis (Prof. B Oancea PhD), University of Bucharest, Bucharest, Romania; Bioinformatics Department (Prof. B Oancea PhD), National Institute of Research and Development for Biological Sciences, Bucharest, Romania; Department of Biomedicine and Prevention (F Oddi PhD), University of Rome "Tor Vergata", Rome, Italy; Department of Veterinary Public Health and Preventive Medicine (I A Odetokun PhD), University of Ilorin, Ilorin, Nigeria; Department of Physiotherapy (O O Ojedoyin PhD), Federal University of Health Sciences Ila Orangun Osun, Nigeria, Ila-Orangun, Nigeria; Department of Physiotherapy (O O Ojedoyin PhD), University of KwaZulu-Natal, Durban, South Africa; School of Pharmacy (O C Okonji MSc), University of the Western Cape, Cape Town, South Africa; Department of Psychiatry (Prof. A T Olagunju PhD), University of Lagos, Lagos, Nigeria; Department of Public Health (J Olickal PhD), Amrita Institute of Medical Sciences, Kochi, India; International Center for Materials Sciences and Technology (A Omar Bali PhD), Western Caspian University, Baku, Azerbaijan; Biochemistry, Pharmacology and Toxicology Department (H Onohuean PhD), Kampala International University, Western Campus, Iahaka-Bushenyi, Uganda; Bridgeport Hospital, Department of Internal Medicine (M C Oparah MD), Yale New Haven Health/Bridgeport Hospital, Bridgeport, CT, USA; Centre of Regenerative Medicine (M Ordak PhD), Medical University of Bialystok, Bialystok, Poland; Department of Biological Sciences (A Osborne MSc), Njala University, Freetown, Sierra Leone; Division of Medicine (T Oyelade PhD), University College London, London, UK; School of Medicine (T Oyelade PhD), Keele University, Keele, UK; National School of Public Health (A Padron-Monedero PhD), Institute of Health Carlos Iii, Madrid, Spain; Department of Mental Health (R Palma-Alvarez PhD), Hospital Universitari Vall d'Hebron (cibersam), Barcelona, Spain; Biomedical Network Research Centre On Mental Health (cibersam), Barcelona, Spain (R Palma-Alvarez PhD); Kalinga Institute of Industrial Technology Bhubaneswar (A K Panda PhD), Kalinga Institute of Industrial Technology in India, Bhubaneswar, India; School of Sciences (Prof. D Pande Katare PhD), School of Sciences, Noida International University, Greater Noida, India; Research Department (A Pandey MPH), Public Health Research Society Nepal, Kathmandu, Nepal; The Royal Marsden Nhs Foundation Trust (S R Pandey PhD), The Royal Marsden Nhs Foundation Trust, London, England; Faculty of Science and Engineering (S R Pandey PhD), Anglia Ruskin University, London, UK; Department of Emergency Medicine (Prof. I Pantazopoulos PhD), University of Thessaly, Larissa, Greece; Vision and Eye

Research Institute (Prof. S Pardhan PhD), Anglia Ruskin University, Cambridge, UK; Department of Epidemiology (T Parekh PhD), University of Delaware, Newark, DE, USA; Division of Health Policy and Management (R R Parikh MD), University of Minnesota, Minneapolis, MN, USA; Forensic Medicine and Toxicology (P Parmar MD), All India Institute of Medical Sciences, Hyderabad, India; Department of Medical Sciences (R Passera PhD), University of Torino, Torino, Italy; Department of Imaging (R Passera PhD), AOU Città della Salute e della Scienza di Torino (AOU City of Health and Science of Turin), Torino, Italy; Zoology Section (S Patel PhD), Regional Institute of Education (rie), Ajmer, India; Ingenus Pharmaceuticals, Llc (S Patel MS), Long Island University, Brooklyn, Fairfield, NJ, USA; Department of Physiology (A Patil MD), College of Nursing (R V R MSc), All India Institute of Medical Sciences, Nagpur, India; Second Propedeutic Department of Internal Medicine (Prof. D Patoulias PhD), Aristotle University of Thessaloniki, Thessaloniki, Greece; Faculty of Humanities and Social Sciences (U Paudel PhD), Department of Community Medicine and Public Health (P M S Pradhan MD), Tribhuvan University, Kathmandu, Nepal; Australian Institute of Health Innovation (P Peprah MSc), Macquarie University, Sydney, NSW, Australia; School of Population Health (Prof. G Pereira PhD), Curtin University, Bentley, WA, Australia; Centre for Fertility and Health (Prof. G Pereira PhD), Norwegian Institute of Public Health, Oslo, Norway; Mathematical and Computer Sciences (O Peter PhD), University of Medical Sciences, Ondo, Ondo, Nigeria; College of Health Sciences (T T Pham PhD), VinUniversity, Hanoi, Viet Nam; Research Advancement Consortium in Health, Hanoi, Viet Nam (T T Pham PhD); Department of Promoting Health, Maternal-Infant, Excellence and Internal and Specialized Medicine (promise) G. D'Alessandro (E Pirera MD), University of Palermo, Palermo, Italy; Department of Epidemiology and Evidence-Based Medicine (R V Polibin PhD), I.M. Sechenov First Moscow State Medical University, Moscow, Russia; Department of Data Management and Analysis (R Poluru PhD), The Inclen Trust International, New Delhi, India; Dental Public Health (P Porwal MSc), Pushpagiri Institute of Medical Sciences, Tiruvella, India; Department of Maternal-Child Nursing and Public Health (E J S Prates BS), Federal University of Minas Gerais, Belo Horizonte, Brazil; Department of Biostatistics, Epidemiology, and Informatics (J Puvvula PhD), University of Pennsylvania, Philadelphia, PA, USA; Department of Neonatology (I Qattea MD), Case Western Reserve University, Akron, OH, USA; Oman Dental College, Oman (Prof. R A Radhakrishnan PhD); Department of Medical Oncology (Prof. V Radhakrishnan MD), Cancer Institute (w.i.a), Chennai, India; Department of Epidemiology (P Raghuveer MD), National Institute of Mental Health and Neurosciences, Bengaluru, India; Department of Medical Biology (Prof. F Rahim PhD), Kocaeli University, Izmit, Türkiye; Internal Medical Faculty (Prof. F Rahim PhD), Osh State University, Osh, Kyrgyzstan; Department of Environmental Health Engineering (S Rahimi PhD), Health Sciences Research Center (S Rahimi PhD, M Sarmadi MSc), Torbat Heydariyeh University of Medical Sciences, Torbat Heydariyeh, Iran; Rural Health Research Institute (M Rahman MPH), Charles Sturt University in Australia, Orange, NSW, Australia; Infectious Disease Division (M Rahman MPH), International Centre for Diarrhoeal Disease Research, Dhaka, Bangladesh; College of Medicine and Health Sciences (M Rahman PhD), National University of Science and Technology, Sohar, Oman; Department of Population Science and Human Resource Development (Prof. M Rahman DrPH), University of Rajshahi, Rajshahi, Bangladesh; Dr. Rajendra Prasad Government Medical College, Tanda, Kangra, India (Prof. S K Raina MD); Department of Population Health (M Ramadan DrPH), King Saud Bin Abdulaziz University for Health Sciences, Jeddah, Saudi Arabia; Department of Midwifery (K Ramadhan MPH), Ministry of Health of the Republic of Indonesia, Palu, Indonesia; Department of Radiology (S Ramasamy MD), Stanford University, Stanford, CA, USA; Department of Biotechnology (Prof. P W Ramteke PhD), Hislop College, Nagpur, India; Department of Molecular Biology & Genetic Engineering

(Prof. P W Ramteke PhD), Rtm Nagpur University, Nagpur, India; All India Institute of Medical Sciences, Bilaspur, India (L Rana MD); Department of Research (C L Ranabhat PhD), Eastern Scientific Llc, Richmond, KY, USA; Planetary Health Research Centre (phrc), Kathmandu, Nepal (C L Ranabhat PhD); Department of Oral Pathology, Microbiology and Forensic Odontology (S J Rao MDS), Sharavathi Dental College and Hospital, Shimogga, India; Department of Family Medicine (Prof. D Rathish PhD), Department of Community Medicine (N Wickramasinghe MD), Rajarata University of Sri Lanka, Anuradhapura, Sri Lanka; Siksha 'o' Anusandhan University (Prof. T R Rautray PhD), Siksha o Anusandhan University, Bhubaneswar, India; Academic Public Health England (Prof. S Rawaf MD), Public Health England, London, UK; School of Environment (M Rezaei PhD), Tehran University, Tehran, Iran; Non-communicable Diseases Research Center (N Rezaei MD), Tehran University of Medical Sciences, Tehran, Iran; Department of Community Medicine (R Rohilla MD), Government Medical College, Chandigarh, India; Centre for Healthy Brain Ageing (CHeBA) (S Röhr PhD, S Röhr PhD), School of Population Health (Prof. B A Saddik PhD), University of New South Wales, Sydney, NSW, Australia; Department of Population Health (Prof. C Rojanaworarit PhD), Hofstra University, Hempstead, NY, USA; Department of Analytical and Applied Economics (Prof. H Rout PhD, C Swain MPhil), Rusa Centre of Excellence in Public Policy and Governance (Prof. H Rout PhD), Utkal University, Bhubaneswar, India; Research Department (S Roy MSc), Indian Institute of Public Health, Delhi, India; Department of Oral and Maxillofacial Surgery (C S N PhD), Jagadguru Sri Shivarathreeswara University, Mysore, India; Department of Pharmaceuticals (Prof. M Sachdeva Dhingra PhD), Bihar College of Pharmacy, Patna, India; Department of Public Health and Epidemiology (Prof. B A Saddik PhD), Khalifa University, Abu Dhabi, United Arab Emirates; Department of Pharmaceutical Chemistry (Prof. M Saeb PhD), International Medical University, Gdańsk, Poland; Széchenyi István University, Győr, Hungary (Prof. U Saeed PhD); Department of Psychosocial Science (Prof. D Sagoe PhD), University of Bergen, Bergen, Norway; ICMR - National Institute for Research in Bacterial Infections (icmr Nirbi) (Prof. I Saha PhD), Indian Council of Medical Research, Kolkata, India; Biotechnology Research Center (Prof. A Sahebkar PhD), Mashhad University of Medical Sciences, Mashhad, Iran; Centre for Biotechnology (A Sahoo PhD), Siksha o Anusandhan (Deemed to Be University), Bhubaneswar, India (A K Verma PhD); Department of Statistics (M R Sajid PhD), University of Gujrat, Gujrat, Pakistan; LMU-Munich, Munich, Germany (J W Sakshaug PhD); Institute for Employment Research, Nuremberg, Germany (J W Sakshaug PhD); Cephas Health Research Initiative Inc, Ibadan, Nigeria (A A Salami BDS); Department of Entomology (A M Samy PhD), Medical Ain Shams Research Institute (masri) (A M Samy PhD), Ain Shams University, Cairo, Egypt; CVM Nursing College (Prof. S S Sarate MScNursing), The Charutar Vidya Mandal (CVM) University, Anand, India; Department of Public Health (Y Sarikhani PhD), Jahrom University of Medical Sciences, Jahrom, Iran; Faculty of Science (M Sarmadi MSc), Queensland University of Technology, Brisbane, QLD, Australia; Clinical Studies and Trials Unit (Development Research) (A Satija PhD), Indian Council of Medical Research, New Delhi, India; Department of Pharmacology (S Satyam PhD), Rak Medical and Health Sciences University, Ras Al Khaimah, United Arab Emirates; Department of Preventive and Social Medicine (G Saya MD), Jawaharlal Institute of Postgraduate Medical Education and Research, Puducherry, India; Universidad Esan (Prof. C Schinckus PhD), Universidad Esan, Lima, Peru; Graduate School of Business (Prof. C Schinckus PhD), University of the Fraser Valley, Abbotsford, BC, Canada; Faculty of Medicine (A Schuermans BSc), Department of Cardiovascular Sciences (A Schuermans BSc, J Van Den Eynde BSc), Department of Public Health and Primary Care (H Shi MD), Katholieke Universiteit Leuven, Leuven, Belgium; Faculty of Dentistry (Prof. S Selvaraj PhD), University of Puthisastra, Phnom Penh, Cambodia; Emergency Department (S Senthilkumaran PhD), Manian Medical Centre, Erode, India; Department of Medicine (Y

Sethi MD), Swami Vivekanand Subharti University, Meerut, India; Department of Global Public Health (A N Shaaban PhD), Karolinska Institute, Stockholm, Sweden; Department of Pharmacy Practice (M Shabil MPharm), Ms Ramaiah University of Applied Sciences, Bangalore, India; Rita A. Patel Institute of Physiotherapy (S Shah PhD), The Charutar Vidya Mandal (cvm) University, Anand, India; Department of Pathology and Laboratory Medicine (S Sham MD), Northwell Health, New York, NY, USA; Department of Forensic Science (Prof. B K Sharma PhD), Shree Guru Gobind Singh Tricentenary University, Gurugram, India; Department of Social and Behavioral Health (Prof. M Sharma PhD), University of Nevada Las Vegas, Las Vegas, NV, USA; Department of University Institute of Biotechnology (R Sharma PhD), Chandigarh University, Punjab, India; International Agency for Research On Cancer, Lyon, France (M Sheikh PhD); Alva's Insitute of Medical Sciences & Research Centre (Prof. M Shetty MD), Rajiv Gandhi University of Health Sciences, Moodubidire, India; Department of Orthodontics (S Shirazi DDS), Henry S.Goldman School of Dental Medicine (V Vikram MDS), Boston University, Boston, MA, USA; Washington State University, Spokane, WA, USA (Prof. K Shishani PhD); Department of Veterinary Public Health and Preventive Medicine (A Shittu MSc), Usmanu Danfodiyo University, Sokoto, Sokoto, Nigeria; Oulu Business School (I Shiue PhD), Martti Ahtisaari Institute (I Shiue PhD), University of Oulu, Oulu, Finland; Department of Epidemiology and Public Health (A Shrestha MGS), University of Maryland School of Medicine, Baltimore, MD, USA; Department of Research and Academics (S Shrestha PhD), Kathmandu Cancer Center, Bhaktapur, Nepal; Person-Centered Research (S Shrestha PhD), Monash University, Box Hill, VIC, Australia; Department of Biotechnology and Microbiology (D Shrivastav PhD), Noida International University, Greater Noida, India; Kenneth H. Cooper Institute (Prof. K Shuval PhD), Texas Tech University Health Sciences Center, Dallas, TX, USA; School of Public Health (N Sidamo PhD), Department of Midwifery (G Temesgen MSc), Arba Minch University, Arba Minch, Ethiopia; Department of Microbiology (A Singh PhD), Department of Zoology (Prof. B Vellingiri PhD), Central University of Punjab, Bathinda, India; Department of Agriculture and Environmental Sceinces (Prof. B P Singh PhD), National Institute of Food Technology Entrepreneurship and Management-Kundli (niftem-k), Sonipat, India; University Centre for Research and Development (ucrd) (B Singh PhD), Chandigarh University, Mohali, India; School of Medicine (Prof. J A Singh MD), Baylor College of Medicine, Houston, TX, USA; Department of Medicine Service (Prof. J A Singh MD), Us Department of Veterans Affairs (va), Houston, TX, USA; National Institute of Cancer Prevention and Research, Noida, India (P Singh PhD); Department of Pulmonary Medicine (Prof. V Singh MD), Mahaveer Jaipuria Rajasthan Hospital, Jaipur, India; SRM Medical College Hospital and Research Centre (G Sivan PhD), Sri Ramaswamy Memorial Institute of Science and Technology, Chengalpettu, India; Bahrain Defence Force Royal Medical Services (G Sivaramakrishnan MSD), Bahrain Defence Force Royal Medical Services in Bahrain, Riffa, Bahrain; Books Committee (V Y Skryabin MD), Royal College of Psychiatrists, London, UK; Department of Development Studies (M Sohel MPH), Daffodil International University, Dhaka, Bangladesh; School of Public Health (L K Solbana MPH), Wollega University, Nekemte, Ethiopia; Hospital Universitario de la Princesa (Prof. J B Soriano MD), Autonomous University of Madrid, Madrid, Spain; Centro de Investigación Biomédica En Red Enfermedades Respiratorias (Center for Biomedical Research in Respiratory Diseases Network), Madrid, Spain (Prof. J B Soriano MD); Department of Public Health (M Sorrentino MD), University of Naples "Federico Ii", Naples, Italy; Department of Public Health, Experimental and Forensic Medicine (M Sorrentino MD), University of Pavia, Pavia, Italy; Research and Development Cell (V Srivastava PhD), Lovely Professional University, Delhi, India; Department of Medicine (P Steiropoulos MD), Democritus University of Thrace, Alexandroupolis, Greece; School of Public Health (Z Sun PhD), Kunming Medical University, Kunming, China; North Eastern Indira Gandhi Regional Institute of Health and Medical

Sciences (neigrihms), Shillong, India (S Sundaram MD); The George Institute for Global Health, Sydney, NSW, Australia (Prof. J Sundström PhD); Community Medicine (A Tambi MBBS), Mahatma Gandhi Institute of Medical Sciences, Sevagram, India; Department of Dermatology (M Tampa PhD), Carol Davila University of Medicine and Pharmacy, Bucharest, Romania; Department of Dermato-Venereology (M Tampa PhD), Dr. Victor Babes Clinical Hospital of Infectious Diseases and Tropical Diseases, Bucharest, Romania; Department of Otorhinolaryngology–Head & Neck Surgery (B Tan MBBS), Singapore General Hospital, Singapore, Singapore; National Research and Innovation Agency, Jakarta, Indonesia (I U Tarigan PhD); Department of Preventive Medicine (M Teramoto MD, Y Wang MS), Northwestern University, Chicago, IL, USA; Department of Internal Medicine (T H Tran MD), University of Medicine and Pharmacy at Ho Chi Minh City, Ho Chi Minh City, Viet Nam; Department of Business Analytics (T H Tran MD), University of Massachusetts Dartmouth, Dartmouth, MA, USA; Department of Clinical and Experimental Medicine (D Trico MD), University of Pisa, Pisa, Italy; Human Development and Health Science Faculty (Prof. A Tsatsakis DSc), Universidad Ecotec in Ecuador, Guayaguil, Ecuador; Department of Psychiatry (E Tsermpini PhD), Dalhousie University, Halifax, NS, Canada; Department of Occupational Health and Safety (A R Tualeka PhD), University of Development, Surabaya, Indonesia; Hayatabad Medical Complex (H Ullah FCPS), Postgraduate Medical Institute, Peshawar, Pakistan; Department of Cardiovascular, Endocrine-metabolic Diseases and Aging (B Unim PhD), National Institute of Health, Rome, Italy; Department of Psychiatry (A Usman Sambo FWACP), Federal Neuropsychiatric Hospital, Kaduna, Nigeria; Irfan Suat Gunsel Operational Research Institute (B Uzun PhD), Near East University, Nicosia, Türkiye; Department of Orthodontics (H Uzunçıbuk PhD), University of Trakya, Edirne, Türkiye; Department of Community Medicine (S Vajjala MD), Government Medical College Yadadri, Yadadri, India; Department of Epidemiology and Public Health (S Vajjala MD), Sankhyatraya Science and Research Association, New Delhi, India; Department of Psychology (Z Vally PhD), Zayed University, Abu Dhabi, United Arab Emirates; Department of Biochemistry (S Vasishta PhD), Apollo Institute of Medical Sciences and Research Chittoor, Chittoor, India; Department of Human Genetics & Molecular Biology (Prof. B Vellingiri PhD), Bharathiar University, Coimbatore, India; Raffles Neuroscience Centre (Prof. N Venketasubramanian MSc), Raffles Hospital, Singapore, Singapore; Digital Health Research Center, Lima, Peru (D Villarreal-Zegarra MPH); Universidad Científica Del Sur, Lima, Peru (D Villarreal-Zegarra MPH); School of Agriculture and Food Sustainability (Prof. L Wang PhD), The University of Queensland, Brisbane, QLD, Australia; Department of Neurosurgery (S Wang MD), Beijing Tiantan Hospital, Beijing, China; Department of Orthopaedics (F Wei PhD), General Hospital of Central Theater Command, Wuhan, China; Fourth Military Medical University, Xi'an, China (F Wei PhD); Division of Intramural Research (F Williams PhD), National Institutes of Health, Rockville, MD, USA; Public Health Sciences Division (H Xiao PhD), Statistics and Data Management Center (H Xiao PhD), Fred Hutchinson Cancer Research Center, Seattle, WA, USA; Department of Intelligent Medical Engineering (Prof. W Xie DrPH), Anhui Medical University, Anhui, China; Department of Surgery (Prof. W Xie DrPH), The First Affiliated Hospital of Anhui Medical University, Hefei, Anhui, China; Department of Gastroenterology (K Yang MD), Beijing Friendship Hospital Affiliated to Capital Medical University, Beijing, China; Department of Health Management (A Yiğit PhD, Prof. V Yiğit PhD), Süleyman Demirel University, Isparta, Türkiye; Faculty of Epidemiology and Population Health (B A Yirdaw PhD), London School of Hygiene & Tropical Medicine, London, UK; Department of Community Medicine (S Yogeshkumar MD), Jawaharlal Nehru Medical College, Belagavi, India; Department of Pediatrics (Prof. D Yon MD), Kyung Hee University, Seoul, South Korea; Department of Biostatistics (Prof. N Yonemoto PhD), University of Toyama, Toyama, Japan; Department of Public Health (Prof. N Yonemoto PhD), Juntendo University, Tokyo, Japan; School of

Dentistry (Prof. M S Zafar PhD), University of Jordan, Amman, Jordan; Department of Environmental and Occupational Health (E Zainal Abidin PhD), Universiti Putra Malaysia, Serdang, Malaysia; Faculty of Medicine and Health Sciences (F Zakham PhD), Hodeidah University, Hodeidah, Yemen; Centre for Public Health and Wellbeing (H Zandian PhD), University of the West of England, Bristol, UK; Department of Administration (Prof. M Zastrozhin PhD), PGxAI, San Francisco, CA, USA; Mohammed Vi International School of Public Health (Prof. S Zbiri PhD), Mohammed Vi University of Sciences and Health (um6ss), Casablanca, Morocco; Institute for Healthcare System Analysis (ia2s), Paris, France (Prof. S Zbiri PhD); Department of Pediatrics and Child Health Nursing (A Zemariam MSc), Woldia University, Woldia, Ethiopia; Tianjin Medical University General Hospital (Z Zhang MD), Tianjin Centers for Disease Control and Prevention, Tianjin, China; Department of Hepatology (Prof. M Zheng MD), Wenzhou Medical University, Wenzhou, China; Department of Psychiatry (C Zhou MD), University of Ottawa, Ottawa, ON, Canada

Authors' Contributions

Managing the overall research enterprise

Erin O'Connell, Emmanuela Gakidou, Erin Mullany

Writing the first draft of the manuscript

Gabriela Gil, Luisa Flor, Brooks Morgan

Primary responsibility for applying analytical methods to produce estimates

Brooks Morgan, Gabriela Gil, Luisa Flor, Xiaochen Dai

Primary responsibility for seeking, cataloguing, extracting, or cleaning data; designing or coding figures and tables

Jason Anderson, Oliver McElvaney, Brooks Morgan, Gabriela Gil

Providing data or critical feedback on data sources

Bhoomadevi A, Rashad Abdul-Ghani, Rizwan Suliankatchi Abdulkader, Olugbenga Olusola Abiodun, Ahmed Abu-Zaid, Fuad Hamdi A. Abuadas, Mohammad H Abuadas, Anirudh Balakrishna Acharya, Apurba Acharya, Swetha Acharya, Muhammad Sohail Afzal, Feleke Doyore Agide, Sajjad Ahmad, Ayman Ahmed, MD. Faisal Ahmed, Naveed Ahmed, Shahzaib Ahmed, Tomi Akinyemiju, Omar Al Omari, Yazan Al Thaher, Rasmieh Mustafa Al-Amer, Sabah Al-Marwani, Ahmad Rajeh Al-Qudimat, Ali Salman Al-Shami, Turki M Alanzi, Abdelazeem M Algammal, Khalid F Alhabib, Amidu Alhassan, Ashraf Alhumaidi, Syed Shujait Ali, Syed Mohamed Aljunid, Sameer Abdulmalik Alkubati, Wesam Taher Almagharbeh, Margret Beaula Alocious Sukumar, Jaber S Alqahtani, Awais Altaf, Hassan Alwafi, Hany Aly, Tarek Tawfik Amin, Jason A Anderson, A.H. Sruthi Anil Kumar, Kabilan Annadurai, Saeid Anvari, Razique Anwer, Tahira Ashraf, Muhammad Shahzad Aslam, Yuni Asri, Seyyed Shamsadin Athari, Alok Atreya, Avinash Aujayeb,

Manal Awad, Ahmad Ayed, Syed Babar Jamal Bacha, Riadh Badraoui, Khlood K Baghlaf, Abdul Rasheed Bahar, Yasemin Bahar, Raju Balaji, Yatan Pal Singh Balhara, Hiba Jawdat Barqawi, Amadou Barrow, MD Abu Bashar, Uzma Iqbal Belgaumi, Kelemu Zelalem Berhanu, Sonu Bhaskar, Sudip Bhattacharya, Bijit Biswas, Archith Boloor, Dejana Braithwaite, Senthilkumar C, Sandra Carvalho, Joao Mauricio Castaldelli-Maia, Vijay Kumar Chattu, Sirshendu Chaudhuri, Akhilanand Chaurasia, Saravanan Chinnaiyan, Bryan Chong, Dinh-Toi Chu, Sunghyun Chung, Natalia Cruz-Martins, Xiaochen Dai, Gloria Dalla Costa, Lalit Dandona, Rakhi Dandona, Samuel Demissie Darcho, Ismail Dergaa, Vinoth Gnana Chellaiyan Devanbu, Kimia Didehvar, Hoa Thi Do, Ojas Prakashbhai Doshi, Jiang Du, Emeka W. Dumbili, Senbagam Duraisamy, Jasmin Dönicke, Michael Ekholuenetale, Suhaib El Khatib, Iman El Sayed, Ashraf A El-Metwally, Abdelgawad Salah Eltahawy, Collins Nduka Esomchi, Devaraj Ezhilarasan, Adeniyi Francis Fagbamigbe, Carla Sofia e Sá Farinha, Alireza Feizkhah, Anteneh Fikrie, Luisa S Flor, Muktar A Gadanya, Emmanuela Gakidou, Balasankar Ganesan, Balasubramanian Ganesh, Fernando Jr. Barroga Garcia, Haftay Gebremedhin, Sailaja Ghimire, Sama Ghoba, Ehsan Gholami, Zainab Gholami, Thinh Gia Gia Nguyen, Gabriela Fernanda Gil, Pouya Goleij, Divya Gopinath, Bin Guan, Zenawi Hagos Gufue, Damitha Asanga Gunawardane, Sasidhar Gunturu, Bhawna Gupta, Prakash C Gupta, Yonas Deressa Guracho, Gamze Gökulu, Yohannes Gebreegziabher Haile, Ferdous Hakim, Nasrin Hanifi, Faizul Hasan, Towhid Hasan, Ahmad Shoeb Hashmi, Simon I Hay, Zaw Myo Hein, Mohamed Fawzi Hemida, Sahadat Hossain, Mehdi Hosseinzade, Weijun Huang, Luigi Francesco Iannone, Segun Emmanuel Ibitoye, Olayinka Stephen Ilesanmi, Lalu Muhammad Irham, Juan S Izquierdo-Condoy, Jaison Jacob, Abhishek Jaiswal, Mihajlo Jakovljevic, Rajiv Janardhanan, Achala Upendra Jayatilleke, Bijay Mukesh Jeswani, Jobin Jose, Alex Joseph, Charity Ehimwenma Joshua, Hema Shree K, Billingsley Kaambwa, Zubair Kabir, Vidya Kadashetti, Dler Hussein Kadir, Pradnya Vishal Kakodkar, Mona Kanaan, Jiseung Kang, Shalini Kapoor, Neila Karagezova, Mohmed Isaqali Karobari, Manoj Kumar Kashyap, Maseer Khan, Moien AB Khan, Muhammad Umer Khan, Zahid Khan, Zelal Kharaba, Sameer Uttamaro Khasbage, Samira Khoshvaght, P Ratan Khuman, Min Seo Kim, Marta A. Kisiel, Shivakumar KM, Oleksii Korzh, Archana Koul, Sindhura Lakshmi Koulmane Laxminarayana, Kewal Krishan, Chong-Han Kua, Omar Kujan, Dhasarathi Kumar, Diwakar Kumar, G Anil Kumar, Laksh Kumar, Mohan Kumar, V Udaya Kumar, Maria Dyah Kurniasari, Taeho Kwak, Chandrakant Lahariya, Yilma Markos Larebo, Savita Lasrado, Munjae Lee, Seung Won Lee, Hui Li, Jiaying Li, Qiang Li, Zhengrui Li, Yanxue Lian, Bingsheng Liu, Gang Liu, Jue Liu, Pincheng Luo, Hung N Luu, Miltiadis D Lytras, Monika Machoy-Rakoczy, Samatar Abshir Mahamed, Kashish Malhotra, Lokesh Manjani, Medha Mathur, Enkeleint A Mechili, Jitendra Kumar Meena, Vini Mehta, Satish Melwani, Godfred Antony Menezes, Ritesh G Menezes, Tarig H Merghani, Erkin M Mirrakhimov, Tridip Mitra, Farduus Ibraahim Mohamed, Nouh Saad Mohamed, Zaidan Mohammed, Ali H Mokdad, Hirbaye Mokona, Rohith Motappa, Sumaira Mubarik, Amartya Mukhopadhyay, Christopher J L Murray, David Musoke, Ganesh R Naik, Gopal Nambi, Shumaila Nargus, Zuhair S Natto, Biswa Prakash Nayak, Kinjal Nayak, Cuong Tat Nguyen, Duy Cao Nguyen, Huong Lan Thi Nguyen, Trang Nguyen, Fred Nugen, Bogdan Oancea, Ismail A Odetokun, Olusegun Olatunji Ojedoyin, Osaretin Christabel Okonji, Andrew T Olagunju, Ahmed Omar Bali, Hope Onohuean, Mahesh P A, Alok Kumar Panda, Deepshikha Pande Katare, Romil R Parikh, Shivani Patel, Ashlesh Patil, Uttam Paudel, Shrikant Pawar, Prince Peprah, Gavin Pereira, Ramesh Poluru, Thantrira Porntaveetus, Amit Porwal, Priyanka Porwal, Elton Junio Sady Prates, Bharathi M Purohit, Jagadeesh Puvvula, Raghu Anekal Radhakrishnan, Venkatraman Radhakrishnan, Pracheth Raghuveer, Fakher Rahim, Sajjad Rahimi, Firman Suryadi Rahman, Mohammad Hifz Ur Rahman, Amita Rai, Sunil Kumar Raina, Jeffrey Pradeep Raj, Mahmoud Mohammed Ramadan, Kadar Ramadhan, Shakthi Kumaran Ramasamy, Lokesh Rana, Chhabi Lal Ranabhat, Kumuda Rao, Sowmya J Rao, Tapash Ranjan

Rautray, Salman Rawaf, Abdul Rehman, Mina Rezaei, Chanapong Rojanaworarit, Shubhanjali Roy, Cameron John Sabet, Basema Ahmad Saddik, Umar Saeed, Vaibhav Sahni, Ambika Sahoo, Mirza Rizwan Sajid, Joseph W Sakshaug, Afeez Abolarinwa Salami, Abdallah M Samy, Mohammad Sarmadi, Shakta Mani Satyam, Christophe Schinckus, Art Schuermans, Siddharthan Selvaraj, Subramanian Senthilkumaran, Yashendra Sethi, Ahmed Nabil Shaaban, Muhammed Shabil, Sweni Shah, Sunder Sham, Muhammad Aaqib Shamim, Mohammed Shannawaz, Bhoopesh Kumar Sharma, Ravi Kumar Sharma, Vishal Sharma, Mahabalesh Shetty, Premalatha K Shetty, Aminu Shittu, Ivy Shiue, Sunil Shrestha, Dharmsheel Shrivastav, Negussie Boti Sidamo, Abhinav Singh, Amit Singh, Bhim Pratap Singh, Brij Pal Singh, Jasvinder A Singh, Prashant Kumar Singh, Gisha Sivan, Gowri Sivaramakrishnan, Valentin Yurievich Skryabin, Farrukh Sobia, Lencho Kajela Solbana, Solikhah Solikhah, Mansi Soni, Chandrashekhar T Sreeramareddy, Vipul Srivastava, Marjia Sultana, Johan Sundström, Chandan Kumar Swain, Celine Tabche, Mircea Tampa, Aigul Yelgondiyevna Tazhiyeva, Madi Tleshev, Domenico Trico, Evangelia Eirini Tsermpini, Abdul Rohim Tualeka, Himayat Ullah, Bhaskaran Unnikrishnan, Rakesh V R, Sai Mahesh Vajjala, Zahir Vally, Jef Van den Eynde, Joe Varghese, Sampara Vasishta, Vishnu Priya Veeraraghavan, Balachandar Vellingiri, Narayanaswamy Venketasubramanian, David Villarreal-Zegarra, Liang Wang, Shu Wang, Dakshitha Praneeth Wickramasinghe, Zemenu Shiferaw Shiferaw Yadita, Naohiro Yonemoto, Muhammad Sohail Zafar, Michael Zastrozhin, Mohammed G M Zeariya, Alemu Birara Zemariam, Haijun Zhang, Carl Zhou, Mohamed Ali Zoromba, Ahed H Zyoud

Developing methods or computational machinery

Aleksandr Y Aravkin, Xiaochen Dai, Emmanuela Gakidou, Gabriela Fernanda Gil, Simon I Hay, Ali H Mokdad, Brooks W Morgan, Christopher J L Murray

Providing critical feedback on methods or results

Bhoomadevi A, Rashad Abdul-Ghani, Rizwan Suliankatchi Abdulkader, Parisa Abedi, Olugbenga Olusola Abiodun, Nagah M. Abourashed, Dariush Abtahi, Rana Kamal Abu-Farha, Ahmed Abu-Zaid, Fuad Hamdi A. Abuadas, Mohammad H Abuadas, Anirudh Balakrishna Acharya, Apurba Acharya, Swetha Acharya, Babatope Oluwadamilare Adebiyi, Miracle Ayomikun Adesina, Usha Adiga, Aanuoluwapo Adeyimika Afolabi, Muhammad Sohail Afzal, Feleke Doyore Agide, Aqeel Ahmad, Khabir Ahmad, Khurshid Ahmad, Sajjad Ahmad, Ayman Ahmed, MD. Faisal Ahmed, Naveed Ahmed, Shahzaib Ahmed, Akil Akhtar, Tomi Akinyemiju, Omar Al Omari, Mohammad Al Qadire, Yazan Al Thaher, Mohammad Ahmmad Mahmoud Al Zoubi, Mohammed Sultan Al-Ak'hali, Rasmieh Mustafa Al-Amer, Shams Moh'd Fares Khalaf Al-Anzi, Ahmad M Al-Bashaireh, Sabah Al-Marwani, Ahmad Rajeh Al-Qudimat, Ali Salman Al-Shami, Yaser Mohammed Al-Worafi, Abdulmohsen Hamdan Al-Zalabani, Adel S Al-Zubairi, Timothy Olukunle Aladelusi, Mohammad Khursheed Alam, Turki M Alanzi, Fahad D Algahtani, Abdelazeem M Algammal, Khalid F Alhabib, Amidu Alhassan, Ashraf Alhumaidi, Inayat Ali, Irfan Ali, Kamran Ali, Mohammad Daud Ali, Rafat Ali, Syed Shujait Ali, Syed Mohamed Aljunid, Sameer Abdulmalik Alkubati, Wesam Taher Almagharbeh, Mohammed J. Almalki, Margret Beaula Alocious Sukumar, Mahmoud A Alomari, Jaber S Alqahtani, Mohammad R Alqudimat, Ahmed Yaseen Alqutaibi, Mohammed Amjed Alsaegh, Najim Z Alshahrani, Zaid Altaany, Awais Altaf, Hassan Alwafi, Hany Aly, Mohammad Sharif Ibrahim Alyahya, Karem H Alzoubi, Tarek Tawfik Amin, Etsay Woldu Anbesu, Syam S. Andra, A.H. Sruthi Anil Kumar, Kabilan Annadurai, Saeid Anvari, Razique Anwer, Gururaj Arakeri, Harshit Arora, Mohammad Ashfaque, Tahira Ashraf, Muhammad Shahzad Aslam, Yuni Asri, Omer Atac, Seyyed Shamsadin Athari, Samuel Babajide Atoyebi, Alok Atreya, Avinash Aujayeb, Manal Awad, Kemal Lemnuro Awol, Ahmad Ayed, Syed Babar Jamal Bacha, Ashish D Badiye, Riadh Badraoui, Youngoh Bae, Nasser Bagheri, Khlood K Baghlaf,

Abdul Rasheed Bahar, Yasemin Bahar, Razieh Bahreini, Parvathy Balachandran, Raju Balaji, Yatan Pal Singh Balhara, Biswajit Banik, Hiba Jawdat Barqawi, Amadou Barrow, Fadi Basha, MD Abu Bashar, Almaz Nibret Belay, Uzma Iqbal Belgaumi, Abdulrahman Babatunde Bello, Ibrahim Olajide Bello, Kelemu Zelalem Berhanu, Pankaj Bhardwaj, Sonu Bhaskar, Sudip Bhattacharya, Bijit Biswas, Eyob Ketema Bogale, Archith Boloor, Dejana Braithwaite, Zahid A Butt, Senthilkumar C, Zhong Cao, Márcia Carvalho, Sandra Carvalho, Joao Mauricio Castaldelli-Maia, Rama Mohan Chandika, Vijay Kumar Chattu, Sirshendu Chaudhuri, Akhilanand Chaurasia, Snehal Prakash Chavhan, Meng Xuan Chen, Saravanan Chinnaiyan, Bryan Chong, Sonali Gajanan Choudhari, Dinh-Toi Chu, Isaac Sunday Chukwu, Sunghyun Chung, Alexandru Corlateanu, Natalia Cruz-Martins, Omid Dadras, Namrata Dagli, Xiaochen Dai, Gloria Dalla Costa, Lalit Dandona, Rakhi Dandona, Samuel Demissie Darcho, Yagnik Dave, Ismail Dergaa, Vinoth Gnana Chellaiyan Devanbu, Syed Masudur Rahman Dewan, Arkadeep Dhali, Amol S Dhane, Bibha Dhungel, Kimia Didehvar, Hoa Thi Do, Ojas Prakashbhai Doshi, Jiang Du, Emeka W. Dumbili, Senbagam Duraisamy, Siddhartha Dutta, Jasmin Dönicke, Kristina Edvardsson, Michael Ekholuenetale, Suhaib El Khatib, Iman El Sayed, Ashraf A El-Metwally, Mohamed Ahmed Eladl, Omar Abdelsadek Abdou Elmeligy, Abdelgawad Salah Eltahawy, Collins Nduka Esomchi, Devaraj Ezhilarasan, Adeniyi Francis Fagbamigbe, Ali Faramarzi, Mohammad Fareed, Carla Sofia e Sá Farinha, Munazza Fatima, Feten Fekih-Romdhane, Anteneh Fikrie, Filippos T Filippidis, Florian Fischer, James L Fisher, Luisa S Flor, Muktar A Gadanya, Márió Gajdács, Emmanuela Gakidou, Balasankar Ganesan, Balasubramanian Ganesh, Shivaprakash Gangachannaiah, Fernando Jr. Barroga Garcia, Rupesh K Gautam, Haftay Gebremedhin, Mizan Habtemichael Gebresilassie, Khalid Yaser Ghailan, Lobna Gharaibeh, Sailaja Ghimire, Ehsan Gholami, Zainab Gholami, Thinh Gia Gia Nguyen, Artyom Urievich Gil, Gabriela Fernanda Gil, Divya Gopinath, Michal Grivna, Bin Guan, Zenawi Hagos Gufue, Damitha Asanga Gunawardane, Sasidhar Gunturu, Anish Kumar Gupta, Bhawna Gupta, Himanshu Gupta, Rajeev Gupta, Sunanda Gupta, Yonas Deressa Guracho, Gamze Gökulu, Parham Habibzadeh, Yohannes Gebreegziabher Haile, Ferdous Hakim, Esam Halboub, Nasrin Hanifi, Arief Hargono, Ahmed I Hasaballah, Faizul Hasan, Ikramul Hasan, Towhid Hasan, Ahmad Shoeb Hashmi, Md Saquib Hasnain, Simon I Hay, Behzad Heibati, Mohammad Heidari, Zaw Myo Hein, Mohamed Fawzi Hemida, Brenda Yuliana Herrera-Serna, Yuta Hiraike, Ashley Mark Hopkins, Lubna Hossain, Sahadat Hossain, Mehdi Hosseinzade, Weijun Huang, Javid Hussain, Bing-Fang Hwang, Luigi Francesco Iannone, Segun Emmanuel Ibitoye, Olayinka Stephen Ilesanmi, Irena M Ilic, Milena D Ilic, Mohammad Tarique Imam, Mustapha Immurana, Lalu Muhammad Irham, M Mofizul Islam, Farhad Islami, Juan S Izquierdo-Condoy, Jaison Jacob, Abhishek Jaiswal, Mihajlo Jakovljevic, Rajiv Janardhanan, Ruwan Duminda Jayasinghe, Achala Upendra Jayatilleke, Gwang Hun Jeong, Bijay Mukesh Jeswani, Zixiang Ji, Shuai Jin, Jobin Jose, Alex Joseph, Nitin Joseph, Charity Ehimwenma Joshua, Hema Shree K, Billingsley Kaambwa, Zubair Kabir, Vidya Kadashetti, Dler Hussein Kadir, Pradnya Vishal Kakodkar, Ebbie Kalan, Mehnaz Kamal, Mona Kanaan, Jiseung Kang, Kehinde Kazeem Kanmodi, Surya Kant, Neeti Kapoor, Shalini Kapoor, Debasish Kar, Sujita Kumar Kar, Neila Karagezova, Mohmed Isaqali Karobari, Manoj Kumar Kashyap, Chukwudi Keke, Asaad Khalid, Maseer Khan, Moien AB Khan, Muhammad Arslan Khan, Muhammad Umer Khan, Sumaiya Khan, Zahid Khan, Zelal Kharaba, Sameer Uttamaro Khasbage, Samira Khoshvaght, Mahmood Khosrowjerdi, P Ratan Khuman, Marta A. Kisiel, Shivakumar KM, Prakash Babu Kodali, Oleksii Korzh, Archana Koul, Sindhura Lakshmi Koulmane Laxminarayana, Kewal Krishan, Abhishek Krishna, Deepak Krishnamurthy, Nitya Krishnasamy, Pranav S Kshtriya, Chong-Han Kua, Garry Kuan, Claudia E. Kuehni, Omar Kujan, ASHOK KUMAR, Dhasarathi Kumar, Diwakar Kumar, G Anil Kumar, Laksh Kumar, Mohan Kumar, Nithin Kumar, Pawan Kumar, Pradeep Kumar, V Udaya Kumar, Om P Kurmi, Maria Dyah Kurniasari, Krishna Prasad Kurpad, Taeho Kwak, Chandrakant Lahariya, Hanpeng Lai, Timo

Lajunen, Yilma Markos Larebo, Savita Lasrado, Munjae Lee, Seung Won Lee, Hui Li, Jiaying Li, Qiang Li, Si Li, Xingang Li, Zhengrui Li, Yanxue Lian, Daniel Lindholm, Bingsheng Liu, Gang Liu, Jue Liu, Giancarlo Lucchetti, Abhilash Ludhiadch, Pincheng Luo, Hung N Luu, Miltiadis D Lytras, José Francisco López-Gil, Monika Machoy-Rakoczy, Ahmed A. Madfa, Samatar Abshir Mahamed, Mansour Adam Mahmoud, Kashish Malhotra, Lokesh Manjani, Farheen Mansoor, Miquel Martorell, Yasith Mathangasinghe, Medha Mathur, Neeta Mathur, Khurshid A Mattoo, Enkeleint A Mechili, John Robert Carabeo Medina, Jitendra Kumar Meena, Ravi Mehrotra, Vini Mehta, Satish Melwani, Aamir Raoof Memon, Godfred Antony Menezes, Ritesh G Menezes, Tarig H Merghani, Tomislav Mestrovic, Chamila Dinushi Kukulege Mettananda, Mohamed M. M. Metwally, Muhammad Agus Mikrajab, Giuseppe Minervini, Erkin M Mirrakhimov, Vinaytosh Mishra, Sanjeev Misra, Tridip Mitra, Farduus Ibraahim Mohamed, Nouh Saad Mohamed, Ibrahim Mohammadzadeh, Zaidan Mohammed, Syam Mohan, Nagaishwarya Moka, Ali H Mokdad, Hirbaye Mokona, Himel Mondal, Rafael Silveira Moreira, Rohith Motappa, Sumaira Mubarik, Amartya Mukhopadhyay, Malaisamy Muniyandi, Christopher J L Murray, Krishna Murti, Vignesh Murugan, David Musoke, Sathish Muthu, Kiran P. P. Nagdeo, Ganesh R Naik, Gopal Nambi, Vinay Nangia, Shumaila Nargus, Zuhair S Natto, Biswa Prakash Nayak, Kinjal Nayak, Andrew Kamsoko Ndakotsu, Cuong Tat Nguyen, Duy Cao Nguyen, Huong Lan Thi Nguyen, Trang Nguyen, Ali Nikoobar, Vikram Niranjan, Fred Nugen, Wahyu Pudji Nugraheni, Nazik Nurelhuda, Bogdan Oancea, Ramez M Odat, Ismail A Odetokun, Olusegun Olatunji Ojedoyin, Osaretin Christabel Okonji, Andrew T Olagunju, Jeby Jose Olickal, Ahmed Omar Bali, Hope Onohuean, Miracle Chinonso Oparah, Michal Ordak, Tope Oyelade, Mahesh P A, Alok Kumar Panda, Deepshikha Pande Katare, Sanjib Raj Raj Pandey, Ioannis Pantazopoulos, Shahina Pardhan, Tarang Parekh, Romil R Parikh, Arpit Parmar, Pragnesh Parmar, Sameena Parveen, Roberto Passera, Bhargav Umeshchandra Patel, Satyananda Patel, Shivani Patel, Vipin Patidar, Ashlesh Patil, Dimitrios Patoulias, Uttam Paudel, Shrikant Pawar, Saikiran Pendem, Prince Peprah, Gavin Pereira, Olumuyiwa James Peter, Tung Thanh Pham, Roman V Polibin, Ramesh Poluru, Thantrira Porntaveetus, Amit Porwal, Priyanka Porwal, Pranil Man Singh Pradhan, Elton Junio Sady Prates, Harsh Priya, Bharathi M Purohit, Jagadeesh Puvvula, Ibrahim Qattea, Raghu Anekal Radhakrishnan, Venkatraman Radhakrishnan, Yashpal Singh Raghav, Pracheth Raghuveer, Fakher Rahim, Sajjad Rahimi, Firman Suryadi Rahman, Md Ferdous Rahman, Mohammad Hifz Ur Rahman, Mosiur Rahman, Amita Rai, Sunil Kumar Raina, Jeffrey Pradeep Raj, Mahmoud Mohammed Ramadan, Majed Ramadan, Kadar Ramadhan, Shakthi Kumaran Ramasamy, Pramod W Ramteke, Lokesh Rana, Shailendra Singh Rana, Chhabi Lal Ranabhat, Kumuda Rao, Sowmya J Rao, Devarajan Rathish, Tapash Ranjan Rautray, Salman Rawaf, Abdul Rehman, Mina Rezaei, Nazila Rezaei, Ravi Rohilla, Chanapong Rojanaworarit, Himanshu Sekhar Rout, Shubhanjali Roy, Rohini Ruhil, Susanne Röhr, Cameron John Sabet, Mamta Sachdeva Dhingra, Basema Ahmad Saddik, Mohammad Reza Saeb, Umar Saeed, Dominic Sagoe, Indranil Saha, Vaibhav Sahni, Ambika Sahoo, Mirza Rizwan Sajid, Joseph W Sakshaug, Afeez Abolarinwa Salami, Mohamed A Saleh, Janmejaya Samal, Abdallah M Samy, Siddaram Shivaji Sarate, Yaser Sarikhani, Mohammad Sarmadi, Gargi Sachin Sarode, Sachin C Sarode, Mukesh Kumar Sathya Narayanan, Aanchal Satija, Shakta Mani Satyam, Ganesh Kumar Saya, Christophe Schinckus, Art Schuermans, Siddharthan Selvaraj, Mohammad H Semreen, Subramanian Senthilkumaran, Yashendra Sethi, Seyed Mohammad Seyed Alshohadaei, Ahmed Nabil Shaaban, Arash Shaban-Nejad, Muhammed Shabil, Sweni Shah, Muhammad Aaqib Shamim, Alfiya Shamsutdinova, Abhishek Shankar, Mohammed Shannawaz, Bhoopesh Kumar Sharma, Ravi Kumar Sharma, Shreya Sharma, Vishal Sharma, Somia Shehzadi, Mahdi Sheikh, Mahabalesh Shetty, Hui-Zhong Shi, Arun Shirali, SHIRINDOKHT SHIRAZI, Kawkab Shishani, Aminu Shittu, Ivy Shiue, Ambreen Shoaib, Aman Shrestha, Sunil Shrestha, Dharmsheel Shrivastav, Kerem Shuval, Negussie Boti Sidamo,

Abhinav Singh, Amit Singh, Bhim Pratap Singh, Brij Pal Singh, Jasvinder A Singh, Jawahar Singh, Prashant Kumar Singh, Ragini D Singh, Virendra Singh, Gisha Sivan, Gowri Sivaramakrishnan, Valentin Yurievich Skryabin, Farrukh Sobia, Md Salman Sohel, Lencho Kajela Solbana, Solikhah Solikhah, Mansi Soni, Joan B Soriano, Chandrashekhar T Sreeramareddy, Vipul Srivastava, Marjia Sultana, Zhong Sun, Shanthosh Priyan Sundaram, Johan Sundström, Jithin Surendran, Chandan Kumar Swain, Celine Tabche, Mohammad Tabish, Iman M Talaat, Avni Tambi, Mircea Tampa, Benjamin Kye Jyn Tan, Ingan Ukur Tarigan, Aigul Yelgondiyevna Tazhiyeva, Masayuki Teramoto, Kavumpurathu Raman Thankappan, Rekha Thapar, Madi Tleshev, Domenico Trico, Evangelia Eirini Tsermpini, Himayat Ullah, Bhaskaran Unnikrishnan, Abdulkadir Usman Sambo, Berna Uzun, Hande Uzunçıbuk, Rakesh V R, Sai Mahesh Vajjala, Zahir Vally, Jef Van den Eynde, Joe Varghese, Siddhartha Alluri Varma, Sampara Vasishta, Vishnu Priya Veeraraghavan, Balachandar Vellingiri, U Venkatesh, Narayanaswamy Venketasubramanian, Vignesh Vikram, David Villarreal-Zegarra, Liang Wang, Shu Wang, Yaojie Wang, Fei-Long Wei, Dakshitha Praneeth Wickramasinghe, Nuwan Darshana Wickramasinghe, Shulan Wu, Hong Xiao, Wanqing Xie, Zemenu Shiferaw Shiferaw Yadita, Kaiqi Yang, Renjulal Yesodharan, Biksegn Asrat Asrat Yirdaw, Arzu Yiğit, Vahit Yiğit, S Yogeshkumar, Dong Keon Yon, Naohiro Yonemoto, Monal Yuwanati, Muhammad Sohail Zafar, Emilia Zainal Abidin, Fathiah Zakham, Hamed Zandian, Michael Zastrozhin, Saad Zbiri, Mohammed G M Zeariya, Alemu Birara Zemariam, Eyael M Zeru, Haijun Zhang, Zhiqiang Zhang, Lei Zheng, Ming-Hua Zheng, Carl Zhou, Mohamed Ali Zoromba, Ahed H Zyoud

Drafting the work or revising it critically for important intellectual content

Bhoomadevi A, Hasan Aalruz, Rashad Abdul-Ghani, Olugbenga Olusola Abiodun, Olumide Abiodun, Nagah M. Abourashed, Rana Kamal Abu-Farha, Ahmed Abu-Zaid, Fuad Hamdi A. Abuadas, Mohammad H Abuadas, Anirudh Balakrishna Acharya, Apurba Acharya, Swetha Acharya, Babatope Oluwadamilare Adebiyi, Juliana Bunmi Adetunji, Usha Adiga, Aanuoluwapo Adeyimika Afolabi, Muhammad Sohail Afzal, Feleke Doyore Agide, Khabir Ahmad, Ayman Ahmed, Naveed Ahmed, Shahzaib Ahmed, Sara Akeel, Akil Akhtar, Tomi Akinyemiju, Omar Al Omari, Yazan Al Thaher, Mohammad Ahmmad Mahmoud Al Zoubi, Mohammed Sultan Al-Ak'hali, Rasmieh Mustafa Al-Amer, Shams Moh'd Fares Khalaf Al-Anzi, Ahmad Rajeh Al-Qudimat, Ali Salman Al-Shami, Yaser Mohammed Al-Worafi, Abdulmohsen Hamdan Al-Zalabani, Adel S Al-Zubairi, Timothy Olukunle Aladelusi, Mohammed S Aldossary, Raouf Alebshehy, Aqeel M Alenazi, Fahad D Algahtani, Abdelazeem M Algammal, Khalid F Alhabib, Fadwa Naji Alhalaiqa, Amidu Alhassan, Ashraf Alhumaidi, Inayat Ali, Kamran Ali, Syed Shujait Ali, Syed Mohamed Aljunid, Sameer Abdulmalik Alkubati, Wesam Taher Almagharbeh, Margret Beaula Alocious Sukumar, Mahmoud A Alomari, Jaber S Alqahtani, Sahel Majed Alrousan, Mohammed Amjed Alsaegh, Najim Z Alshahrani, Zaid Altaany, Awais Altaf, Hassan Alwafi, Hany Aly, Mohammad Sharif Ibrahim Alyahya, Karem H Alzoubi, Revati Amin, Tarek Tawfik Amin, Abhishek Anil, Prapti Anjana, Kabilan Annadurai, Saeid Anvari, Gururaj Arakeri, Monika Arora, Umesh Raj Aryal, Mohammad Ashfaque, Muhammad Shahzad Aslam, Yuni Asri, Anil Raj Assariparambil, Omer Atac, Seyyed Shamsadin Athari, Samuel Babajide Atoyebi, Alok Atreya, Avinash Aujayeb, Manal Awad, Ahmad Ayed, Syed Babar Jamal Bacha, Ashish D Badiye, Riadh Badraoui, Youngoh Bae, Abdul Rasheed Bahar, Yasemin Bahar, Parvathy Balachandran, Yatan Pal Singh Balhara, Hiba Jawdat Barqawi, Amadou Barrow, MD Abu Bashar, Uzma Iqbal Belgaumi, Abdulrahman Babatunde Bello, Ibrahim Olajide Bello, Kelemu Zelalem Berhanu, Sonu Bhaskar, ASHOK KUMAR BHATI, Sudip Bhattacharya, Bijit Biswas, Archith Boloor, Dejana Braithwaite, Senthilkumar C, Sanjay C J, Zhong Cao, Felix Carvalho, Márcia Carvalho, Sandra Carvalho, Joao Mauricio Castaldelli-Maia, Maria Sofia Cattaruzza, Sandip Chakraborty, Rama Mohan Chandika, Vijay Kumar Chattu, Sirshendu Chaudhuri, Akhilanand Chaurasia, Snehal Prakash Chavhan, Bryan Chong, Sunghyun Chung, Alexandru Corlateanu,

Claudia Cosma, Natalia Cruz-Martins, Namrata Dagli, Xiaochen Dai, Gloria Dalla Costa, Mouni Damineni, Samuel Demissie Darcho, Yagnik Dave, Marco Del Riccio, Ismail Dergaa, Vinoth Gnana Chellaiyan Devanbu, Syed Masudur Rahman Dewan, Arkadeep Dhali, Amol S Dhane, Kimia Didehvar, Hoa Thi Do, Pranavi Dohare, Sushil Dohare, Cátia Domingues, Ojas Prakashbhai Doshi, Jiang Du, Emeka W. Dumbili, Senbagam Duraisamy, Siddhartha Dutta, Jasmin Dönicke, Michael Ekholuenetale, Suhaib El Khatib, Iman El Sayed, Ashraf A El-Metwally, Omar Abdelsadek Abdou Elmeligy, Abdelgawad Salah Eltahawy, Collins Nduka Esomchi, Elochukwu Ezenwankwo, Devaraj Ezhilarasan, Adeniyi Francis Fagbamigbe, Ali Faramarzi, Mohammad Fareed, Munazza Fatima, Feten Fekih-Romdhane, Nuno Ferreira, Anteneh Fikrie, Filippos T Filippidis, Florian Fischer, James L Fisher, Luisa S Flor, Muktar A Gadanya, Márió Gajdács, Emmanuela Gakidou, Balasankar Ganesan, Balasubramanian Ganesh, Shivaprakash Gangachannaiah, Rupesh K Gautam, Haftay Gebremedhin, Lobna Gharaibeh, Sailaja Ghimire, Ehsan Gholami, Zainab Gholami, Thinh Gia Gia Nguyen, Artyom Urievich Gil, Gabriela Fernanda Gil, Divya Gopinath, Michal Grivna, Zenawi Hagos Gufue, Damitha Asanga Gunawardane, Sasidhar Gunturu, Bhawna Gupta, Himanshu Gupta, Rajeev Gupta, Sunanda Gupta, Gamze Gökulu, Parham Habibzadeh, Yohannes Gebreegziabher Haile, Ferdous Hakim, Esam Halboub, Sajid Hameed, Nasrin Hanifi, Ahmed I Hasaballah, Faizul Hasan, Shamimul Hasan, Towhid Hasan, Nada Tawfig Hashim, Ahmad Shoeb Hashmi, Md Saquib Hasnain, Nageeb Hassan, Simon I Hay, Behzad Heibati, Zaw Myo Hein, Mohamed Fawzi Hemida, Brenda Yuliana Herrera-Serna, Yuta Hiraike, Lubna Hossain, Sahadat Hossain, Mehdi Hosseinzade, Weijun Huang, Luigi Francesco Iannone, Segun Emmanuel Ibitoye, Olayinka Stephen Ilesanmi, Irena M Ilic, Milena D Ilic, Mustapha Immurana, Lalu Muhammad Irham, CV Irshad, Farhad Islami, Juan S Izquierdo-Condoy, Abhishek Jaiswal, Mihajlo Jakovljevic, Qazi Mohammad Sajid Jamal, Rajiv Janardhanan, Ruwan Duminda Jayasinghe, Achala Upendra Jayatilleke, Gwang Hun Jeong, Bijay Mukesh Jeswani, Shuai Jin, Jobin Jose, Nitin Joseph, Charity Ehimwenma Joshua, Hema Shree K, Vaishali K, Vidya Kadashetti, Pradnya Vishal Kakodkar, Mona Kanaan, Jiseung Kang, Kehinde Kazeem Kanmodi, Surya Kant, Neeti Kapoor, Shalini Kapoor, Debasish Kar, Mohmed Isaqali Karobari, Manoj Kumar Kashyap, Chukwudi Keke, Asaad Khalid, Maseer Khan, Moien AB Khan, Muhammad Umer Khan, Serab Khan, Sumaiya Khan, Zahid Khan, Zelal Kharaba, Sameer Uttamaro Khasbage, Samira Khoshvaght, Mahmood Khosrowjerdi, Min Seo Kim, Marta A. Kisiel, Shivakumar KM, Prakash Babu Kodali, Oleksii Korzh, Sindhura Lakshmi Koulmane Laxminarayana, Kewal Krishan, Pranav S Kshtriya, Chong-Han Kua, Garry Kuan, Claudia E. Kuehni, Mukhtar Kulimbet, Diwakar Kumar, Laksh Kumar, Mohan Kumar, Pawan Kumar, Pradeep Kumar, Rakesh Kumar, Tushar Kumar, V Udaya Kumar, Om P Kurmi, Maria Dyah Kurniasari, Krishna Prasad Kurpad, Taeho Kwak, Chandrakant Lahariya, Hanpeng Lai, Agung Dwi Laksono, Yilma Markos Larebo, Savita Lasrado, Hui Li, Jiaying Li, Qiang Li, Si Li, Xingang Li, Zhengrui Li, Yanxue Lian, Daniel Lindholm, Bingsheng Liu, Jue Liu, Giancarlo Lucchetti, Abhilash Ludhiadch, Pincheng Luo, Hung N Luu, Miltiadis D Lytras, José Francisco López-Gil, Monika Machoy-Rakoczy, Samatar Abshir Mahamed, Kashish Malhotra, Lokesh Manjani, Miquel Martorell, Yasith Mathangasinghe, Medha Mathur, Khurshid A Mattoo, Oliver J McElvaney, Enkeleint A Mechili, John Robert Carabeo Medina, Jitendra Kumar Meena, Vini Mehta, Aamir Raoof Memon, Godfred Antony Menezes, Ritesh G Menezes, Tarig H Merghani, Tomislav Mestrovic, Chamila Dinushi Kukulege Mettananda, Mohamed M. M. Metwally, Muhammad Agus Mikrajab, Tridip Mitra, Farduus Ibraahim Mohamed, Nouh Saad Mohamed, Zaidan Mohammed, Nagaishwarya Moka, Ali H Mokdad, Hirbaye Mokona, Himel Mondal, Rafael Silveira Moreira, Brooks W Morgan, Rohith Motappa, Amartya Mukhopadhyay, Malaisamy Muniyandi, Christopher J L Murray, Vignesh Murugan, David Musoke, Sathish Muthu, Karikalan Nagarajan, Kiran P. P. Nagdeo, Gopal Nambi, Shumaila Nargus, Mohammad Zakaria Nassani, Zuhair S Natto, Biswa Prakash Nayak, Kinjal Nayak,

Andrew Kamsoko Ndakotsu, Sabina Onyinye Nduaguba, Samata Nepal, Marie Ng, Cuong Tat Nguyen, Duy Cao Nguyen, Huong Lan Thi Nguyen, Trang Nguyen, Vikram Niranjan, Fred Nugen, Wahyu Pudji Nugraheni, Nazik Nurelhuda, Bogdan Oancea, Ramez M Odat, Fabio Massimo Oddi, Ismail A Odetokun, Olusegun Olatunji Ojedoyin, Osaretin Christabel Okonji, Andrew T Olagunju, Jeby Jose Olickal, Ahmed Omar Bali, Hope Onohuean, Miracle Chinonso Oparah, Michal Ordak, Augustus Osborne, Tope Oyelade, Mahesh P A, Alicia Padron-Monedero, Raul Felipe Palma-Alvarez, Alok Kumar Panda, Deepshikha Pande Katare, Ashok Pandey, Ioannis Pantazopoulos, Shahina Pardhan, Romil R Parikh, Sameena Parveen, Roberto Passera, Satyananda Patel, Shivani Patel, Vipin Patidar, Ashlesh Patil, Dimitrios Patoulias, Uttam Paudel, Shrikant Pawar, Gavin Pereira, Olumuyiwa James Peter, Edoardo Pirera, Thantrira Porntaveetus, Amit Porwal, Priyanka Porwal, Pranil Man Singh Pradhan, Elton Junio Sady Prates, Harsh Priya, Bharathi M Purohit, Jagadeesh Puvvula, Ibrahim Qattea, Venkatraman Radhakrishnan, Pracheth Raghuveer, Fakher Rahim, Sajjad Rahimi, Firman Suryadi Rahman, Mohammad Hifz Ur Rahman, Amita Rai, Sunil Kumar Raina, Jeffrey Pradeep Raj, Mahmoud Mohammed Ramadan, Kadar Ramadhan, Shakthi Kumaran Ramasamy, Lokesh Rana, Chhabi Lal Ranabhat, Kumuda Rao, Sowmya J Rao, Devarajan Rathish, Tapash Ranjan Rautray, Salman Rawaf, Abdul Rehman, Mina Rezaei, Ravi Rohilla, Chanapong Rojanaworarit, Shubhanjali Roy, Rohini Ruhil, Susanne Röhr, Chandan S N, Cameron John Sabet, Mamta Sachdeva Dhingra, Basema Ahmad Saddik, Umar Saeed, Dominic Sagoe, Indranil Saha, Amirhossein Sahebkar, Vaibhav Sahni, Ambika Sahoo, Mirza Rizwan Sajid, Joseph W Sakshaug, Afeez Abolarinwa Salami, Abdallah M Samy, Aswini Saravanan, Yaser Sarikhani, Mohammad Sarmadi, Gargi Sachin Sarode, Sachin C Sarode, Mukesh Kumar Sathya Narayanan, Aanchal Satija, Shakta Mani Satyam, Ganesh Kumar Saya, Art Schuermans, Siddharthan Selvaraj, Mohammad H Semreen, Subramanian Senthilkumaran, Yashendra Sethi, Seyed Mohammad Seyed Alshohadaei, Ahmed Nabil Shaaban, Arash Shaban-Nejad, Muhammed Shabil, Sweni Shah, Muhammad Aaqib Shamim, Alfiya Shamsutdinova, Abhishek Shankar, Manoj Sharma, Vishal Sharma, Somia Shehzadi, Mahdi Sheikh, Mahabalesh Shetty, Pavanchand Shetty, Premalatha K Shetty, Arun Shirali, SHIRINDOKHT SHIRAZI, Kawkab Shishani, Aminu Shittu, Ambreen Shoaib, Aman Shrestha, Sunil Shrestha, Dharmsheel Shrivastav, Kerem Shuval, Negussie Boti Sidamo, Abhinav Singh, Bhim Pratap Singh, Brij Pal Singh, Jasvinder A Singh, Poornima Suryanath Singh, Ragini D Singh, Surjit Singh, Gowri Sivaramakrishnan, Valentin Yurievich Skryabin, Farrukh Sobia, Lencho Kajela Solbana, Solikhah Solikhah, Mansi Soni, Joan B Soriano, Michele Sorrentino, Chandrashekhar T Sreeramareddy, Vipul Srivastava, Paschalis Steiropoulos, Marjia Sultana, Zhong Sun, Johan Sundström, Celine Tabche, Mohammad Tabish, Iman M Talaat, Avni Tambi, Mircea Tampa, Benjamin Kye Jyn Tan, Aigul Yelgondiyevna Tazhiyeva, Gebremaryam Temesgen, Reem Temsah, Thang Huu Tran, Domenico Trico, Evangelia Eirini Tsermpini, Himayat Ullah, Brigid Unim, Bhaskaran Unnikrishnan, Abdulkadir Usman Sambo, Sai Mahesh Vajjala, Zahir Vally, Jef Van den Eynde, Joe Varghese, Siddhartha Alluri Varma, Sampara Vasishta, Vishnu Priya Veeraraghavan, Balachandar Vellingiri, U Venkatesh, Narayanaswamy Venketasubramanian, Akshaya Kumar Verma, Vignesh Vikram, David Villarreal-Zegarra, Liang Wang, Shu Wang, Yaojie Wang, Fei-Long Wei, Dakshitha Praneeth Wickramasinghe, Nuwan Darshana Wickramasinghe, Faustine Williams, Hong Xiao, Zemenu Shiferaw Shiferaw Yadita, Renjulal Yesodharan, Arzu Yiğit, Vahit Yiğit, S Yogeshkumar, Dong Keon Yon, Naohiro Yonemoto, Monal Yuwanati, Muhammad Sohail Zafar, Emilia Zainal Abidin, Fathiah Zakham, Hamed Zandian, Michael Zastrozhin, Mohammed G M Zeariya, Alemu Birara Zemariam, Carl Zhou, Mohamed Ali Zoromba, Ahed H Zyoud

Managing the estimation or publications process

Emmanuela Gakidou, Simon I Hay, Christopher J L Murray
